# Supplementary material for: NiH-catalyzed anti-Markovnikov hydroamidation of unactivated alkenes with 1,4,2-dioxazol-5-ones for the direct synthesis of N-alkyl amides
Source: Commun Chem. 2022 Dec 22;5:176. doi: 10.1038/s42004-022-00791-4 (PMC9814879; doi:10.1038/s42004-022-00791-4)
Supplement: Supplementary file 2 — Supporting information [file 42004_2022_791_MOESM2_ESM.pdf]

Supporting Information

NiH-Catalyzed Curtin-Hammett Type anti-Markovnikov Hydroamidation of Terminal Alkenes with 1,4,2-Dioxazol-5-ones

Bingnan Du, Chun-Ming Chan, Yuxin Ouyang, Kalok Chan, Zhengyang Lin,* Wing-Yiu Yu*

E-mail: wing-yiu.yu@polyu.edu.hk

Supplementary Methods

1. General Information ········ 2

2. Full Substrate and Product Scope ········ 3

   2.1 Scope of dioxazolones and amides ········ 3

   2.2 Scope of alkenes/alkynes and amides ········ 8

   2.3 Exceptional examples ········ 15

3. Preparation of Dioxazolones ········ 16

4. Preparation of Alkenes ········ 24

5. Preparation of **117** and DBpin ········ 31

6. General Procedures for Ni-catalyzed Hydroamidation ········ 32

7. Reaction Optimization ········ 60

8. Mechanistic Studies ········ 65

   8.1 Reactions in the presence of radical scavengers ········ 65

   8.2 Hydroboration/amidation ········ 66

   8.3 Deuterium labeling studies ········ 68

9. Supplementary References ········ 76

1. General Information

All the solvents and reagents were obtained from commercial sources and used without purification unless stated otherwise. All glasswares were dried overnight at 150 °C prior to use. All reactions were performed in a glovebox. Thin layer chromatography (TLC) was performed on silica gel plates. Visualization on TLC was performed by UV light (254 nm) irradiation or treatment with aqueous potassium permanganate solution followed by heating. Flash column chromatography was performed on a silica gel (Merck, 230-400 mesh) column. ^{1}H and ^{13}C NMR spectra were recorded on a Brüker DPX-400 MHz spectrometer. The chemical shift (δ) values are given in ppm and are referenced to residual solvent peaks. Coupling constants (J) were reported in hertz (Hz). The NMR yield values were determined with dibromomethane as internal standard, which shows a singlet signal at δ_H 4.9 ppm in $CDCl_3$. Mass spectra and high-resolution mass spectra (HRMS) were obtained on a VG MICROMASS Fison VG platform, a Finnigan Model Mat 95 ST instrument, or a Brüker APEX 47e FT-ICR mass spectrometer. X-ray crystallographic study was performed by a Brüker CCD area detector diffractometer.

2. Full Substrate and Product Scope

2.1 Scope of dioxazolones and amides

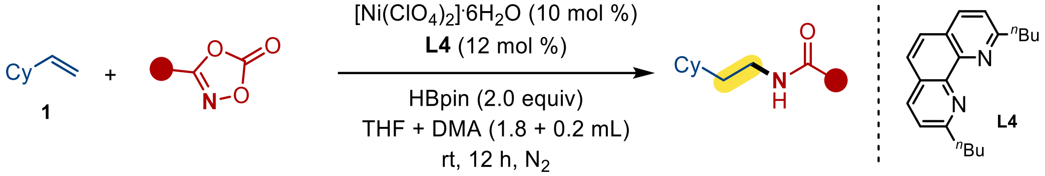

Alkene	Dioxazolone	Linear *N*-alkyl amide[a]
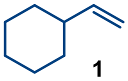 1	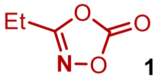 113	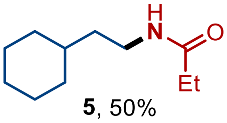 **5**, 50%
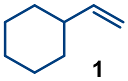 1	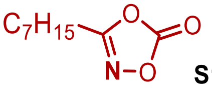 S1	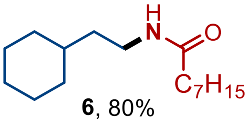 **6**, 80%
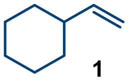 1	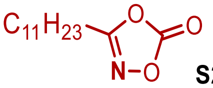 S2	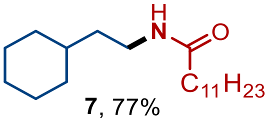 **7**, 77%
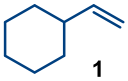 1	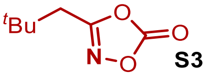 S3	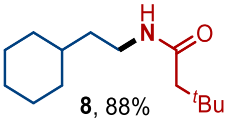 **8**, 88%
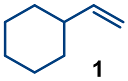 1	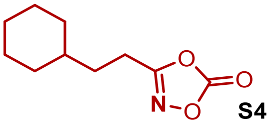 S4	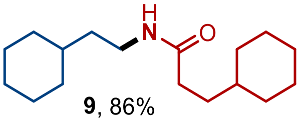 **9**, 86%
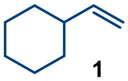 1	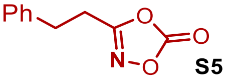 S5	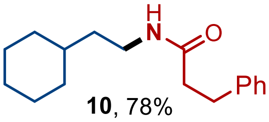 **10**, 78%
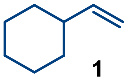 1	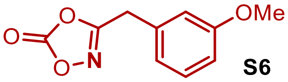 S6	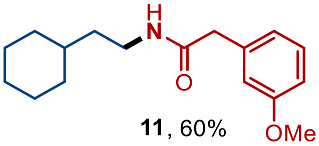 **11**, 60%
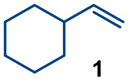 1	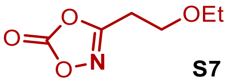 S7	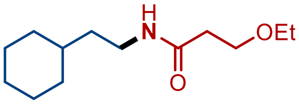 **12**, 62%
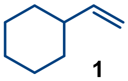 1	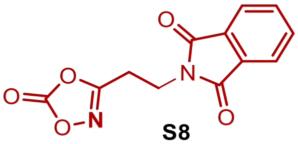 S8	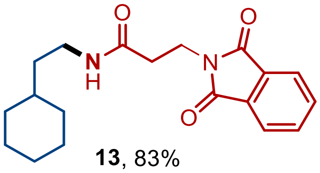 **13**, 83%

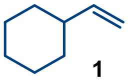 **1**	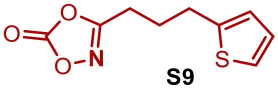 **S9**	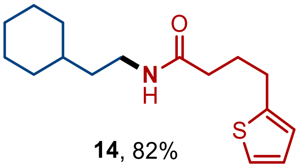 **14**, 82%
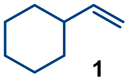 **1**	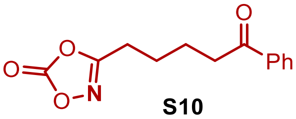 **S10**	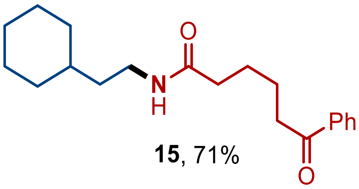 **15**, 71%
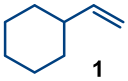 **1**	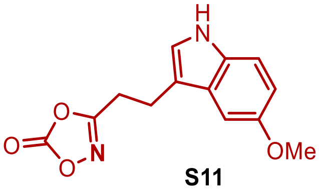 **S11**	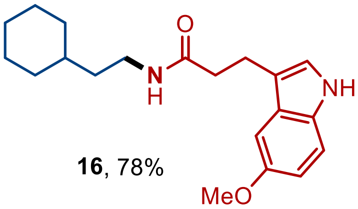 **16**, 78%
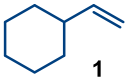 **1**	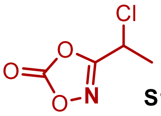 **S12**	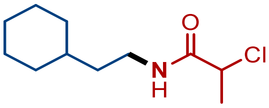 **17**, 71%
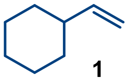 **1**	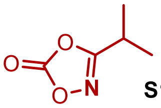 **S13**	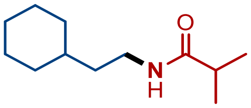 **18**, 89%
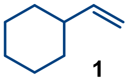 **1**	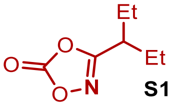 **S14**	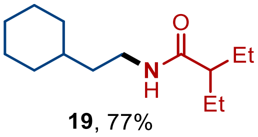 **19**, 77%
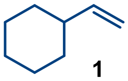 **1**	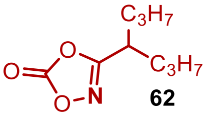 **62**	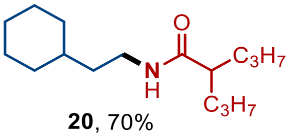 **20**, 70%
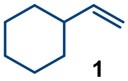 **1**	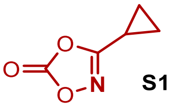 **S15**	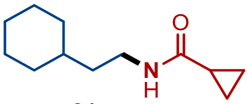 **21**, 70%
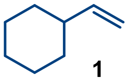 **1**	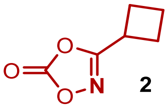 **2**	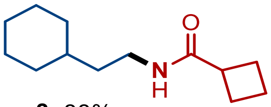 **3**, 88%
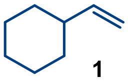 **1**	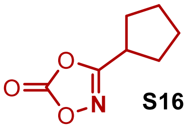 **S16**	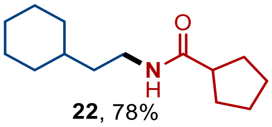 **22**, 78%

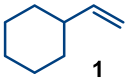 **1**	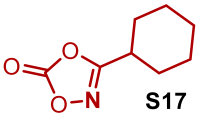 **S17**	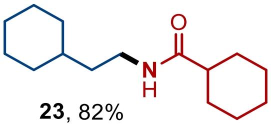 **23**, 82%
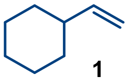 **1**	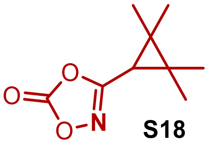 **S18**	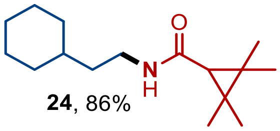 **24**, 86%
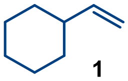 **1**	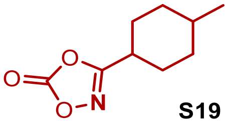 **S19**	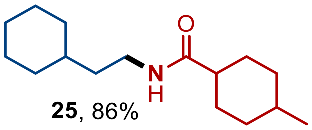 **25**, 86%
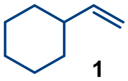 **1**	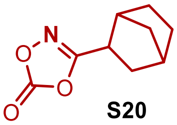 **S20**	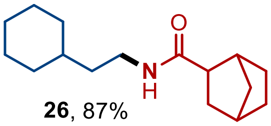 **26**, 87%
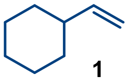 **1**	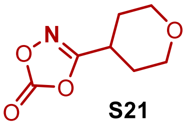 **S21**	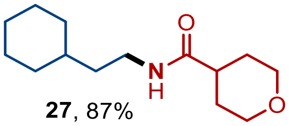 **27**, 87%
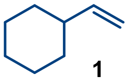 **1**	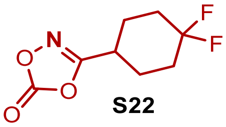 **S22**	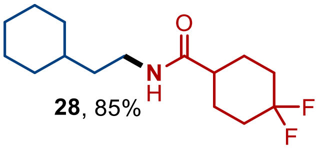 **28**, 85%
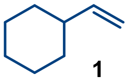 **1**	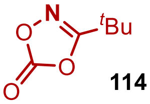 **114**	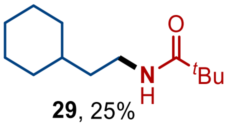 **29**, 25%
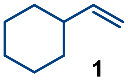 **1**	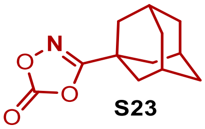 **S23**	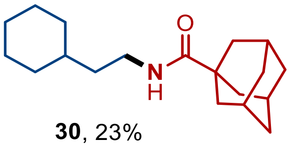 **30**, 23%
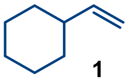 **1**	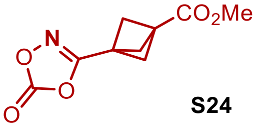 **S24**	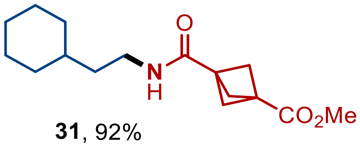 **31**, 92%
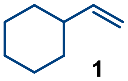 **1**	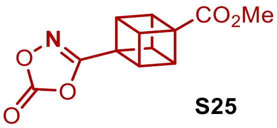 **S25**	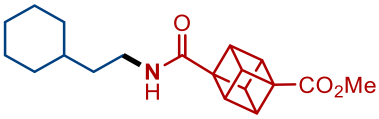 **32**, 70%
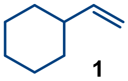 **1**	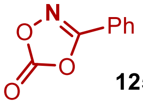 **125**	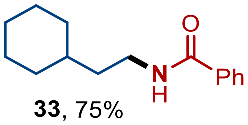 **33**, 75%

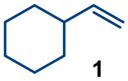 **1**	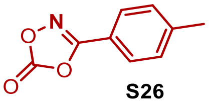 **S26**	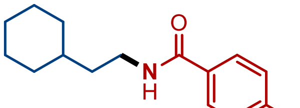 **34**, 82%
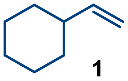 **1**	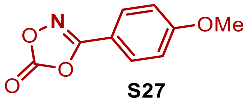 **S27**	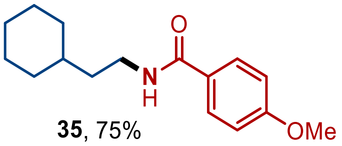 **35**, 75%
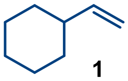 **1**	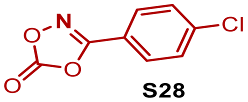 **S28**	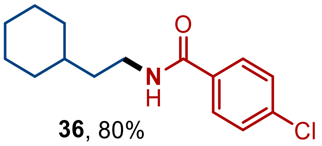 **36**, 80%
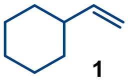 **1**	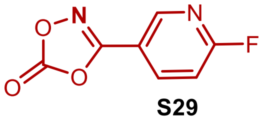 **S29**	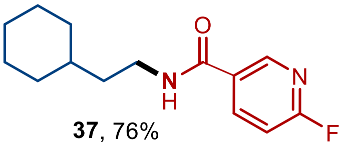 **37**, 76%
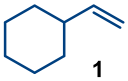 **1**	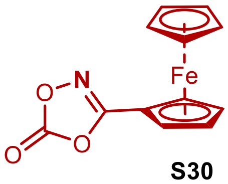 **S30**	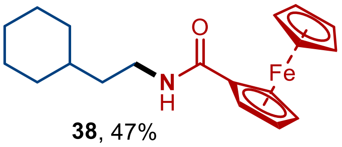 **38**, 47%
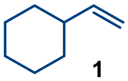 **1**	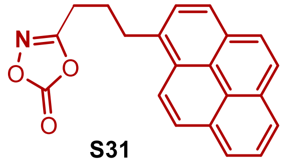 **S31**	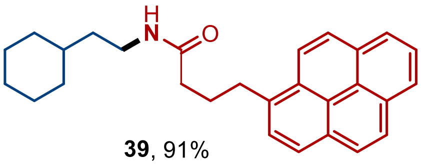 **39**, 91%
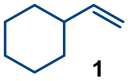 **1**	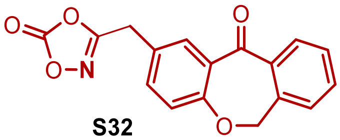 **S32**	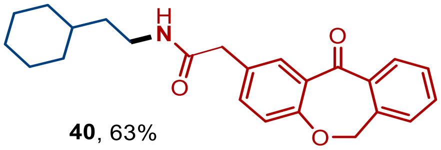 **40**, 63%
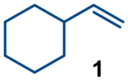 **1**	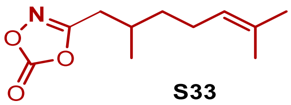 **S33**	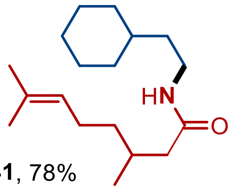 **41**, 78%
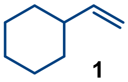 **1**	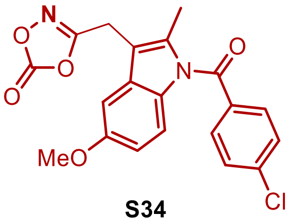 **S34**	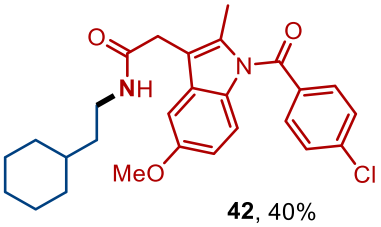 **42**, 40%

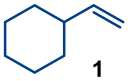 **1**	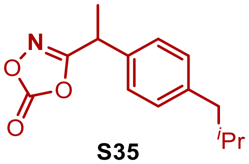 **S35**	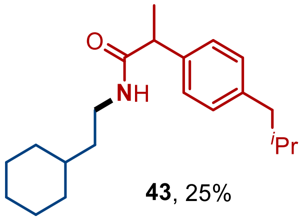 **43**, 25%
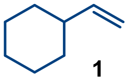 **1**	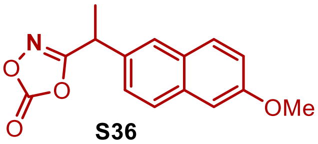 **S36**	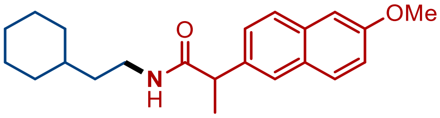 **44**, 36%
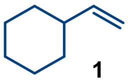 **1**	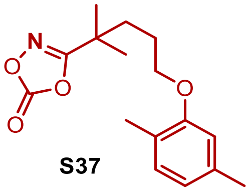 **S37**	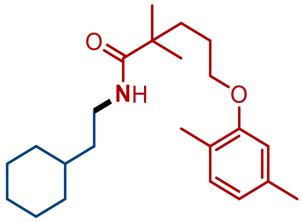 **45**, 8%

[a]Isolated yields. Reaction conditions: **1** (0.2 mmol), dioxazolones (0.4 mmol), $[Ni(ClO_4)_2]\cdot 6H_2O$ (10 mol %), **L4** (12 mol %), HBpin (2.0 equiv) and THF + DMA (1.8 + 0.2 mL) in N_2 at room temperature for 12 h.

2.2 Scope of alkenes / alkynes and amides

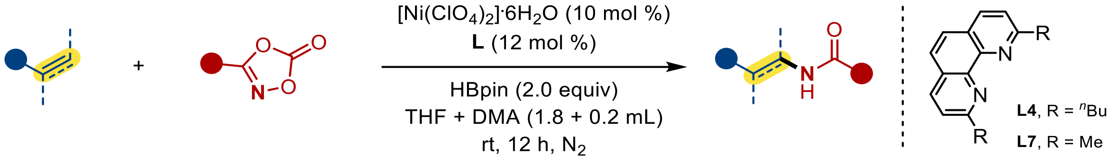

Alkene	Dioxazolone	Linear *N*-alkyl amide[a]	Branched *N*-alkyl amide[a]
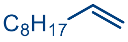 **S38**	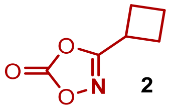 **2**	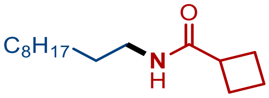 **46**, 65%	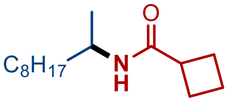 **S85**, 25%
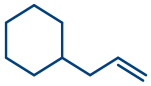 **S39**	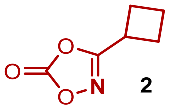 **2**	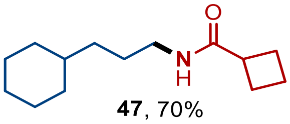 **47**, 70%	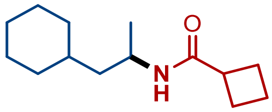 **S86**, 23%[b]
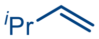 **S40**	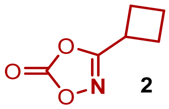 **2**	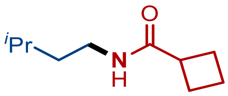 **48**, 88%	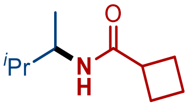 **S87**, n.d.
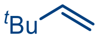 **S41**	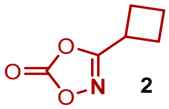 **2**	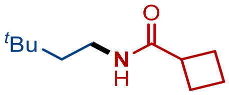 **49**, 33%	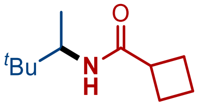 **S88**, n.d.
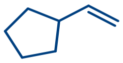 **S42**	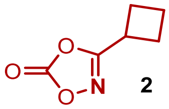 **2**	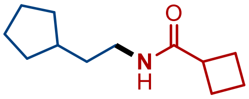 **50**, 80%	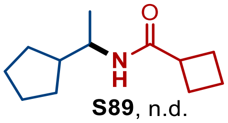 **S89**, n.d.
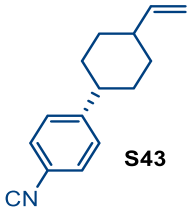 **S43**	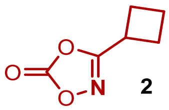 **2**	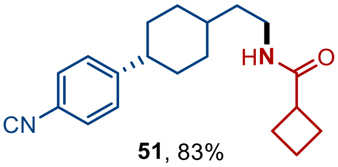 **51**, 83%	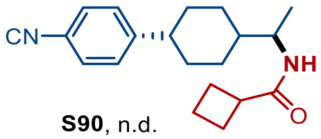 **S90**, n.d.
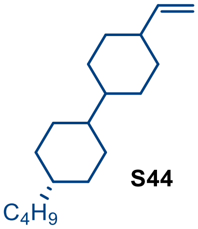 **S44**	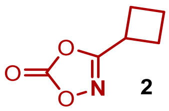 **2**	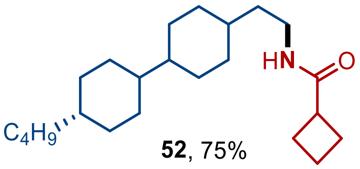 **52**, 75%	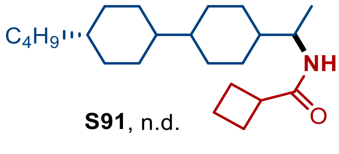 **S91**, n.d.

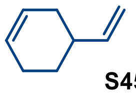 **S45**	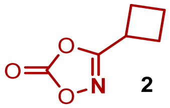 **2**	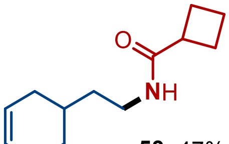 **53**, 47%	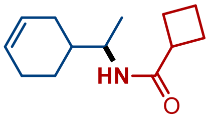 **S92**, n.d.
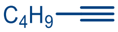 **S46**	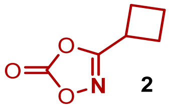 **2**	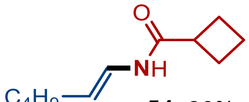 **54**, 26%	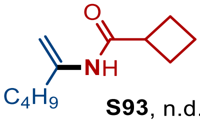 **S93**, n.d.
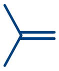 **S47**	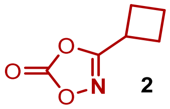 **2**	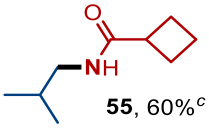 **55**, 60%[c]	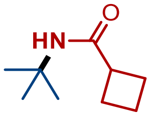 **S94**, n.d.
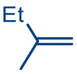 **S48**	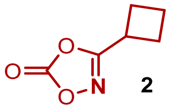 **2**	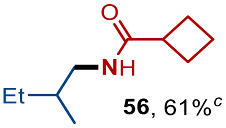 **56**, 61%[c]	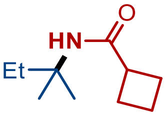 **S95**, n.d.
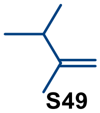 **S49**	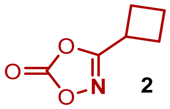 **2**	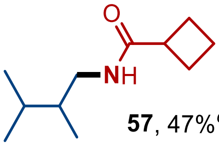 **57**, 47%[c]	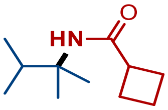 **S96**, n.d.
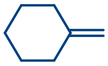 **S50**	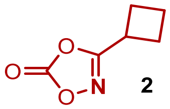 **2**	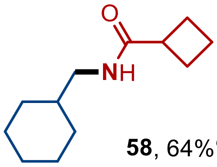 **58**, 64%[c]	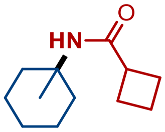 **S97**, n.d.
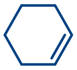 **115**	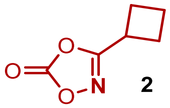 **2**	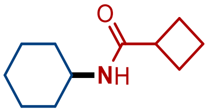 **59**, 49%[c]	-
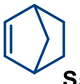 **S51**	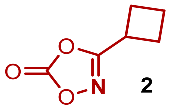 **2**	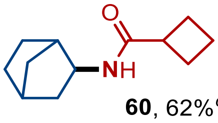 **60**, 62%[c]	-
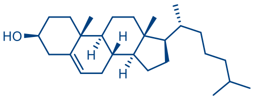 **S52**	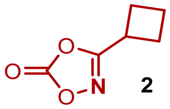 **2**	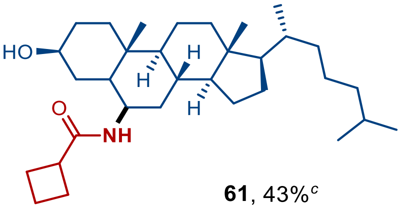 **61**, 43%[c]	-

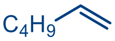 **124**	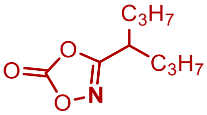 **62**	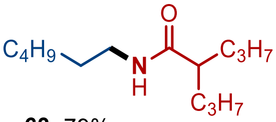 **63**, 79%	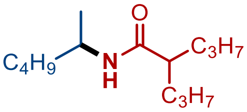 **S98**, 12%[b]
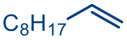 **128**	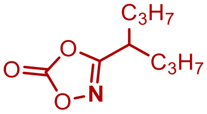 **62**	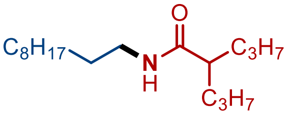 **64**, 76%	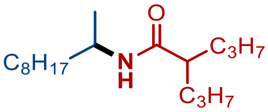 **S99**, 14%[b]
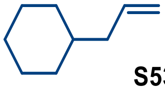 **S53**	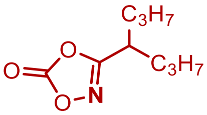 **62**	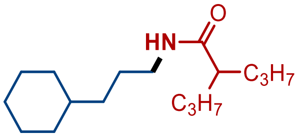 **65**, 86%	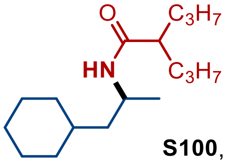 **S100**, 4%[b]
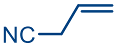 **S54**	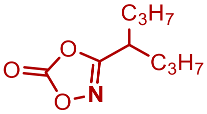 **62**	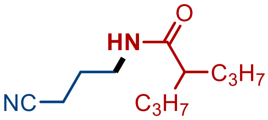 **66**, 18%	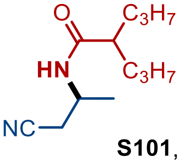 **S101**, 73%
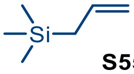 **S55**	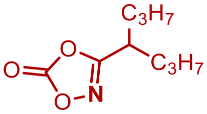 **62**	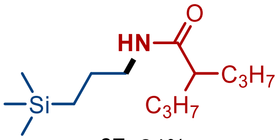 **67**, 24%	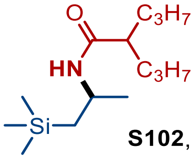 **S102**, 70%
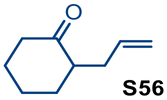 **S56**	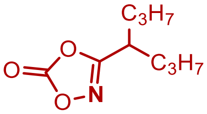 **62**	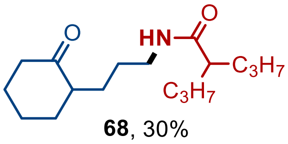 **68**, 30%	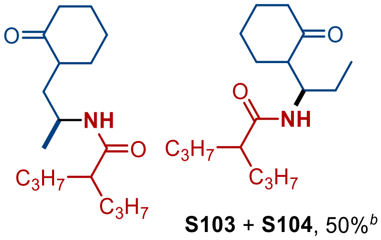 **S103** + **S104**, 50%[b]
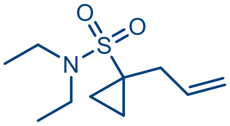 **S57**	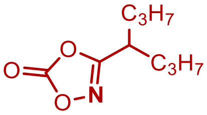 **62**	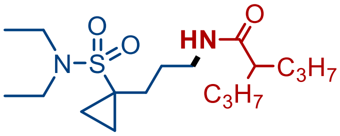 **69**, 74%	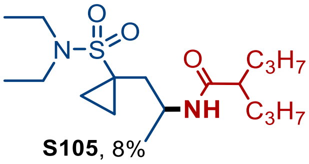 **S105**, 8%
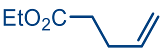 **S58**	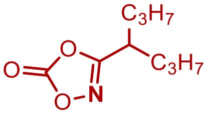 **62**	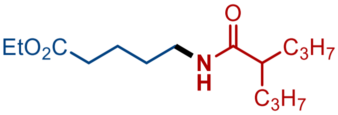 **70**, 67%	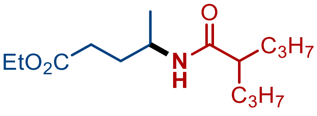 **S106**, 11%

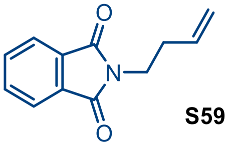 **S59**	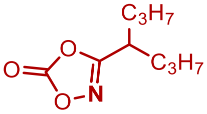 **62**	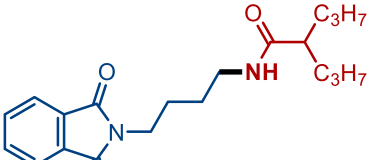 **71**, 61%	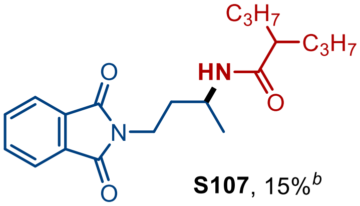 **S107**, 15%[b]
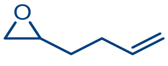 **S60**	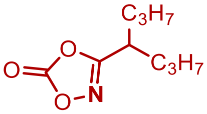 **62**	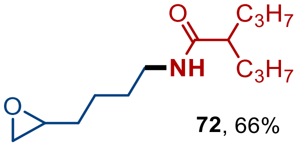 **72**, 66%	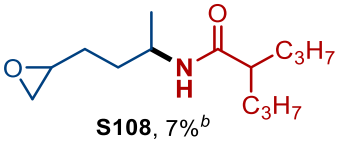 **S108**, 7%[b]
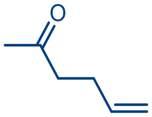 **S61**	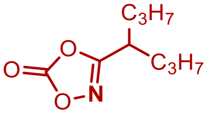 **62**	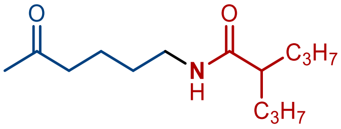 **73**, 36%	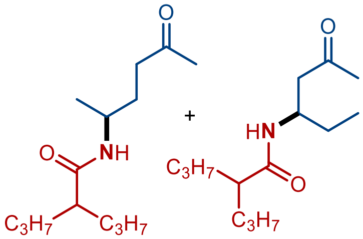 **S109**, 23% **S110**, 30%
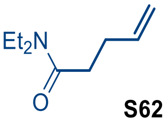 **S62**	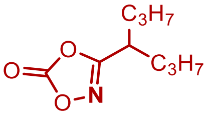 **62**	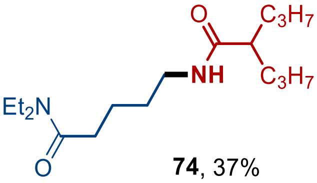 **74**, 37%	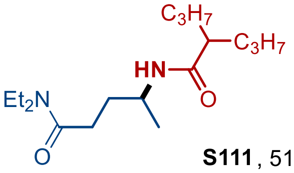 **S111**, 51%
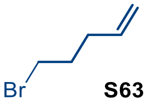 **S63**	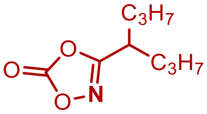 **62**	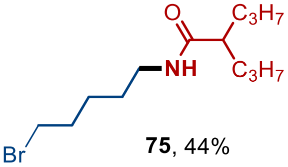 **75**, 44%	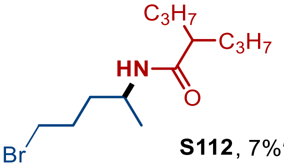 **S112**, 7%[b]
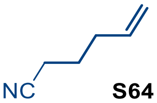 **S64**	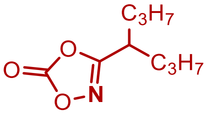 **62**	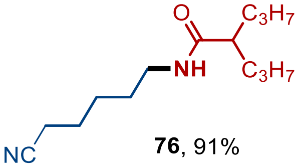 **76**, 91%	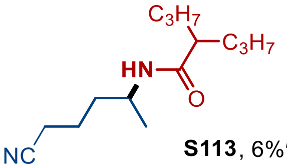 **S113**, 6%[b]
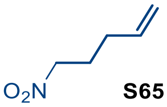 **S65**	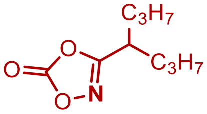 **62**	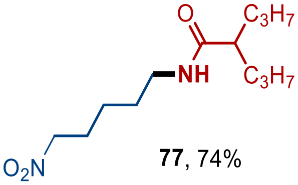 **77**, 74%	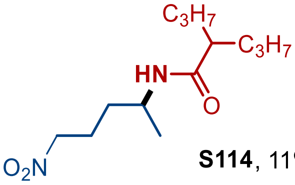 **S114**, 11%

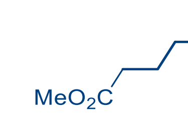 **S66**	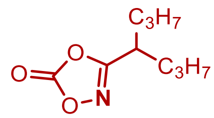 **62**	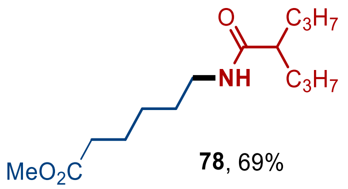 **78**, 69%	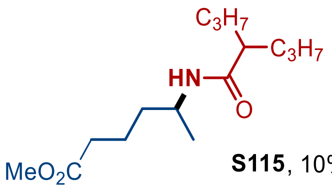 **S115**, 10%
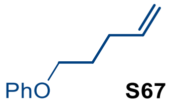 **S67**	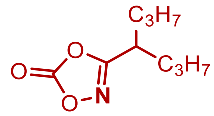 **62**	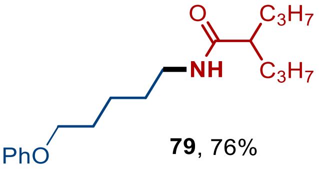 **79**, 76%	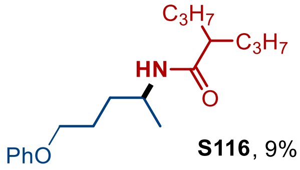 **S116**, 9%
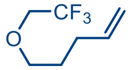 **S68**	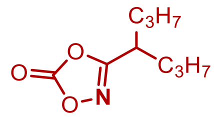 **62**	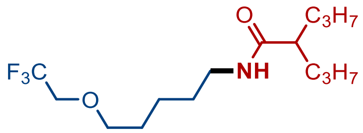 **80**, 70%	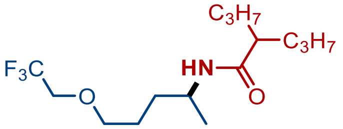 **S117**, 13%
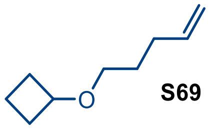 **S69**	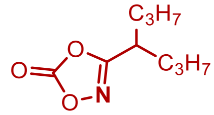 **62**	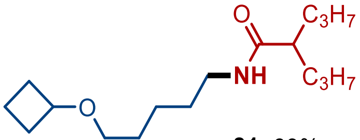 **81**, 69%	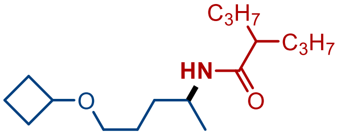 **S118**, 17%
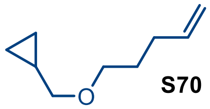 **S70**	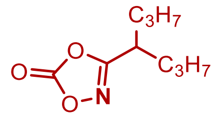 **62**	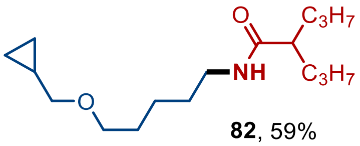 **82**, 59%	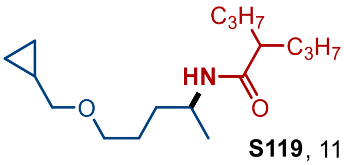 **S119**, 11%
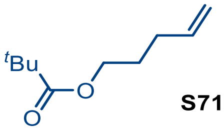 **S71**	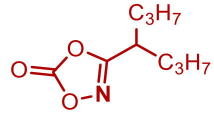 **62**	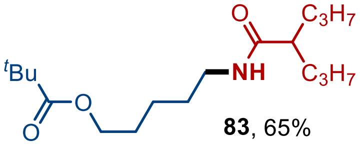 **83**, 65%	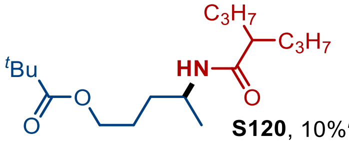 **S120**, 10%[b]
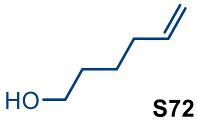 **S72**	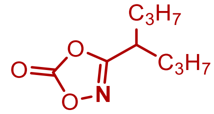 **62**	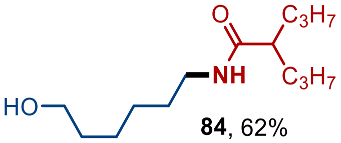 **84**, 62%	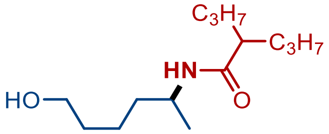 **S121**, 9%[b]
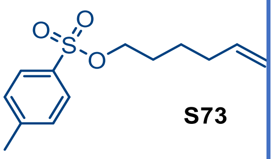 **S73**	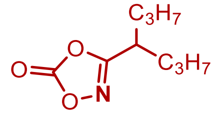 **62**	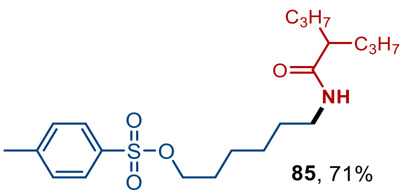 **85**, 71%	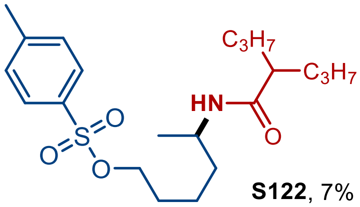 **S122**, 7%

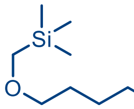 **S74**	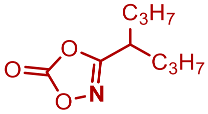 **62**	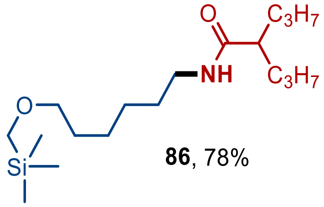 **86**, 78%	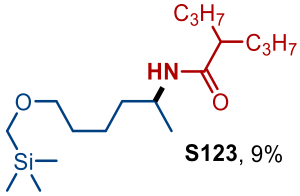 **S123**, 9%
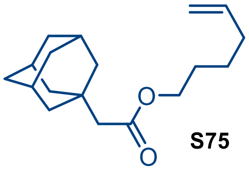 **S75**	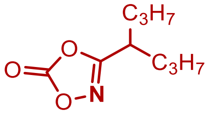 **62**	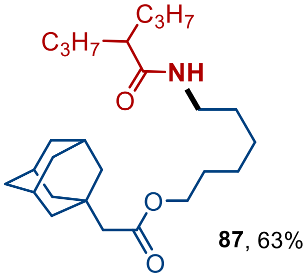 **87**, 63%	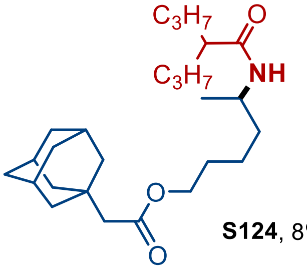 **S124**, 8%
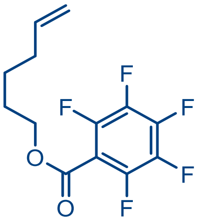 **S76**	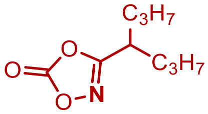 **62**	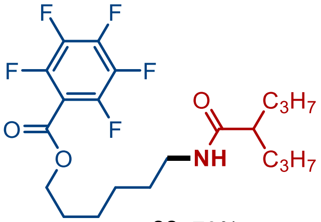 **88**, 79%	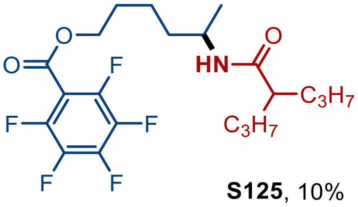 **S125**, 10%
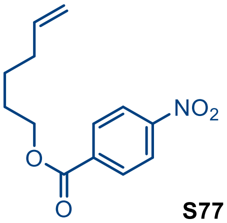 **S77**	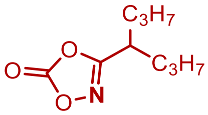 **62**	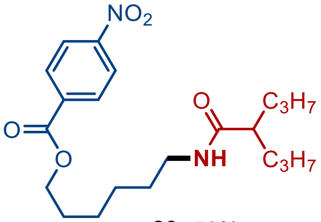 **89**, 56%	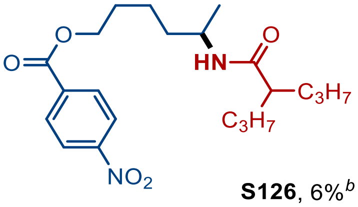 **S126**, 6%[b]
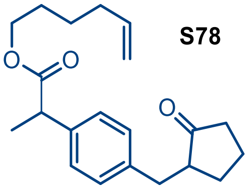 **S78**	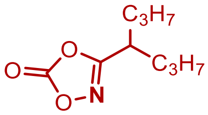 **62**	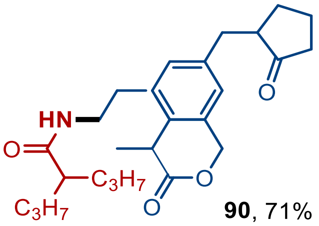 **90**, 71%	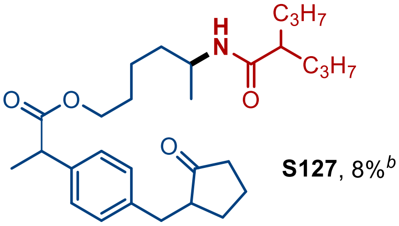 **S127**, 8%[b]
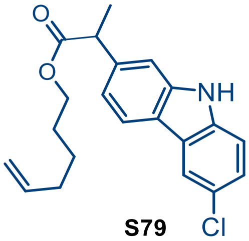 **S79**	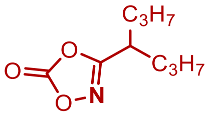 **62**	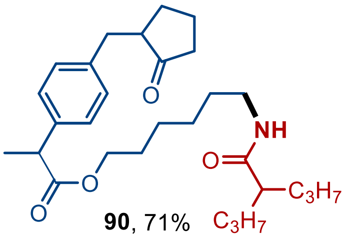 **90**, 71%	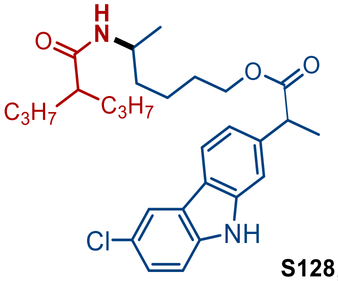 **S128**,7%

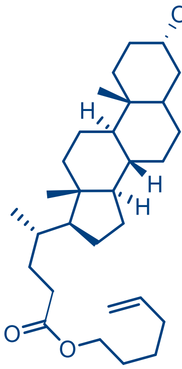 **S80**	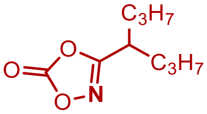 **62**	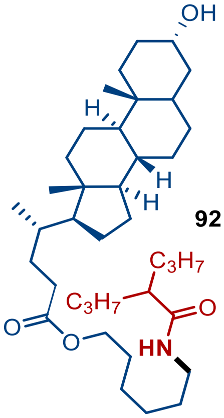 **92**, 67%	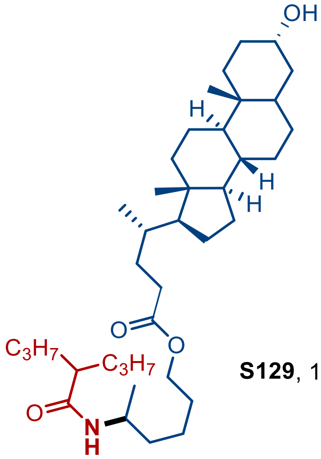 **S129**, 10%[b]
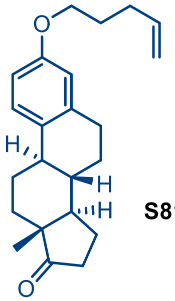 **S81**	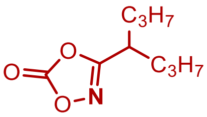 **62**	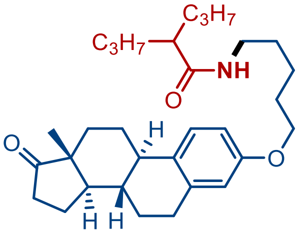 **93**, 62%	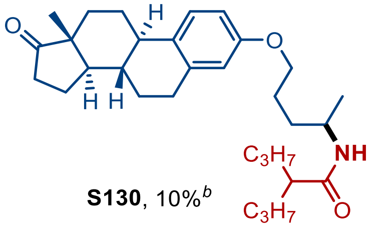 **S130**, 10%[b]
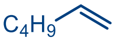 **124**	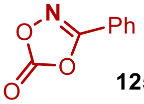 **125**	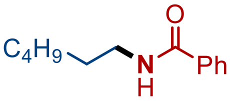 **S84**, 77%	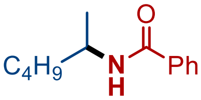 **S132**, 18%

[a]Isolated yields. Reaction conditions: **1** (0.2 mmol), dioxazolones (0.4 mmol), $[Ni(ClO_4)_2]\cdot 6H_2O$ (10 mol %), **L4** (12 mol %), HBpin (2.0 equiv) and THF + DMA (1.8 + 0.2 mL) in N_2 at room temperature for 12 h. [b]NMR yields using CH_2Br_2 as internal standard. [c]**L3** instead of **L4**.

2.3 Exceptional examples

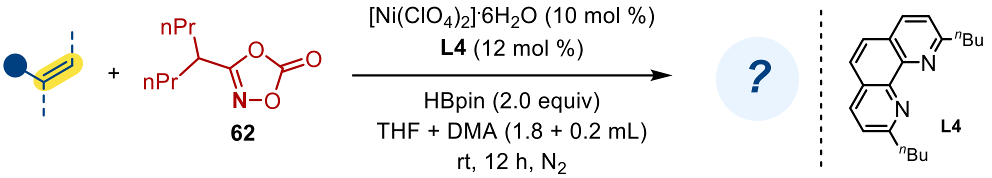

Alkene and Dioxazolone	Products[a]
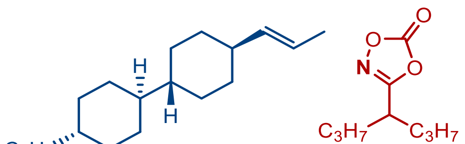 **94** **62**	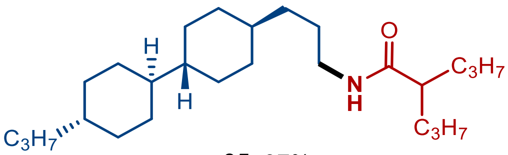 **95**, 37%
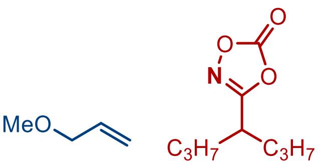 **96** **62**	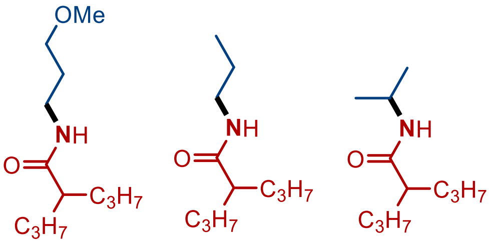 **97**, 43% **98**, 30% **99**, 7%

[a]Isolated yields. Reaction conditions: alkenes (0.2 mmol), dioxazolones (0.2 mmol or 0.4 mmol), $[Ni(ClO_4)_2]\cdot6H_2O$ (10 mol %), **L4** (12 mol %), HBpin (2.0 equiv) and THF + DMA (1.8 + 0.2 mL) in N_2 at room temperature for 12 h.

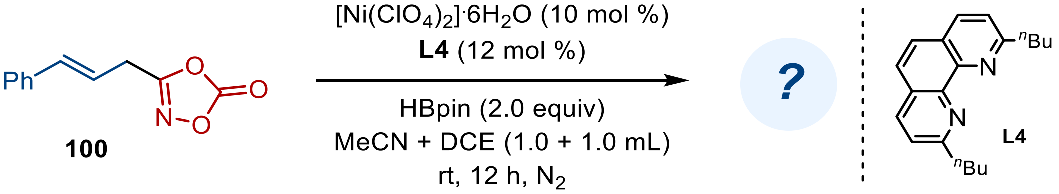

Substrate	Product[a]
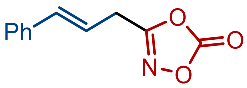 **100**	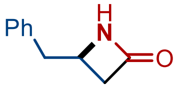 **101**, 61%

[a]Isolated yields. Reaction conditions: **100** (0.2 mmol), $[Ni(ClO_4)_2]\cdot6H_2O$ (10 mol %), **L4** (12 mol %), HBpin (2.0 equiv) and MeCN + DCE (1.0 + 1.0 mL) in N_2 at room temperature for 12 h.

3. Preparation of Dioxazolones[1]

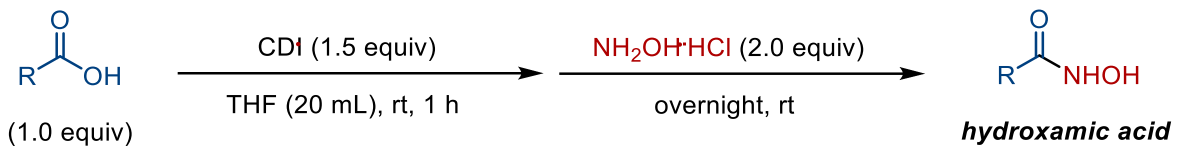

To a 50 mL round bottom flask equipped with a magnetic stir bar, carboxylic acid (10 mmol, 1.0 equiv) was added in a solution of THF (20 mL). Then CDI (1,1'-carbonyldiimidazole) (15 mmol, 1.5 equiv) was added to the solution. The mixture was allowed to stir at room temperature for 1 h. $NH_2OH{\cdot}HCl$ (20 mmol, 2.0 equiv) was then added to the solution. The mixture was stirred overnight. The reaction was quenched with 20 mL 5% $KHSO_4$ aq. and extracted with ethyl acetate (20 mL × 3). The combined organic phase was dried over Na_2SO_4 and concentrated in vacuo. The crude product (hydroxamic acid) was used directly without further purification.

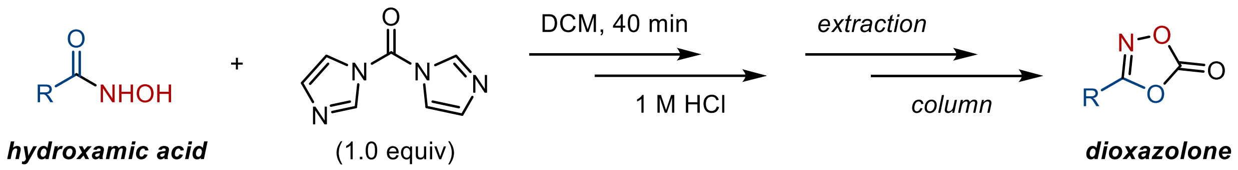

The crude hydroxamic acid was dissolved in DCM (30 mL) and CDI (1.0 equiv) was added to the solution at room temperature. The mixture was then stirred vigorously for 30 min and quenched with 1 M HCl (20 mL). The organic layer in the crude mixture was separated by a separating funnel. The remaining aqueous layer was extracted with DCM (50 mL × 3). The combined organic phase was dried over Na_2SO_4, concentrated in vacuo. The corresponding dioxazolones were purified by flash chromatography (n-hexane : ethyl acetate = 100 : 1 to 4 : 1) with the yields were calculated based on the amount of carboxylic acid.

Physical characterization of dioxazolones

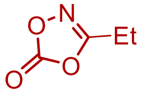

3-Ethyl-1,4,2-dioxazol-5-one (113): colorless oil. **^{1}H NMR** (400 MHz, $CDCl_3$) δ 2.64 (q, *J* = 7.6 Hz, 2H), 1.29 (t, *J* = 7.6 Hz, 3H) ppm. **^{13}C NMR** (101 MHz, $CDCl_3$) δ 167.48, 154.22, 18.60, 8.67 ppm.

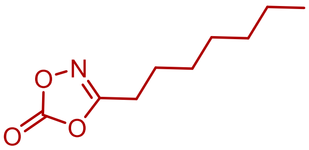

3-Heptyl-1,4,2-dioxazol-5-one (S1): colorless oil. **^{1}H NMR** (400 MHz, $CDCl_3$) δ 2.61 (t, *J* = 7.5 Hz, 2H), 1.71 (p, *J* = 7.5 Hz, 2H), 1.44 – 1.31 (m, 4H), 1.27 (d, *J* = 6.8 Hz, 12H), 0.88 (t, *J* = 6.8 Hz, 3H) ppm. **^{13}C NMR** (101 MHz, $CDCl_3$) δ 166.74, 31.88, 29.54, 29.50, 29.28, 28.92, 28.70, 24.74, 24.51, 22.66, 14.08 ppm.

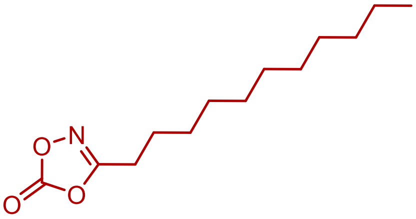

3-Undecyl-1,4,2-dioxazol-5-one (S2): colorless oil. **^{1}H NMR** (400 MHz, $CDCl_3$) δ 2.61 (t, *J* = 7.5 Hz, 2H), 1.71 (p, *J* = 7.4 Hz, 2H), 1.43 – 1.22 (m, 8H), 0.88 (t, *J* = 6.7 Hz, 3H) ppm. **^{13}C NMR** (101 MHz, $CDCl_3$) δ 166.76, 154.23, 31.45, 28.64, 28.57, 24.71, 24.49, 22.50, 13.96 ppm.

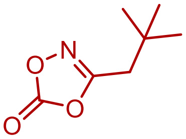

3-Neopentyl-1,4,2-dioxazol-5-one (S3): colorless oil. **^{1}H NMR** (400 MHz, $CDCl_3$) δ 2.51 (s, 2H), 1.07 (s, 9H) ppm. **^{13}C NMR** (101 MHz, $CDCl_3$) δ 165.73, 154.36, 38.50, 31.42, 29.49 ppm.

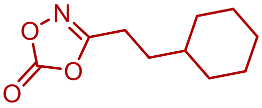

3-(2-Cyclohexylethyl)-1,4,2-dioxazol-5-one (S4): colorless oil. **^{1}H NMR** (400 MHz, $CDCl_3$) δ 2.68 – 2.58 (m, 2H), 1.75 – 1.56 (m, 7H), 1.36 – 1.13 (m, 4H), 1.00 – 0.86 (m, 2H) ppm. **^{13}C NMR** (101 MHz, $CDCl_3$) δ 167.03, 154.23, 36.78, 32.68, 31.73, 26.30, 25.99, 22.36 ppm.

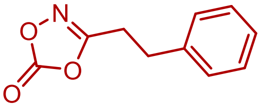

3-Phenethyl-1,4,2-dioxazol-5-one (S5): colorless oil. **^{1}H NMR** (400 MHz, $CDCl_3$) δ 7.40 – 7.12 (m, 5H), 3.07 – 2.99 (m, 2H), 2.96 – 2.88 (m, 2H) ppm. **^{13}C NMR** (101 MHz, $CDCl_3$) δ 165.87, 154.05, 138.04, 128.95, 128.21, 127.19, 30.46, 26.68 ppm.

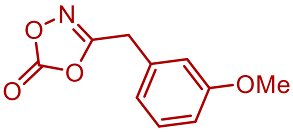

3-(3-Methoxybenzyl)-1,4,2-dioxazol-5-one (S6): colorless oil. **^{1}H NMR** (400 MHz, $CDCl_3$) δ 7.30 (dd, *J* = 8.3, 7.5 Hz, 1H), 6.91 – 6.85 (m, 2H), 6.83 (t, *J* = 2.1 Hz, 1H), 3.89 (s, 2H), 3.81 (s, 3H) ppm. **^{13}C NMR** (101 MHz, $CDCl_3$) δ 171.02, 164.52, 154.20, 56.03, 51.78, 47.69, 47.63, 46.82 ppm.

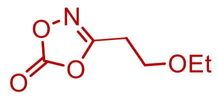

3-(2-Ethoxyethyl)-1,4,2-dioxazol-5-one (S7): colorless oil. **^{1}H NMR** (400 MHz, $CDCl_3$) δ 3.73 (t, *J* = 6.2 Hz, 2H), 3.51 (q, *J* = 7.0 Hz, 2H), 2.88 (t, *J* = 6.1 Hz, 2H), 1.18 (t, *J* = 7.0 Hz, 3H) ppm. **^{13}C NMR** (101 MHz, $CDCl_3$) δ 164.94, 154.10, 66.66, 64.25, 26.07, 14.87 ppm.

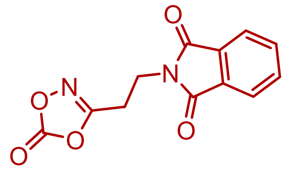

2-(2-(5-Oxo-1,4,2-dioxazol-3-yl)ethyl)isoindoline-1,3-dione (S8): white solid. **^{1}H NMR** (400 MHz, $CDCl_3$) δ 7.92 – 7.82 (m, 2H), 7.80 – 7.68 (m, 2H), 4.08 (t, *J* = 6.4 Hz, 2H), 3.02 (t, *J* = 6.4 Hz, 2H) ppm. **^{13}C NMR** (101 MHz, $CDCl_3$) δ 167.71, 164.04, 153.65, 134.45, 131.64, 123.69, 33.20, 24.71 ppm.

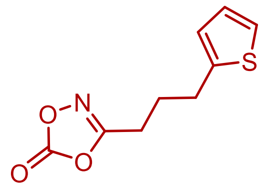

3-(3-(Thiophen-2-yl)propyl)-1,4,2-dioxazol-5-one (S9): pale yellow oil. **^{1}H NMR** (400 MHz, $CDCl_3$) δ 7.17 (dd, *J* = 5.2, 1.2 Hz, 1H), 6.94 (dd, *J* = 5.1, 3.4 Hz, 1H), 6.83 (dd, *J* = 3.4, 1.2 Hz, 1H), 2.98 (t, *J* = 7.2 Hz, 2H), 2.67 (t, *J* = 7.5 Hz, 2H), 2.11 (p, *J* = 7.3 Hz, 2H) ppm. **^{13}C NMR** (101 MHz, $CDCl_3$) δ 166.28, 154.06, 142.29, 127.03, 125.21, 123.91, 28.64, 26.25, 23.84 ppm.

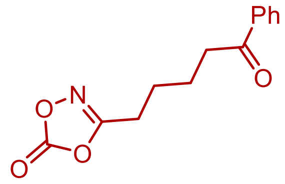

3-(5-Oxo-5-phenylpentyl)-1,4,2-dioxazol-5-one (S10): colorless oil. **^{1}H NMR** (400 MHz, $CDCl_3$) δ 7.97 – 7.92 (m, 2H), 7.60 – 7.55 (m, 1H), 7.50 – 7.44 (m, 2H), 3.04 (t, *J* = 6.7 Hz, 2H), 2.69 (t, *J* = 7.1 Hz, 2H), 1.91 – 1.78 (m, 4H) ppm. **^{13}C NMR** (101 MHz, $CDCl_3$) δ 199.07, 166.36, 154.11, 136.73, 133.25, 128.69, 127.97, 37.53, 24.80, 24.09, 23.05 ppm.

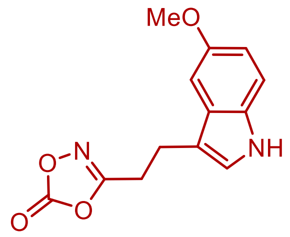

3-(2-(5-Methoxy-1H-indol-3-yl)ethyl)-1,4,2-dioxazol-5-one (S11): white solid. **^{1}H NMR** (400 MHz, $CDCl_3$) δ 7.97 (s, 1H), 7.26 (d, *J* = 8.9 Hz, 1H), 7.00 (dd, *J* = 20.5, 2.4 Hz, 2H), 6.89 (dd, *J* = 8.8, 2.4 Hz, 1H), 3.87 (s, 3H), 3.17 (tt, *J* = 7.0, 1.0 Hz, 2H), 3.03 – 2.97 (m, 2H) ppm. **^{13}C NMR** (101 MHz, $CDCl_3$) δ 166.29, 154.29, 154.18, 131.41, 127.07, 122.64, 112.68, 112.31, 112.23, 100.03, 55.96, 25.82, 20.55 ppm.

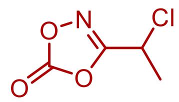

3-(1-Chloroethyl)-1,4,2-dioxazol-5-one (S12): colorless oil. **^{1}H NMR** (400 MHz, $CDCl_3$) δ 4.86 (q, *J* = 7.0 Hz, 1H), 1.88 (d, *J* = 7.0 Hz, 3H) ppm. **^{13}C NMR** (101 MHz, $CDCl_3$) δ 165.16, 153.04, 44.13, 20.38 ppm.

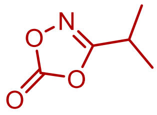

3-Isopropyl-1,4,2-dioxazol-5-one (S13): colorless oil. ^{1}H NMR (400 MHz, $CDCl_3$) δ 2.95 (dq, *J* = 13.9, 7.0 Hz, 1H), 1.35 – 1.30 (m, 6H) ppm. ^{13}**C NMR** (101 MHz, $CDCl_3$) δ 170.24, 154.35, 26.15, 18.03 ppm.

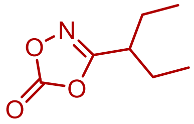

3-(Pentan-3-yl)-1,4,2-dioxazol-5-one (S14): colorless oil. ^{1}**H NMR** (400 MHz, $CDCl_3$) δ 2.56 (p, *J* = 7.2 Hz, 1H), 1.72 (q, *J* = 7.4 Hz, 4H), 0.96 (t, *J* = 7.5 Hz, 6H) ppm. ^{13}**C NMR** (101 MHz, $CDCl_3$) δ 168.68, 154.35, 40.08, 23.95, 11.31 ppm.

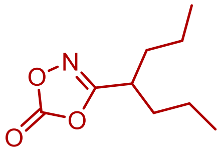

3-(Heptan-4-yl)-1,4,2-dioxazol-5-one (62): colorless oil. ^{1}**H NMR** (400 MHz, $CDCl_3$) δ 2.69 (tt, *J* = 8.6, 5.9 Hz, 1H), 1.66 – 1.54 (m, 4H), 1.36 – 1.27 (m, 4H), 0.90 (t, *J* = 7.3 Hz, 6H) ppm. ^{13}**C NMR** (101 MHz, $CDCl_3$) δ 168.95, 36.44, 33.27, 20.13, 13.61 ppm.

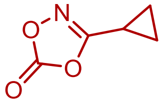

3-Cyclopropyl-1,4,2-dioxazol-5-one (S15): colorless oil. ^{1}**H NMR** (400 MHz, $CDCl_3$) δ 1.91 (tt, *J* = 8.3, 5.1 Hz, 1H), 1.23 – 1.11 (m, 4H) ppm. ^{13}**C NMR** (101 MHz, $CDCl_3$) δ 167.84, 153.95, 7.32, 5.42 ppm.

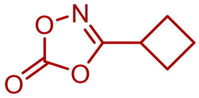

3-Cyclobutyl-1,4,2-dioxazol-5-one (2): colorless oil. ^{1}**H NMR** (400 MHz, $CDCl_3$) δ 3.46 (qd, *J* = 8.4, 1.1 Hz, 1H), 2.38 (td, *J* = 8.8, 7.1 Hz, 4H), 2.21 – 2.00 (m, 2H) ppm. ^{13}**C NMR** (101 MHz, $CDCl_3$) δ 168.44, 154.49, 29.51, 25.18, 18.99 ppm.

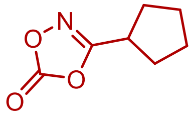

3-Cyclopentyl-1,4,2-dioxazol-5-one (S16): colorless oil. ^{1}**H NMR** (400 MHz, $CDCl_3$) δ 3.06 (tt, *J* = 8.6, 7.1 Hz, 1H), 2.04 (dddd, *J* = 12.1, 9.5, 5.0, 3.2 Hz, 2H), 1.90 – 1.62 (m, 6H) ppm. ^{13}**C NMR** (101 MHz, $CDCl_3$) δ 169.52, 154.44, 35.14, 29.33, 25.43 ppm.

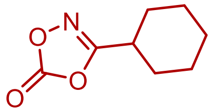

3-Cyclohexyl-1,4,2-dioxazol-5-one (S17): colorless oil. ^{1}**H NMR** (400 MHz, $CDCl_3$) δ 2.67 (tt, *J* = 11.2, 3.6 Hz, 1H), 2.04 – 1.95 (m, 2H), 1.83 (dp, *J* = 11.0, 3.7 Hz, 2H), 1.76 – 1.67 (m, 1H), 1.59 – 1.46 (m, 2H), 1.42 – 1.25 (m, 3H). ^{13}**C NMR** (101 MHz, $CDCl_3$) δ 169.31, 154.34, 34.67, 28.17, 25.25, 24.85 ppm.

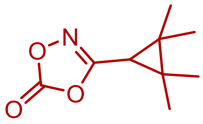

3-(2,2,3,3-Tetramethylcyclopropyl)-1,4,2-dioxazol-5-one (S18): colorless oil. ^{1}**H NMR** (400 MHz, $CDCl_3$) δ 1.29 (s, 1H), 1.27 (d, *J* = 2.5 Hz, 6H), 1.23 (d, *J* = 2.7 Hz, 6H) ppm. ^{13}**C NMR** (101 MHz, $CDCl_3$) δ 166.18, 154.25, 77.35, 77.04, 29.92, 28.00, 22.92, 17.74 ppm.

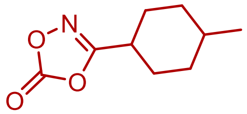

3-(4-Methylcyclohexyl)-1,4,2-dioxazol-5-one (S19): white solid. **^{1}H NMR** (400 MHz, $CDCl_3$) δ 2.56 (tt, *J* = 12.3, 3.5 Hz, 1H), 2.08 – 1.98 (m, 2H), 1.89 – 1.78 (m, 2H), 1.56 – 1.35 (m, 3H), 1.01 (qd, *J* = 13.4, 3.5 Hz, 2H), 0.91 (d, *J* = 6.6 Hz, 3H) ppm. **^{13}C NMR** (101 MHz, $CDCl_3$) δ 169.44, 154.33, 34.62, 33.63, 31.67, 28.23, 22.22 ppm.

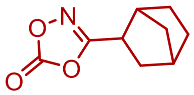

3-(Bicyclo[2.2.1]heptan-2-yl)-1,4,2-dioxazol-5-one (S20): colorless oil. **^{1}H NMR** (400 MHz, $CDCl_3$) δ 3.01 (t, *J* = 5.8 Hz, 1H), 2.64 (dddd, *J* = 12.7, 6.2, 4.8, 1.6 Hz, 2H), 2.55 (d, *J* = 4.0 Hz, 1H), 2.40 (dt, *J* = 6.5, 4.4 Hz, 2H), 1.85 (ddt, *J* = 12.9, 6.2, 3.7 Hz, 2H), 1.50 – 1.46 (m, 3H), 1.30 (ddt, *J* = 10.3, 8.9, 2.7 Hz, 5H) ppm. **^{13}C NMR** (101 MHz, $CDCl_3$) δ 169.10, 168.68, 154.54, 154.48, 40.44, 39.95, 39.55, 38.20, 37.28, 36.56, 36.37, 36.01, 33.10, 30.81, 29.31, 29.11, 28.36, 24.28 ppm.

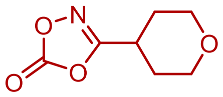

3-(Tetrahydro-2*H*-pyran-4-yl)-1,4,2-dioxazol-5-one (S21): white solid. **^{1}H NMR** (400 MHz, $CDCl_3$) δ 4.04 (dt, *J* = 11.9, 3.6 Hz, 1H), 3.51 (ddd, *J* = 11.9, 10.7, 2.9 Hz, 1H), 2.96 (tt, *J* = 10.8, 4.4 Hz, 0H), 2.01 – 1.79 (m, 2H) ppm. **^{13}C NMR** (101 MHz, $CDCl_3$) δ 167.94, 153.97, 66.38, 32.10, 27.66 ppm.

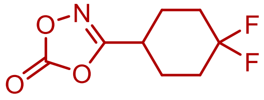

3-(4,4-Difluorocyclohexyl)-1,4,2-dioxazol-5-one (S22): white solid. **^{1}H NMR** (400 MHz, $CDCl_3$) δ 2.80 (td, *J* = 9.1, 8.0, 4.6 Hz, 1H), 2.25 – 2.09 (m, 4H), 1.98 – 1.80 (m, 4H) ppm. **^{13}C NMR** (101 MHz, $CDCl_3$) δ 167.66 (d, *J* = 1.9 Hz), 153.83, 126.36 – 117.78 (m), 32.42, 32.07 (t, *J* = 25.1 Hz), 24.86 – 24.19 (m) ppm. **^{19}F NMR** (376 MHz, $CDCl_3$) δ -94.56 (d, *J* = 240.3 Hz), -100.49 (d, *J* = 240.8 Hz) ppm.

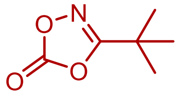

3-(Tert-butyl)-1,4,2-dioxazol-5-one (114): colorless oil. **^{1}H NMR** (400 MHz, $CDCl_3$) δ 1.35 (s, 9H) ppm. **^{13}C NMR** (101 MHz, $CDCl_3$) δ 172.37, 154.48, 32.57, 26.27 ppm.

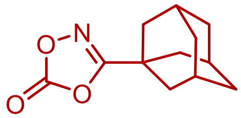

3-(Adamantan-1-yl)-1,4,2-dioxazol-5-one (S23): white solid. **^{1}H NMR** (400 MHz, $CDCl_3$) δ 2.15 – 2.06 (m, 1H), 1.95 (d, *J* = 2.9 Hz, 2H), 1.88 – 1.69 (m, 2H) ppm. **^{13}C NMR** (101 MHz, $CDCl_3$) δ 171.72, 154.65, 38.08, 36.09, 34.60, 27.27 ppm.

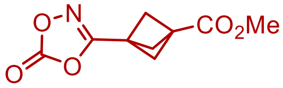

Methyl-3-(5-oxo-1,4,2-dioxazol-3-yl)bicyclo[1.1.1]pentane-1-carboxylate (S24): white solid. **^{1}H NMR** (400 MHz, $CDCl_3$) δ 3.71 (s, 3H),

2.50 (s, 6H) ppm. **^{13}C NMR** (101 MHz, $CDCl_3$) δ 168.23, 162.89, 153.73, 53.22, 52.13, 39.87, 30.83 ppm.

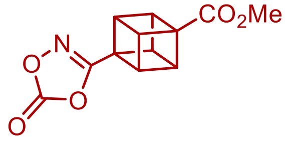

Methyl-4-(5-oxo-1,4,2-dioxazol-3-yl)cubane-1-carboxylate (S25): white solid. **^{1}H NMR** (400 MHz, $CDCl_3$) δ 4.43 – 4.32 (m, 6H), 3.71 (s, 3H) ppm. **^{13}C NMR** (101 MHz, $CDCl_3$) δ 171.02, 164.52, 154.20, 56.03, 51.78, 47.69, 47.63, 46.82 ppm.

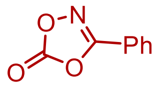

3-Phenyl-1,4,2-dioxazol-5-one (125): white solid. **^{1}H NMR** (400 MHz, $CDCl_3$) δ 7.92 – 7.80 (m, 2H), 7.69 – 7.60 (m, 1H), 7.60 – 7.47 (m, 2H) ppm. **^{13}C NMR** (101 MHz, $CDCl_3$) δ 163.56, 153.86, 133.80, 129.41, 126.63, 120.15 ppm.

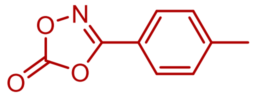

3-(*p*-Tolyl)-1,4,2-dioxazol-5-one (S26): white solid. **^{1}H NMR** (400 MHz, $CDCl_3$) δ 7.76 – 7.69 (m, 2H), 7.34 (d, J = 8.1 Hz, 2H), 2.45 (s, 3H) ppm. **^{13}C NMR** (101 MHz, $CDCl_3$) δ 163.66, 153.97, 144.84, 130.09, 126.57, 117.25, 21.81 ppm.

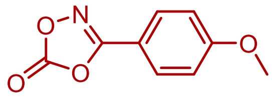

3-(4-Methoxyphenyl)-1,4,2-dioxazol-5-one (S27): white solid. **^{1}H NMR** (400 MHz, $CDCl_3$) δ 7.82 – 7.76 (m, 2H), 7.07 – 6.99 (m, 2H), 3.89 (s, 3H) ppm. **^{13}C NMR** (101 MHz, $CDCl_3$) δ 163.88, 163.43, 154.06, 128.58, 114.89, 112.14, 55.62 ppm.

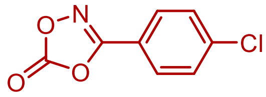

3-(4-Chlorophenyl)-1,4,2-dioxazol-5-one (S28): white solid. **^{1}H NMR** (400 MHz, $CDCl_3$) δ 7.87 – 7.76 (m, 2H), 7.59 – 7.49 (m, 2H) ppm. **^{13}C NMR** (101 MHz, $CDCl_3$) δ 162.84, 153.53, 140.40, 129.91, 127.90, 118.58 ppm.

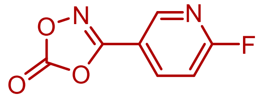

3-(6-Fluoropyridin-3-yl)-1,4,2-dioxazol-5-one (S29): pale brown solid. **^{1}H NMR** (400 MHz, $CDCl_3$) δ 8.75 (d, J = 2.5 Hz, 1H), 8.28 (ddd, J = 9.1, 7.1, 2.5 Hz, 1H), 7.17 (dd, J = 8.6, 3.0 Hz, 1H) ppm. **^{13}C NMR** (101 MHz, $CDCl_3$) δ 166.08 (d, J = 248.9 Hz), 161.15, 153.00, 147.16 (d, J = 16.9 Hz), 139.27 (d, J = 9.3 Hz), 115.25 (d, J = 5.0 Hz), 111.09 (d, J = 37.9 Hz) ppm. ^{19}F NMR (376 MHz, $CDCl_3$) δ -58.00 ppm.

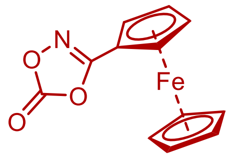

(2-(5-Oxo-1,4,2-dioxazol-3-yl)cyclopenta-2,4-dien-1-yl)(cyclopenta-2,4-dien-1-yl)iron (S30): pale brown solid. **^{1}H NMR** (400 MHz, $CDCl_3$) δ 4.80 (t, J = 1.9 Hz, 2H), 4.57 (t, J = 2.0 Hz, 2H), 4.29 (s, 5H) ppm. **^{13}C NMR** (101 MHz, $CDCl_3$) δ 71.96, 70.39, 67.83, 61.89 ppm.

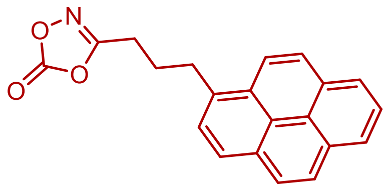

3-(3-(Pyren-1-yl)propyl)-1,4,2-dioxazol-5-one (S31): white solid. **^{1}H NMR** (400 MHz, $CDCl_3$) δ 8.22 – 8.15 (m, 3H), 8.15 – 8.09 (m, 2H), 8.03 (d, J = 11.4 Hz, 3H), 7.82 (d, J = 7.8 Hz, 1H), 3.43 (t, J = 7.5 Hz, 2H), 2.66 (t, J = 7.4 Hz, 2H), 2.26 (p, J = 7.5 Hz, 2H) ppm. **^{13}C NMR** (101 MHz, $CDCl_3$) δ 166.41, 133.83, 131.41, 130.81, 130.35, 128.69, 127.83, 127.44, 127.24, 127.08, 126.06, 125.25, 125.18, 125.04, 124.93, 124.89, 122.70, 32.10, 25.97, 24.35 ppm.

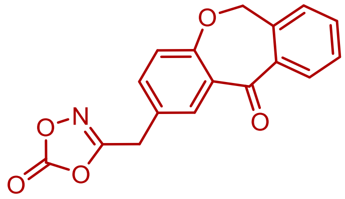

3-((11-Oxo-6,11-dihydrodibenzo[b,e]oxepin-2-yl)methyl)-1,4,2-dioxazol-5-one (S32): white solid. **^{1}H NMR** (400 MHz, $CDCl_3$) δ 8.17 (d, J = 2.5 Hz, 1H), 7.89 (dd, J = 7.7, 1.4 Hz, 1H), 7.58 (td, J = 7.5, 1.4 Hz, 1H), 7.49 (td, J = 7.6, 1.3 Hz, 1H), 7.39 (ddd, J = 9.3, 8.0, 1.9 Hz, 2H), 7.08 (d, J = 8.5 Hz, 1H), 5.20 (s, 2H), 3.95 (s, 2H) ppm. **^{13}C NMR** (101 MHz, $CDCl_3$) δ 190.40, 165.10, 161.27, 153.83, 140.20, 135.48, 135.33, 133.07, 132.63, 129.56, 129.45, 127.96, 125.59, 123.98, 122.02, 73.69, 30.40 ppm.

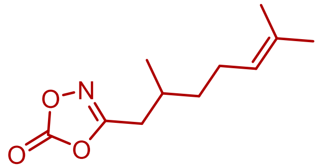

3-(2,6-Dimethylhept-5-en-1-yl)-1,4,2-dioxazol-5-one (S33): colorless oil. **^{1}H NMR** (400 MHz, $CDCl_3$) δ 5.06 (dddd, J = 7.1, 5.6, 2.9, 1.5 Hz, 1H), 2.61 (dd, J = 15.3, 5.9 Hz, 1H), 2.44 (dd, J = 15.2, 8.0 Hz, 1H), 2.09 – 1.90 (m, 3H), 1.68 (d, J = 1.4 Hz, 3H), 1.60 (d, J = 1.3 Hz, 3H), 1.45 – 1.26 (m, 2H), 1.02 (d, J = 6.7 Hz, 3H) ppm. **^{13}C NMR** (101 MHz, $CDCl_3$) δ 166.10, 154.22, 132.34, 123.37, 36.29, 31.73, 29.77, 25.67, 25.15, 19.25, 17.67 ppm.

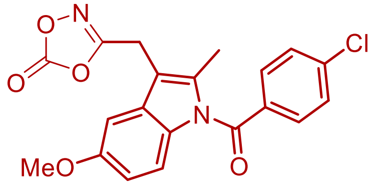

3-((1-(4-Chlorobenzoyl)-5-methoxy-2-methyl-1H-indol-3-yl)methyl)-1,4,2-dioxazol-5-one (S34): white solid. **^{1}H NMR** (400 MHz, $CDCl_3$) δ 7.66 (d, J = 8.5 Hz, 2H), 7.47 (d, J = 8.5 Hz, 2H), 6.93 (d, J = 2.4 Hz, 1H), 6.83 (d, J = 9.0 Hz, 1H), 6.70 (dd, J = 9.1, 2.5 Hz, 1H), 3.99 (s, 2H), 3.83 (s, 3H), 2.44 (s, 3H) ppm. **^{13}C NMR** (101 MHz, $CDCl_3$) δ 168.16, 164.56, 156.27, 153.89, 139.74, 137.08, 133.34, 131.29, 130.75, 129.43, 129.28, 115.17, 112.24, 108.33, 100.64, 55.76, 20.43, 13.11 ppm.

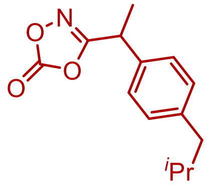

3-(1-(4-Isobutylphenyl)ethyl)-1,4,2-dioxazol-5-one (S35): white solid. **^{1}H NMR** (400 MHz, $CDCl_3$) δ 7.18 (q, J = 8.3 Hz, 4H), 4.04 (q, J = 7.2 Hz, 1H), 2.48 (d, J = 7.2 Hz, 2H), 1.92 – 1.80 (m, 1H), 1.67 (d, J = 7.2 Hz, 3H), 0.91 (d, J =

6.6 Hz, 6H) ppm. **13C NMR** (101 MHz, $CDCl_3$) δ 168.54, 142.11, 134.04, 129.91, 127.05, 44.97, 36.70, 30.13, 22.32, 17.19 ppm.

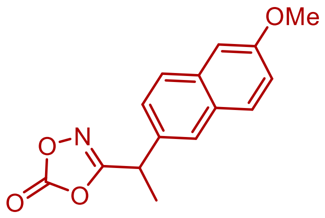

3-(1-(6-Methoxynaphthalen-2-yl)ethyl)-1,4,2-dioxazol-5-one (S36): white solid. **1H NMR** (400 MHz, $CDCl_3$) δ 7.74 (dd, J = 14.0, 8.7 Hz, 2H), 7.68 (d, J = 1.9 Hz, 1H), 7.35 (dd, J = 8.5, 1.9 Hz, 1H), 7.19 (dd, J = 8.9, 2.6 Hz, 1H), 7.14 (d, J = 2.5 Hz, 1H), 4.20 (q, J = 7.3 Hz, 1H), 3.93 (s, 3H), 1.75 (d, J = 7.2 Hz, 3H) ppm. **13C NMR** (101 MHz, $CDCl_3$) δ 167.94, 153.97, 66.38, 32.10, 27.66 ppm.

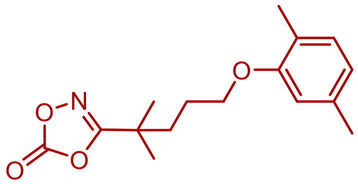

3-(5-(2,5-Dimethylphenoxy)-2-methylpentan-2-yl)-1,4,2-dioxazol-5-one (S37): colorless oil. **1H NMR** (400 MHz, $CDCl_3$) δ 7.03 (d, J = 7.4 Hz, 1H), 6.75 – 6.65 (m, 1H), 6.61 (d, J = 1.6 Hz, 1H), 3.96 (t, J = 5.6 Hz, 2H), 2.33 (s, 3H), 2.19 (s, 3H), 1.90 – 1.77 (m, 4H), 1.39 (s, 6H) ppm. **13C NMR** (101 MHz, $CDCl_3$) δ 171.60, 156.70, 136.55, 130.44, 123.56, 121.02, 111.95, 67.14, 36.08, 35.92, 24.62, 24.18, 21.39, 15.73 ppm.

4. Preparation of alkenes

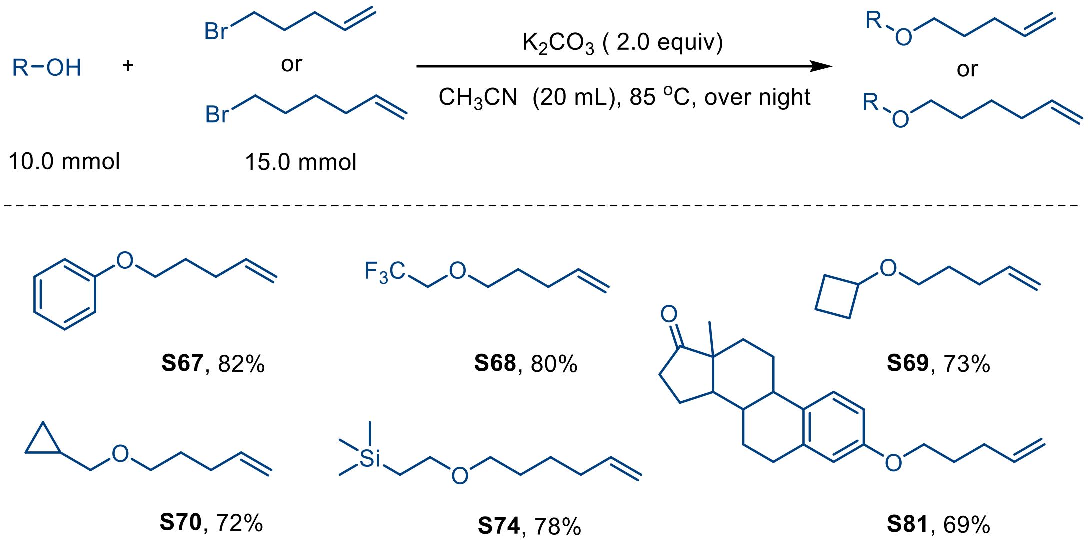

To a 50 mL round bottom flask with a magnetic stir bar was added alcohol (10.0 mmol), 5-bromopent-1-ene or 6-bromohex-1-ene (15.0 mmol), K_2CO_3 (2.0 mmol) and CH_3CN (20 mL). The reaction mixture was sealed with a stopper. After stirring for overnight at 85 ºC, the reaction mixture was poured into water (50 mL) and then extracted with DCM (20 mL × 3). The combined organic extract was dried over Mg_2SO_4, and the filtrate was concentrated in vacuo. The residue was purified by flash column chromatography (n-hexane : ethyl acetate = 50 : 1 to 20 : 1) to give the corresponding product.[2]

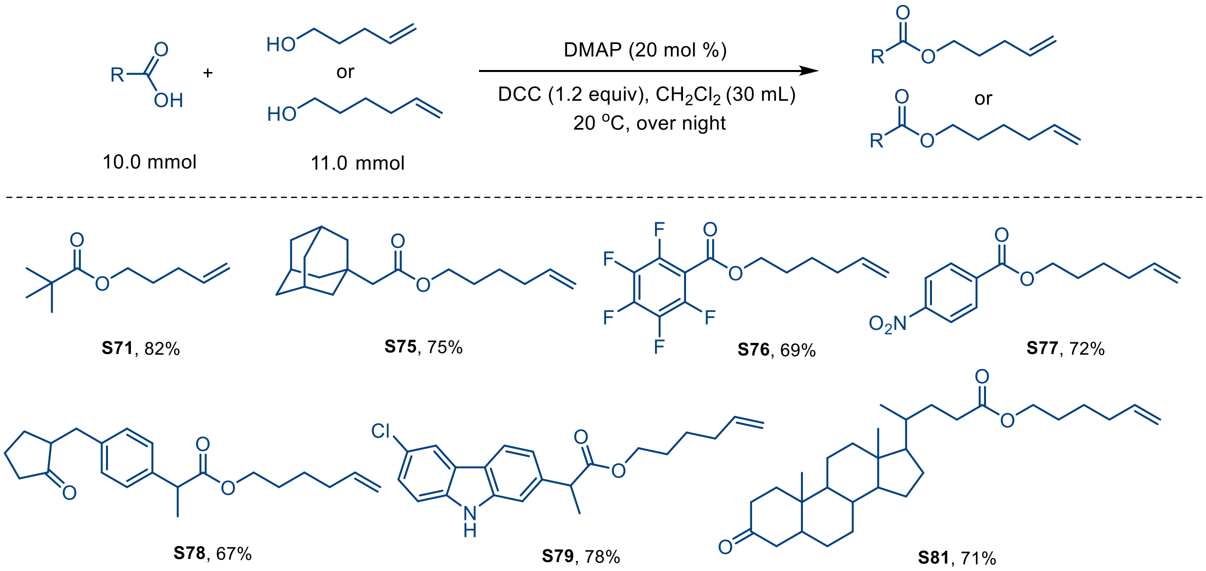

To a 50 mL round bottom flask with a magnetic stir bar was added acid (10.0 mmol), alcohol (11.0 mmol), DCC (*N, N'*-dicyclohexylcarbodiimide) (12.0 mmol), DMAP (2.0 mmol) and DCM (30 mL). The reaction mixture was sealed with a rubber septum. After stirring for overnight at 20 ºC, the reaction mixture was poured into water (50 mL) and then extracted with DCM (20 mL × 3). The combined organic layer was dried over Mg_2SO_4, and the filtrate was concentrated in vacuo. The residue was purified by flash column chromatography (n-hexane : ethyl acetate = 50 : 1 to 10 : 1) to give the corresponding product.[3]

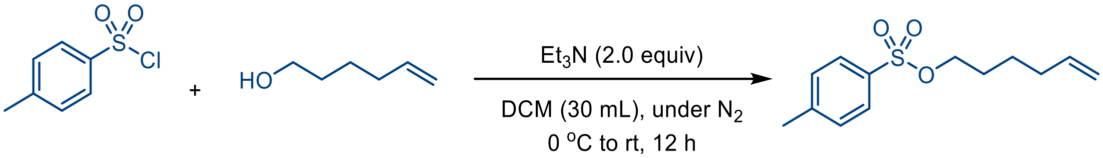

12.0 mmol   10.0 mmol   **S73**, 83%

To a 50 mL round bottom flask with a magnetic stir bar was added hex-5-en-1-ol (10.0 mmol) and DCM (20 mL). The reaction mixture was sealed with a rubber septum. After stirring 5 mins at 0 ºC, Et_3N (20.0 mmol) added dropwise with a syringe. Then p-toluenesulfonyl chloride (12.0 mmol) was diluted with DCM (10 mL) and added with a syringe over 20 min. The reaction mixture was stirred for further 12 hours at 20 ºC. After the reaction was completed, the reaction mixture was extracted with DCM (20 ml × 3). The combined organic layer was dried over Mg_2SO_4, and the filtrate was concentrated in vacuo. The residue was purified by flash column chromatography (n-hexane : ethyl acetate = 20 : 1) to give the corresponding product.[4]

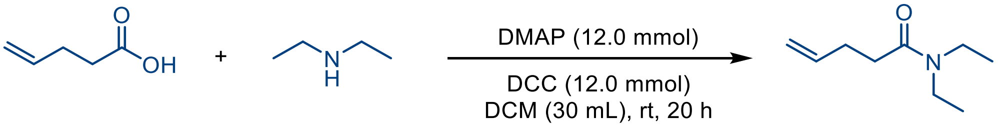

10.0 mmol   12.0 mmol   **S62**, 88%

To a 50 mL round bottom flask with a magnetic stir bar was added pent-4-enoic acid (10.0 mmol), diethylamine (12.0 mmol), DCC (*N, N'*-dicyclohexylcarbodiimide) (12.0 mmol), DMAP (12.0 mmol) and DCM (30 mL). The reaction mixture was sealed with a rubber septum. After stirring 20 hours at 20 ºC, reaction mixture was poured in water (50 mL) and then extracted with DCM (20 ml × 3). The combined organic layer was dried over Mg_2SO_4, and the filtrate was concentrated in vacuo. The residue was purified by flash column chromatography (n-hexane : ethyl acetate = 5 : 1) to give the corresponding product.[5]

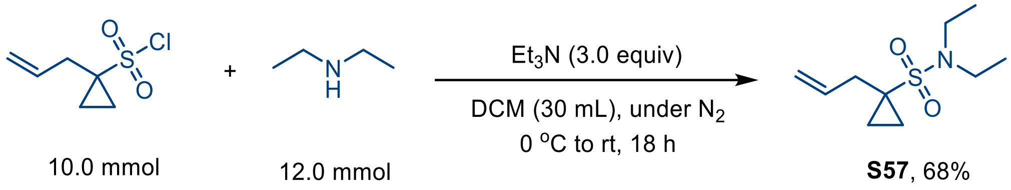

To a 50 mL round bottom flask with a magnetic stir bar was added diethylamine (12.0 mmol), Et_3N (30.0 mmol) DCM (30 mL). The reaction mixture was sealed with a rubber septum. Stirring 5 mins at 0 ºC before 1-allylcyclopropane-1-sulfonyl chloride (10.0 mmol) was added to the reaction mixture. After stirring 18 hours at 20 ºC, reaction mixture was poured in water (50 mL) and then extracted with DCM (20 mL × 3). The combined organic layer was dried over Mg_2SO_4, and the filtrate was concentrated in vacuo. The residue was purified by flash column chromatography (n-hexane : ethyl acetate = 5 : 1) to give the corresponding product.[6]

Physical characterization of alkenes

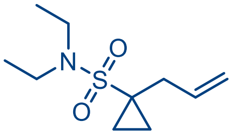

***N,N*-Diethyl-1-[[1-(2-propen-1-yl)cyclopropyl]sulfonamide (S57):** colorless oil. **^{1}H NMR** (400 MHz, $CDCl_3$) δ 5.69 (ddtd, *J* = 17.3, 10.2, 7.2, 1.1 Hz, 1H), 5.12 – 5.00 (m, 2H), 3.32 (qd, *J* = 7.1, 1.2 Hz, 4H), 2.56 (dd, *J* = 7.3, 1.4 Hz, 2H), 1.33 – 1.28 (m, 2H), 1.19 (td, *J* = 7.1, 1.2 Hz, 6H), 0.86 – 0.75 (m, 2H) ppm. **^{13}C NMR** (101 MHz, $CDCl_3$) δ 132.88, 118.71, 41.67, 39.24, 34.39, 14.33, 10.10 ppm. **HRMS** (ESI): calcd. for $C_{10}H_{20}NO_2S^+$: 218.1209. found: 218.1212.

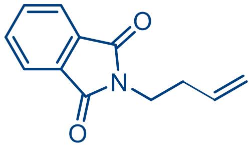

2-(But-3-en-1-yl)isoindoline-1,3-dione (S59): white solid. **^{1}H NMR** (400 MHz, $CDCl_3$) δ 7.88 – 7.78 (m, 2H), 7.78 – 7.67 (m, 2H), 5.78 (ddt, *J* = 17.1, 10.2, 6.9 Hz, 1H), 5.12 – 4.96 (m, 2H), 3.76 (t, *J* = 7.1 Hz, 2H), 2.44 (qt, *J* = 7.0, 1.3 Hz, 2H) ppm. **^{13}C NMR** (101 MHz, $CDCl_3$) δ 168.35, 134.48, 133.89, 132.08, 123.21, 117.56, 37.32, 32.84 ppm. **HRMS** (ESI): calcd. for $C_{12}H_{12}NO_2^+$: 202.0863. found: 202.0858.

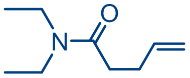

***N,N*-Diethylpent-4-enamide (S62):** colorless oil. **^{1}H NMR** (400 MHz, $CDCl_3$) δ 5.84 (tdd, *J* = 13.5, 5.3, 3.1 Hz, 1H), 5.09 – 4.89 (m, 2H), 3.32 (dqd, *J* = 28.1, 7.1, 2.4 Hz, 4H), 2.37 (q, *J* = 2.5 Hz, 4H), 1.11 (dtd, *J* = 25.3, 7.1, 2.5 Hz, 6H) ppm. **^{13}C NMR** (101 MHz, $CDCl_3$) δ 171.29, 137.73, 114.98, 41.89, 40.07, 32.36, 29.46, 14.32, 13.08 ppm. **HRMS** (ESI): calcd. for $C_9H_{18}NO^+$: 156.1383. found: 156.1386.

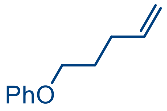

(Pent-4-en-1-yloxy)benzene (S67): colorless oil. **^{1}H NMR** (400 MHz, CDCl$_3$) δ 7.32 – 7.20 (m, 2H), 6.96 – 6.85 (m, 3H), 5.85 (ddt, *J* = 16.9, 10.2, 6.6 Hz, 1H), 5.11 – 4.95 (m, 2H), 3.96 (t, *J* = 6.5 Hz, 2H), 2.24 (q, *J* = 7.1 Hz, 2H), 1.93 – 1.82 (m, 2H) ppm. **^{13}C NMR** (101 MHz, CDCl$_3$) δ 159.07, 137.88, 129.43, 120.56, 115.17, 114.54, 67.07, 30.16, 28.49 ppm. **HRMS** (EI): calcd. for $C_{11}H_{14}O$: 162.1045. found: 162.1043.

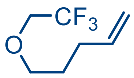

5-(2,2,2-Trifluoroethoxy)pent-1-ene (S68): colorless oil. **^{1}H NMR** (400 MHz, CDCl$_3$) δ 5.80 (ddt, *J* = 16.9, 10.2, 6.7 Hz, 1H), 5.09 – 4.93 (m, 2H), 3.80 (q, *J* = 8.8 Hz, 2H), 3.61 (t, *J* = 6.5 Hz, 2H), 2.14 (q, *J* = 7.1 Hz, 2H), 1.78 – 1.65 (m, 2H) ppm. **^{13}C NMR** (101 MHz, CDCl$_3$) δ 137.74, 125.47, 122.69, 115.12, 72.07, 68.82, 68.49, 68.15, 29.87, 28.63. **^{19}F NMR** (376 MHz, CDCl$_3$) δ -74.27 ppm. **HRMS** (EI): calcd. for $C_7H_{11}F_3O$: 168.0762. found: 168.0757.

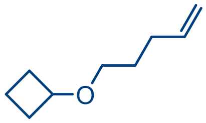

(Pent-4-en-1-yloxy)cyclobutane (S69): colorless oil. **^{1}H NMR** (400 MHz, CDCl$_3$) δ 5.87 – 5.69 (m, 1H), 5.12 – 4.78 (m, 3H), 4.12 (dt, *J* = 8.5, 6.6 Hz, 2H), 2.34 (dtq, *J* = 9.7, 7.6, 2.4 Hz, 1H), 2.18 – 2.01 (m, 4H), 1.86 – 1.68 (m, 3H), 1.65 – 1.51 (m, 1H) ppm. **^{13}C NMR** (101 MHz, CDCl$_3$) δ 154.33, 137.26, 115.42, 71.50, 67.28, 67.13, 30.08, 29.79, 27.83, 13.03 ppm. **HRMS** (EI): calcd. for $C_9H_{16}O$: 140.1201. found: 140.1198.

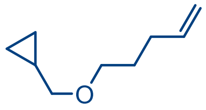

((Pent-4-en-1-yloxy)methyl)cyclopropane (S70): colorless oil. **^{1}H NMR** (400 MHz, CDCl$_3$) δ 5.79 (ddt, *J* = 16.9, 10.2, 6.6 Hz, 1H), 5.10 – 4.91 (m, 2H), 4.13 (td, *J* = 6.6, 1.2 Hz, 2H), 3.95 (d, *J* = 7.4 Hz, 2H), 2.20 – 2.06 (m, 2H), 1.81 – 1.68 (m, 2H), 1.16 (tt, *J* = 11.1, 3.9 Hz, 1H), 0.66 – 0.53 (m, 2H), 0.33 – 0.22 (m, 1H) ppm. **^{13}C NMR** (101 MHz, CDCl$_3$) δ 155.37, 137.29, 137.26, 115.41, 72.77, 67.28, 67.24, 29.79, 27.85, 27.83, 9.76, 3.25 ppm. **HRMS** (EI): calcd. for $C_9H_{16}O$: 140.1201. found: 1401205.

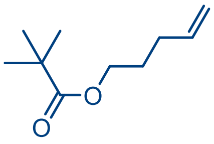

Pent-4-en-1-yl pivalate (S71): colorless oil. **^{1}H NMR** (400 MHz, CDCl$_3$) δ 5.80 (ddt, *J* = 17.0, 10.2, 6.7 Hz, 1H), 5.12 – 4.87 (m, 2H), 4.05 (t, *J* = 6.5 Hz, 2H), 2.16 – 2.06 (m, 2H), 1.76 – 1.69 (m, 2H), 1.19 (s, 9H) ppm. **^{13}C NMR** (101 MHz, CDCl$_3$) δ 178.57, 137.55, 115.24, 63.66, 38.74, 30.08, 27.83, 27.20 ppm. **HRMS** (EI): calcd. for $C_{10}H_{18}O_2$: 170.1307. found: 170.1301.

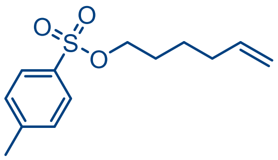

Hex-5-en-1-yl 4-methylbenzenesulfonate (S73): colorless oil. **^{1}H NMR** (400 MHz, CDCl$_3$) δ 7.81 – 7.73 (m, 2H), 7.34 (d, *J* = 8.0 Hz, 2H), 5.79 – 5.63 (m, 1H), 4.98 – 4.89 (m, 2H), 4.02 (t, *J* = 6.4 Hz, 2H), 2.45 (s, 3H), 2.02 – 1.94 (m, 2H), 1.72 – 1.62 (m, 2H), 1.45 – 1.36 (m, 2H) ppm. **^{13}C**

NMR (101 MHz, $CDCl_3$) δ 144.68, 137.91, 133.21, 129.82, 127.89, 115.06, 70.44, 32.91, 28.20, 24.55, 21.63 ppm. **HRMS** (EI): calcd. for $C_{13}H_{18}O_3S$: 254.0977. found: 254.0979.

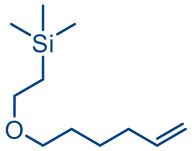

(2-(Hex-5-en-1-yloxy)ethyl)trimethylsilane (S74): colorless oil. **^{1}H NMR** (400 MHz, $CDCl_3$) δ 5.77 (ddt, *J* = 17.0, 10.2, 6.7 Hz, 1H), 5.09 – 4.88 (m, 2H), 4.31 – 4.16 (m, 2H), 4.11 (t, *J* = 6.6 Hz, 2H), 2.07 (q, *J* = 7.2 Hz, 2H), 1.74 – 1.62 (m, 2H), 1.50 – 1.40 (m, 2H), 1.11 – 0.96 (m, 2H), 0.03 (s, 9H) ppm. **^{13}C NMR** (101 MHz, $CDCl_3$) δ 155.36, 138.23, 114.85, 67.59, 66.20, 33.23, 28.12, 24.96, 17.54, -1.57 ppm. **HRMS** (EI): calcd. for $C_{11}H_{24}OSi$: 200.1596. found: 200.1604.

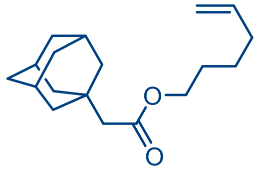

Hex-5-en-1-yl 2-((3r,5r,7r)-adamantan-1-yl)acetate (S75): colorless oil. **^{1}H NMR** (400 MHz, $CDCl_3$) δ 5.79 (ddt, *J* = 16.9, 10.1, 6.7 Hz, 1H), 5.07 – 4.92 (m, 2H), 4.05 (t, *J* = 6.6 Hz, 2H), 2.12 – 2.04 (m, 4H), 1.96 (q, *J* = 3.2 Hz, 3H), 1.73 – 1.59 (m, 15H), 1.51 – 1.40 (m, 2H) ppm. **^{13}C NMR** (101 MHz, $CDCl_3$) δ 171.94, 138.38, 114.78, 63.84, 49.05, 42.42, 36.75, 33.27, 32.74, 28.63, 28.14, 25.31 ppm. **HRMS** (EI): calcd. for $C_{18}H_{28}O_2$: 276.2089. found: 276.2084.

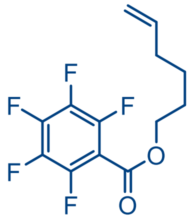

Hex-5-en-1-yl 2,3,4,5,6-pentafluorobenzoate (S76): colorless oil. **^{1}H NMR** (400 MHz, $CDCl_3$) δ 5.87 – 5.73 (m, 1H), 5.08 – 4.94 (m, 2H), 4.39 (t, *J* = 6.5 Hz, 2H), 2.16 – 2.07 (m, 2H), 1.77 (dq, *J* = 8.6, 6.6 Hz, 2H), 1.58 – 1.49 (m, 2H) ppm. **^{13}C NMR** (101 MHz, $CDCl_3$) δ 159.07, 138.06, 115.00, 66.75, 33.11, 27.80, 24.99 ppm. **^{19}F NMR** (376 MHz, $CDCl_3$) δ -138.40 – -138.54 (m), -149.04 (tt, *J* = 20.9, 4.4 Hz), -160.46 – -160.66 (m) ppm. **HRMS** (EI): calcd. for $C_{14}H_{11}F_5O_2$: 294.0679. found: 294.0668.

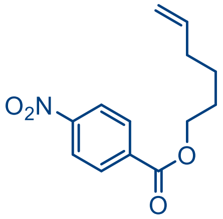

Hex-5-en-1-yl 4-nitrobenzoate (S77): pale yellow oil. **^{1}H NMR** (400 MHz, $CDCl_3$) δ 8.28 (dd, *J* = 8.9, 2.6 Hz, 2H), 8.25 – 8.15 (m, 2H), 5.89 – 5.74 (m, 1H), 5.10 – 4.92 (m, 2H), 4.37 (td, *J* = 6.6, 1.9 Hz, 2H), 2.19 – 2.10 (m, 2H), 1.87 – 1.75 (m, 2H), 1.61 – 1.50 (m, 2H) ppm. **^{13}C NMR** (101 MHz, $CDCl_3$) δ 164.72, 150.51, 138.13, 135.82, 130.66, 123.53, 115.06, 65.88, 33.25, 28.04, 25.23 ppm. **HRMS** (EI): calcd. for $C_{13}H_{15}NO_4$: 249.1001. found: 249.0998.

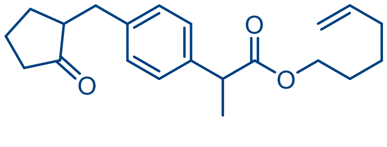

Hex-5-en-1-yl 2-(4-((2-oxocyclopentyl)methyl)phenyl)propanoate (S78): colorless oil. **^{1}H NMR** (400 MHz, $CDCl_3$) δ 7.23 – 7.17 (m, 2H), 7.13 – 7.07 (m, 2H), 5.73 (ddt, *J* = 16.9, 10.2, 6.6 Hz, 1H), 5.00 – 4.90 (m, 2H), 4.06 (t, *J* = 6.6 Hz, 2H), 3.68 (q, *J* = 7.2 Hz, 1H), 3.12 (dd, *J* = 13.9, 4.1 Hz, 1H), 2.50 (dd, *J* = 13.9, 9.5 Hz, 1H), 2.37 – 2.28

(m, 2H), 2.14 – 1.95 (m, 5H), 1.78 – 1.68 (m, 1H), 1.57 (ddd, *J* = 14.5, 6.8, 5.2 Hz, 3H), 1.47 (d, *J* = 7.2 Hz, 3H), 1.35 (tt, *J* = 9.9, 6.4 Hz, 2H) ppm. **^{13}C NMR** (101 MHz, $CDCl_3$) δ 174.65, 138.79, 138.49, 138.33, 129.07, 127.52, 114.73, 64.58, 50.98, 45.18, 38.18, 35.19, 33.17, 29.21, 27.96, 25.04, 20.54, 18.43 ppm. **HRMS** (EI): calcd. for $C_{21}H_{28}O_3$: 328.2038. found: 328.2042.

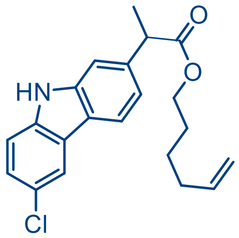

Hex-5-en-1-yl 2-(6-chloro-9H-carbazol-2-yl)propanoate (S79): colorless oil. **^{1}H NMR** (400 MHz, $CDCl_3$) δ 8.18 (s, 1H), 7.99 – 7.88 (m, 2H), 7.39 – 7.26 (m, 3H), 7.17 (dd, *J* = 8.2, 1.5 Hz, 1H), 5.70 (ddt, *J* = 16.9, 10.2, 6.6 Hz, 1H), 5.00 – 4.85 (m, 2H), 4.09 (td, *J* = 6.6, 2.8 Hz, 2H), 3.87 (q, *J* = 7.1 Hz, 1H), 2.03 – 1.95 (m, 2H), 1.62 – 1.55 (m, 5H), 1.39 – 1.32 (m, 2H) ppm. **^{13}C NMR** (101 MHz, $CDCl_3$) δ 174.97, 140.37, 139.35, 138.31, 138.07, 125.78, 124.91, 124.32, 121.61, 120.55, 119.95, 119.68, 114.74, 111.57, 109.55, 64.84, 46.01, 33.17, 27.99, 25.06, 18.85 ppm. **HRMS** (ESI): calcd. for $C_{21}H_{23}ClNO_2^+$: 356.1412. found: 356.1415.

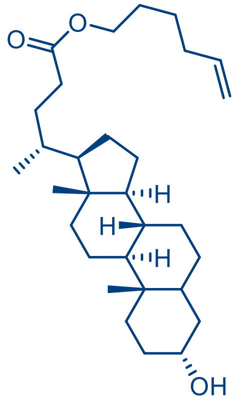

Hex-5-en-1-yl (4R)-4-((3R,8R,9S,10S,13R,14S,17R)-3-hydroxy-10,13-dimethylhexadecahydro-1H-cyclopenta[a]phenanthren-17-yl)pentanoate (S80): colorless oil. **^{1}H NMR** (400 MHz, $CDCl_3$) δ 5.77 (ddt, *J* = 16.9, 10.2, 6.7 Hz, 1H), 4.97 (dd, *J* = 20.2, 13.6 Hz, 2H), 4.69 (tt, *J* = 10.9, 4.7 Hz, 1H), 4.07 (dt, *J* = 25.6, 6.9 Hz, 3H), 3.59 (tt, *J* = 10.8, 4.7 Hz, 1H), 2.30 (dd, *J* = 10.0, 5.1 Hz, 2H), 2.22 – 2.12 (m, 2H), 2.10 – 2.00 (m, 4H), 1.99 – 1.91 (m, 2H), 1.87 – 1.72 (m, 12H), 1.62 (q, *J* = 7.9, 7.4 Hz, 5H), 1.48 – 1.31 (m, 18H), 1.24 (h, *J* = 7.7, 6.7 Hz, 9H), 1.06 (tq, *J* = 15.2, 9.7, 7.0 Hz, 11H), 0.89 (t, *J* = 4.4 Hz, 12H), 0.62 (s, 6H) ppm. **^{13}C NMR** (101 MHz, $CDCl_3$) δ 174.37, 138.33, 114.81, 74.06, 71.76, 64.17, 56.51, 56.08, 42.74, 42.10, 41.87, 40.43, 40.17, 36.43, 35.86, 35.78, 35.36, 35.29, 35.05, 34.57, 33.28, 32.28, 31.72, 31.34, 31.03, 30.52, 28.18, 28.09, 27.21, 27.02, 26.66, 26.44, 26.30, 25.21, 24.24, 24.19, 23.38, 23.33, 20.82, 18.27, 12.08, 12.04 ppm. **HRMS** (ESI): calcd. for $C_{30}H_{51}O_3^+$: 459.3833. found: 459.3839.

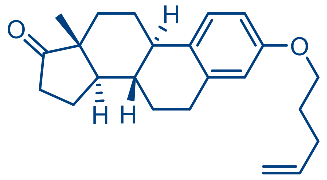

(8S,9R,13R,14R)-13-Methyl-3-(pent-4-en-1-yloxy)-6,7,8,9,11,12,13,14,15,16-decahydro-17H-cyclopenta[a]phenanthren-17-one (S81): white solid. **^{1}H NMR** (400 MHz, $CDCl_3$) δ 7.19 (d, *J* = 8.6 Hz, 1H), 6.72 (dd, *J* = 8.5, 2.8 Hz, 1H), 6.65 (d, *J* = 2.7 Hz, 1H), 5.86 (ddt, *J* = 16.9, 10.2, 6.6 Hz, 1H), 5.12 – 4.96 (m, 2H), 3.95 (t, *J* = 6.4 Hz, 2H), 2.95 – 2.86 (m, 2H), 2.58 – 2.37 (m, 2H), 2.24 (q, *J* = 6.9 Hz, 3H), 2.17 – 1.80 (m, 6H), 1.66 – 1.39 (m, 7H), 0.91 (s, 3H) ppm. **^{13}C NMR** (101 MHz, $CDCl_3$) δ 157.09, 137.91, 137.71, 131.93, 126.30, 115.13, 114.59, 112.15, 67.10,

50.44, 48.03, 44.01, 38.41, 35.89, 31.61, 30.15, 29.67, 28.53, 26.59, 25.94, 21.61, 13.87 ppm.
HRMS (EI): calcd. for $C_{23}H_{30}O_2$: 338.2246. found: 338.2251.

5. Preparation of 117[7] and DBpin[8]

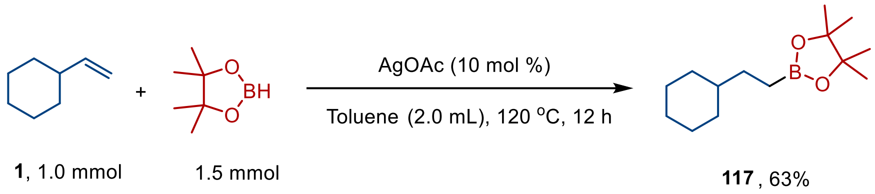

In a nitrogen-filled glovebox, to a 10 mL vial equipped with a magnetic stir was added vinylcyclohexane **1** (1.0 mmol), 4,4,5,5-tetramethyl-1,3,2-dioxaborolane (1.5 mmol), AgOAc (0.1 mmol) and toluene (2.0 mL). Then the 10 mL vial was capped and removed from the glovebox. The reaction mixture was stirred at 120 °C for 12 hours. After that, the reaction mixture was cooled to room temperature and diluted with ethyl acetate. The solution was then filtered through a pad of Celite®. The combined organic layer was dried over $MgSO_4$ and concentrated in vacuo. The residue was purified by flash column chromatography (n-hexane) to give the corresponding product **117**.

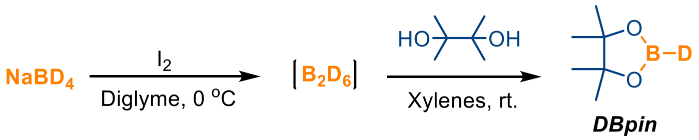

In a nitrogen-filled glovebox, to a three neck 100 mL round bottom flask with a magnetic stir bar was fitted with a dropping funnel. $NaBD_4$ (0.5 g, 12.0 mmol) and diethylene glycol dimethyl ether (10 mL) were added to the flask. Then iodine (1.6 g, 6.3 mmol) and diethylene glycol dimethyl ether (10 mL) were added to the funnel. To another 50 mL round flask with a magnetic stir bar was added pinacol (0.48 g, 4.0 mmol) and anhydrous xylenes (3 mL). The two flasks were sealed with rubber septum and connected with a double tipped needle. The whole reaction unit was then removed from the glovebox and the 50 mL round flask was connected to a nitrogen-filled balloon. Iodine solution was added dropwise to the reaction mixture over 1h at 0 °C and the resulting gas (B_2D_6) was vented to the pinacol solution (50 mL flask) through the needle. After completion of addition of the iodine solution, the 100 mL flask was allowed to stir at room temperature for another 1h until no bubble could be observed in the 50 mL flask. Finally, DBpin was given in xylenes solution.

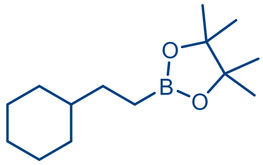

2-(2-Cyclohexylethyl)-4,4,5,5-tetramethyl-1,3,2-dioxaborolane (117): colorless oil (63%). **^{1}H NMR** (400 MHz, $CDCl_3$) δ 1.78 – 1.64 (m, 4H), 1.36 – 1.01 (m, 19H), 0.89 – 0.70 (m, 4H) ppm. **^{13}C NMR** (101 MHz, $CDCl_3$) δ

82.82, 39.95, 32.98, 31.36, 26.77, 26.44, 24.80 ppm. **HRMS** (ESI): calcd. for $C_{14}H_{28}BO_2^+$: 239.2177. found: 239.2172.

6. General procedures for Ni-catalyzed hydroamidation

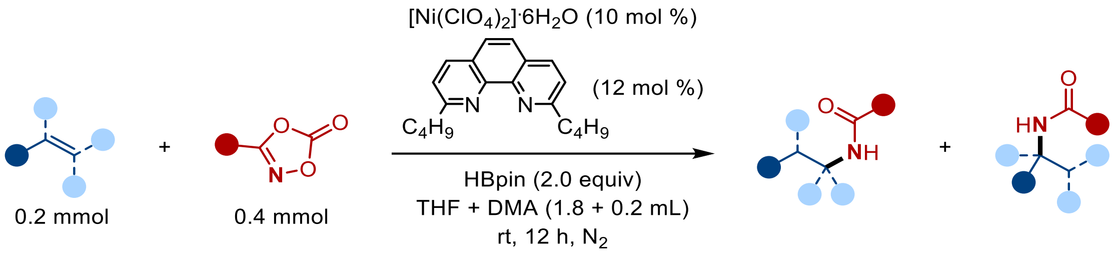

In a nitrogen-filled glovebox, a 8 mL vial containing a magnetic stir bar was charged with $Ni(ClO_4)_2·6H_2O$ (7.3 mg, 10 mol %) and 2,9-dibutyl-1,10-phenanthroline (7.0 mg, 12 mol %). Anhydrous THF (1.8 mL) and DMA (0.2 mL) were then added to the mixture via syringes. The vial was then screw-capped and stirred for 10 min at room temperature to give a brownish yellow solution.

Alkene (0.20 mmol, 1.0 equiv) and 1,4,2-dioxazol-5-one (0.40 mmol, 2.0 equiv) were added to a separate 8 mL vial with a magnetic stirrer bar. The [Ni + L] standard solution was then transferred to the alkene-dioxazolone mixture with vigorous stirring. 4,4,5,5-Tetramethyl-1,3,2-dioxaborolane [HBpin, (0.40 mmol, 2.0 equiv)] was then added dropwise to the mixture via a syringe. A dark green solution mixture would appear upon addition of HBpin. The vial was then capped and removed from the glovebox. The mixture was then stirred at room temperature for 12 h. [*The color of the solution will turn paler following the reaction time (dark green → green → yellow → pale yellow)*].

The crude mixture was transferred to a 25 mL round bottom flask and concentrated in vacuo. The residue was then filtered through a short-packed column with silica gel with ethyl acetate as eluent. The residue was again concentrated in vacuo. The residue was purified by column chromatography (n-hexane : ethyl acetate = 5 : 1 to 1 : 1), and the desired amide product can be visualized by TLC using $KMnO_4$ stain.

Physical characterization of hydroamidation products

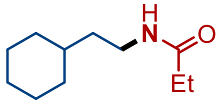

***N*-(2-Cyclohexylethyl)propionamide (5):** white solid (50%). **M.P.** 69–70 ºC. **^{1}H NMR** (400 MHz, $CDCl_3$) δ 5.51 (s, 1H), 3.32 – 3.14 (m, 2H), 2.16 (tt, J = 7.6, 4.4 Hz, 2H), 1.76 – 1.61 (m, 5H), 1.40 – 1.32 (m, 2H), 1.22 (d, J = 2.7 Hz, 3H), 1.13 (td, J = 7.7, 2.8 Hz, 4H), 0.89 (tq, J = 10.7, 7.5, 5.2 Hz, 2H) ppm. **^{13}C NMR** (101 MHz, $CDCl_3$) δ 173.65, 37.32, 37.11, 35.40, 33.14, 29.75, 26.46, 26.18, 24.81, 9.91 ppm. **HRMS** (ESI): calcd. for $C_{11}H_{22}NO^+$: 184.1696. found: 184.1699.

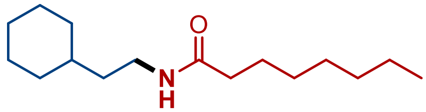

***N*-(2-Cyclohexylethyl)octanamide (6):** white solid (80%). **M.P.** 42–43 ºC. **^{1}H NMR** (400 MHz, $CDCl_3$) δ 5.54 (t, J = 5.6 Hz, 1H), 3.29 – 3.20 (m, 2H), 2.16 – 2.09 (m, 2H), 1.76 – 1.60 (m, 6H), 1.39 – 1.06 (m, 15H), 0.91 – 0.79 (m, 5H) ppm. **^{13}C NMR** (101 MHz, $CDCl_3$) δ 173.06, 37.28, 37.14, 36.90, 35.40, 33.16, 31.70, 29.26, 29.02, 26.49, 26.21, 25.85, 22.59, 14.04 ppm. **HRMS** (ESI): calcd. for $C_{16}H_{32}NO^+$: 254.2478. found: 254.2482.

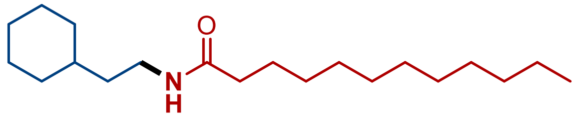

***N*-(2-Cyclohexylethyl)dodecanamide (7):** white solid (77%). **M.P.** 59–60 ºC. **^{1}H NMR** (400 MHz, $CDCl_3$) δ 5.65 – 5.53 (m, 1H), 3.28 – 3.20 (m, 2H), 2.16 – 2.08 (m, 2H), 1.71 – 1.56 (m, 7H), 1.36 (dt, J = 8.9, 6.9 Hz, 2H), 1.30 – 1.13 (m, 20H), 0.93 – 0.80 (m, 5H) ppm. **^{13}C NMR** (101 MHz, $CDCl_3$) δ 173.06, 37.28, 37.13, 36.89, 35.40, 33.16, 31.89, 29.61, 29.59, 29.51, 29.38, 29.31, 26.49, 26.21, 25.85, 22.66, 14.08 ppm. **HRMS** (ESI): calcd. for $C_{20}H_{40}NO^+$: 310.3104. found: 310.3106.

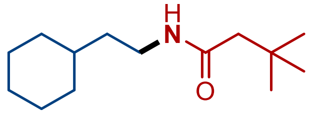

***N*-(2-Cyclohexylethyl)-3,3-dimethylbutanamide (8):** white solid (75%). **M.P.** 55–57 ºC. **^{1}H NMR** (400 MHz, $CDCl_3$) δ 5.47 (s, 1H), 3.28 – 3.19 (m, 2H), 2.01 (s, 2H), 1.72 – 1.59 (m, 4H), 1.41 – 1.32 (m, 2H), 1.29 – 1.10 (m, 3H), 1.01 (s, 9H), 0.94 – 0.83 (m, 2H) ppm. **^{13}C NMR** (101 MHz, $CDCl_3$) δ 171.70, 50.83, 37.31, 37.29, 35.54, 33.25, 30.90, 29.94, 26.61, 26.34 ppm. **HRMS** (ESI): calcd. for $C_{14}H_{28}NO^+$: 226.2176. found: 226.2171.

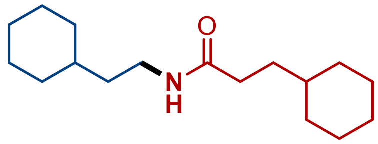

3-Cyclohexyl-*N*-(2-cyclohexylethyl)propanamide (9): white solid (86%). **M.P.** 77–78 ºC. **^{1}H NMR** (400 MHz, $CDCl_3$) δ 5.50 (d, J = 5.8 Hz, 1H), 3.30 – 3.18 (m, 2H), 2.03 (tt, J = 11.8, 3.4 Hz, 1H), 1.87 – 1.73 (m, 4H), 1.65 (tdd, J = 15.8, 7.6, 3.6 Hz, 6H), 1.47 – 1.32 (m, 4H), 1.32 – 1.09 (m, 7H), 0.89 (qd, J = 13.9, 12.6, 3.5 Hz, 2H) ppm. **^{13}C NMR** (101 MHz,

$CDCl_3$) δ 173.28, 37.36, 37.29, 37.15, 35.39, 34.38, 33.23, 33.17, 33.09, 26.55, 26.50, 26.24, 26.22 ppm. **HRMS** (ESI): calcd. for $C_{17}H_{32}NO^+$: 266.2478. found: 266.2483.

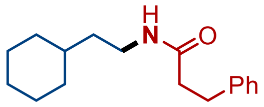

***N*-(2-Cyclohexylethyl)-3-phenylpropanamide (10):** white solid (78%). **M.P.** 60–61 ºC. **^{1}H NMR** (400 MHz, $CDCl_3$) δ 7.32 – 7.25 (m, 2H), 7.19 (dt, *J* = 7.4, 3.1 Hz, 3H), 5.47 (t, *J* = 5.7 Hz, 1H), 3.28 – 3.17 (m, 2H), 2.96 (t, *J* = 7.7 Hz, 2H), 2.46 (dd, *J* = 8.4, 7.0 Hz, 2H), 1.72 – 1.60 (m, 6H), 1.31 (q, *J* = 7.0 Hz, 2H), 1.24 – 1.11 (m, 4H), 0.95 – 0.80 (m, 2H) ppm. **^{13}C NMR** (101 MHz, $CDCl_3$) δ 171.95, 140.94, 128.48, 128.32, 126.18, 38.52, 37.28, 36.99, 35.22, 33.09, 31.78, 26.47, 26.16 ppm. **HRMS** (ESI): calcd. for $C_{17}H_{26}NO^+$: 260.2009. found: 260.2010.

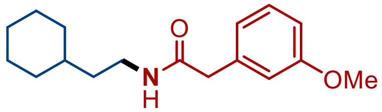

***N*-(2-Cyclohexylethyl)-2-(3-methoxyphenyl)acetamide (11):** colorless oil (60%). **^{1}H NMR** (400 MHz, $CDCl_3$) δ 7.27 (t, *J* = 7.9 Hz, 1H), 6.95 – 6.64 (m, 3H), 5.39 (s, 1H), 3.81 (s, 3H), 3.53 (s, 2H), 3.29 – 3.16 (m, 2H), 1.71 – 1.57 (m, 5H), 1.36 – 1.25 (m, 2H), 1.25 – 1.06 (m, 4H), 0.94 – 0.74 (m, 2H) ppm. **^{13}C NMR** (101 MHz, $CDCl_3$) δ 170.65, 160.03, 136.57, 130.02, 121.70, 115.02, 112.82, 55.21, 43.97, 37.55, 36.82, 35.41, 33.09, 26.44, 26.17 ppm. **HRMS** (ESI): calcd. for $C_{17}H_{26}NO_2^+$: 276.1958. found: 276.1959.

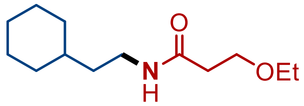

***N*-(2-Cyclohexylethyl)-3-ethoxypropanamide (12):** colorless oil (62%). **^{1}H NMR** (400 MHz, $CDCl_3$) δ 6.27 (s, 1H), 3.66 (t, *J* = 5.8 Hz, 2H), 3.52 (q, *J* = 7.0 Hz, 2H), 3.27 (td, *J* = 7.4, 5.6 Hz, 2H), 2.44 (t, *J* = 5.8 Hz, 2H), 1.80 – 1.65 (m, 5H), 1.39 (q, *J* = 6.9 Hz, 2H), 1.28 – 1.12 (m, 7H), 0.96 – 0.84 (m, 2H) ppm. **^{13}C NMR** (101 MHz, $CDCl_3$) δ 171.47, 66.81, 66.63, 66.46, 37.11, 37.07, 36.94, 35.35, 33.14, 26.49, 26.22, 15.10 ppm. **HRMS** (ESI): calcd. for $C_{13}H_{26}NO_2^+$: 228.1958. found: 228.1953.

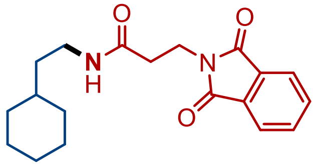

***N*-(2-Cyclohexylethyl)-3-(1,3-dioxoisoindolin-2-yl)propanamide (13):** white solid (83%). **M.P.** 191–192 ºC. **^{1}H NMR** (400 MHz, $CDCl_3$) δ 7.89 – 7.79 (m, 2H), 7.76 – 7.67 (m, 2H), 5.65 (s, 1H), 4.00 (t, *J* = 7.3 Hz, 2H), 3.32 – 3.21 (m, 2H), 2.61 (t, *J* = 7.3 Hz, 2H), 1.73 – 1.60 (m, 7H), 1.40 – 1.29 (m, 2H), 1.26 – 1.11 (m, 4H), 0.92 – 0.81 (m, 2H) ppm. **^{13}C NMR** (101 MHz, $CDCl_3$) δ 169.50, 168.13, 134.01, 131.97, 123.27, 37.37, 36.88, 35.26, 34.91, 34.45, 33.06, 26.43, 26.13 ppm. **HRMS** (ESI): calcd. for $C_{19}H_{25}N_2O_3^+$: 329.1865. found: 329.1859.

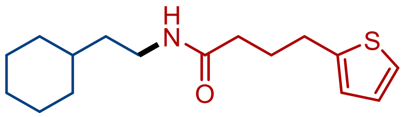

***N*-(2-Cyclohexylethyl)-4-(thiophen-2-yl)butanamide (14):** colorless oil (82%). **^{1}H NMR** (400 MHz, $CDCl_3$) δ 7.09 (dd, *J* = 5.1, 1.2 Hz, 1H), 6.89 (dd, *J* = 5.1, 3.4 Hz, 1H), 6.77 (d, *J* = 3.5 Hz, 1H), 5.66 (t, *J* = 5.7 Hz, 1H), 3.34 – 3.12 (m, 2H), 2.85 (t, *J* = 7.3 Hz, 2H), 2.18 (t, *J* = 7.4 Hz, 2H), 1.99 (p, *J* = 7.4 Hz, 2H), 1.78 – 1.57 (m, 7H), 1.36 (q, *J* = 7.0 Hz, 2H), 1.30 – 1.07 (m, 4H), 0.89 (qd, *J* = 10.6, 9.7, 4.8 Hz, 3H) ppm. **^{13}C NMR** (101 MHz, $CDCl_3$) δ 172.37, 144.33, 126.78, 124.47, 123.14, 37.35, 37.11, 35.61, 35.39, 33.16, 29.18, 27.53, 26.50, 26.22, 24.84 ppm. **HRMS** (ESI): calcd. for $C_{16}H_{26}NOS^+$: 280.1730. found: 280.1733.

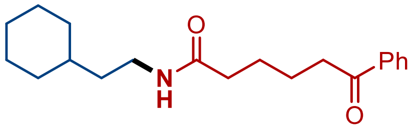

***N*-(2-Cyclohexylethyl)-6-oxo-6-phenylhexanamide (15):** white solid (71%). **M.P.** 65–66 ºC. **^{1}H NMR** (400 MHz, $CDCl_3$) δ 7.94 (d, *J* = 7.6 Hz, 2H), 7.54 (t, *J* = 7.3 Hz, 1H), 7.44 (t, *J* = 7.6 Hz, 2H), 5.70 (t, *J* = 5.6 Hz, 1H), 3.26 (q, *J* = 6.8 Hz, 2H), 2.99 (t, *J* = 6.6 Hz, 2H), 2.21 (t, *J* = 6.9 Hz, 2H), 1.78 – 1.61 (m, 9H), 1.38 (q, *J* = 7.2 Hz, 2H), 1.31 – 1.08 (m, 4H), 0.95 – 0.83 (m, 2H) ppm. **^{13}C NMR** (101 MHz, $CDCl_3$) δ 200.17, 172.53, 136.92, 133.05, 128.60, 128.02, 38.16, 37.37, 37.08, 36.59, 35.40, 33.16, 26.50, 26.21, 25.31, 23.69 ppm. **HRMS** (ESI): calcd. for $C_{20}H_{30}NO_2^+$: 316.2271. found: 316.2273.

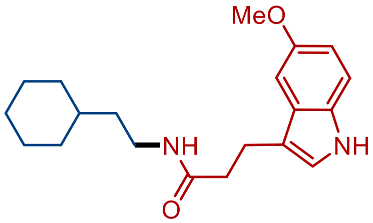

***N*-(2-Cyclohexylethyl)-3-(5-methoxy-1H-indol-3-yl)propanamide (16):** white solid (78%). **M.P.** 130–131 ºC. **^{1}H NMR** (400 MHz, $CDCl_3$) δ 8.13 (s, 1H), 7.27 – 7.20 (m, 1H), 7.03 (d, *J* = 2.5 Hz, 1H), 6.95 (d, *J* = 2.4 Hz, 1H), 6.85 (dd, *J* = 8.8, 2.4 Hz, 1H), 5.37 (t, *J* = 5.5 Hz, 1H), 3.85 (s, 3H), 3.26 – 3.13 (m, 2H), 3.07 (t, *J* = 7.4 Hz, 2H), 2.54 (t, *J* = 7.4 Hz, 2H), 1.66 – 1.62 (m, 3H), 1.25 (q, *J* = 7.0 Hz, 3H), 1.14 (qd, *J* = 12.8, 11.4, 5.9 Hz, 4H), 0.85 (ddt, *J* = 14.4, 9.8, 3.2 Hz, 3H) ppm. **^{13}C NMR** (101 MHz, $CDCl_3$) δ 172.64, 153.93, 131.54, 127.52, 122.55, 114.68, 112.14, 111.96, 100.62, 55.99, 37.33, 37.31, 36.96, 35.26, 33.10, 26.48, 26.19, 21.42 ppm. **HRMS** (ESI): calcd. for $C_{20}H_{29}N_2O_2^+$: 329.2229. found: 329.2226.

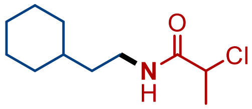

2-Chloro-N-(2-cyclohexylethyl)propanamide (17): pale brown solid (71%). **M.P.** 56–57 ºC. **^{1}H NMR** (400 MHz, $CDCl_3$) δ 6.53 (s, 1H), 4.39 (q, *J* = 7.0 Hz, 1H), 3.35 – 3.20 (m, 2H), 1.70 (dd, *J* = 10.9, 5.4 Hz, 8H), 1.42 (q, *J* = 7.1 Hz, 2H), 1.33 – 1.11 (m, 4H), 0.91 (qd, *J* = 13.7, 12.6, 3.6 Hz, 2H) ppm. **^{13}C NMR** (101 MHz, $CDCl_3$) δ 169.31, 56.16, 37.83, 36.76, 35.41, 33.12, 26.45, 26.17, 22.82 ppm. **HRMS** (ESI): calcd. for $C_{11}H_{21}ClNO_2^+$: 218.1306. found: 218.1309.

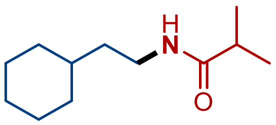

***N*-(2-Cyclohexylethyl)isobutyramide (18):** white solid (89%). **M.P.** 62–63 ºC. **^{1}H NMR** (400 MHz, $CDCl_3$) δ 5.59 (s, 1H), 3.26 – 3.19 (m, 2H), 2.31 (hept, *J* = 6.9 Hz, 1H), 1.71 – 1.57 (m, 5H), 1.35 (dd, *J* = 8.1, 6.8 Hz, 2H), 1.30 – 1.13 (m, 4H), 1.12 (s, 3H), 1.10 (s, 3H), 0.94 – 0.82 (m, 2H) ppm. **^{13}C NMR** (101 MHz, $CDCl_3$) δ 176.95, 37.33, 37.24, 35.74, 35.57, 33.27, 26.59, 26.31, 19.75 ppm. **HRMS** (ESI): calcd. for $C_{12}H_{24}NO^+$: 198.1863. found: 198.1857.

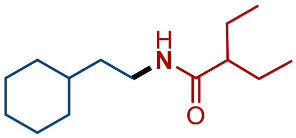

***N*-(2-Cyclohexylethyl)-2-ethylbutanamide (19):** white solid (77%). **M.P.** 94–95 ºC. **^{1}H NMR** (400 MHz, $CDCl_3$) δ 5.48 (s, 1H), 3.31 – 3.22 (m, 2H), 1.80 (tt, *J* = 9.4, 5.1 Hz, 1H), 1.73 – 1.55 (m, 7H), 1.41 (ddt, *J* = 26.3, 8.8, 6.1 Hz, 4H), 1.31 – 1.10 (m, 4H), 0.87 (q, *J* = 8.3, 7.4 Hz, 8H) ppm. **^{13}C NMR** (101 MHz, $CDCl_3$) δ 175.55, 51.62, 37.26, 37.08, 35.40, 33.15, 26.50, 26.24, 25.80, 12.11 ppm. **HRMS** (ESI): calcd. for $C_{14}H_{28}NO^+$: 226.2165. found: 226.2169.

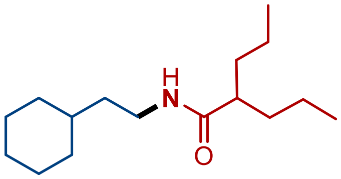

***N*-(2-Cyclohexylethyl)-2-propylpentanamide (20):** white solid (70%). **M.P.** 110–111 ºC. **^{1}H NMR** (400 MHz, $CDCl_3$) δ 5.47 (d, *J* = 5.7 Hz, 1H), 3.30 – 3.22 (m, 2H), 1.98 (tt, *J* = 9.5, 4.4 Hz, 1H), 1.72 – 1.51 (m, 7H), 1.40 – 1.10 (m, 12H), 0.88 (q, *J* = 6.9, 6.4 Hz, 8H) ppm. **^{13}C NMR** (101 MHz, $CDCl_3$) δ 175.81, 47.78, 37.19, 37.07, 35.40, 35.32, 33.15, 26.50, 26.24, 20.82, 14.10 ppm. **HRMS** (ESI): calcd. for $C_{16}H_{32}NO^+$: 254.2478. found: 254.2476.

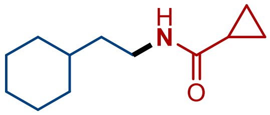

***N*-(2-Cyclohexylethyl)cyclopropanecarboxamide (21):** white solid (70%). **M.P.** 82–83 ºC. **^{1}H NMR** (400 MHz, $CDCl_3$) δ 5.65 (s, 1H), 3.27 (dt, *J* = 7.8, 5.8 Hz, 2H), 1.74 – 1.60 (m, 5H), 1.41 – 1.09 (m, 7H), 0.97 – 0.82 (m, 4H), 0.70 (dq, *J* = 7.2, 3.9 Hz, 2H) ppm. **^{13}C NMR** (101 MHz, $CDCl_3$) δ 173.35, 37.59, 37.24, 35.41, 33.17, 26.51, 26.22, 14.76, 6.92 ppm. **HRMS** (ESI): calcd. for $C_{12}H_{22}NO^+$: 196.1696. found: 196.1699.

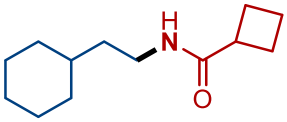

***N*-(2-Cyclohexylethyl)cyclobutanecarboxamide (3):** white solid (88%). **M.P.** 62–63 ºC. **^{1}H NMR** (400 MHz, $CDCl_3$) δ 5.46 (s, 1H), 3.36 – 3.14 (m, 2H), 2.95 (p, *J* = 8.5 Hz, 1H), 2.24 (pd, *J* = 9.1, 2.3 Hz, 2H), 2.16 – 2.04 (m, 2H), 1.98 – 1.77 (m, 2H), 1.71 – 1.58 (m, 5H), 1.45 – 1.31 (m, 2H), 1.31 – 1.08 (m, 4H), 0.88 (qd, *J* = 13.8, 12.6, 3.6 Hz, 2H) ppm. **^{13}C NMR** (101 MHz, $CDCl_3$) δ 174.91, 40.11, 37.36, 37.27, 35.55, 33.27, 27.09 – 24.57 (m), 18.24 ppm. **HRMS** (ESI): calcd. for $C_{13}H_{24}NO^+$: 210.1863. found: 210.1855.

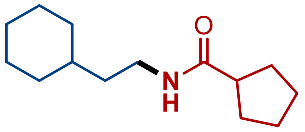

***N*-(2-Cyclohexylethyl)cyclopentanecarboxamide (22):** white solid (78%). **M.P.** 85–87 ºC. **^{1}H NMR** (400 MHz, $CDCl_3$) δ 5.51 (s, 1H), 3.29 – 3.19 (m, 2H), 2.47 (p, *J* = 7.9 Hz, 1H), 1.86 – 1.48 (m, 13H), 1.37 (dt, *J* = 9.0, 6.9 Hz, 2H), 1.32 – 1.08 (m, 4H), 0.89 (qd, *J* = 13.8, 12.6, 3.6 Hz, 2H) ppm. **^{13}C NMR** (101 MHz, $CDCl_3$) δ 176.08, 45.97, 37.34, 37.19, 35.47, 33.17, 30.45, 26.50, 26.22, 25.90 ppm. **HRMS** (ESI): calcd. for $C_{14}H_{26}NO^+$: 224.2009. found: 224.2013.

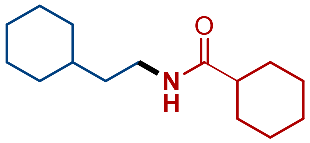

***N*-(2-Cyclohexylethyl)cyclohexanecarboxamide (23):** white solid (82%). **M.P.** 111–112 ºC. **^{1}H NMR** (400 MHz, $CDCl_3$) δ 5.50 (d, *J* = 5.8 Hz, 1H), 3.30 – 3.18 (m, 2H), 2.03 (tt, *J* = 11.8, 3.4 Hz, 1H), 1.87 – 1.73 (m, 4H), 1.65 (tdd, *J* = 15.8, 7.6, 3.6 Hz, 6H), 1.47 – 1.32 (m, 4H), 1.32 – 1.09 (m, 7H), 0.89 (qd, *J* = 13.9, 12.6, 3.5 Hz, 2H) ppm. **^{13}C NMR** (101 MHz, $CDCl_3$) δ 175.97, 45.62, 37.15, 35.45, 33.17, 29.74, 26.49, 26.22, 25.77 ppm. **HRMS** (ESI): calcd. for $C_{15}H_{28}NO^+$: 238.2165. found: 238.2169.

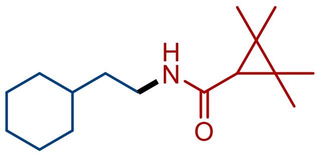

***N*-(2-Cyclohexylethyl)-2,2,3,3-tetramethylcyclopropane-1-carboxamide (24):** colorless oil (86%). **^{1}H NMR** (400 MHz, $CDCl_3$) δ 5.44 (s, 1H), 3.27 – 3.18 (m, 2H), 1.73 – 1.57 (m, 5H), 1.40 – 1.31 (m, 2H), 1.23 (s, 8H), 1.19 – 1.14 (m, 2H), 1.12 (s, 6H), 0.95 – 0.82 (m, 2H), 0.80 (s, 1H) ppm. **^{13}C NMR** (101 MHz, $CDCl_3$) δ 171.51, 37.69, 37.37, 37.17, 35.50, 33.18, 27.71, 26.52, 26.24, 23.69, 16.90 ppm. **HRMS** (ESI): calcd. for $C_{16}H_{30}NO^+$: 252.2322. found: 252.2326.

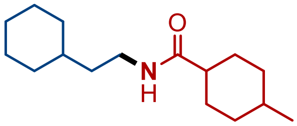

***N*-(2-Cyclohexylethyl)-4-methylcyclohexane-1-carboxamide (25):** white solid (75%). **M.P.** 136–137 ºC. **^{1}H NMR** (400 MHz, $CDCl_3$) δ 5.48 (t, *J* = 5.6 Hz, 1H), 3.29 – 3.17 (m, 2H), 2.01 – 1.91 (m, 1H), 1.84 (dd, *J* = 13.8, 3.5 Hz, 2H), 1.70 (dddd, *J* = 23.0, 11.0, 6.4, 3.1 Hz, 7H), 1.51 – 1.31 (m, 5H), 1.27 – 1.07 (m, 4H), 0.94 – 0.81 (m, 7H) ppm. **^{13}C NMR** (101 MHz, $CDCl_3$) δ 176.05, 45.45, 37.16, 35.45, 34.49, 33.17, 32.03, 29.71, 26.50, 26.22, 22.53 ppm. **HRMS** (ESI): calcd. for $C_{16}H_{30}NO^+$: 252.2327. found: 252.2325.

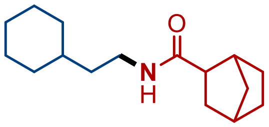

***N*-(2-Cyclohexylethyl)bicyclo[2.2.1]heptane-2-carboxamide (26):** white solid (87%). **M.P.** 141–142 ºC. **^{1}H NMR** (400 MHz, $CDCl_3$) δ 5.41 (s, 1H), 3.32 – 3.15 (m, 2H), 2.40 – 2.22 (m, 2H), 2.06 (dd, *J* = 9.0, 5.3 Hz, 1H), 1.83 (dt, *J* = 11.9, 4.1 Hz, 2H), 1.68 (dq, *J* = 12.9, 4.0 Hz, 5H), 1.60 – 1.47 (m, 3H), 1.37 (dt,

J = 14.6, 7.9 Hz, 3H), 1.26 – 1.08 (m, 7H), 0.95 – 0.84 (m, 2H) ppm. **^{13}C NMR** (101 MHz, $CDCl_3$) δ 175.64, 48.13, 41.53, 37.35, 37.23, 36.51, 35.93, 35.46, 34.41, 33.19, 33.16, 29.84, 28.66, 26.51, 26.23 ppm. **HRMS** (ESI): calcd. for $C_{16}H_{28}NO^+$: 250.2171. found: 250.2169.

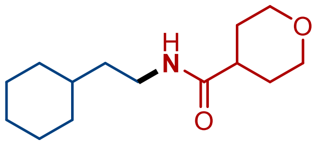

***N*-(2-Cyclohexylethyl)tetrahydro-2H-pyran-4-carboxamide (27):** white solid (87%). **M.P.** 104–105 ºC. **^{1}H NMR** (400 MHz, $CDCl_3$) δ 5.46 (s, 0H), 4.06 – 3.94 (m, 1H), 3.39 (td, J = 11.3, 3.2 Hz, 1H), 3.30 – 3.20 (m, 1H), 2.41 – 2.23 (m, 0H), 1.85 – 1.60 (m, 4H), 1.38 (dt, J = 9.0, 7.0 Hz, 1H), 1.29 – 1.10 (m, 1H), 0.91 (qd, J = 10.7, 10.0, 5.0 Hz, 1H) ppm. **^{13}C NMR** (101 MHz, $CDCl_3$) δ 174.20, 67.44, 42.46, 37.44, 37.24, 35.59, 33.29, 29.48, 26.60, 26.33 ppm. **HRMS** (ESI): calcd. for $C_{14}H_{26}NO_2^+$: 240.1969. found: 240.1962.

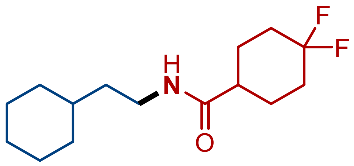

***N*-(2-Cyclohexylethyl)-4,4-difluorocyclohexane-1-carboxamide (28):** white solid (85%). **M.P.** 121–122 ºC. **^{1}H NMR** (400 MHz, $CDCl_3$) δ 5.58 (s, 1H), 3.28 – 3.21 (m, 2H), 2.14 (ddq, J = 15.3, 7.5, 3.7 Hz, 3H), 1.93 – 1.74 (m, 5H), 1.67 (tq, J = 12.4, 8.1, 6.4 Hz, 6H), 1.37 (q, J = 7.1 Hz, 2H), 1.30 – 1.09 (m, 4H), 0.90 (qd, J = 10.8, 9.9, 4.7 Hz, 2H) ppm. **^{13}C NMR** (101 MHz, $CDCl_3$) δ 173.91 (d, J = 2.1 Hz), 131.22 – 117.17 (m), 42.90, 37.21 (d, J = 24.8 Hz), 35.45, 33.19 – 32.86 (m), 32.74 (d, J = 23.5 Hz), 26.45, 26.19, 26.00, 25.91 ppm. **^{19}F NMR** (376 MHz, $CDCl_3$) δ -92.84 (d, J = 236.7 Hz), -100.82 (d, J = 236.5 Hz). **HRMS** (ESI): calcd. for $C_{15}H_{26}F_2NO^+$: 274.1988. found: 274.1980.

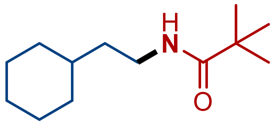

***N*-(2-Cyclohexylethyl)pivalamide (29):** white solid (25%). **M.P.** 59–60 ºC. **^{1}H NMR** (400 MHz, $CDCl_3$) δ 5.55 (s, 1H), 3.28 – 3.21 (m, 2H), 1.75 – 1.64 (m, 5H), 1.42 – 1.35 (m, 2H), 1.32 – 1.18 (m, 4H), 1.18 (s, 9H), 0.97 – 0.85 (m, 2H) ppm. **^{13}C NMR** (101 MHz, $CDCl_3$) δ 178.24, 38.59, 37.45, 37.12, 35.58, 33.19, 27.61, 26.50, 26.23 ppm. **HRMS** (ESI): calcd. for $C_{13}H_{26}NO^+$: 212.202. found: 212.2013.

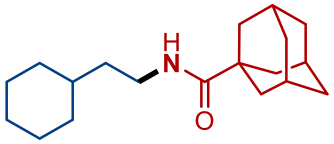

***N*-(2-Cyclohexylethyl)cyclobutanecarboxamide (30):** white solid (23%). **M.P.** 134–135 ºC. **^{1}H NMR** (400 MHz, $CDCl_3$) δ 5.51 (s, 1H), 3.49 – 3.03 (m, 3H), 2.03 (s, 1H), 1.83 (d, J = 2.9 Hz, 8H), 1.70 (qd, J = 10.1, 9.3, 6.1 Hz, 16H), 1.37 (dt, J = 8.9, 6.9 Hz, 3H), 1.31 – 1.09 (m, 5H), 0.90 (qd, J = 13.8, 12.6, 3.6 Hz, 3H) ppm. **^{13}C NMR** (101 MHz, $CDCl_3$) δ 177.90, 40.67, 39.46, 37.31, 37.29, 36.70, 35.69, 33.33, 28.31, 26.64, 26.37 ppm. **HRMS** (ESI): calcd. for $C_{19}H_{32}NO^+$: 290.2489. found: 290.2482.

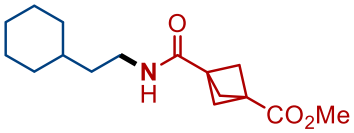

Methyl 3-((2-cyclohexylethyl)carbamoyl)bicyclo[1.1.1]pentane-1-carboxylate (31): white solid (92%). **M.P.** 104–105 ºC. **^{1}H NMR** (400 MHz, $CDCl_3$) δ 5.64 (t, *J* = 5.8 Hz, 1H), 3.66 (s, 3H), 3.22 (ddd, *J* = 9.3, 7.6, 5.8 Hz, 2H), 2.23 (s, 6H), 1.72 – 1.53 (m, 5H), 1.42 – 1.29 (m, 2H), 1.26 – 1.08 (m, 4H), 0.88 (qd, *J* = 10.8, 9.9, 4.9 Hz, 2H). **^{13}C NMR** (101 MHz, $CDCl_3$) δ 169.88, 168.95, 52.20, 51.80, 39.35, 37.36, 37.00, 36.68, 35.49, 33.13, 26.44, 26.15. **HRMS** (ESI): calcd. for $C_{16}H_{26}NO_3^+$: 280.1913. found: 280.1908.

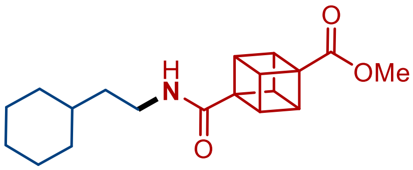

Methyl-4-((2-cyclohexylethyl)carbamoyl)cubane-1-carboxylate (32): white solid (70%). **M.P.** 163–164 ºC. **^{1}H NMR** (400 MHz, $CDCl_3$) δ 5.67 (t, *J* = 5.8 Hz, 1H), 4.20 – 4.09 (m, 6H), 3.66 (s, 3H), 3.24 (ddd, *J* = 9.3, 7.6, 5.8 Hz, 2H), 1.70 – 1.59 (m, 5H), 1.40 – 1.33 (m, 2H), 1.28 – 1.04 (m, 5H), 0.87 (qd, *J* = 10.3, 9.4, 4.8 Hz, 2H) ppm. **^{13}C NMR** (101 MHz, $CDCl_3$) δ 172.03, 171.07, 57.83, 55.77, 51.56, 46.93, 46.60, 37.19, 37.14, 35.47, 33.14, 26.45, 26.15 ppm. **HRMS** (ESI): calcd. for $C_{19}H_{26}NO_3^+$: 316.1907. found: 316.1909.

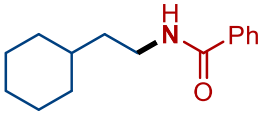

***N*-(2-Cyclohexylethyl)benzamide (33):** white solid (75%). **M.P.** 81–82 ºC. **^{1}H NMR** (400 MHz, $CDCl_3$) δ 7.76 – 7.72 (m, 1H), 7.49 – 7.44 (m, 1H), 7.39 (dd, *J* = 8.2, 6.6 Hz, 1H), 6.32 (s, 0H), 3.53 – 3.34 (m, 1H), 1.80 – 1.60 (m, 3H), 1.49 (dt, *J* = 8.9, 7.0 Hz, 1H), 1.35 – 1.11 (m, 2H), 0.93 (qd, *J* = 13.9, 12.9, 3.9 Hz, 1H) ppm. **^{13}C NMR** (101 MHz, $CDCl_3$) δ 167.61, 134.99, 131.32, 128.58, 126.96, 38.05, 37.21, 35.60, 33.28, 26.59, 26.31 ppm. **HRMS** (ESI): calcd. for $C_{15}H_{22}NO^+$: 232.1707. found: 232.1699.

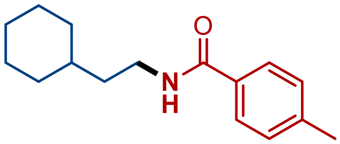

***N*-(2-Cyclohexylethyl)-4-methylbenzamide (34):** white solid (82%). **M.P.** 101–102 ºC. **^{1}H NMR** (400 MHz, $CDCl_3$) δ 7.65 (d, *J* = 8.2 Hz, 2H), 7.20 (d, *J* = 7.9 Hz, 2H), 6.16 (s, 1H), 3.45 (ddd, *J* = 8.8, 7.5, 5.8 Hz, 2H), 2.38 (s, 3H), 1.76 – 1.62 (m, 5H), 1.53 – 1.45 (m, 2H), 1.33 (ddt, *J* = 10.9, 7.3, 3.8 Hz, 1H), 1.25 – 1.11 (m, 3H), 0.98 – 0.88 (m, 2H) ppm. **^{13}C NMR** (101 MHz, $CDCl_3$) δ 167.41, 141.60, 132.05, 129.15, 126.84, 37.88, 37.16, 35.52, 33.20, 26.51, 26.23, 21.40 ppm. **HRMS** (ESI): calcd. for $C_{16}H_{24}NO^+$: 246.1852. found: 246.1857.

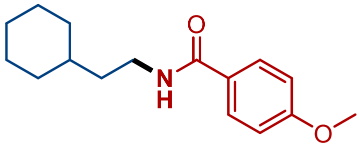

***N*-(2-Cyclohexylethyl)-4-methoxybenzamide (35):** white solid (75%). **M.P.** 93–94 ºC. **^{1}H NMR** (400 MHz, $CDCl_3$) δ 7.76 – 7.67 (m, 2H), 6.91 – 6.85 (m, 2H), 3.82 (s, 3H), 3.42 (ddd, *J* = 8.9, 7.5, 5.8 Hz, 2H), 1.75 – 1.60 (m, 5H), 1.47 (dt, *J* = 8.9, 6.9 Hz, 2H), 1.32

(dqt, *J* = 10.7, 7.2, 3.4 Hz, 1H), 1.24 – 1.07 (m, 3H), 0.97 – 0.84 (m, 2H) ppm. **^{13}C NMR** (101 MHz, $CDCl_3$) δ 167.01, 161.99, 128.64, 127.19, 113.66, 55.37, 37.89, 37.19, 35.51, 33.20, 26.51, 26.22 ppm. **HRMS** (ESI): calcd. for $C_{16}H_{24}NO_2^+$: 262.1802. found: 262.1803.

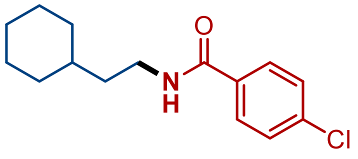

4-Chloro-*N*-(2-cyclohexylethyl)benzamide (36): white solid (80%). **M.P.** 112–113 ºC. **^{1}H NMR** (400 MHz, $CDCl_3$) δ 7.71 – 7.66 (m, 2H), 7.39 – 7.34 (m, 2H), 6.27 (t, *J* = 5.7 Hz, 1H), 3.48 – 3.38 (m, 2H), 1.76 – 1.61 (m, 5H), 1.48 (dt, *J* = 9.0, 7.0 Hz, 2H), 1.32 (dtd, *J* = 10.9, 7.4, 3.5 Hz, 1H), 1.27 – 1.10 (m, 3H), 0.99 – 0.86 (m, 2H) ppm. **^{13}C NMR** (101 MHz, $CDCl_3$) δ 166.44, 137.48, 133.24, 128.73, 128.32, 38.07, 37.07, 35.51, 33.18, 26.48, 26.20 ppm. **HRMS** (ESI): calcd. for $C_{15}H_{21}ClNO^+$: 266.1306. found: 266.1308.

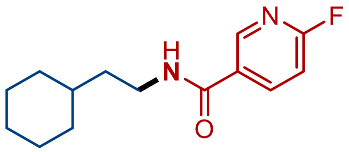

***N*-(2-Cyclohexylethyl)-6-fluoronicotinamide (37):** white solid (76%). **M.P.** 81–82 ºC. **^{1}H NMR** (400 MHz, $CDCl_3$) δ 8.58 (d, *J* = 2.5 Hz, 1H), 8.22 (td, *J* = 8.0, 2.5 Hz, 1H), 6.96 (dd, *J* = 8.5, 2.9 Hz, 1H), 6.49 (t, *J* = 5.7 Hz, 1H), 3.48 – 3.41 (m, 2H), 1.73 – 1.61 (m, 5H), 1.49 (dt, *J* = 8.9, 6.9 Hz, 2H), 1.31 (ddq, *J* = 10.8, 7.2, 3.7 Hz, 1H), 1.24 – 1.10 (m, 3H), 0.93 (td, *J* = 11.5, 2.9 Hz, 2H) ppm. **^{13}C NMR** (101 MHz, $CDCl_3$) δ 164.88 (d, *J* = 244.0 Hz), 164.50, 146.56 (d, *J* = 15.9 Hz), 140.81 (d, *J* = 8.8 Hz), 128.88 (d, *J* = 4.6 Hz), 109.63 (d, *J* = 37.3 Hz), 38.17, 36.97, 35.47, 33.14, 26.43, 26.16 ppm. ^{19}F NMR (376 MHz, $CDCl_3$) δ -63.86 ppm. **HRMS** (ESI): calcd. for $C_{14}H_{20}FN_2O^+$: 251.1554. found: 251.1557.

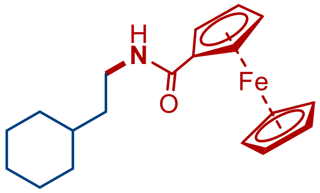

(2-((2-Cyclohexylethyl)carbamoyl)cyclopenta-2,4-dien-1-yl)(cyclopenta-2,4-dien-1-yl)iron (38): yellow solid (47%). **M.P.** 155–157 ºC. **^{1}H NMR** (400 MHz, $CDCl_3$) δ 45.61 (s, 1H), 4.65 (s, 1H), 4.32 (s, 1H), 3.40 (q, *J* = 6.7 Hz, 2H), 1.84 – 1.69 (m, 3H), 1.62 (s, 1H), 1.48 (q, *J* = 7.1 Hz, 2H), 1.35 (ddt, *J* = 10.8, 7.3, 3.6 Hz, 0H), 1.28 – 1.14 (m, 3H), 0.97 (qd, *J* = 11.6, 10.2, 2.5 Hz, 2H) ppm. **^{13}C NMR** (101 MHz, $CDCl_3$) δ 169.95, 70.25, 69.70, 68.01, 37.45, 37.36, 35.47, 33.25, 26.53, 26.28 ppm. **HRMS** (ESI): calcd. for $C_{19}H_{26}FeNO^+$: 340.1358. found: 340.1356.

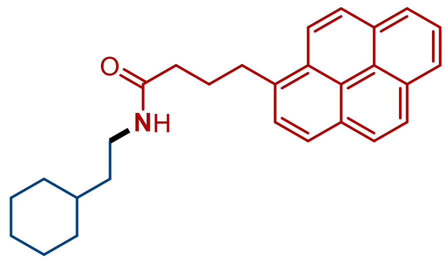

***N*-(2-Cyclohexylethyl)-4-(pyren-1-yl)butanamide (39):** white solid (91%). **M.P.** 144–145 ºC. **^{1}H NMR** (400 MHz, $CDCl_3$) δ 8.28 (d, *J* = 9.3 Hz, 1H), 8.18 – 8.12 (m, 2H), 8.09 (d, *J* = 8.6 Hz, 2H), 8.00 (d, *J* = 12.5 Hz, 3H), 7.84 (d, *J* = 7.8 Hz, 1H), 5.35 (s, 1H), 3.36 (t, *J* = 6.9 Hz, 2H), 3.27 – 3.19 (m, 2H), 2.25 – 2.15 (m, 4H), 1.72 – 1.60 (m, 5H), 1.31 (q, *J* = 6.9 Hz, 2H), 1.26 – 1.08 (m, 4H), 0.87 (q, *J* = 10.7, 10.1 Hz, 2H) ppm. **^{13}C NMR** (101 MHz, $CDCl_3$) δ 172.45, 135.93, 131.43, 130.92, 129.94, 127.48, 127.37, 126.71, 125.86, 125.00, 124.91, 124.78, 123.42, 37.36, 37.10, 36.06, 35.39, 33.14, 32.73, 27.45, 26.50, 26.21 ppm. **HRMS** (ESI): calcd. for $C_{28}H_{32}NO^{+}$: 398.2489. found: 398.2481.

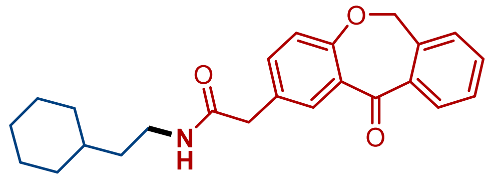

***N*-(2-Cyclohexylethyl)-2-(11-oxo-6,11-dihydrodibenzo[b,e]oxepin-2-yl)acetamide (40):** white solid (63%). **M.P.** 151–152 ºC. **^{1}H NMR** (400 MHz, $CDCl_3$) δ 8.08 (d, *J* = 2.4 Hz, 1H), 7.88 (dd, *J* = 7.7, 1.4 Hz, 1H), 7.56 (td, *J* = 7.5, 1.4 Hz, 1H), 7.50 – 7.41 (m, 2H), 7.36 (dd, *J* = 7.4, 1.3 Hz, 1H), 7.04 (d, *J* = 8.4 Hz, 1H), 5.47 (s, 1H), 5.18 (s, 2H), 3.54 (s, 2H), 3.27 – 3.18 (m, 2H), 1.67 – 1.59 (m, 5H), 1.32 (dt, *J* = 8.9, 6.9 Hz, 2H), 1.22 – 1.07 (m, 4H), 0.91 – 0.80 (m, 2H) ppm. **^{13}C NMR** (101 MHz, $CDCl_3$) δ 190.84, 170.45, 160.55, 140.39, 136.41, 135.52, 132.88, 132.32, 129.47, 129.31, 128.99, 127.87, 125.28, 121.49, 73.64, 42.80, 37.66, 36.89, 35.41, 33.11, 26.45, 26.18 ppm. **HRMS** (ESI): calcd. for $C_{24}H_{27}NaNO_3^{+}$: 400.1883. found: 400.1887.

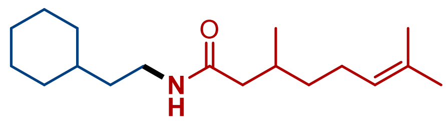

***N*-(2-Cyclohexylethyl)-3,7-dimethyloct-6-enamide (41):** white solid (78%). **M.P.** 54–55 ºC. **^{1}H NMR** (400 MHz, $CDCl_3$) δ 5.57 (t, *J* = 5.8 Hz, 1H), 5.06 (tt, *J* = 7.0, 1.5 Hz, 1H), 3.24 (tdd, *J* = 7.2, 5.6, 1.6 Hz, 2H), 2.18 – 2.11 (m, 1H), 2.03 – 1.85 (m, 5H), 1.71 – 1.58 (m, 9H), 1.36 (q, *J* = 7.2 Hz, 3H), 1.29 – 1.08 (m, 6H), 0.94 – 0.86 (m, 5H) ppm. **^{13}C NMR** (101 MHz, $CDCl_3$) δ 172.41, 131.39, 124.40, 44.65, 37.24, 37.14, 36.92, 35.38, 33.15, 30.49, 26.49, 26.21, 25.67, 25.48, 19.50, 17.63 ppm. **HRMS** (ESI): calcd. for $C_{18}H_{34}NO^{+:}$ 280.2635. found: 280.2637.

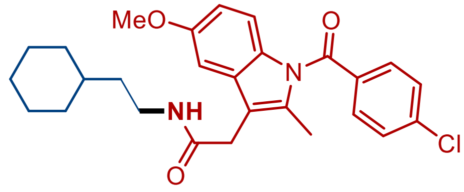

2-(1-(4-Chlorobenzoyl)-5-methoxy-2-methyl-1H-indol-3-yl)-*N*-(2-cyclohexylethyl)acetamide (42): pale yellow solid (40%). **M.P.** 145–146 ºC. **^{1}H NMR** (400 MHz, $CDCl_3$) δ 7.67 – 7.54 (m, 2H), 7.51 – 7.36 (m, 2H), 6.92 – 6.81 (m, 2H), 6.68 (dd, *J* = 9.0, 2.5 Hz, 1H), 5.64 (t, *J* = 5.8 Hz, 1H), 3.80 (s, 3H), 3.61 (s, 2H), 3.20 (dt, *J*

= 7.8, 6.0 Hz, 2H), 2.36 (s, 3H), 1.65 – 1.54 (m, 5H), 1.31 – 1.18 (m, 7H), 1.09 (t, *J* = 9.8 Hz, 4H), 0.89 – 0.73 (m, 2H) ppm. **^{13}C NMR** (101 MHz, $CDCl_3$) δ 169.65, 156.27, 139.53, 136.25, 133.58, 131.14, 130.87, 130.32, 129.18, 115.07, 112.99, 112.27, 100.85, 55.70, 37.48, 36.86, 35.31, 33.04, 32.23, 26.39, 26.11, 24.83, 13.23 ppm. **HRMS** (ESI): calcd. for $C_{27}H_{32}ClN_2O_3^+$: 467.2096. found: 467.2099.

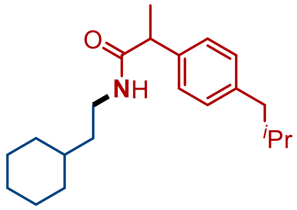

***N*-(2-*N*-(2-Cyclohexylethyl)-2-(4-isobutylphenyl)propanamide (43):** white solid (25%). **M.P.** 108–110 ºC. **^{1}H NMR** (400 MHz, $CDCl_3$) δ 7.18 (d, *J* = 8.1 Hz, 2H), 7.11 (d, *J* = 8.1 Hz, 2H), 5.24 (s, 1H), 3.50 (q, *J* = 7.2 Hz, 1H), 3.20 (tdd, *J* = 7.4, 5.7, 1.9 Hz, 2H), 2.45 (d, *J* = 7.2 Hz, 2H), 1.85 (dt, *J* = 13.5, 6.8 Hz, 1H), 1.69 – 1.59 (m, 5H), 1.50 (d, *J* = 7.2 Hz, 3H), 1.27 (q, *J* = 7.2 Hz, 2H), 1.11 (dd, *J* = 11.1, 3.3 Hz, 4H), 0.90 (d, *J* = 6.6 Hz, 8H) ppm. **^{13}C NMR** (101 MHz, $CDCl_3$) δ 174.26, 140.66, 138.69, 129.58, 127.36, 46.80, 45.00, 37.40, 36.82, 35.29, 33.09, 33.04, 30.16, 26.44, 26.17, 22.35, 18.38 ppm. **HRMS** (ESI): calcd. for $C_{21}H_{34}NO^+$: 316.2635. found: 316.2639.

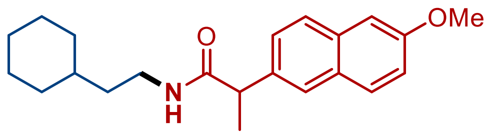

***N*-(2-Cyclohexylethyl)-2-(6-methoxynaphthalen-2-yl)propanamide (44):** white solid (36%). **M.P.** 114–116 ºC. **^{1}H NMR** (400 MHz, $CDCl_3$) δ 7.72 (dd, *J* = 8.7, 4.7 Hz, 2H), 7.68 – 7.63 (m, 1H), 7.37 (dd, *J* = 8.5, 1.9 Hz, 1H), 7.18 – 7.11 (m, 2H), 5.28 (s, 1H), 3.92 (s, 3H), 3.67 (q, *J* = 7.2 Hz, 1H), 3.19 (td, *J* = 7.5, 5.8 Hz, 2H), 1.59 (d, *J* = 7.2 Hz, 8H), 1.25 (dt, *J* = 8.7, 6.9 Hz, 2H), 1.17 – 1.04 (m, 4H), 0.81 (tt, *J* = 14.5, 7.7 Hz, 2H) ppm. **^{13}C NMR** (101 MHz, $CDCl_3$) δ 171.51, 37.69, 37.37, 37.17, 35.50, 33.18, 27.71, 26.52, 26.24, 23.69, 16.90 ppm. **HRMS** (ESI): calcd. for $C_{22}H_{29}NaNO_2^+$: 362.2091. found: 362.2094.

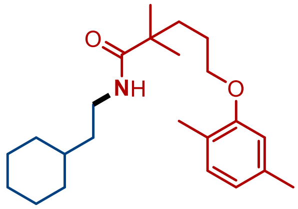

***N*-(2-Cyclohexylethyl)-5-(2,5-dimethylphenoxy)-2,2-dimethylpentanamide (45):** colorless oil (8%). **^{1}H NMR** (400 MHz, $CDCl_3$) δ 7.00 (d, *J* = 7.4 Hz, 1H), 6.69 – 6.54 (m, 2H), 5.65 (t, *J* = 5.7 Hz, 1H), 3.91 (t, *J* = 5.8 Hz, 2H), 3.33 – 3.24 (m, 2H), 2.30 (s, 3H), 2.18 (s, 3H), 1.78 – 1.63 (m, 9H), 1.39 (dt, *J* = 8.7, 6.9 Hz, 2H), 1.21 (s, 10H), 0.91 (qd, *J* = 13.7, 12.6, 3.6 Hz, 2H) ppm. **^{13}C NMR** (101 MHz, $CDCl_3$) δ 177.20, 156.92, 136.48, 130.27, 123.45, 120.72, 112.04, 77.38, 77.07, 76.75, 67.97, 41.78, 37.58, 37.44, 37.14, 35.54, 33.16, 26.49, 26.23, 25.57, 25.13, 21.39, 15.80 ppm. **HRMS** (ESI): calcd. for $C_{23}H_{38}NO_2^+$: 360.2897. found: 360.2901.

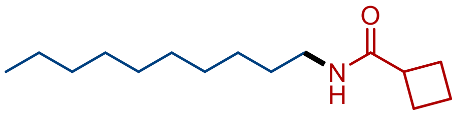

***N*-Decylcyclobutanecarboxamide (46):** white solid (65%). **M.P.** 49–50ºC. **^{1}H NMR** (400 MHz, $CDCl_3$) δ 5.42 (s, 0H), 3.21 (td, *J* = 7.3, 5.8 Hz, 1H), 2.96 (p, *J* = 8.6 Hz, 0H), 2.25 (pd, *J* = 9.1, 2.3 Hz, 1H), 2.12 (dtt, *J* = 11.8, 8.5, 2.4 Hz, 1H), 1.96 – 1.82 (m, 1H), 1.46 (t, *J* = 7.2 Hz, 1H), 1.25 (q, *J* = 5.5 Hz, 5H), 0.86 (t, *J* = 6.7 Hz, 1H) ppm. **^{13}C NMR** (101 MHz, $CDCl_3$) δ 174.86, 40.02, 39.44, 31.87, 29.70, 29.52, 29.29, 26.91, 25.38, 24.78, 22.66, 18.13, 14.08 ppm. **HRMS** (ESI): calcd. for $C_{15}H_{30}NO^+$: 240.2333. found: 240.2327.

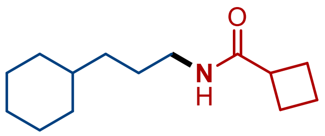

***N*-(3-Cyclohexylpropyl)cyclobutanecarboxamide (47):** white solid (70%). **M.P.** 53–54 ºC. **^{1}H NMR** (400 MHz, $CDCl_3$) δ 5.38 (s, 1H), 3.23 – 3.16 (m, 2H), 2.96 (p, *J* = 8.6 Hz, 1H), 2.25 (pd, *J* = 9.1, 2.4 Hz, 2H), 2.13 (ddt, *J* = 12.4, 9.0, 4.5 Hz, 2H), 1.97 – 1.80 (m, 2H), 1.71 – 1.61 (m, 5H), 1.48 (p, *J* = 7.5 Hz, 2H), 1.24 – 1.11 (m, 6H), 0.90 – 0.80 (m, 2H) ppm. **^{13}C NMR** (101 MHz, $CDCl_3$) δ 174.97, 40.16, 39.86, 37.50, 34.72, 33.44, 27.18, 26.75, 26.45, 25.52, 18.26 ppm. **HRMS** (ESI): calcd. for $C_{14}H_{26}NO^+$: 246.1828. found: 246.1833.

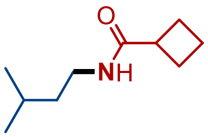

***N*-Isopentylcyclobutanecarboxamide (48):** white solid (88%). **M.P.** 84–85 ºC.**^{1}H NMR** (400 MHz, $CDCl_3$) δ 5.54 (s, 1H), 3.29 – 3.17 (m, 2H), 3.00 – 2.88 (m, 1H), 2.28 – 2.16 (m, 2H), 2.14 – 2.04 (m, 2H), 1.97 – 1.76 (m, 2H), 1.57 (dt, *J* = 13.4, 6.7 Hz, 1H), 1.39 – 1.28 (m, 2H), 0.87 (d, *J* = 6.6 Hz, 6H) ppm. **^{13}C NMR** (101 MHz, $CDCl_3$) δ 174.88, 39.97, 38.58, 37.70, 25.87, 25.38, 25.34, 24.80, 22.43, 18.14, 18.12 ppm. **HRMS** (ESI): calcd. for $C_{10}H_{20}NO^+$: 170.1539. found: 170.1543.

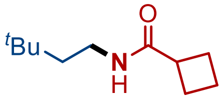

***N*-(3,3-Dimethylbutyl)cyclobutanecarboxamide (49):** white solid (33%). **M.P.** 66–67 ºC. **^{1}H NMR** (400 MHz, $CDCl_3$) δ 5.28 (s, 1H), 3.31 – 3.17 (m, 2H), 2.95 (pd, *J* = 8.5, 1.0 Hz, 1H), 2.32 – 2.17 (m, 2H), 2.17 – 2.05 (m, 2H), 1.97 – 1.79 (m, 2H), 1.46 – 1.34 (m, 2H), 0.92 (s, 10H) ppm. **^{13}C NMR** (101 MHz, $CDCl_3$) δ 174.75, 43.44, 40.02, 36.07, 29.91, 29.38, 25.33, 18.10 ppm. **HRMS** (ESI): calcd. for $C_{11}H_{22}NO^+$: 184.1696. found: 184.1698.

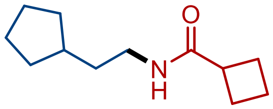

***N*-(2-Cyclopentylethyl)cyclobutanecarboxamide (50):** white solid (80%). **M.P.** 62–63 ºC. **^{1}H NMR** (400 MHz, $CDCl_3$) δ 5.44 (s, 1H), 3.23 (dt, *J* = 7.8, 5.8 Hz, 2H), 2.95 (p, *J* = 8.5 Hz, 1H), 2.24 (pd, *J* = 9.1, 2.4 Hz, 2H), 2.11 (qt, *J* = 8.6, 3.0 Hz, 2H), 1.99 – 1.90 (m, 1H), 1.89 – 1.81 (m, 1H), 1.79 – 1.70 (m, 3H), 1.62 – 1.53 (m, 2H), 1.49 (ddd, *J* = 12.4, 7.2, 4.1 Hz, 4H), 1.15 – 1.01 (m, 2H) ppm. **^{13}C NMR** (101

MHz, $CDCl_3$) δ 174.81, 40.02, 38.89, 37.79, 35.99, 32.61, 25.37, 25.08, 18.13 ppm. **HRMS** (ESI): calcd. for $C_{12}H_{22}NO^+$: 196.1696. found: 196.1699.

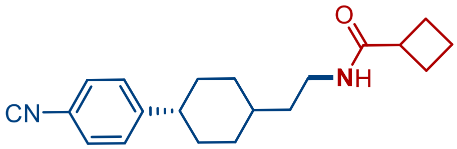

***N*-(2-(4-(4-Isocyanophenyl)-cyclohexyl)ethyl)-cyclobutanecarboxamide (51):** white solid (83%). **M.P.** 151–152 ºC. **^{1}H NMR** (400 MHz, $CDCl_3$) δ 7.59 – 7.49 (m, 2H), 7.31 – 7.21 (m, 2H), 5.49 (t, *J* = 6.0 Hz, 1H), 3.32 – 3.22 (m, 2H), 2.96 (p, *J* = 8.6 Hz, 1H), 2.49 (tt, *J* = 12.1, 3.2 Hz, 1H), 2.24 (pd, *J* = 9.1, 2.4 Hz, 2H), 2.11 (dddd, *J* = 11.9, 8.5, 6.1, 2.7 Hz, 2H), 1.88 (dtd, *J* = 17.2, 9.7, 8.5, 2.9 Hz, 6H), 1.48 – 1.28 (m, 5H), 1.08 (qd, *J* = 13.7, 13.2, 3.6 Hz, 2H) ppm. **^{13}C NMR** (101 MHz, $CDCl_3$) δ 174.89, 152.97, 132.16, 127.64, 119.12, 109.61, 44.52, 39.97, 37.20, 37.03, 34.93, 33.62, 33.00, 25.36, 18.14 ppm. **HRMS** (ESI): calcd. for $C_{20}H_{27}N_2O^+$: 311.2118. found: 311.2119.

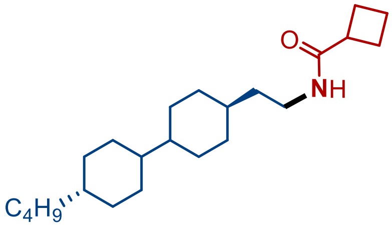

***N*-(2-(4'-Butyl-[1,1'-bi(cyclohexan)]-4-yl)-ethyl)-cyclobutanecarboxamide (52):** white solid (75%). **M.P.** 164–166 ºC. **^{1}H NMR** (400 MHz, $CDCl_3$) δ 5.34 (t, *J* = 5.3 Hz, 1H), 3.24 (ddd, *J* = 8.9, 7.6, 5.8 Hz, 2H), 2.95 (p, *J* = 8.5 Hz, 1H), 2.25 (pd, *J* = 9.1, 2.3 Hz, 2H), 2.17 – 2.07 (m, 2H), 1.97 – 1.88 (m, 1H), 1.82 (s, 1H), 1.76 – 1.64 (m, 8H), 1.36 (dt, *J* = 8.9, 6.8 Hz, 2H), 1.24 (tq, *J* = 8.3, 5.4, 4.3 Hz, 5H), 1.17 – 1.09 (m, 3H), 1.01 – 0.80 (m, 14H) ppm. **^{13}C NMR** (101 MHz, $CDCl_3$) δ 174.76, 43.40, 43.28, 40.01, 37.89, 37.36, 37.18, 35.74, 33.63, 33.41, 30.07, 29.89, 29.23, 25.36, 23.00, 18.11, 14.11 ppm. **HRMS** (ESI): calcd. for $C_{23}H_{42}NO^+$: 348.3261. found: 348.3263.

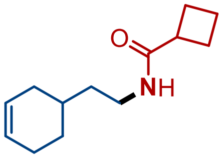

***N*-(2-(Cyclohex-3-en-1-yl)ethyl)cyclobutanecarboxamide (53):** white solid (47%). **M.P.** 49–51 ºC. **^{1}H NMR** (400 MHz, $CDCl_3$) δ 5.69 – 5.57 (m, 2H), 5.37 (s, 1H), 3.29 (td, *J* = 7.5, 5.7 Hz, 2H), 2.96 (p, *J* = 8.6 Hz, 1H), 2.25 (pd, *J* = 9.1, 2.4 Hz, 2H), 2.11 (dddd, *J* = 16.0, 10.0, 5.1, 3.4 Hz, 3H), 2.02 (tdd, *J* = 10.0, 4.6, 2.9 Hz, 2H), 1.98 – 1.83 (m, 2H), 1.77 – 1.55 (m, 3H), 1.52 – 1.42 (m, 2H), 1.28 – 1.19 (m, 1H) ppm. **^{13}C NMR** (101 MHz, $CDCl_3$) δ 174.83, 127.01, 126.13, 40.00, 37.22, 36.33, 31.59, 31.22, 28.64, 25.36, 24.96, 18.11 ppm. **HRMS** (ESI): calcd. for $C_{13}H_{22}NO^+$: 208.1696. found: 208.1699.

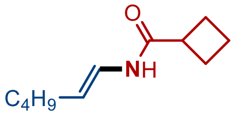

(*E*)-*N*-(Hex-1-en-1-yl)cyclobutanecarboxamide (54): colorless oil (26%). **^{1}H NMR** (400 MHz, $CDCl_3$) δ 6.85 (s, 1H), 6.74 (ddt, *J* = 13.7, 10.5, 1.5 Hz, 1H), 5.11 (dt, *J* = 14.2, 7.2 Hz, 1H), 2.99 (p, *J* = 8.5 Hz, 1H), 2.29 (qd, *J* = 9.1,

2.5 Hz, 2H), 2.14 (ddd, *J* = 12.0, 8.4, 3.7 Hz, 2H), 2.02 – 1.84 (m, 5H), 1.34 – 1.29 (m, 3H), 0.89 (d, *J* = 7.0 Hz, 3H) ppm. **HRMS** (ESI): calcd. for $C_{11}H_{20}NO^+$: 182.1539. found: 182.1533.

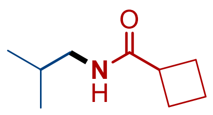

***N*-Isobutyl-2-propylpentanamide (55):** white solid (60%). **M.P.** 41–42 ºC. **^{1}H NMR** (400 MHz, $CDCl_3$) δ 5.50 (s, 1H), 3.05 (t, *J* = 6.4 Hz, 2H), 3.02 – 2.92 (m, 1H), 2.25 (pd, *J* = 9.1, 2.4 Hz, 2H), 2.17 – 2.06 (m, 2H), 1.99 – 1.80 (m, 2H), 1.73 (dq, *J* = 13.5, 6.8 Hz, 1H), 0.88 (d, *J* = 6.7 Hz, 6H) ppm. **^{13}C NMR** (101 MHz, $CDCl_3$) δ 174.91, 46.65, 40.01, 28.54, 25.38, 20.04, 18.13 ppm. **HRMS** (ESI): calcd. for $C_9H_{18}NO^+$: 156.1383. found: 156.1384.

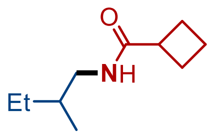

***N*-(2-Methylbutyl)cyclobutanecarboxamide (56):** white solid (61%). **M.P.** 41–42 ºC. **^{1}H NMR** (400 MHz, $CDCl_3$) δ 5.42 (s, 1H), 3.25 – 3.14 (m, 1H), 3.09 – 2.91 (m, 2H), 2.33 – 2.19 (m, 2H), 2.17 – 2.07 (m, 2H), 1.97 – 1.82 (m, 2H), 1.56 – 1.47 (m, 1H), 1.40 – 1.31 (m, 1H), 1.18 – 1.07 (m, 1H), 0.91 – 0.84 (m, 7H) ppm. **^{13}C NMR** (101 MHz, $CDCl_3$) δ 174.93, 44.94, 40.04, 34.96, 26.98, 25.39, 18.13, 17.11, 11.24 ppm. **HRMS** (ESI): calcd. for $C_{10}H_{20}NO^+$: 170.1545. found: 170.1541.

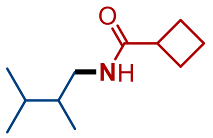

***N*-(2,3-Dimethylbutyl)cyclobutanecarboxamide (57):** white solid (47%). **M.P.** 88-89 ºC. **^{1}H NMR** (400 MHz, $CDCl_3$) δ 5.30 (s, 1H), 3.25 (dt, *J* = 13.4, 5.8 Hz, 1H), 3.11 – 2.93 (m, 2H), 2.31 – 2.20 (m, 2H), 2.18 – 2.09 (m, 2H), 2.02 – 1.82 (m, 2H), 1.63 – 1.44 (m, 2H), 0.91 (d, *J* = 6.8 Hz, 3H), 0.83 (t, *J* = 6.6 Hz, 6H) ppm. **^{13}C NMR** (101 MHz, $CDCl_3$) δ 174.89, 43.34, 40.06, 39.00, 30.05, 25.40, 20.42, 18.13, 17.88, 13.52 ppm. **HRMS** (ESI): calcd. for $C_{11}H_{22}NO^+$: 184.1701. found: 184.1698.

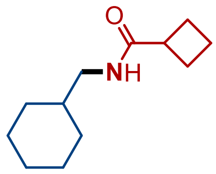

***N*-(Cyclohexylmethyl)cyclobutanecarboxamide (58):** white solid (64%). **M.P.** 85–86 ºC. **^{1}H NMR** (400 MHz, $CDCl_3$) δ 5.46 (s, 1H), 3.07 (t, *J* = 6.4 Hz, 2H), 2.97 (p, *J* = 8.6 Hz, 1H), 2.25 (dq, *J* = 11.6, 9.2 Hz, 2H), 2.12 (dtd, *J* = 12.0, 8.6, 3.2 Hz, 2H), 1.98 – 1.82 (m, 2H), 1.74 – 1.59 (m, 5H), 1.42 (ddt, *J* = 11.2, 7.4, 3.7 Hz, 1H), 1.26 – 1.11 (m, 3H), 0.90 (qd, *J* = 12.5, 11.8, 4.0 Hz, 2H) ppm. **^{13}C NMR** (101 MHz, $CDCl_3$) δ 174.93, 45.57, 40.05, 38.01, 30.83, 26.41, 25.82, 25.43, 18.15 ppm. **HRMS** (ESI): calcd. for $C_{12}H_{22}NO^+$: 196.1696. found: 196.1698.

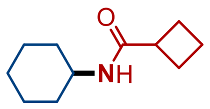

***N*-Cyclohexylcyclobutanecarboxamide (59):** white solid (49%). **M.P.** 112–113 ºC. **^{1}H NMR** (400 MHz, $CDCl_3$) δ 5.24 (s, 1H), 3.73 (dtt, *J* = 11.3, 8.0, 4.0 Hz, 1H), 2.93 (p, *J* = 8.6 Hz, 1H), 2.22 (qd, *J* = 9.1, 2.6 Hz, 2H), 2.10 (dtd, *J* = 12.0, 8.6, 3.2 Hz, 2H), 1.94 – 1.76 (m, 4H), 1.63 (ddt, *J* = 33.7, 12.8, 3.9 Hz, 3H), 1.43 – 1.27 (m,

2H), 1.20 – 1.01 (m, 3H) ppm. **^{13}C NMR** (101 MHz, $CDCl_3$) δ 173.94, 47.89, 40.12, 33.26, 25.56, 25.36, 24.89, 18.08 ppm. **HRMS** (ESI): calcd. for $C_{11}H_{20}NO^+$: 182.1545. found: 182.1544.

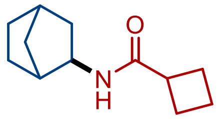

***N*-((2S)-Bicyclo[2.2.1]heptan-2-yl)cyclobutanecarboxamide (60):** white solid (62%). **M.P.** 155–157 ºC. **^{1}H NMR** (400 MHz, $CDCl_3$) δ 5.24 (s, 1H), 3.70 (td, *J* = 7.7, 3.5 Hz, 1H), 2.92 (p, *J* = 8.5 Hz, 1H), 2.28 – 2.06 (m, 6H), 1.93 – 1.74 (m, 3H), 1.45 (dddq, *J* = 23.1, 15.8, 7.3, 4.0 Hz, 2H), 1.25 (ddd, *J* = 11.0, 8.5, 2.3 Hz, 2H), 1.19 – 1.07 (m, 3H) ppm. **^{13}C NMR** (101 MHz, $CDCl_3$) δ 174.06, 52.53, 42.41, 40.55, 40.01, 35.69, 35.54, 28.10, 26.47, 25.33, 18.11 ppm. **HRMS** (ESI): calcd. for $C_{12}H_{20}NO^+$: 194.1539. found: 194.1542.

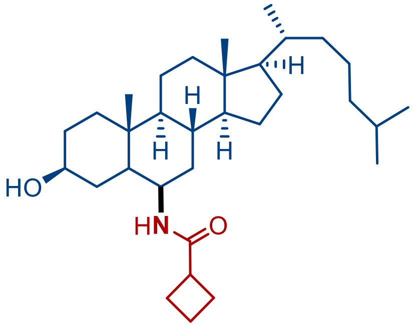

***N*-((3S,6R,8S,9S,10R,13R,14S,17R)-3-Hydroxy-10,13-dimethyl-17-((R)-6-methylheptan-2-yl)hexadecahydro-1H-cyclopenta[a]phenanthren-6-yl)cyclobutanecarboxamide (61):** white solid (43%). **M.P.** 175–177 ºC. **^{1}H NMR** (400 MHz, $CDCl_3$) δ 5.34 (dd, *J* = 4.9, 2.3 Hz, 1H), 3.51 (dt, *J* = 11.2, 6.0 Hz, 1H), 2.33 – 2.17 (m, 2H), 1.99 (tt, *J* = 16.1, 3.1 Hz, 2H), 1.83 (dtd, *J* = 12.8, 7.1, 3.4 Hz, 3H), 1.70 – 0.95 (m, 31H), 0.94 – 0.80 (m, 12H), 0.67 (s, 3H) ppm. **^{13}C NMR** (101 MHz, $CDCl_3$) δ 121.70, 71.78, 56.78, 56.17, 50.15, 42.33, 42.31, 39.80, 39.53, 37.27, 36.51, 36.21, 35.80, 31.92, 31.66, 28.25, 28.02, 24.30, 23.85, 22.83, 22.58, 21.10, 19.41, 18.73, 11.87 ppm. **HRMS** (ESI): calcd. for $C_{32}H_{56}NO_2^+$: 486.4306. found: 486.4309.

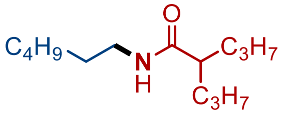

***N*-Hexyl-2-propylpentanamide (63):** white solid (79%). **M.P.** 55–56 ºC. **^{1}H NMR** (400 MHz, $CDCl_3$) δ 5.54 (d, *J* = 5.8 Hz, 1H), 3.23 (td, *J* = 7.2, 5.8 Hz, 2H), 2.07 – 1.94 (m, 1H), 1.66 – 1.41 (m, 4H), 1.38 – 1.19 (m, 12H), 0.94 – 0.79 (m, 9H) ppm. **^{13}C NMR** (101 MHz, $CDCl_3$) δ 175.82, 47.78, 39.26, 35.30, 31.43, 29.69, 26.54, 22.51, 20.80, 14.07, 13.93 ppm. **HRMS** (ESI): calcd. for $C_{14}H_{30}NO^+$: 228.2322. found: 228.2325.

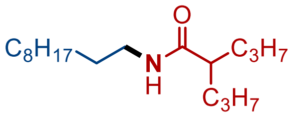

***N*-Decyl-2-propylpentanamide (64):** white solid (76%). **M.P.** 61–62 ºC. **^{1}H NMR** (400 MHz, $CDCl_3$) δ 5.44 (d, *J* = 6.0 Hz, 1H), 3.28 – 3.18 (m, 2H), 1.97 (dq, *J* = 9.5, 4.7 Hz, 1H), 1.62 – 1.53 (m, 2H), 1.47 (q, *J* = 7.0 Hz, 2H), 1.31 – 1.23 (m, 19H), 0.87 (td, *J* = 7.1, 4.6 Hz, 10H) ppm. **^{13}C NMR** (101 MHz, $CDCl_3$) δ

175.82, 47.84, 39.27, 35.31, 31.86, 29.74, 29.52, 29.49, 29.27, 29.25, 26.89, 22.64, 20.82, 14.09, 14.06 ppm. **HRMS** (ESI): calcd. for $C_{18}H_{38}NO^+$: 284.2948. found: 284.2950.

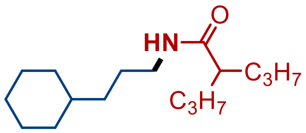

***N*-(3-Cyclohexylpropyl)-2-propylpentanamide (65):** white solid (86%). **M.P.** 80–81 ºC. **^{1}H NMR** (400 MHz, $CDCl_3$) δ 5.45 (t, *J* = 5.8 Hz, 1H), 3.22 (td, *J* = 7.2, 5.8 Hz, 2H), 1.97 (dd, *J* = 9.2, 4.7 Hz, 1H), 1.72 – 1.55 (m, 7H), 1.49 (t, *J* = 7.4 Hz, 2H), 1.38 – 1.15 (m, 12H), 0.88 (t, *J* = 7.0 Hz, 8H) ppm. **^{13}C NMR** (101 MHz, $CDCl_3$) δ 175.79, 47.83, 39.56, 37.32, 35.30, 34.60, 33.31, 27.09, 26.61, 26.30, 20.82, 14.09 ppm. **HRMS** (ESI): calcd. for $C_{17}H_{34}NO^+$: 268.2635 found: 268.2638.

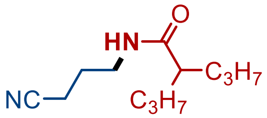

***N*-(3-Cyanopropyl)-2-propylpentanamide (66):** white solid (18%). **M.P.** 57–58 ºC. **^{1}H NMR** (400 MHz, $CDCl_3$) δ 5.67 (s, 1H), 3.38 (q, *J* = 6.6 Hz, 2H), 2.39 (t, *J* = 7.2 Hz, 2H), 2.03 (tt, *J* = 9.6, 5.0 Hz, 1H), 1.90 (p, *J* = 7.0 Hz, 2H), 1.62 – 1.50 (m, 2H), 1.42 – 1.21 (m, 6H), 0.89 (t, *J* = 7.2 Hz, 6H) ppm. **^{13}C NMR** (101 MHz, $CDCl_3$) δ 176.55, 119.21, 47.66, 38.12, 35.17, 25.71, 20.84, 14.82, 14.06 ppm. **HRMS** (ESI): calcd. for $C_{12}H_{23}N_2O^+$: 211.1805. found:211.1808.

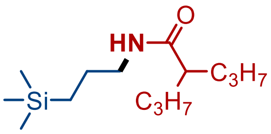

2-Propyl-N-(3-(trimethylsilyl)propyl)pentanamide (67): colorless oil (24%). **^{1}H NMR** (400 MHz, $CDCl_3$) δ 5.44 (s, 1H), 3.23 (q, *J* = 6.7 Hz, 2H), 1.98 (tt, *J* = 9.5, 4.4 Hz, 1H), 1.69 – 1.55 (m, 3H), 1.52 – 1.43 (m, 2H), 1.38 – 1.20 (m, 7H), 0.89 (t, *J* = 7.1 Hz, 6H), 0.53 – 0.44 (m, 2H), -0.02 (s, 9H) ppm. **^{13}C NMR** (101 MHz, $CDCl_3$) δ 175.78, 47.90, 42.34, 35.31, 24.36, 20.83, 14.10, 13.83, -1.80 ppm. **HRMS** (ESI): calcd. for $C_{14}H_{32}NOSi^+$: 258.2248. found: 258.2254.

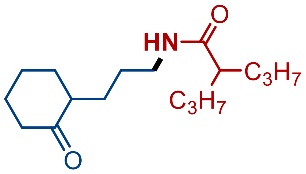

***N*-(3-(2-Oxocyclohexyl)propyl)-2-propylpentanamide (68):** colorless oil (30%). **^{1}H NMR** (400 MHz, $CDCl_3$) δ 5.74 (t, *J* = 5.6 Hz, 1H), 3.21 (ddt, *J* = 23.8, 13.1, 6.5 Hz, 2H), 2.38 – 2.26 (m, 2H), 2.14 – 1.79 (m, 5H), 1.78 – 1.41 (m, 7H), 1.38 – 1.16 (m, 8H), 0.87 (td, *J* = 7.2, 1.4 Hz, 6H) ppm. **^{13}C NMR** (101 MHz, $CDCl_3$) δ 213.44, 176.03, 50.35, 47.78, 42.16, 39.16, 35.29, 34.22, 28.06, 27.21, 26.54, 25.05, 20.83, 14.10 ppm. **HRMS** (ESI): calcd. for $C_{17}H_{32}NO_2^+$: 282.2428. found: 282.2422.

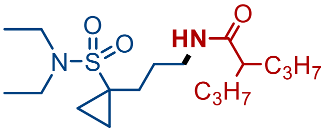

***N*-(3-(1-(N,N-Diethylsulfamoyl)cyclopropyl)propyl)-2-propyl-pentanamide (69):** colorless oil (74%). **^{1}H NMR** (400 MHz, $CDCl_3$) δ 5.64 (d, *J* = 6.0 Hz, 1H), 3.28 (dq, *J* = 29.5, 6.6, 6.1 Hz, 6H), 1.98 (tt, *J* = 9.6, 4.7 Hz, 1H), 1.75 – 1.69 (m, 3H), 1.60 – 1.51 (m, 2H), 1.39 – 1.17 (m, 15H), 0.88 (t, *J* =

7.1 Hz, 6H), 0.79 (t, *J* = 3.6 Hz, 2H) ppm. **^{13}C NMR** (101 MHz, $CDCl_3$) δ 176.16, 47.79, 41.58, 39.43, 39.03, 35.25, 29.32, 27.02, 20.88, 14.27, 14.12, 11.90 ppm. **HRMS** (ESI): calcd. for $C_{18}H_{37}N_2O_3^+$: 361.2519. found: 361.2520.

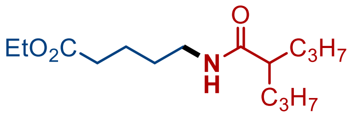

Ethyl 5-(2-propylpentanamido)pentanoate (70): white solid (67%). **M.P.** 47–48 ºC. **^{1}H NMR** (400 MHz, $CDCl_3$) δ 5.64 (d, *J* = 6.1 Hz, 1H), 4.10 (q, *J* = 7.1 Hz, 2H), 3.24 (td, *J* = 6.9, 5.7 Hz, 2H), 2.30 (t, *J* = 7.2 Hz, 2H), 1.98 (tq, *J* = 8.7, 4.1 Hz, 1H), 1.67 – 1.45 (m, 6H), 1.40 – 1.14 (m, 9H), 0.86 (t, *J* = 7.1 Hz, 6H) ppm. **^{13}C NMR** (101 MHz, $CDCl_3$) δ 175.97, 173.50, 60.30, 47.71, 38.72, 35.25, 33.71, 29.10, 22.05, 20.79, 14.18, 14.06 ppm. **HRMS** (ESI): calcd. for $C_{15}H_{30}NO_3^+$: 272.2220. found: 272.2221.

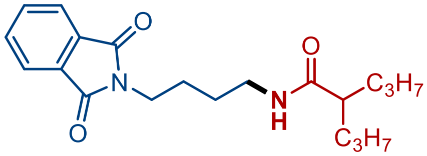

***N*-(3-Cyclohexylpropyl)-2-propylpentanamide (71):** white solid (61%). **M.P.** 137–138 ºC. **^{1}H NMR** (400 MHz, $CDCl_3$) δ 7.79 (dt, *J* = 7.5, 3.8 Hz, 2H), 7.68 (dd, *J* = 5.5, 3.1 Hz, 2H), 5.73 (t, *J* = 5.9 Hz, 1H), 3.67 (t, *J* = 7.1 Hz, 2H), 3.28 (q, *J* = 6.6 Hz, 2H), 1.98 (dq, *J* = 9.5, 4.6 Hz, 1H), 1.68 (q, *J* = 7.4 Hz, 2H), 1.61 – 1.48 (m, 4H), 1.33 – 1.17 (m, 6H), 0.84 (t, *J* = 7.0 Hz, 6H) ppm. **^{13}C NMR** (101 MHz, $CDCl_3$) δ 176.04, 168.38, 133.95, 132.06, 123.19, 47.69, 38.72, 37.45, 35.26, 26.92, 26.14, 20.83, 14.10 ppm. **HRMS** (ESI): calcd. for $C_{20}H_{29}N_2O_3^+$: 345.2173. found: 345.2172.

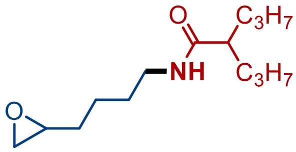

***N*-(4-(Oxiran-2-yl)butyl)-2-propylpentanamide (72):** white solid (66%). **M.P.** 45–46 ºC. **^{1}H NMR** (400 MHz, $CDCl_3$) δ 5.56 (d, *J* = 6.5 Hz, 1H), 3.25 (qd, *J* = 6.6, 4.6 Hz, 2H), 2.89 (ddd, *J* = 6.8, 5.4, 3.3 Hz, 1H), 2.73 (dd, *J* = 5.0, 4.0 Hz, 1H), 2.45 (dd, *J* = 5.0, 2.7 Hz, 1H), 1.98 (tt, *J* = 9.6, 4.7 Hz, 1H), 1.67 – 1.42 (m, 9H), 1.38 – 1.19 (m, 8H), 0.87 (t, *J* = 7.1 Hz, 7H) ppm. **^{13}C NMR** (101 MHz, $CDCl_3$) δ 176.00, 52.15, 47.79, 46.96, 39.08, 35.29, 31.97, 29.47, 23.41, 20.84, 14.11 ppm. **HRMS** (ESI): calcd. for $C_{14}H_{28}NO_2^+$: 242.2120. found: 242.2116.

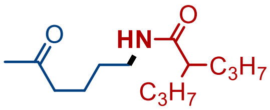

***N*-(5-Oxohexyl)-2-propylpentanamide (73):** white solid (36%). **M.P.** 47–48 ºC. **^{1}H NMR** (400 MHz, $CDCl_3$) δ 5.60 (s, 1H), 3.27 (td, *J* = 6.8, 5.8 Hz, 2H), 2.50 (t, *J* = 7.0 Hz, 2H), 2.16 (s, 3H), 2.02 (tt, *J* = 9.6, 4.7 Hz, 1H), 1.70 (s, 1H), 1.59 – 1.47 (m, 3H), 1.42 – 1.22 (m, 8H), 0.91 (t, *J* = 7.2 Hz, 6H) ppm. **^{13}C NMR** (101 MHz, $CDCl_3$) δ 208.83, 176.03, 47.84, 42.95, 38.75, 35.30, 29.97, 29.70, 29.09, 20.85, 20.59, 14.12 ppm. **HRMS** (ESI): calcd. for $C_{14}H_{28}NO_2^+$: 242.2115. found: 242.2119.

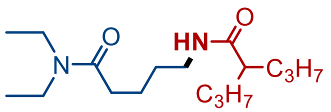

***N,N*-Diethyl-5-(2-propylpentanamido)pentanamide (74):** white solid (37%). **M.P.** 52–53 ºC. **^{1}H NMR** (400 MHz, $CDCl_3$) δ 5.97 (t, *J* = 5.8 Hz, 1H), 3.41 – 3.21 (m, 6H), 2.31 (t, *J* = 7.1 Hz, 2H), 2.01 (tt, *J* = 9.6, 4.6 Hz, 1H), 1.73 – 1.64 (m, 2H), 1.62 – 1.48 (m, 4H), 1.39 – 1.18 (m, 7H), 1.12 (dt, *J* = 24.8, 7.1 Hz, 6H), 0.87 (t, *J* = 7.1 Hz, 6H) ppm. **^{13}C NMR** (101 MHz, $CDCl_3$) δ 176.03, 171.82, 47.69, 41.91, 40.14, 38.69, 35.27, 32.22, 29.16, 22.12, 20.80, 14.31, 14.10, 13.07 ppm. **HRMS** (ESI): calcd. for $C_{17}H_{35}N_2O_2^+$: 299.2693. found: 299.2700.

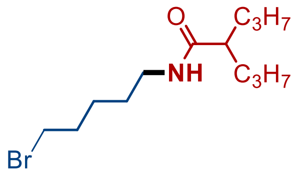

***N*-(5-Bromopentyl)-2-propylpentanamide (75):** white solid (44%). **M.P.** 44–45 ºC. **^{1}H NMR** (400 MHz, $CDCl_3$) δ 5.55 (t, *J* = 6.0 Hz, 1H), 3.39 (t, *J* = 6.6 Hz, 2H), 3.25 (q, *J* = 6.5 Hz, 2H), 1.98 (dq, *J* = 9.6, 4.7 Hz, 1H), 1.90 – 1.84 (m, 2H), 1.63 – 1.43 (m, 7H), 1.37 – 1.21 (m, 7H), 0.87 (t, *J* = 7.1 Hz, 6H) ppm. **^{13}C NMR** (101 MHz, $CDCl_3$) δ 176.02, 47.81, 38.97, 35.30, 33.65, 32.21, 28.91, 25.38, 20.85, 14.13 ppm. **HRMS** (ESI): calcd. for $C_{13}H_{27}BrNO^+$: 292.1276. found: 292.1271.

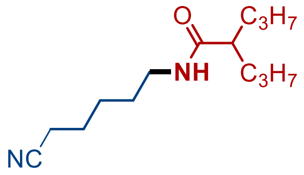

***N*-(5-Cyanopentyl)-2-propylpentanamide (76):** white solid (91%). **M.P.** 47–48 ºC. **^{1}H NMR** (400 MHz, $CDCl_3$) δ 5.58 (d, *J* = 6.1 Hz, 1H), 3.31 – 3.19 (m, 2H), 2.34 (t, *J* = 7.1 Hz, 2H), 1.99 (tt, *J* = 9.5, 4.7 Hz, 1H), 1.68 (p, *J* = 7.2 Hz, 2H), 1.62 – 1.43 (m, 6H), 1.40 – 1.20 (m, 6H), 0.87 (t, *J* = 7.1 Hz, 6H) ppm. **^{13}C NMR** (101 MHz, $CDCl_3$) δ 176.08, 119.51, 47.72, 38.74, 35.25, 28.97, 25.85, 24.91, 20.82, 17.06, 14.08 ppm. **HRMS** (ESI): calcd. for $C_{14}H_{27}N_2O^+$: 239.2118. found: 239.2123.

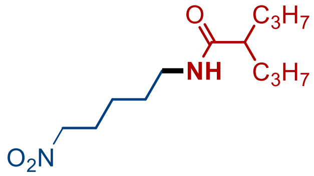

***N*-(5-Nitropentyl)-2-propylpentanamide (77):** white solid (74%). **M.P.** 44–46 ºC. **^{1}H NMR** (400 MHz, $CDCl_3$) δ 5.55 (d, *J* = 6.1 Hz, 1H), 4.37 (t, *J* = 6.9 Hz, 2H), 3.26 (q, *J* = 6.7 Hz, 2H), 2.00 (ddd, *J* = 14.0, 10.9, 6.1 Hz, 3H), 1.65 – 1.50 (m, 5H), 1.45 – 1.33 (m, 4H), 1.24 (ddd, *J* = 14.1, 7.1, 2.8 Hz, 4H), 0.88 (t, *J* = 7.1 Hz, 7H) ppm. **^{13}C NMR** (101 MHz, $CDCl_3$) δ 176.11, 75.46, 47.77, 38.69, 35.27, 29.06, 26.87, 23.56, 20.84, 14.10 ppm. **HRMS** (ESI): calcd. for $C_{13}H_{27}N_2O_3^+$: 259.2022. found: 259.2018.

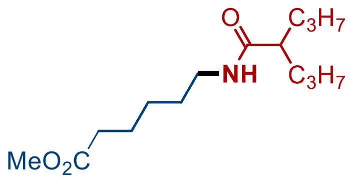

Methyl 6-(2-propylpentanamido)hexanoate (78): white solid (69%). **M.P.** 108–109 ºC. **^{1}H NMR** (400 MHz, $CDCl_3$) δ 5.57 (d, *J* = 6.2 Hz, 1H), 3.64 (s, 3H), 3.23 (q, *J* = 6.7 Hz, 2H), 2.29 (t, *J* = 7.4 Hz, 2H), 1.98 (dt, *J* = 9.3, 4.7 Hz, 1H), 1.65 – 1.46 (m, 6H), 1.36 –

1.20 (m, 8H), 0.86 (t, *J* = 7.1 Hz, 6H) ppm. **^{13}C NMR** (101 MHz, $CDCl_3$) δ 175.93, 174.04, 51.48, 47.76, 38.95, 35.29, 33.85, 29.38, 26.34, 24.42, 20.82, 14.10 ppm. **HRMS** (ESI): calcd. for $C_{15}H_{30}NO_3^+$: 272.2226. found: 272.2223.

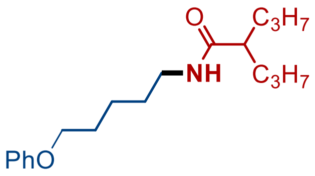

***N*-(5-Phenoxypentyl)-2-propylpentanamide (79):** white solid (76%). **M.P.** 77–78 ºC. **^{1}H NMR** (400 MHz, $CDCl_3$) δ 7.31 – 7.21 (m, 2H), 6.88 (d, *J* = 8.1 Hz, 3H), 5.59 (d, *J* = 6.0 Hz, 1H), 3.95 (t, *J* = 6.3 Hz, 2H), 3.28 (q, *J* = 6.5 Hz, 2H), 2.00 (dd, *J* = 9.3, 4.7 Hz, 1H), 1.84 – 1.76 (m, 2H), 1.63 – 1.49 (m, 6H), 1.36 – 1.23 (m, 6H), 0.89 (t, *J* = 7.1 Hz, 6H) ppm. **^{13}C NMR** (101 MHz, $CDCl_3$) δ 175.97, 158.99, 129.43, 120.57, 114.46, 67.51, 47.79, 39.16, 35.32, 29.53, 28.92, 23.52, 20.85, 14.14 ppm. **HRMS** (ESI): calcd. for $C_{19}H_{32}NO_2^+$: 306.2433. found: 306.2429.

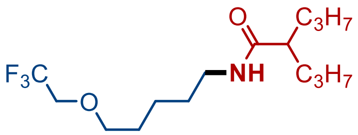

2-Propyl-N-(5-(2,2,2-trifluoroethoxy)pentyl)pentanamide (80): colorless oil (70%). **^{1}H NMR** (400 MHz, $CDCl_3$) δ 5.47 (t, *J* = 6.0 Hz, 1H), 3.78 (q, *J* = 8.8 Hz, 2H), 3.59 (t, *J* = 6.3 Hz, 2H), 3.25 (q, *J* = 6.7 Hz, 2H), 1.98 (dq, *J* = 9.5, 4.7 Hz, 1H), 1.68 – 1.48 (m, 6H), 1.41 – 1.21 (m, 8H), 0.87 (d, *J* = 6.9 Hz, 6H) ppm. **^{13}C NMR** (101 MHz, $CDCl_3$) δ 175.95, 122.67, 72.61, 68.46, 68.13, 47.83, 39.06, 35.30, 29.43, 29.06, 23.21, 20.83, 14.09 ppm. **^{19}F NMR** (376 MHz, $CDCl_3$) δ -74.23 ppm. **HRMS** (ESI): calcd. for $C_{15}H_{29}F_3NO_2^+$: 312.2145. found:312.2148.

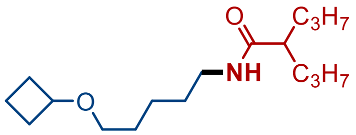

***N*-(5-Cyclobutoxypentyl)-2-propylpentanamide (81):** colorless oil (69%). **^{1}H NMR** (400 MHz, $CDCl_3$) δ 5.48 (t, *J* = 5.9 Hz, 1H), 4.97 – 4.83 (m, 1H), 4.09 (t, *J* = 6.5 Hz, 2H), 3.25 (td, *J* = 7.0, 5.8 Hz, 2H), 2.34 (dddd, *J* = 10.7, 7.7, 6.6, 2.7 Hz, 2H), 2.11 (dtd, *J* = 12.7, 10.0, 7.9 Hz, 2H), 1.98 (tt, *J* = 9.6, 4.7 Hz, 1H), 1.84 – 1.74 (m, 1H), 1.71 – 1.63 (m, 2H), 1.60 – 1.49 (m, 4H), 1.43 – 1.17 (m, 9H), 0.88 (t, *J* = 7.1 Hz, 6H) ppm. **^{13}C NMR** (101 MHz, $CDCl_3$) δ 175.96, 154.36, 71.52, 67.51, 47.81, 39.04, 35.29, 30.08, 29.36, 28.30, 23.13, 20.84, 14.11, 13.02 ppm. **HRMS** (ESI): calcd. for $C_{17}H_{34}NO_2^+$: 284.2590. found: 284.2586.

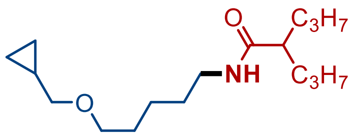

***N*-(5-(Cyclopropylmethoxy)pentyl)-2-propylpentanamide (82):** white solid (59%). **M.P.** 45–46 ºC. **^{1}H NMR** (400 MHz, $CDCl_3$) δ 5.46 (t, *J* = 6.1 Hz, 1H), 4.12 (t, *J* = 6.5 Hz, 2H), 3.95 (d, *J* = 7.3 Hz, 2H), 3.25 (q, *J* = 6.7 Hz, 2H), 1.98 (tt, *J* = 9.5, 4.5 Hz, 1H), 1.72 – 1.66 (m, 3H), 1.60 – 1.49 (m, 4H), 1.44 – 1.16 (m, 10H), 0.88 (t, *J* = 7.1 Hz, 6H), 0.66 – 0.51 (m, 2H), 0.36 – 0.24 (m, 2H) ppm. **^{13}C**

NMR (101 MHz, $CDCl_3$) δ 175.94, 155.40, 72.81, 67.63, 47.83, 39.05, 35.29, 29.38, 28.32, 23.14, 20.85, 14.11, 9.76, 3.27 ppm. **HRMS** (ESI): calcd. for $C_{17}H_{34}NO_2^+$: 284.2590. found: 284.2587.

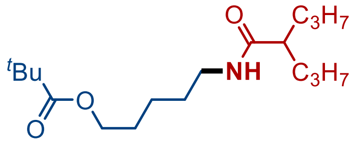

5-(2-Propylpentanamido)pentyl pivalate (83): white solid (65%). **M.P.** 88–89 °C. **^{1}H NMR** (400 MHz, $CDCl_3$) δ 5.52 (t, *J* = 5.8 Hz, 1H), 4.03 (t, *J* = 6.5 Hz, 2H), 3.25 (q, *J* = 6.7 Hz, 2H), 1.98 (tt, *J* = 9.5, 4.7 Hz, 1H), 1.69 – 1.48 (m, 7H), 1.40 – 1.22 (m, 8H), 1.17 (s, 9H), 0.87 (t, *J* = 7.1 Hz, 6H) ppm. **^{13}C NMR** (101 MHz, $CDCl_3$) δ 178.64, 175.97, 64.10, 47.80, 39.11, 38.73, 35.28, 29.41, 28.32, 27.18, 23.34, 20.83, 14.11 ppm. **HRMS** (ESI): calcd. for $C_{18}H_{36}NO_3^+$: 314.2695. found: 314.2691.

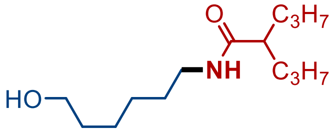

***N*-(6-Hydroxyhexyl)-2-propylpentanamide (84):** white solid (62%). **M.P.** 33–35 °C. **^{1}H NMR** (400 MHz, $CDCl_3$) δ 5.67 (t, *J* = 6.0 Hz, 1H), 3.60 (t, *J* = 6.5 Hz, 2H), 3.23 (q, *J* = 6.7 Hz, 2H), 2.16 (s, 1H), 1.98 (tt, *J* = 9.7, 4.5 Hz, 1H), 1.60 – 1.45 (m, 6H), 1.41 – 1.15 (m, 11H), 0.86 (t, *J* = 7.1 Hz, 6H) ppm. **^{13}C NMR** (101 MHz, $CDCl_3$) δ 176.10, 62.52, 47.74, 39.05, 35.27, 32.53, 29.70, 26.47, 25.25, 20.82, 14.10 ppm. **HRMS** (ESI): calcd. for $C_{14}H_{30}NO_2^+$: 244.2277. found: 244.2273.

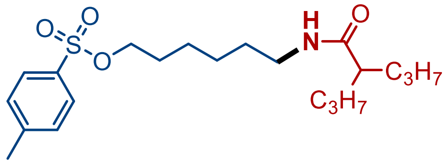

6-(2-Propylpentanamido)hexyl 4-methyl-benzenesulfonate (85): white solid (71%). **M.P.** 56–58 °C. **^{1}H NMR** (400 MHz, $CDCl_3$) δ 7.79 – 7.69 (m, 2H), 7.32 (d, *J* = 8.1 Hz, 2H), 5.65 (t, *J* = 5.8 Hz, 1H), 3.98 (t, *J* = 6.4 Hz, 2H), 3.17 (td, *J* = 7.2, 5.8 Hz, 2H), 2.42 (s, 3H), 1.98 (dt, *J* = 9.3, 4.7 Hz, 1H), 1.64 – 1.51 (m, 4H), 1.42 (q, *J* = 7.2 Hz, 2H), 1.33 – 1.19 (m, 11H), 0.85 (t, *J* = 7.1 Hz, 6H) ppm. **^{13}C NMR** (101 MHz, $CDCl_3$) δ 175.98, 144.77, 133.08, 129.85, 127.82, 70.49, 47.65, 38.96, 35.28, 29.49, 28.69, 26.15, 24.98, 21.61, 20.80, 14.10 ppm. **HRMS** (ESI): calcd. for $C_{21}H_{36}NO_4S^+$: 398.2360. found: 398.2365.

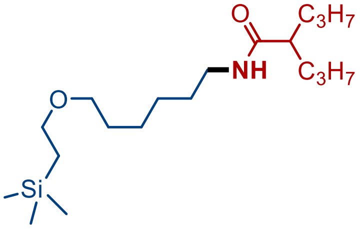

2-Propyl-N-(6-(2-(trimethylsilyl)ethoxy)hexyl)pentanamide (86): colorless oil (78%). **^{1}H NMR** (400 MHz, $CDCl_3$) δ 5.53 (d, *J* = 7.7 Hz, 1H), 4.31 – 4.15 (m, 2H), 4.09 (t, *J* = 6.6 Hz, 2H), 3.22 (td, *J* = 7.1, 5.8 Hz, 2H), 1.97 (dq, *J* = 9.6, 4.7 Hz, 1H), 1.70 – 1.42 (m, 7H), 1.42 – 1.15 (m, 11H), 1.07 – 0.98 (m, 2H), 0.86 (t, *J* = 7.2 Hz, 6H), 0.02 (s, 8H) ppm. **^{13}C NMR** (101 MHz, $CDCl_3$) δ 175.91, 155.36, 67.58, 66.24, 47.79, 39.10, 35.30, 29.62, 28.59, 26.46, 25.37, 20.83, 17.54, 14.10, -1.58 ppm. **HRMS** (ESI): calcd. for $C_{19}H_{42}NO_2Si^+$: 344.2985. found: 344.2981.

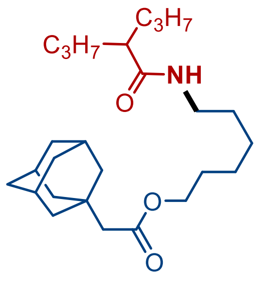

6-(2-Propylpentanamido)hexyl 2-((3r,5r,7r)-adamantan-1-yl)acetate (87): colorless oil (63%). **^{1}H NMR** (400 MHz, $CDCl_3$) δ 5.51 (t, *J* = 5.8 Hz, 1H), 4.02 (t, *J* = 6.7 Hz, 2H), 3.23 (td, *J* = 7.2, 5.9 Hz, 2H), 2.04 (s, 2H), 1.97 – 1.92 (m, 3H), 1.67 (s, 3H), 1.64 – 1.56 (m, 12H), 1.49 (t, *J* = 7.0 Hz, 2H), 1.40 – 1.18 (m, 11H), 0.87 (t, *J* = 7.1 Hz, 6H) ppm. **^{13}C NMR** (101 MHz, $CDCl_3$) δ 175.92, 171.96, 63.80, 49.03, 47.81, 42.41, 39.12, 36.74, 35.30, 32.73, 29.67, 28.61, 26.47, 25.64, 20.84, 14.12 ppm. **HRMS** (ESI): calcd. for $C_{26}H_{46}NO_3^+$: 420.3478. found: 420.3472.

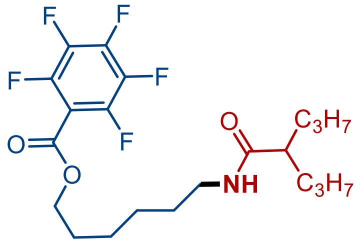

6-(2-Propylpentanamido)hexyl 2,3,4,5,6-pentafluorobenzoate (88): white solid (79%). **M.P.** 64–66 ºC. **^{1}H NMR** (400 MHz, $CDCl_3$) δ 5.51 (d, *J* = 6.1 Hz, 1H), 4.36 (t, *J* = 6.5 Hz, 2H), 3.24 (td, *J* = 7.2, 5.8 Hz, 2H), 1.98 (tt, *J* = 9.6, 4.5 Hz, 1H), 1.73 (dt, *J* = 8.2, 6.6 Hz, 2H), 1.57 – 1.22 (m, 14H), 0.87 (t, *J* = 7.2 Hz, 6H) ppm. **^{13}C NMR** (101 MHz, $CDCl_3$) δ 175.95, 66.72, 47.80, 39.09, 35.29, 29.66, 28.32, 26.39, 25.41, 20.83, 14.08 ppm. **^{19}F NMR** (376 MHz, $CDCl_3$) δ -138.35 – -138.49 (m), -148.78, -148.83 (t, *J* = 4.4 Hz), -148.89, -160.39 (td, *J* = 20.2, 6.1 Hz) ppm. **HRMS** (ESI): calcd. for $C_{21}H_{29}F_5NO_3^+$: 438.2068. found: 438.2061.

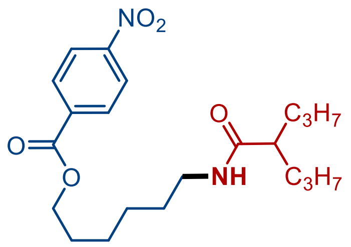

6-(2-Propylpentanamido)hexyl 4-nitrobenzoate (89): white solid (56%). **M.P.** 91–92 ºC. **^{1}H NMR** (400 MHz, $CDCl_3$) δ 8.49 – 8.23 (m, 2H), 8.19 (d, *J* = 7.7 Hz, 2H), 5.50 (t, *J* = 5.9 Hz, 1H), 4.35 (t, *J* = 6.6 Hz, 2H), 3.26 (q, *J* = 6.8 Hz, 2H), 1.98 (dd, *J* = 9.3, 4.8 Hz, 1H), 1.78 (q, *J* = 7.1 Hz, 2H), 1.62 – 1.22 (m, 14H), 0.87 (t, *J* = 7.0 Hz, 6H) ppm. **^{13}C NMR** (101 MHz, $CDCl_3$) δ 175.92, 164.72, 150.54, 135.80, 130.66, 123.51, 65.82, 47.79, 39.08, 35.28, 29.69, 28.55, 26.47, 25.59, 20.82, 14.08 ppm. **HRMS** (ESI): calcd. for $C_{21}H_{33}N_2O_5^+$: 393.2389. found: 393.2386.

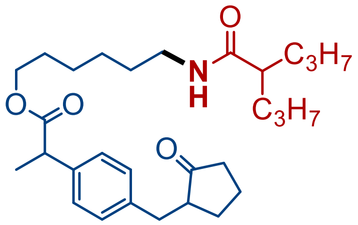

6-(2-Propylpentanamido)hexyl 2-(4-((2-oxocyclopentyl)methyl) phenyl)propanoate (90): colorless oil (71%). **^{1}H NMR** (400 MHz, $CDCl_3$) δ 7.19 (d, *J* = 8.0 Hz, 2H), 7.10 (d, *J* = 7.8 Hz, 2H), 5.65 – 5.45 (m, 1H), 4.03 (t, *J* = 6.6 Hz, 2H), 3.66 (q, *J* = 7.2 Hz, 1H), 3.20 (q, *J* = 6.7 Hz, 2H), 3.09 (dd, *J* = 13.9, 4.2 Hz, 1H), 2.50 (dd, *J* = 13.9, 9.4 Hz, 1H), 2.32 (ddd, *J* = 20.1, 8.8, 3.2 Hz, 2H), 2.14 – 1.90 (m, 4H), 1.58 – 1.18 (m, 20H), 0.87 (t, *J* = 7.0 Hz, 6H) ppm. **^{13}C NMR** (101 MHz, $CDCl_3$) δ 175.92, 174.68, 138.75, 138.47,

129.06, 127.51, 64.52, 50.92, 47.76, 45.16, 39.08, 38.17, 35.30, 35.16, 29.63, 29.19, 28.44, 26.38, 25.37, 20.83, 20.53, 18.42, 14.12 ppm. **HRMS** (ESI): calcd. for $C_{29}H_{46}NO_4^+$: 472.3427. found: 472.3423.

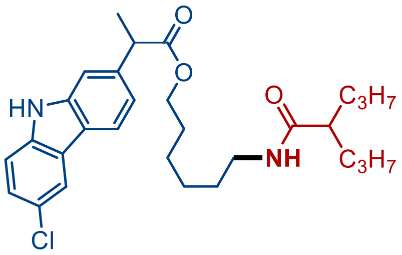

6-(2-Propylpentanamido)hexyl 2-(6-chloro-9H-carbazol-2-yl)propanoate (91): colorless oil (66%). **^{1}H NMR** (400 MHz, $CDCl_3$) δ 9.61 (s, 1H), 8.01 – 7.90 (m, 2H), 7.41 – 7.29 (m, 3H), 7.15 (dd, *J* = 8.1, 1.5 Hz, 1H), 5.49 (t, *J* = 5.7 Hz, 1H), 4.15 (dt, *J* = 10.9, 6.2 Hz, 1H), 4.00 (dt, *J* = 11.2, 5.9 Hz, 1H), 3.86 (q, *J* = 7.1 Hz, 1H), 3.15 (tdd, *J* = 7.3, 5.7, 3.6 Hz, 2H), 2.06 – 1.97 (m, 1H), 1.68 – 1.52 (m, 6H), 1.45 – 1.17 (m, 12H), 0.90 (td, *J* = 7.2, 2.0 Hz, 6H) ppm. **^{13}C NMR** (101 MHz, $CDCl_3$) δ 176.44, 174.78, 139.11, 138.52, 125.51, 124.39, 120.40, 119.74, 119.24, 111.83, 109.75, 64.42, 47.80, 46.11, 39.15, 35.33, 29.34, 28.36, 26.11, 25.25, 20.86, 18.48, 14.13 ppm. **HRMS** (ESI): calcd. for $C_{29}H_{40}ClN_2O_3^+$: 499.2727. found: 499.2724.

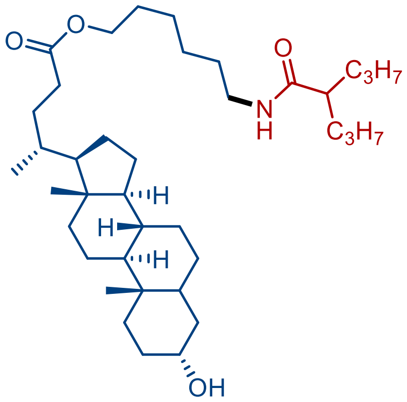

6-(2-Propylpentanamido)hexyl (4R)-4-((3R,8R,9S,10S,13R,14S,17R)-3-hydroxy-10,13-dimethylhexadecahydro-1H-cyclopenta[a]phenanthren-17-yl)pentanoate (92): colorless oil (67%). **^{1}H NMR** (400 MHz, $CDCl_3$) δ 5.59 (t, *J* = 5.8 Hz, 1H), 4.02 (t, *J* = 6.6 Hz, 2H), 3.59 (tt, *J* = 10.6, 4.7 Hz, 1H), 3.22 (q, *J* = 6.7 Hz, 2H), 2.31 (ddd, *J* = 15.2, 10.0, 5.2 Hz, 1H), 2.18 (ddd, *J* = 15.6, 9.5, 6.6 Hz, 1H), 2.01 – 1.71 (m, 9H), 1.63 – 1.44 (m, 9H), 1.41 – 0.96 (m, 29H), 0.90 – 0.80 (m, 13H), 0.61 (s, 3H) ppm. **^{13}C NMR** (101 MHz, $CDCl_3$) δ 71.77, 64.12, 56.49, 55.93, 47.75, 42.72, 42.09, 40.42, 40.17, 39.13, 36.42, 35.84, 35.36, 35.33, 35.29, 34.56, 31.26, 31.01, 30.51, 29.64, 28.58, 28.18, 27.19, 26.49, 26.42, 25.56, 24.19, 23.37, 20.83, 20.81, 18.26, 14.12, 12.02 ppm. **HRMS** (ESI): calcd. for $C_{38}H_{68}NO_4^+$: 602.5148. found: 602.5145.

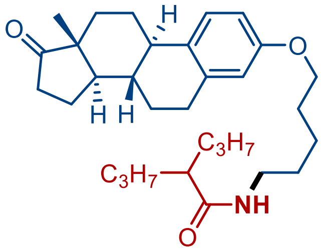

***N*-(5-(((8S,9R,13R,14R)-13-Methyl-17-oxo-7,8,9,11,12,13,14,15,16,17-decahydro-6H-cyclopenta[a]phenanthren-3-yl)oxy)pentyl)-2-propylpentanamide (93):** white solid (62%). **M.P.** 132–134 °C. **^{1}H NMR** (400 MHz, $CDCl_3$) δ 7.18 (d, *J* = 8.6 Hz, 1H), 6.69 (dd, *J* = 8.6, 2.7 Hz, 1H), 6.62 (d, *J* = 2.7 Hz, 1H), 5.52 (s, 1H), 3.92 (t, *J* = 6.3 Hz, 2H), 3.28 (q, *J* = 6.5 Hz, 2H), 2.88 (dq, *J* = 10.7, 7.0, 6.2 Hz, 2H), 2.49 (dd, *J* = 18.8, 8.6 Hz, 1H), 2.43 – 2.35 (m, 1H), 2.30 – 2.19 (m, 1H), 2.18 –

1.92 (m, 5H), 1.77 (q, J = 6.4 Hz, 3H), 1.67 – 1.42 (m, 12H), 1.38 – 1.17 (m, 7H), 0.96 – 0.83 (m, 9H) ppm. **^{13}C NMR** (101 MHz, $CDCl_3$) δ 175.95, 157.03, 137.73, 131.96, 126.31, 114.52, 112.10, 67.57, 50.42, 48.02, 47.83, 43.99, 39.17, 38.39, 35.89, 35.32, 31.60, 29.66, 29.50, 28.93, 26.57, 25.93, 23.51, 21.59, 20.86, 14.14, 13.86 ppm. **HRMS** (ESI): calcd. for $C_{31}H_{48}NO_3^+$: 482.3634. found: 482.3632.

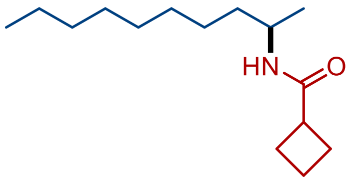

***N*-(Decan-2-yl)cyclobutanecarboxamide (134):** white solid (25%). **M.P.** 56–57 ºC. **^{1}H NMR** (400 MHz, $CDCl_3$) δ 5.06 (d, J = 8.6 Hz, 1H), 3.95 (dq, J = 8.6, 6.5 Hz, 1H), 2.94 (p, J = 8.5 Hz, 1H), 2.25 (ddt, J = 14.0, 9.0, 4.4 Hz, 2H), 2.13 (dtd, J = 11.6, 8.2, 3.6 Hz, 2H), 1.99 – 1.80 (m, 2H), 1.39 (q, J = 7.1, 6.4 Hz, 2H), 1.26 (d, J = 7.7 Hz, 12H), 1.10 (d, J = 6.6 Hz, 3H), 0.87 (t, J = 6.7 Hz, 3H) ppm ppm. **^{13}C NMR** (101 MHz, $CDCl_3$) δ 174.14, 44.97, 40.14, 37.07, 31.85, 29.51, 29.23, 26.00, 25.41, 25.32, 22.65, 21.05, 18.10, 14.08 ppm. **HRMS** (ESI): calcd. for $C_{15}H_{30}NO^+$: 240.2322. found: 240.2329.

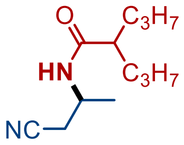

***N*-(1-Cyanopropan-2-yl)-2-propylpentanamide (S101):** white solid (73%). **M.P.** 70–71 ºC. **^{1}H NMR** (400 MHz, $CDCl_3$) δ 5.74 (d, J = 7.4 Hz, 1H), 4.30 – 4.17 (m, 1H), 2.82 (dd, J = 16.7, 5.4 Hz, 1H), 2.50 (dd, J = 16.8, 3.8 Hz, 1H), 2.04 (tt, J = 9.7, 4.9 Hz, 1H), 1.56 (dtdd, J = 12.5, 8.9, 5.6, 3.1 Hz, 2H), 1.37 – 1.31 (m, 5H), 1.26 (tdd, J = 9.4, 7.4, 5.3 Hz, 4H), 0.88 (td, J = 7.2, 3.3 Hz, 6H) ppm. **^{13}C NMR** (101 MHz, $CDCl_3$) δ 176.00, 117.21, 47.37, 41.62, 35.18, 35.08, 24.63, 20.76, 20.71, 19.20, 14.06, 14.04 ppm. **HRMS** (ESI): calcd. for $C_{12}H_{23}N_2O^+$: 211.1805. found:211.1809.

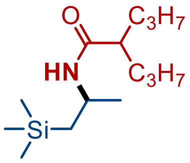

2-Propyl-N-(1-(trimethylsilyl)propan-2-yl)pentanamide (S102): colorless oil (70%). **^{1}H NMR** (400 MHz, $CDCl_3$) δ 5.30 (d, J = 7.8 Hz, 1H), 4.09 (tt, J = 8.3, 6.4 Hz, 1H), 1.96 – 1.86 (m, 1H), 1.61 – 1.49 (m, 2H), 1.37 – 1.19 (m, 6H), 1.14 (d, J = 6.4 Hz, 3H), 0.87 (t, J = 7.2 Hz, 7H), 0.71 (dd, J = 14.5, 8.4 Hz, 1H), 0.02 (s, 9H) ppm. **^{13}C NMR** (101 MHz, $CDCl_3$) δ 174.56, 47.81, 42.95, 35.40, 35.22, 26.08, 24.18, 20.85, 20.79, 14.15, 14.13 ppm. **HRMS** (ESI): calcd. for $C_{14}H_{32}NOSi^+$: 258.2248. found: 258.2253.

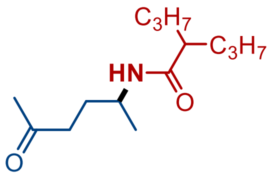

***N*-(5-Oxohexan-2-yl)-2-propylpentanamide (S103):** white solid (23%). **M.P.** 53–54 ºC. **^{1}H NMR** (400 MHz, CDCl3) δ 5.42 (d, J = 8.5 Hz, 1H), 3.95 (dq, J = 8.1, 6.6 Hz, 1H), 2.57 (dt, J = 18.2, 7.1 Hz, 1H), 2.44 (dt, J = 18.2, 6.9 Hz, 1H), 2.13 (s, 3H), 1.94 (tt, J = 9.6, 4.6 Hz, 1H), 1.69 (d, J = 6.9 Hz, 2H), 1.56 (ddt, J = 17.0, 9.3, 4.8 Hz, 2H), 1.39 – 1.22 (m, 6H), 1.13 (d, J = 6.6 Hz, 3H), 0.88 (t, J

= 7.1 Hz, 6H) ppm. **^{13}C NMR** (101 MHz, $CDCl_3$) δ 208.94, 175.50, 47.92, 45.13, 40.69, 35.38, 35.19, 30.04, 30.00, 21.62, 20.90, 20.80, 14.12 ppm. **HRMS** (ESI): calcd. for $C_{14}H_{28}NO_2^+$: 242.2115. found: 242.2120.

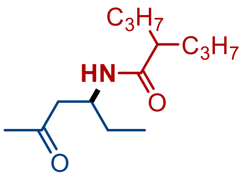

***N*-(5-Oxohexan-3-yl)-2-propylpentanamide (S104):** white solid (30%). **M.P.** 55–57 ^{o}C. **^{1}H NMR** (400 MHz, $CDCl_3$) δ 5.89 (d, *J* = 9.0 Hz, 1H), 4.14 (tq, *J* = 8.5, 5.5 Hz, 1H), 2.66 (t, *J* = 4.9 Hz, 2H), 2.15 (s, 3H), 1.99 (dq, *J* = 9.4, 4.6 Hz, 1H), 1.66 (s, 1H), 1.58 – 1.50 (m, 3H), 1.36 – 1.19 (m, 6H), 0.88 (ddd, *J* = 12.4, 8.2, 6.4 Hz, 9H) ppm. **^{13}C NMR** (101 MHz, $CDCl_3$) δ 208.34, 175.58, 47.80, 47.44, 47.12, 35.31, 35.23, 30.51, 27.16, 20.81, 20.73, 14.10, 14.09, 10.88 ppm. **HRMS** (ESI): calcd. for $C_{14}H_{28}NO_2^+$: 242.2115. found: 242.2121.

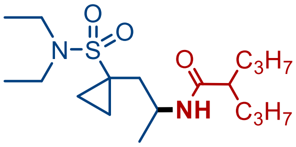

***N*-(1-(1-(N,N-Diethylsulfamoyl)cyclopropyl)propan-2-yl)-2-propylpentanamide (S105):** white solid (8%). **M.P.** 56–57 ^{o}C. **^{1}H NMR** (400 MHz, $CDCl_3$) δ 6.25 (d, *J* = 7.2 Hz, 1H), 3.90 (dq, *J* = 9.6, 6.4 Hz, 1H), 3.42 (dq, *J* = 14.4, 7.2 Hz, 2H), 3.31 (dq, *J* = 14.3, 7.1 Hz, 2H), 2.23 (dd, *J* = 15.6, 9.6 Hz, 1H), 2.02 (tt, *J* = 9.5, 4.3 Hz, 1H), 1.71 – 1.59 (m, 3H), 1.40 – 1.26 (m, 7H), 1.23 – 1.18 (m, 8H), 0.89 (t, *J* = 7.1 Hz, 6H) ppm. **^{13}C NMR** (101 MHz, $CDCl_3$) δ 175.71, 47.61, 43.42, 41.65, 37.71, 36.88, 35.34, 35.20, 21.37, 20.78, 14.45, 14.15, 14.12, 10.83 ppm. **HRMS** (ESI): calcd. for $C_{18}H_{37}N_2O_3^+$: 361.2519. found: 361.2524.

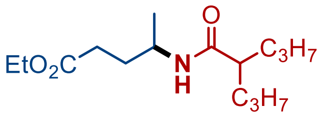

Ethyl-4-(2-propylpentanamido)pentanoate (S106): white solid (11%). **M.P.** 57–58 ^{o}C. **^{1}H NMR** (400 MHz, $CDCl_3$) δ 5.45 (d, *J* = 8.5 Hz, 1H), 4.25 – 3.91 (m, 3H), 2.45 – 2.26 (m, 2H), 1.95 (tt, *J* = 9.6, 4.5 Hz, 1H), 1.84 – 1.67 (m, 3H), 1.57 (dddt, *J* = 16.7, 14.9, 8.9, 4.5 Hz, 2H), 1.35 – 1.21 (m, 8H), 1.15 (d, *J* = 6.5 Hz, 3H), 0.88 (t, *J* = 7.2 Hz, 6H) ppm. **^{13}C NMR** (101 MHz, $CDCl_3$) δ 175.42, 173.79, 60.54, 47.92, 44.98, 35.34, 35.18, 31.34, 31.19, 21.30, 20.81, 20.77, 14.15, 14.09 ppm. **HRMS** (ESI): calcd. for $C_{15}H_{30}NO_3^+$: 272.2220. found: 272.2225.

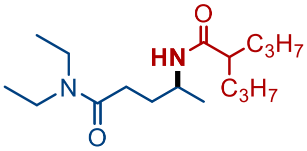

***N,N*-Diethyl-4-(2-propylpentanamido)pentanamide (S111):** white solid (51%). **M.P.** 58–59 ^{o}C. **^{1}H NMR** (400 MHz, $CDCl_3$) δ 6.23 (d, *J* = 7.9 Hz, 1H), 3.90 (dddd, *J* = 10.5, 7.8, 6.3, 4.3 Hz, 1H), 3.45 (dq, *J* = 14.1, 7.1 Hz, 1H), 3.31 – 3.22 (m, 3H), 2.45 (dt, *J* = 16.5, 6.6 Hz, 1H), 2.25 (dt, *J* = 16.5, 6.9 Hz, 1H), 1.97 – 1.91 (m, 2H), 1.87 – 1.80 (m, 1H), 1.74 (dtd, *J* = 14.1, 6.7, 4.4 Hz, 1H), 1.61 – 1.49 (m, 2H), 1.35 – 1.19 (m, 6H), 1.16 – 1.04 (m, 10H), 0.85 (q, *J* = 7.1 Hz,

6H) ppm. **^{13}C NMR** (101 MHz, $CDCl_3$) δ 175.58, 172.11, 47.89, 45.87, 41.84, 40.36, 35.38, 35.19, 30.73, 30.23, 21.68, 20.87, 20.73, 14.12, 14.08, 13.00 ppm. **HRMS** (ESI): calcd. for $C_{17}H_{35}N_2O_2^+$: 299.2693. found: 299.2699.

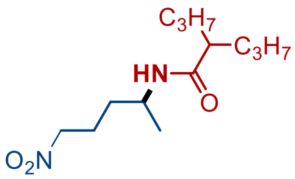

***N*-(5-Nitropentan-2-yl)-2-propylpentanamide (S114):** white solid (11%). **M.P.** 52–53 ºC. **^{1}H NMR** (400 MHz, $CDCl_3$) δ 5.23 (d, *J* = 8.8 Hz, 1H), 4.42 (d, *J* = 6.8 Hz, 2H), 4.17 – 3.99 (m, 1H), 2.01 (dtd, *J* = 19.5, 9.4, 8.4, 5.3 Hz, 3H), 1.63 – 1.51 (m, 3H), 1.39 – 1.23 (m, 7H), 1.16 (d, *J* = 6.6 Hz, 3H), 0.89 (td, *J* = 7.1, 2.6 Hz, 7H) ppm. **^{13}C NMR** (101 MHz, $CDCl_3$) δ 175.60, 75.23, 47.90, 44.00, 35.36, 35.22, 33.73, 24.14, 21.35, 20.88, 20.83, 14.11 ppm. **HRMS** (ESI): calcd. for $C_{13}H_{27}N_2O_3^+$: 259.2022. found: 259.2021.

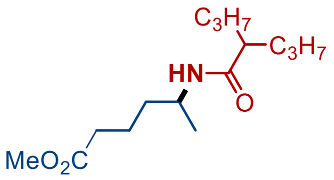

Methyl-5-(2-propylpentanamido)hexanoate (S115): white solid (10%). **M.P.** 110–112 ºC. **^{1}H NMR** (400 MHz, $CDCl_3$) δ 5.25 (d, *J* = 8.6 Hz, 1H), 4.08 – 3.95 (m, 1H), 3.66 (s, 3H), 2.33 (td, *J* = 7.3, 4.7 Hz, 2H), 1.96 (tt, *J* = 9.4, 4.3 Hz, 1H), 1.66 – 1.52 (m, 4H), 1.48 – 1.40 (m, 2H), 1.39 – 1.25 (m, 6H), 1.13 (d, *J* = 6.6 Hz, 3H), 0.89 (t, *J* = 6.9 Hz, 6H) ppm. **^{13}C NMR** (101 MHz, $CDCl_3$) δ 175.29, 173.95, 51.52, 47.95, 44.59, 36.19, 35.40, 35.27, 33.60, 21.35, 21.22, 20.83, 14.13 ppm. **HRMS** (ESI): calcd. for $C_{15}H_{30}NO_3^+$: 272.2226. found: 272.2227.

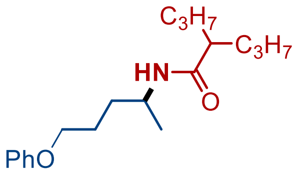

***N*-(5-Phenoxypentan-2-yl)-2-propylpentanamide (S116):** white solid (9%). **M.P.** 85–86 ºC. **^{1}H NMR** (400 MHz, $CDCl_3$) δ 7.34 – 7.27 (m, 2H), 6.98 – 6.85 (m, 3H), 5.26 (d, *J* = 8.7 Hz, 1H), 4.16 – 4.05 (m, 1H), 3.98 (td, *J* = 6.2, 2.0 Hz, 2H), 1.95 (dt, *J* = 9.5, 4.7 Hz, 1H), 1.82 (ddt, *J* = 11.0, 6.7, 3.6 Hz, 2H), 1.63 – 1.55 (m, 4H), 1.40 – 1.21 (m, 6H), 1.17 (d, *J* = 6.5 Hz, 3H), 0.90 (td, *J* = 7.1, 3.2 Hz, 6H) ppm. **^{13}C NMR** (101 MHz, $CDCl_3$) δ 175.30, 158.91, 129.45, 120.65, 114.50, 67.41, 48.01, 44.70, 35.40, 35.28, 33.54, 26.00, 21.36, 20.87, 20.84, 14.14 ppm. **HRMS** (ESI): calcd. for $C_{19}H_{32}NO_2^+$: 306.2433. found: 306.2434.

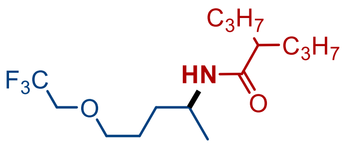

2-Propyl-N-(5-(2,2,2-trifluoroethoxy)pentan-2-yl)pentanamide (S117): colorless oil (13%). **^{1}H NMR** (400 MHz, $CDCl_3$) δ 5.21 (d, *J* = 8.6 Hz, 1H), 4.03 (tt, *J* = 7.9, 6.1 Hz, 1H), 3.79 (q, *J* = 8.8 Hz, 2H), 3.61 (t, *J* = 6.1 Hz, 2H), 1.95 (tt, *J* = 9.4, 4.3 Hz, 1H), 1.68 – 1.54 (m, 5H), 1.36 – 1.23 (m, 7H), 1.14 (d, *J* = 6.6 Hz, 3H), 0.89 (t, *J* = 6.9 Hz, 6H) ppm. **^{13}C NMR** (101 MHz, $CDCl_3$) δ 175.31, 72.43, 68.50, 68.16, 47.98, 44.56, 35.40, 35.27, 33.23, 29.70, 26.17, 21.33, 20.85, 20.82, 14.12, 14.10

ppm. **^{19}F NMR** (376 MHz, $CDCl_3$) δ -74.23 ppm. **HRMS** (ESI): calcd. for $C_{15}H_{29}F_3NO_2^+$: 312.2145. found:312.2151.

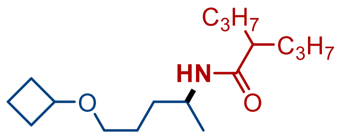

***N*-(5-Cyclobutoxypentan-2-yl)-2-propylpentanamide (S118):** colorless oil (17%). **^{1}H NMR** (400 MHz, $CDCl_3$) δ 5.19 (d, *J* = 8.6 Hz, 1H), 4.97 – 4.84 (m, 1H), 4.11 (t, *J* = 6.4 Hz, 2H), 4.03 (ddq, *J* = 14.7, 8.4, 6.5 Hz, 1H), 2.41 – 2.28 (m, 2H), 2.21 – 2.05 (m, 2H), 1.95 (tt, *J* = 9.5, 4.4 Hz, 1H), 1.87 – 1.73 (m, 1H), 1.68 (ddt, *J* = 11.8, 6.9, 3.8 Hz, 2H), 1.55 (dddd, *J* = 18.4, 9.6, 7.5, 4.1 Hz, 4H), 1.35 – 1.22 (m, 7H), 1.13 (d, *J* = 6.6 Hz, 3H), 0.89 (t, *J* = 7.0 Hz, 6H) ppm. **^{13}C NMR** (101 MHz, $CDCl_3$) δ 175.31, 154.29, 71.56, 67.51, 47.97, 44.64, 35.39, 35.26, 33.22, 30.09, 25.54, 21.34, 20.87, 20.83, 14.13, 13.02 ppm. **HRMS** (ESI): calcd. for $C_{17}H_{34}NO_2^+$: 284.2590. found: 284.2588.

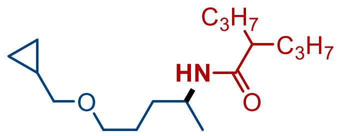

***N*-(5-(Cyclopropylmethoxy)pentan-2-yl)-2-propylpentanamide (S119):** white solid (11%). **M.P.** 53–54 ºC. **^{1}H NMR** (400 MHz, $CDCl_3$) δ 5.19 (d, *J* = 8.7 Hz, 1H), 4.14 (t, *J* = 6.4 Hz, 2H), 4.04 (tt, *J* = 7.9, 6.0 Hz, 1H), 3.95 (d, *J* = 7.3 Hz, 2H), 1.95 (tt, *J* = 9.4, 4.3 Hz, 1H), 1.70 (ddt, *J* = 12.2, 7.5, 4.1 Hz, 2H), 1.61 – 1.48 (m, 4H), 1.39 – 1.25 (m, 6H), 1.14 (d, *J* = 6.6 Hz, 3H), 0.89 (t, *J* = 7.0 Hz, 6H), 0.63 – 0.56 (m, 2H), 0.31 (dt, *J* = 6.1, 4.7 Hz, 2H) ppm. **^{13}C NMR** (101 MHz, $CDCl_3$) δ 175.30, 155.32, 72.86, 67.62, 47.97, 44.65, 35.39, 35.26, 33.21, 25.56, 21.34, 20.87, 20.83, 14.13, 9.76, 3.28 ppm. **HRMS** (ESI): calcd. for $C_{17}H_{34}NO_2^+$: 284.2590. found: 284.2585.

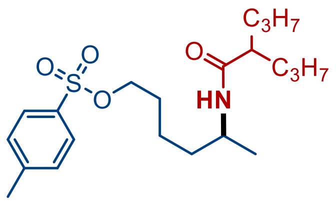

5-(2-Propylpentanamido)hexyl-4-methylbenzenesulfonate (S122): white solid (7%). **M.P.** 68–69 ºC. **^{1}H NMR** (400 MHz, $CDCl_3$) δ 7.84 – 7.72 (m, 2H), 7.34 (d, *J* = 8.1 Hz, 2H), 5.19 (d, *J* = 8.6 Hz, 1H), 4.06 – 3.89 (m, 3H), 2.44 (s, 3H), 1.94 (tt, *J* = 9.5, 4.5 Hz, 1H), 1.67 – 1.51 (m, 4H), 1.37 – 1.23 (m, 10H), 1.08 (d, *J* = 6.6 Hz, 3H), 0.88 (td, *J* = 7.2, 1.4 Hz, 6H) ppm. **^{13}C NMR** (101 MHz, $CDCl_3$) δ 175.27, 144.74, 129.84, 127.86, 70.31, 47.90, 44.55, 36.12, 35.39, 35.25, 29.69, 28.55, 21.96, 21.63, 21.17, 20.83, 20.81, 14.12 ppm. **HRMS** (ESI): calcd. for $C_{21}H_{36}NO_4S^+$: 398.2360. found: 398.2367.

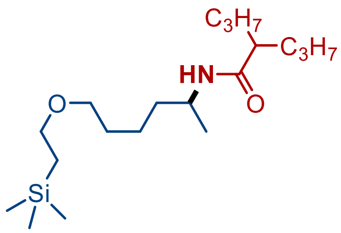

2-Propyl-N-(6-(2-(trimethylsilyl)ethoxy)hexan-2-yl)pentanamide (S123): colorless oil (9%). **^{1}H NMR** (400 MHz, $CDCl_3$) δ 5.16 (d, *J* = 8.6 Hz, 1H), 4.26 – 4.16 (m, 2H), 4.11 (tt, *J* = 6.5, 3.4 Hz, 2H), 4.01 (p, *J* = 6.8 Hz, 1H), 1.94 (dq, *J* = 9.4, 4.6 Hz, 1H), 1.68 (dt, *J* = 13.5, 6.7 Hz, 2H), 1.59 (s, 5H), 1.42 (dq, *J* = 11.6, 6.5 Hz, 4H), 1.33 – 1.22

(m, 6H), 1.12 (d, *J* = 6.6 Hz, 3H), 1.08 – 1.02 (m, 2H), 0.89 (t, *J* = 7.0 Hz, 6H), 0.04 (s, 8H) ppm. **^{13}C NMR** (101 MHz, $CDCl_3$) δ 175.19, 155.39, 67.49, 66.28, 47.97, 44.72, 36.48, 35.41, 35.28, 28.51, 22.31, 21.21, 20.84, 17.55, 14.13, -1.56 ppm. **HRMS** (ESI): calcd. for $C_{19}H_{41}NO_2SiNa^+$: 366.2799. found: 366.2800.

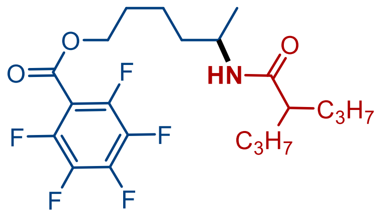

5-(2-Propylpentanamido)hexyl-2,3,4,5,6-pentafluorobenzoate (S125): white solid (10%). **M.P.** 73–74 ºC. **^{1}H NMR** (400 MHz, $CDCl_3$) δ 5.16 (d, *J* = 8.7 Hz, 1H), 4.37 (td, *J* = 6.5, 2.0 Hz, 2H), 4.13 – 3.96 (m, 1H), 2.02 – 1.91 (m, 1H), 1.77 (q, *J* = 6.8 Hz, 2H), 1.66 – 1.55 (m, 4H), 1.47 (dt, *J* = 8.0, 3.9 Hz, 3H), 1.40 – 1.23 (m, 6H), 1.14 (dd, *J* = 6.7, 1.5 Hz, 3H), 0.89 (dd, *J* = 7.3, 5.7 Hz, 6H) ppm. **^{13}C NMR** (101 MHz, $CDCl_3$) δ 175.27, 159.11, 66.66, 47.96, 44.63, 36.43, 35.39, 35.25, 28.19, 22.38, 21.19, 20.83, 14.13, 14.07 ppm. **^{19}F NMR** (376 MHz, $CDCl_3$) δ -138.33 (dp, *J* = 16.4, 5.4 Hz), -148.70 (tt, *J* = 21.1, 4.6 Hz), -160.23 – -160.43 (m) ppm. **HRMS** (ESI): calcd. for $C_{21}H_{29}F_5NO_3^+$: 438.2068. found: 438.2069.

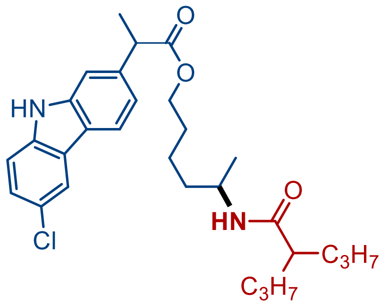

5-(2-Propylpentanamido)hexyl-2-(6-chloro-9H-carbazol-2-yl)propanoate (S128): white solid (7%). **M.P.** 43–44 ºC. ^{1}H NMR (400 MHz, $CDCl_3$) δ 9.96 (s, 1H), 8.02 – 7.90 (m, 2H), 7.43 – 7.29 (m, 3H), 7.12 (dd, *J* = 8.1, 1.5 Hz, 1H), 5.22 (d, *J* = 8.9 Hz, 1H), 4.25 (dt, *J* = 11.6, 6.0 Hz, 1H), 3.99 (ddd, *J* = 19.9, 12.8, 6.0 Hz, 2H), 3.88 (d, *J* = 7.1 Hz, 1H), 2.07 – 1.94 (m, 1H), 1.59 (d, *J* = 5.0 Hz, 5H), 1.42 – 1.23 (m, 12H), 1.01 (d, *J* = 6.6 Hz, 3H), 0.93 (t, *J* = 7.1 Hz, 3H), 0.84 (t, *J* = 6.9 Hz, 3H) ppm. **^{13}C NMR** (101 MHz, $CDCl_3$) δ 175.83, 174.60, 140.90, 138.66, 125.52, 124.17, 121.55, 120.38, 119.79, 119.55, 111.83, 109.30, 64.39, 48.12, 46.30, 45.34, 36.97, 35.49, 35.30, 28.52, 23.33, 21.36, 20.92, 20.88, 19.16, 14.18, 14.09 ppm. **HRMS** (ESI): calcd. for $C_{29}H_{40}ClN_2O_3^+$: 499.2727. found: 499.2724.

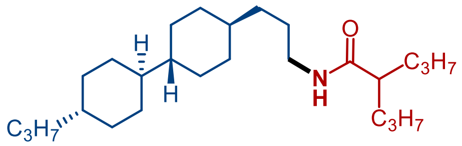

2-Propyl-N-(3-(4'-propyl-[1,1'-bi(cyclohexan)]-4-yl)propyl)pentanamide (95): white solid (37%). **M.P.** 151–152 ºC. **^{1}H NMR** (400 MHz, $CDCl_3$) δ 5.45 (t, *J* = 5.7 Hz, 1H), 3.21 (q, *J* = 6.7 Hz, 2H), 1.97 (dq, *J* = 9.5, 4.7 Hz, 1H), 1.72 (dq, *J* = 17.6, 6.7, 5.9 Hz, 8H), 1.62 – 1.54 (m, 2H), 1.47 (q, *J* = 7.5 Hz, 2H), 1.31 (dddd, *J* = 23.1, 14.2, 10.7, 5.7 Hz, 8H), 1.14 (ddd, *J* = 18.5, 8.7, 3.9 Hz, 6H), 0.98 – 0.78 (m, 19H) ppm. **^{13}C NMR** (101 MHz, $CDCl_3$) δ 175.82, 47.85, 43.46, 43.42, 39.84, 39.59, 37.64, 37.60, 35.33, 34.61,

33.63, 33.57, 30.10, 29.99, 27.22, 20.84, 20.03, 14.41, 14.12 ppm. **HRMS** (ESI): calcd. for $C_{26}H_{50}NO^+$: 392.3887. found: 392.3891.

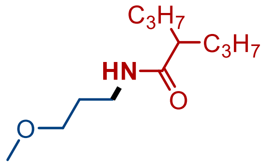

***N*-(3-Methoxypropyl)-2-propylpentanamide (97):** white solid (43%). **M.P.** 66–68 ºC. **^{1}H NMR** (400 MHz, $CDCl_3$) δ 5.98 (s, 1H), 3.46 (t, *J* = 5.8 Hz, 2H), 3.40 – 3.29 (m, 5H), 1.98 (dt, *J* = 9.5, 4.7 Hz, 1H), 1.76 (p, *J* = 6.0 Hz, 2H), 1.64 – 1.52 (m, 2H), 1.39 – 1.23 (m, 6H), 0.88 (t, *J* = 7.1 Hz, 6H) ppm. **^{13}C NMR** (101 MHz, $CDCl_3$) δ 175.88, 71.84, 58.75, 47.71, 37.89, 35.27, 29.19, 24.85, 20.79, 14.10 ppm. **HRMS** (ESI): calcd. for $C_{12}H_{26}NO_2^+$: 216.1958. found: 216.1963.

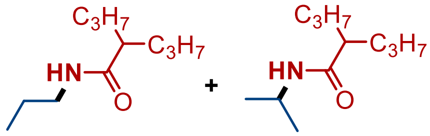

98 **99**

***N*,2-Dipropylpentanamide compound with *N*-isopropyl-2-propylpentanamide (98 : 99 = 4 : 1):** colorless oil (37%). **^{1}H NMR** (400 MHz, $CDCl_3$) δ 5.50 (s, 4H), 5.27 (d, *J* = 8.1 Hz, 1H), 4.10 (dp, *J* = 8.1, 6.5 Hz, 1H), 3.26 – 3.15 (m, 8H), 1.98 (dq, *J* = 9.6, 4.7 Hz, 5H), 1.59 – 1.48 (m, 16H), 1.39 – 1.20 (m, 32H), 1.12 (s, 4H), 0.89 (dt, *J* = 12.3, 7.2 Hz, 44H) ppm. **HRMS** (ESI): calcd. for $C_{11}H_{24}NO^+$: 186.1852. found: 186.1857.

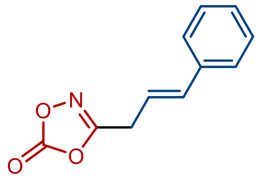

(Z)-3-(3-Phenylallyl)-1,4,2-dioxazol-5-one (100): colorless oil. **^{1}H NMR** (400 MHz, $CDCl_3$) δ 7.38 – 7.24 (m, 5H), 6.69 – 6.57 (m, 1H), 6.12 (dt, *J* = 15.8, 7.0 Hz, 1H), 3.51 (dd, *J* = 7.0, 1.4 Hz, 2H) ppm. **^{13}C NMR** (101 MHz, $CDCl_3$) δ 165.16, 154.04, 136.56, 135.70, 128.77, 128.49, 126.55, 117.07, 28.42.

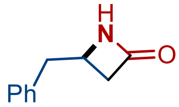

4-Benzylazetidin-2-one (101): white solid (61%). **M.P.** 50–51 ºC. **^{1}H NMR** (400 MHz, $CDCl_3$) δ 7.44 – 7.06 (m, 5H), 6.01 (s, 1H), 3.85 (dtd, *J* = 7.9, 5.4, 2.4 Hz, 1H), 3.07 (ddd, *J* = 14.9, 4.9, 2.2 Hz, 1H), 2.97 (dd, *J* = 13.7, 5.8 Hz, 1H), 2.85 (dd, *J* = 13.7, 7.9 Hz, 1H), 2.69 (dt, *J* = 14.8, 1.8 Hz, 1H) ppm. **^{13}C NMR** (101 MHz, $CDCl_3$) δ 167.53, 137.55, 128.84, 128.75, 126.92, 48.93, 43.32, 41.89 ppm. **HRMS** (ESI): calcd. for $C_{10}H_{12}NO^+$: 162.0913. found: 162.0912.

7. Reaction Optimization

Table S1. Effect of Ligand

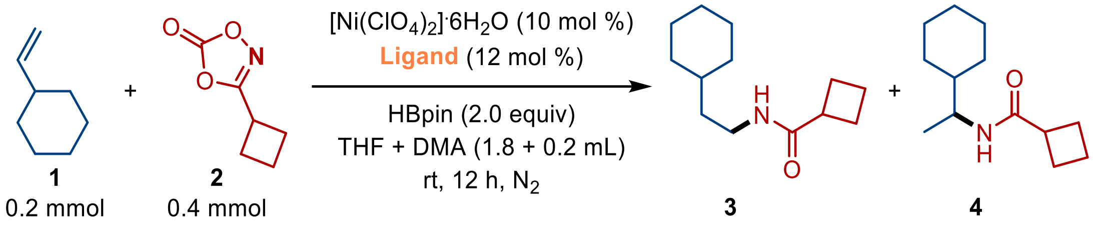

Ligand	**3**[a]	**4**[a]
none	n.d.	n.d.
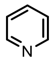	<5%	<5%
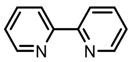	<5%	<5%
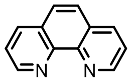	9%	10%
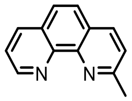	26%	35%
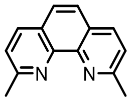	75%	<5%
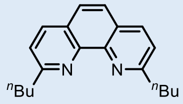	**99%**	**n.d.**
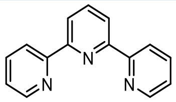	<5%	<5%
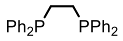	<5%	<5%
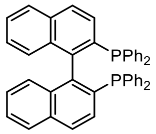	6%	7%
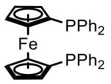	<5%	<5%

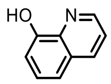	4%	10%
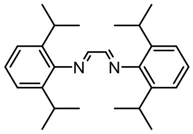	<5%	<5%

[a]NMR yields using CH_2Br_2 as internal standard. Reaction conditions: **1** (0.2 mmol), **2** (0.4 mmol), $[Ni(ClO_4)_2]\cdot 6H_2O$ (10 mol %), ligand (12 mol %), HBpin (2.0 equiv) and THF + DMA (1.8 + 0.2 mL) in N_2 at room temperature for 12 h.

Table S2. Effect of Catalyst

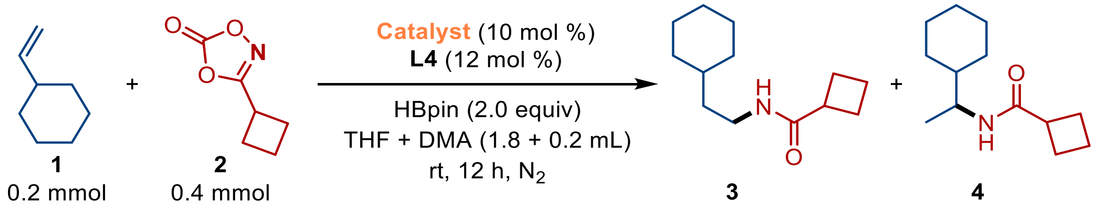

1 0.2 mmol, **2** 0.4 mmol, **3**, **4**

Catalyst	**3**[a]	**4**[a]
**$[Ni(ClO_4)_2]\cdot 6H_2O$**	**99%**	**n.d.**
$Ni(BF_4)_2\cdot 6H_2O$	23%	<5%
$Ni(OAc)_2\cdot 4H_2O$	38%	<5%
$NiF_2\cdot 4H_2O$	53%	<5%
$NiBr_2\cdot 3H_2O$	<5%	n.d.
$Ni(acac)_2\cdot 2H_2O$	40%	<5%
$NiC_2O_4\cdot 2H_2O$	11%	n.d.
$NiCO_3$	29%	<5%
$NiCp_2$	9%	n.d.
$Ni(cod)_2$	16%	n.d.
NiI_2	17%	n.d.
IPrNHC-PPh_3-$NiCl_2$	10%	n.d.
none	n.d.	n.d.

[a]NMR yields using CH_2Br_2 as internal standard. Reaction conditions: **1** (0.2 mmol), **2** (0.4 mmol), catalyst (10 mol %), **L4** (12 mol %), HBpin (2.0 equiv) and THF + DMA (1.8 + 0.2 mL) in N_2 at room temperature for 12 h.

Table S3. Effect of Solvent

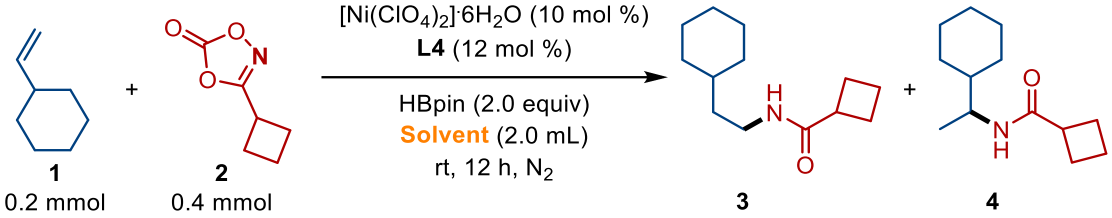

Solvent	3[a]	4[a]
MeOH	35%	n.d.
MeCN	55%	<5%
DCE	90%	<5%
Toluene	44%	<5%
Acetone	21%	n.d.
DMF	19%	n.d.
DMA	56%	<5%
Dioxane	83%	<5%
THF	95%	<5%
DMA + THF (1.8 + 0.2 mL)	60%	<5%
**THF + DMA (1.8 + 0.2 mL)**	**99%**	**n.d.**

[a]NMR yields using CH_2Br_2 as internal standard. Reaction conditions: **1** (0.2 mmol), **2** (0.4 mmol), $[Ni(ClO_4)_2]·6H_2O$ (10 mol %), **L4** (12 mol %), HBpin (2.0 equiv) and solvent (2.0 mL) in N_2 at room temperature for 12 h.

Table S4. Effect of Hydride Reagents

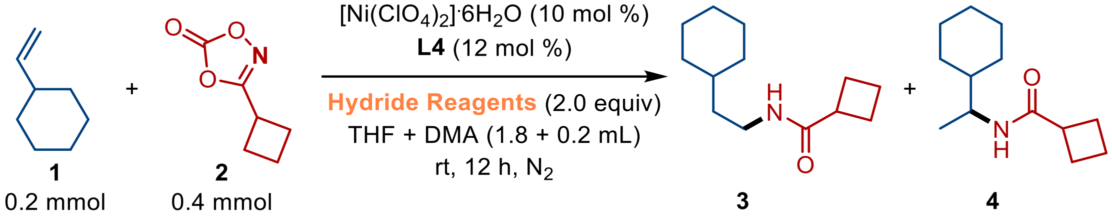

Hydride Reagents	**3**[a]	**4**[a]
none	n.d.	n.d.
MeOH	n.d.	n.d.
$(EtO)_2SiMeH$	12%	n.d.
Et_3SiH	<5%	n.d.
PMHS	<5%	n.d.
Ph_2SiH_2	17%	n.d.
**HBpin**	**99%**	**n.d.**

[a]NMR yields using CH_2Br_2 as internal standard. Reaction conditions: **1** (0.2 mmol), **2** (0.4 mmol), $[Ni(ClO_4)_2]·6H_2O$ (10 mol %), **L4** (12 mol %), hydride reagent (2.0 equiv) and THF + DMA (1.8 + 0.2 mL) in N_2 at room temperature for 12 h.

Table S5. Effect of Temperature

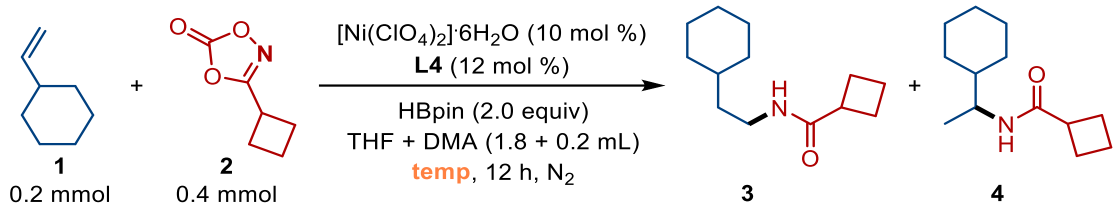

Temperature (°C)	**3**[a]	**4**[a]
**rt**	**99%**	**n.d.**
0	67%	<5%
40	86%	<5%
60	81%	<5%
80	43%	<5%

[a]NMR yields using CH_2Br_2 as internal standard. Reaction conditions: **1** (0.2 mmol), **2** (0.4 mmol), $[Ni(ClO_4)_2]\cdot 6H_2O$ (10 mol %), **L4** (12 mol %), HBpin (2.0 equiv) and THF + DMA (1.8 + 0.2 mL) in N_2 at 0–80 ºC for 12 h.

Table S6. Effect of Additives

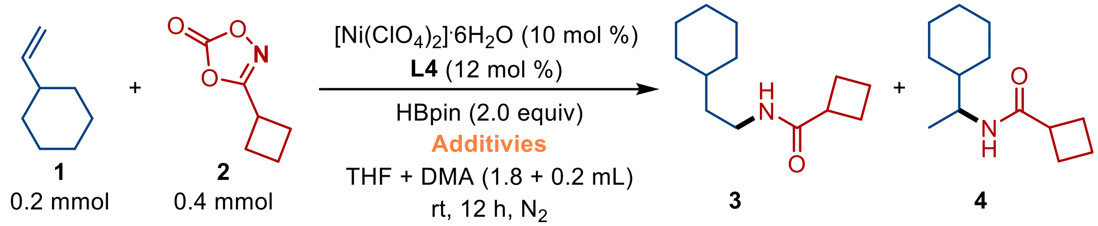

Additivies	**3**[a]	**4**[a]
molecular sieve (4 A, 100 mg)	84%	n.d.
proton sponge (2.0 equiv)	50%	n.d.
H_2O (0.6 equiv)	71%	n.d.
**none**	**99%**	**n.d.**

[a]NMR yields using CH_2Br_2 as internal standard. Reaction conditions: **1** (0.2 mmol), **2** (0.4 mmol), $[Ni(ClO_4)_2]\cdot 6H_2O$ (10 mol %), **L4** (12 mol %), HBpin (2.0 equiv), additives and THF + DMA (1.8 + 0.2 mL) in N_2 at room temperature for 12 h.

8. Mechanistic Studies

8.1 Reactions in the presence of radical scavengers

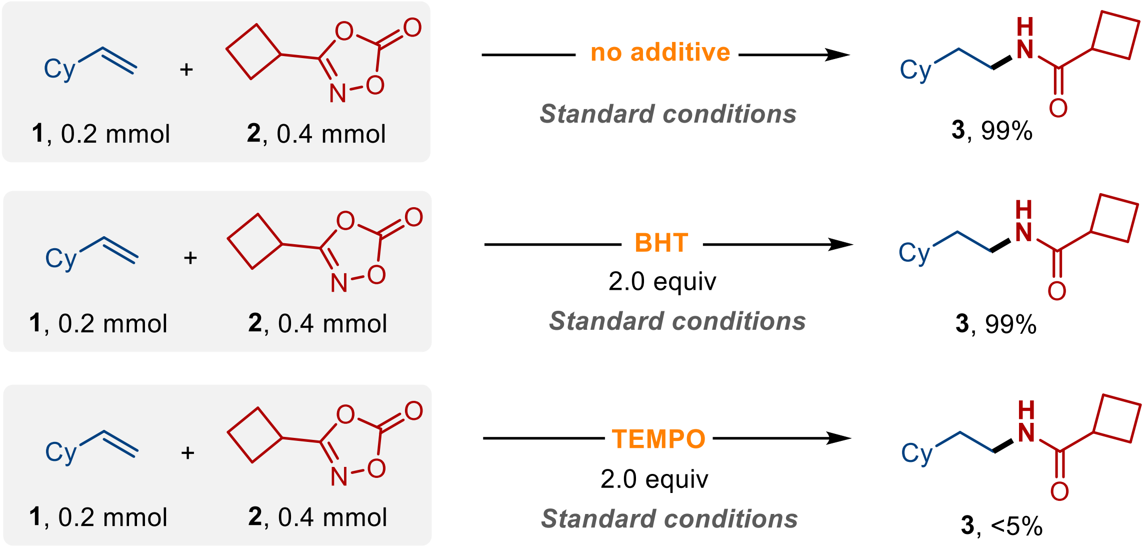

To probe the involvement of carboradical intermediates, radical inhibitors such as BHT (2,6-di-tert-butyl-4-methylphenol) and TEMPO (2,2,6,6-tetramethylpiperidyl-1-oxyl) were added to the reaction mixtures. Compared to the "**1** + **2**" reaction under the standard condition, the addition of BHT exerted negligible effect to the formation of **3** (99%), suggesting that reaction pathways involving radical formation is untenable. Although the **3** formation was completely suppressed (<5%) when the reaction was performed in the presence of TEMPO (2.0 equiv). This could be attributed to the oxidation of the low valent nickel active intermediate by the TEMPO, resulting in the deactivation of the catalysts.

8.2 Hydroboration/amidation

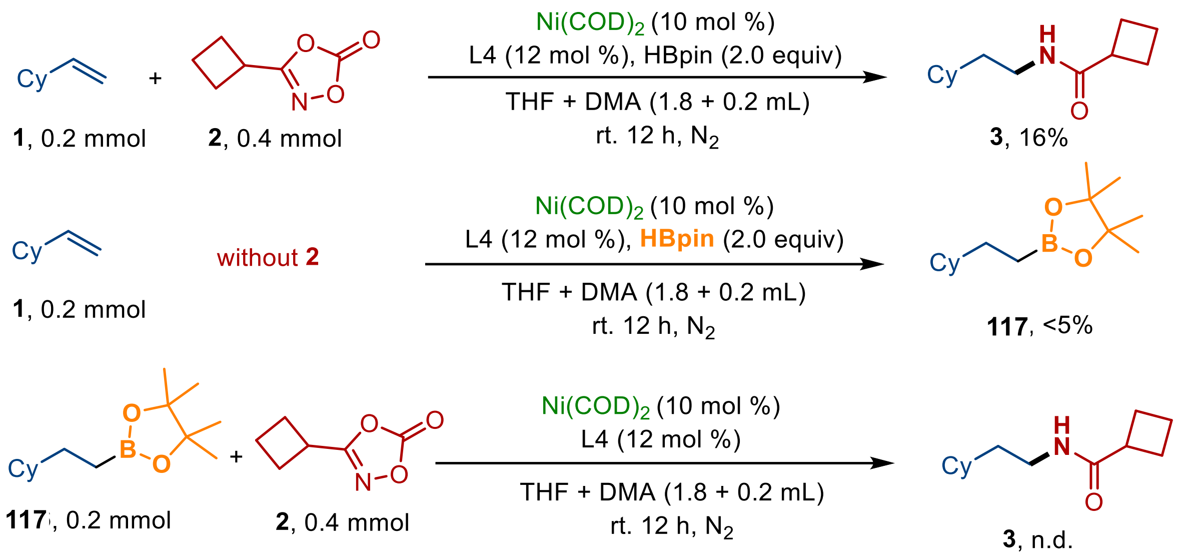

Apart from [Ni(ClO$_4$)$_2$]·6H$_2$O, Ni(COD)$_2$ can also serve as the catalyst to give the hydroamidation product **3** with 16% yield. To examine the possibility of low valent nickel catalyzing the hydroboration/ amidation process, Ni(COD)$_2$ was also subjected to the same sets of mechanistic studies as undertaken for [Ni(ClO$_4$)$_2$]·6H$_2$O. As shown above, Ni(COD)$_2$ is in effective catalyst for alkene hydroboration by HBpin and the cross-coupling reaction of vinylboronate **117**with **2**. This result further excludes the mechanism involving a sequential hydroboration/ amidation pathway.

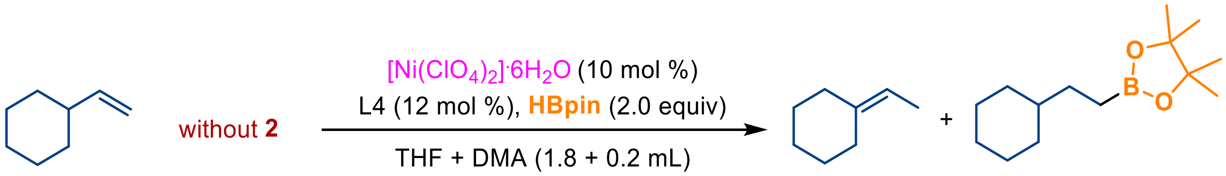

1, 0.2 mmol **116**, 55%. **117**, n.d.

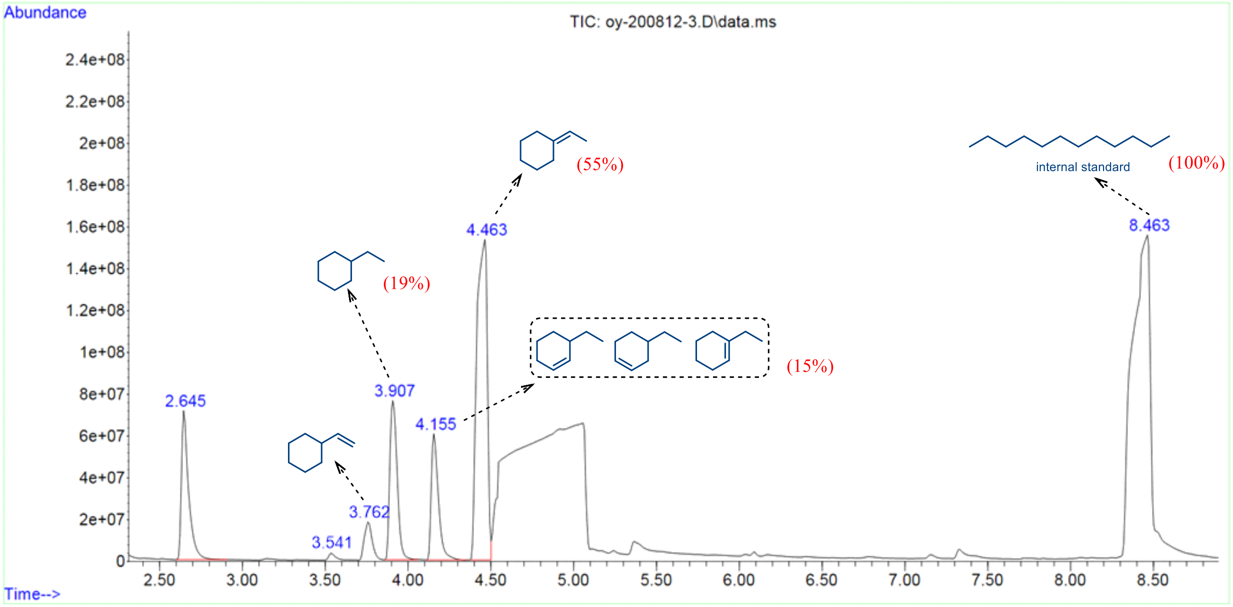

Without dioxazolone, the alkene was found to undergo rearrangement under the Ni-catalyzed conditions. GC-MS analysis of the crude mixtures, using dodecane as internal standard was performed for the reaction of **1** with HBpin. It was found that only a minute quantity of the starting alkene **1** remains; most of the alkene was either hydrogenated or transformed into ethylidenecyclohexane **116** (55%) plus other isomeric alkenes as minor products. The formation of **116** presumably via double bond migration suggested that the hydride insertion/elimination pathway of the organonickel intermediates should be reversible.

8.3 Deuterium labeling studies

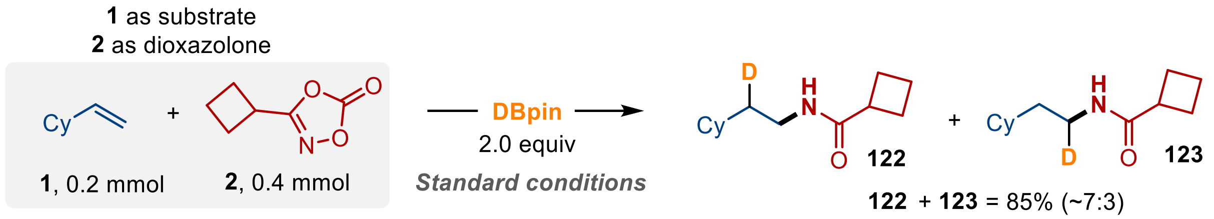

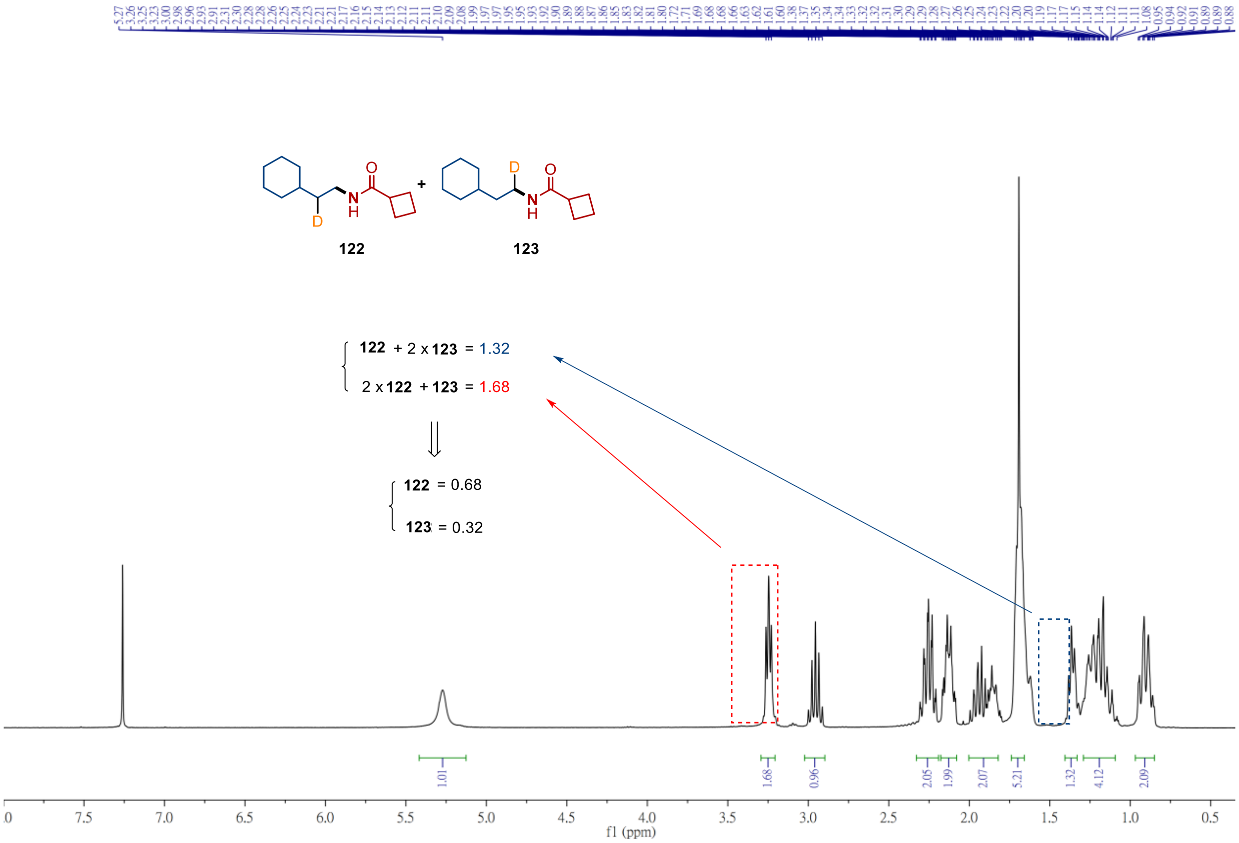

The ^{1}H NMR of **122** with **123** shows a deviation in peak integration at δ 1.37 (t, J = 7.3 Hz, 1.32H) and δ 3.25 (t, J = 6.6 Hz, 1.68H) compared to non-deuterated products, resulting from a deuterium incorporation at the *α* and *β* position. Based on the value of the integration, the ratio of **122** to **123** is calculated to be approximately 7 : 3.

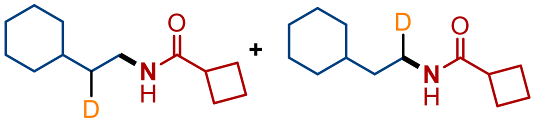

122 **123**

***N*-(2-Cyclohexylethyl-1-d)cyclobutene-carboxamide compound with *N*-(2-cyclohexylethyl-2-d)cyclobutene-carboxamide (122 : 123 = 7 : 3):** white solid (85%). **M.P.** 61–63 ºC.

^{1}H NMR (400 MHz, $CDCl_3$) δ 5.27 (s, 1H), 3.25 (t, *J* = 6.6 Hz, 1.68H), 2.96 (p, *J* = 8.6 Hz, 1H), 2.26 (pd, *J* = 9.1, 2.5 Hz, 2H), 2.13 (dtd, *J* = 12.0, 8.5, 3.1 Hz, 2H), 2.00 – 1.82 (m, 2H), 1.68 (q, *J* = 5.8 Hz, 5H), 1.37 (t, *J* = 7.3 Hz, 1.32H), 1.29 – 1.09 (m, 4H), 0.90 (qd, *J* = 10.8, 9.9, 4.7 Hz, 2H) ppm. **HRMS** (ESI): calcd. for $C_{13}H_{23}DNO^+$: 211.1915. found: 211.1919.

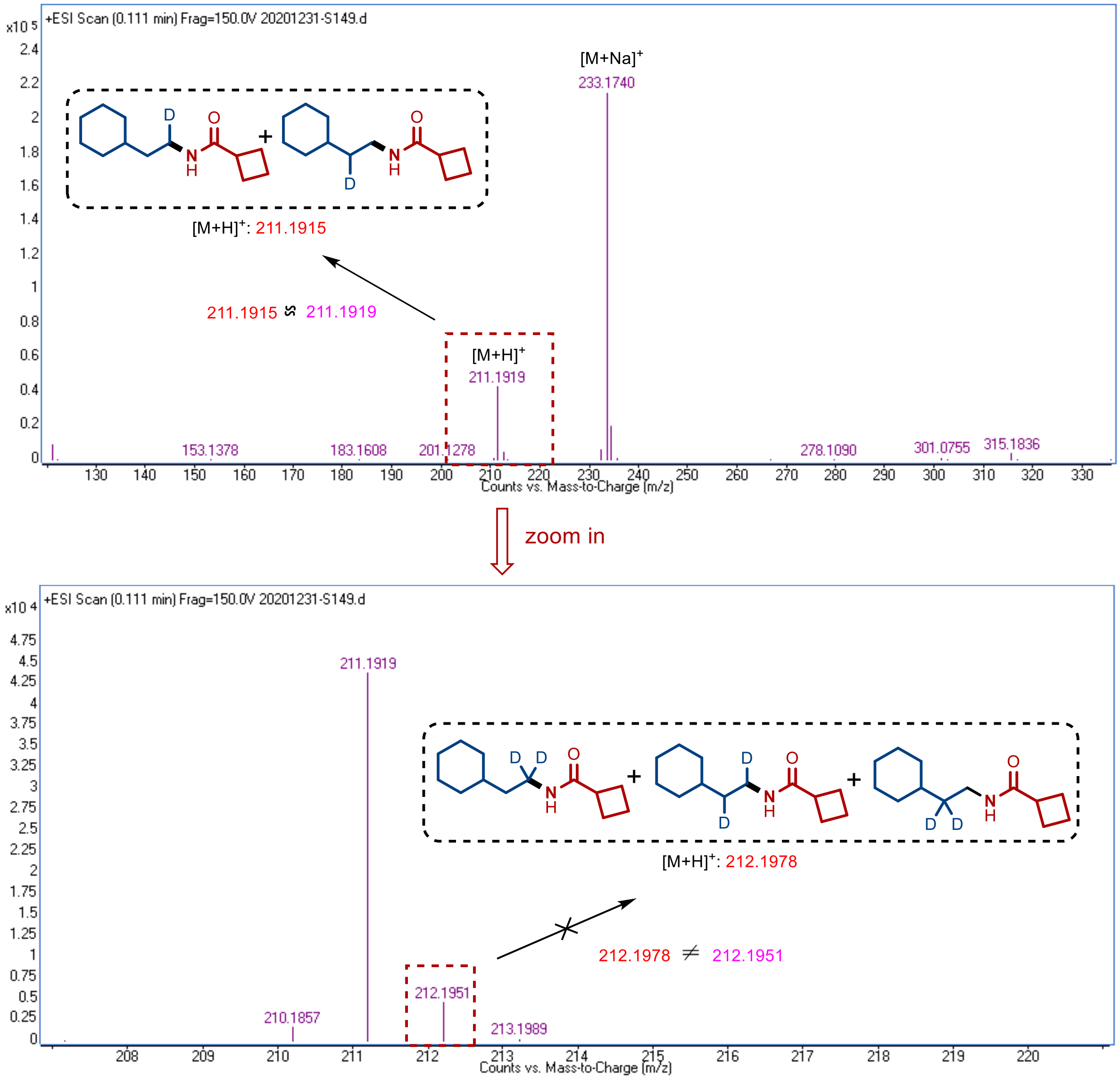

The HRMS of **122** with **123** shows a significant peak at $[M+H]^+$ = 211.1919, resulting from $C_{13}H_{23}DNO^+$: 211.1915. Notably, the peaks at *m/z* = 212.1978 or higher, due to any products containing more than one deuterium, are not detected. This result confirmed that all the product concerned in this isotope study are monodeuterated.

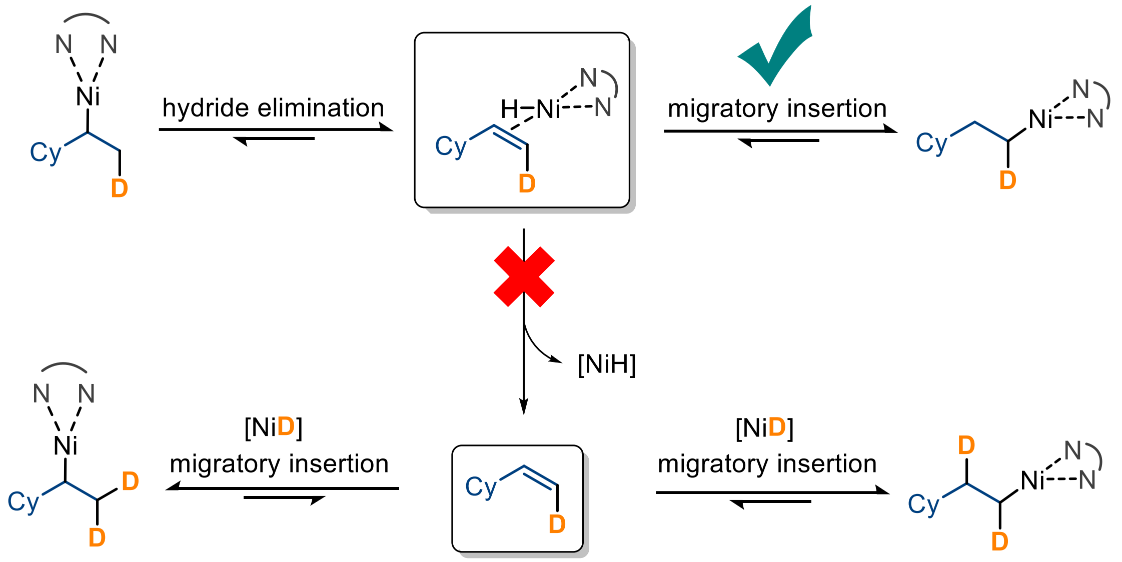

On the basis of the above findings, the [Ni-H] formed from the *b*-hydride elimination should undergo facile migratory insertion with the complexed d-alkenes. It should be noted that the double-deuterated amide products formation requires a secondary reaction of the d-alkenes with another [Ni-D] complex. The lack of double-deuterated amide products suggests that the coordinated alkene does not dissolve to give free d-alkenes prior to the olefin insertion into the [Ni-H] species.[9]

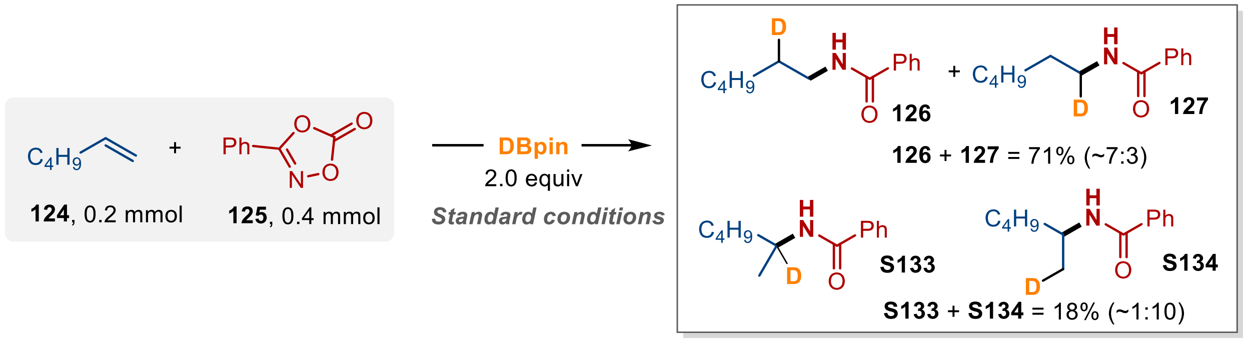

Along with the formation of anti-Markovnikov products, branched (Markovnikov) products (18% yield) were also observed for reaction of **124** and **125**. Similar to the linear products, the branched products were also found to exist in two deuterium-labelled isomers (**S133** : **S134** ~ 1 : 10). The occurrence of the deuterium at both the linear and branched positions strongly implies a reversible hydride elimination/insertion of the organonickel intermediates.

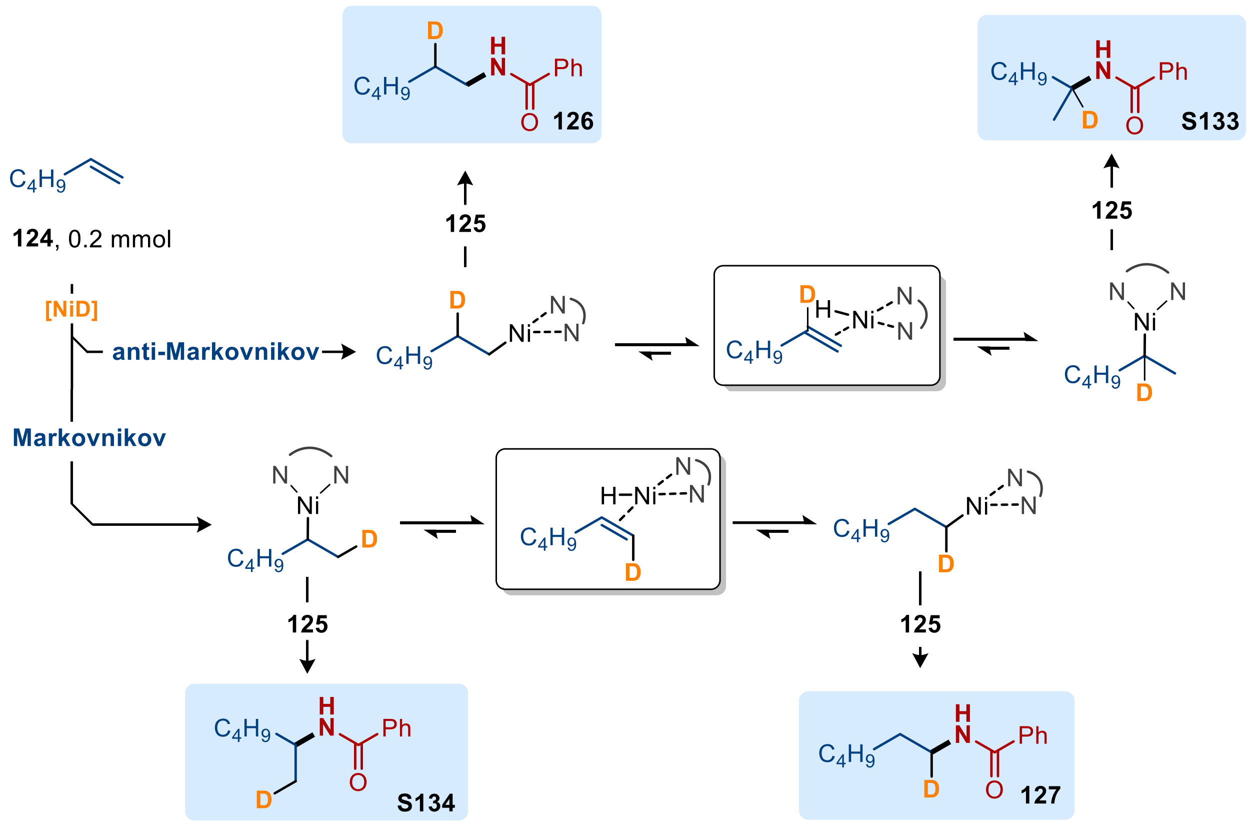

The two Markovnikov products **S133** and **S134** are generated by reaction between **125** and two Markovnikov alkyl-nickel intermediate. Plausibly, the similar pathway is largely suppressed when the alkene is **1** because of its larger steric effect compared to **124**.

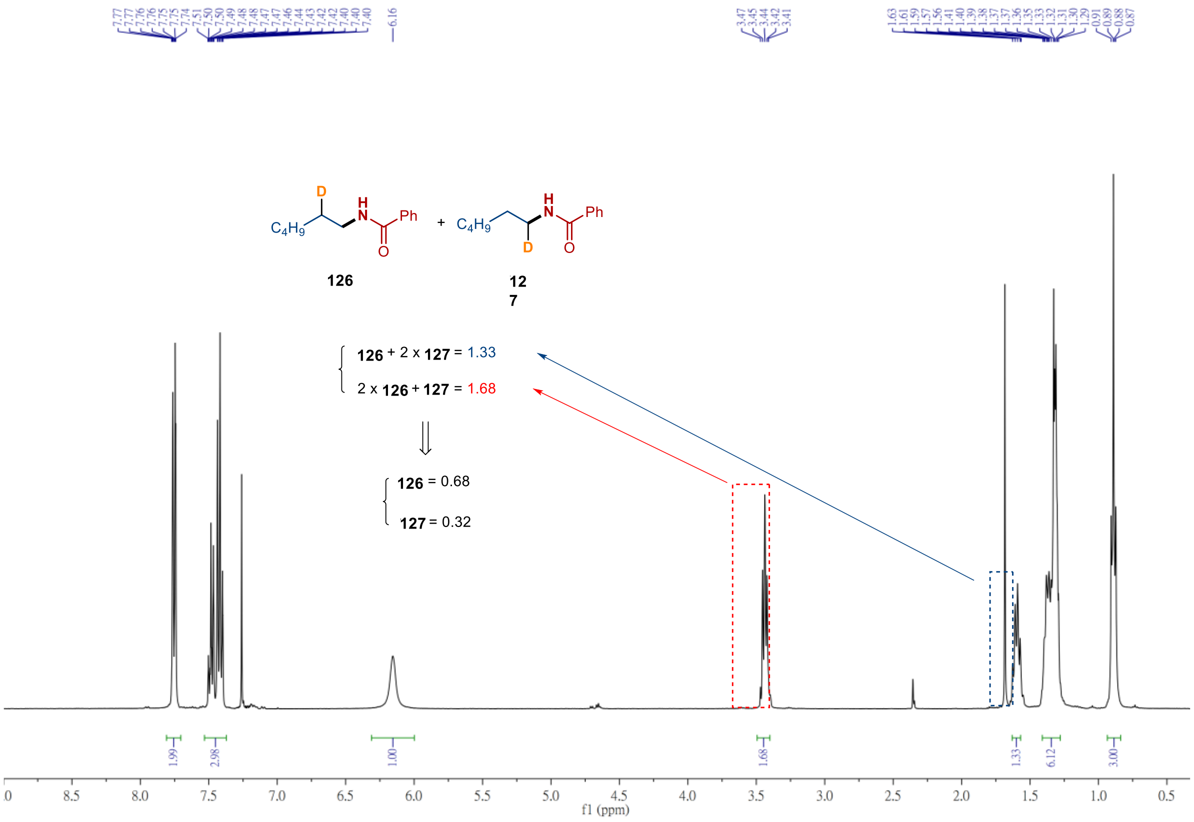

The ^{1}H NMR of **126** with **127** shows a deviation in peak integration at δ 1.60 (q, J = 7.3 Hz, 1.33H) and δ 3.45 (q, J = 6.4 Hz, 1.68H) compared to non-deuterated products, resulting from a deuterium incorporation at the α and β position. Based on the value of the integration, the ratio of **126** to **127** is calculated to be approximately 7 : 3.

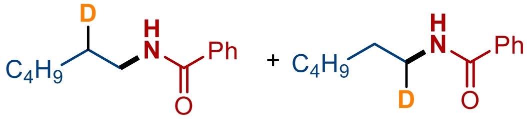

126 **127**

***N*-(Hexyl-1-d)benzamide compound with *N*-(hexyl-2-d)benzamide (126 : 127 = 7 : 3):** white solid (71%). **M.P.** 73–75 ºC. **^{1}H NMR** (400 MHz, $CDCl_3$) δ 7.81 – 7.70 (m, 2H), 7.53 – 7.37 (m, 3H), 6.16 (s, 1H), 3.45 (q, J = 6.4 Hz, 1.68H), 1.60 (q, J = 7.3 Hz, 1.33H), 1.40 – 1.28 (m, 6H), 0.94 – 0.84 (m, 3H) ppm. **HRMS** (ESI): calcd. for $C_{13}H_{19}DNO^+$: 207.1602. found: 207.1606.

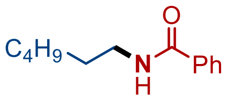

***N*-Hexylbenzamide (S84):** white solid (77%). **M.P.** 74–75 ºC. **^{1}H NMR** (400 MHz, $CDCl_3$) δ 7.85 – 7.71 (m, 2H), 7.56 – 7.36 (m, 3H), 6.33 (s, 1H), 3.42 (td, J = 7.3, 5.7 Hz, 2H), 1.64 – 1.53 (m, 2H), 1.40 – 1.26 (m, 6H), 0.95 – 0.84 (m, 3H) ppm. **^{13}C NMR** (101 MHz, $CDCl_3$) δ 167.56, 134.89, 131.26, 128.50, 126.86, 40.13, 31.52, 29.64, 26.68, 22.57, 14.02 ppm. **HRMS** (ESI): calcd. for $C_{13}H_{20}NO^+$: 206.1539. found: 206.1544.

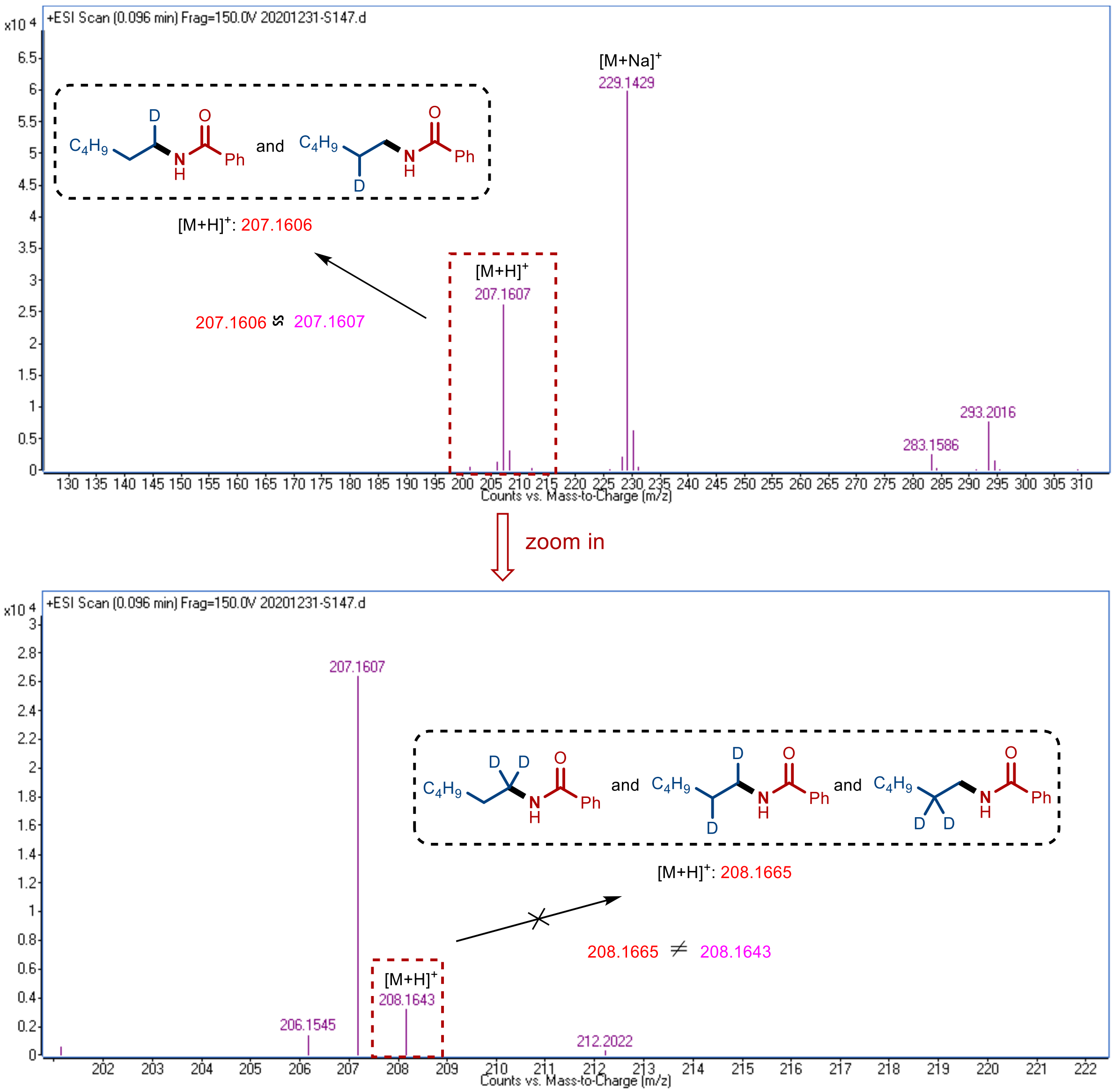

The HRMS of **126** with **127** shows a significant peak at $[M+H]^+ = 207.1607$, resulting from $C_{13}H_{23}DNO^+$: 207.1602. Notably, the peaks at *m/z* = 208.1665 or higher, due to any products containing more than one deuterium, are not detected. This result confirmed that all the product concerned in this isotope study are monodeuterated.

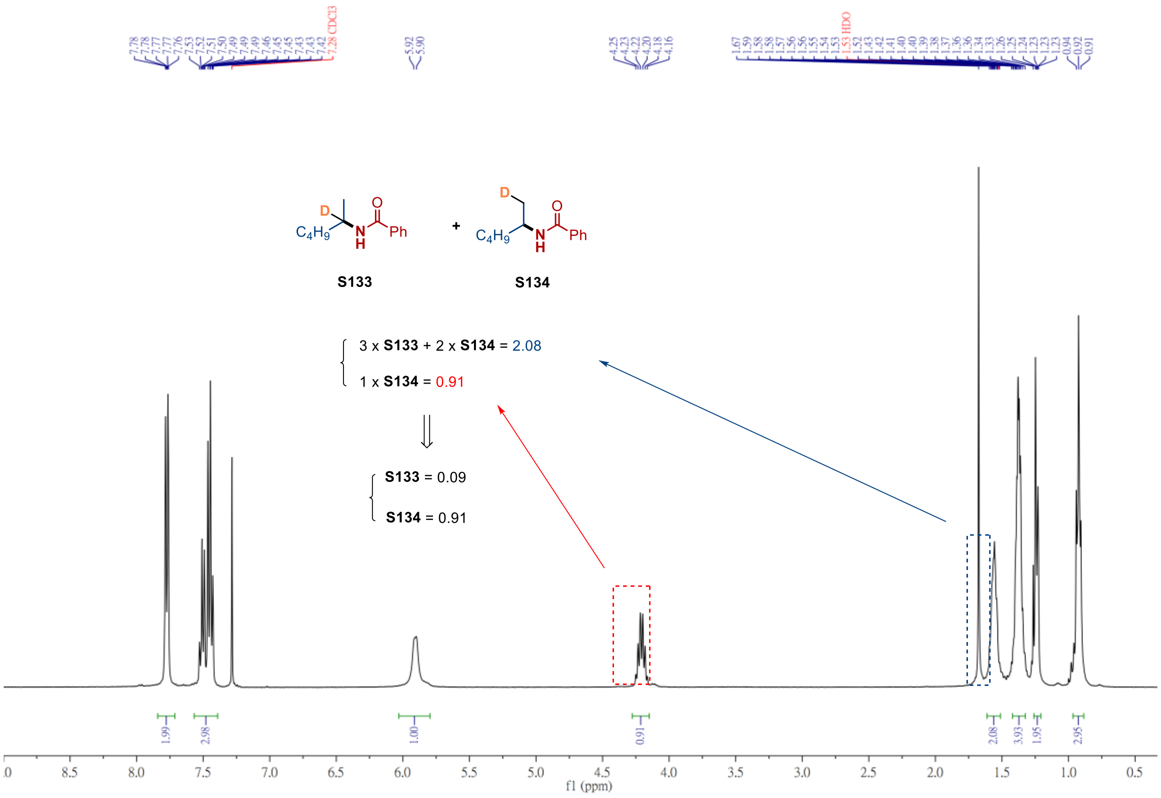

The ^{1}H NMR of **S133** with **S134** shows a deviation in peak integration at δ 1.56 (pd, *J* = 8.4, 7.7, 5.0 Hz, 2.08H) and 4.21 (h, *J* = 6.8 Hz, 0.91H) compared to non-deuterated products, resulting from a deuterium incorporation at the *α* and *β* position. Based on the value of the integration, the ratio of **S133** to **S134** is calculated to be approximately 1 : 10.

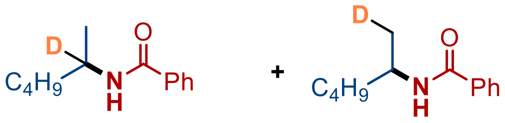

S133 **S134**

***N*-(Hexan-2-yl-1-d)benzamide compound with *N*-(hexan-2-yl-2-d)benzamide (S133 : S134 = 1 : 10):** white solid (18%). **M.P.** 82–84 °C. **^{1}H NMR** (400 MHz, $CDCl_3$) δ 7.84 – 7.71 (m, 2H), 7.57 – 7.39 (m, 3H), 5.91 (d, *J* = 8.3 Hz, 1H), 4.21 (h, *J* = 6.8 Hz, 0.91H), 1.56 (pd, *J* = 8.4, 7.7, 5.0 Hz, 2.08H), 1.45 – 1.31 (m, 4H), 1.28 – 1.20 (m, 2H), 0.92 (t, *J* = 6.9 Hz, 3H) ppm. **HRMS** (ESI): calcd. for $C_{13}H_{19}DNO^+$: 207.1602. found: 207.1606.

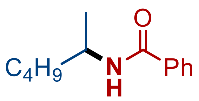

***N*-(Hexan-2-yl)benzamide (S132):** white solid (18%). **M.P.** 83–84 °C. **^{1}H NMR** (400 MHz, $CDCl_3$) δ 7.81 – 7.74 (m, 2H), 7.55 – 7.46 (m, 1H), 7.45 (dd, *J* = 8.2, 6.5 Hz, 2H), 5.91 (d, *J* = 7.3 Hz, 1H), 4.21 (dq, *J* = 8.4, 6.6 Hz, 1H), 1.68 (s, 1H), 1.62 – 1.52 (m, 1H), 1.38 (hd, *J* = 6.2, 5.7, 2.9 Hz, 4H), 1.26 (d, *J* = 6.6 Hz, 3H), 1.02 – 0.88 (m,

4H) ppm. **^{13}C NMR** (101 MHz, $CDCl_3$) δ 166.82, 135.10, 131.25, 128.53, 126.79, 45.76, 36.81, 28.26, 22.61, 21.08, 14.03 ppm. **HRMS** (ESI): calcd. for $C_{13}H_{20}NO^+$: 206.1539. found: 206.1546.

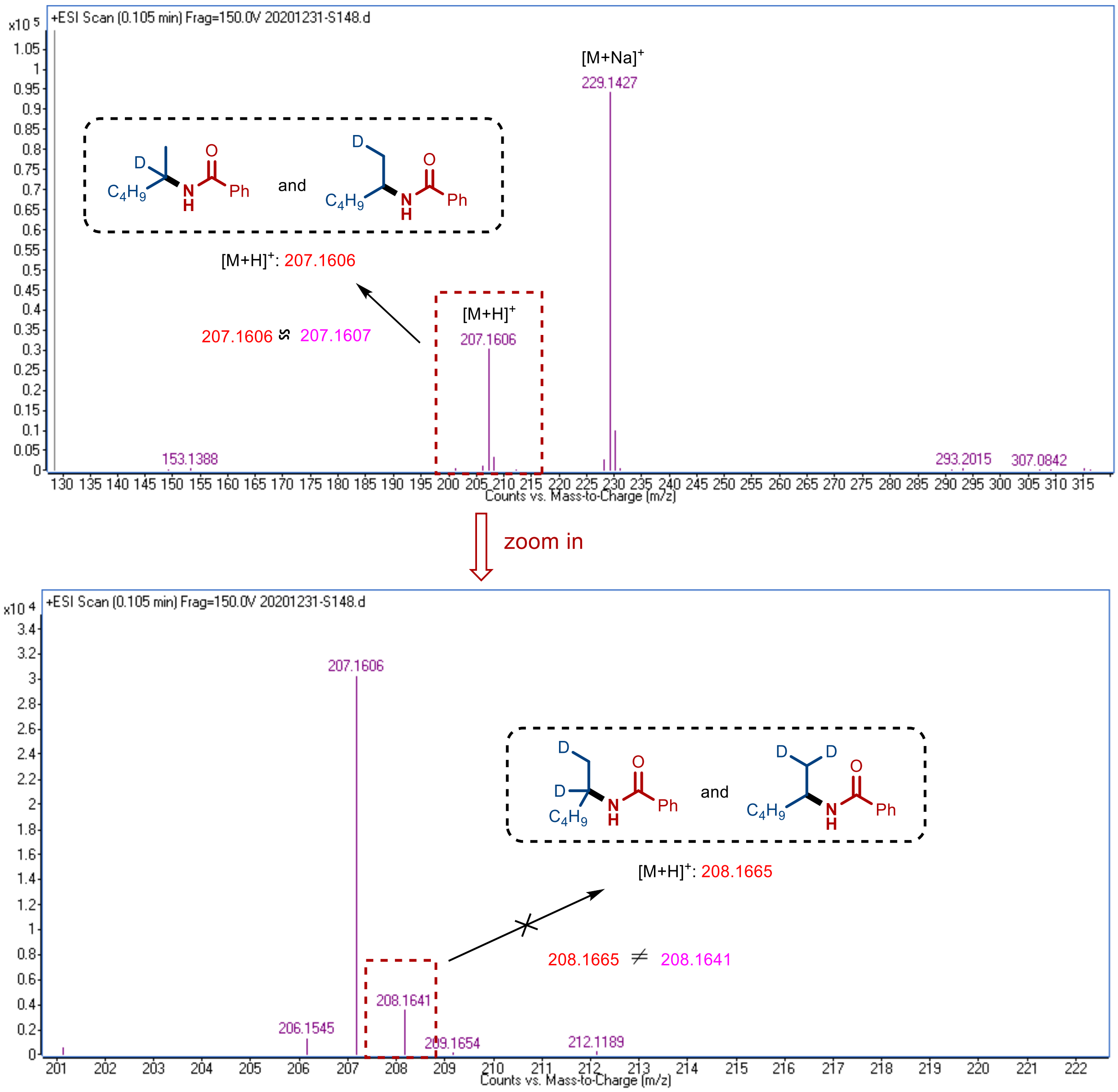

The HRMS of **S133** with **S134** shows a significant peak at $[M+H]^+ = 207.1606$, resulting from $C_{13}H_{23}DNO^+$: 207.1602. Notably, the peaks at *m/z* = 208.1665 or higher, due to any products containing more than one deuterium, are not detected. This result confirmed that all the product concerned in this isotope study are monodeuterated.

9. Reference

1. Han, J.; Qin, Y.; Zhao, D. *ACS Catal*. **2019**, *9*, 6020.
2. Wang, X.; Wu, Y. *Chem. Commun*. **2018**, *54*, 1877.
3. Kambara, T; Tomioka, K. *J. Org. Chem*. **1999**, *64*, 9282.
4. Lee, S. K.; Heo, S.; Lee, J. G.; Kang, K. T.; Kumazawa, K.; Nishida, K.; Shimbo, Y.; Takanishi, Y.; Watanabe, J.; Doi, T.; Takahashi, T.; Takezoe, H. *J. Am. Chem. Soc*. **2005**, *127*, 11085.
5. Itoh, T.; Matsueda, T.; Shimizu, Y.; Kanai, M. *Chem. Eur. J*. **2015**, *21*, 15955.
6. Schneider, C.; Broda, E.; Snieckus, V. *Org. Lett*. **2011**, *13*, 3588.
7. Wang, Y.; Guan, R.; Sivaguru, P.; Cong, X.; Bi, X. *Org. Lett*. **2019**, *21*, 4035.
8. Yu, H. C.; Islam, S. M.; Mankad. N. P.; *ACS Catal*. **2020**, *10*, 3670.
9. Asnet, P.; Dhungana, R. K.; Thapa, S.; Shrestha, B.; KC, S.; Sears, J. M.; Giri, R. *J. Am. Chem. Soc*. **2018**, *140*, 7782.
10. Chai, J.-D.; Head-Gordon, M. *Phys. Chem. Chem. Phys.* **2008**, *10*, 6615.
11. a) Wedig, U.; Dolg, M.; Stoll, H. Preuss, *Quantum Chemistry: The Challenge of Transition Metals and Coordination Chemistry* (Ed. A. Veillard, Reidel, Dordrecht), **1986**, 79; b) Dolg, M.; Wedig, U.; Stoll, H.; Preuss, H. *J. Chem. Phys.*, **1987**, *86*, 866.
12. a) Ditchfield, R.; Hehre, W. J.; Pople, J. A.*J. Chem. Phys.*, **1971**, *54*, 724; b) Hehre, W. J.; Ditchfield, R.; Pople, J. A. *J. Chem. Phys.*, **1972**, *56*, 2257; c) Hariharan, P. C.; Pople, J. A. *Theor. Chem. Acc.*, **1973**, *28*, 213; d) Hariharan, P. C.; Pople, J. A. *Mol. Phys.*, **1974**, *27*, 209.
13. Frisch, M. J.; Trucks, G. W.; Schlegel, H. B.; Scuseria, G. E.; Robb, M. A.; Cheeseman, J. R.; Scalmani, G.; Barone, V.; Petersson, G. A.; Nakatsuji, H.; Li, X.; Caricato, M.; Marenich, A.; Bloino, J.; Janesko, B. G.; Gomperts, R.; Mennucci, B.; Hratchian, H. P.; Ortiz, J. V.; Izmaylov, A. F.; Sonnenberg, J. L.; Williams-Young, D.; Ding, F.; Lipparini, F.; Egidi, F.; Goings, J.; Peng, B.; Petrone, A.; Henderson, T.; Ranasinghe, D.; Zakrzewski, V. G.; Gao, J.; Rega, N.; Zheng, G.; Liang, W.; Hada, M.; Ehara, M.; Toyota, K.; Fukuda, R.; Hasegawa, J.; Ishida, M.; Nakajima, T.; Honda, Y.; Kitao, O.; Nakai, H.; Vreven, T.; Throssell, K.; Montgomery, J. A.; Peralta, J. E.; Ogliaro, F.; Bearpark, M.; Heyd, J. J.; Brothers, E.; Kudin, K. N.; Staroverov, V. N.; Keith, T.; Kobayashi, R.; Normand, J.; Raghavachari, K.; Rendell, A.; Burant, J. C.; Iyengar, S. S.; Tomasi, J.; Cossi, M.; Millam, J. M.; Klene, M.; Adamo, C.; Cammi, R.; Ochterski, J. W.; Martin, R. L.; Morokuma, K.; Farkas, O.; Foresman, D. J. Fox, Gaussian09, Revision D.01. Gaussian, Inc.: Wallingford, CT, **2009**.
14. a) Fukui, K. *Acc. Chem. Res.* **1981**, *14*, 363; b) Hratchian, H. P.; Schlegel, H. B. *Theory and Applications of Computational Chemistry: The First 40 Years* (Ed. C. E. Dykstra, G. Frenking, K. S. Kim, and G. Scuseria), Elsevier, Amsterdam, **2005**, 195.
15. Weigend, F.; Ahlrichs, R.*Phys. Chem. Chem. Phys.*, **2005**, *7*, 3297.
16. Marenich, A. V.; Cramer, C.; Truhlar, D. G. *J. Phys. Chem. B*, **2009**, *113*, 6378.
