# Supplementary material for: NiH-catalyzed anti-Markovnikov hydroamidation of unactivated alkenes with 1,4,2-dioxazol-5-ones for the direct synthesis of N-alkyl amides
Source: Commun Chem. 2022 Dec 22;5:176. doi: 10.1038/s42004-022-00791-4 (PMC9814879; doi:10.1038/s42004-022-00791-4)
Supplement: Supplementary file 3 — Description of Additional Supplementary Files [file 42004_2022_791_MOESM3_ESM.pdf]

Description of Additional Supplementary Files

File name: Supplementary Data 1

Description: X-Ray Crystallographic Data for **40**

File name: Supplementary Data 2

Description: DFT calculations

File name: Supplementary Data 3

Description: NMR Spectra
