# Supplementary material for: NiH-catalyzed anti-Markovnikov hydroamidation of unactivated alkenes with 1,4,2-dioxazol-5-ones for the direct synthesis of N-alkyl amides
Source: Commun Chem. 2022 Dec 22;5:176. doi: 10.1038/s42004-022-00791-4 (PMC9814879; doi:10.1038/s42004-022-00791-4)
Supplement: Supplementary file 4 — Supplementary Data 1 [file 42004_2022_791_MOESM4_ESM.pdf]

X-Ray Crystallographic data for 40

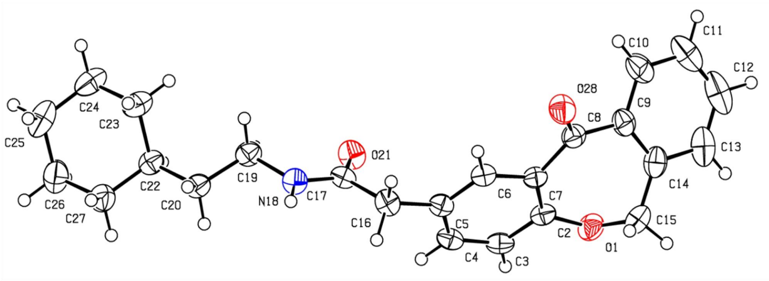

N-(2-Cyclohexylethyl)-2-(11-oxo-6,11-dihydrodibenzo[b,e]oxepin-2-yl)acetamide **(40)**

(CCDC: 2052631)

Crystal structure determination of 40

Crystal Data. $C_{24}H_{27}NO_3$, M_r = 377.46, orthorhombic, *Pbca* (No. 61), a = 9.4287(3) Å, b = 11.8645(6) Å, c = 35.4924(9) Å, *a* = *b* = *g* = 90˚, *V* = 3970.4(2) Å^3, *T* = 180(2) K, *Z* = 8, *Z'* = 1, *m*(CuK$_a$) = 0.657, 21233 reflections measured, 4086 unique (R_{int} = 0.0224) which were used in all calculations. The final wR_2 was 0.1096 (all data) and R_1 was 0.0426 (I > 2(I)).

Table S7. Crystal data and structure refinement for 40

Compound 40			
Formula	$C_{24}H_{27}NO_3$	*Z'*	1
$D_{calc.}$/ g cm^{-3}	1.263	Wavelength/Å	1.54184
m/mm^{-1}	0.657	Radiation type	CuK$_a$
Formula Weight	377.46	Q_{min}/˚	5.312
Colour	None None None	Q_{max}/˚	77.044
Shape	n/a	Measured Refl.	21233
Size/mm^3	n/a×n/a×n/a	Independent Refl.	4086
T/K	180(2)	Reflections with I > 2(I)	3933
Crystal System	orthorhombic	R_{int}	0.0224
Space Group	*Pbca*	Parameters	254
a/Å	9.4287(3)	Restraints	0
b/Å	11.8645(6)	Largest Peak	0.354
c/Å	35.4924(9)	Deepest Hole	-0.171
a/˚	90	GooF	1.044
b/˚	90	wR_2 (all data)	0.1096
g/˚	90	wR_2	0.1087

V/Å^3	3970.4(2)	R_1 (all data)	0.0437
Z	8	R_1	0.0426

Table S8. Fractional Atomic Coordinates (×10^4) and Equivalent Isotropic Displacement Parameters (Å^2×10^3) for 40. U_{eq} is defined as 1/3 of the trace of the orthogonalised U_{ij}.

Atom	x	y	z	U_{eq}
O21	232.1(9)	2261.4(9)	4831.8(3)	42.9(2)
O1	-41.4(11)	-1825.9(8)	3718.4(3)	45.2(2)
N18	2209.3(10)	2884.3(9)	5122.4(3)	36.0(2)
C7	1152.7(12)	19.4(10)	3745.1(3)	32.1(3)
C17	1528.6(12)	2251.0(10)	4870.7(3)	31.6(3)
O28	1844.1(12)	1168.1(8)	3236.4(3)	50.6(3)
C27	1778.4(16)	4608.1(13)	6390.8(4)	48.5(3)
C6	1792.9(12)	796.2(10)	3992.8(3)	32.5(3)
C16	2489.0(12)	1521.3(11)	4629.6(3)	37.2(3)
C4	1003.8(12)	-224.0(11)	4529.6(4)	36.5(3)
C5	1749.0(12)	691.5(10)	4378.6(3)	33.0(3)
C22	1188.3(13)	4584.4(11)	5994.4(4)	38.2(3)
C3	393.0(13)	-1011.4(11)	4298.2(4)	37.7(3)
C2	502.2(13)	-925.8(10)	3908.0(4)	34.6(3)
C23	1006.8(17)	5786.1(12)	5848.8(5)	50.9(4)
C8	1132.3(13)	363.2(10)	3339.9(3)	36.8(3)
C14	-93.7(15)	-1337.0(13)	3059.8(4)	46.0(3)
C9	165.0(14)	-177.7(12)	3062.0(3)	40.4(3)
C19	1503.5(13)	3740.2(11)	5345.4(4)	39.4(3)
C20	2101.9(13)	3852.1(10)	5736.9(4)	37.2(3)
C10	-454.5(17)	508.5(15)	2789.0(4)	54.0(4)
C13	-955.7(19)	-1790.8(17)	2779.7(5)	64.3(5)

Atom	x	y	z	U_{eq}
C11	-1330(2)	46(2)	2517.5(5)	71.7(5)
C26	859(2)	5301.7(15)	6656.9(5)	61.8(4)
C25	684(2)	6496.1(14)	6512.5(6)	68.8(5)
C24	101.1(19)	6502.8(13)	6113.6(5)	59.6(4)
C12	-1565(2)	-1100(2)	2510.8(5)	78.3(6)
C15	559.3(17)	-2060.6(12)	3355.3(4)	49.5(4)

Table S9. Anisotropic Displacement Parameters (×10⁴) 40. The anisotropic displacement factor exponent takes the form: $-2p^2[h^2a^{*2} \times U_{11} + ... + 2hka^* \times b^* \times U_{12}]$

Atom	U_{11}	U_{22}	U_{33}	U_{23}	U_{13}	U_{12}
O21	20.0(4)	59.9(6)	48.7(5)	-3.4(4)	-2.1(3)	0.1(4)
O1	52.7(6)	32.3(5)	50.5(5)	-0.7(4)	1.7(4)	-8.0(4)
N18	20.2(5)	45.0(6)	43.0(6)	-3.3(4)	0.4(4)	0.5(4)
C7	29.4(6)	30.6(6)	36.2(6)	3.3(5)	1.8(4)	2.7(4)
C17	22.3(5)	39.0(6)	33.5(6)	7.9(5)	0.4(4)	-1.9(4)
O28	65.8(7)	46.8(5)	39.1(5)	5.4(4)	6.3(5)	-14.8(5)
C27	49.2(8)	45.3(7)	51.0(8)	-5.2(6)	-6.7(6)	1.0(6)
C6	26.7(5)	32.2(6)	38.6(6)	4.4(5)	3.6(5)	-0.2(4)
C16	22.5(5)	49.8(7)	39.2(6)	-0.6(5)	0.9(5)	-2.3(5)
C4	30.1(6)	43.1(7)	36.3(6)	9.7(5)	0.8(5)	3.7(5)
C5	22.5(5)	38.9(6)	37.4(6)	2.5(5)	0.5(4)	3.0(5)
C22	33.7(6)	34.2(6)	46.7(7)	-1.1(5)	-0.3(5)	-0.7(5)
C3	34.0(6)	33.6(6)	45.6(7)	12.4(5)	0.9(5)	-0.1(5)
C2	30.0(6)	29.2(6)	44.5(7)	3.7(5)	-1.5(5)	1.2(5)
C23	55.7(9)	36.4(7)	60.7(9)	5.0(6)	8.6(7)	5.0(6)
C8	39.3(7)	34.5(6)	36.7(6)	1.0(5)	5.5(5)	1.8(5)
C14	42.8(7)	51.8(8)	43.3(7)	-12.2(6)	7.6(6)	-3.4(6)
C9	39.9(7)	49.7(7)	31.6(6)	-2.5(5)	5.7(5)	0.4(6)
C19	30.7(6)	41.0(7)	46.4(7)	-0.5(5)	0.3(5)	4.3(5)
C20	29.0(6)	34.6(6)	48.2(7)	-0.5(5)	-2.9(5)	0.1(5)
C10	57.0(9)	71.2(10)	33.8(7)	6.2(7)	2.8(6)	2.3(8)
C13	63.0(10)	78.5(12)	51.5(9)	-23.0(8)	5.5(8)	-17.5(9)
C11	66.3(11)	110.9(16)	38.0(8)	6.3(9)	-7.9(7)	-2.7(11)
C26	76.2(11)	58.0(9)	51.2(9)	-14.3(7)	0.8(8)	0.4(8)
C25	77.6(12)	48.4(9)	80.3(12)	-23.2(8)	14.0(10)	-3.7(8)

Atom	U_{11}	U_{22}	U_{33}	U_{23}	U_{13}	U_{12}
C24	64.4(10)	37.2(7)	77.3(11)	-1.4(7)	11.5(8)	11.7(7)
C12	68.9(12)	123.5(18)	42.5(8)	-16.5(10)	-7.5(8)	-19.7(12)
C15	54.1(8)	34.7(7)	59.6(8)	-10.4(6)	5.9(7)	0.4(6)

Table S10. Bond Lengths in Å for 40

Atom	Atom	Length/Å
O21	C17	1.2303(14)
O1	C2	1.3624(15)
O1	C15	1.4348(17)
N18	C17	1.3319(15)
N18	C19	1.4493(16)
C7	C2	1.4028(16)
C7	C6	1.4095(17)
C7	C8	1.4952(17)
C17	C16	1.5173(17)
O28	C8	1.2235(16)
C27	C22	1.5132(19)
C27	C26	1.523(2)
C6	C5	1.3755(17)
C16	C5	1.4999(17)
C4	C3	1.3707(19)
C4	C5	1.4003(17)
C22	C23	1.5262(18)
C22	C20	1.5271(17)
C3	C2	1.3925(18)
C23	C24	1.528(2)
C8	C9	1.4887(18)
C14	C13	1.393(2)
C14	C9	1.397(2)
C14	C15	1.489(2)
C9	C10	1.394(2)
C19	C20	1.5056(18)
C10	C11	1.382(2)

Atom	Atom	Length/Å
C13	C12	1.383(3)
C11	C12	1.378(3)
C26	C25	1.516(3)
C25	C24	1.519(3)

Table S11. Bond Angles in ° for 40

Atom	Atom	Atom	Angle/°
C2	O1	C15	116.57(10)
C17	N18	C19	122.70(10)
C2	C7	C6	116.92(11)
C2	C7	C8	127.51(11)
C6	C7	C8	115.29(10)
O21	C17	N18	123.26(11)
O21	C17	C16	122.37(11)
N18	C17	C16	114.36(10)
C22	C27	C26	112.17(12)
C5	C6	C7	123.30(11)
C5	C16	C17	115.59(10)
C3	C4	C5	120.67(11)
C6	C5	C4	117.79(11)
C6	C5	C16	121.20(11)
C4	C5	C16	121.01(11)
C27	C22	C23	109.80(12)
C27	C22	C20	111.08(11)
C23	C22	C20	113.08(11)

Atom	Atom	Atom	Angle/°
C4	C3	C2	121.01(11)
O1	C2	C3	113.98(11)
O1	C2	C7	125.98(11)
C3	C2	C7	120.03(11)
C22	C23	C24	111.97(12)
O28	C8	C9	118.28(11)
O28	C8	C7	119.64(12)
C9	C8	C7	121.84(11)
C13	C14	C9	119.11(15)
C13	C14	C15	121.43(15)
C9	C14	C15	119.45(12)
C10	C9	C14	119.87(14)
C10	C9	C8	117.74(13)
C14	C9	C8	122.35(12)
N18	C19	C20	113.19(10)
C19	C20	C22	113.03(10)
C11	C10	C9	120.22(17)
C12	C13	C14	120.40(18)
C12	C11	C10	119.97(18)
C25	C26	C27	110.94(14)
C26	C25	C24	111.08(14)
C25	C24	C23	111.61(14)
C11	C12	C13	120.40(17)
O1	C15	C14	110.94(11)

Table S12. Torsion Angles in ° for 40

Atom	Atom	Atom	Atom	Angle/°
C19	N18	C17	O21	-7.18(18)

Atom	Atom	Atom	Atom	Angle/˚
C19	N18	C17	C16	171.96(11)
C2	C7	C6	C5	-2.99(17)
C8	C7	C6	C5	171.45(11)
O21	C17	C16	C5	-8.38(17)
N18	C17	C16	C5	172.46(10)
C7	C6	C5	C4	-1.30(17)
C7	C6	C5	C16	177.95(11)
C3	C4	C5	C6	2.69(17)
C3	C4	C5	C16	-176.56(11)
C17	C16	C5	C6	113.22(12)
C17	C16	C5	C4	-67.55(15)
C26	C27	C22	C23	56.08(17)
C26	C27	C22	C20	-178.10(12)
C5	C4	C3	C2	0.32(18)
C15	O1	C2	C3	-152.74(12)
C15	O1	C2	C7	26.05(17)
C4	C3	C2	O1	174.06(11)
C4	C3	C2	C7	-4.80(18)
C6	C7	C2	O1	-172.75(11)
C8	C7	C2	O1	13.6(2)
C6	C7	C2	C3	5.96(17)
C8	C7	C2	C3	-167.69(12)
C27	C22	C23	C24	-54.76(17)
C20	C22	C23	C24	-179.43(12)
C2	C7	C8	O28	-174.45(12)
C6	C7	C8	O28	11.80(17)
C2	C7	C8	C9	11.36(19)
C6	C7	C8	C9	-162.38(11)

Atom	Atom	Atom	Atom	Angle/˚
C13	C14	C9	C10	0.9(2)
C15	C14	C9	C10	-179.28(13)
C13	C14	C9	C8	-176.81(13)
C15	C14	C9	C8	3.0(2)
O28	C8	C9	C10	-32.82(18)
C7	C8	C9	C10	141.44(13)
O28	C8	C9	C14	144.95(13)
C7	C8	C9	C14	-40.79(18)
C17	N18	C19	C20	145.36(11)
N18	C19	C20	C22	-169.00(10)
C27	C22	C20	C19	176.20(11)
C23	C22	C20	C19	-59.82(15)
C14	C9	C10	C11	0.3(2)
C8	C9	C10	C11	178.18(14)
C9	C14	C13	C12	-1.0(2)
C15	C14	C13	C12	179.19(16)
C9	C10	C11	C12	-1.5(3)
C22	C27	C26	C25	-56.92(19)
C27	C26	C25	C24	55.2(2)
C26	C25	C24	C23	-54.4(2)
C22	C23	C24	C25	54.64(19)
C10	C11	C12	C13	1.5(3)
C14	C13	C12	C11	-0.2(3)
C2	O1	C15	C14	-82.55(14)
C13	C14	C15	O1	-112.08(15)
C9	C14	C15	O1	68.13(17)

Table S13. Hydrogen Fractional Atomic Coordinates (×10^4) and Equivalent Isotropic Displacement Parameters (Å^2×10^3) for 40. U_{eq} is defined as 1/3 of the trace of the orthogonalised U_{ij}.

Atom	x	y	z	U_{eq}
H18	3125.1	2778.65	5154.55	43
H27A	2746.98	4929.97	6385.43	58
H27B	1847.41	3827.17	6487.32	58
H6	2279.22	1423.51	3888.06	39
H16A	3078.75	2021.06	4470.41	45
H16B	3137.33	1100.83	4798.12	45
H4	920.81	-300.19	4795.16	44
H22	224.99	4233.69	6006.62	46
H3	-112.47	-1624.84	4405.72	45
H23A	554.91	5764.11	5597.11	61
H23B	1951.87	6139.8	5820.64	61
H19A	482.54	3553.11	5363.4	47
H19B	1587.03	4474.63	5214.86	47
H20A	3063.69	4183.37	5720.89	45
H20B	2193.02	3092.26	5849.63	45
H10	-274.61	1296.31	2789.59	65
H13	-1126.15	-2579.73	2773.18	77
H11	-1768.9	518.79	2335.55	86
H26A	-84.38	4943.07	6681.18	74
H26B	1302.62	5319.17	6909.72	74
H25A	1613.24	6883.96	6516.89	83
H25B	29.59	6913.69	6680.19	83
H24A	72.13	7287.62	6019.2	72
H24B	-881.3	6208.36	6115.46	72
H12	-2149.75	-1417.34	2320.33	94

Atom	x	y	z	U_{eq}
H15A	403.6	-2863.59	3291.75	59
H15B	1594.8	-1924.3	3362.99	59
