# Supplementary material for: NiH-catalyzed anti-Markovnikov hydroamidation of unactivated alkenes with 1,4,2-dioxazol-5-ones for the direct synthesis of N-alkyl amides
Source: Commun Chem. 2022 Dec 22;5:176. doi: 10.1038/s42004-022-00791-4 (PMC9814879; doi:10.1038/s42004-022-00791-4)
Supplement: Supplementary file 5 — Supplementary Data 2 [file 42004_2022_791_MOESM5_ESM.pdf]

DFT calculations.

All molecular geometries of the model complexes were fully optimized via DFT calculations with the ωB97XD[10] functional using a mixed basis set of Stuttgart-Dresden-Bonn (SDD)[11] for Ni and 6-31G(d,p)[12] for all other atoms in the gas phase as implemented in Gaussian 09 suite of programs[13]. Frequency calculations at the same level of theory have also been performed to identify all of the stationary points as the local minima (no imaginary frequencies) or transition states (one imaginary frequency) and to provide free energies at 298.15 K. Transition states were further confirmed by intrinsic reaction coordinate calculations (IRC)[14]. For solvation correction, single point energies based on the gas-phase optimized structures were calculated with the ωB97XD functional using a mixed basis set of def2TZVP[15] for Ni and 6-311+G(d,p) for all other atoms and employing the SMD model (solvation model based on solute electron density)[7] with Tetrahydrofuran (THF) as the solvent.

The energies of calculated structures

**Level of theory**	wB97XD/SDD (Ni)/6-31G(d,p)		wB97XD/def2TZVP (Ni)/6-311+G(d,p)	
	**E(gas)**	**G(gas)**	**E(sol)**	**G(sol)**
**A**	-939.3112	-939.0320	-2276.9852	-2276.7060
**TS AB**	-939.3049	-939.0263	-2276.9789	-2276.7002
**B**	-939.3349	-939.0522	-2277.0098	-2276.7270
**TS B-C**	-1335.7332	-1335.3935	-2673.5205	-2673.1808
**C**	-1335.7419	-1335.3994	-2673.5288	-2673.1863
**TS C-D**	-1335.6838	-1335.3510	-2673.4878	-2673.1549
**D**	-1147.2042	-1146.8775	-2484.9403	-2484.6136
**TS D-E**	-1558.9870	-1558.4716	-2896.8170	-2896.3016
**E**	-1558.9947	-1558.4789	-2896.8253	-2896.3095
**TS E-F**	-1558.9761	-1558.4620	-2896.8041	-2896.2900
**F**	-1559.1079	-1558.5903	-2896.9336	-2896.4159
**TS F-G**	-1559.0947	-1558.5755	-2896.9193	-2896.4001
**G**	-1559.1373	-1558.6198	-2896.9559	-2896.4383
**H**	-737.6544	-737.3652	-737.8423	-737.5532
**propene**	-117.8705	-117.8152	-117.9010	-117.8456
**methyl-dioxazolone**	-396.3846	-396.3467	-396.5041	-396.4662
**CO2**	-188.5174	-188.5262	-188.5824	-188.5913
**HBpin**	-411.7616	-411.6013	-411.8664	-411.7061
**TS-L L2 (C-D)**	-1608.3788	-1607.8915	-2946.2341	-2945.7467
**TS-B L2 (C-D)**	-1608.3857	-1607.8942	-2946.2386	-2945.7471

**AG**	-1831.8358	-1831.1677	-3169.7101	-3169.0420
**TS-L L3 (C-D)**	-1647.6936	-1647.1780	-2985.5564	-2985.0409
**TS-B L3 (C-D)**	-1647.6966	-1647.1787	-2985.5580	-2985.0401
**BG**	-1871.1490	-1870.4514	-3209.0276	-3208.3300
**TS-L L4 (C-D)**	-1883.5352	-1882.8554	-3221.4442	-3220.7644
**TS-B L4 (C-D)**	-1883.5302	-1882.8471	-3221.4378	-3220.7547
**CG**	-2106.9891	-2106.1269	-3444.9145	-3444.0523
**A-CH**	-1049.6533	-1049.1852	-1049.9044	-1049.4363
**cyclohexylethene**	-313.1949	-313.0224	-313.2662	-313.0936
**cyclobutyl-dioxazolone**	-513.0607	-512.9620	-513.2028	-513.1041
