# Supplementary material for: NiH-catalyzed anti-Markovnikov hydroamidation of unactivated alkenes with 1,4,2-dioxazol-5-ones for the direct synthesis of N-alkyl amides
Source: Commun Chem. 2022 Dec 22;5:176. doi: 10.1038/s42004-022-00791-4 (PMC9814879; doi:10.1038/s42004-022-00791-4)

NMR Spectra

^{1}H and ^{13}C NMR spectrum of **113**

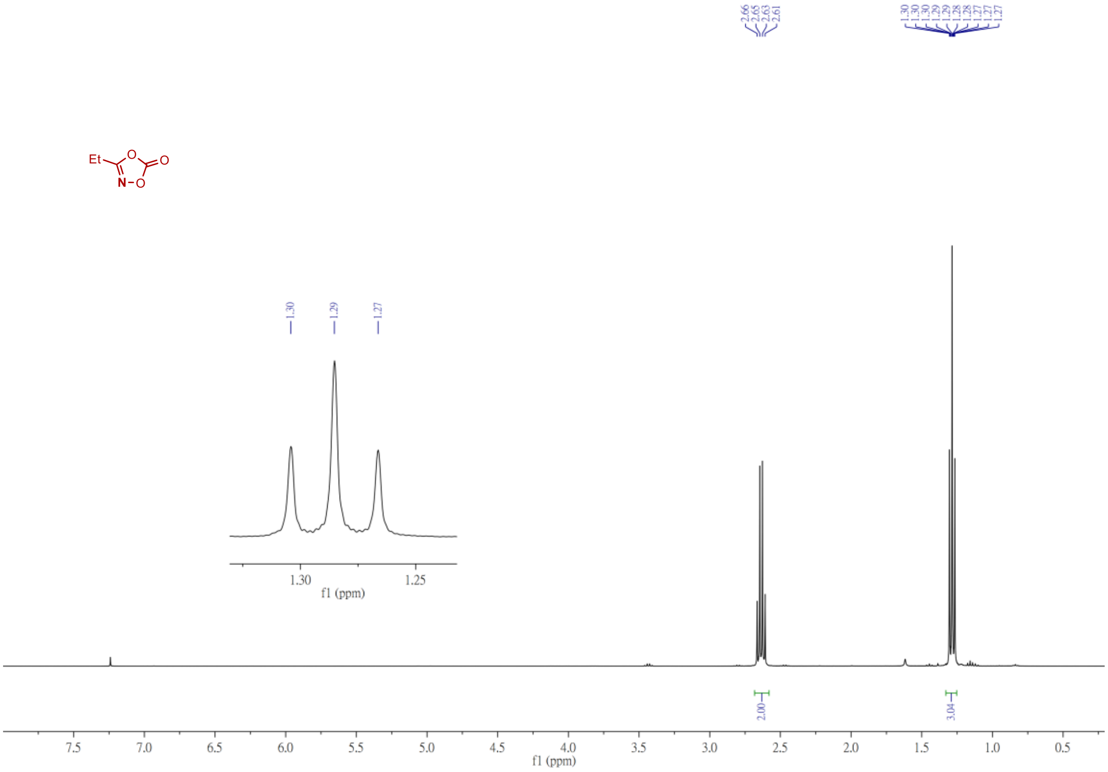

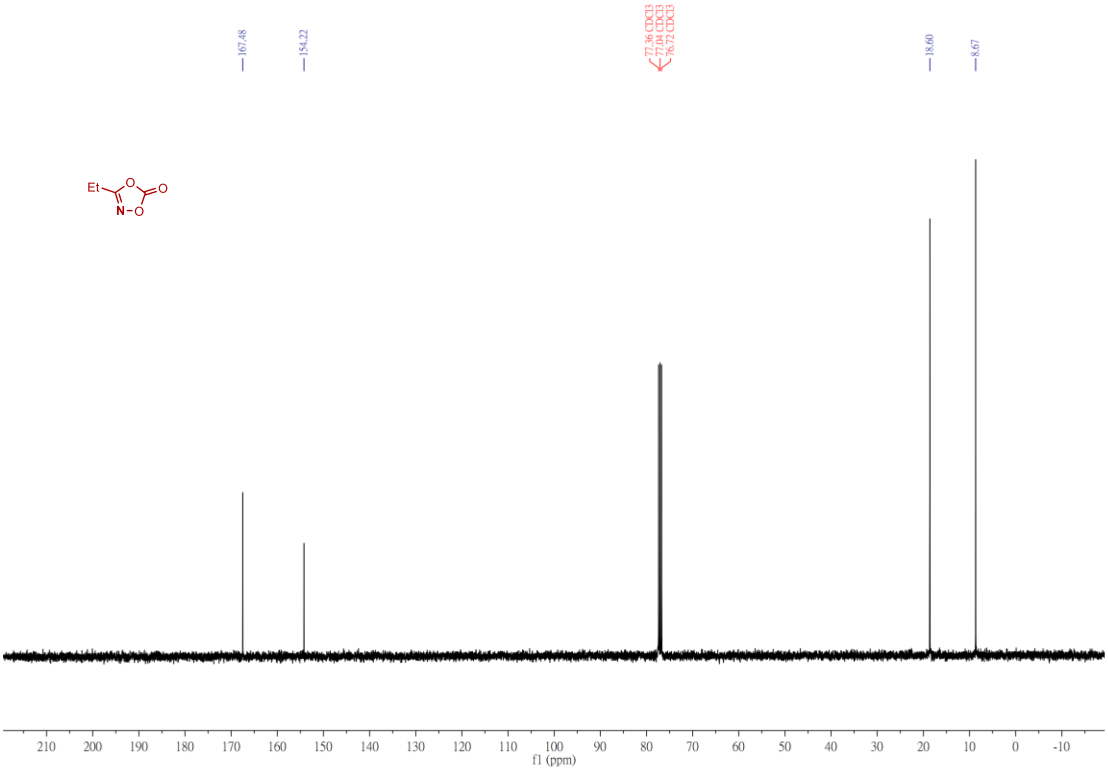

^1H and ^{13}C NMR spectrum of **S1**

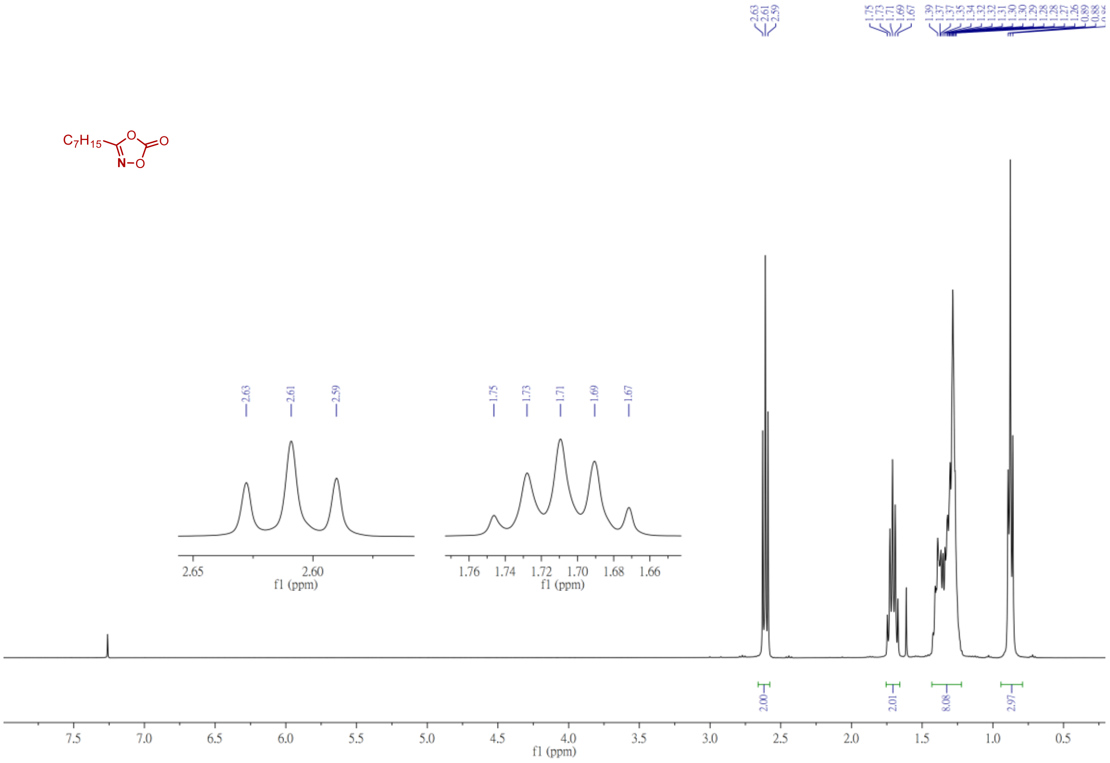

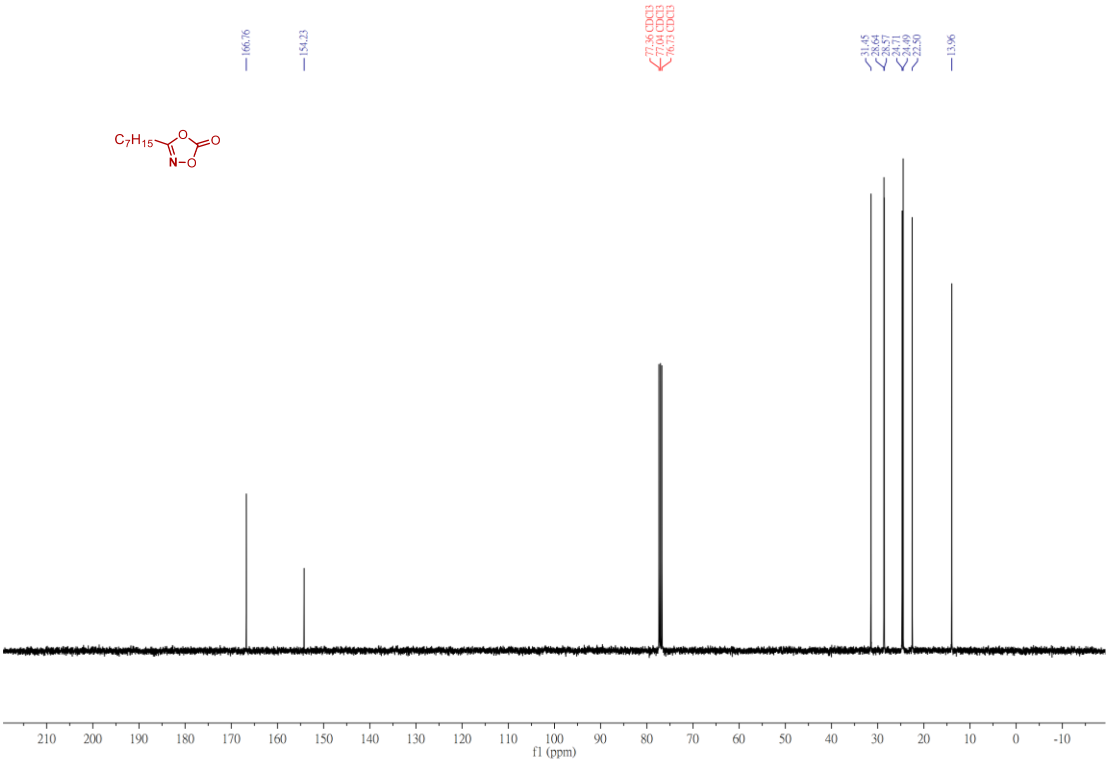

^{1}H and ^{13}C NMR spectrum of **S2**

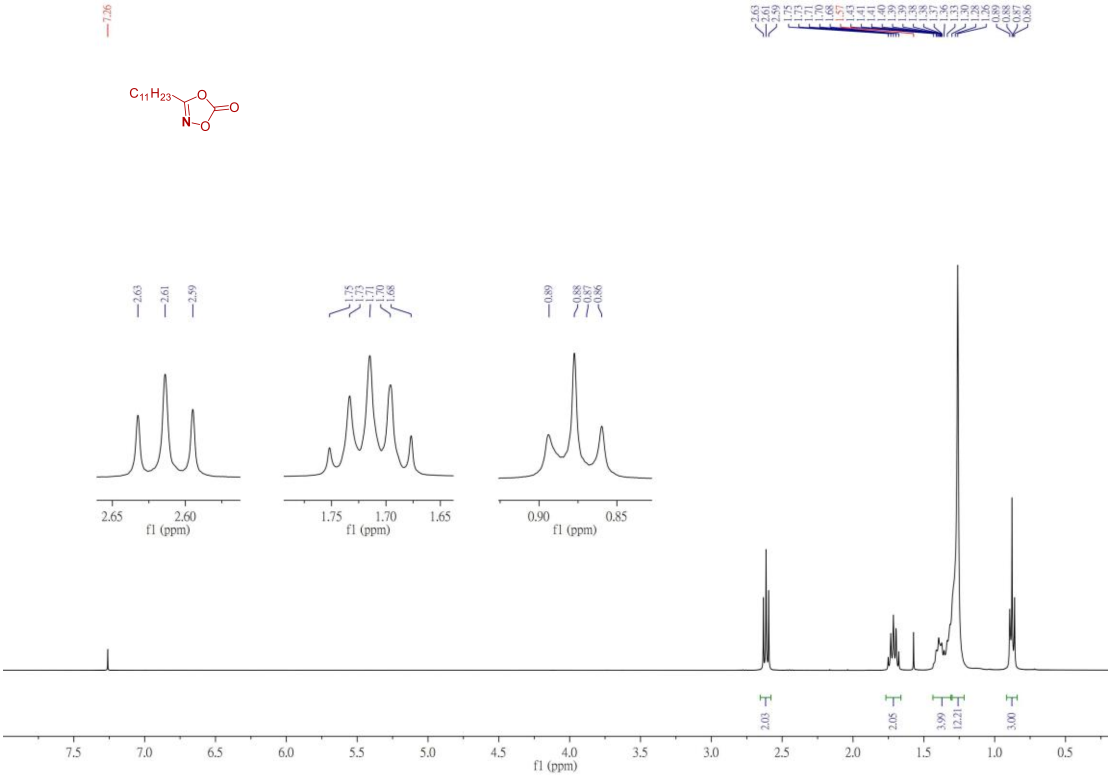

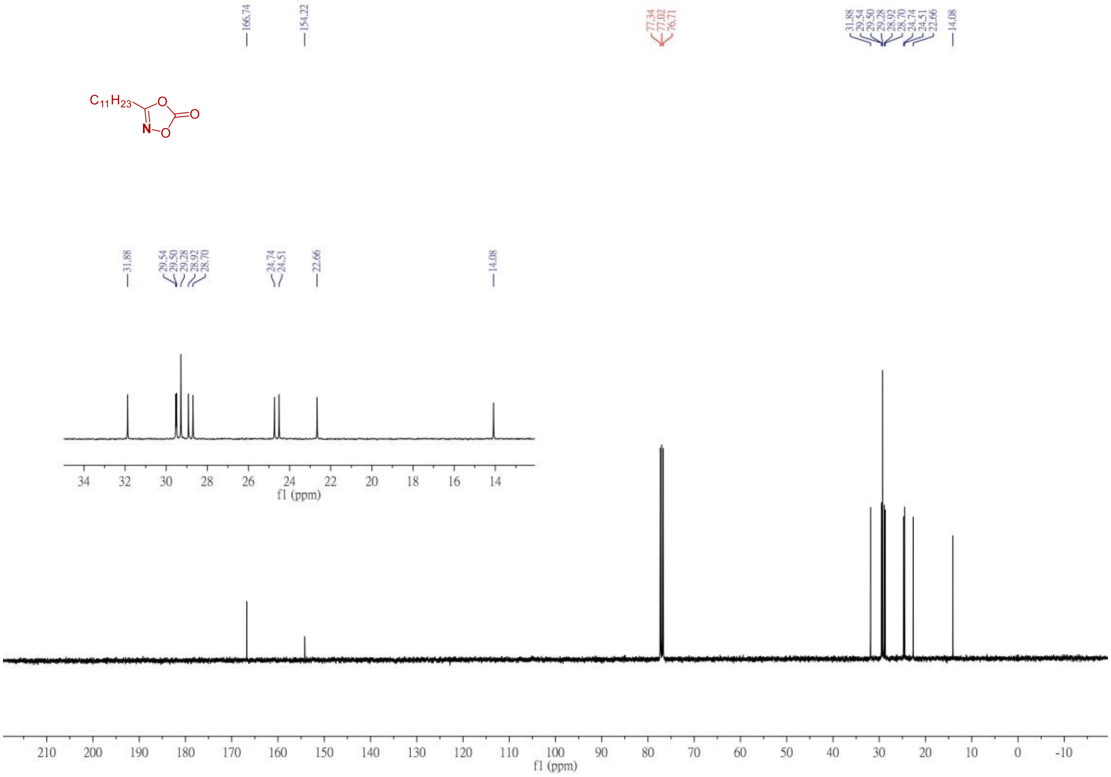

^{1}H and ^{13}C NMR spectrum of **S3**

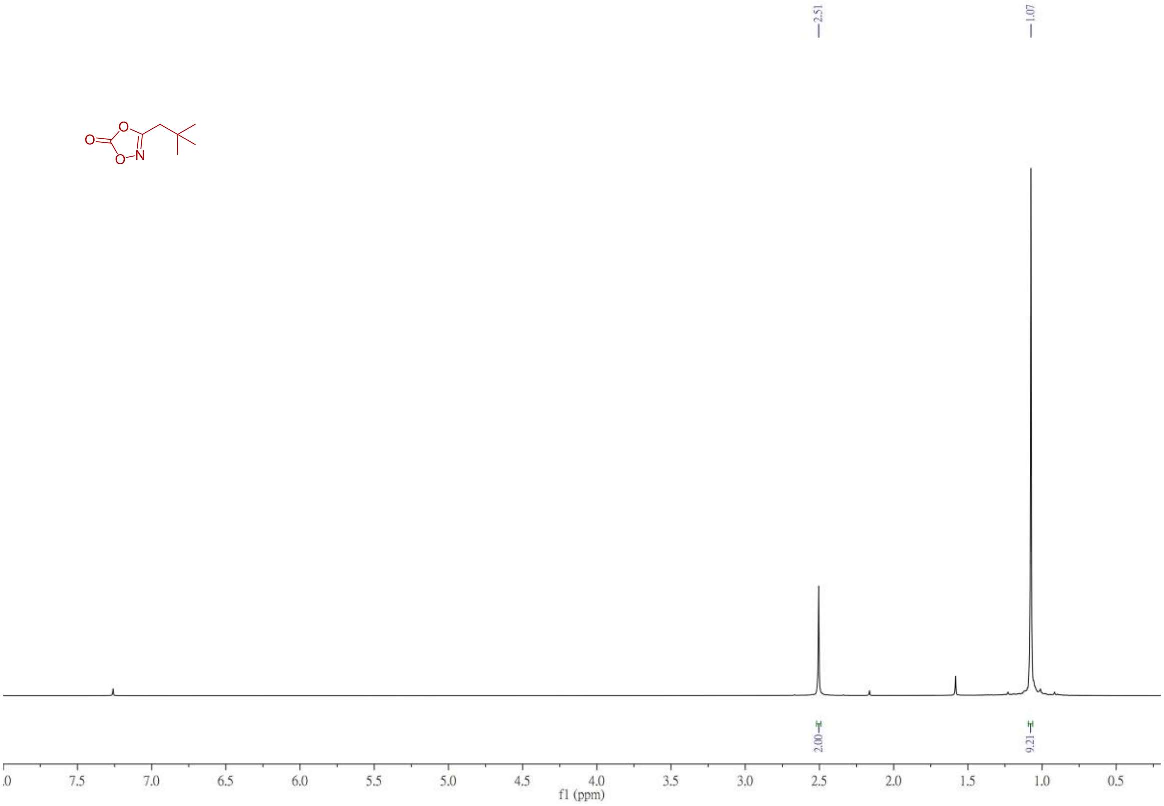

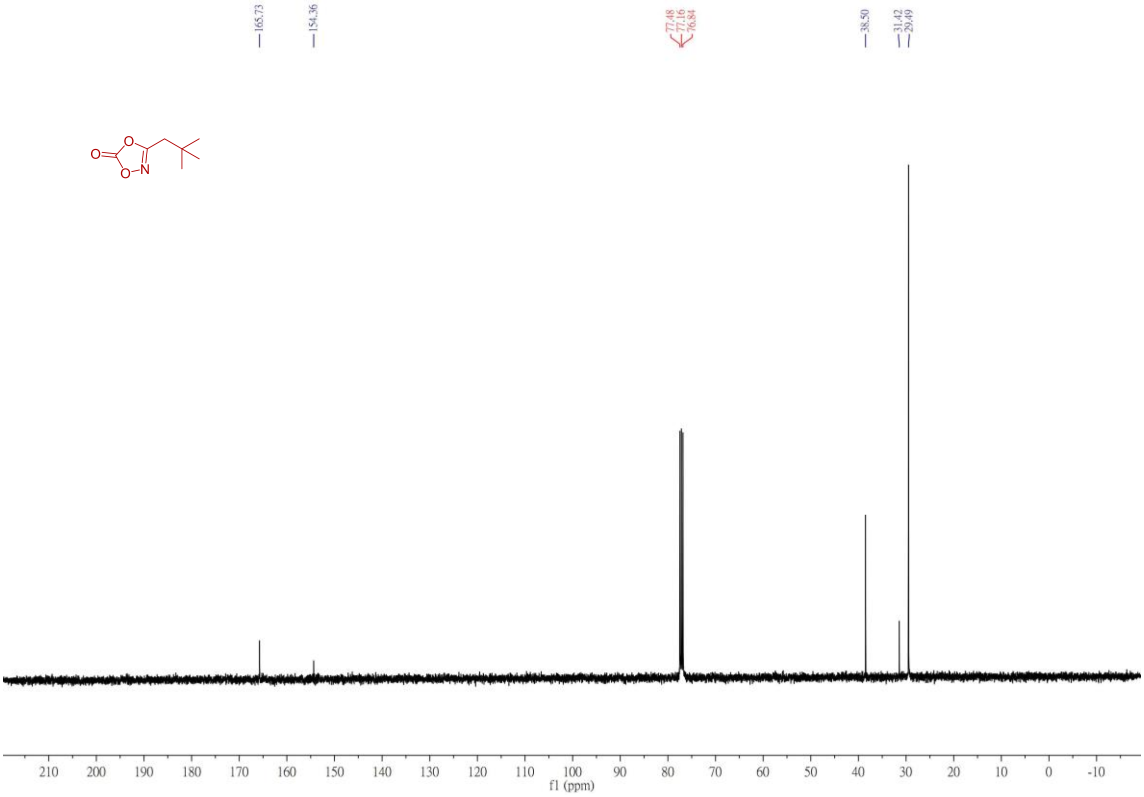

^{1}H and ^{13}C NMR spectrum of **S4**

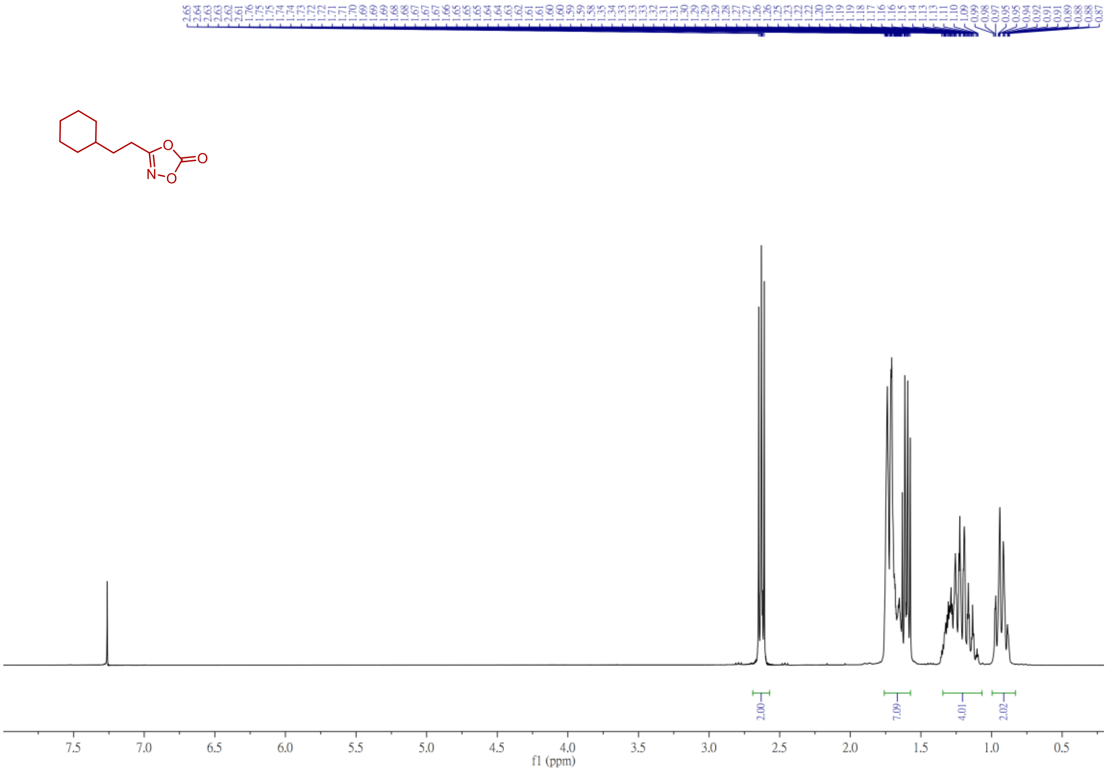

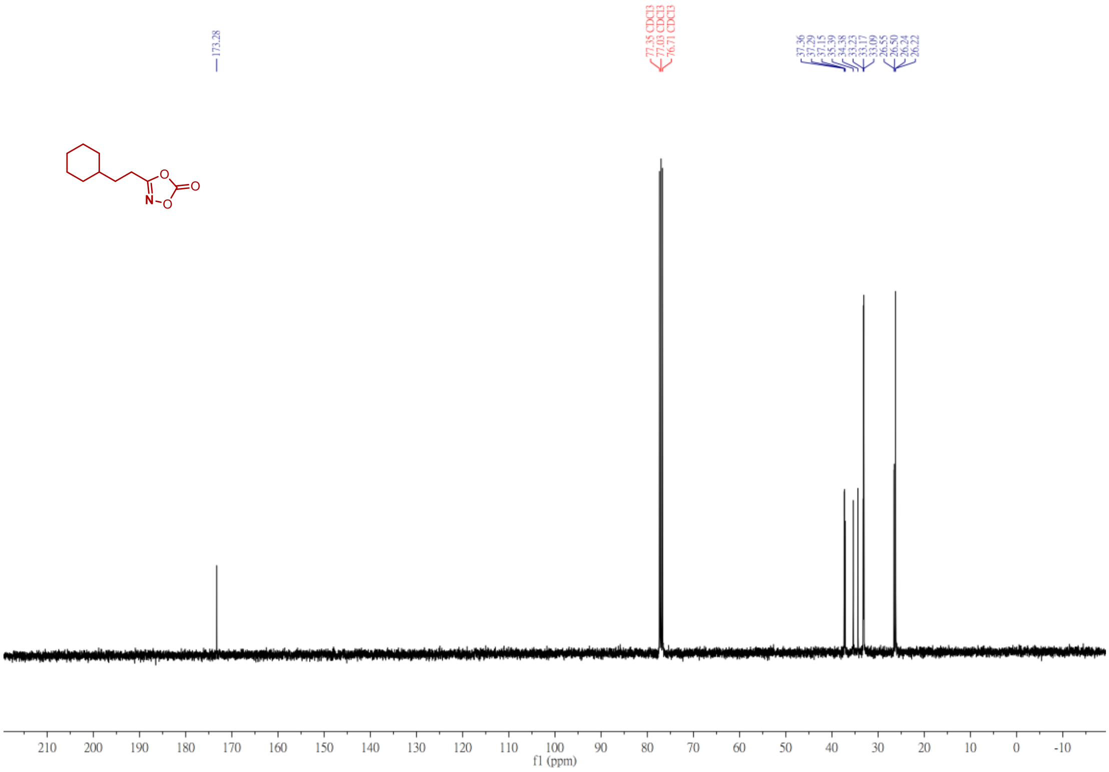

^{1}H and ^{13}C NMR spectrum of **S5**

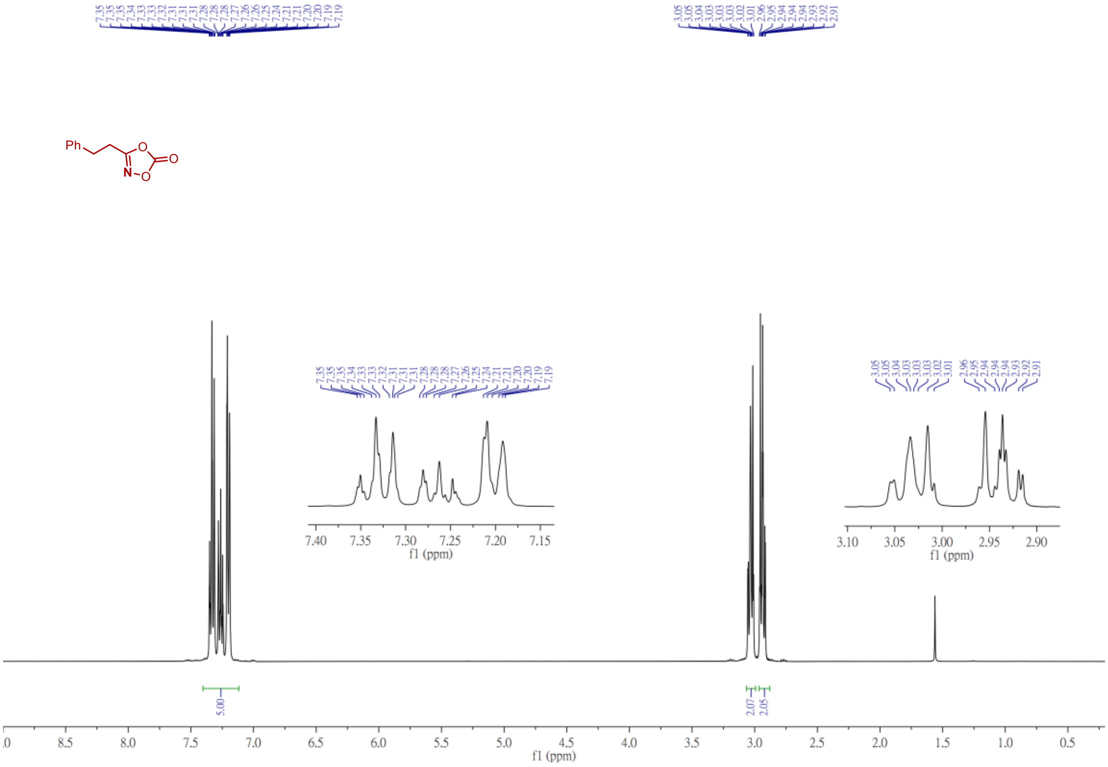

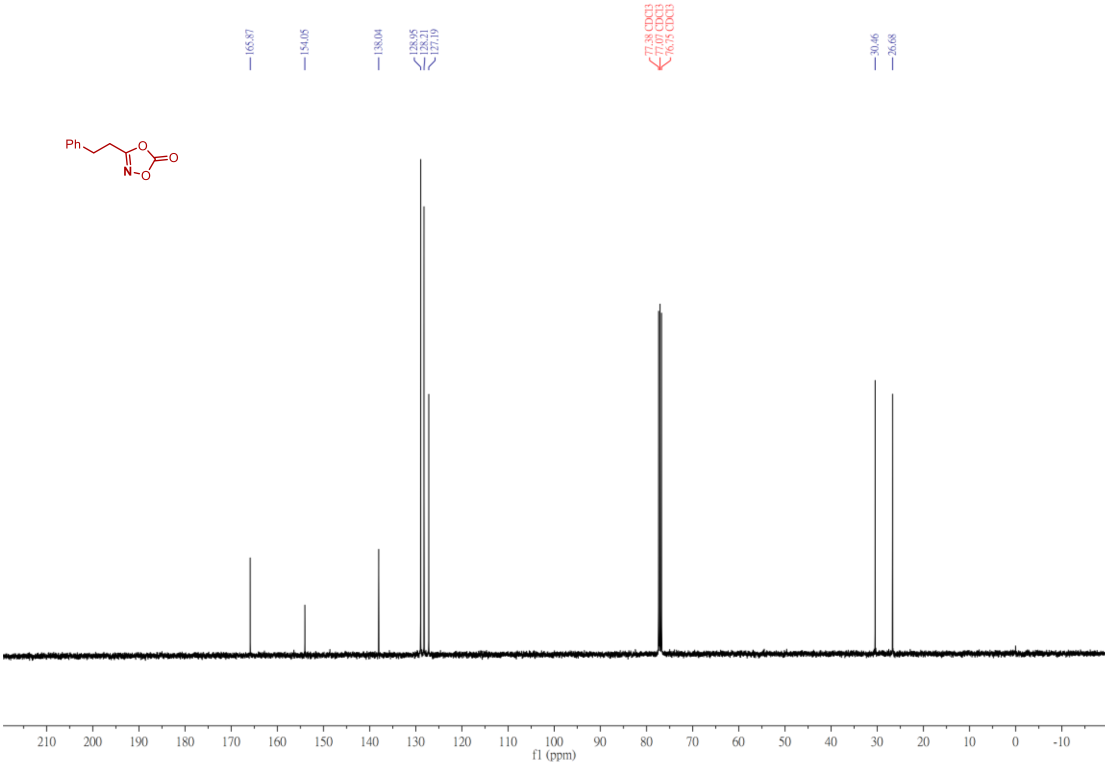

^{1}H and ^{13}C NMR spectrum of **S6**

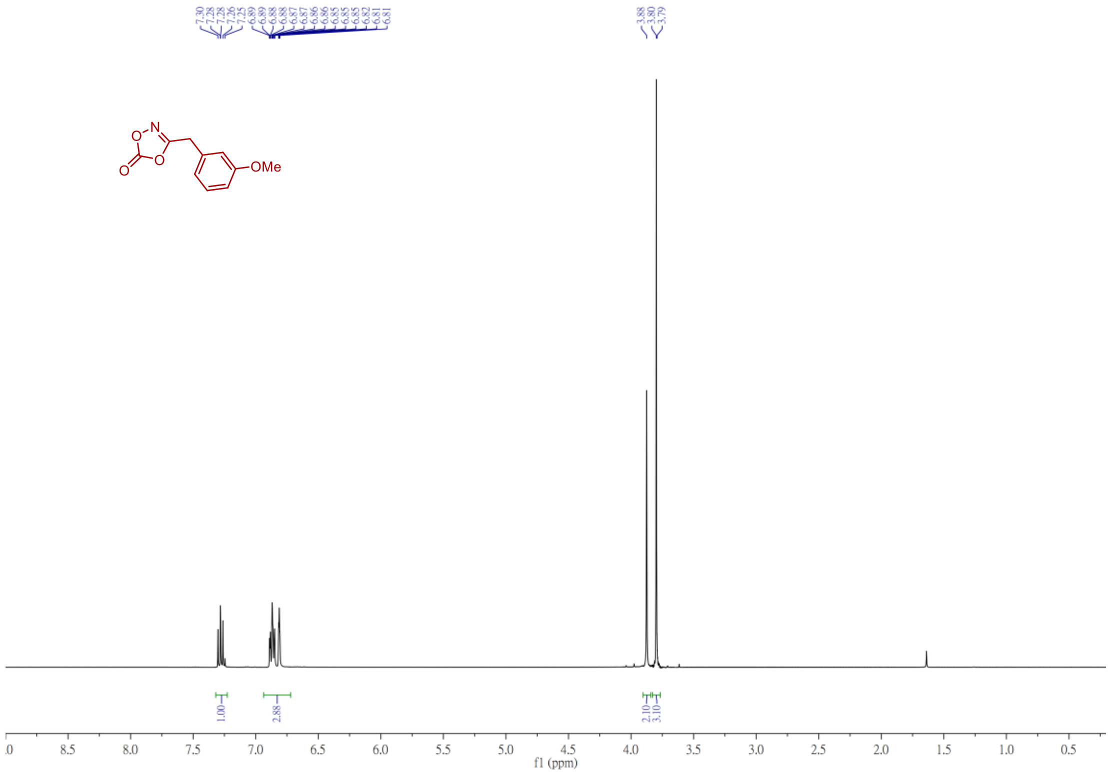

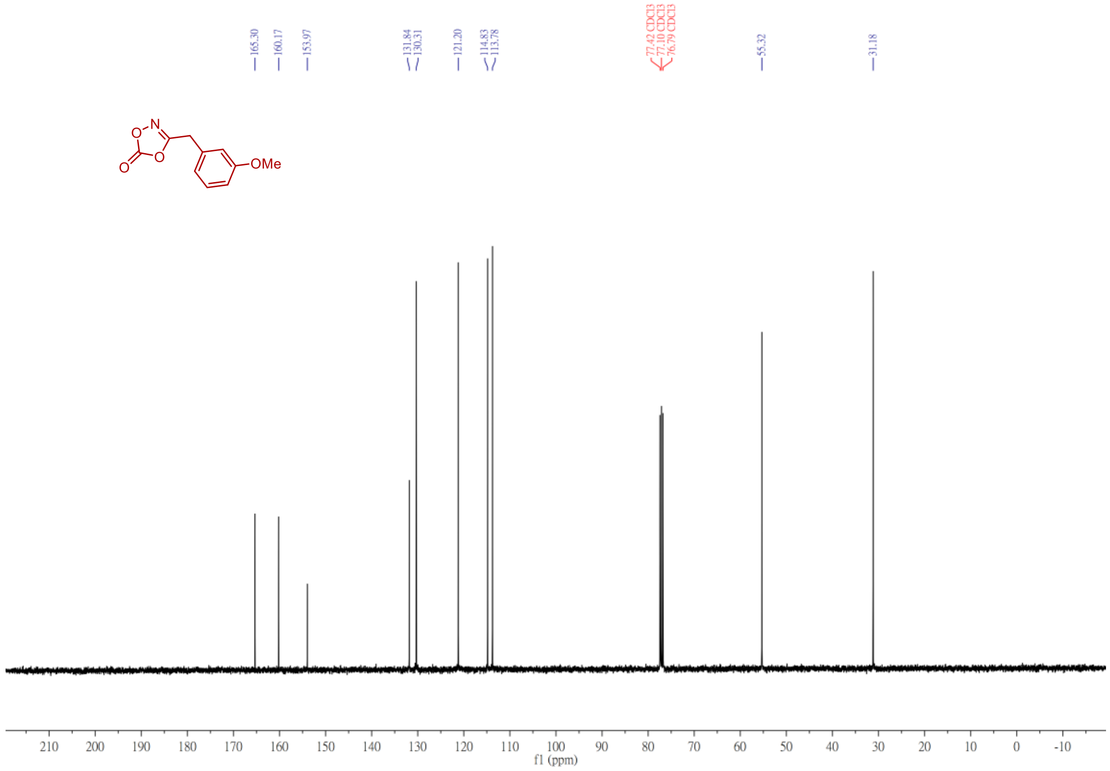

^{1}H and ^{13}C NMR spectrum of **S7**

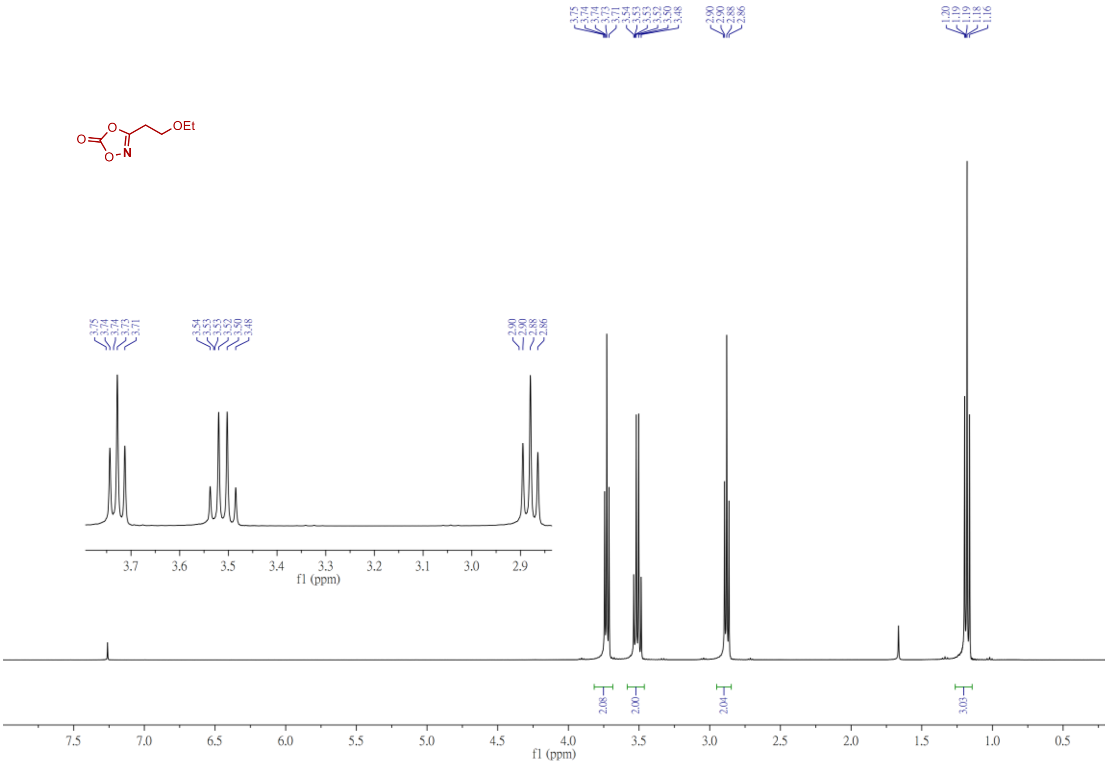

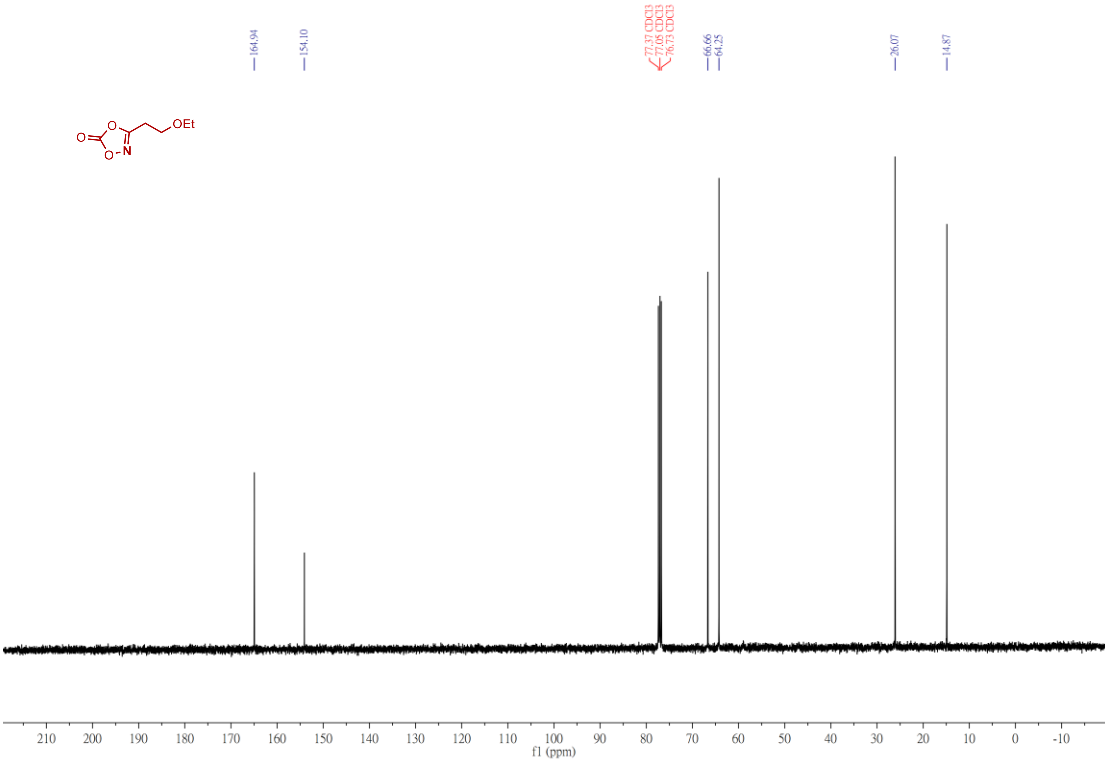

^{1}H and ^{13}C NMR spectrum of **S8**

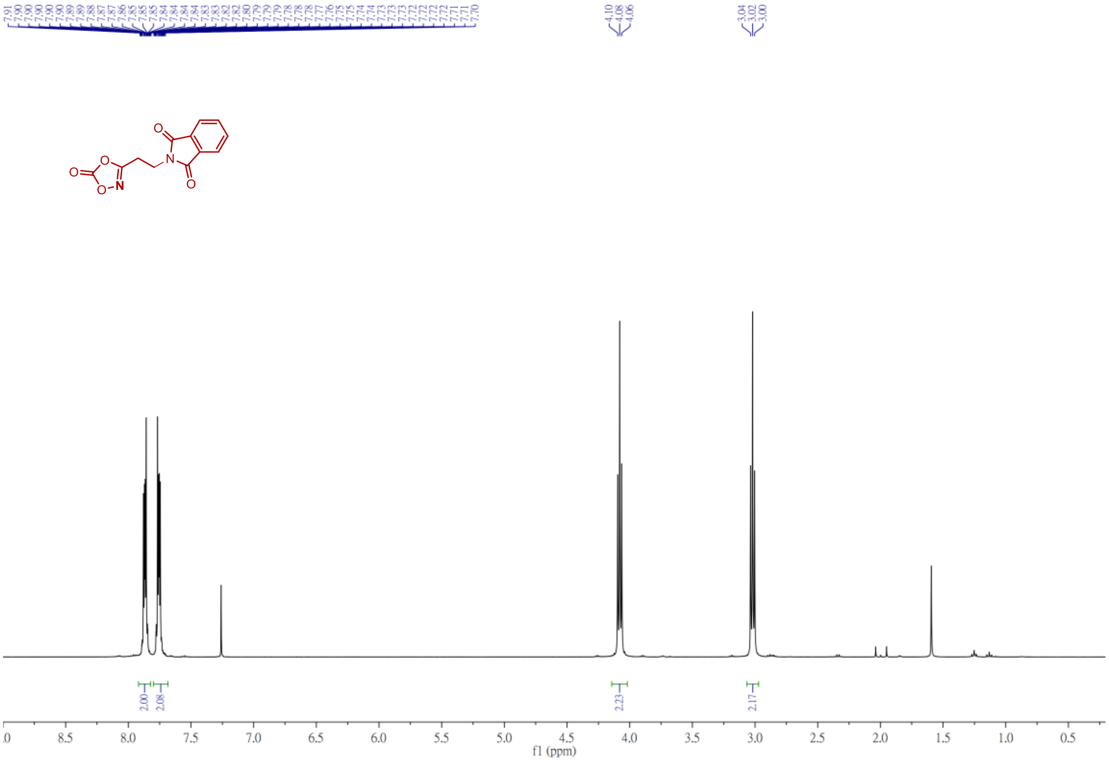

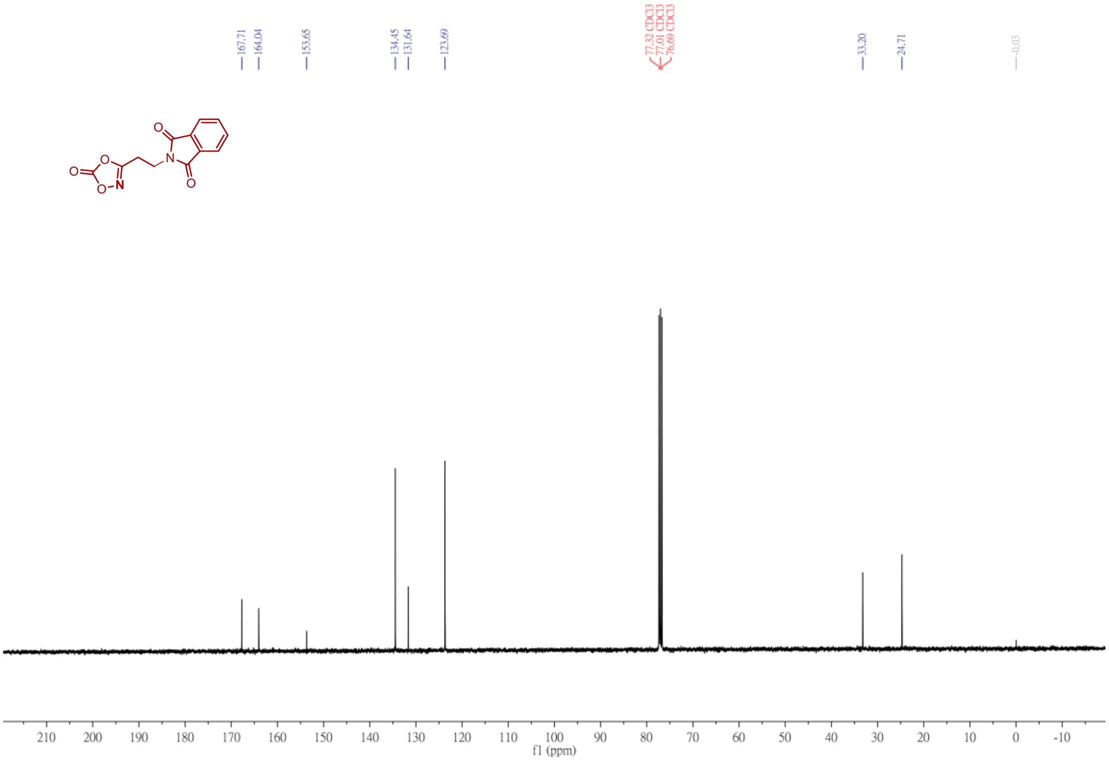

^{1}H and ^{13}C NMR spectrum of **S9**

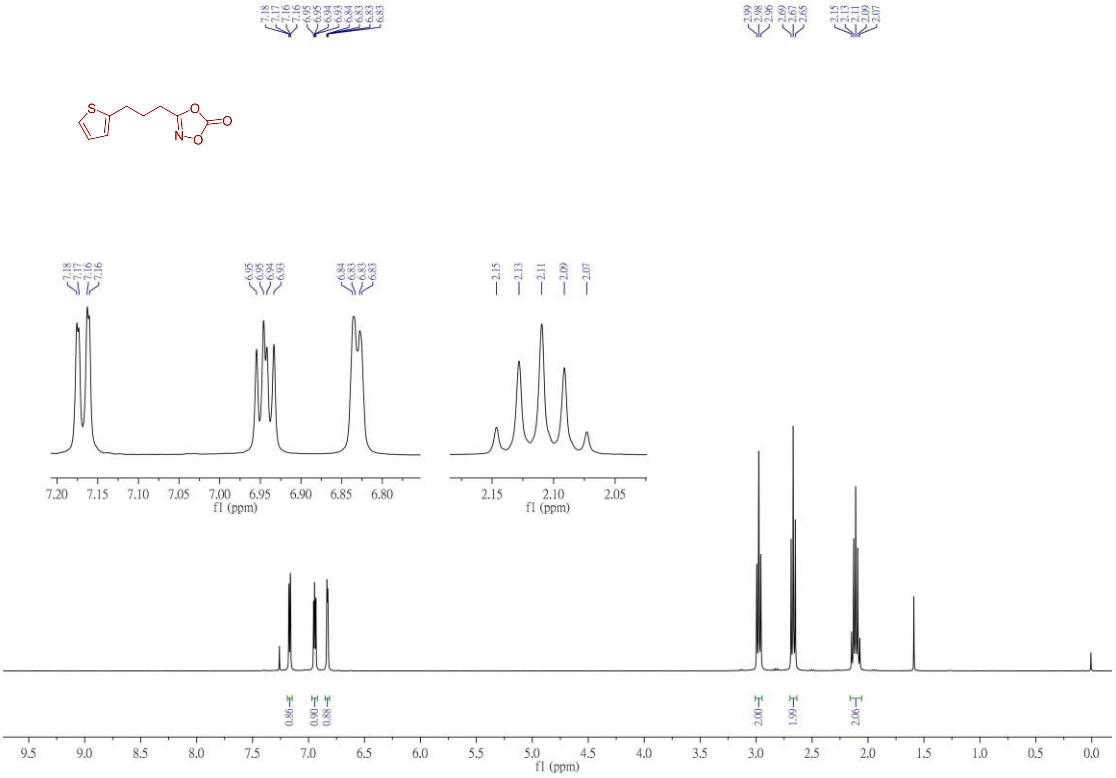

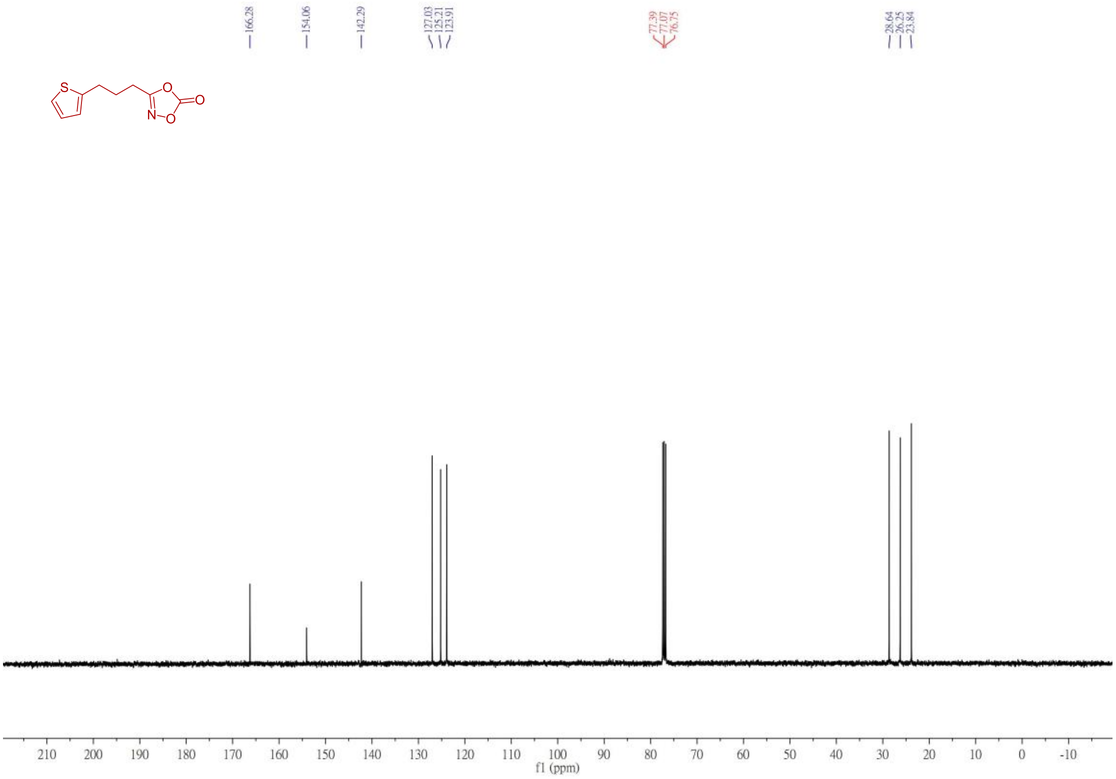

^{1}H and ^{13}C NMR spectrum of **S10**

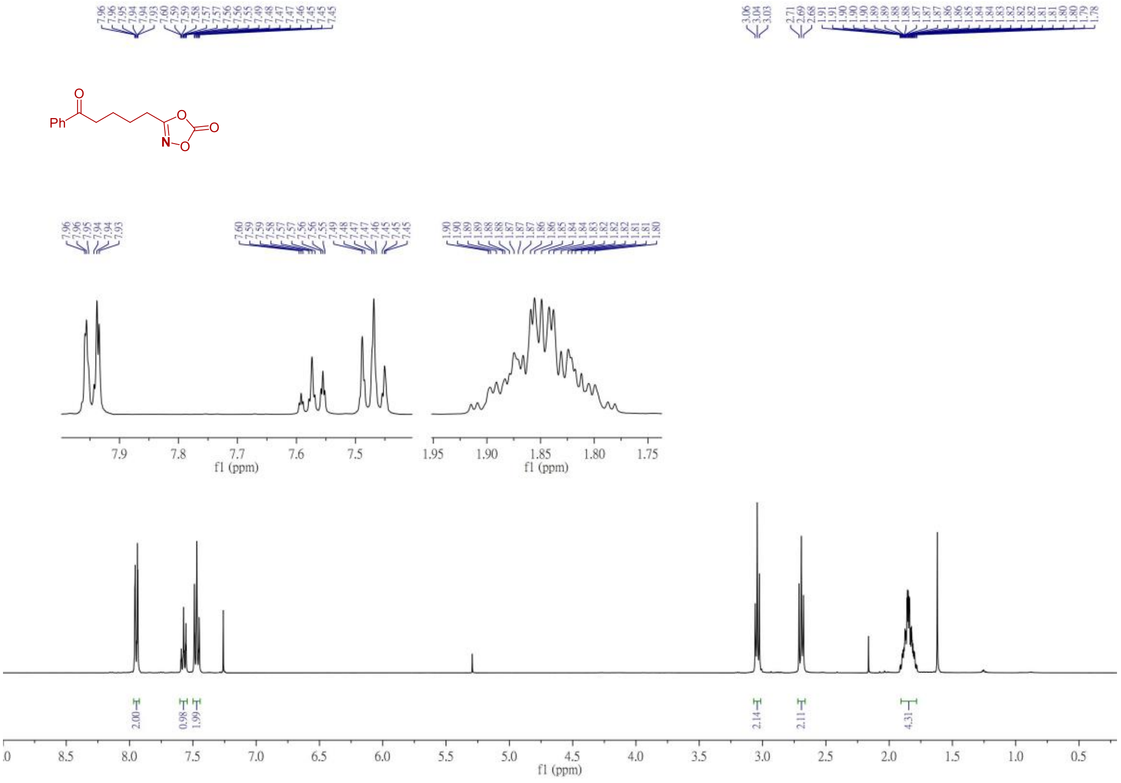

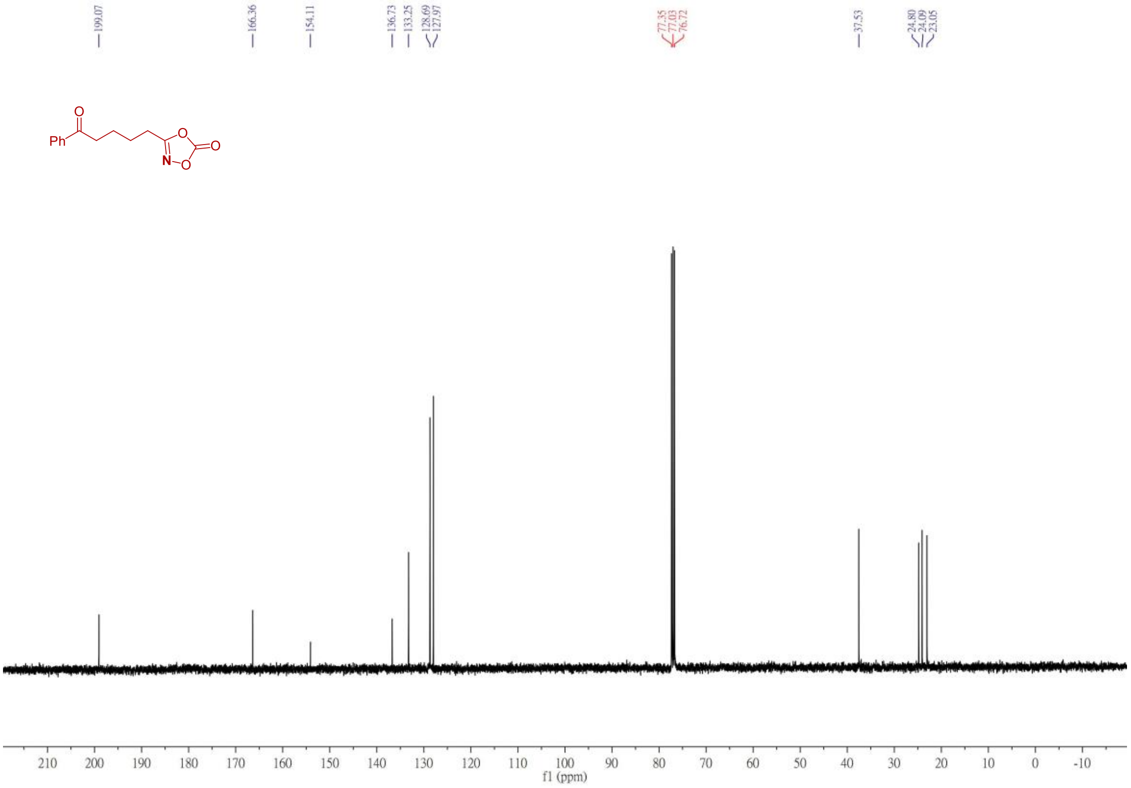

^{1}H and ^{13}C NMR spectrum of **S11**

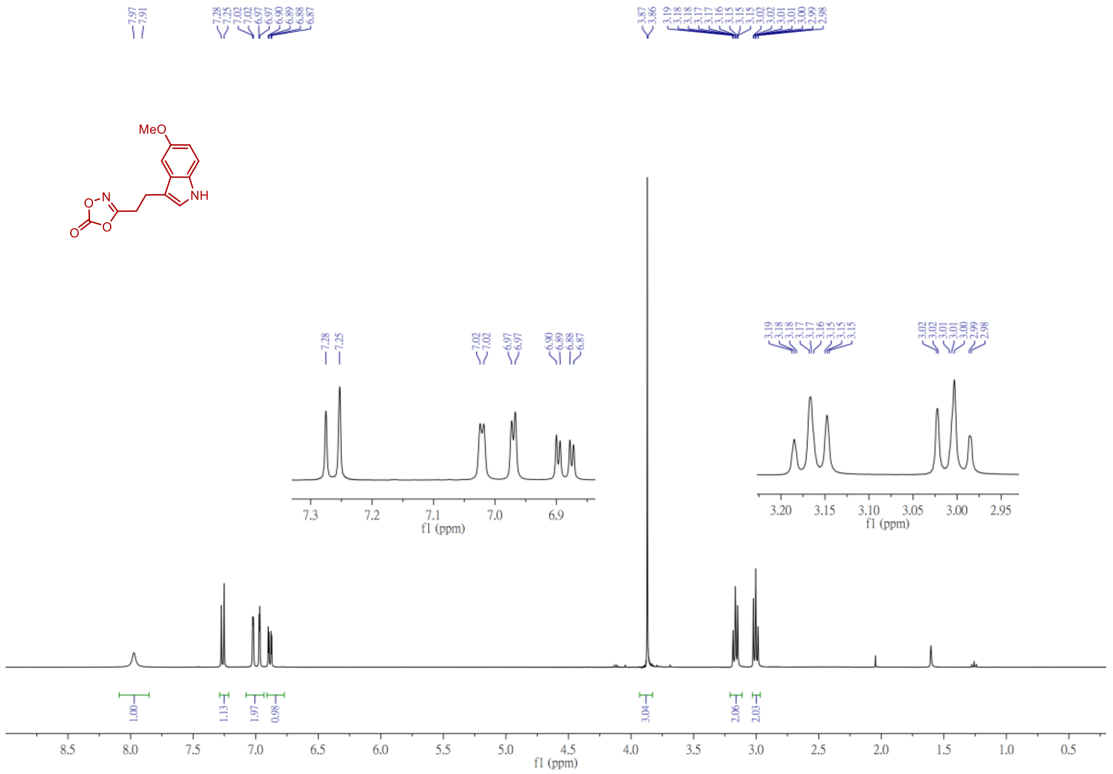

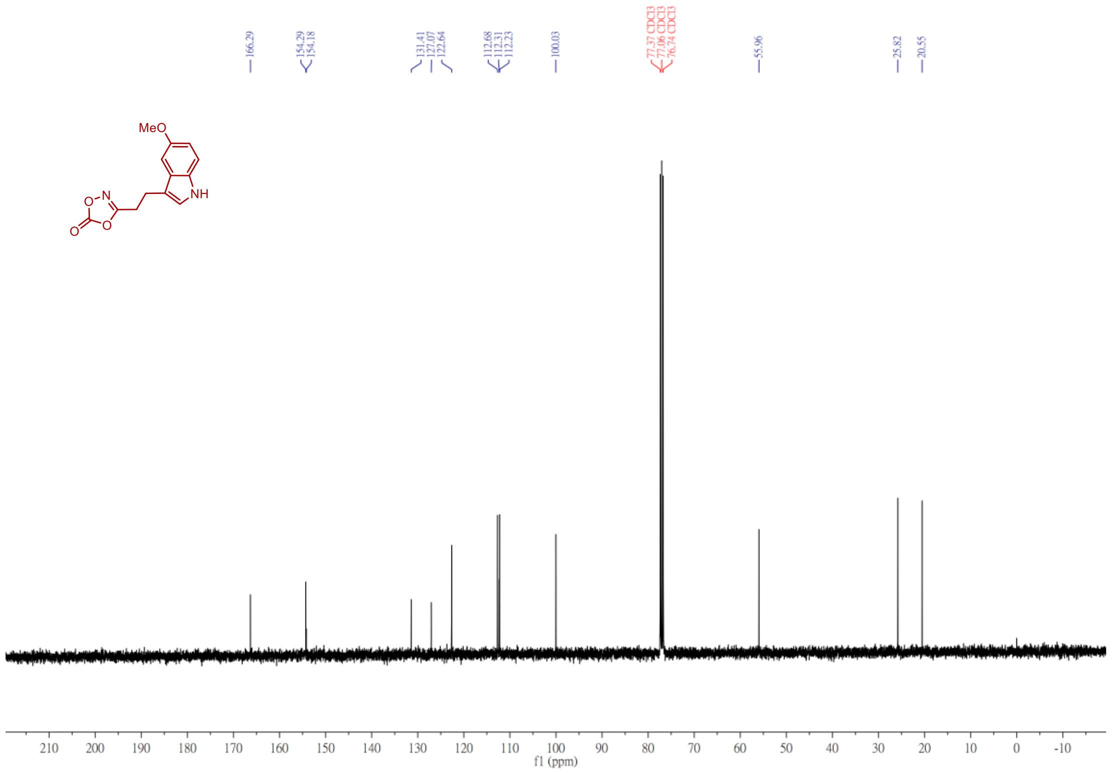

^{1}H and ^{13}C NMR spectrum of **S12**

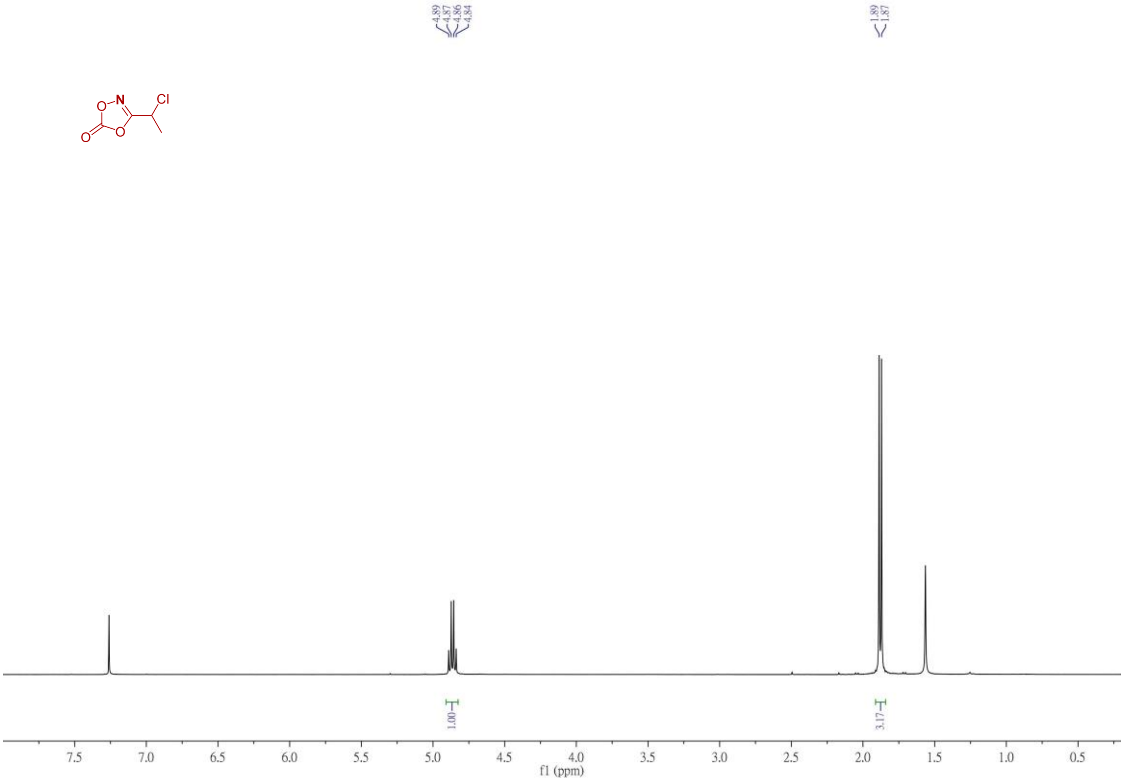

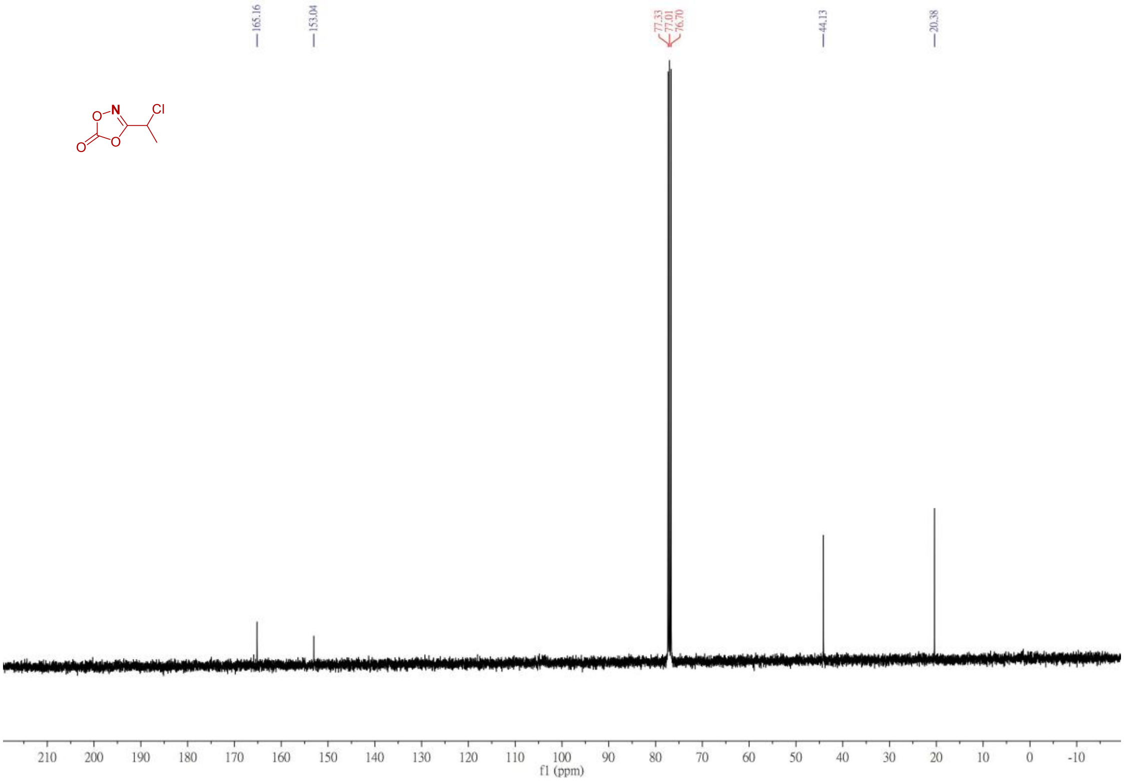

^{1}H and ^{13}C NMR spectrum of **S13**

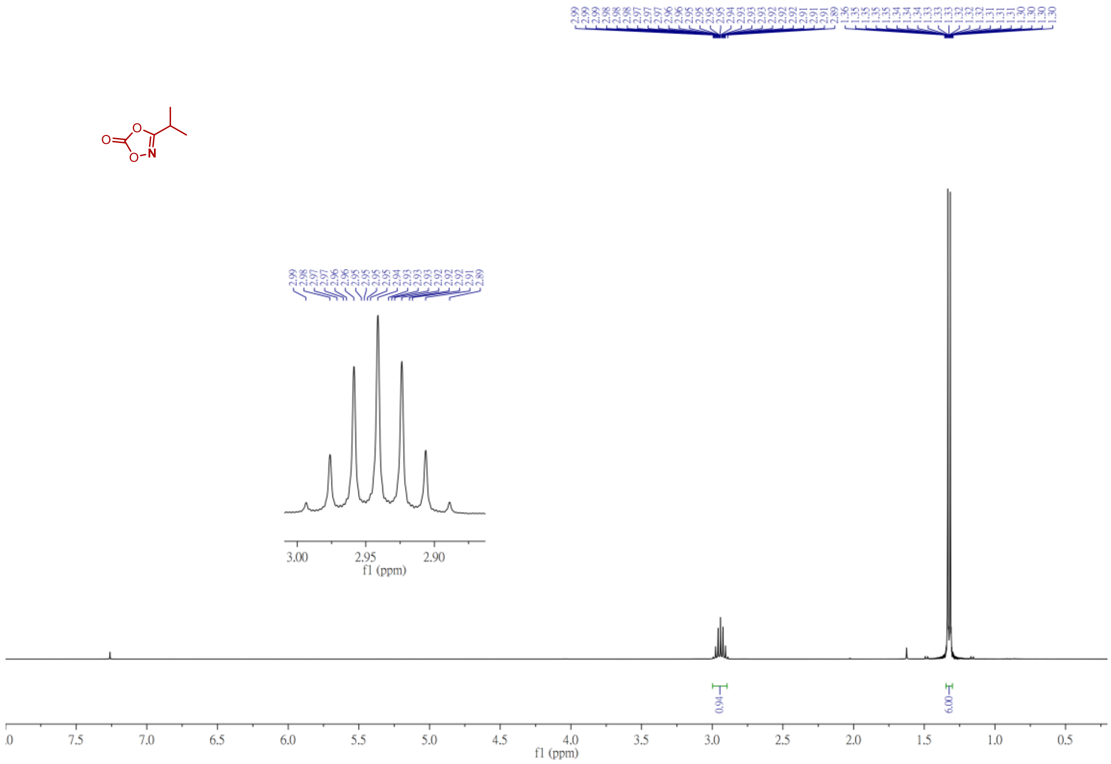

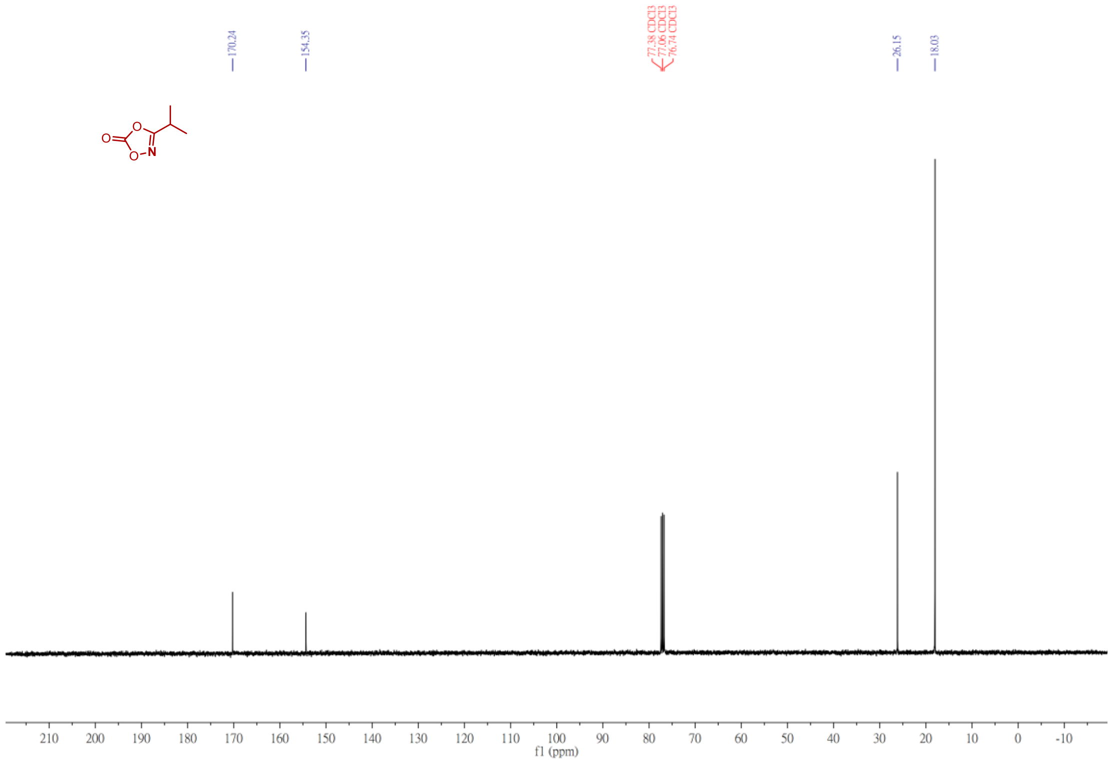

^{1}H and ^{13}C NMR spectrum of **S14**

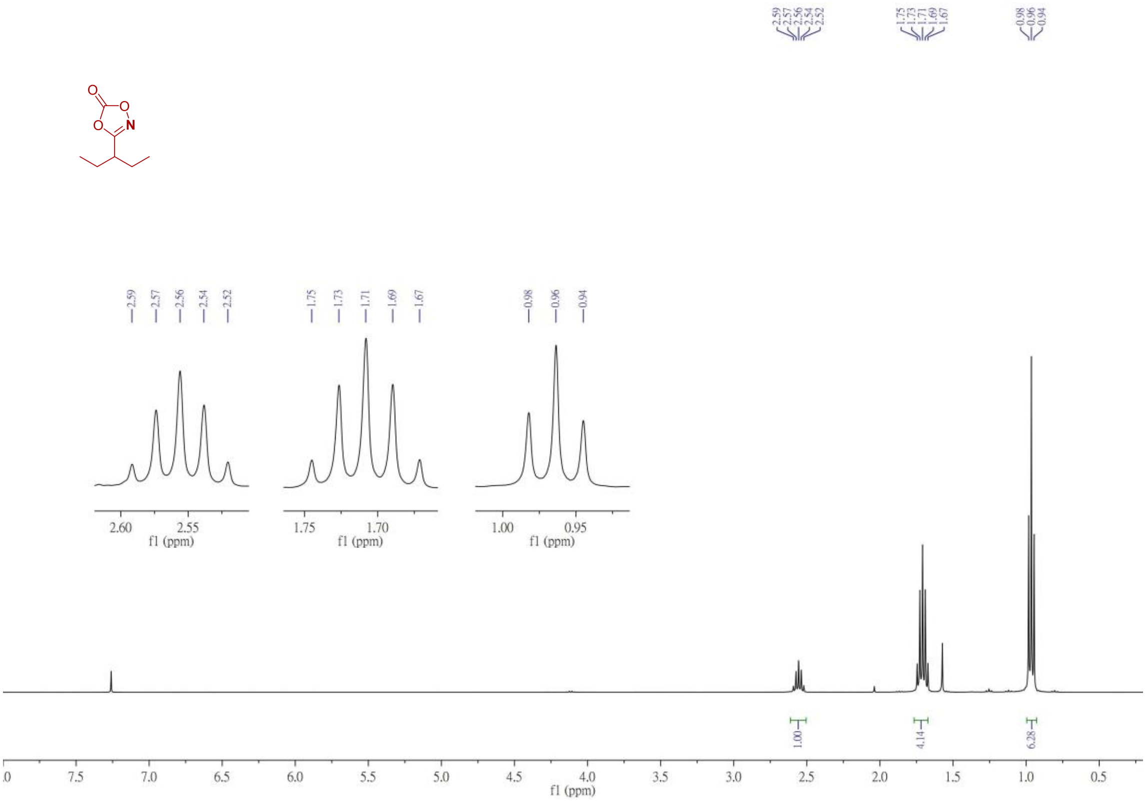

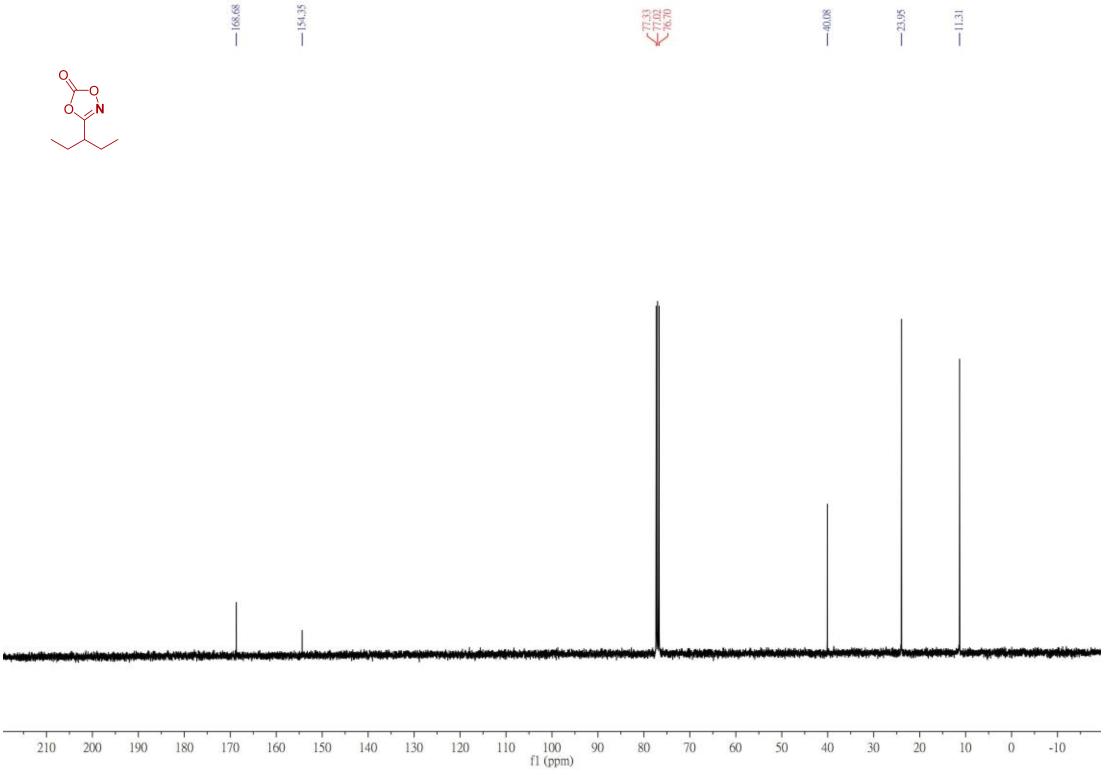

^{1}H and ^{13}C NMR spectrum of **62**

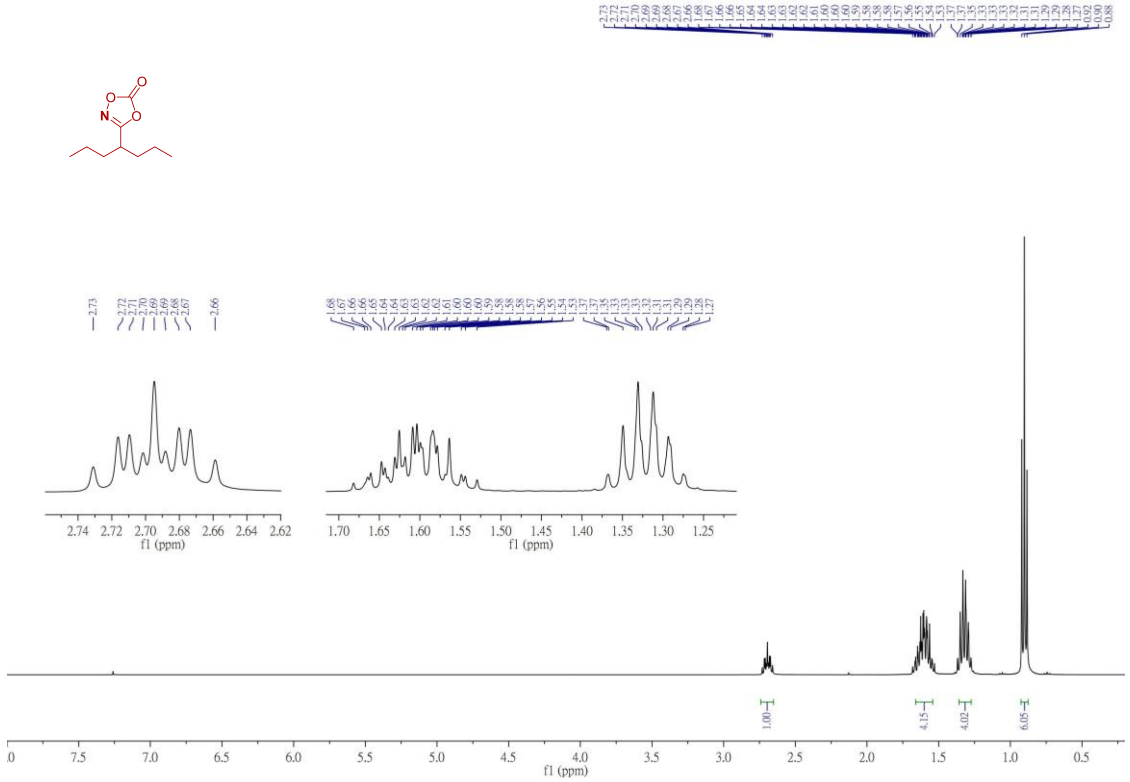

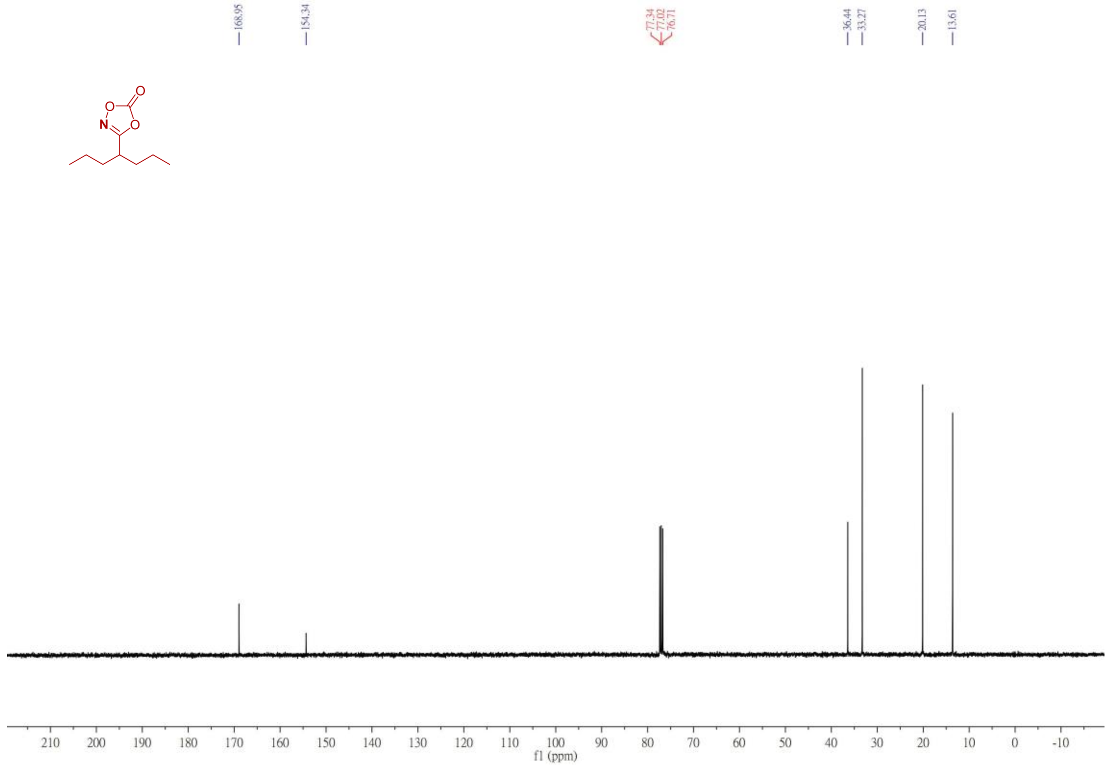

^{1}H and ^{13}C NMR spectrum of **S15**

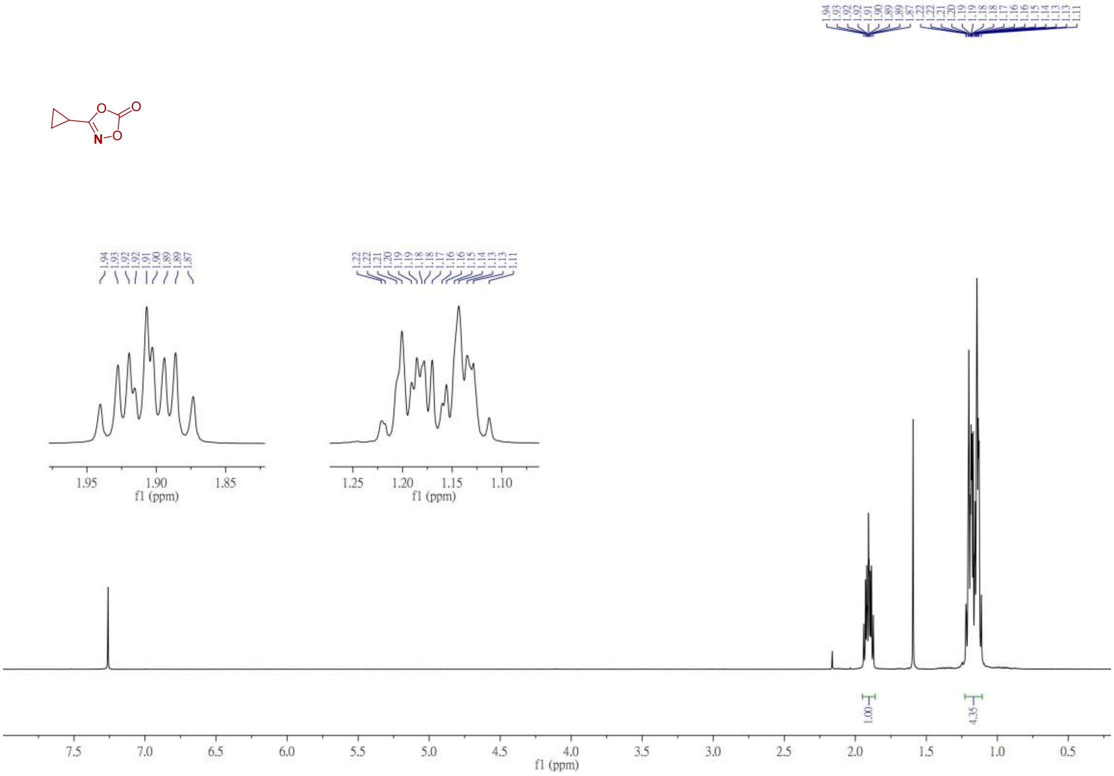

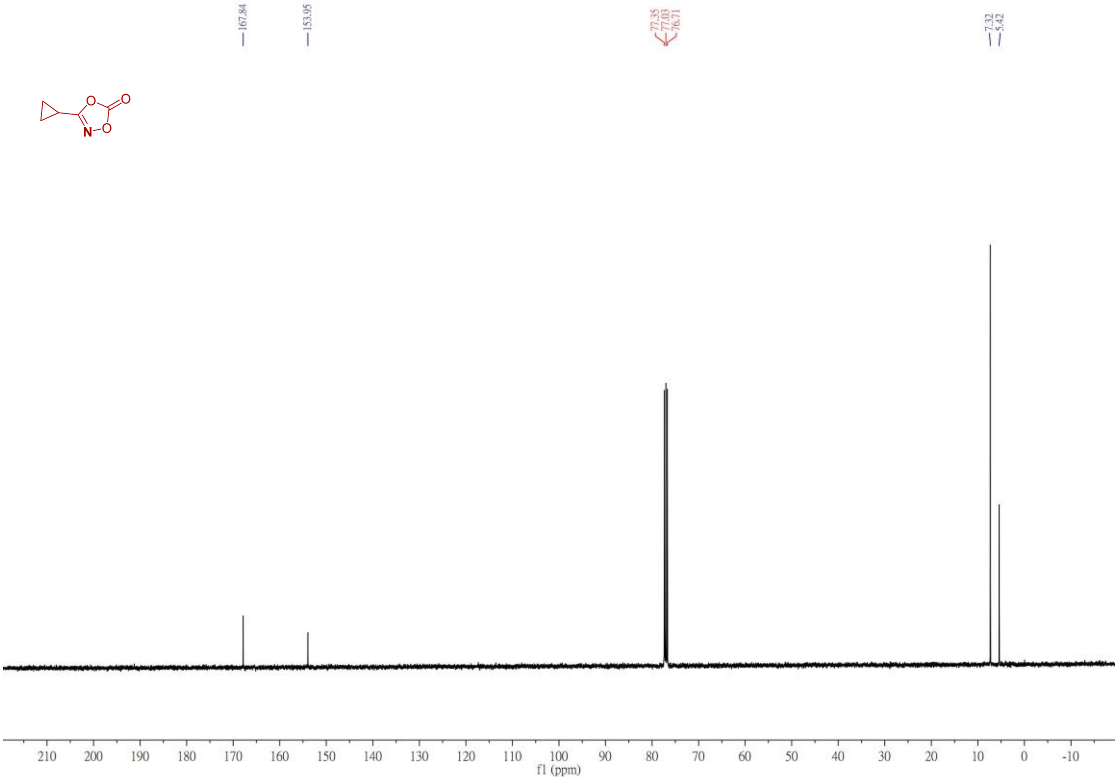

H and ^{13}C NMR spectrum of **2**

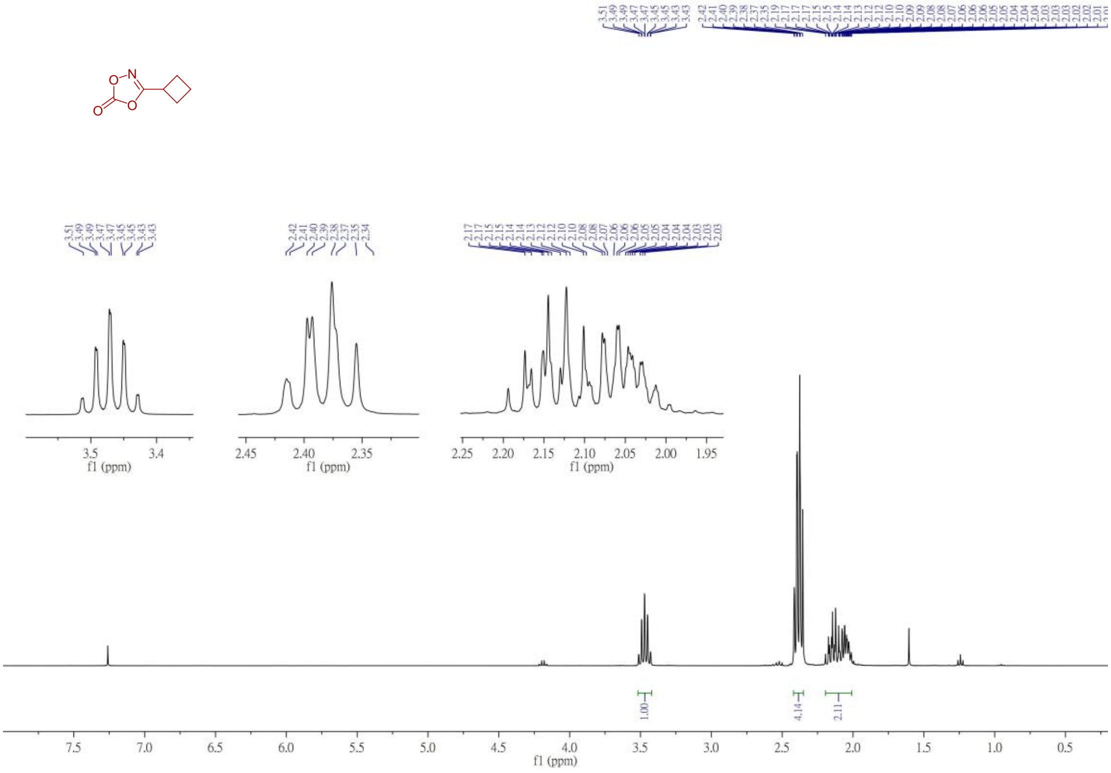

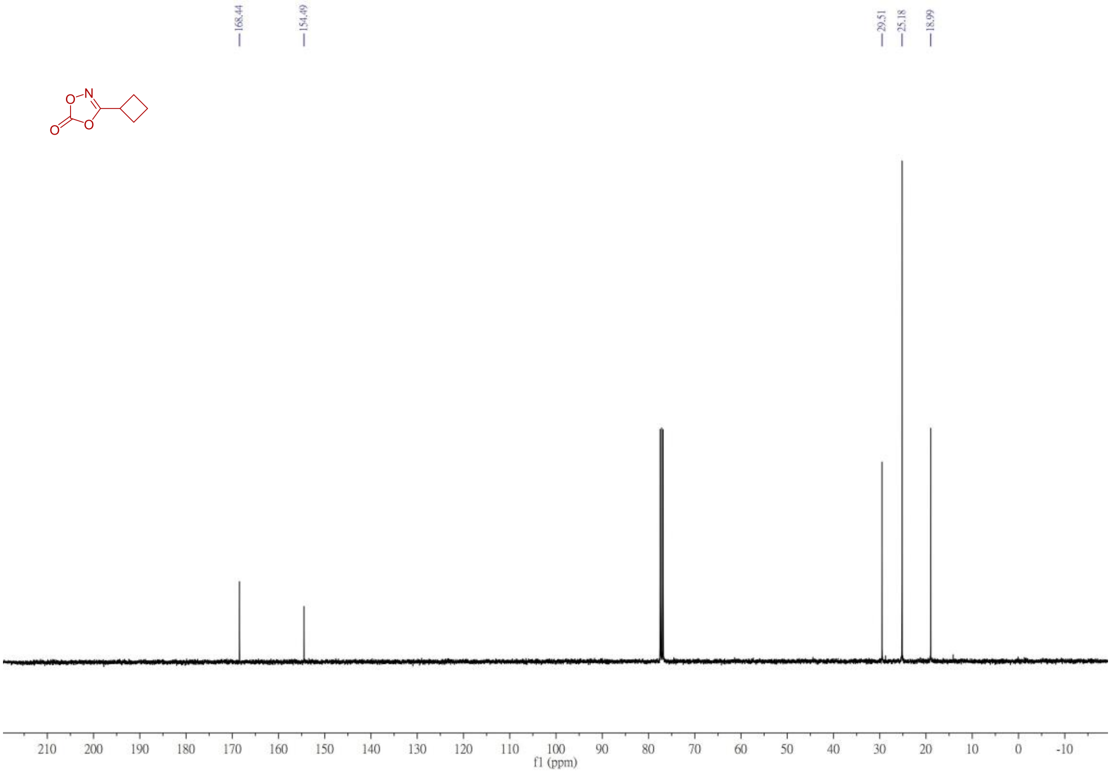

^{1}H and ^{13}C NMR spectrum of **S16**

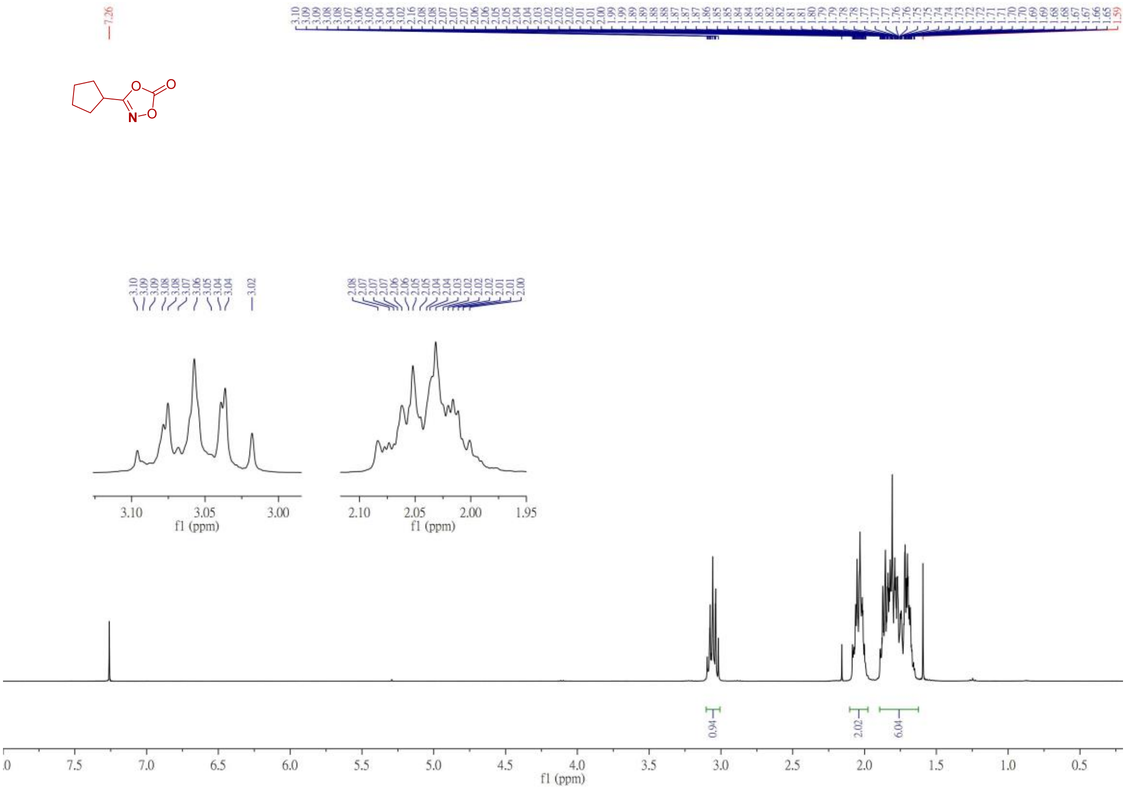

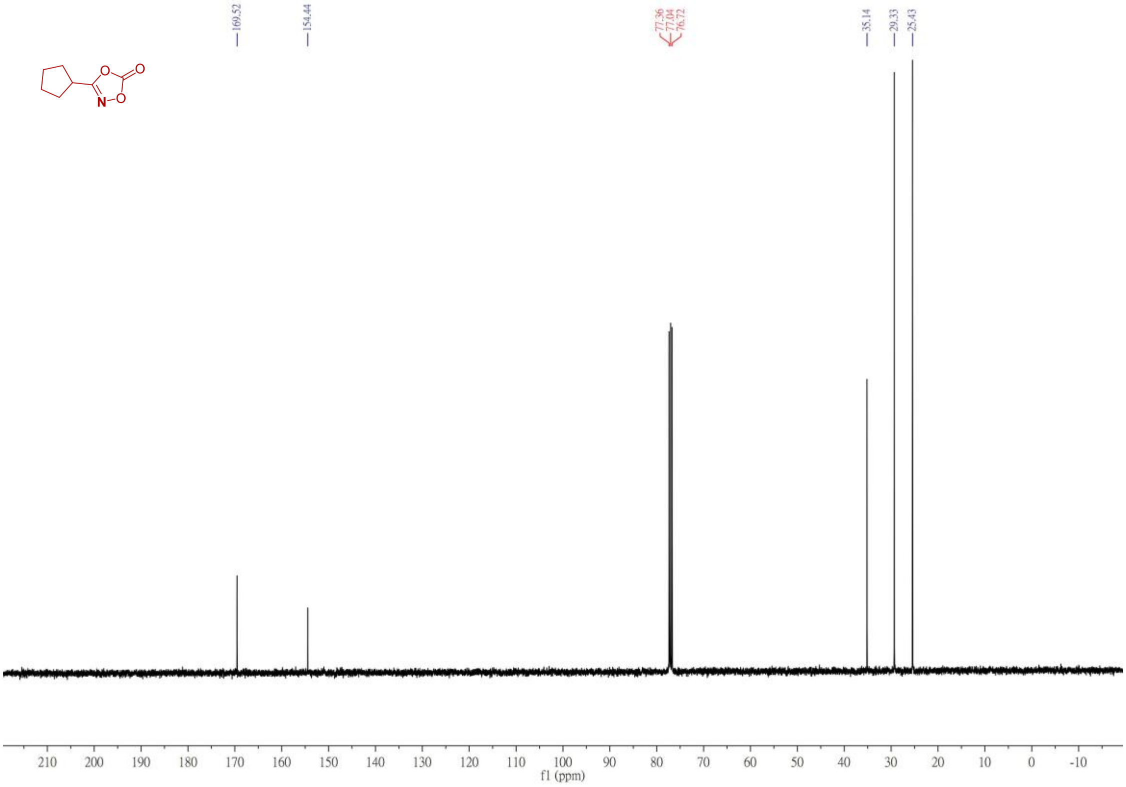

^{1}H and ^{13}C NMR spectrum of **S17**

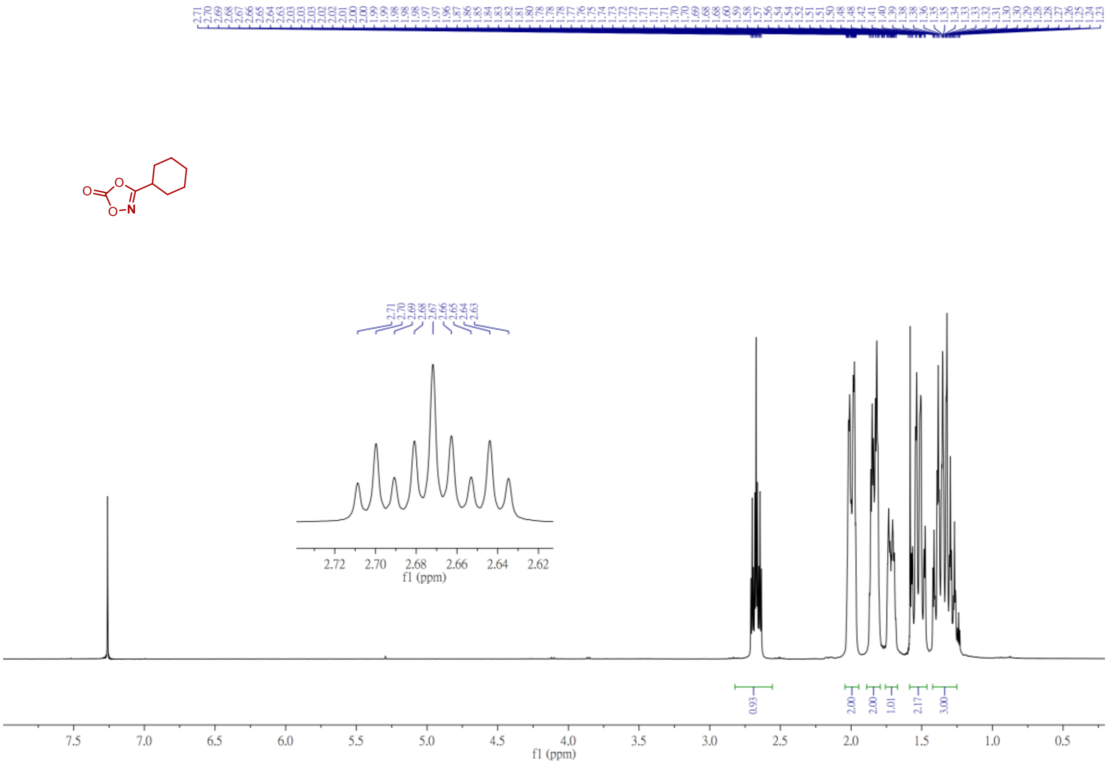

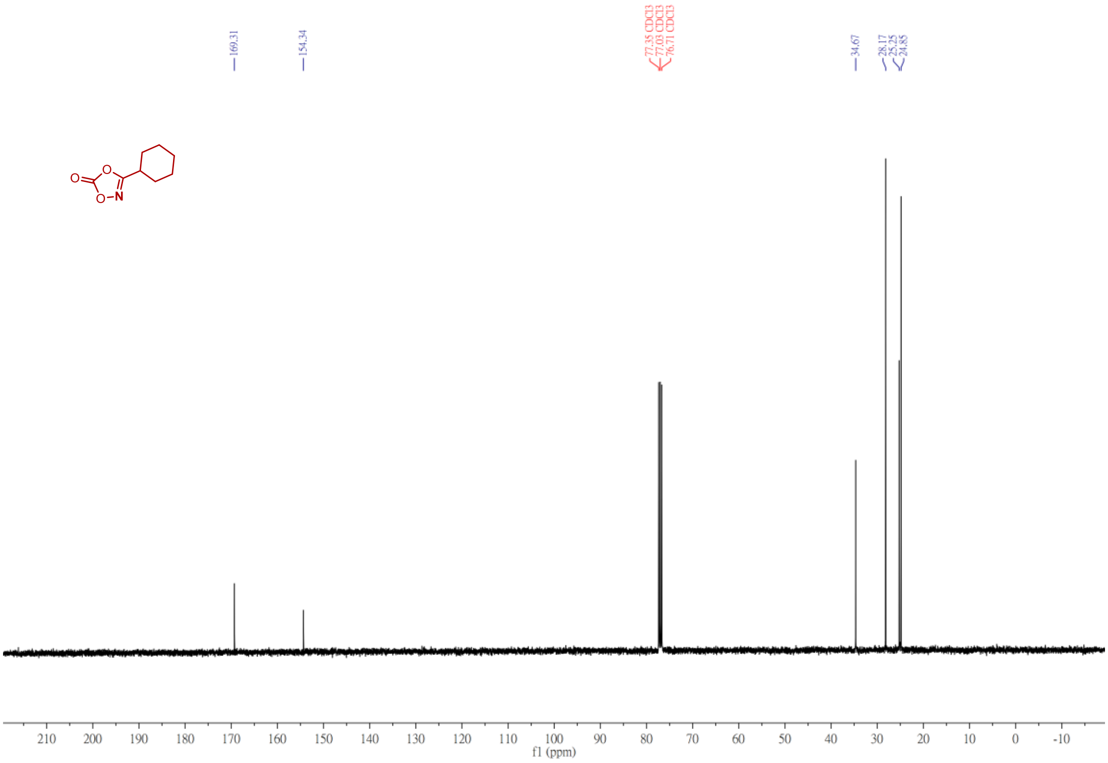

^{1}H and ^{13}C NMR spectrum of **S18**

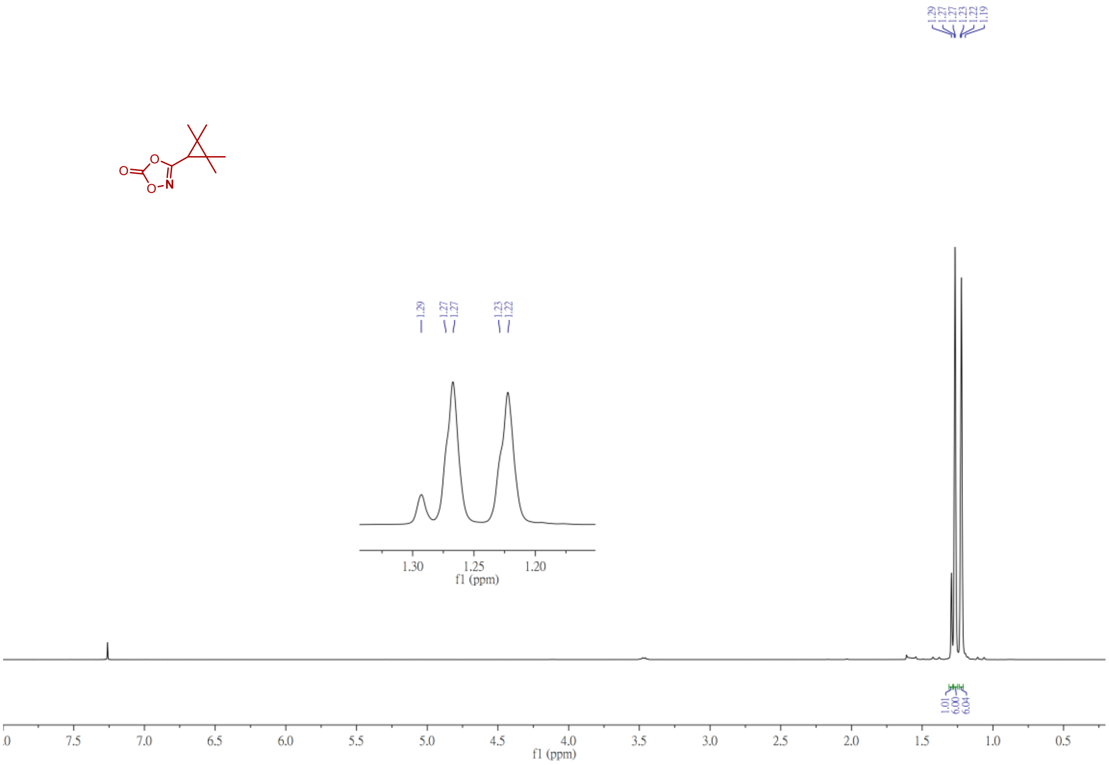

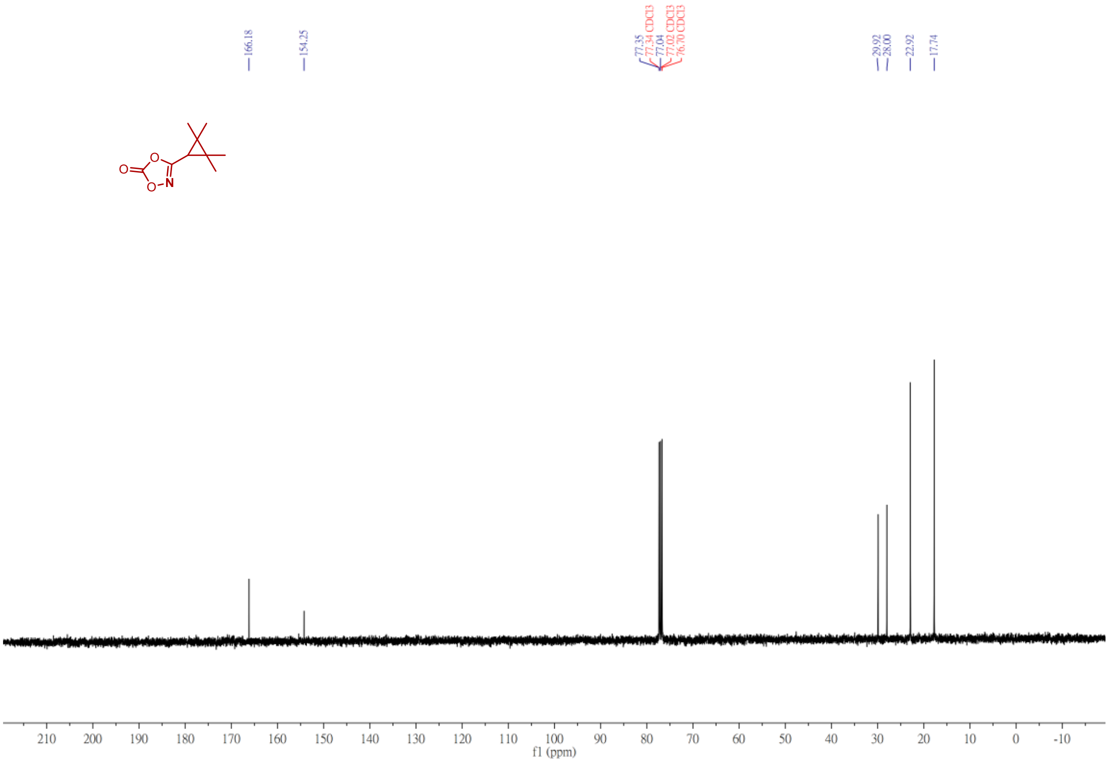

^1H and ^{13}C NMR spectrum of **S19**

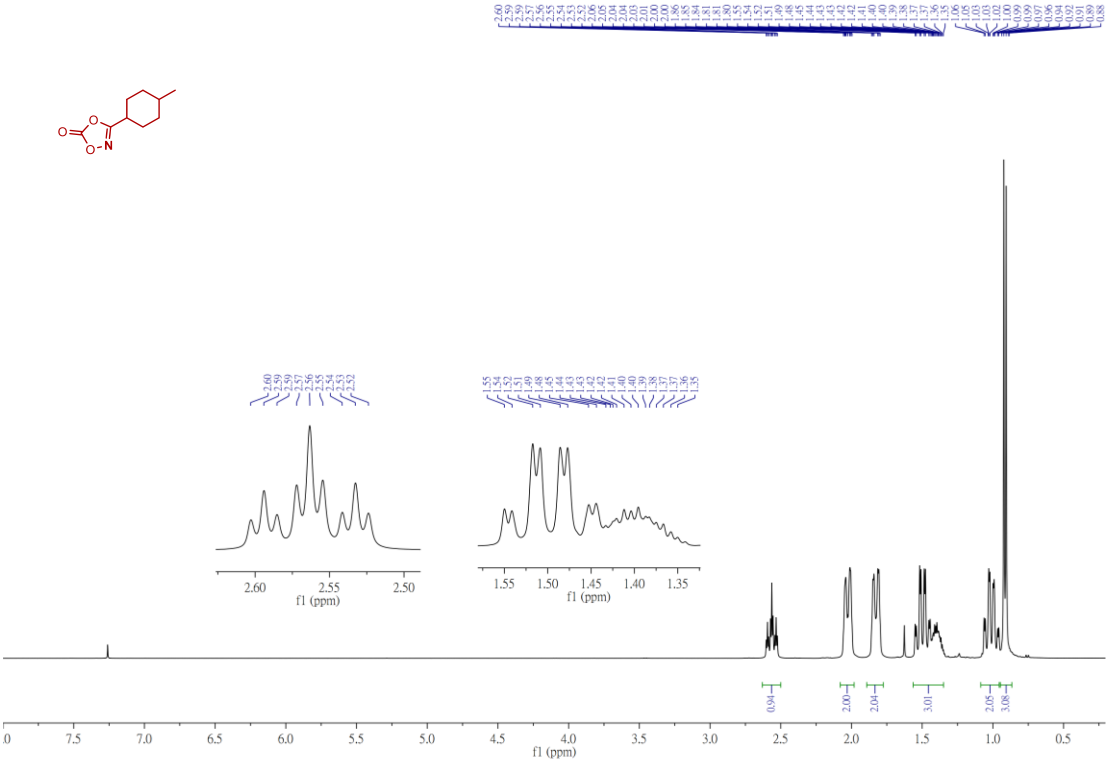

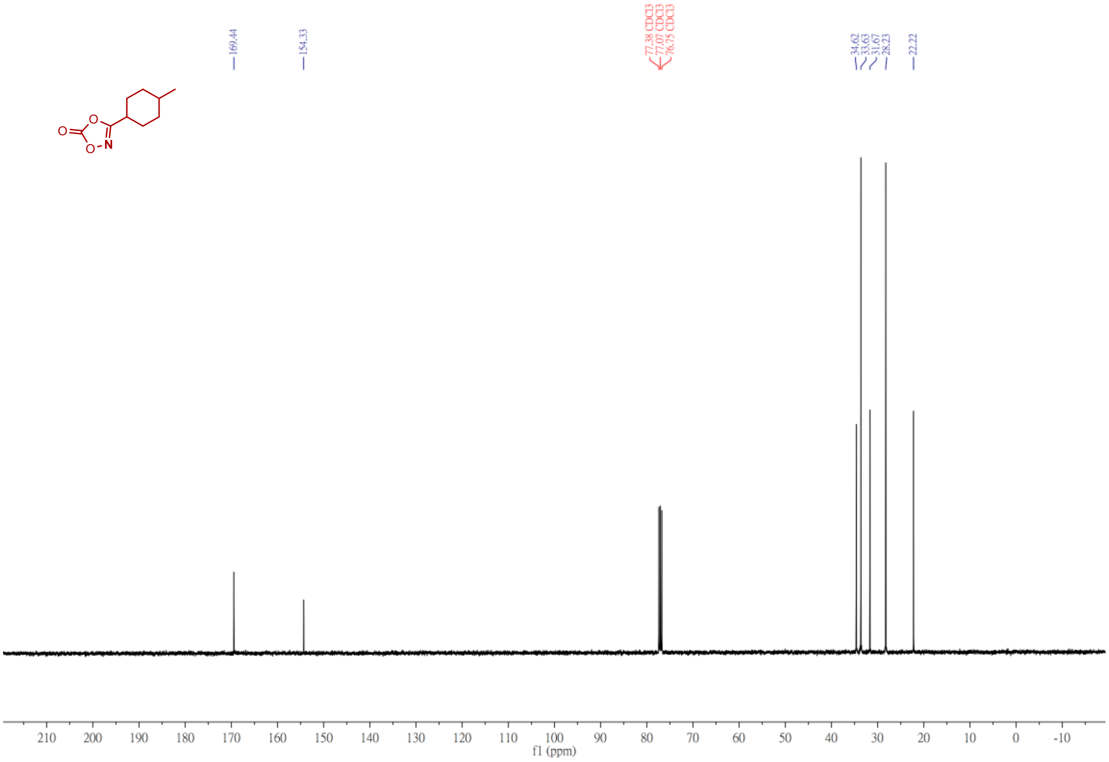

^{1}H and ^{13}C NMR spectrum of **S20**

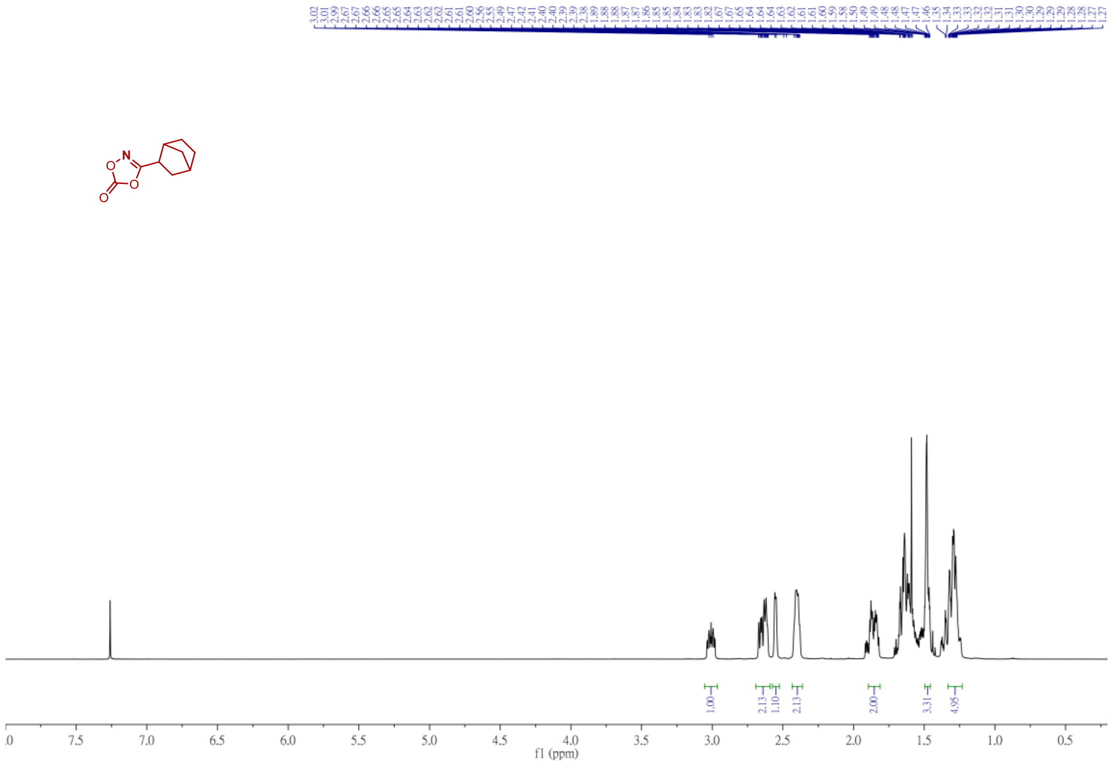

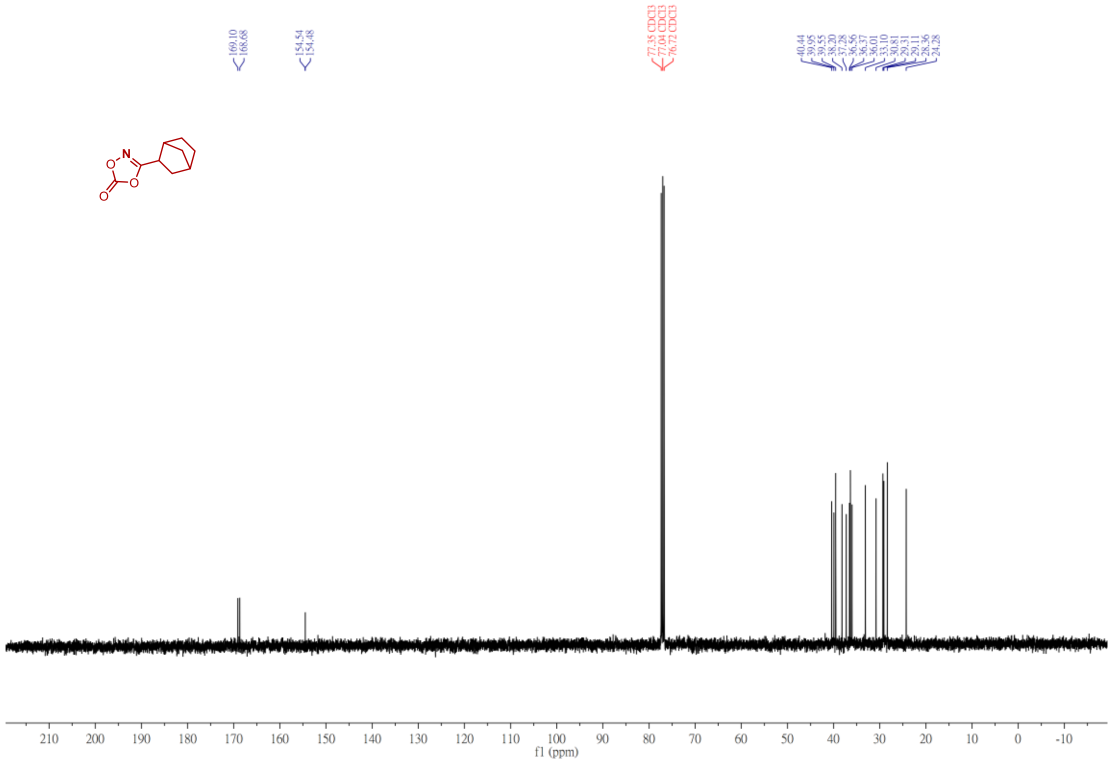

^{1}H and ^{13}C NMR spectrum of **S21**

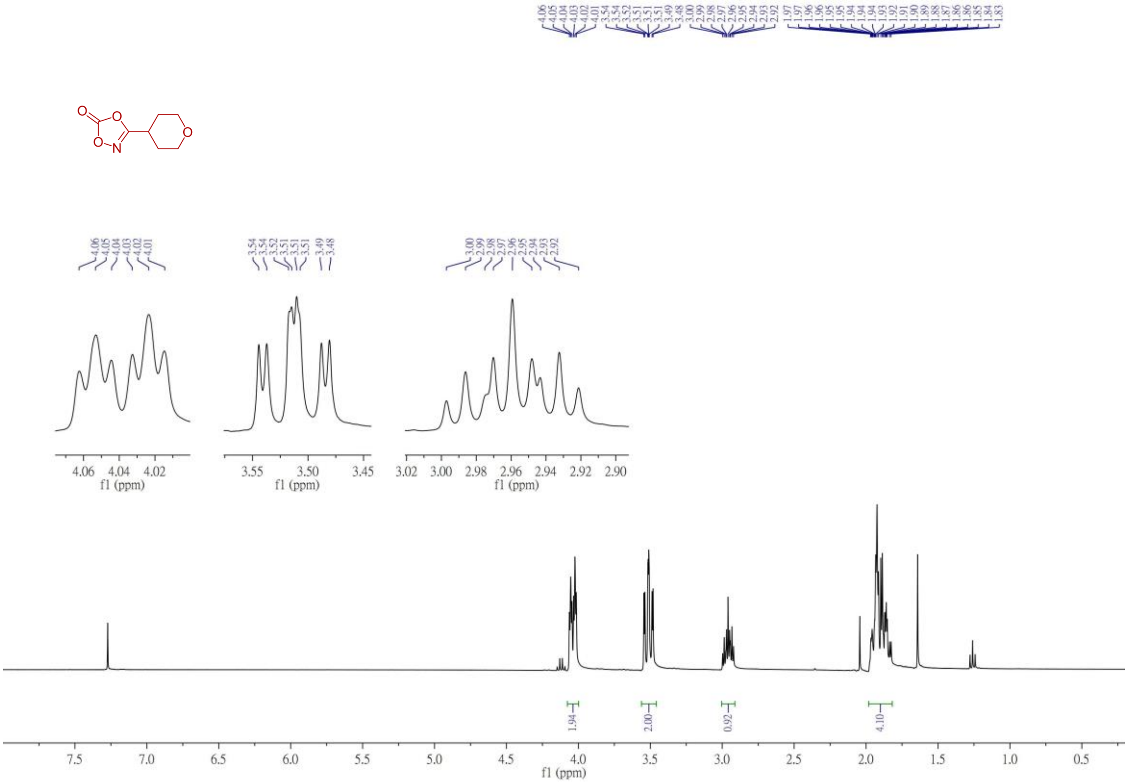

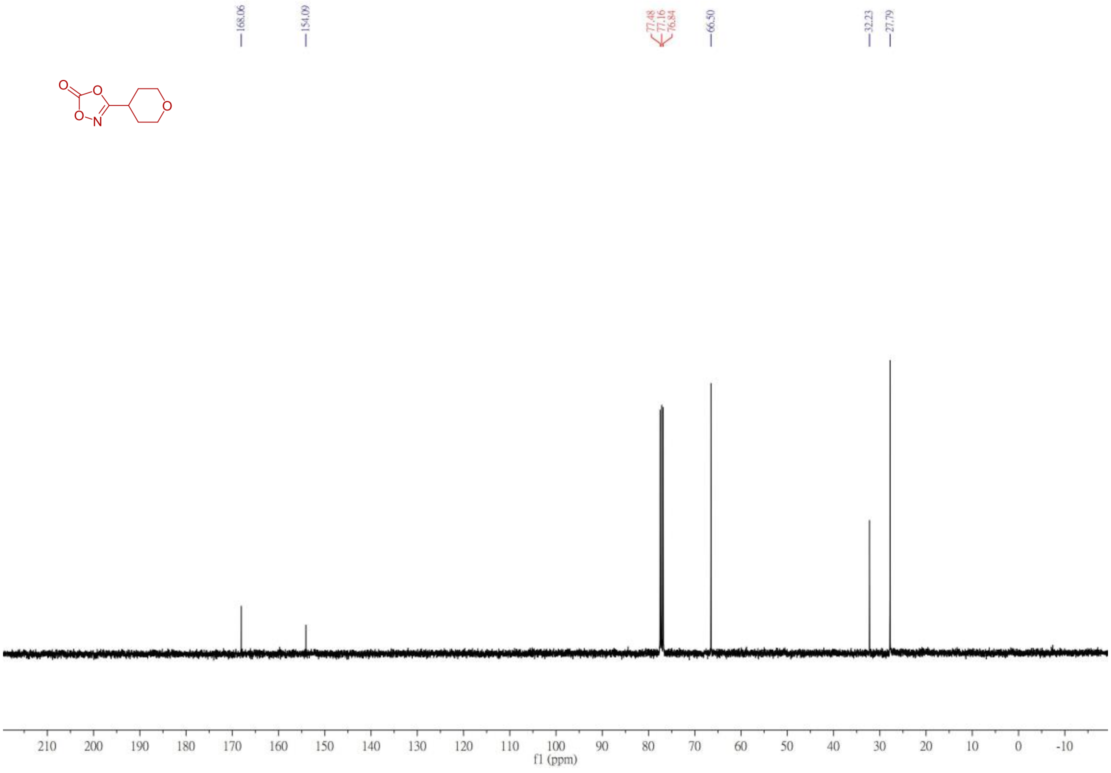

^{1}H and ^{13}C NMR spectrum of **S22**

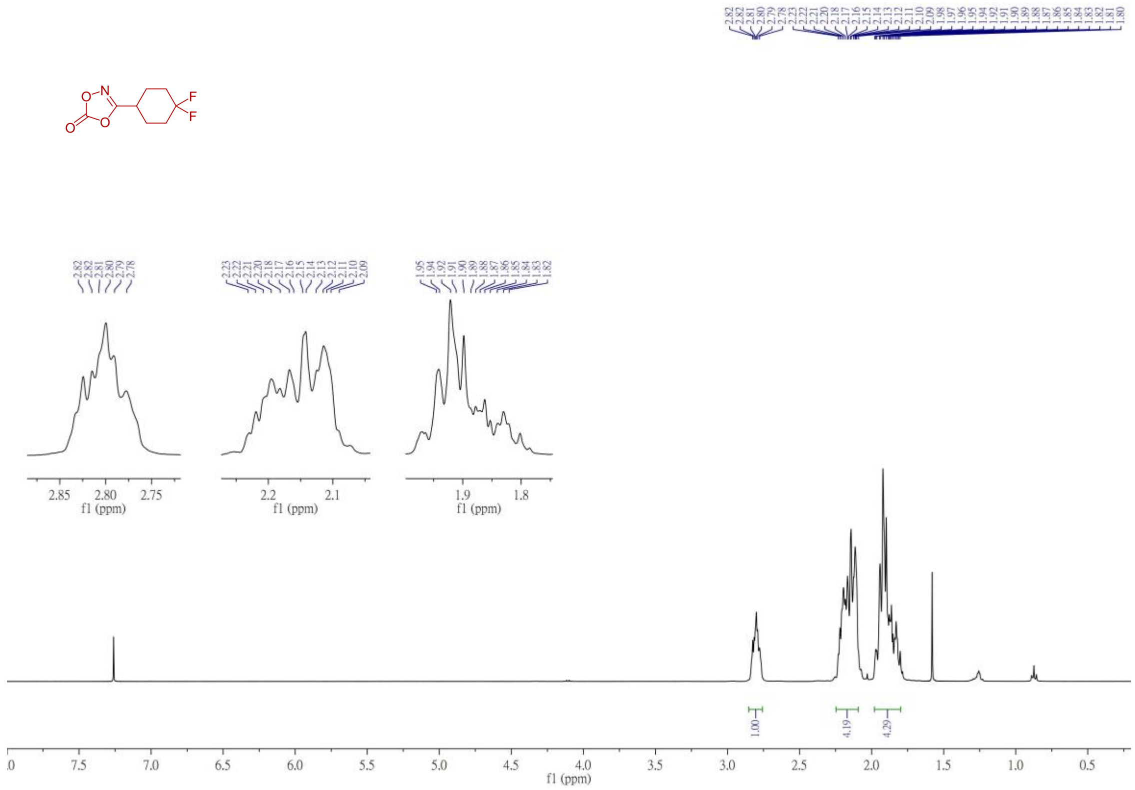

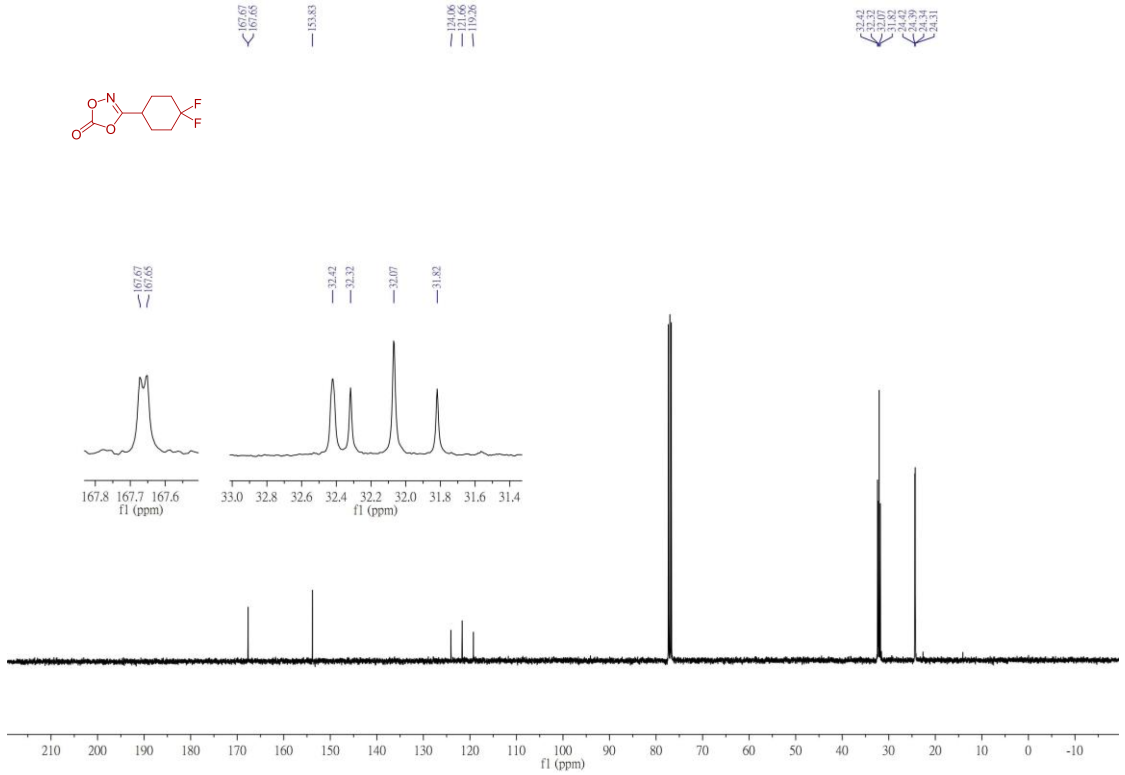

^{19}F NMR spectrum of **S22**

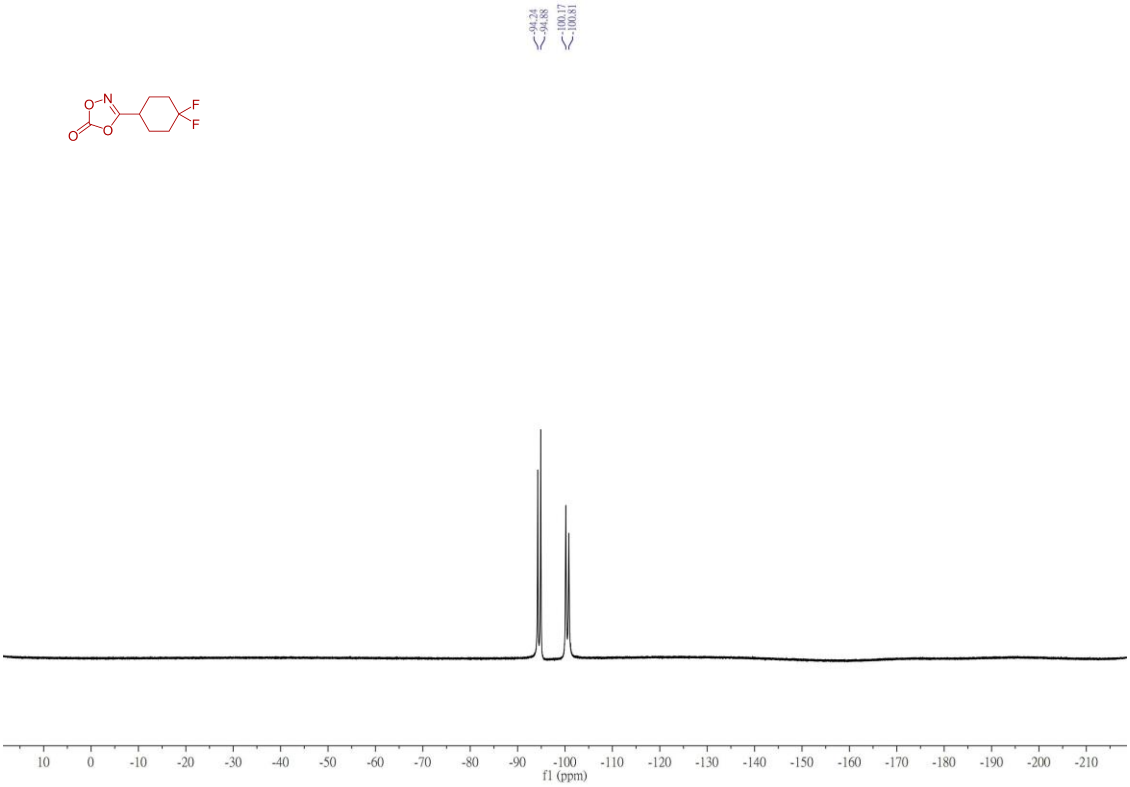

^{1}H and ^{13}C NMR spectrum of **114**

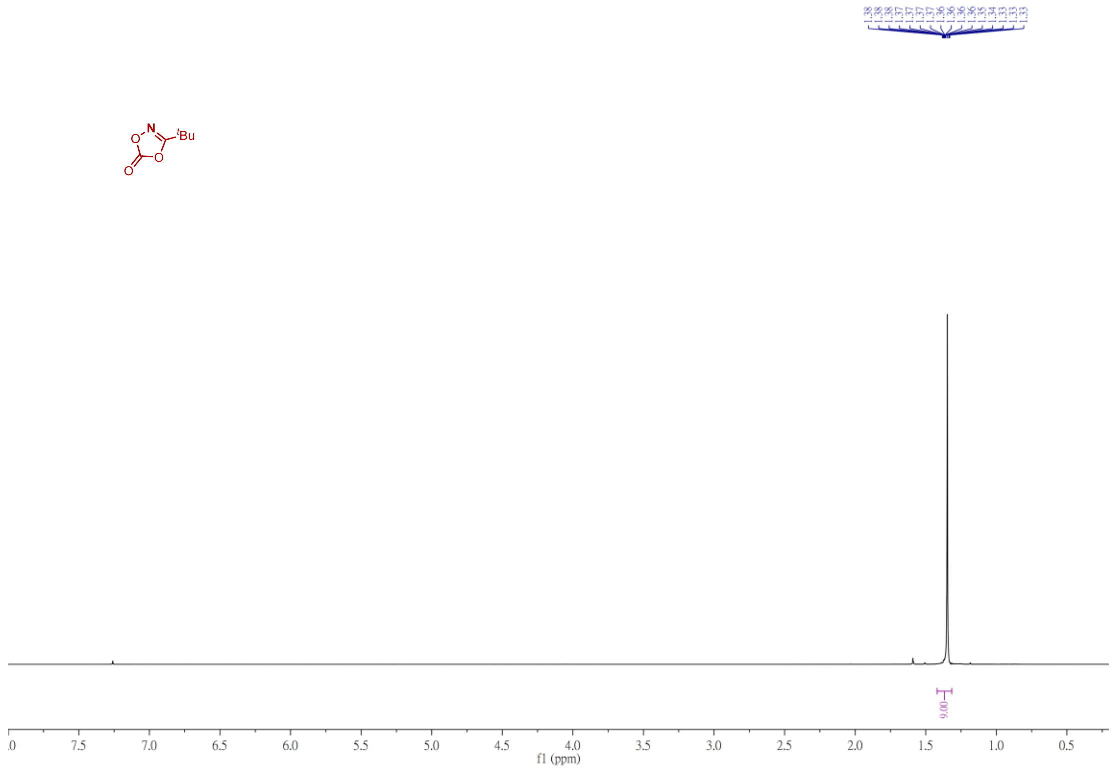

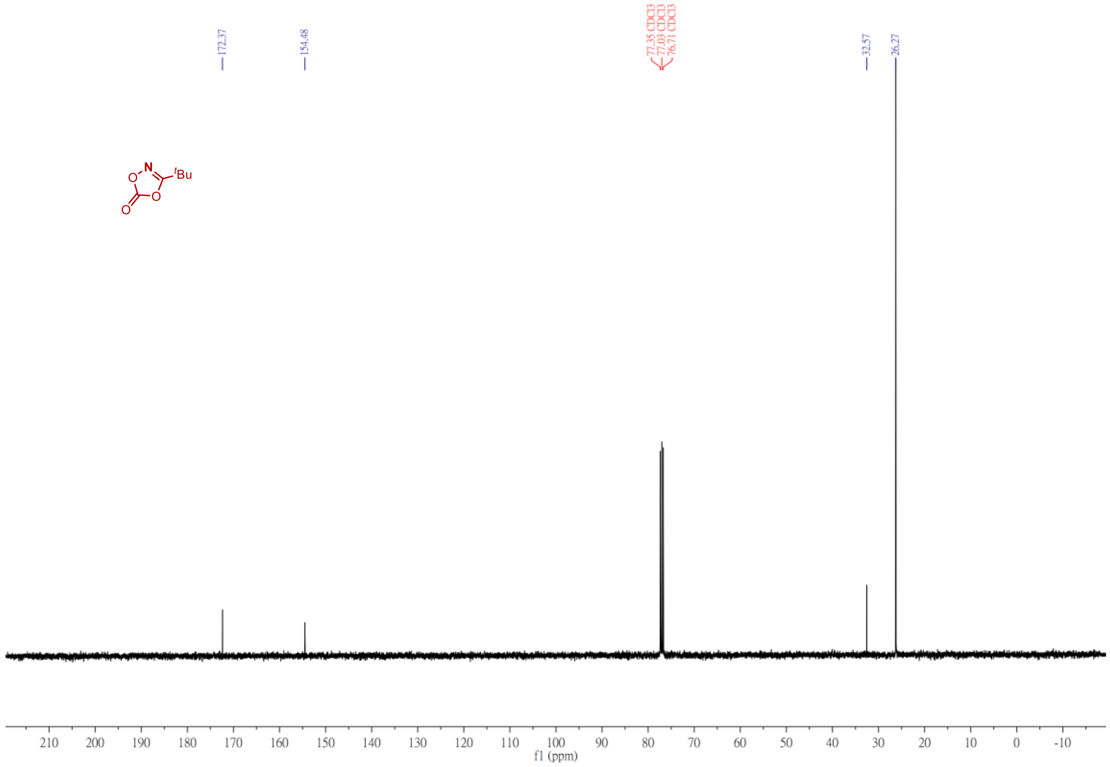

^{1}H and ^{13}C NMR spectrum of **S23**

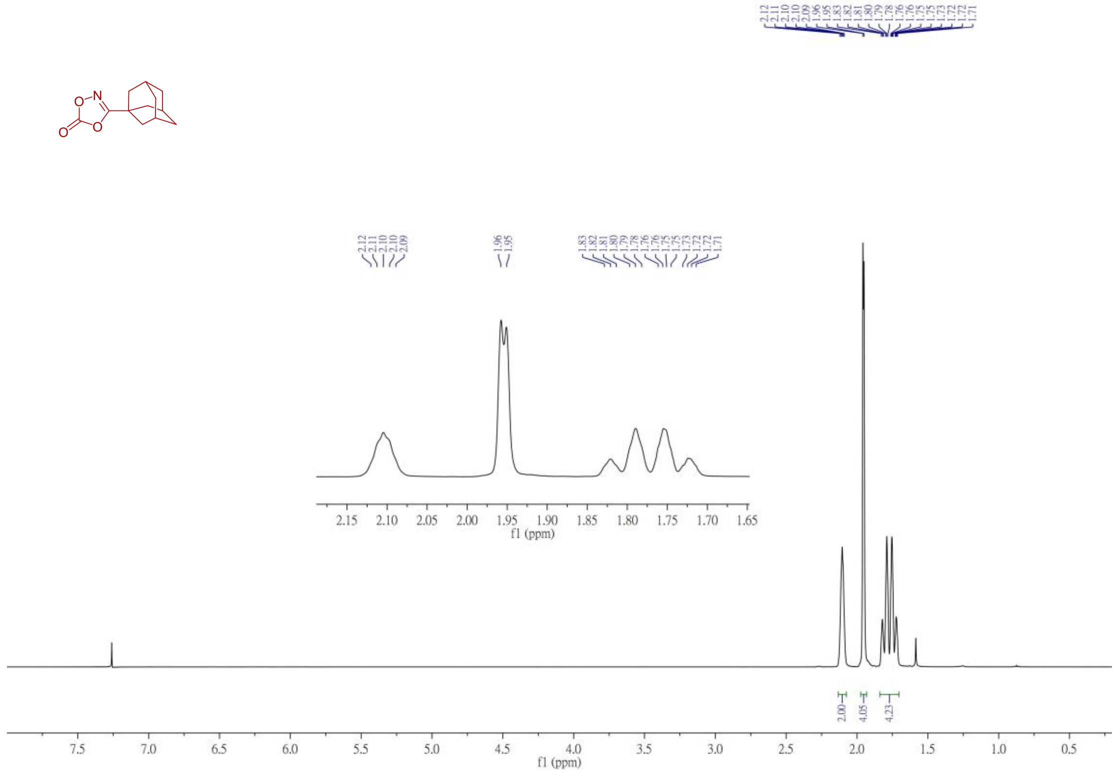

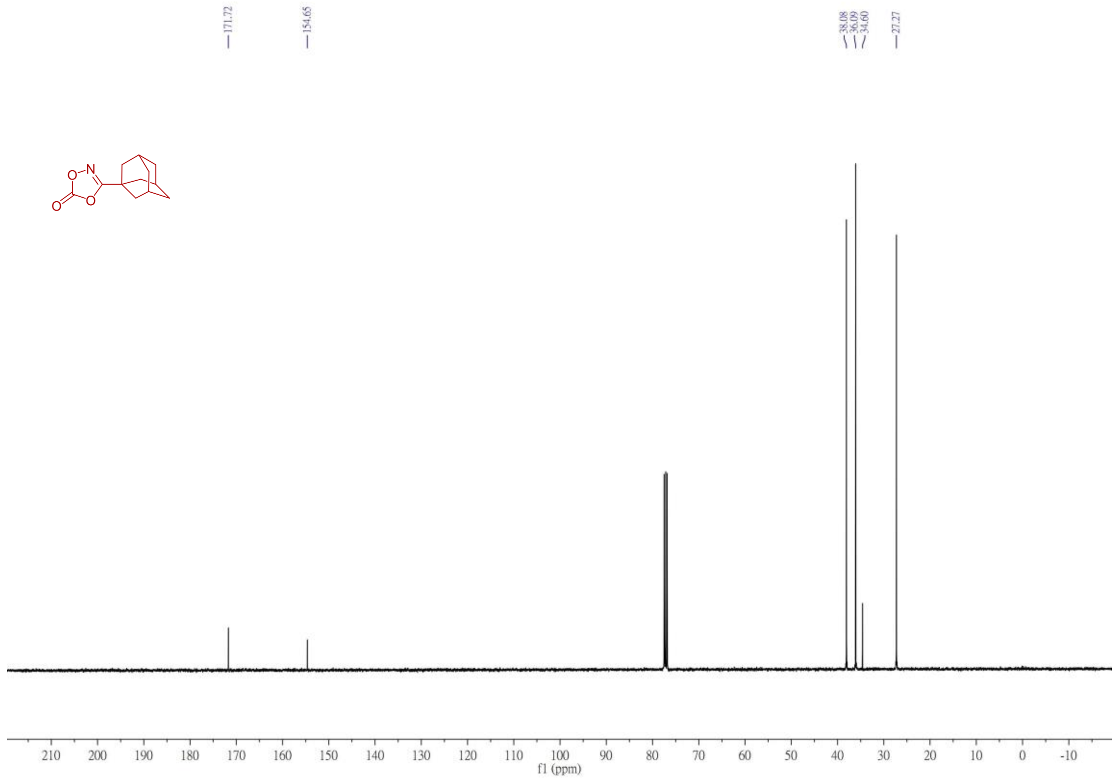

^1H and ^{13}C NMR spectrum of **S24**

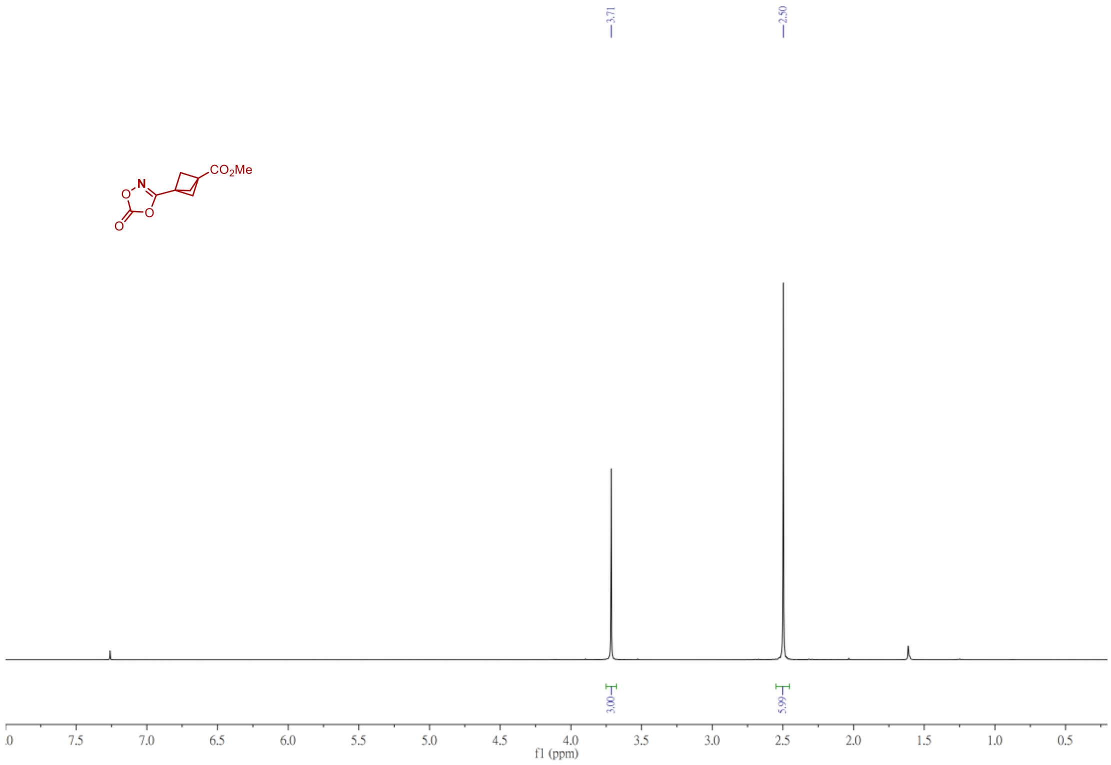

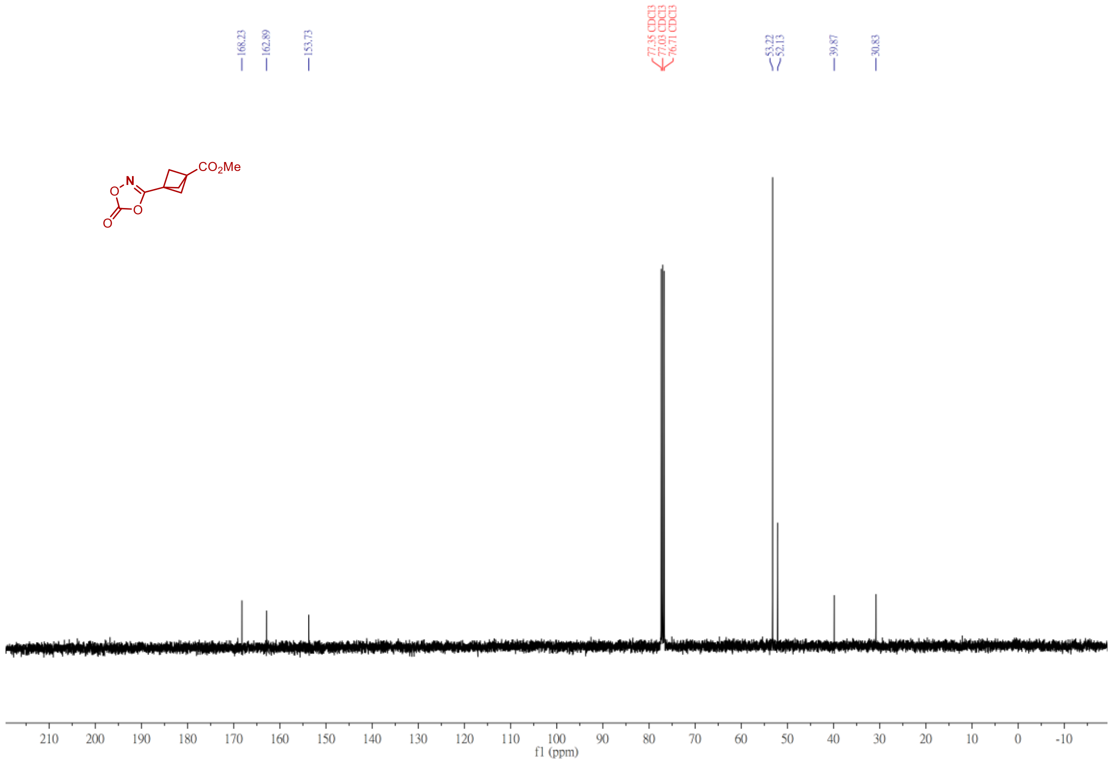

^{1}H and ^{13}C NMR spectrum of **S25**

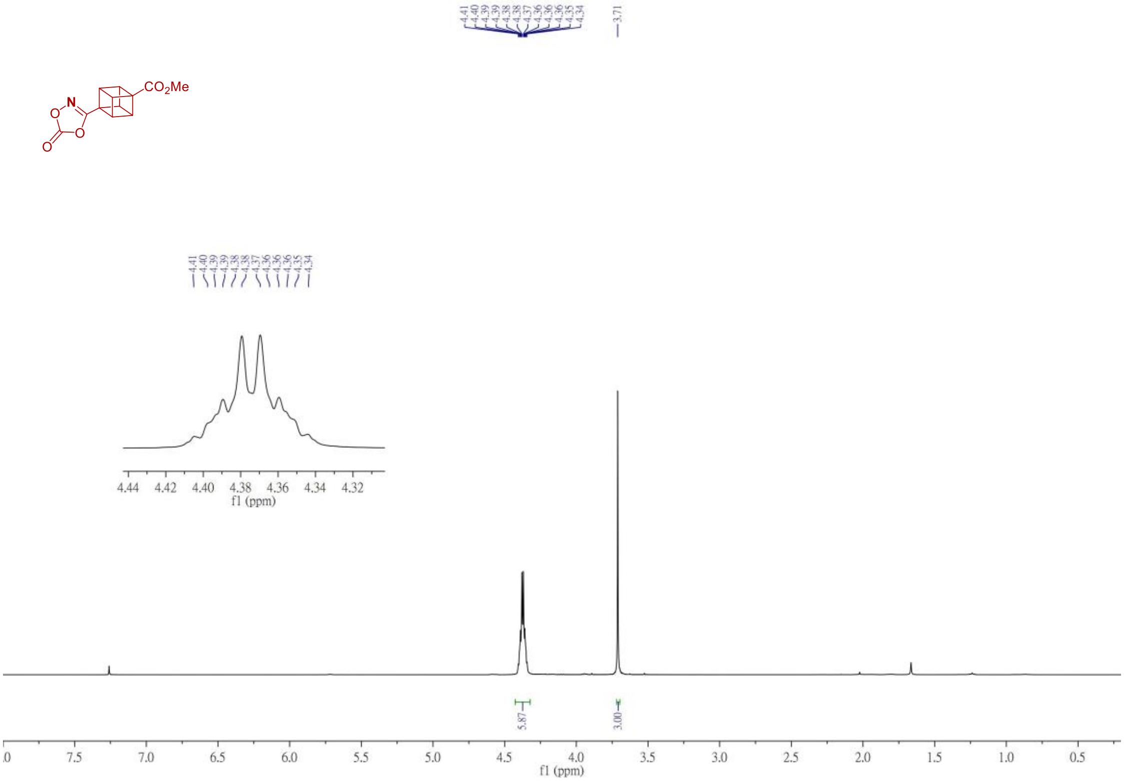

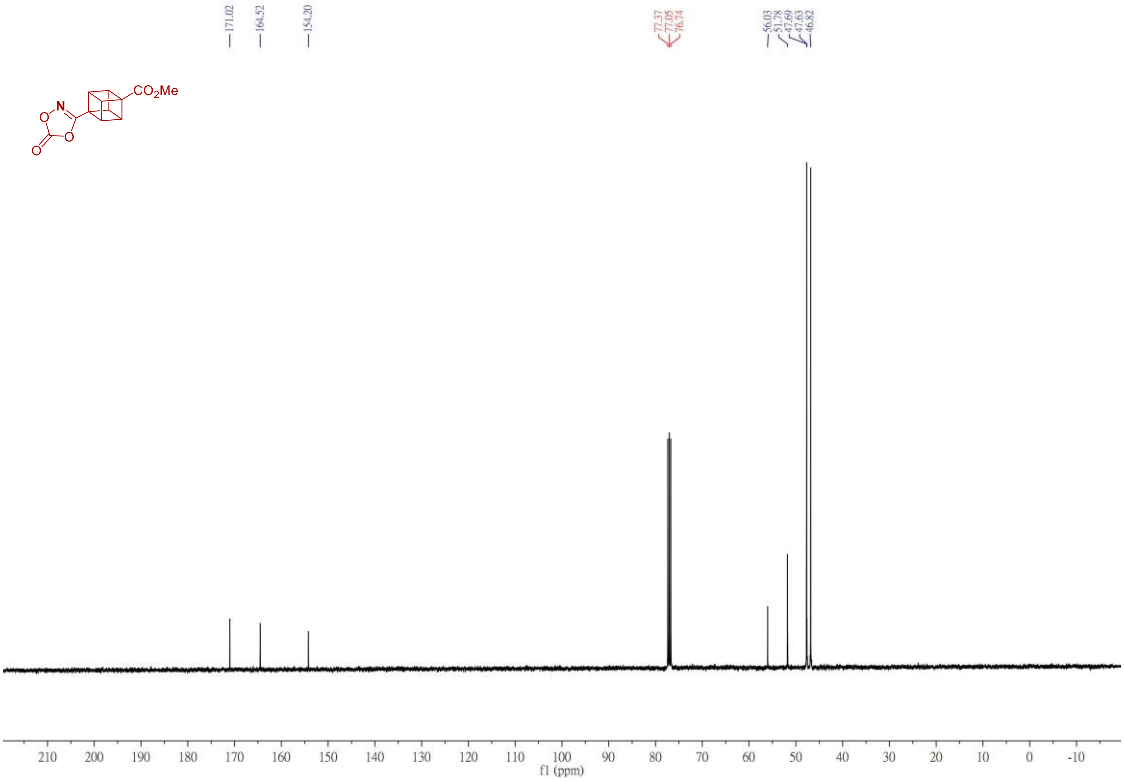

^{1}H and ^{13}C NMR spectrum of **125**

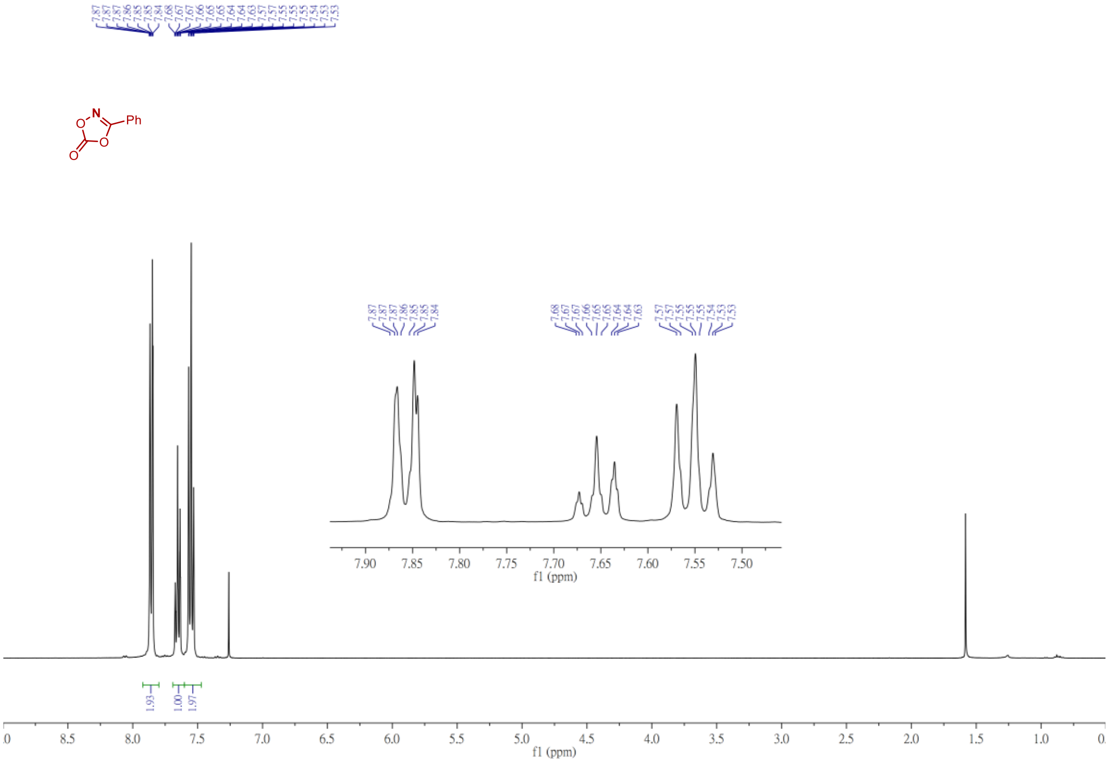

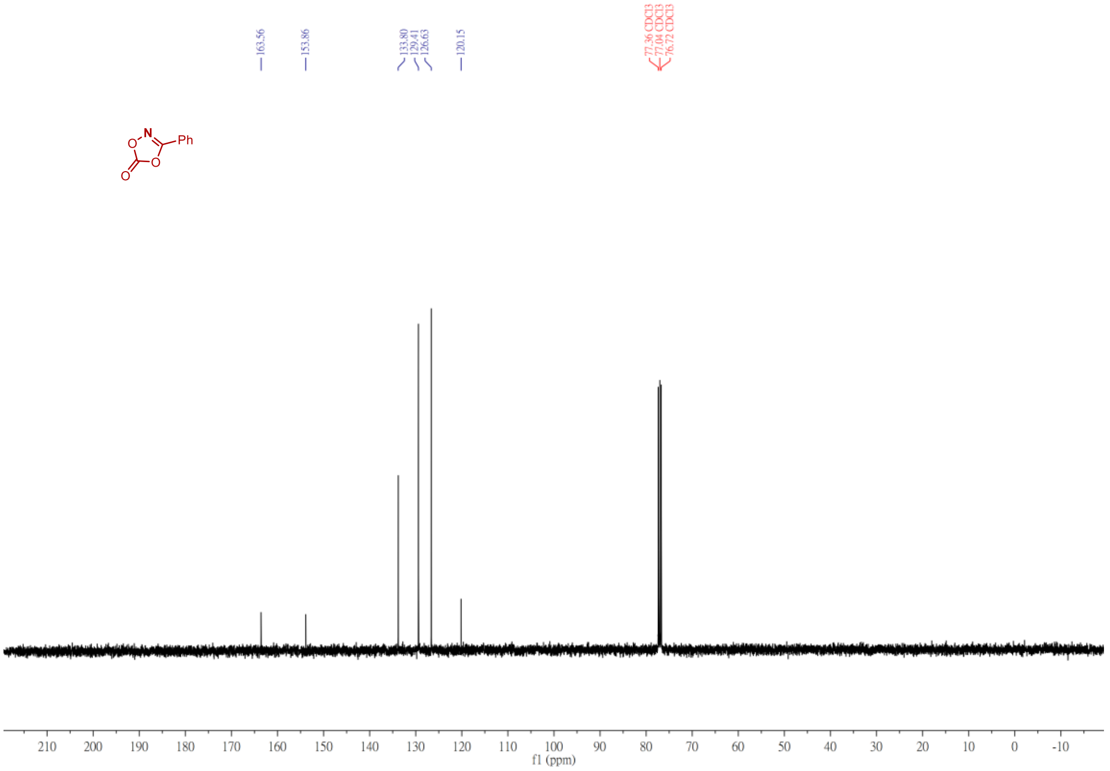

^{1}H and ^{13}C NMR spectrum of **S26**

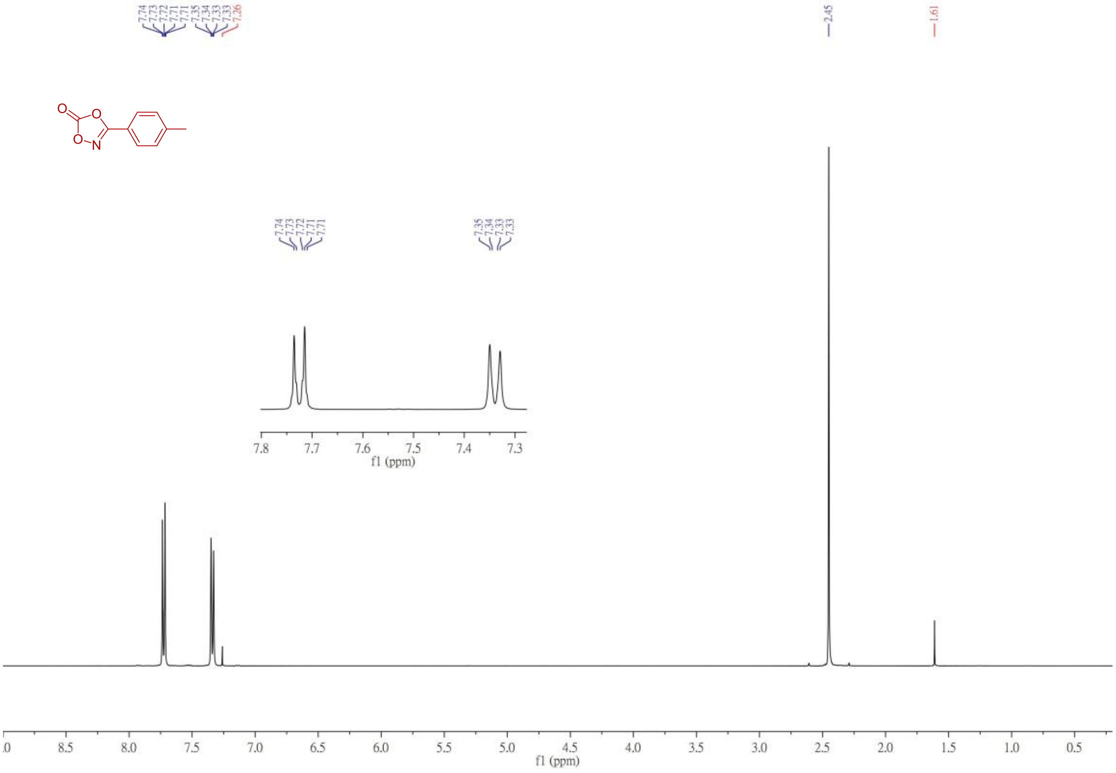

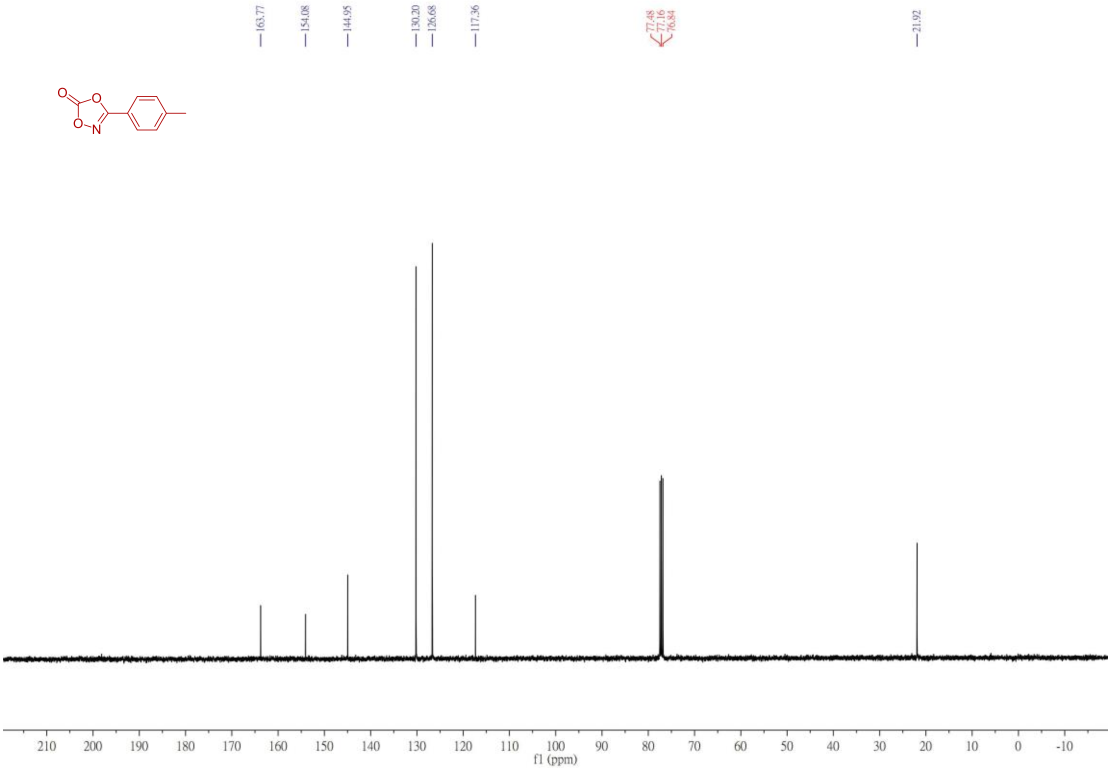

^{1}H and ^{13}C NMR spectrum of **S27**

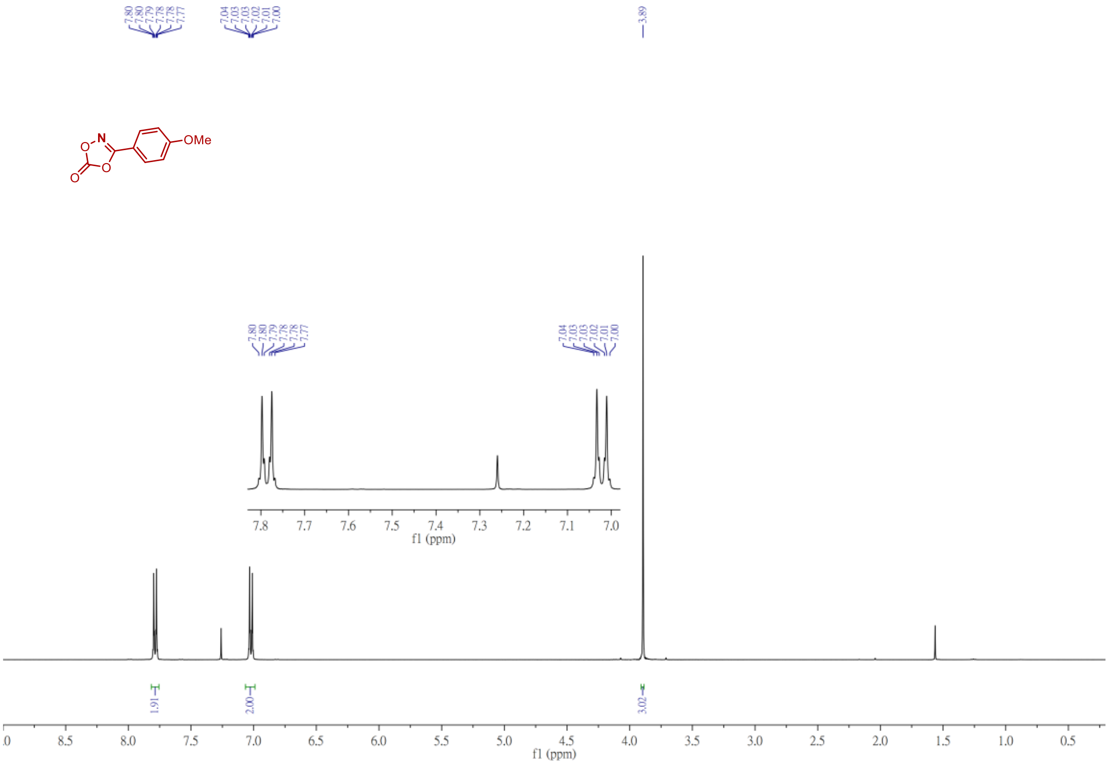

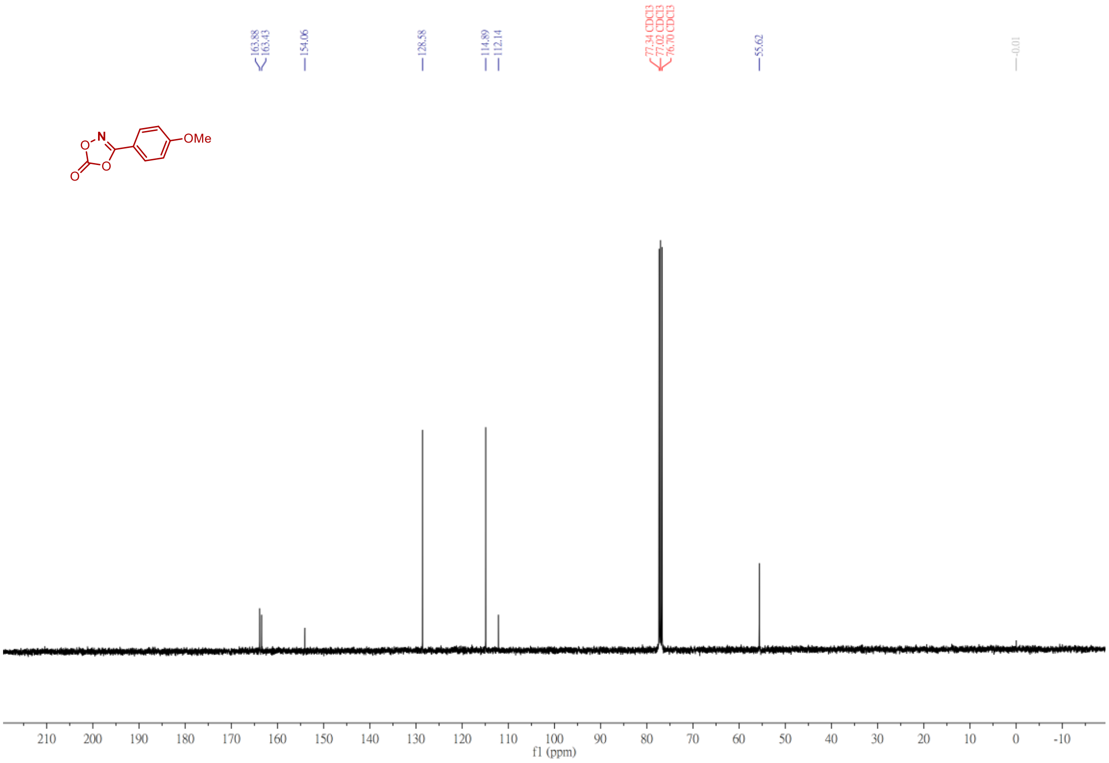

^{1}H and ^{13}C NMR spectrum of **S28**

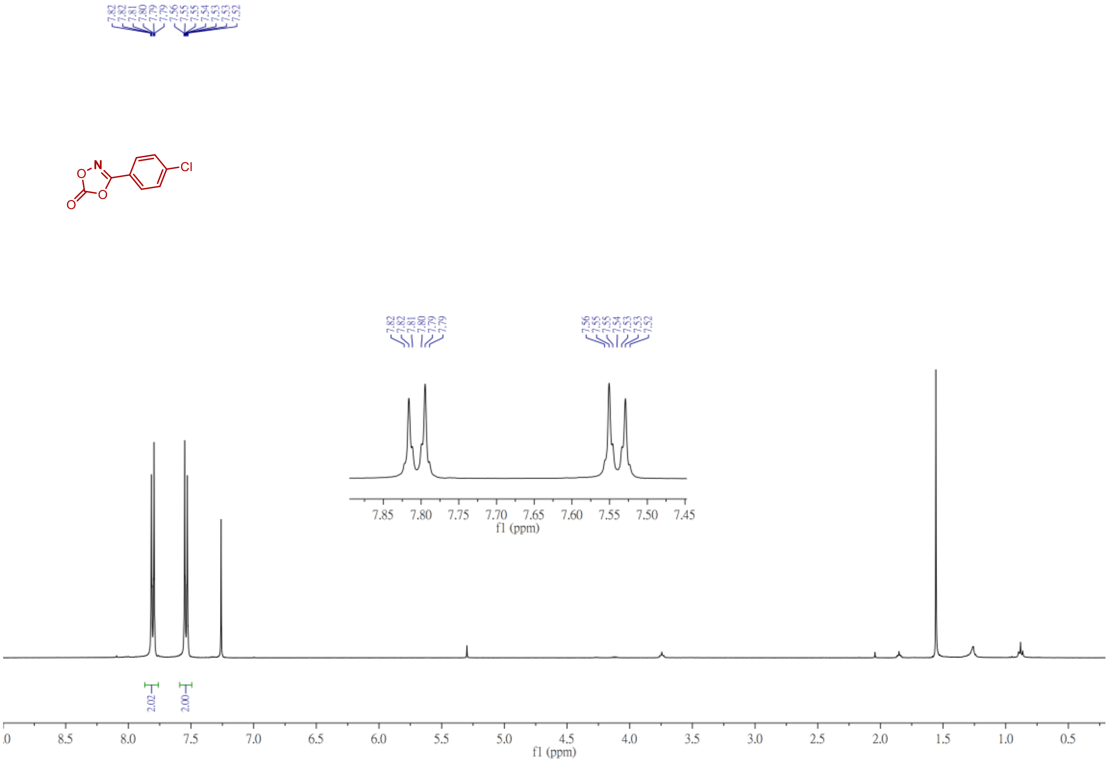

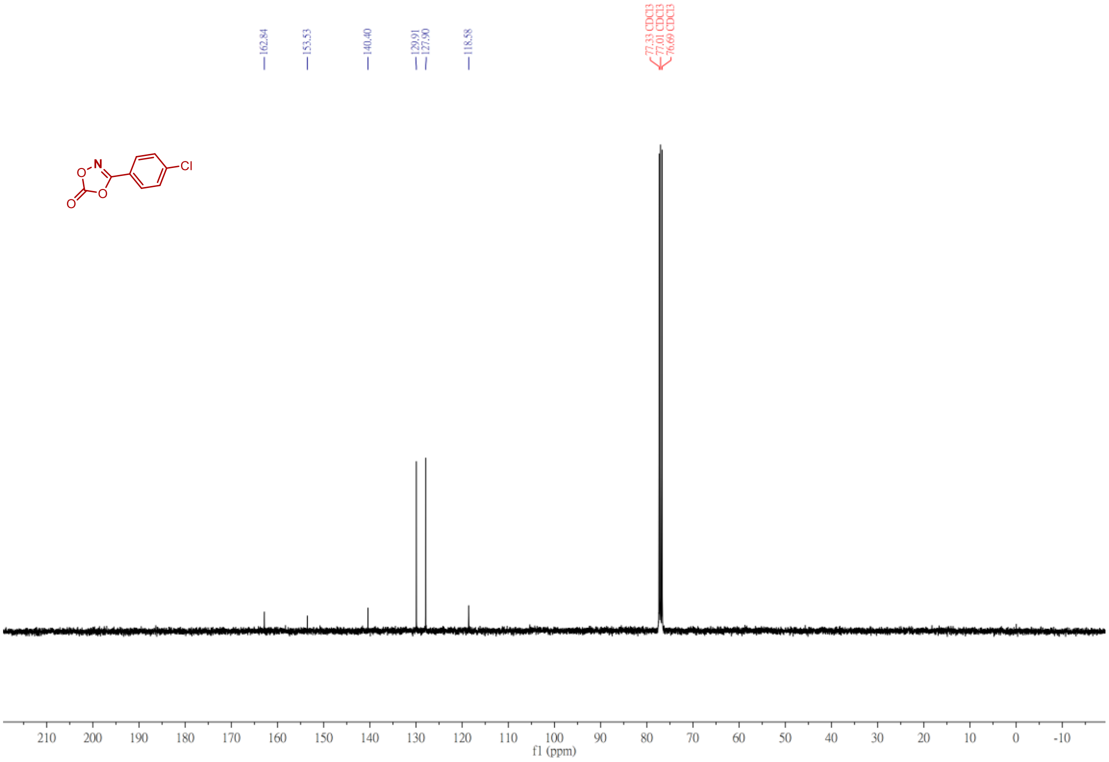

^{1}H and ^{13}C NMR spectrum of **S29**

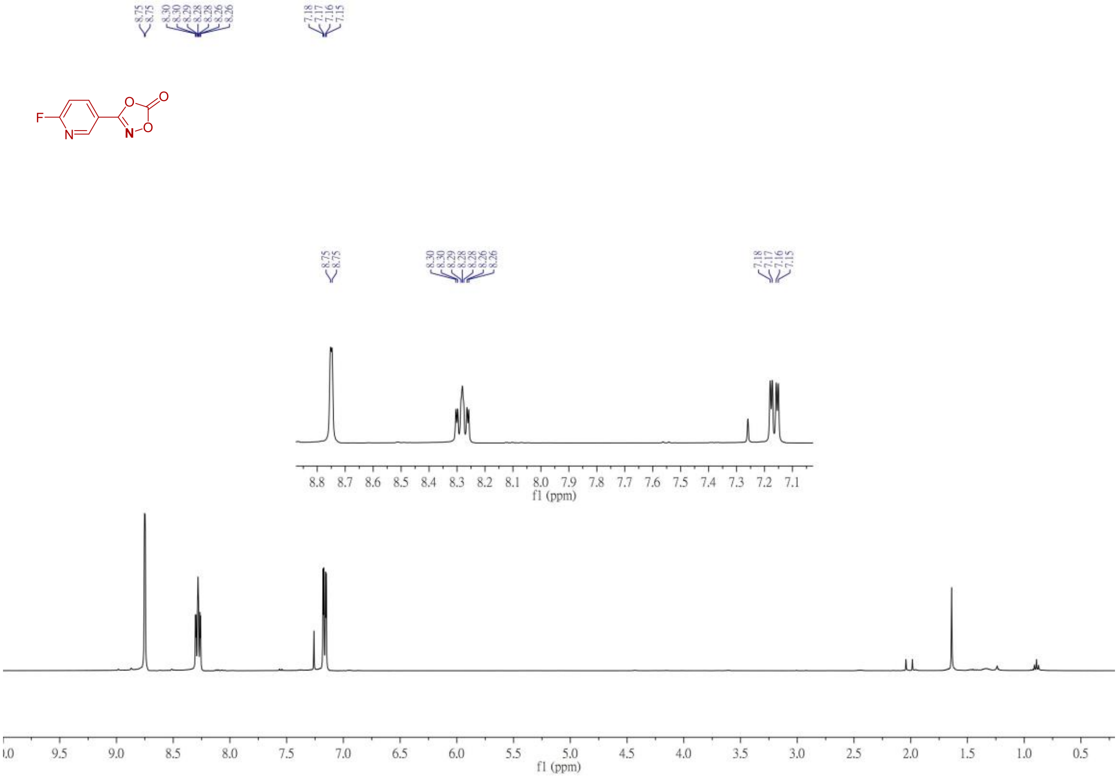

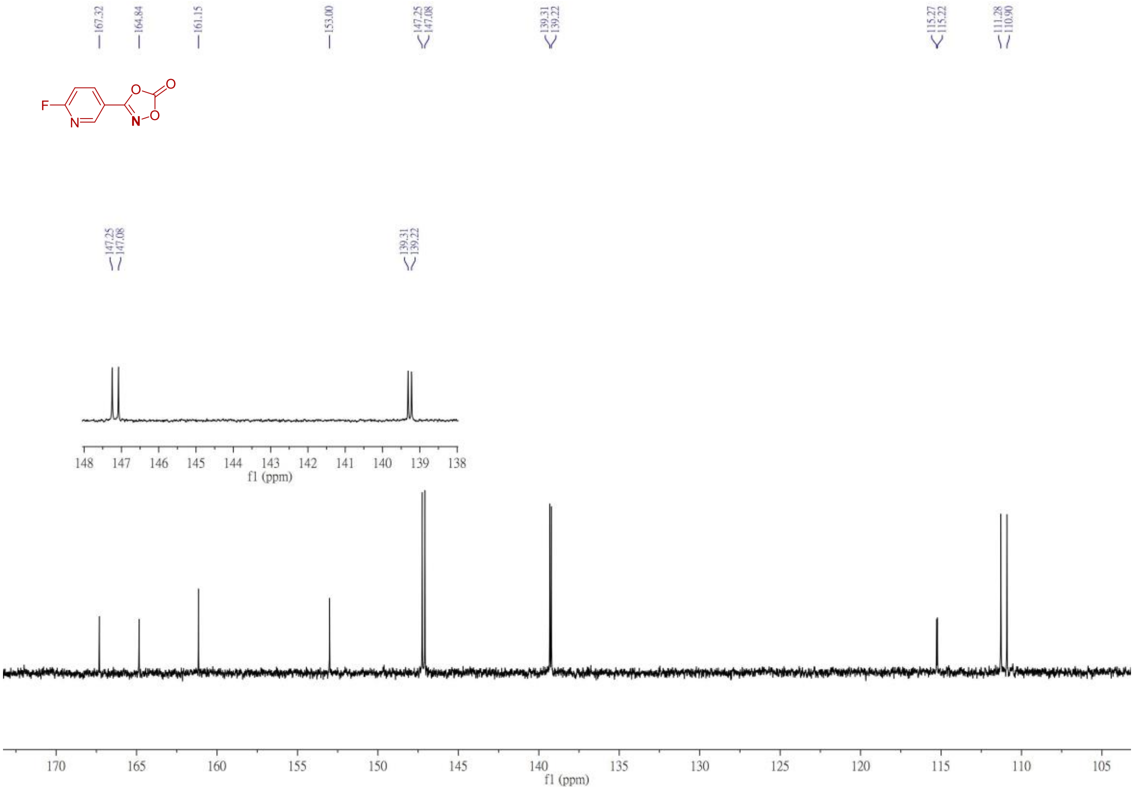

^{19}F NMR spectrum of **S29**

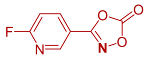

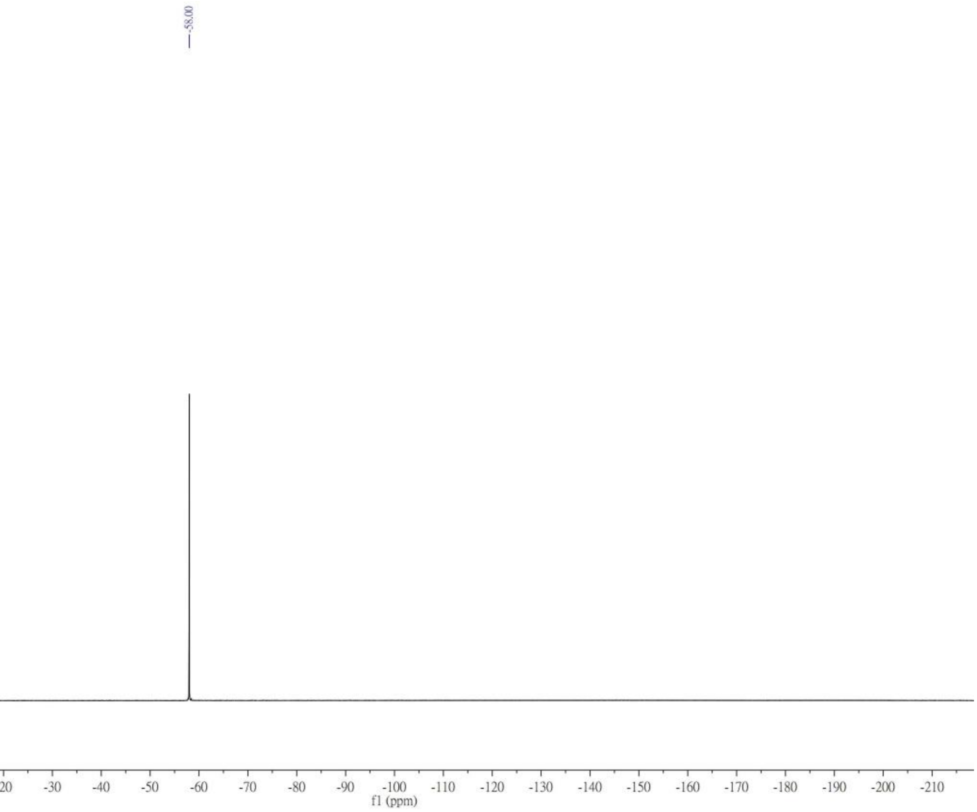

^{1}H and ^{13}C NMR spectrum of **S30**

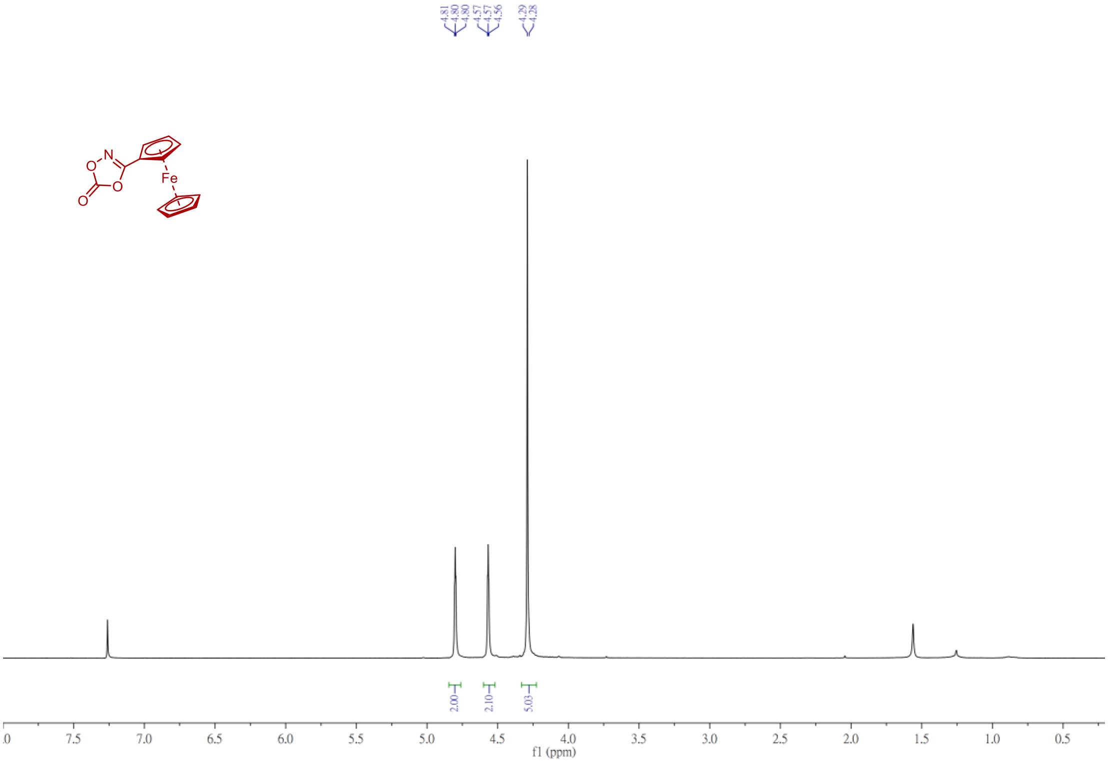

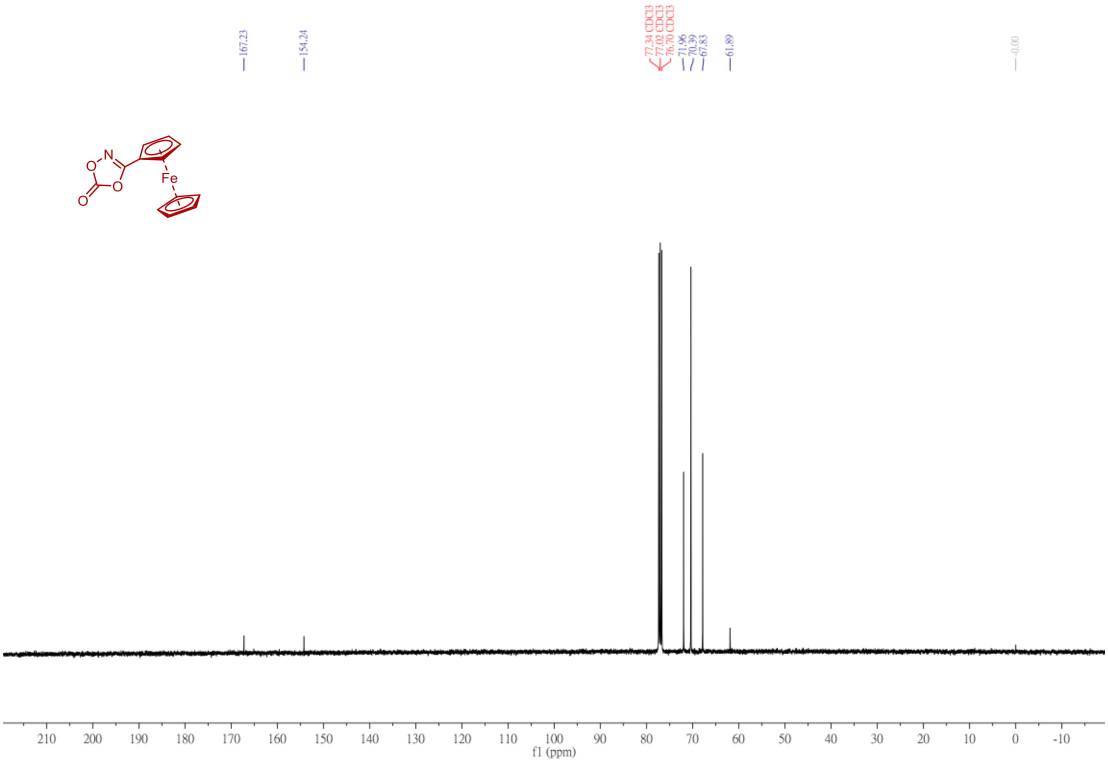

^{1}H and ^{13}C NMR spectrum of **S31**

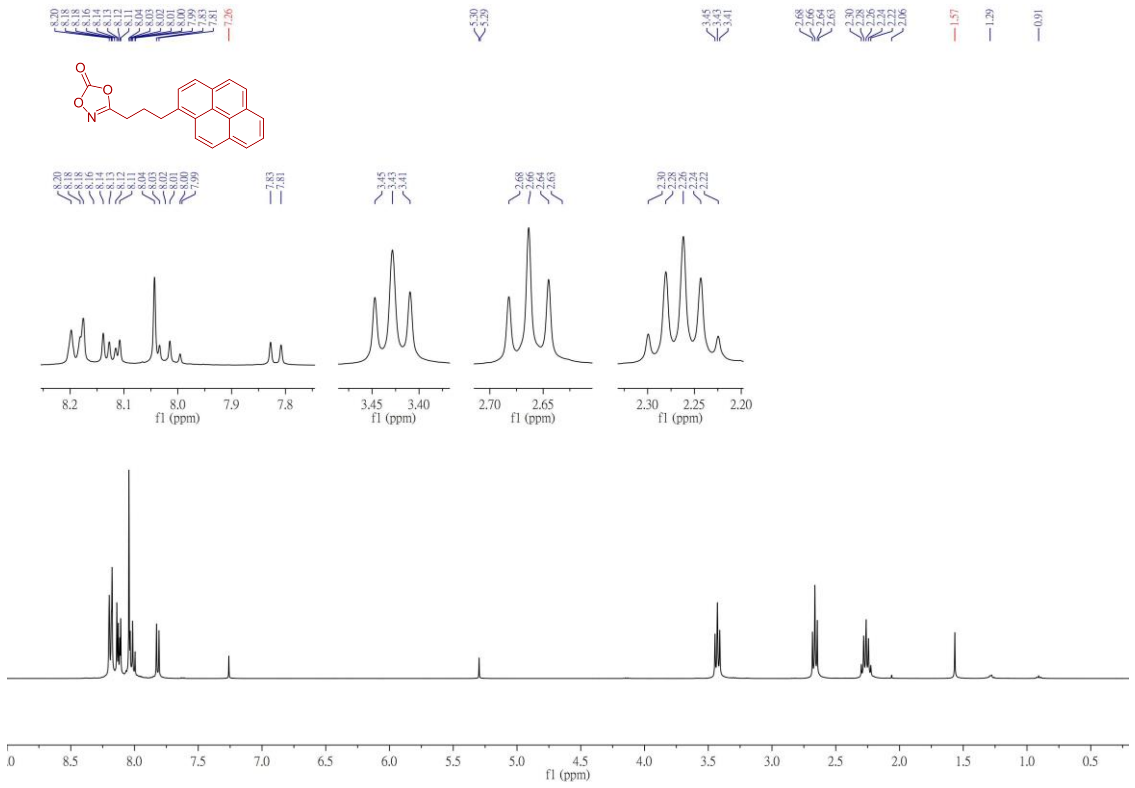

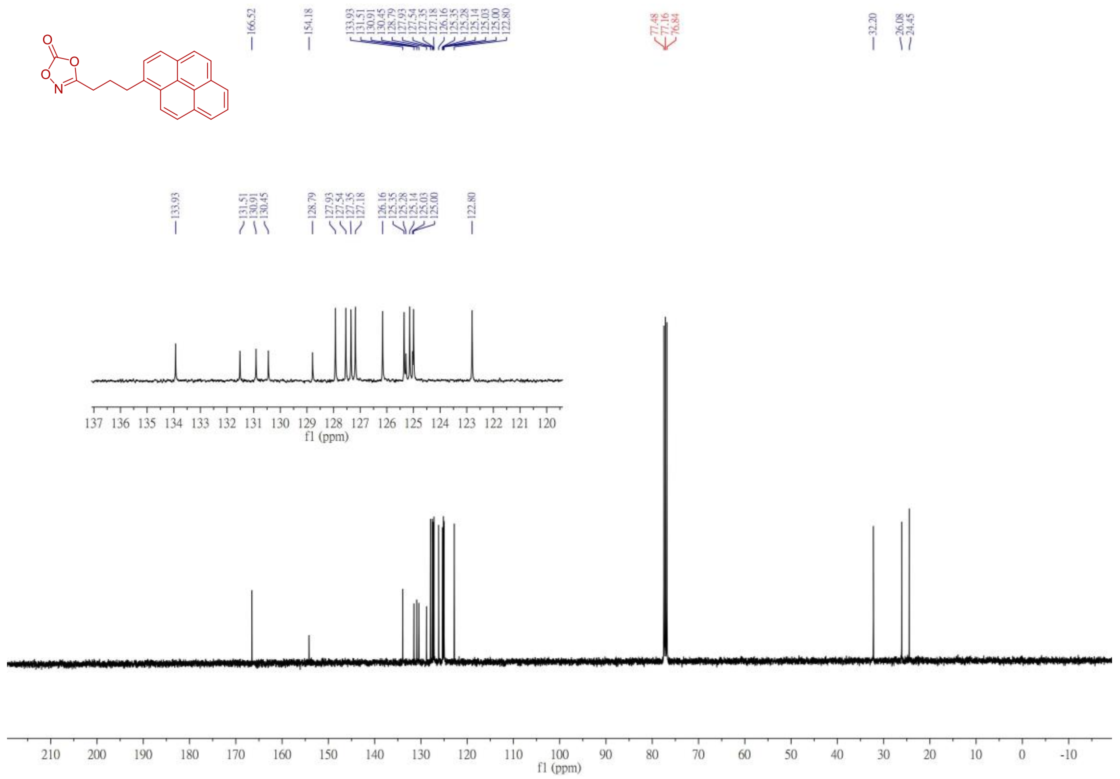

^{1}H and ^{13}C NMR spectrum of **S32**

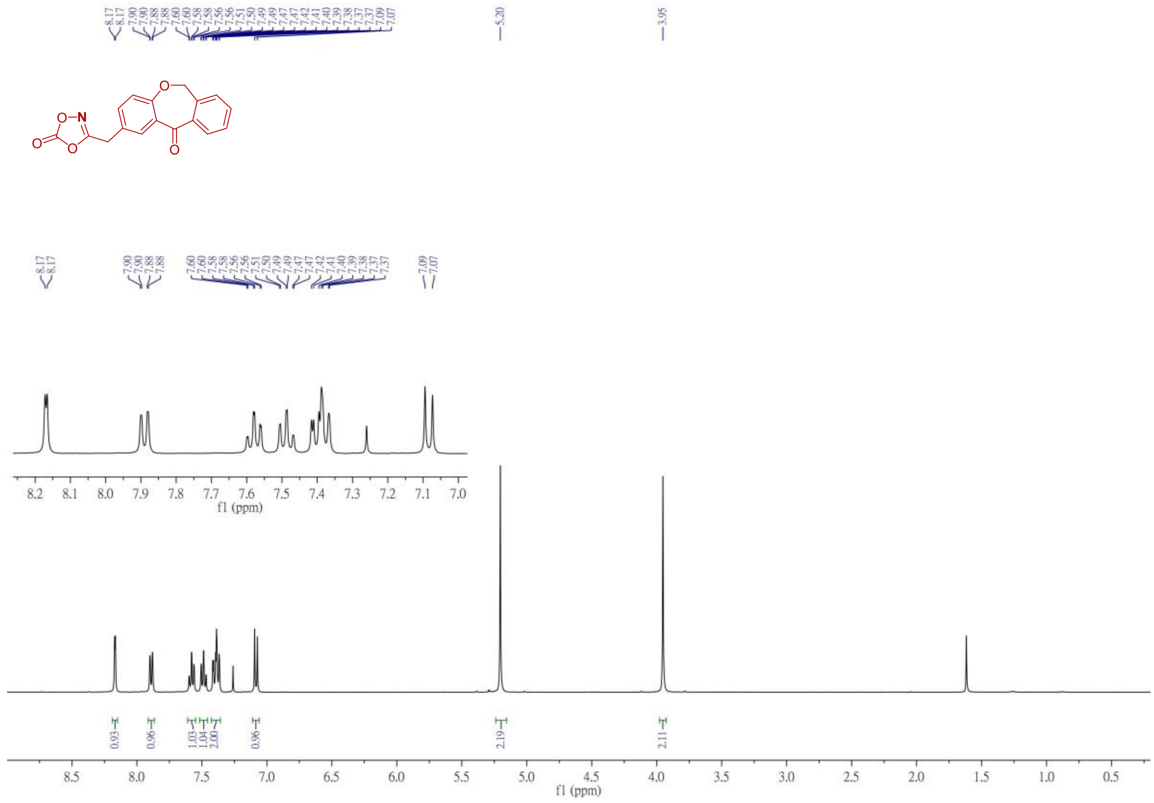

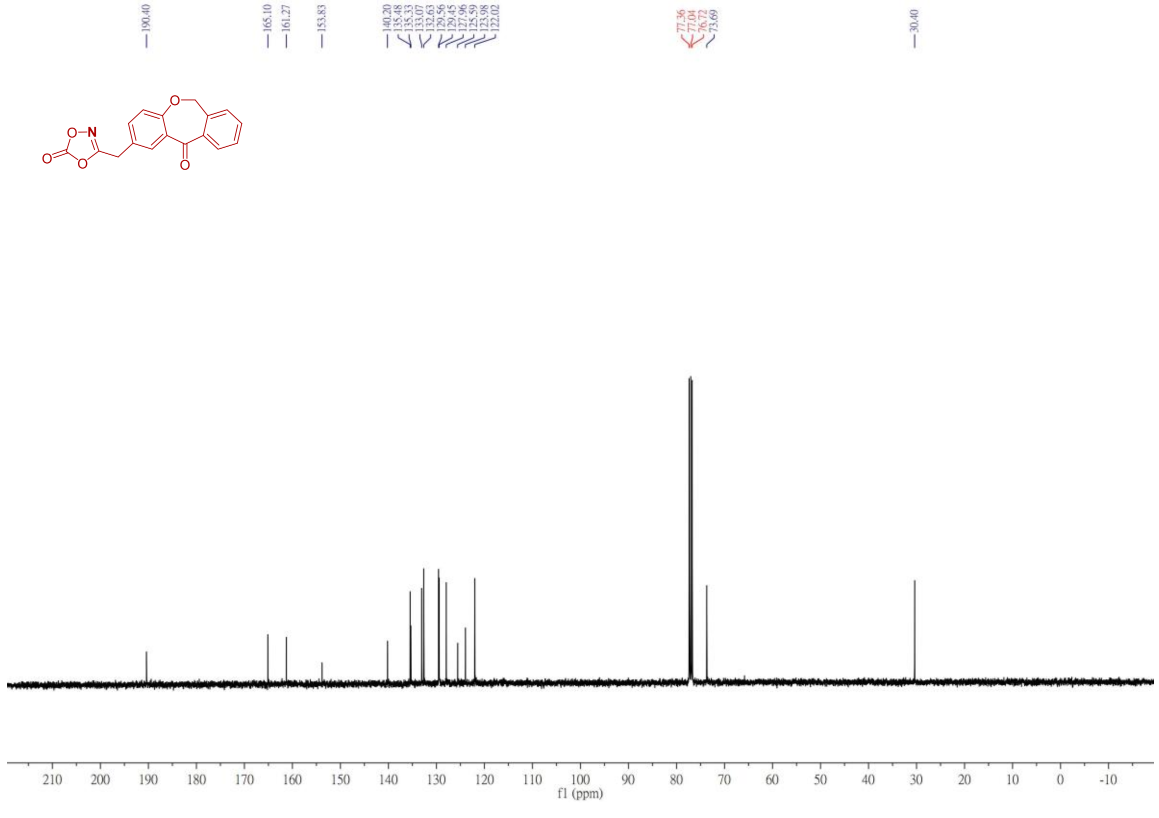

^{1}H and ^{13}C NMR spectrum of **S33**

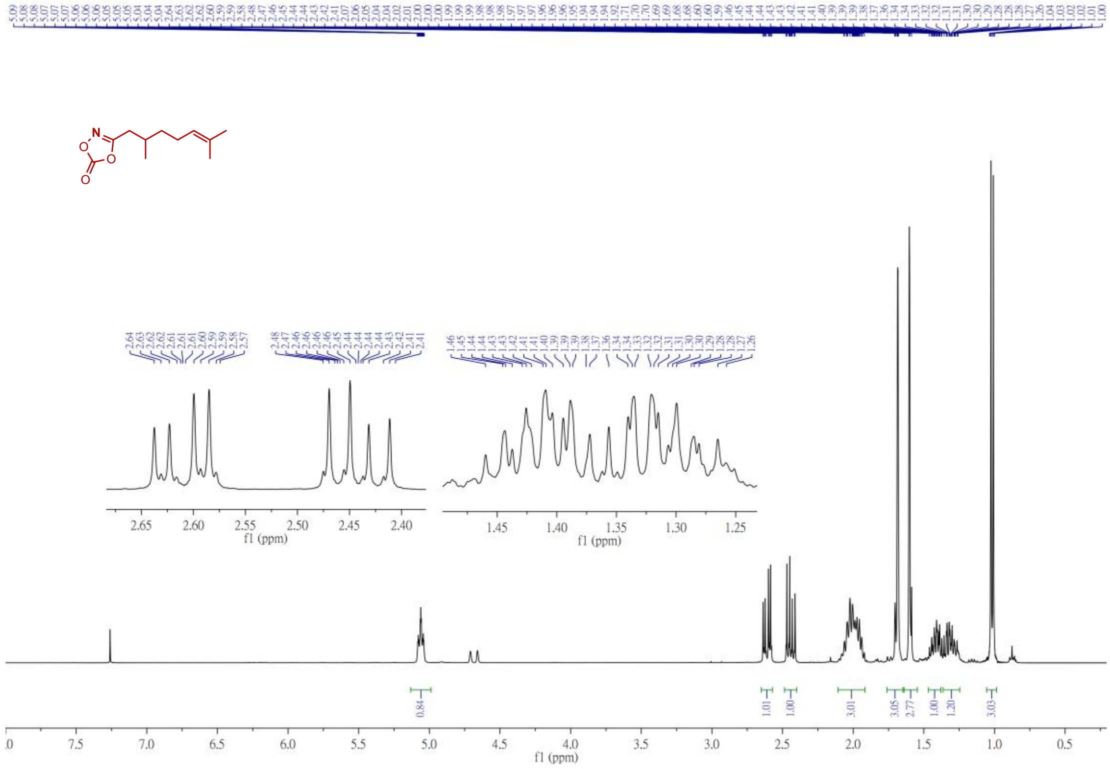

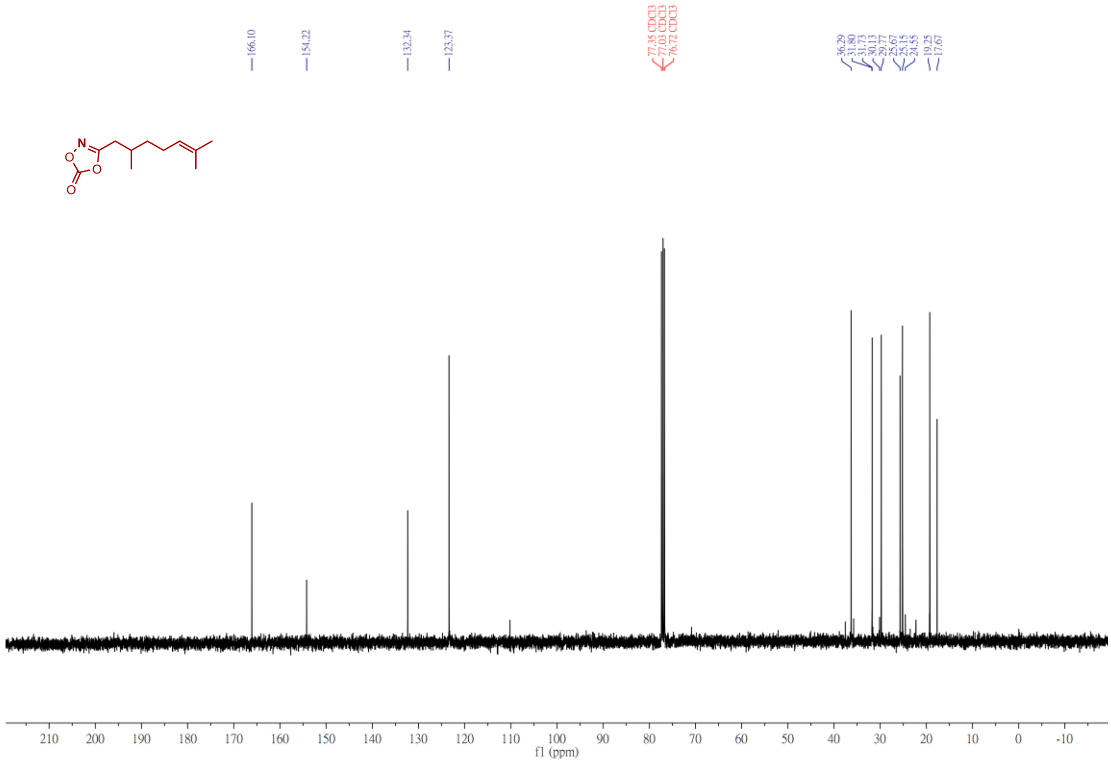

^{1}H and ^{13}C NMR spectrum of **S34**

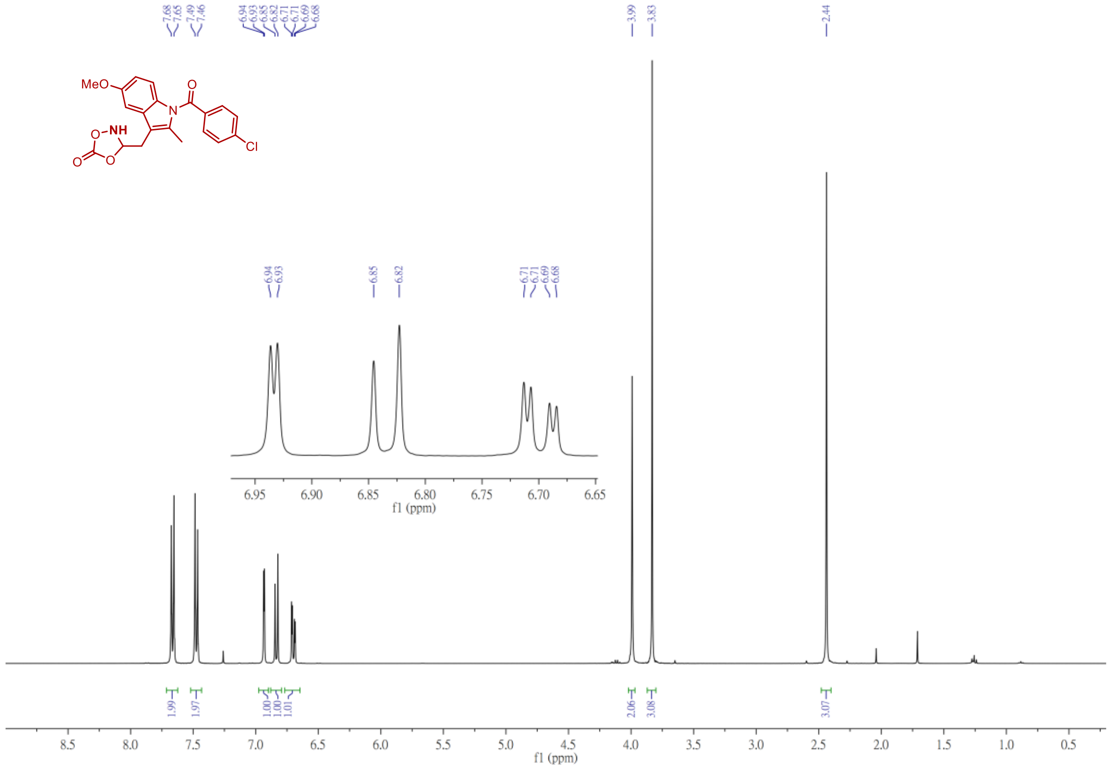

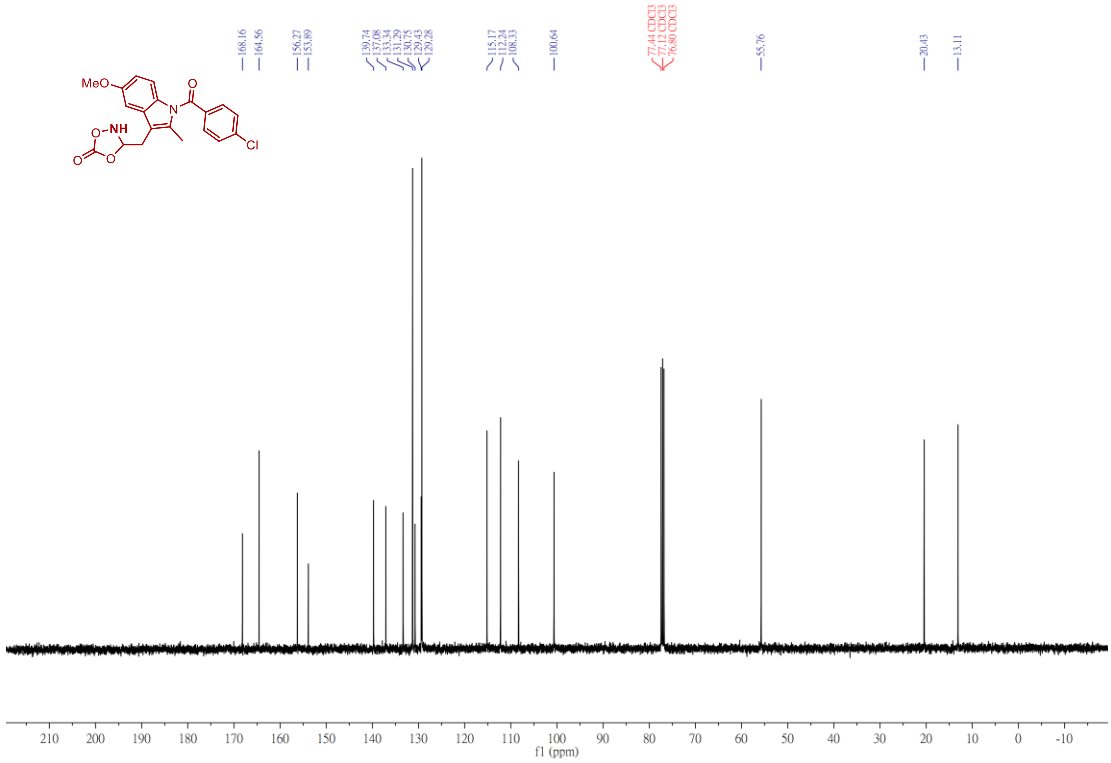

^{1}H and ^{13}C NMR spectrum of **S35**

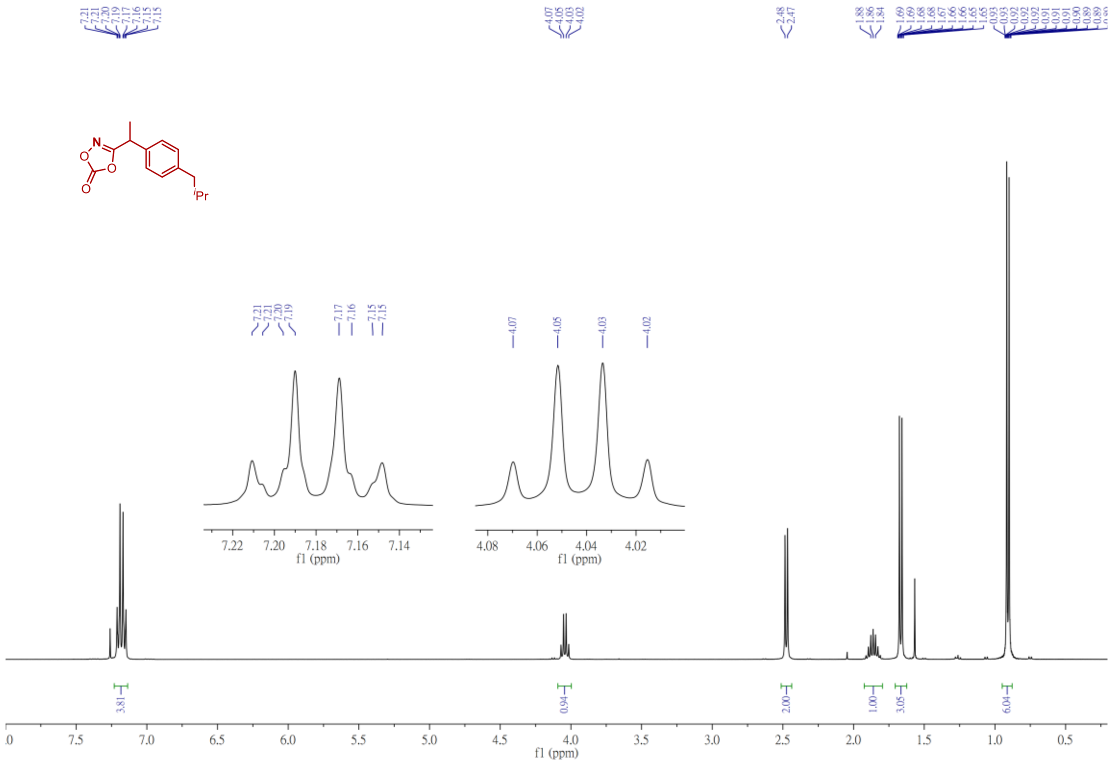

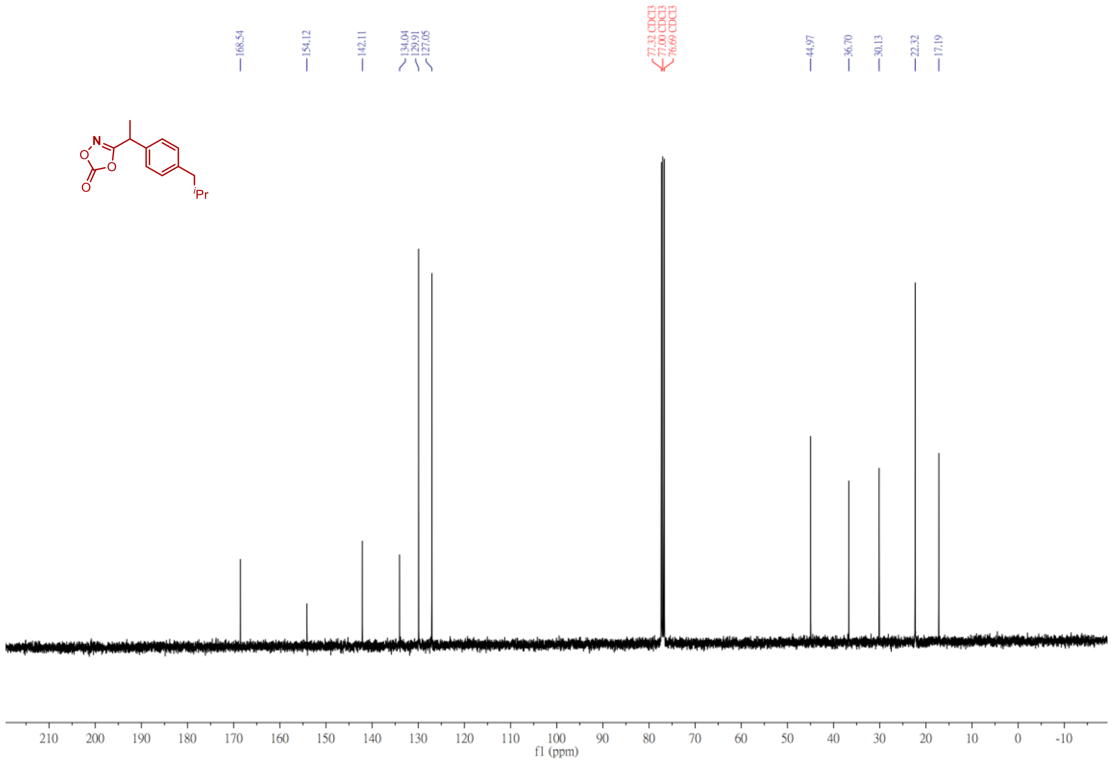

^{1}H and ^{13}C NMR spectrum of **S36**

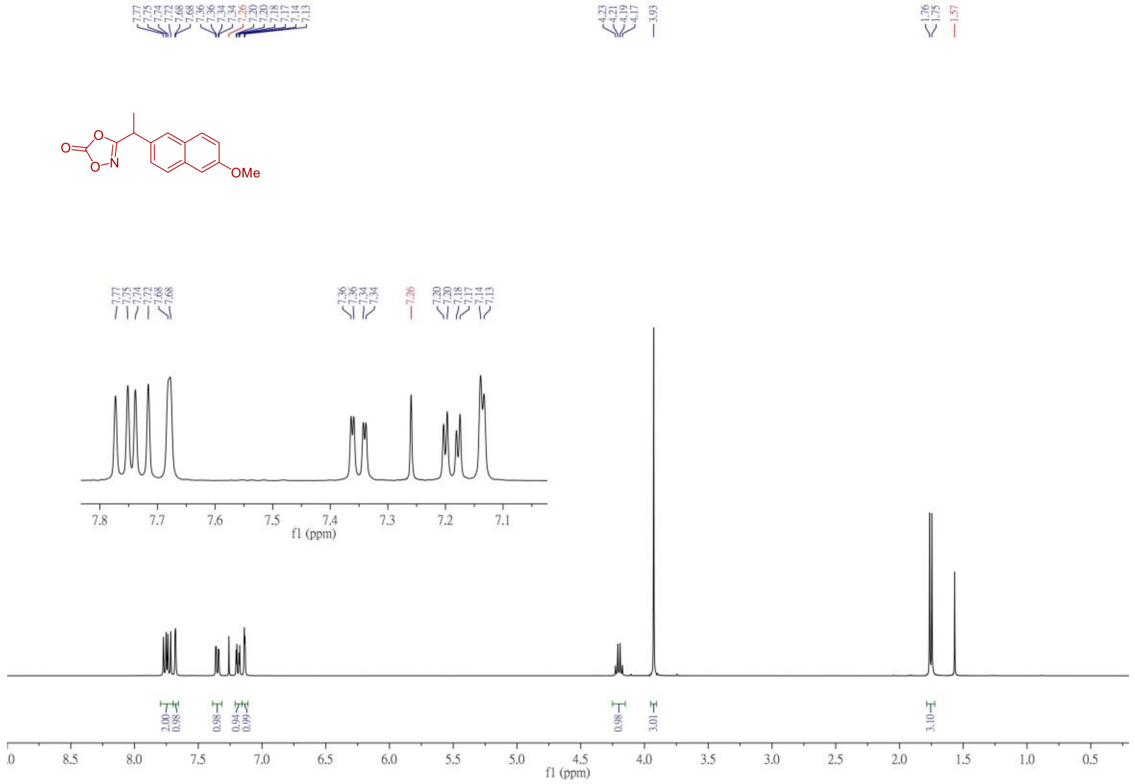

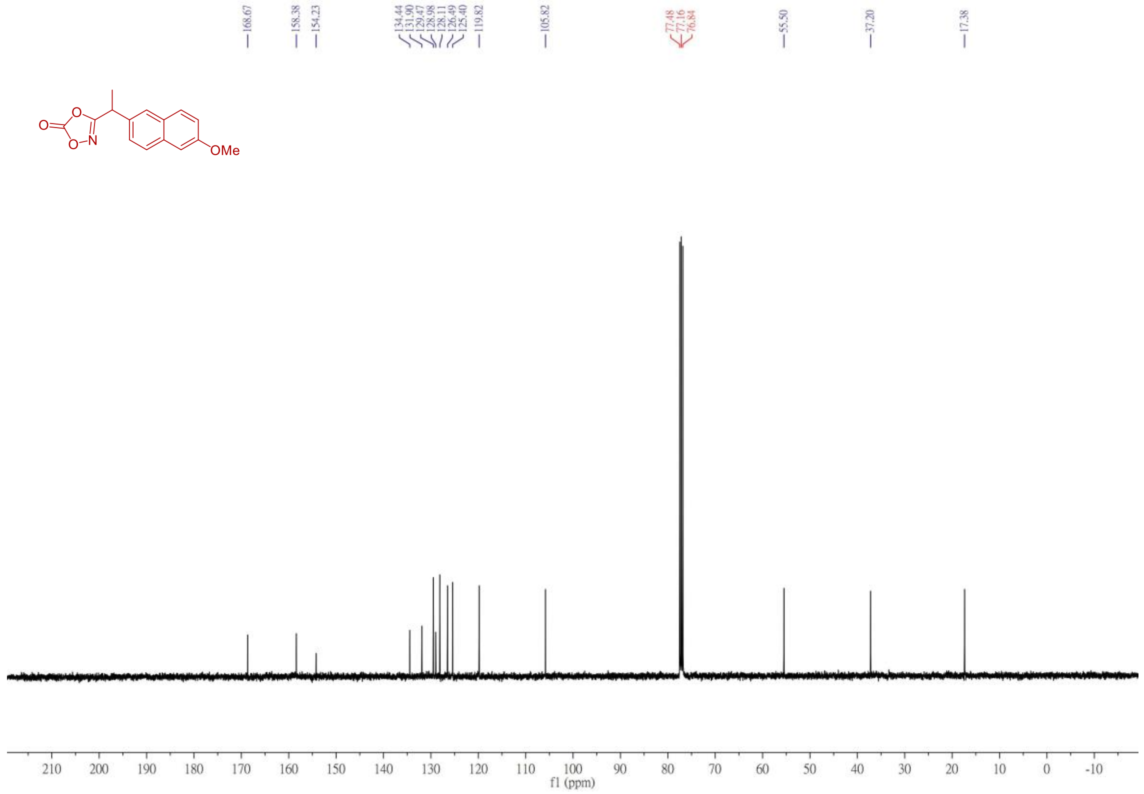

^{1}H and ^{13}C NMR spectrum of **S37**

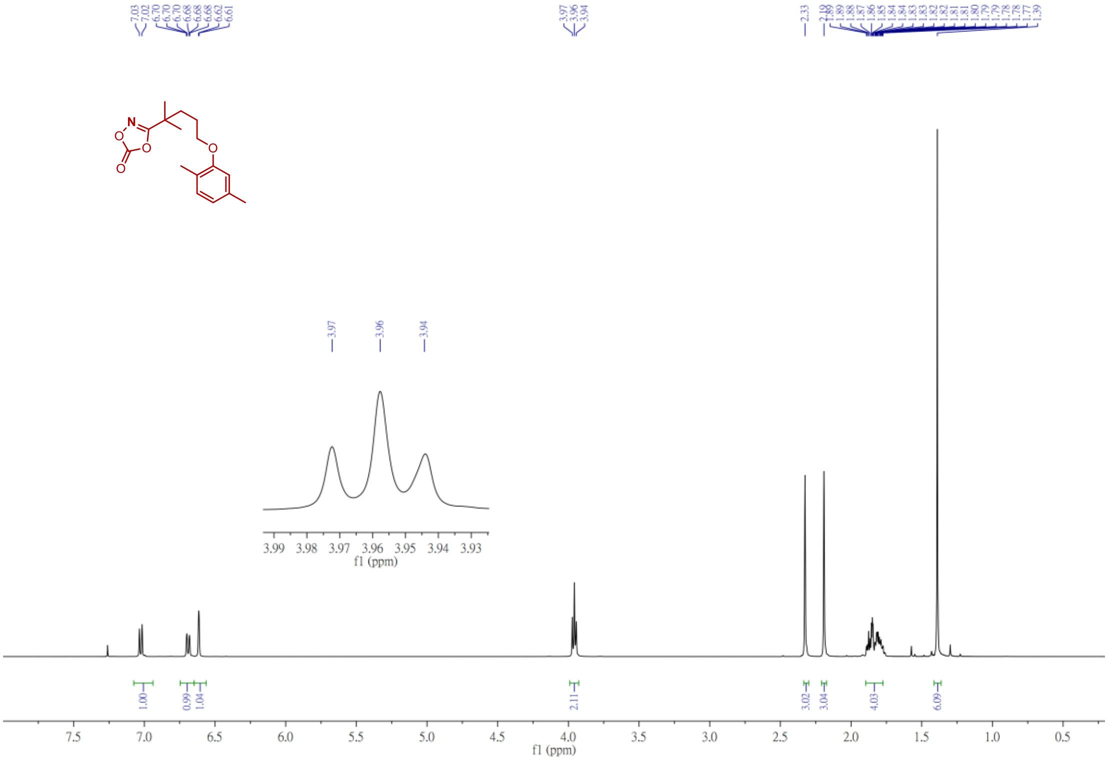

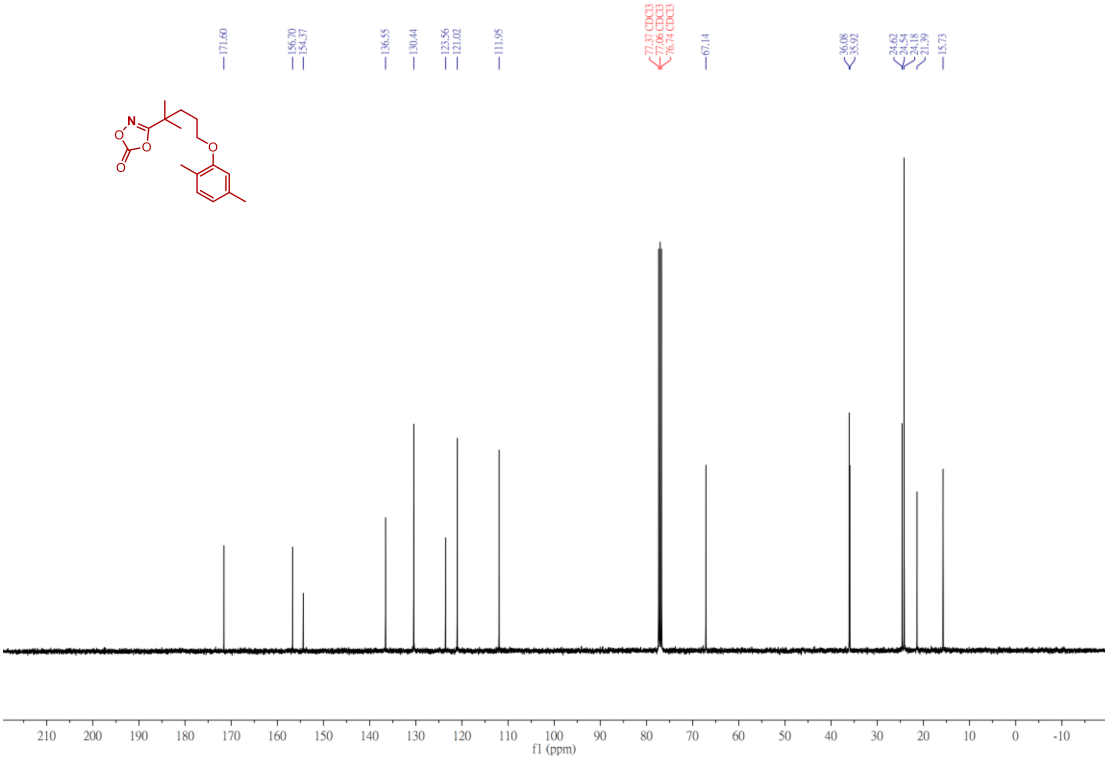

^{1}H and ^{13}C NMR spectrum of **S57**

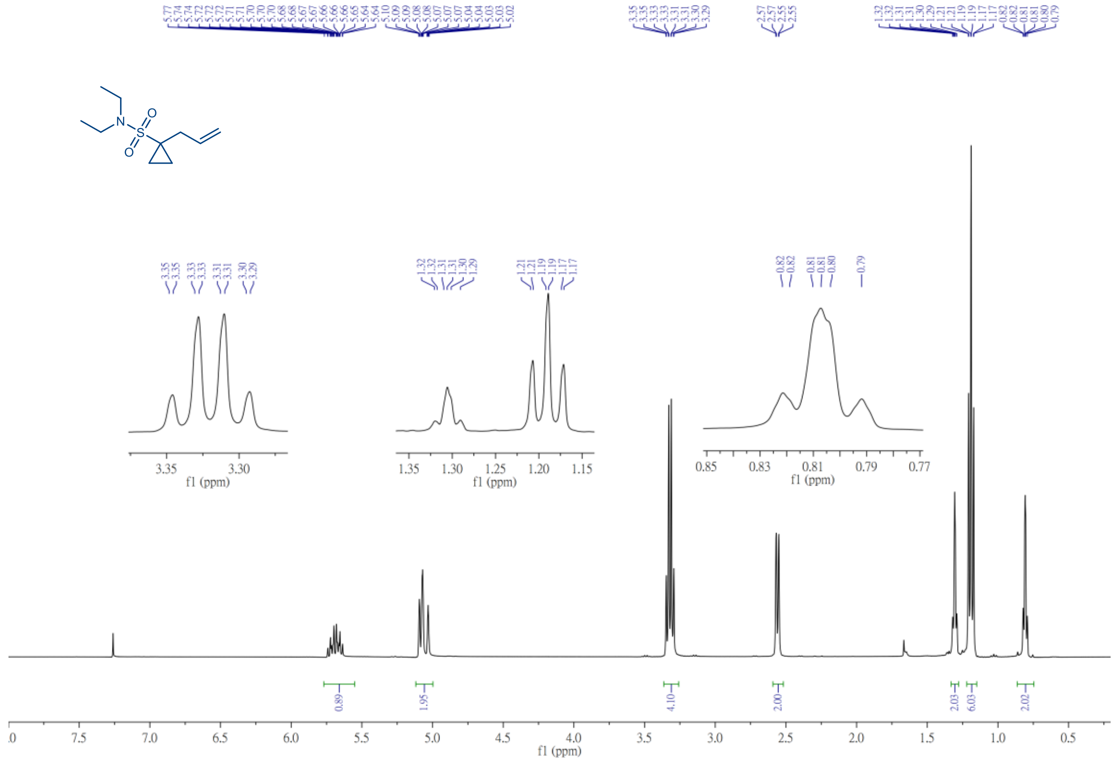

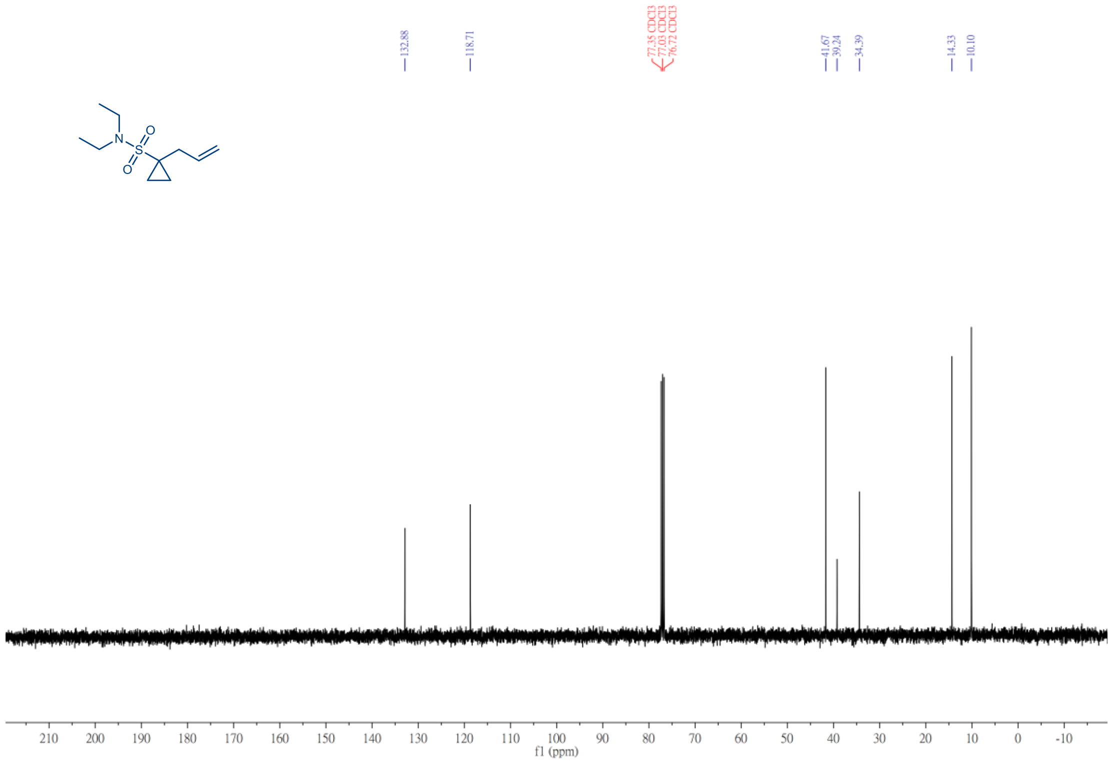

^{1}H and ^{13}C NMR spectrum of **S59**

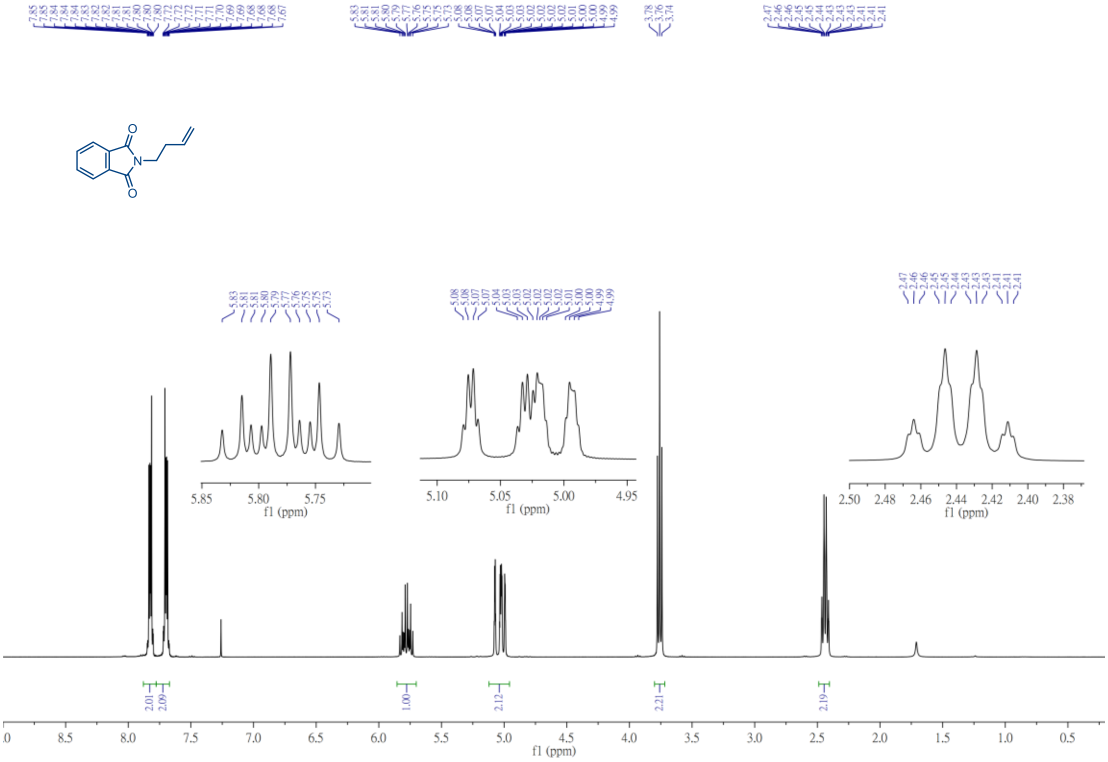

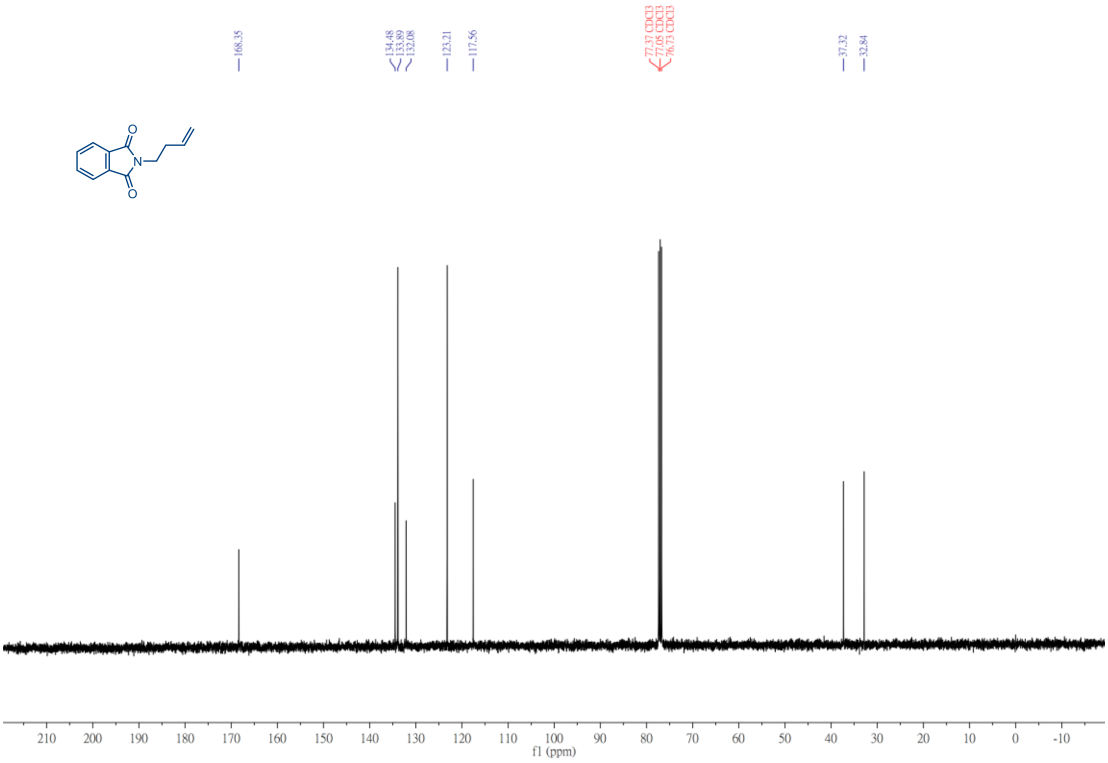

^{1}H and ^{13}C NMR spectrum of **S62**

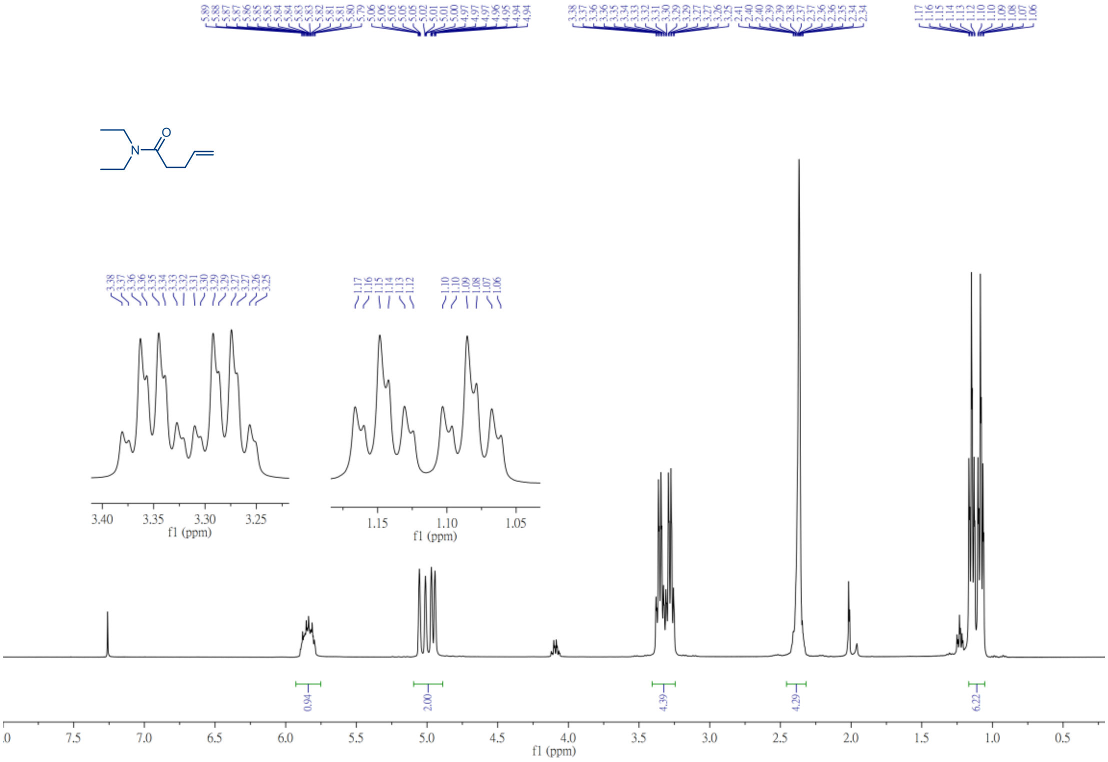

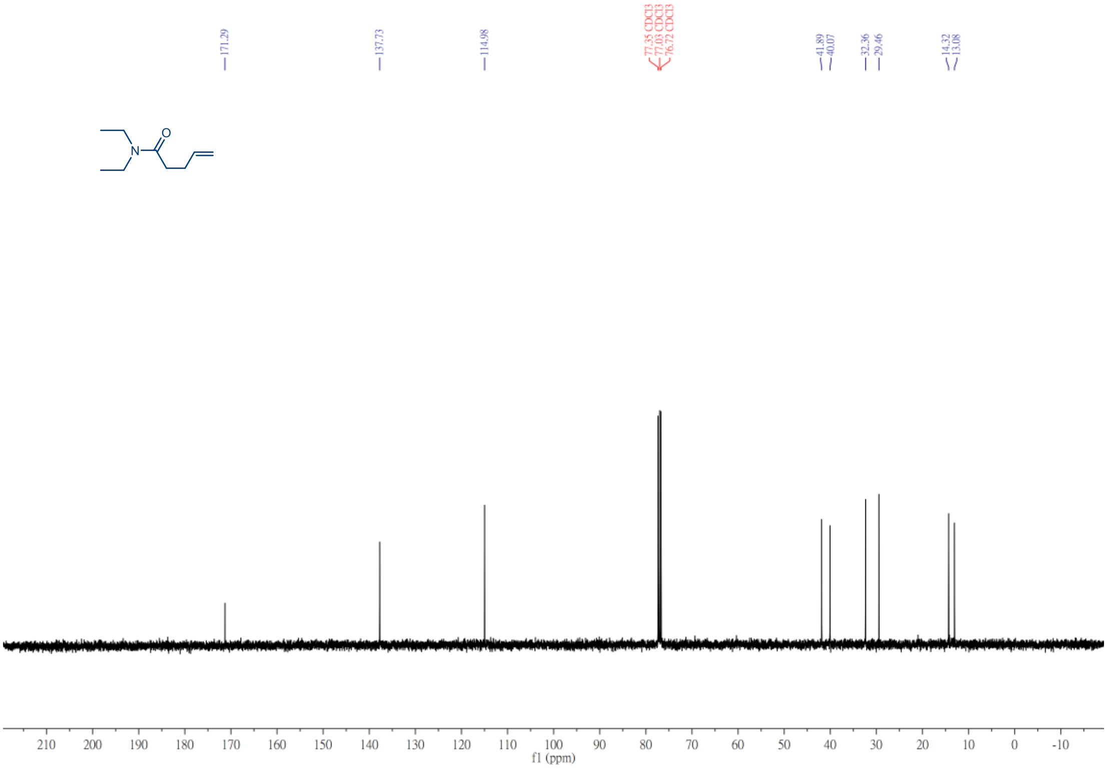

^{1}H and ^{13}C NMR spectrum of **S67**

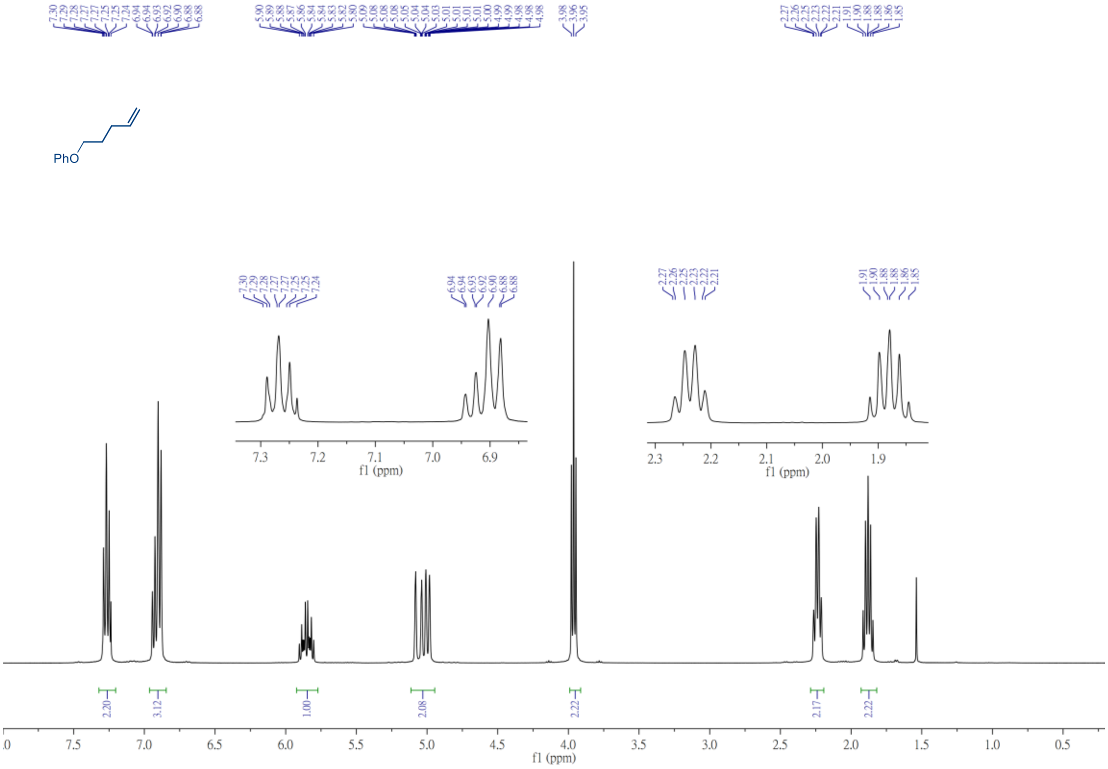

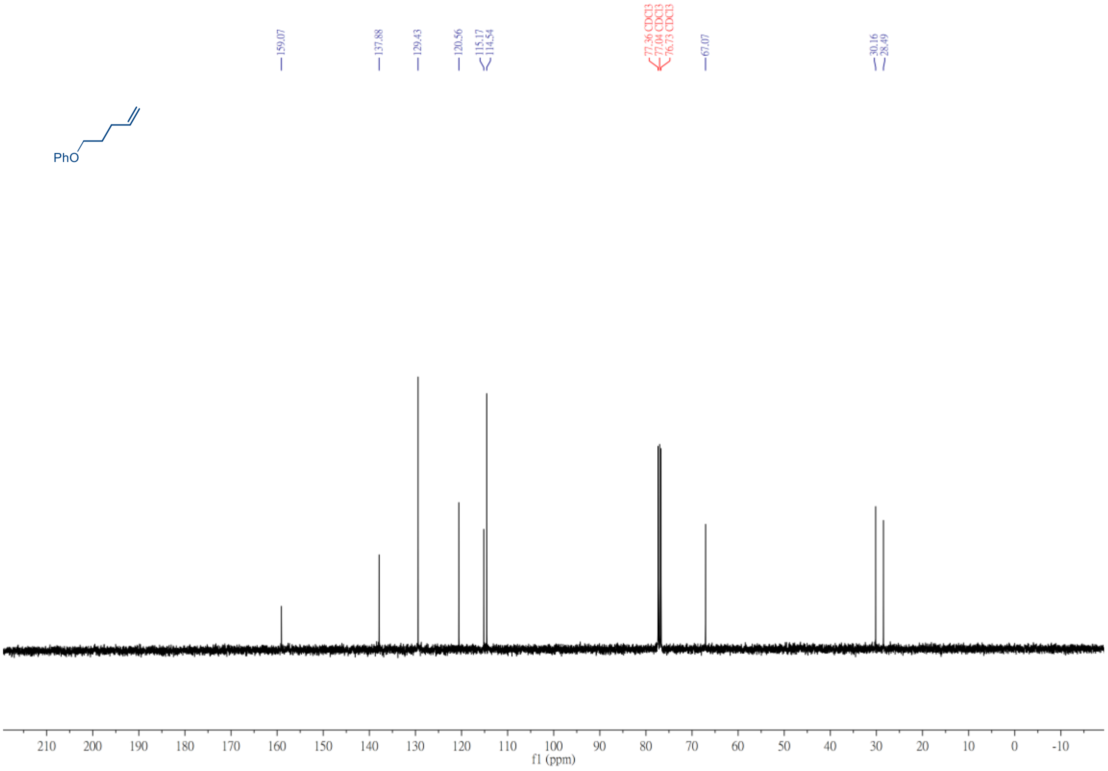

^{1}H and ^{13}C NMR spectrum of **S68**

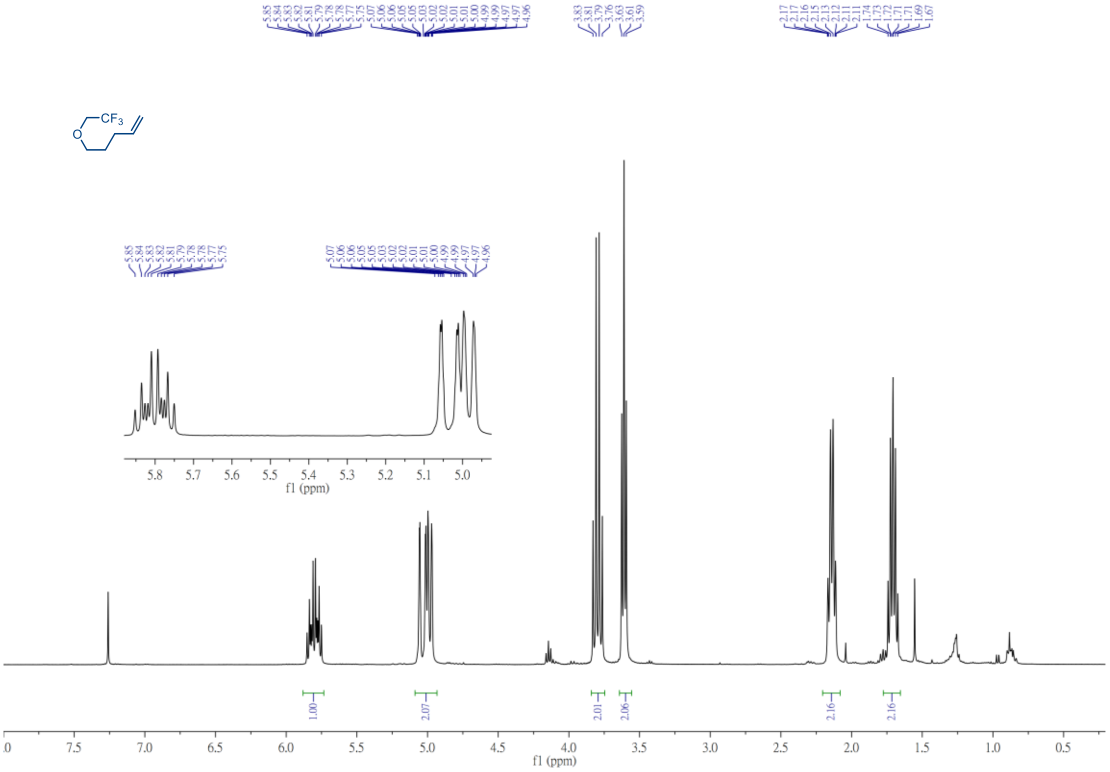

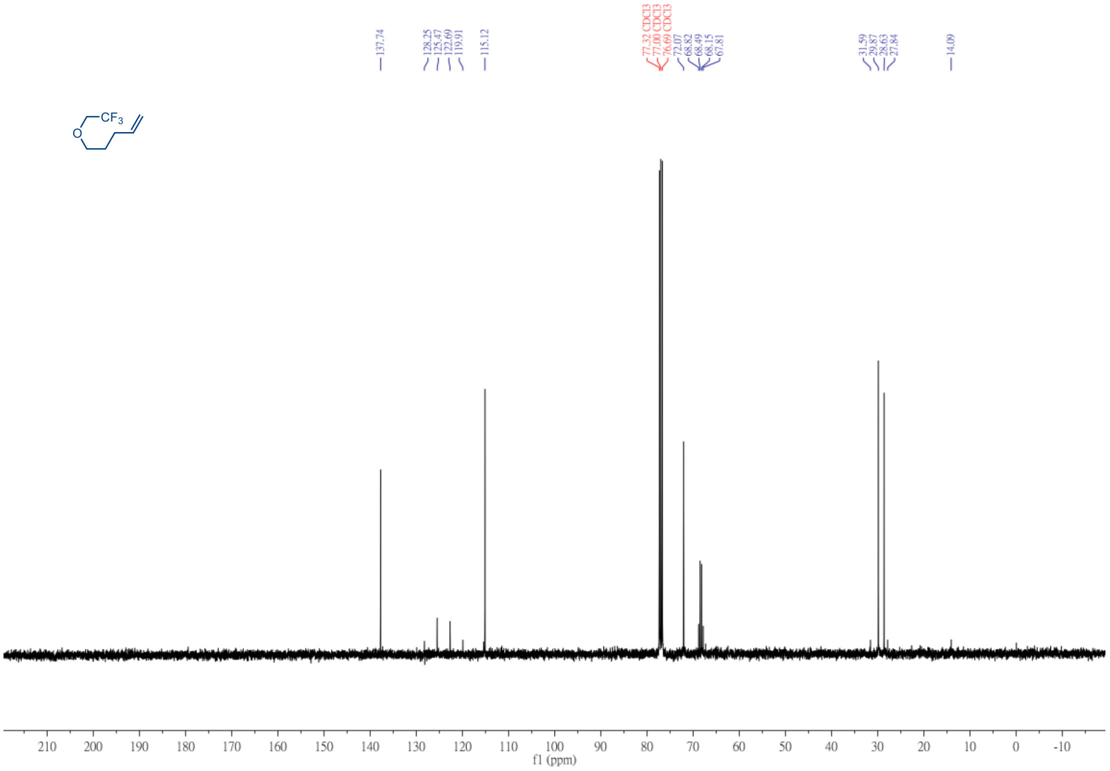

^{19}F NMR spectrum of **S68**

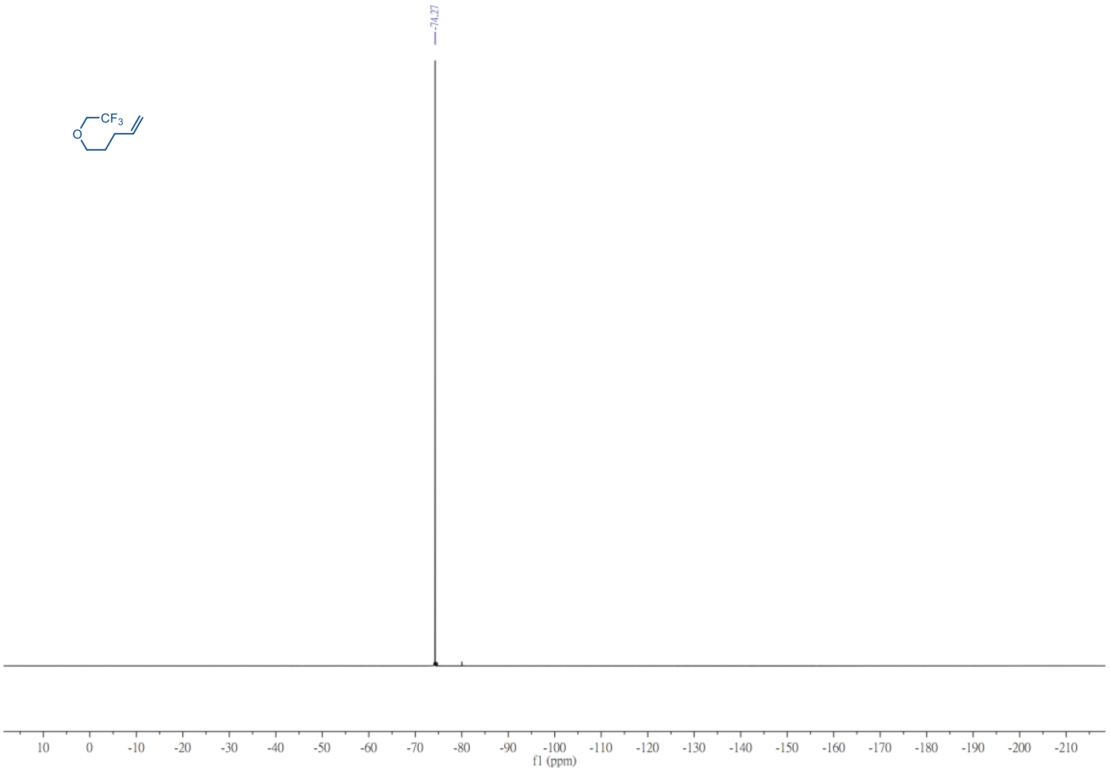

^{1}H and ^{13}C NMR spectrum of **S69**

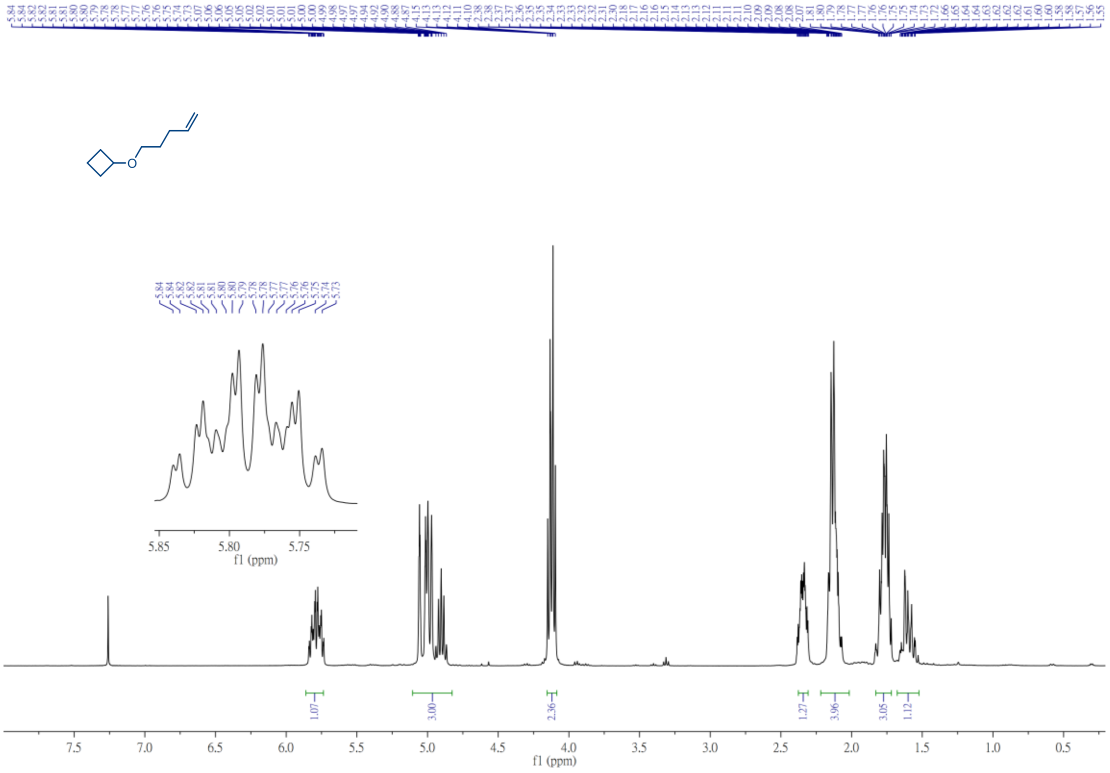

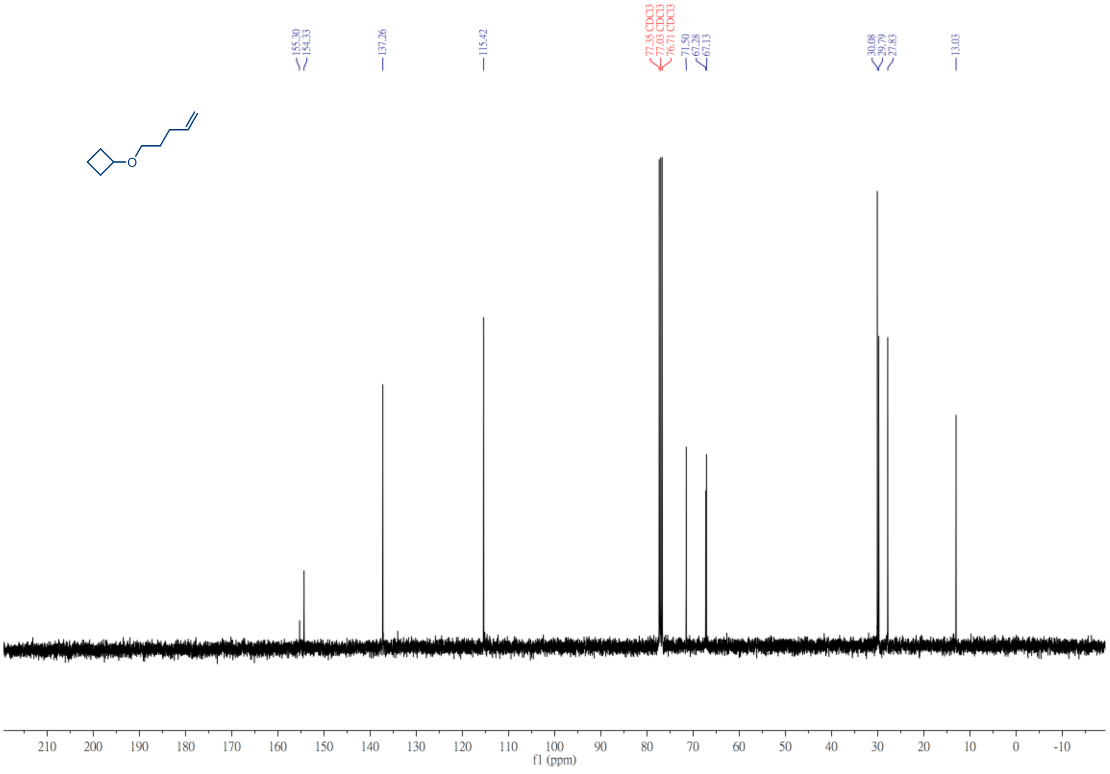

^{1}H and ^{13}C NMR spectrum of **S70**

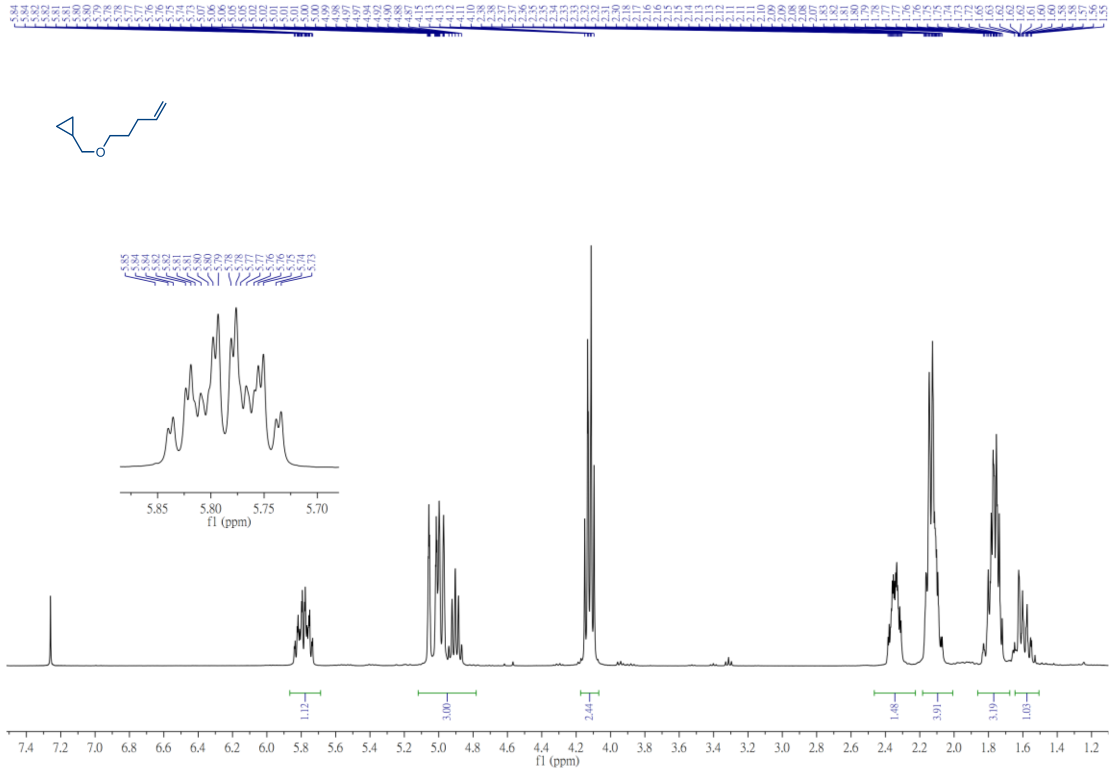

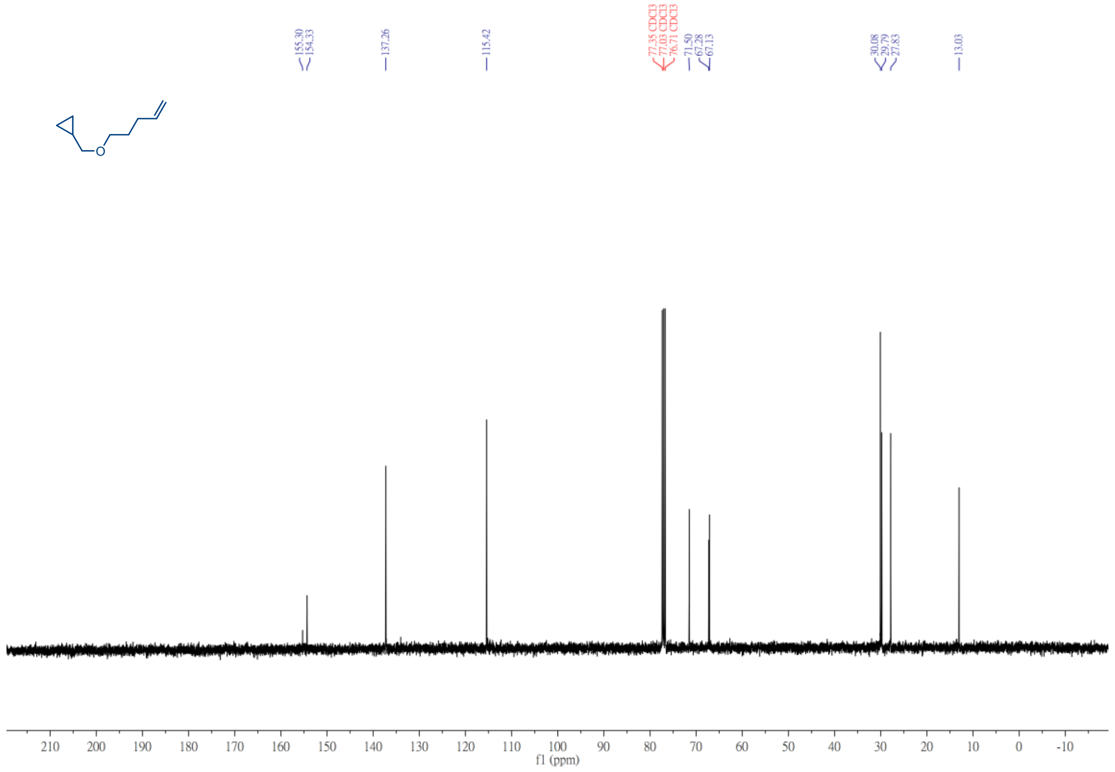

^{1}H and ^{13}C NMR spectrum of **S71**

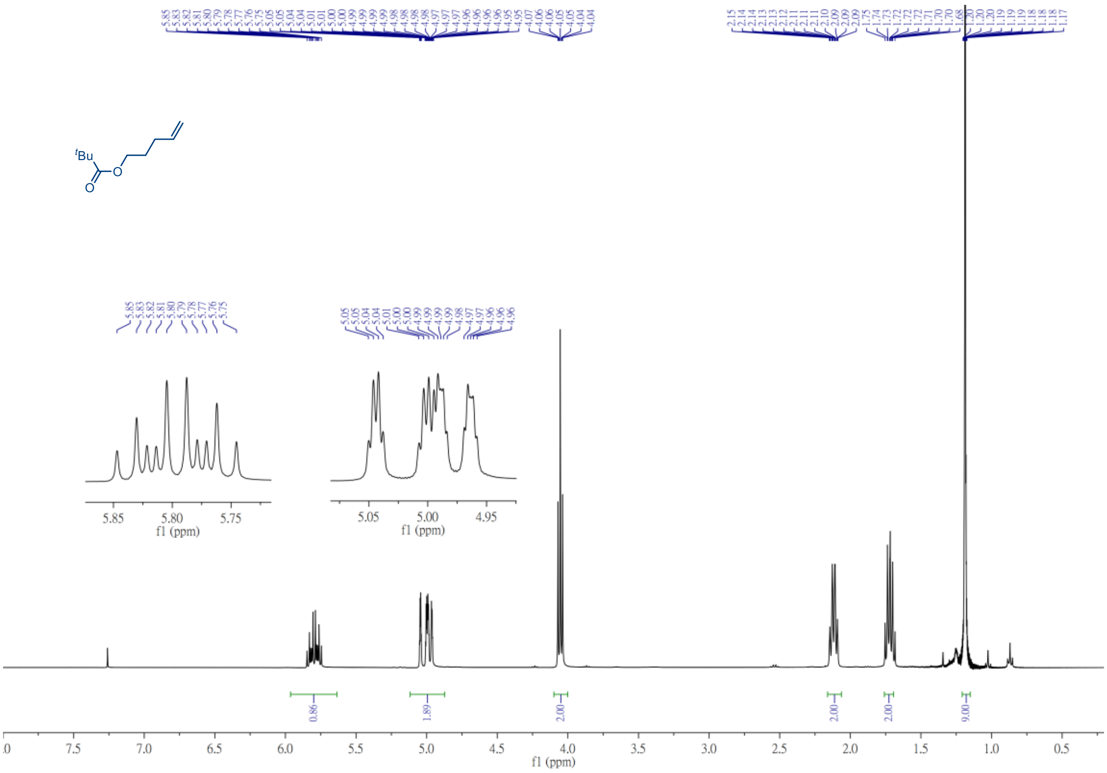

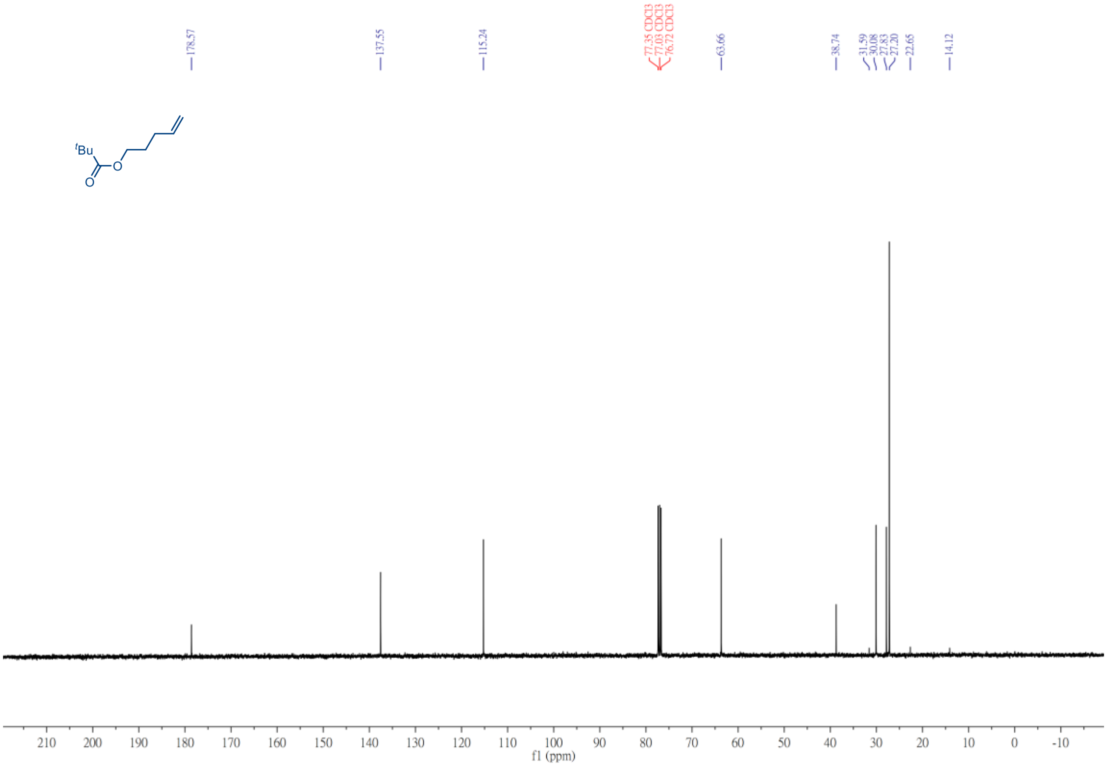

^{1}H and ^{13}C NMR spectrum of **S73**

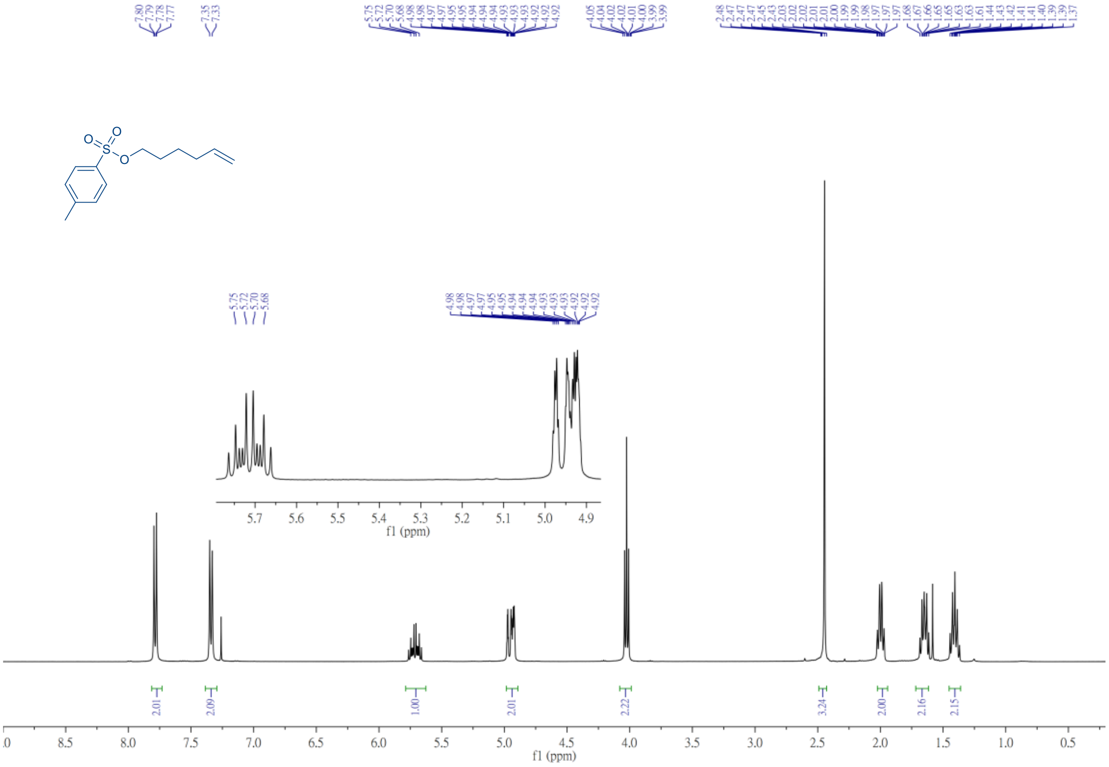

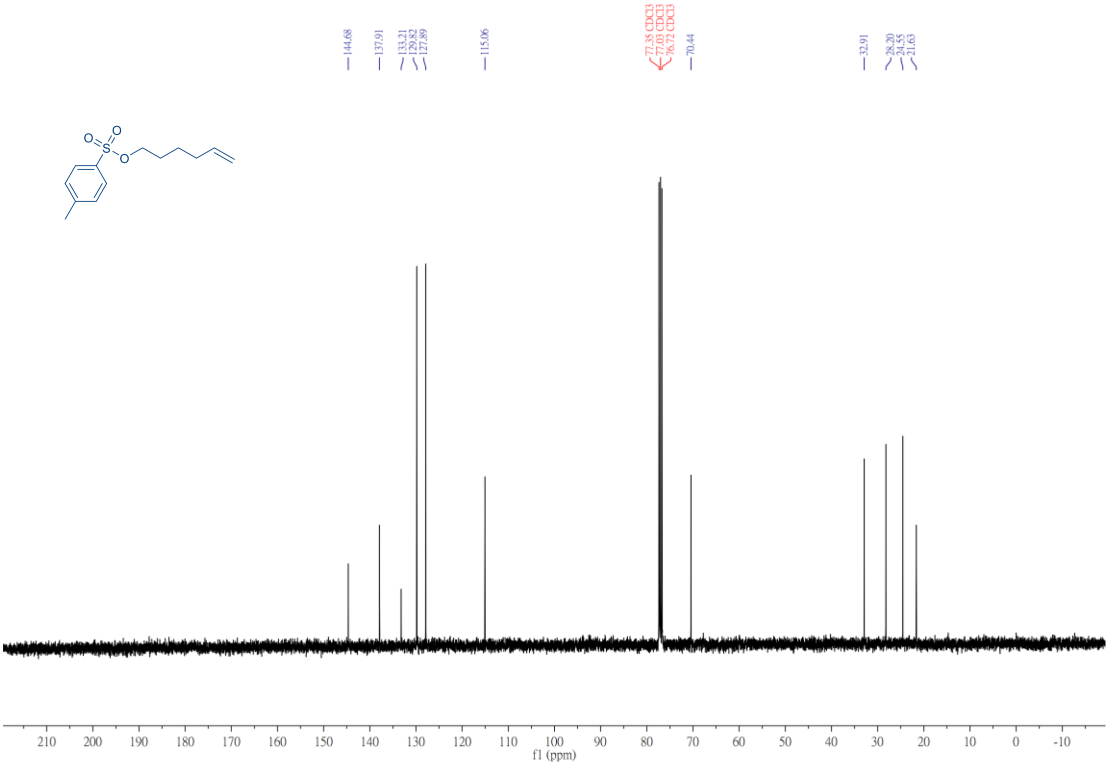

^{1}H and ^{13}C NMR spectrum of **S74**

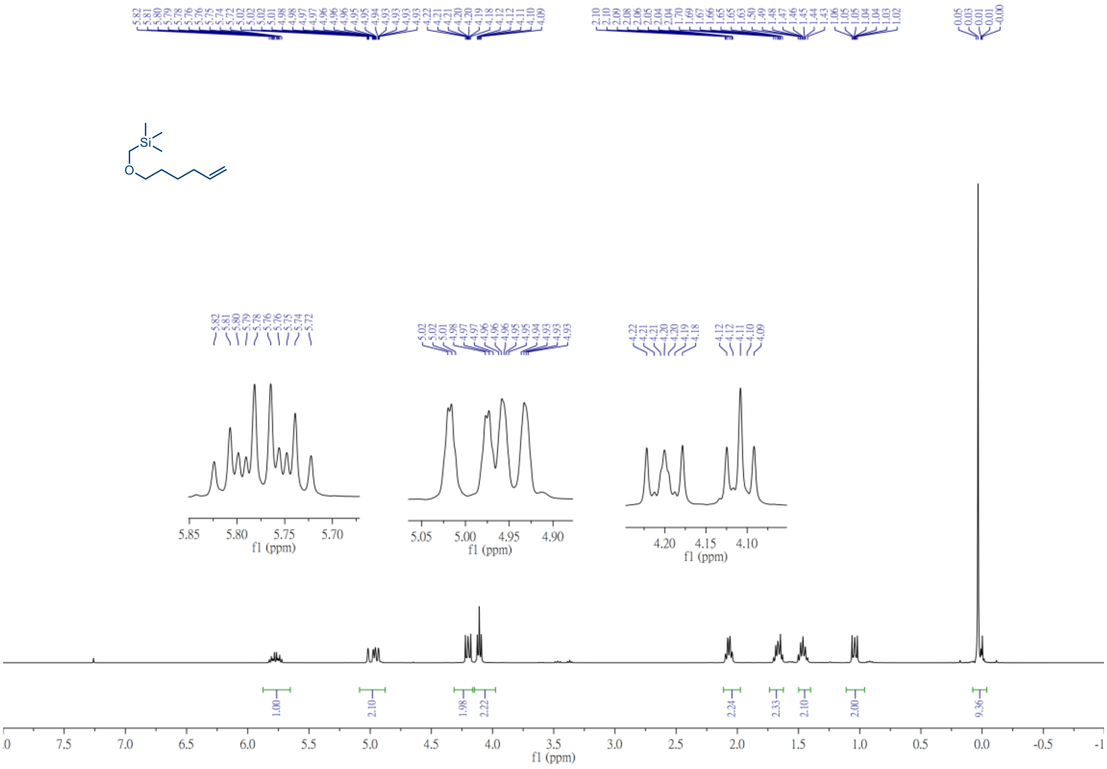

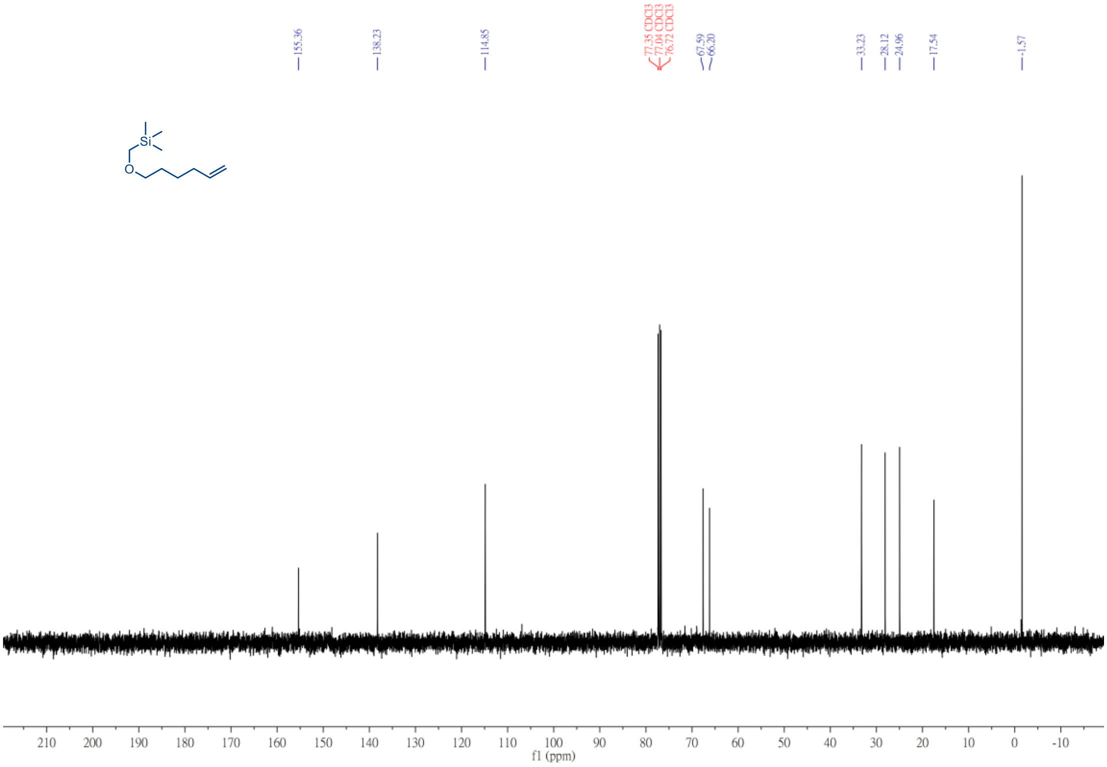

^{1}H and ^{13}C NMR spectrum of **S75**

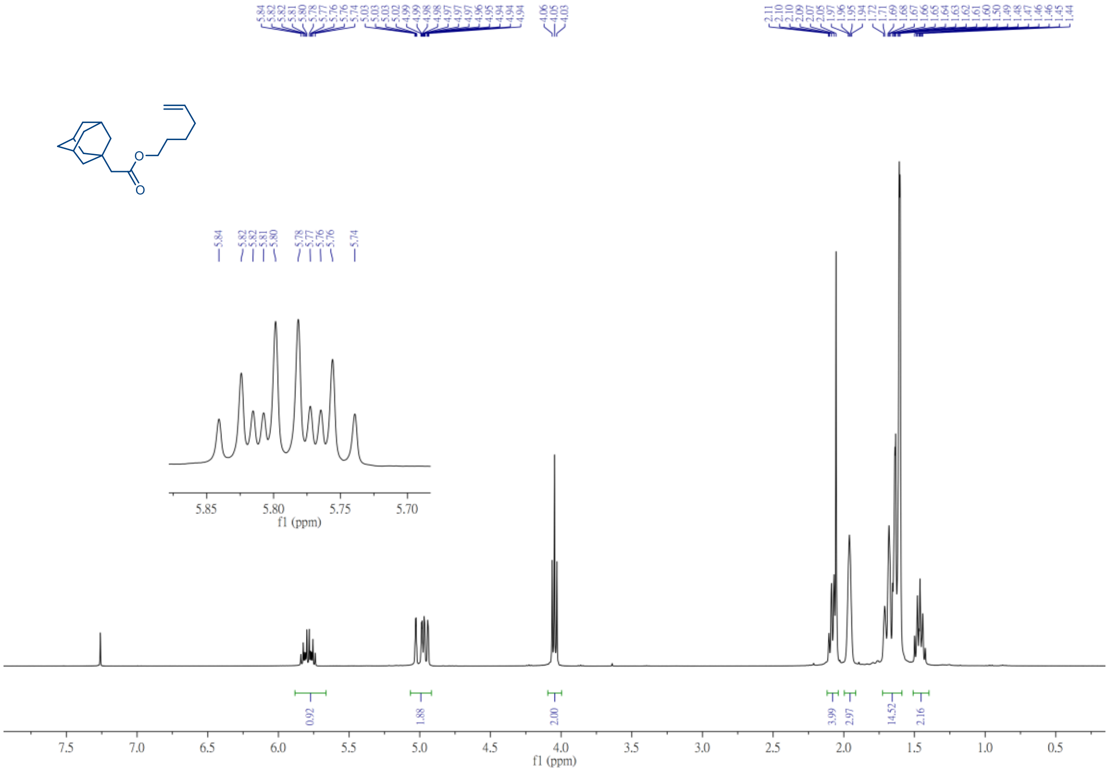

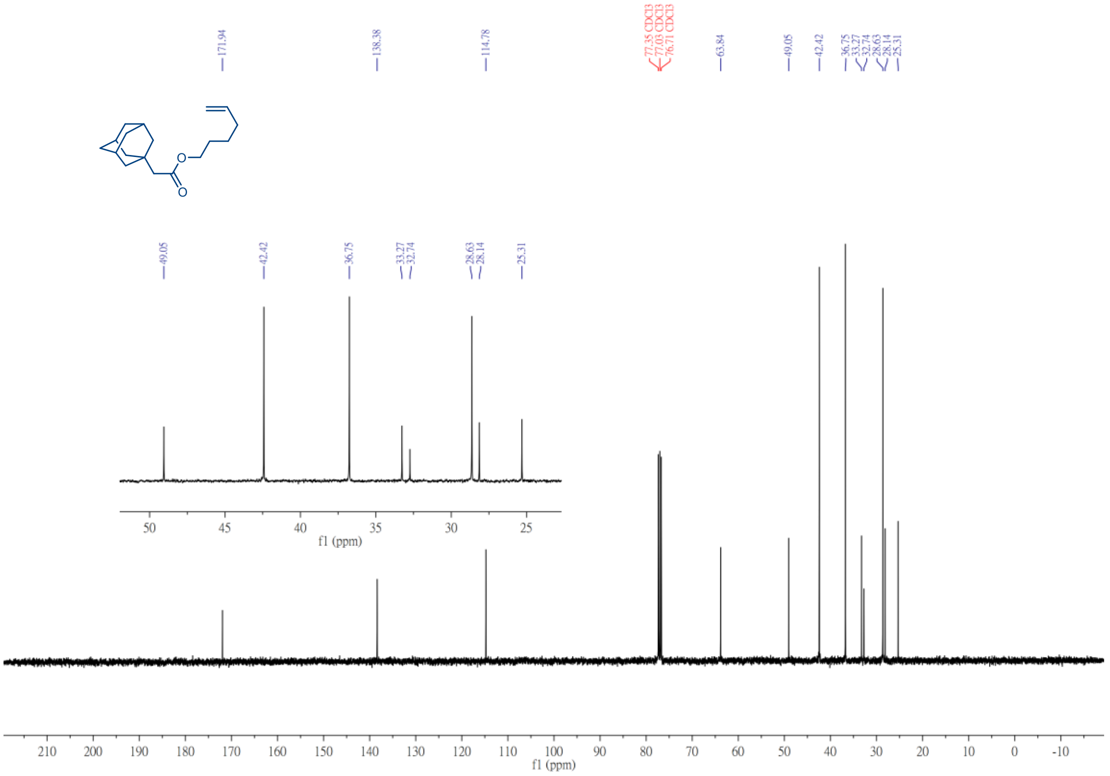

^{1}H and ^{13}C NMR spectrum of **S76**

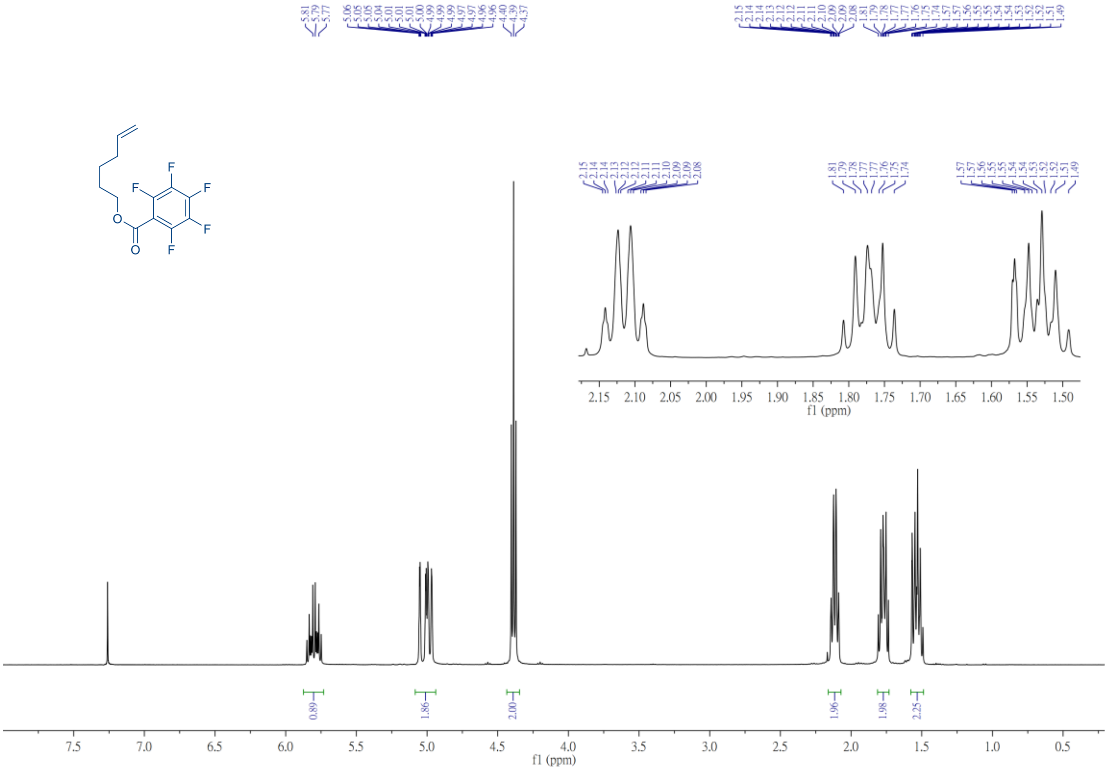

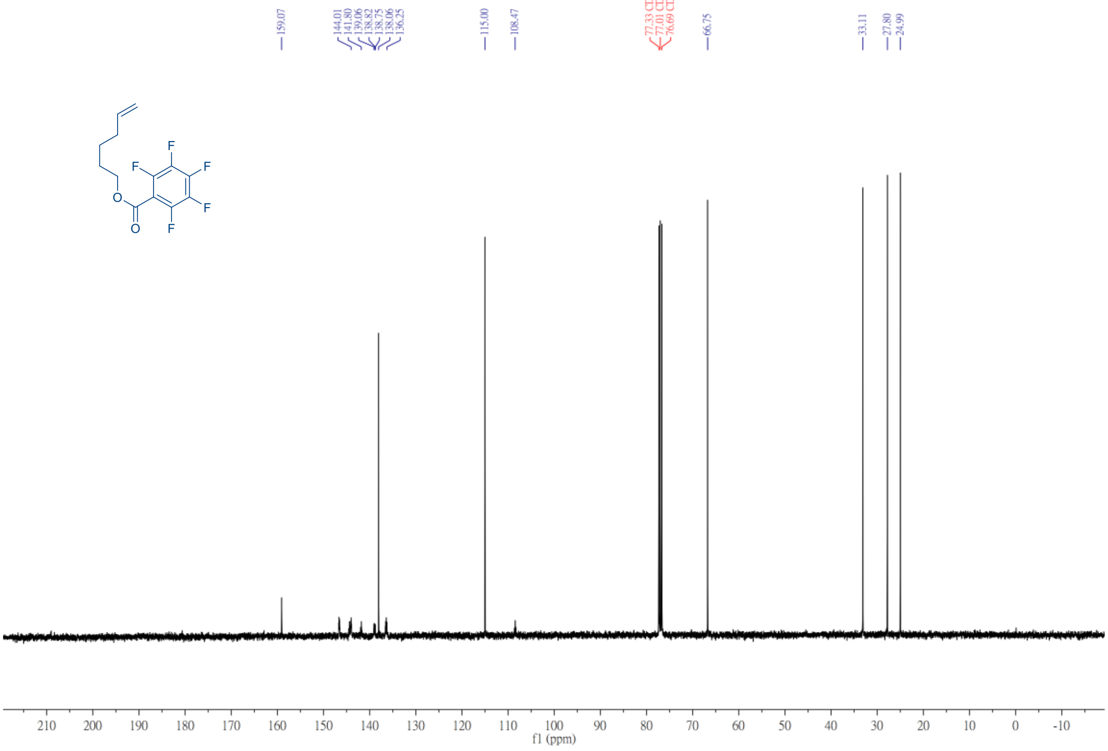

^{19}F NMR spectrum of **S76**

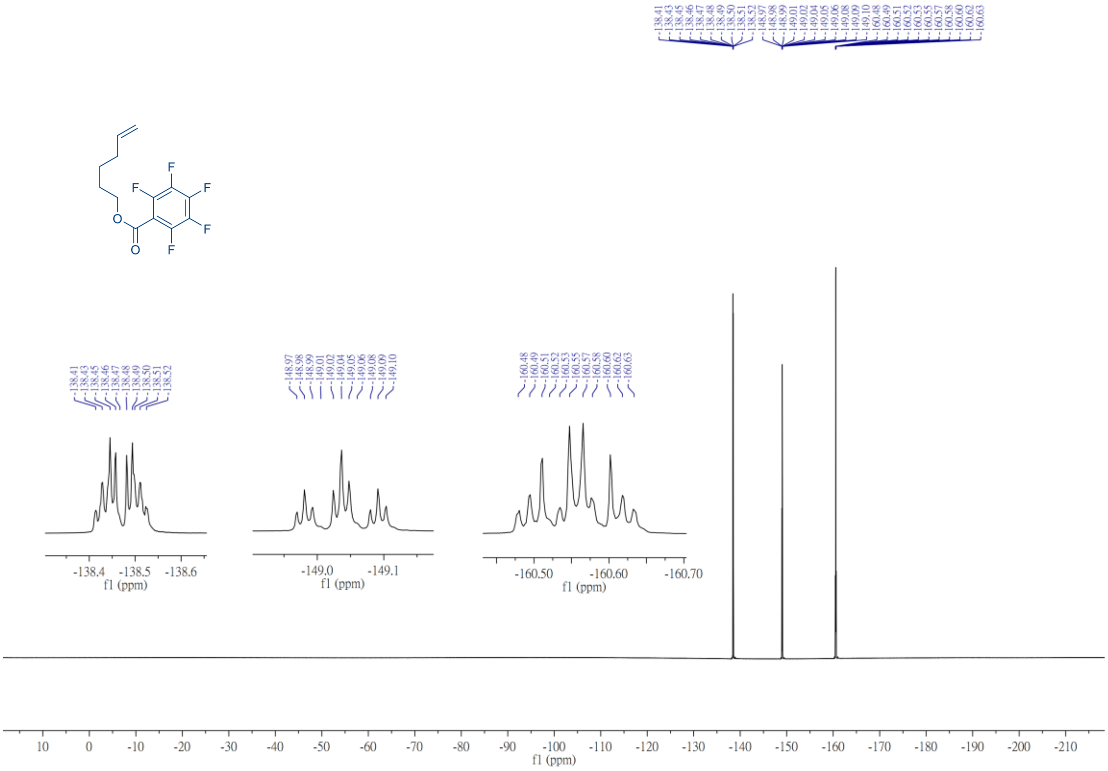

^1H and ^{13}C NMR spectrum of **S77**

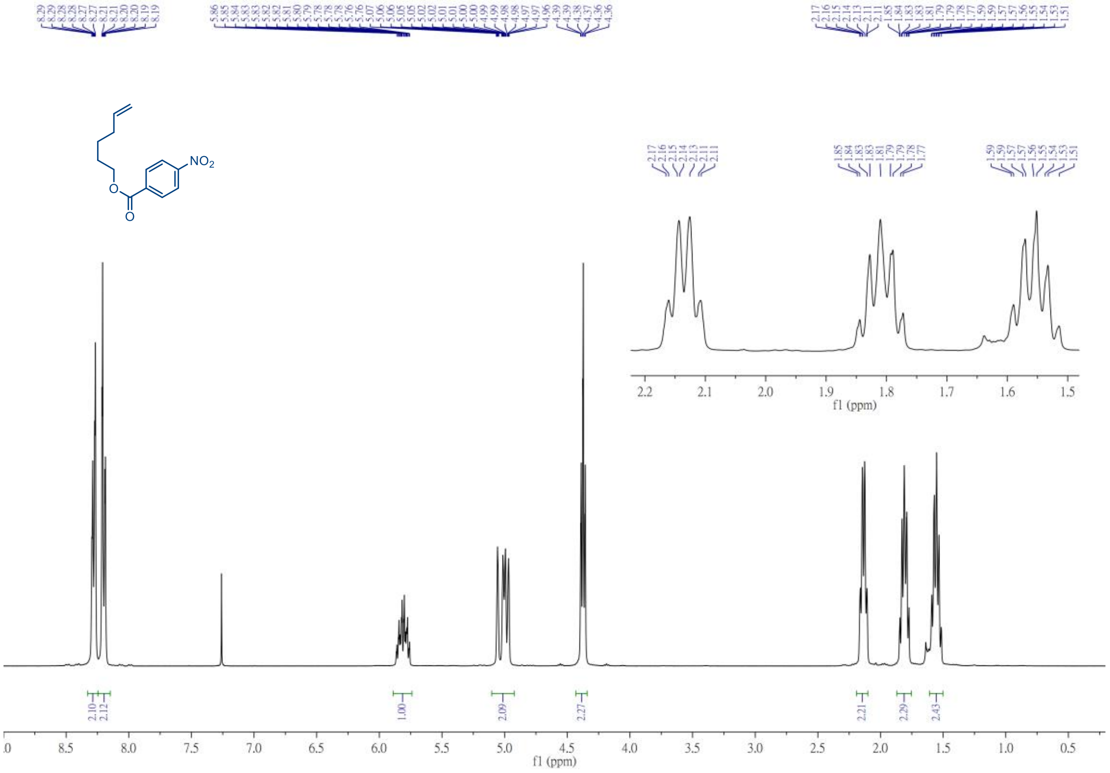

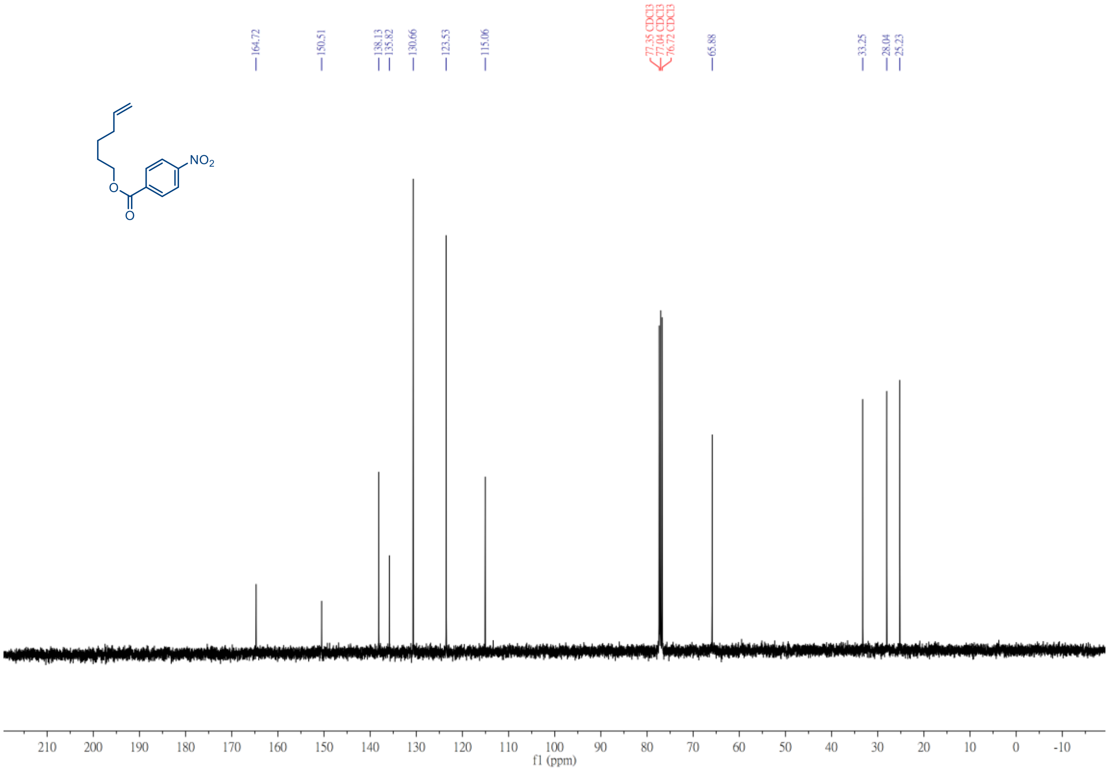

^{1}H and ^{13}C NMR spectrum of **S78**

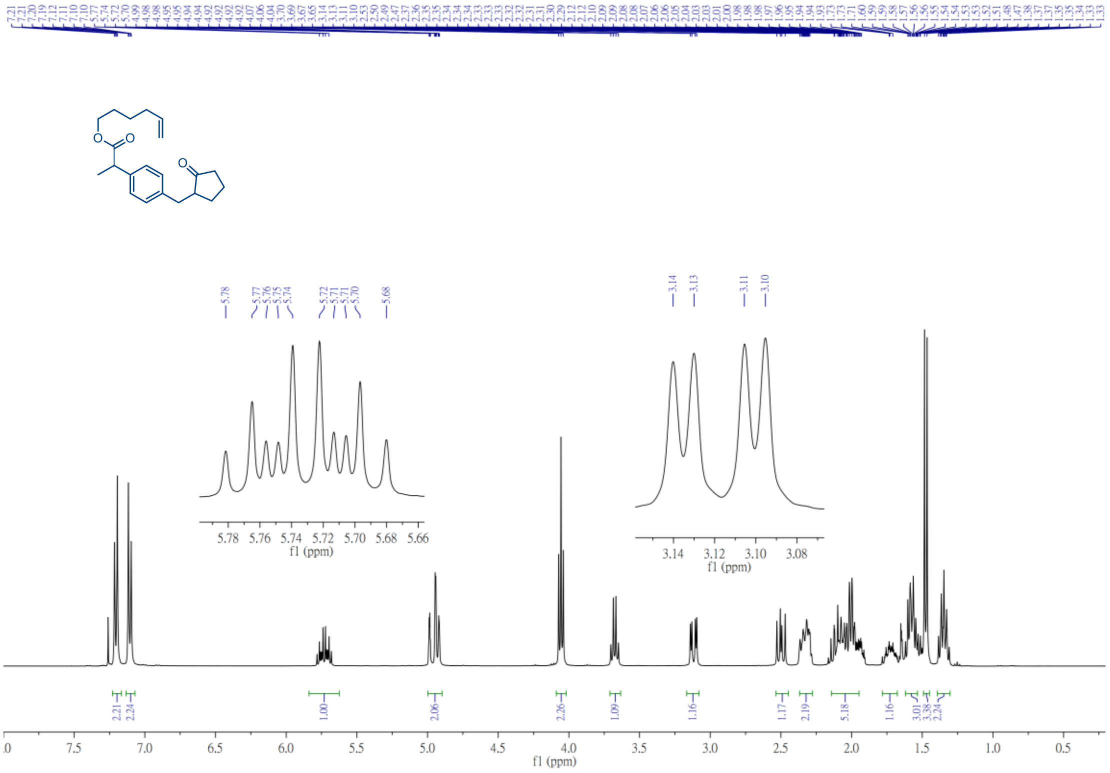

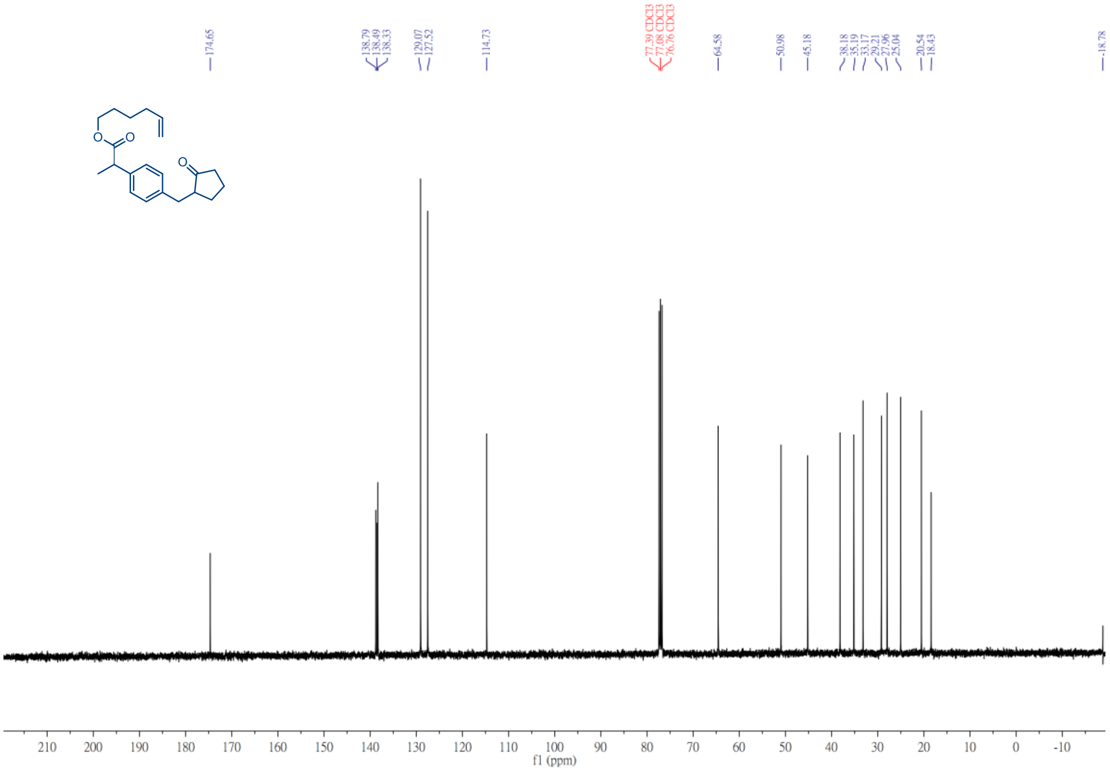

^{1}H and ^{13}C NMR spectrum of **S79**

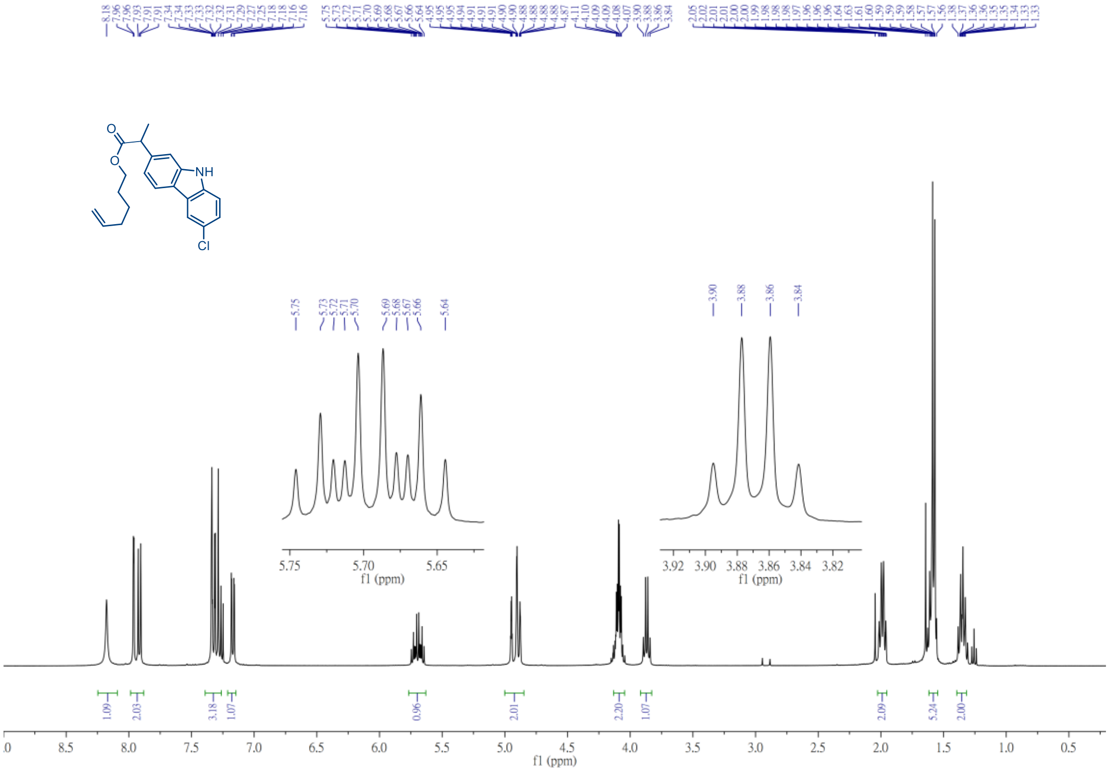

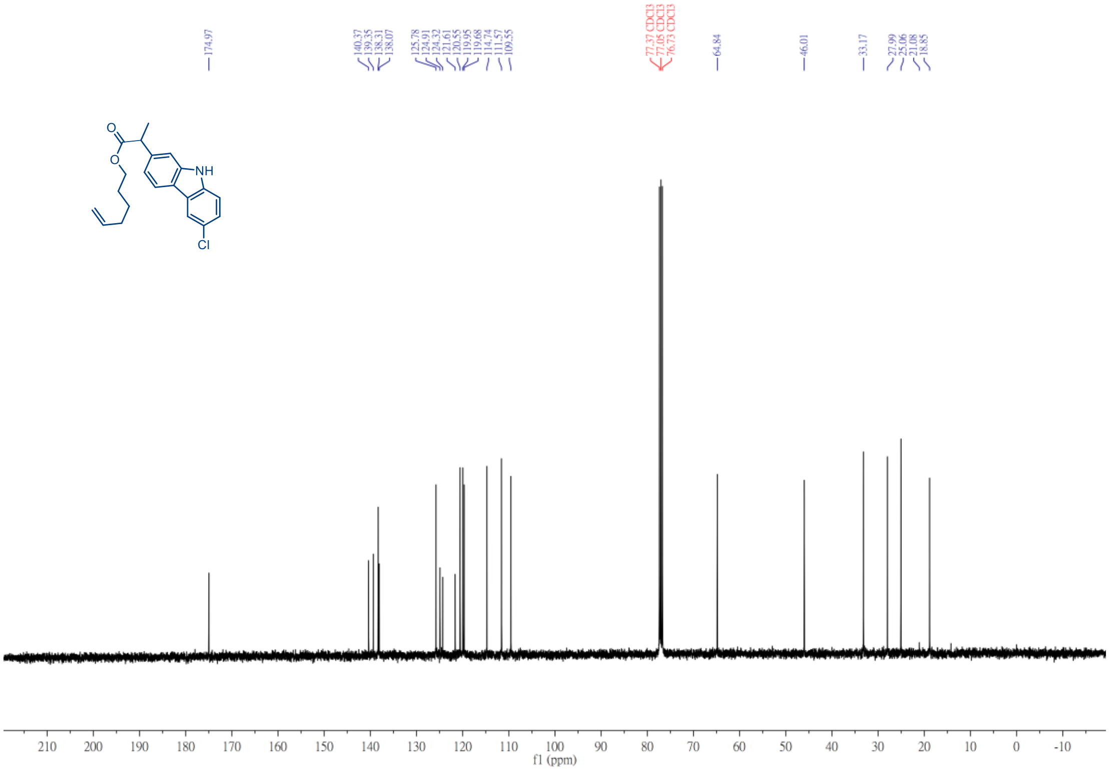

^{1}H and ^{13}C NMR spectrum of **S80**

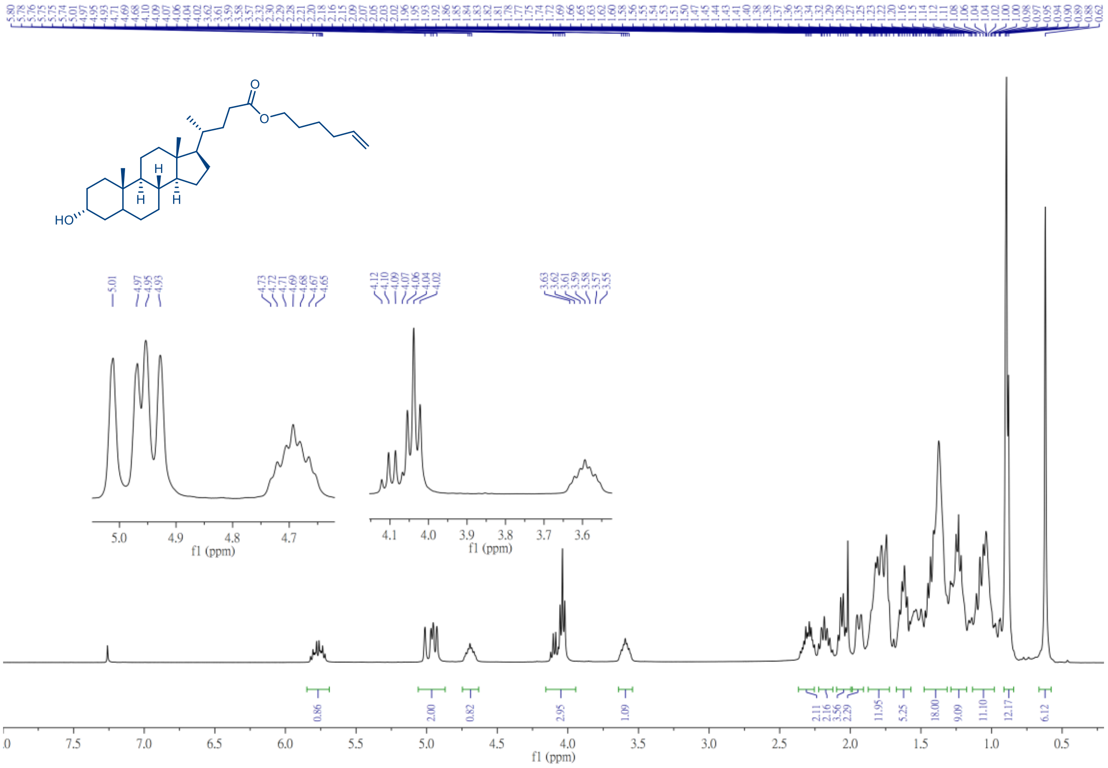

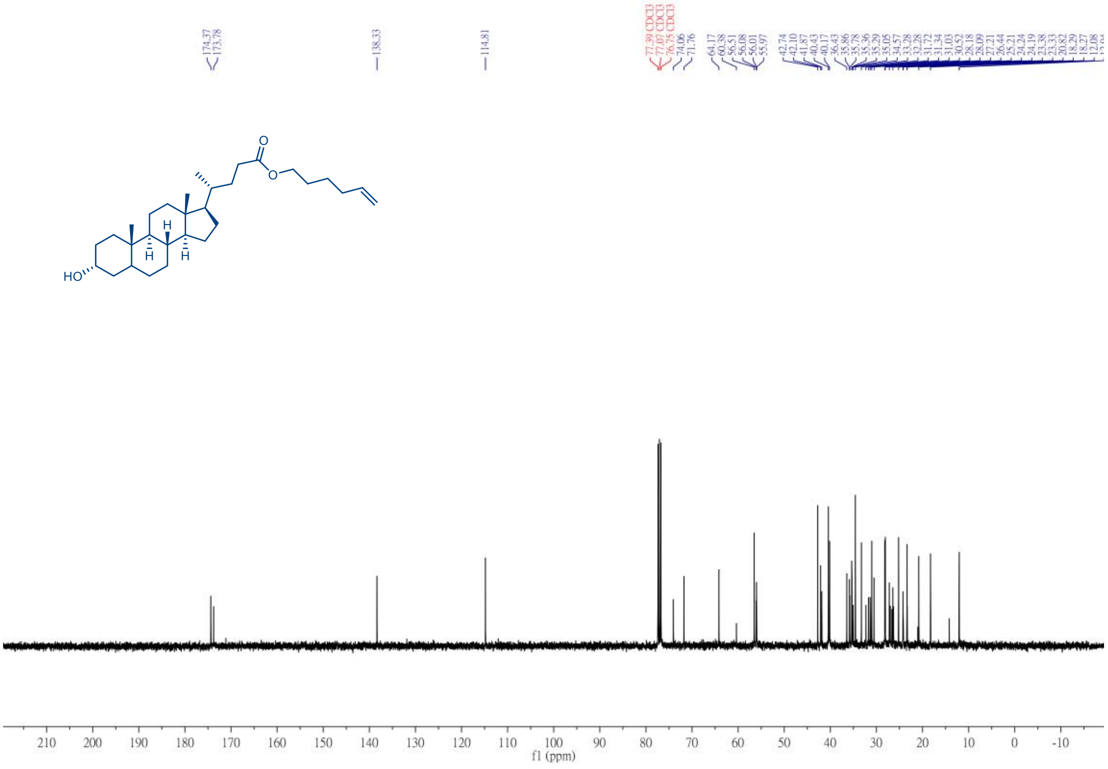

^{1}H and ^{13}C NMR spectrum of **S81**

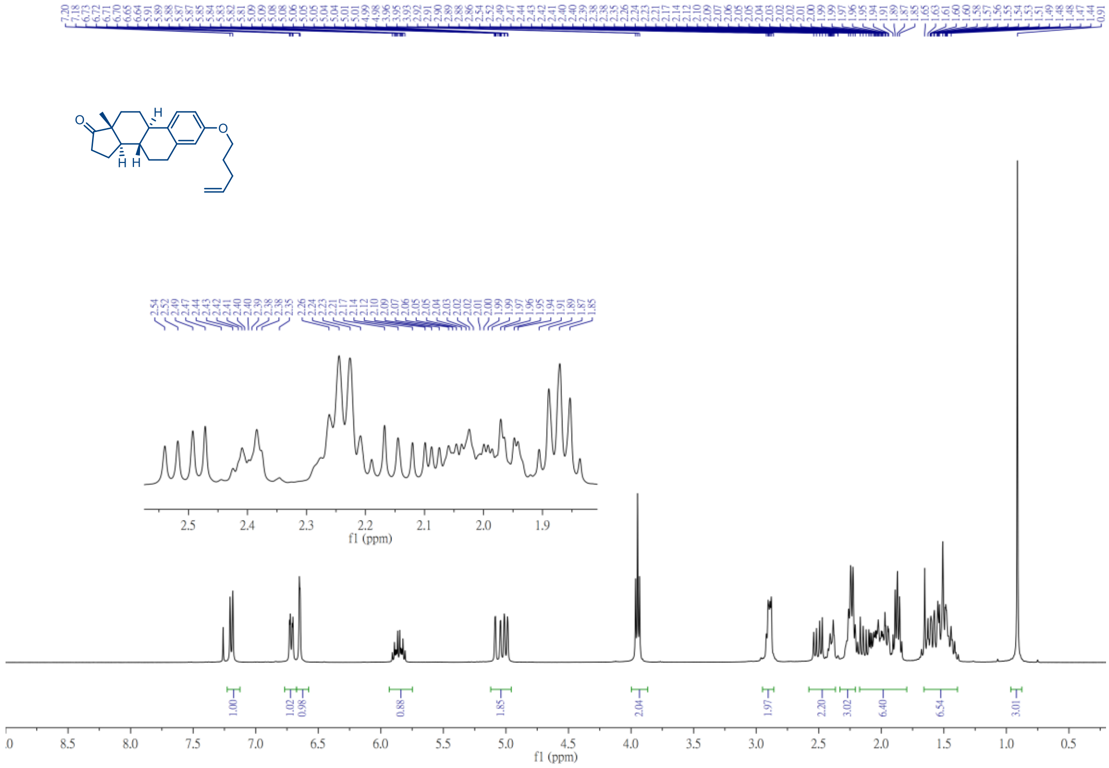

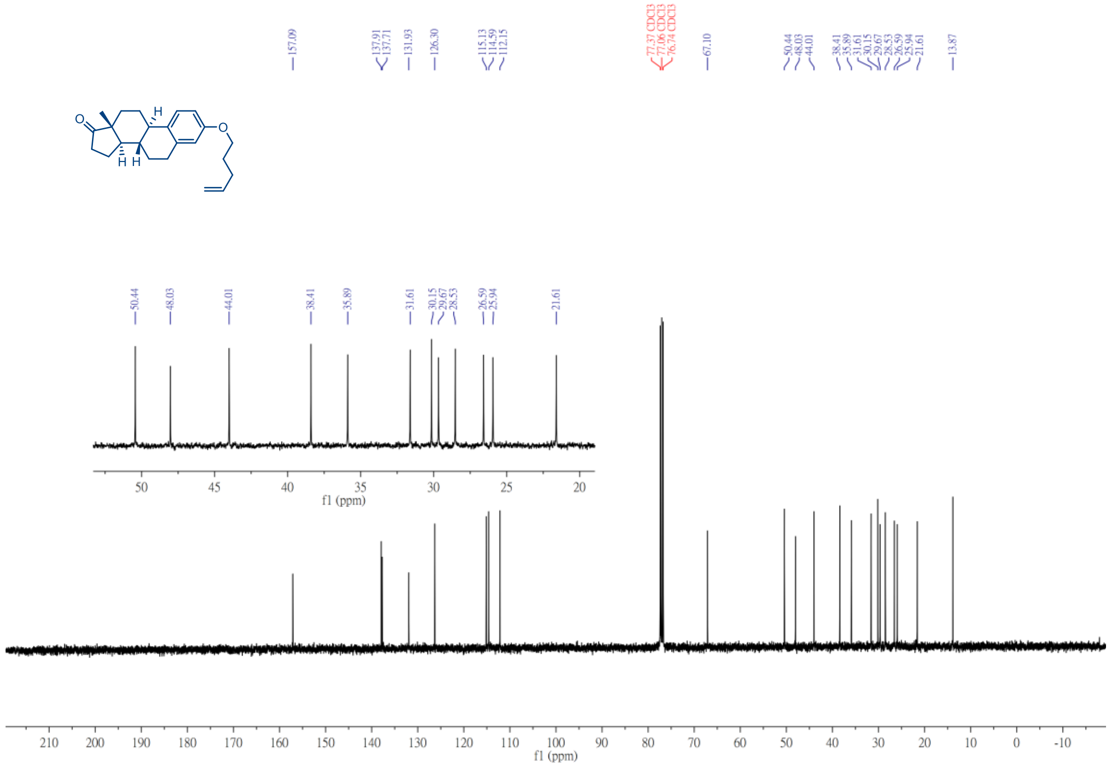

^{1}H and ^{13}C NMR spectrum of **5**

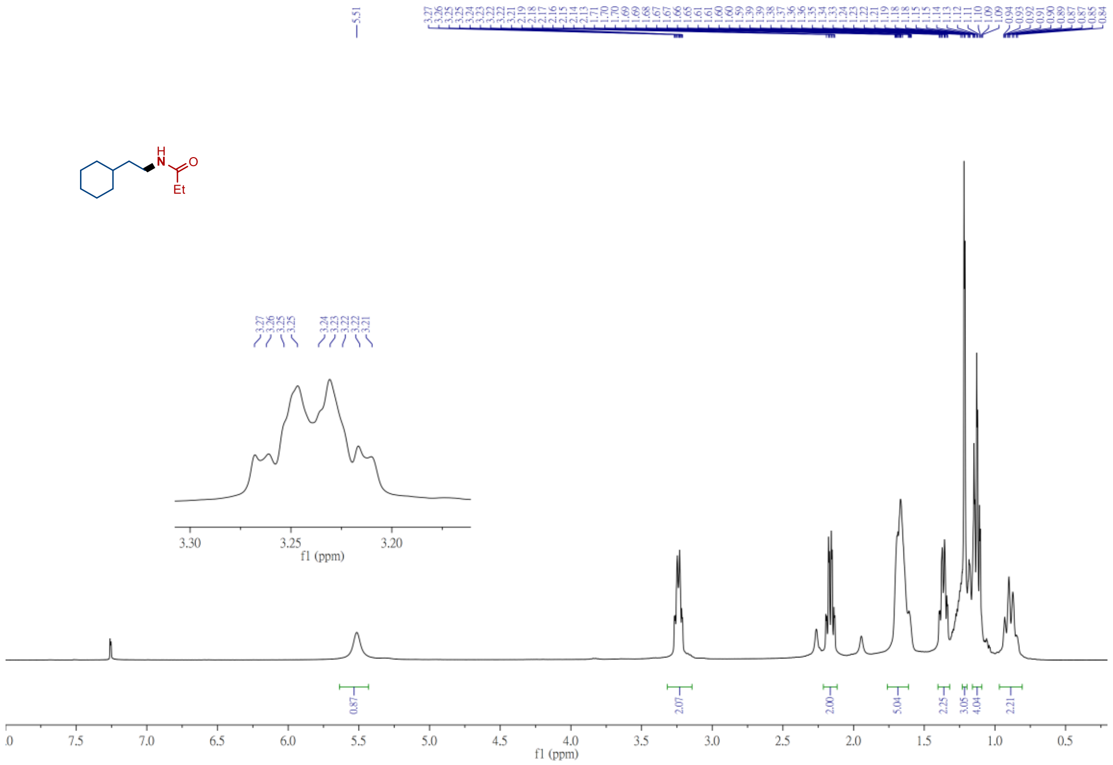

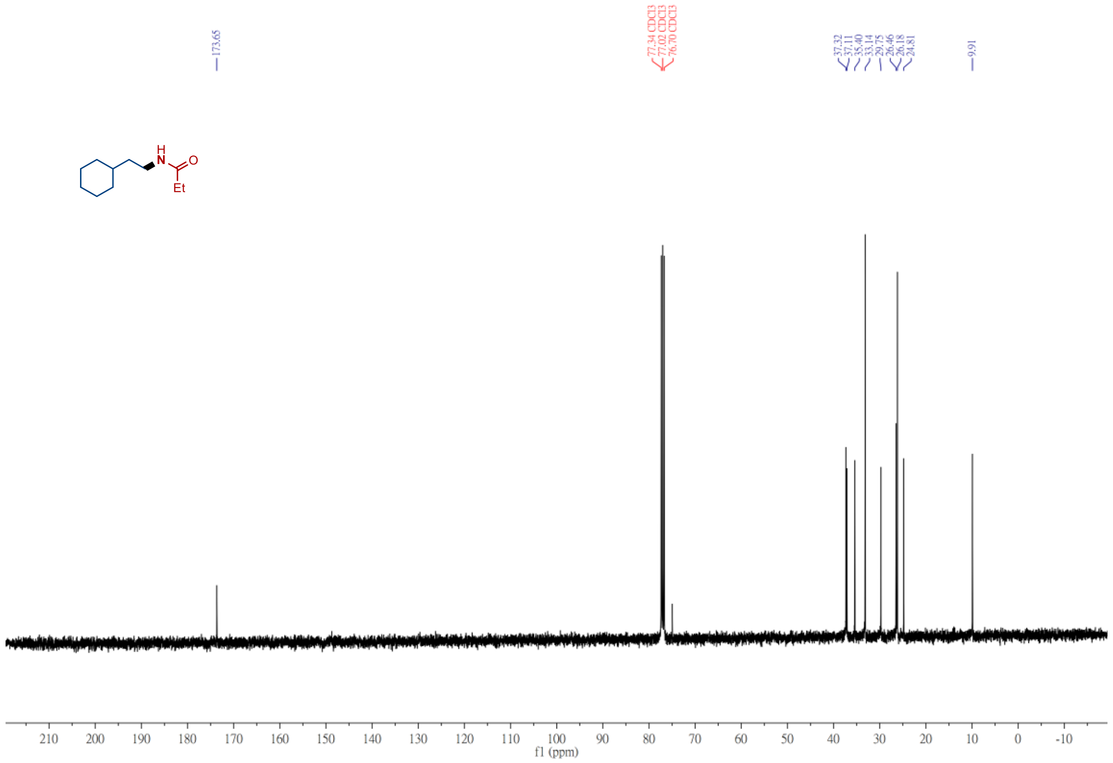

^{1}H and ^{13}C NMR spectrum of **6**

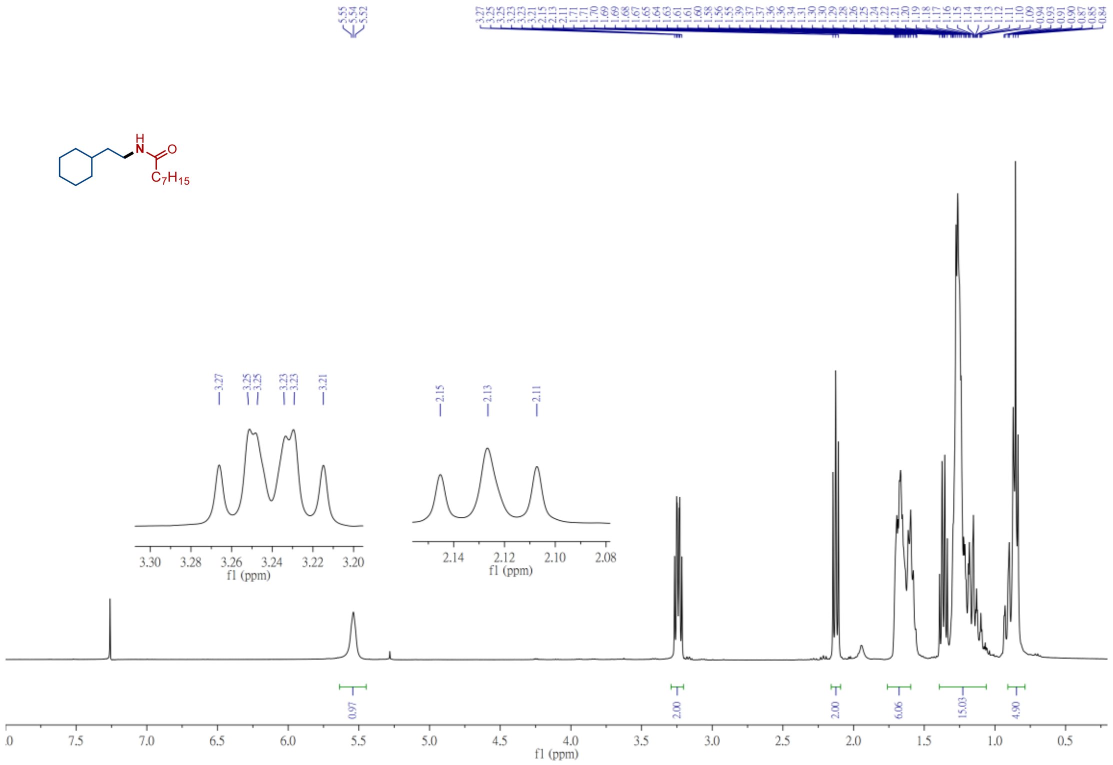

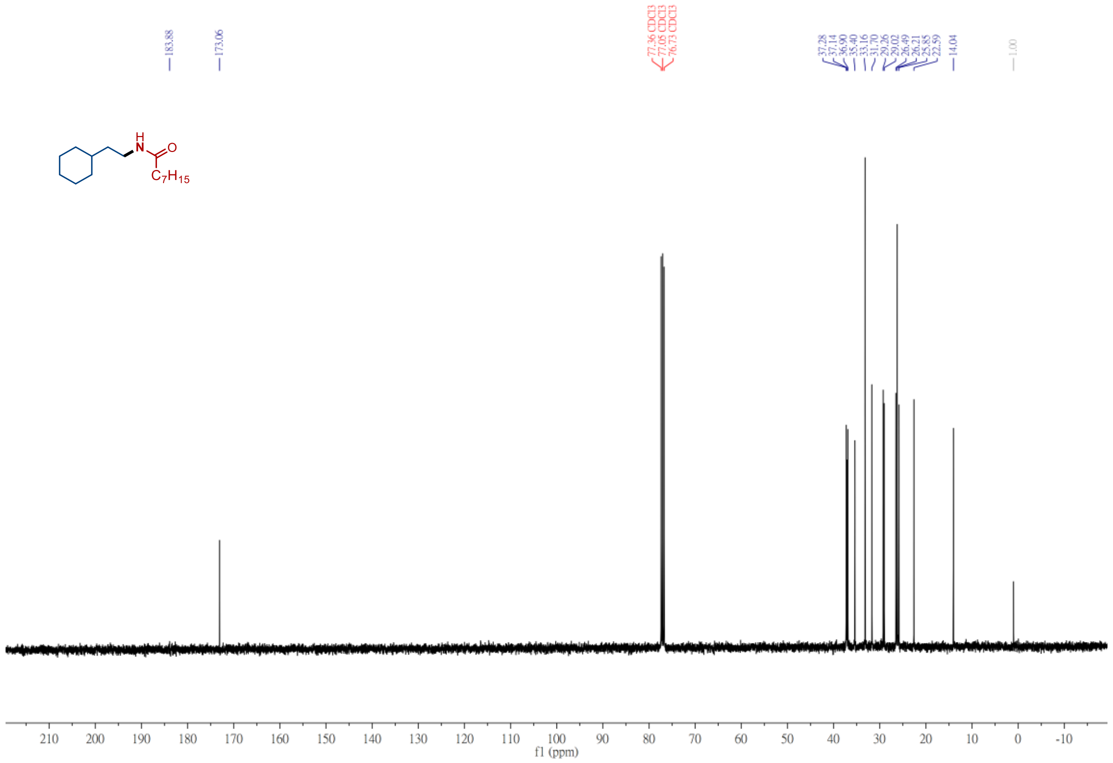

^{1}H and ^{13}C NMR spectrum of **7**

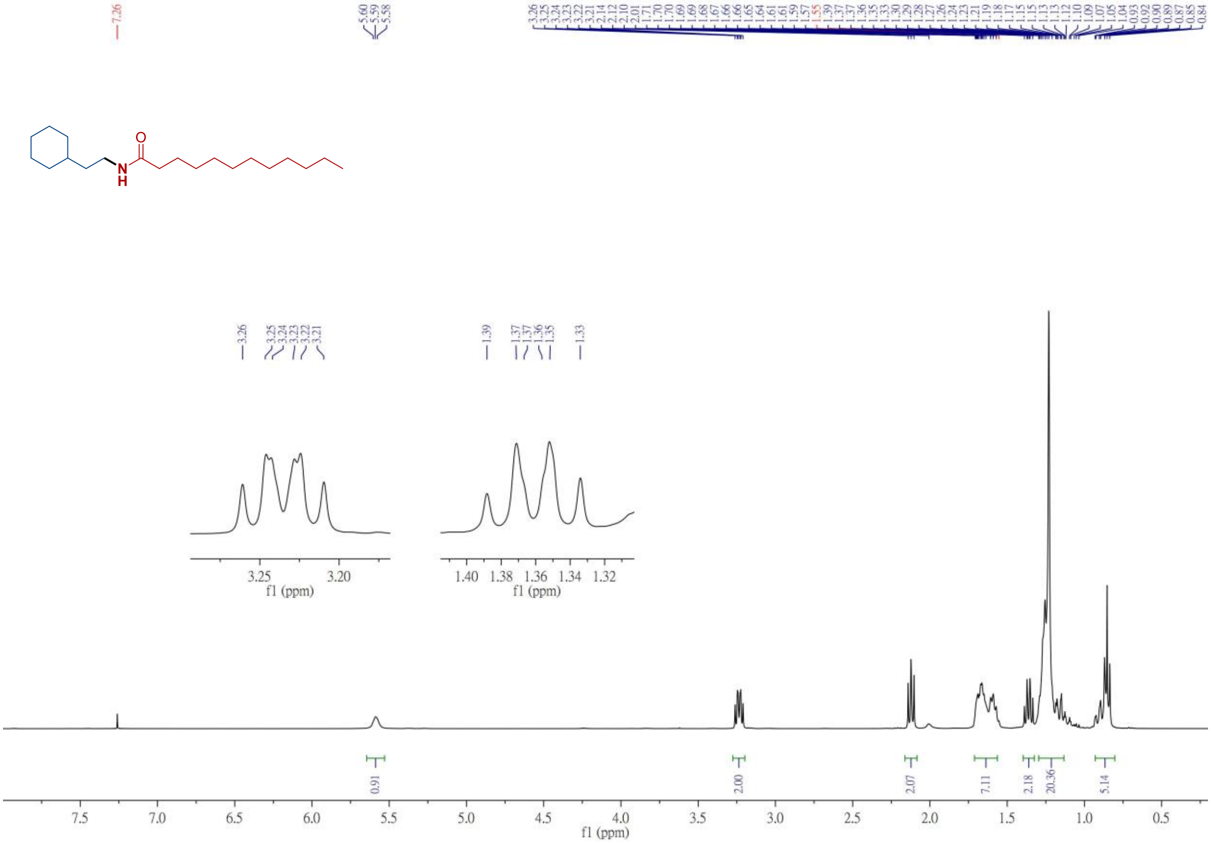

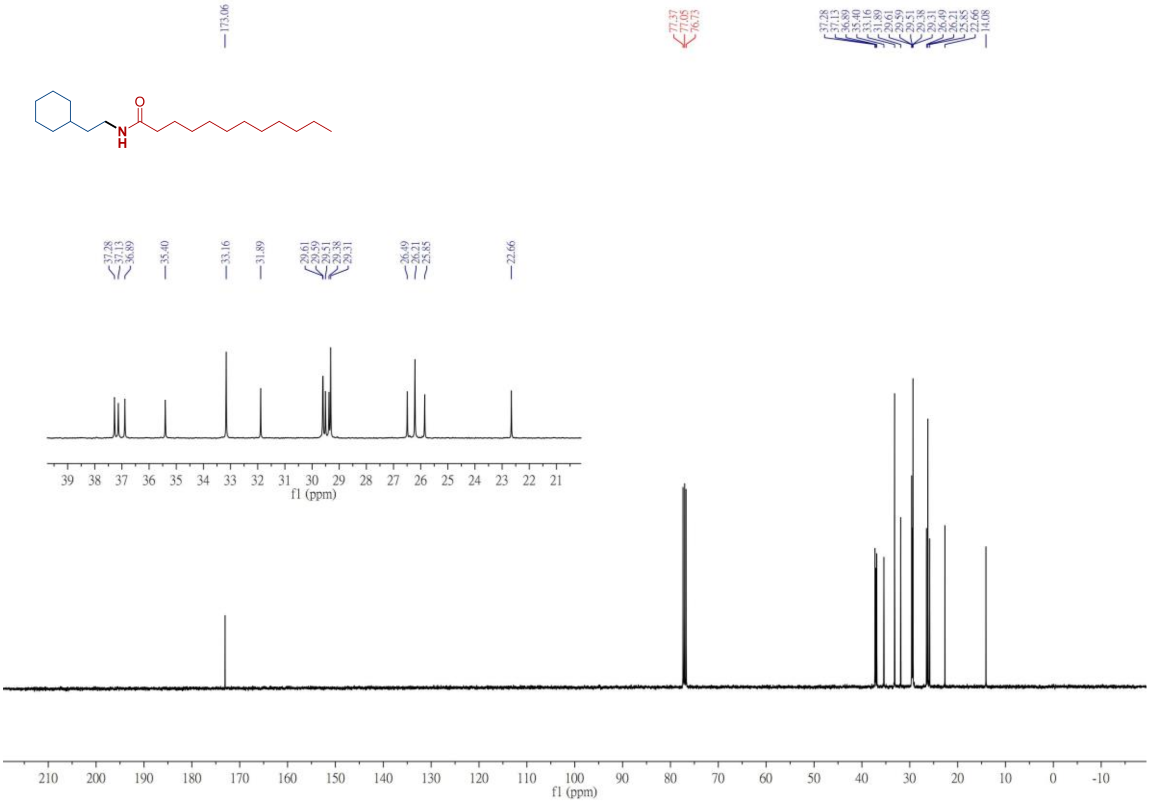

^{1}H and ^{13}C NMR spectrum of **8**

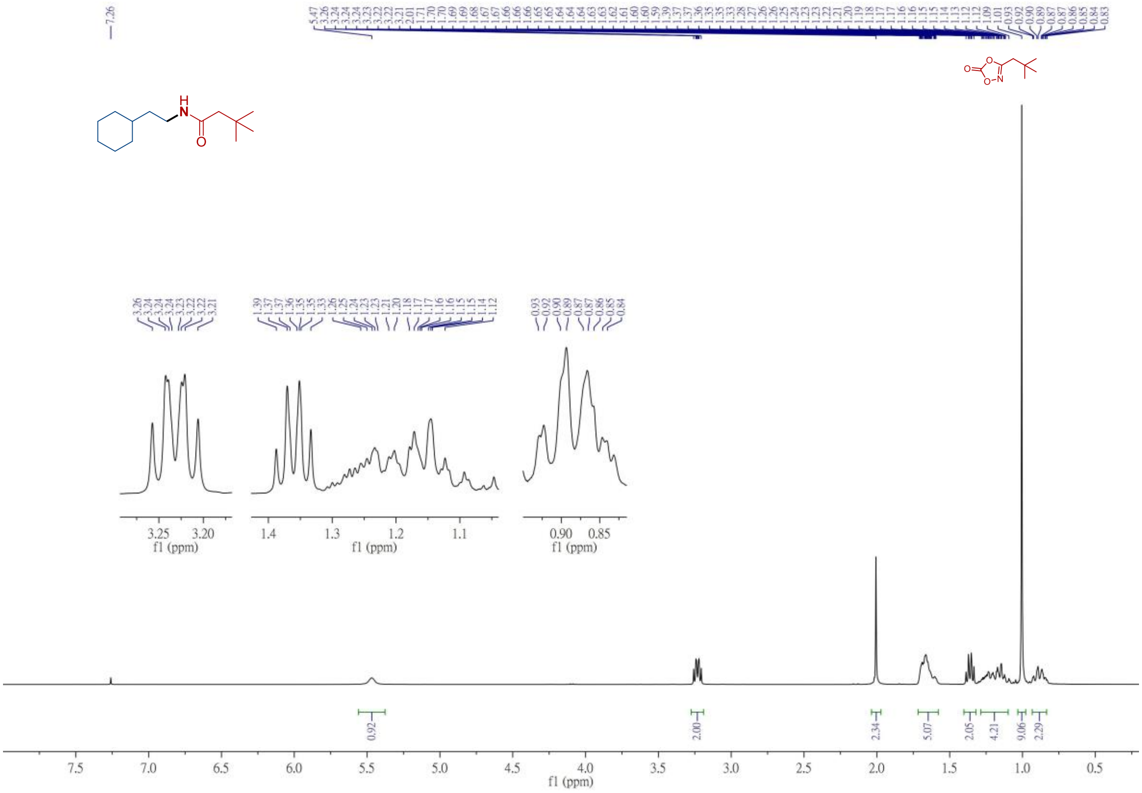

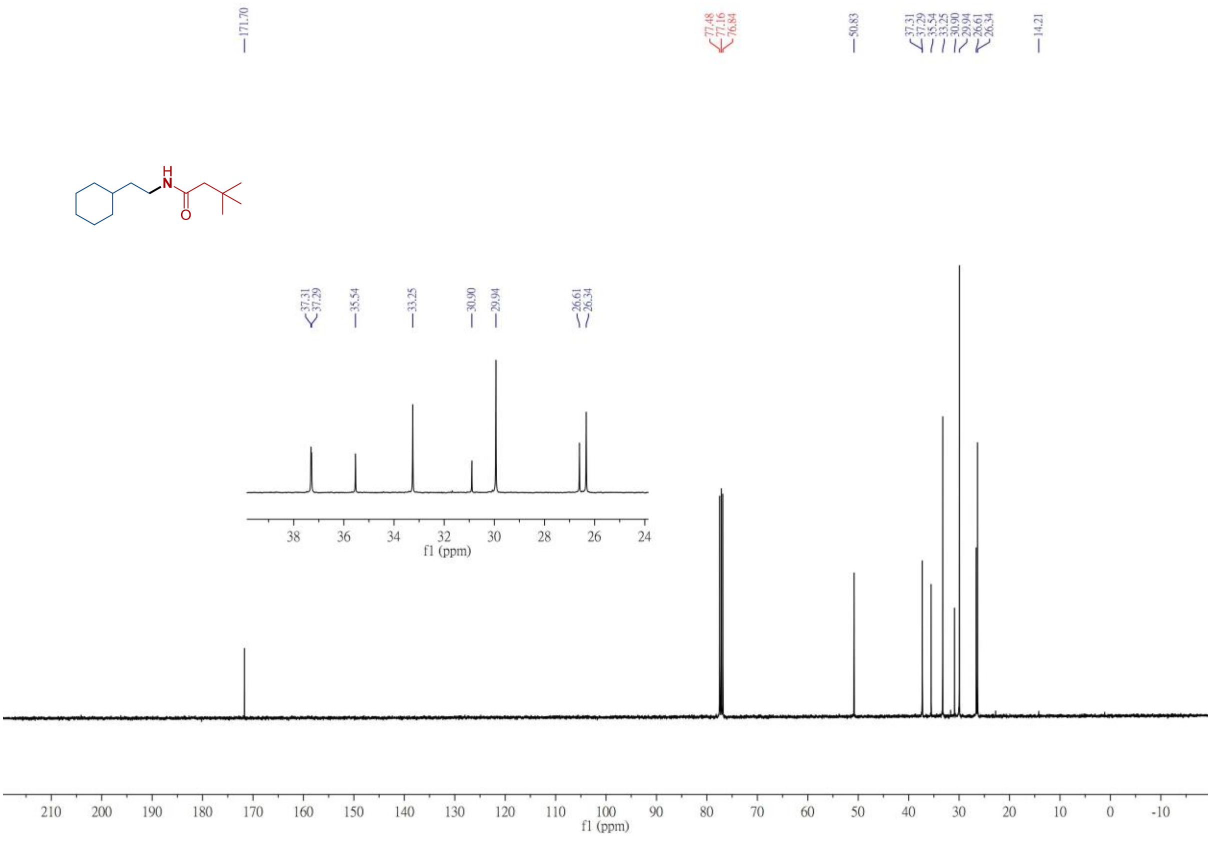

^{1}H and ^{13}C NMR spectrum of **9**

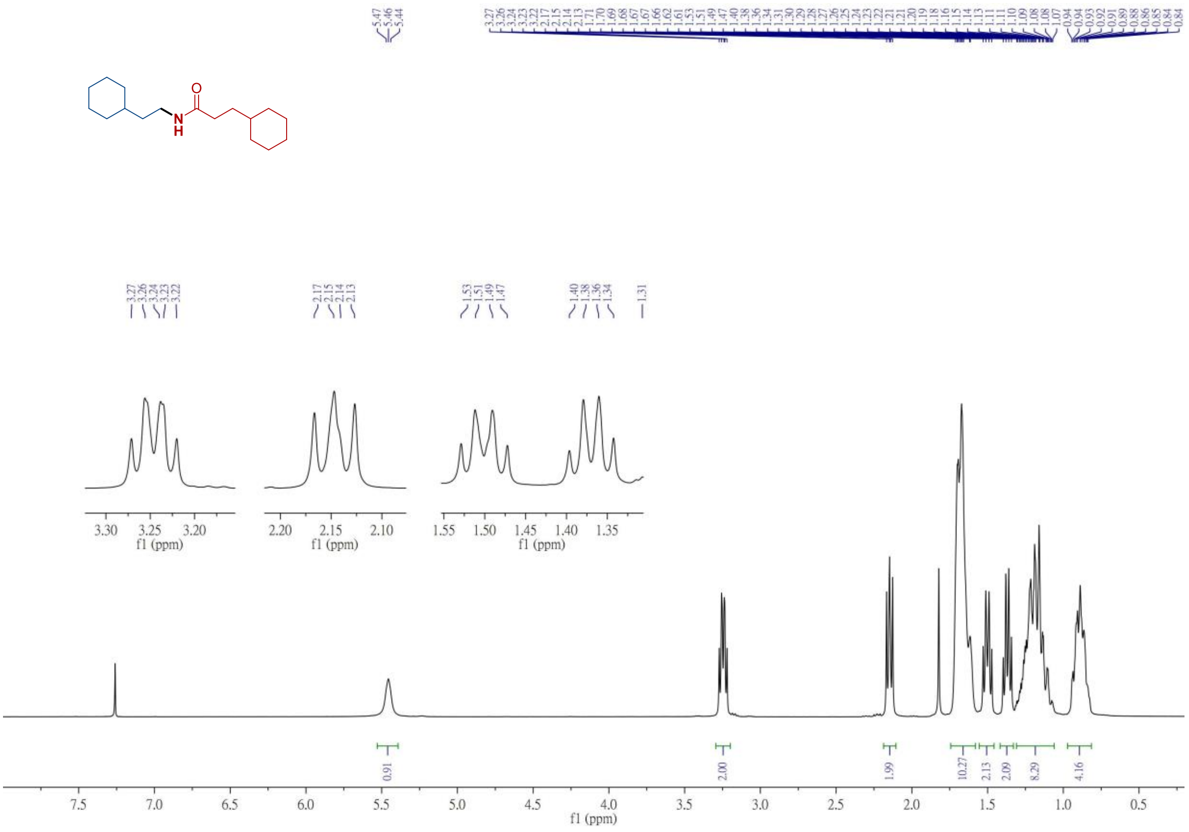

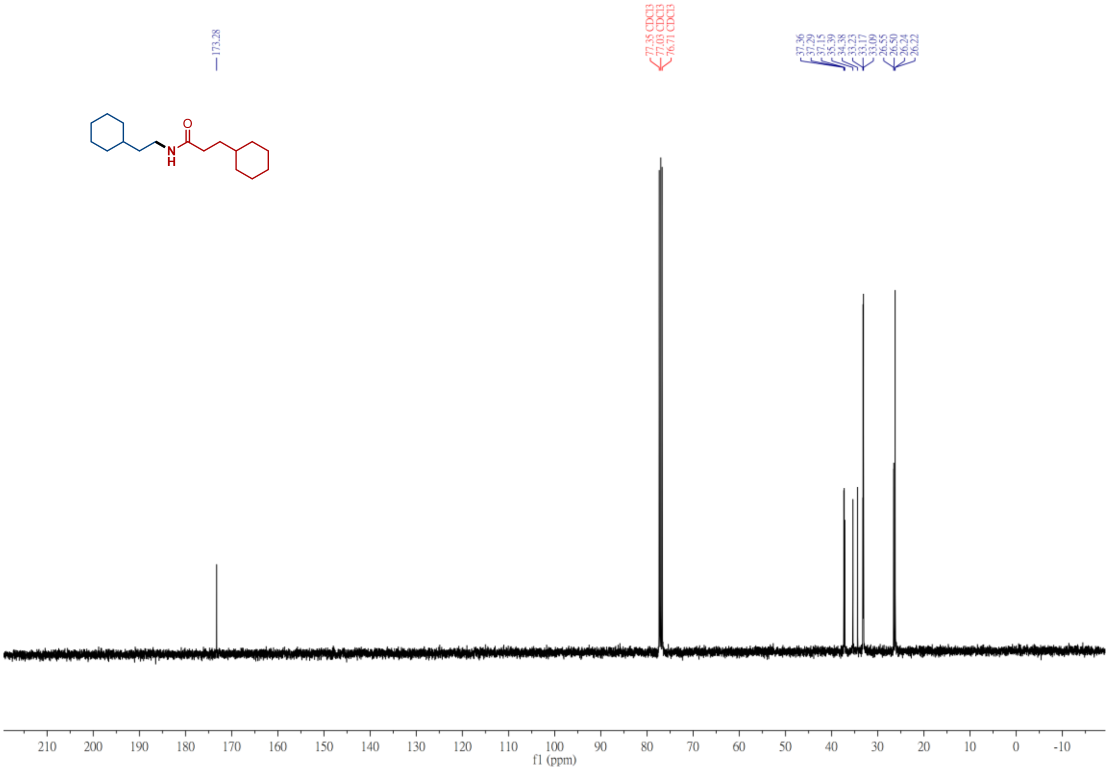

^{1}H and ^{13}C NMR spectrum of **10**

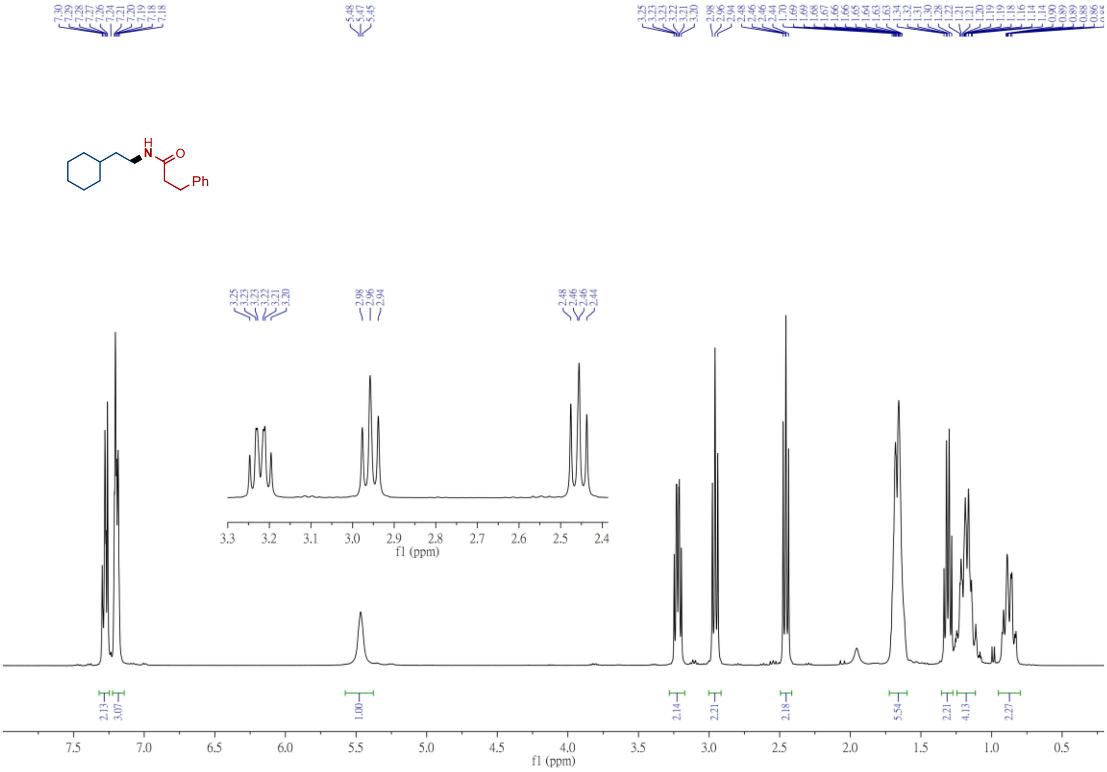

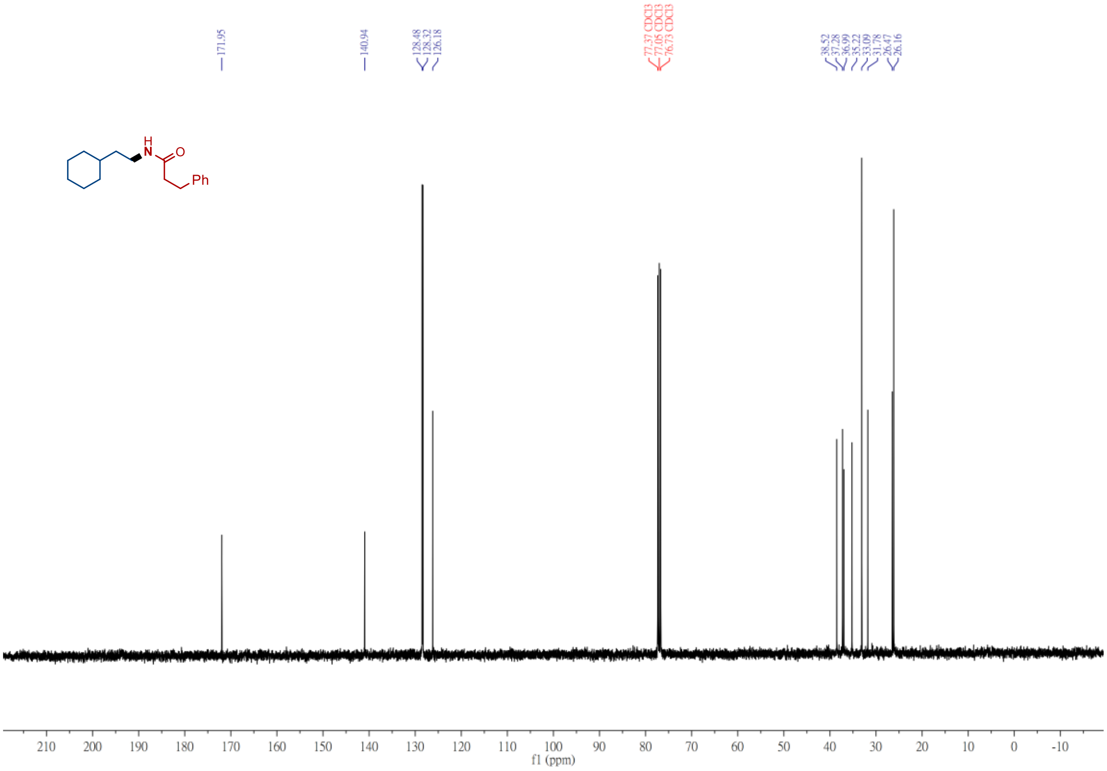

^{1}H and ^{13}C NMR spectrum of **11**

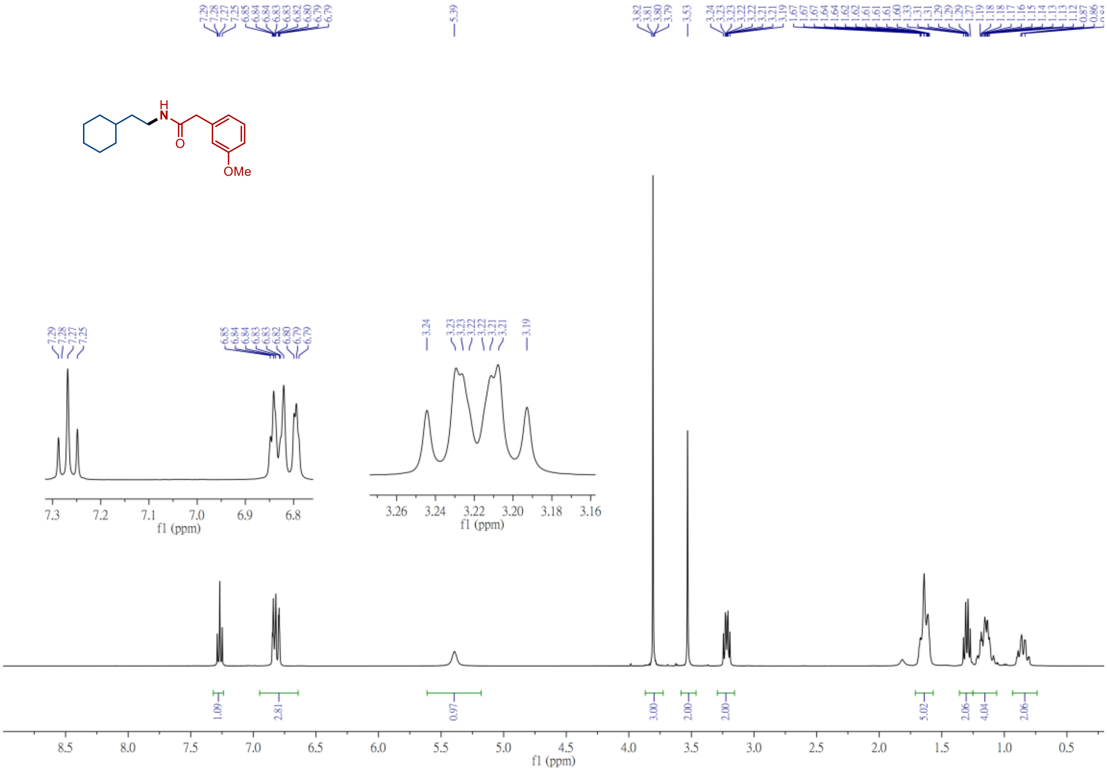

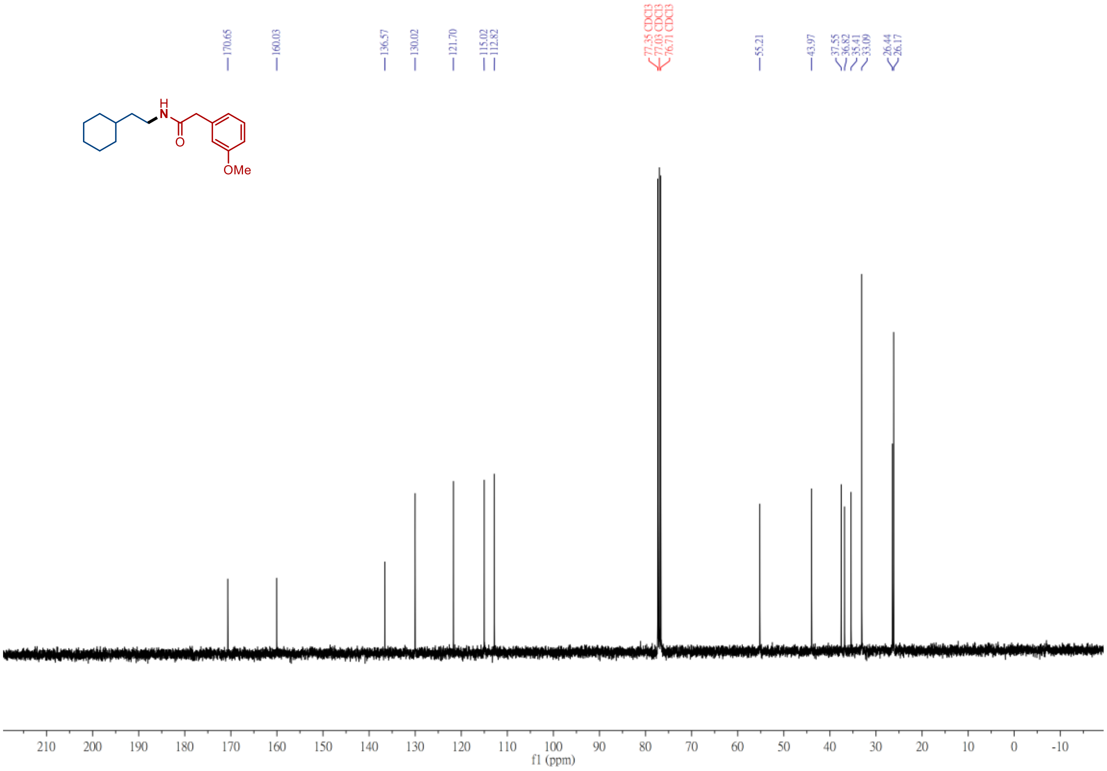

^{1}H and ^{13}C NMR spectrum of **12**

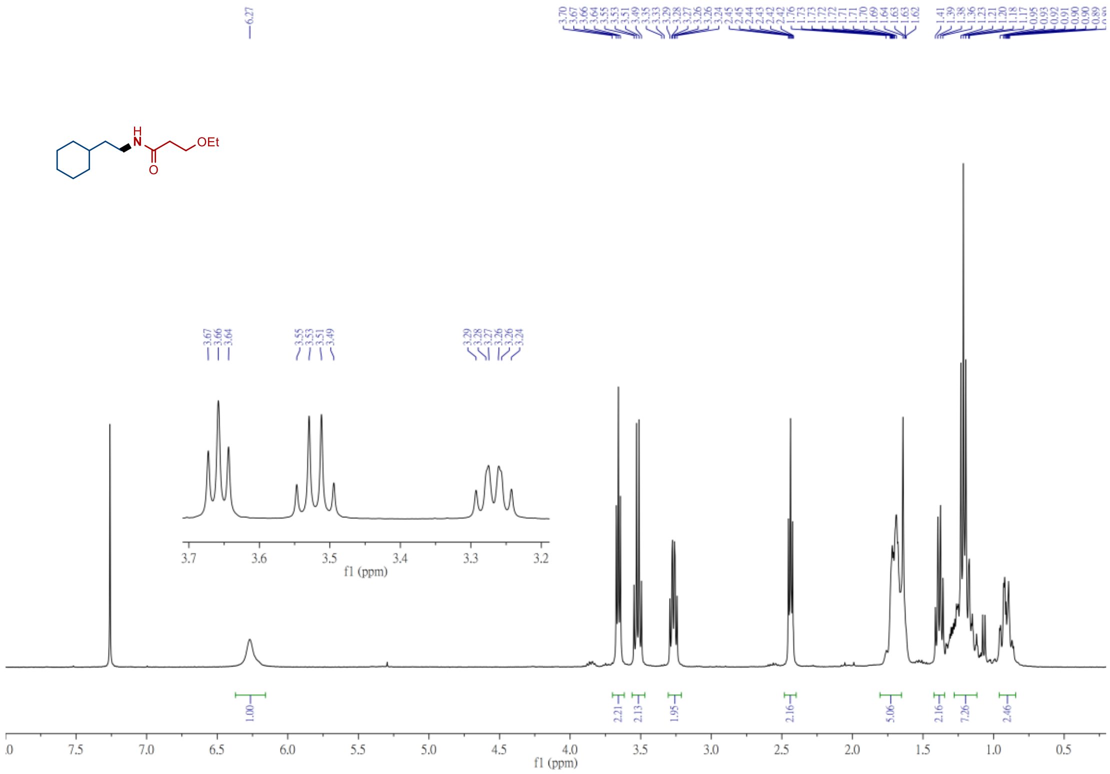

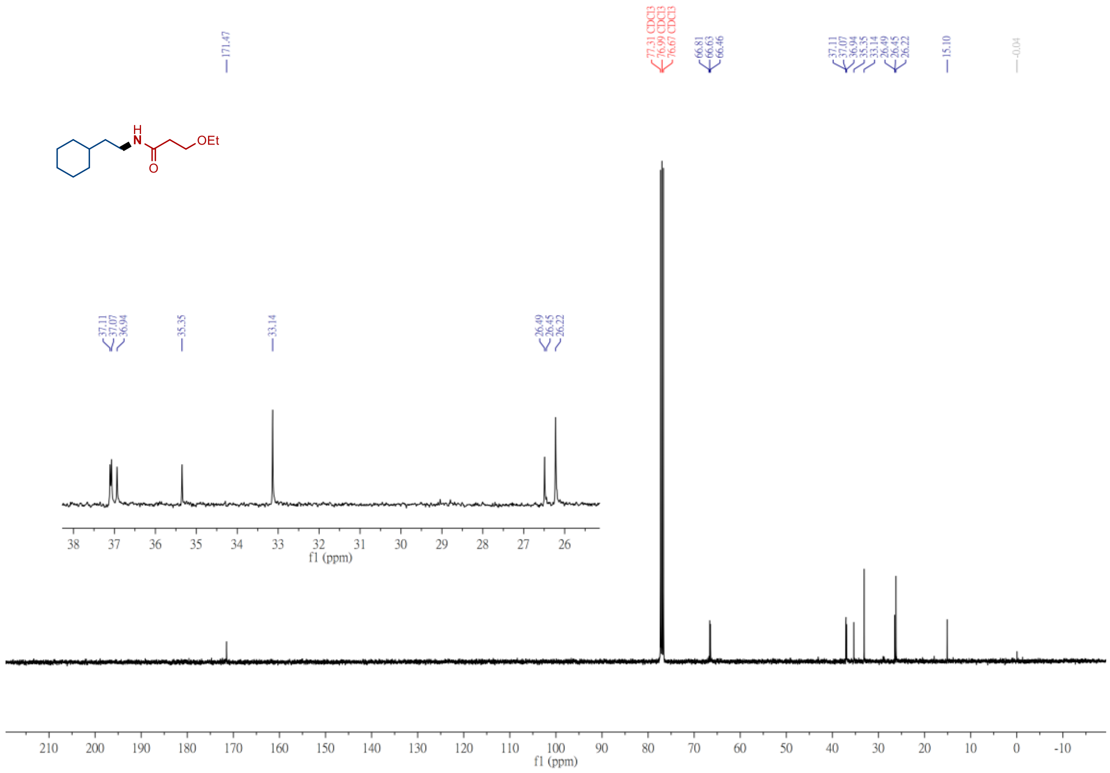

^{1}H and ^{13}C NMR spectrum of **13**

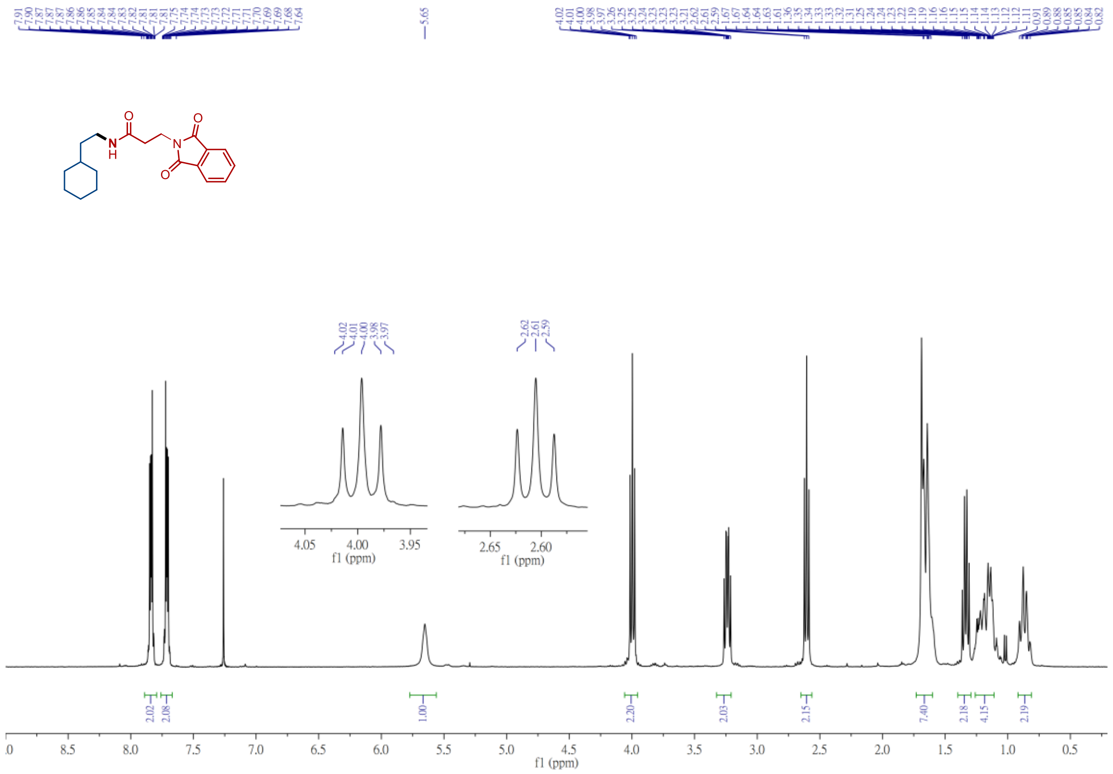

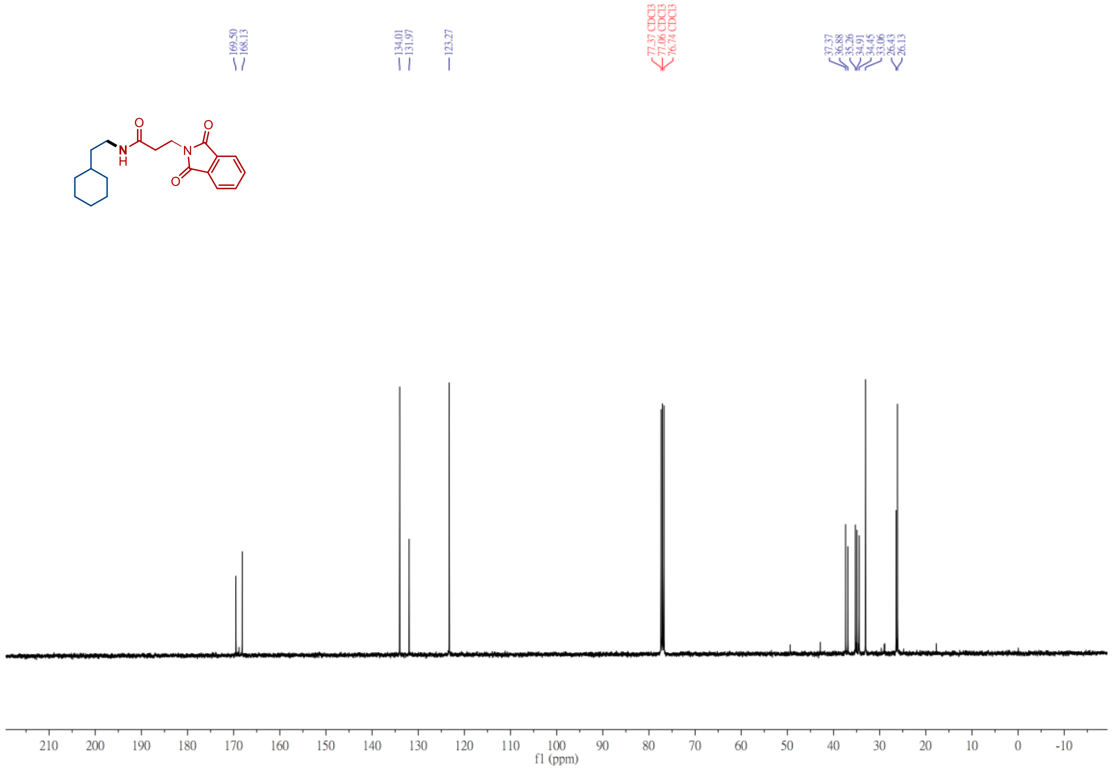

^{1}H and ^{13}C NMR spectrum of **14**

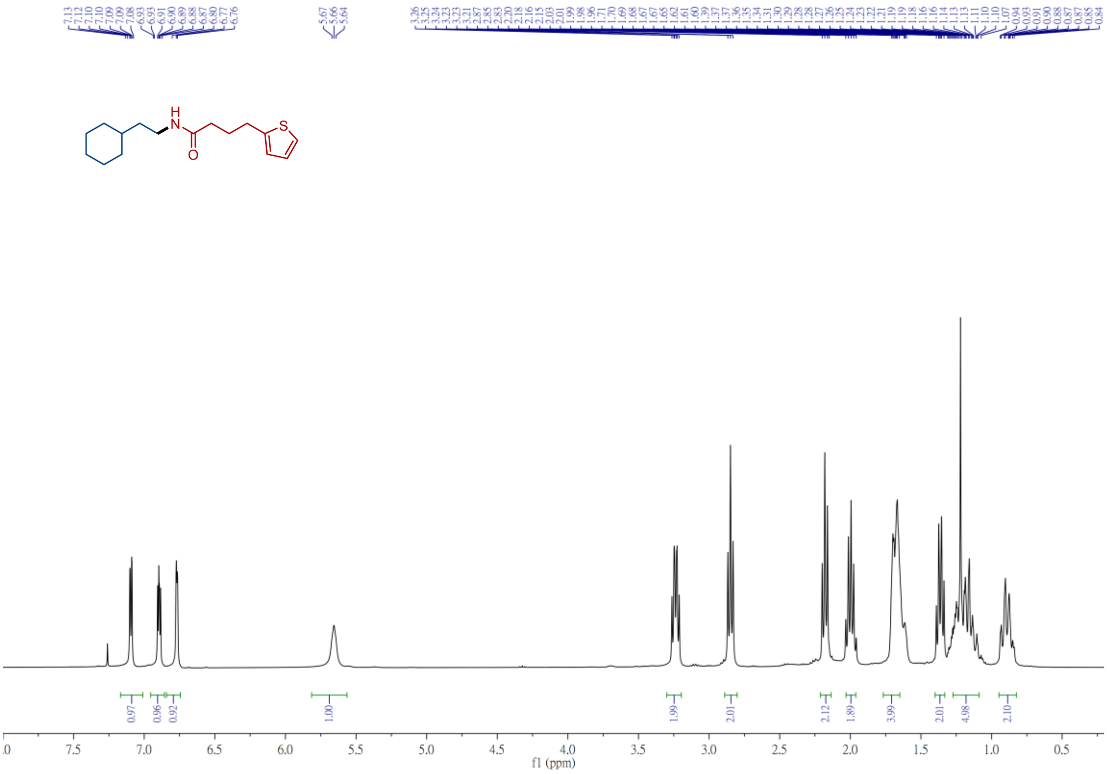

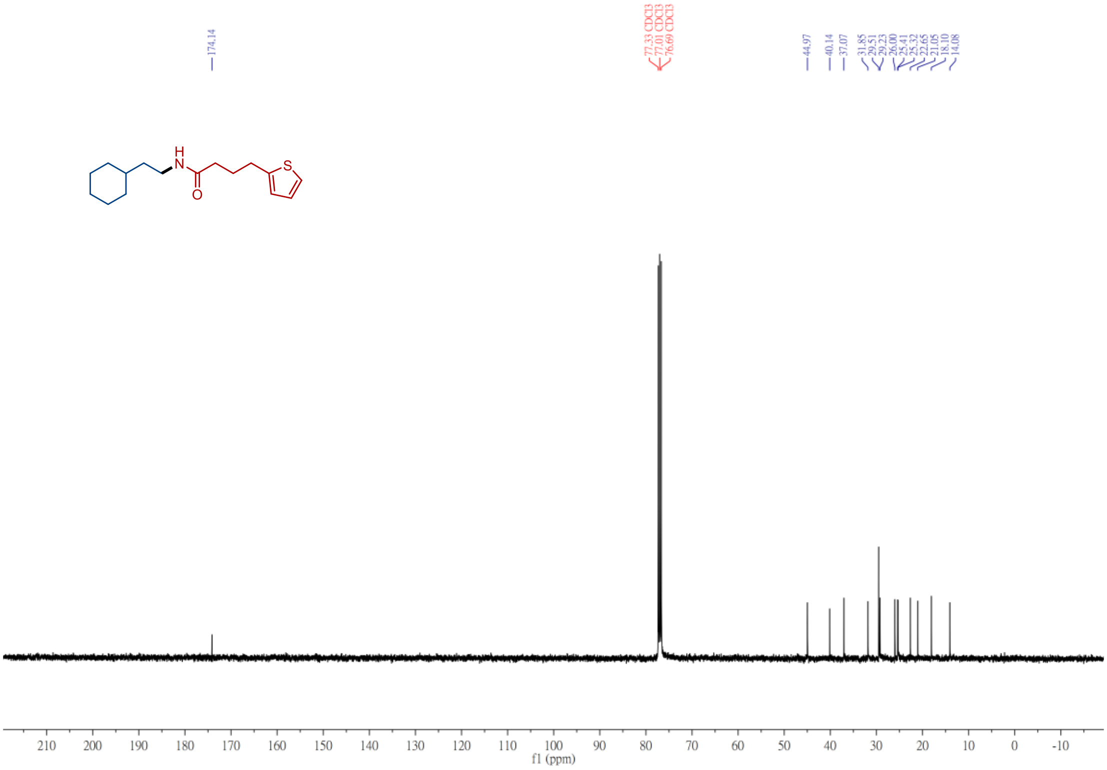

^{1}H and ^{13}C NMR spectrum of **15**

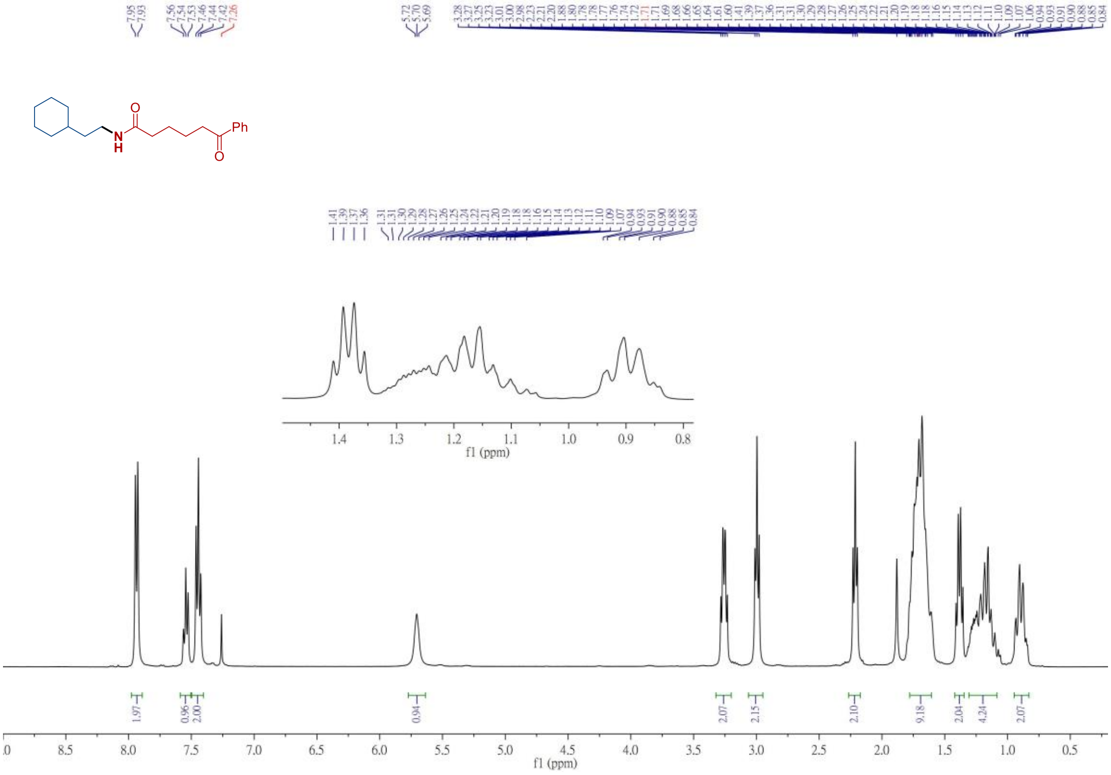

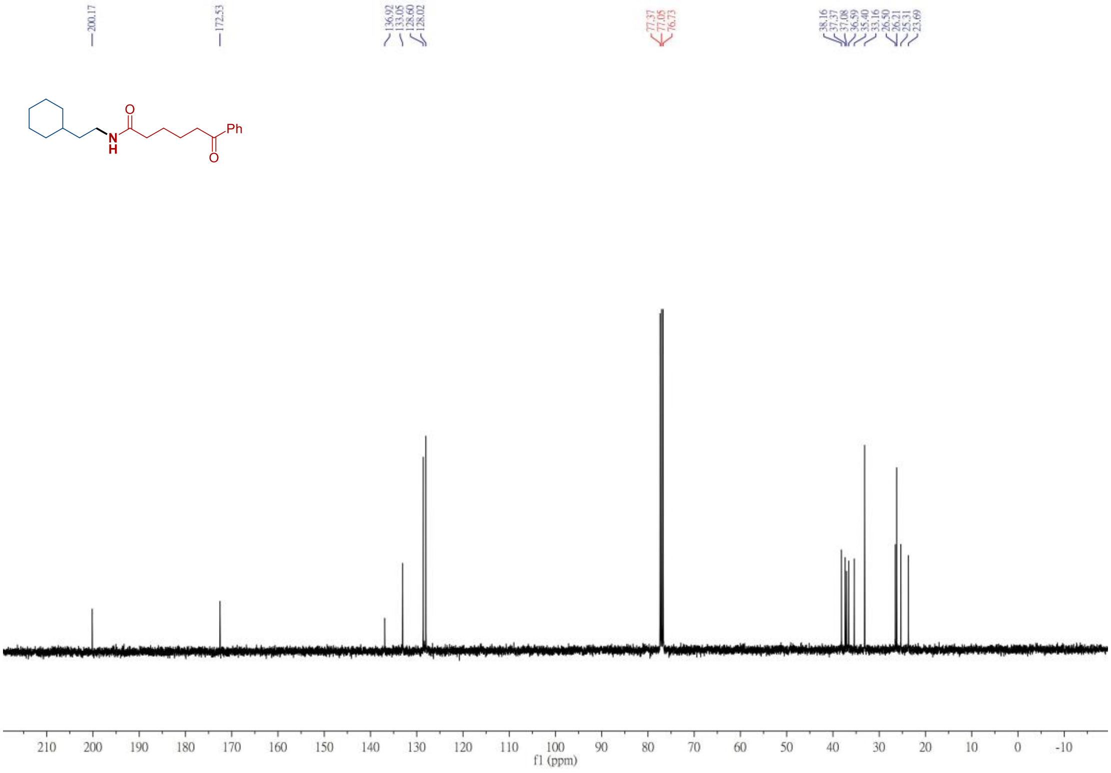

^{1}H and ^{13}C NMR spectrum of **16**

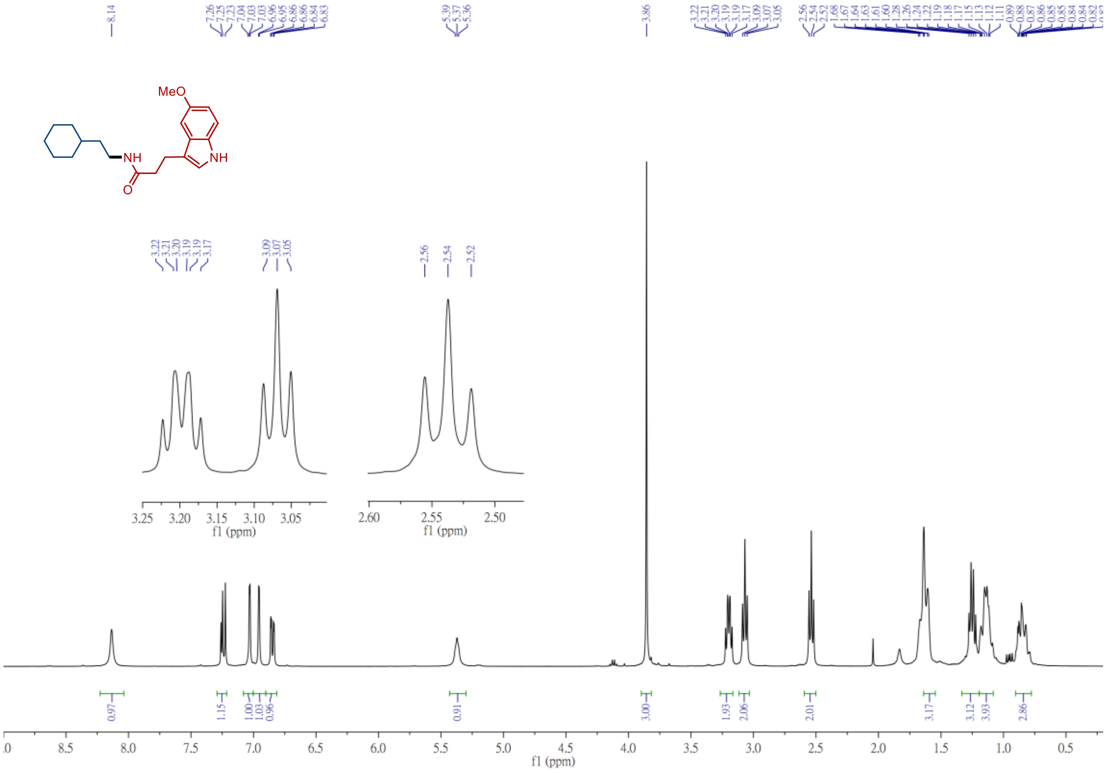

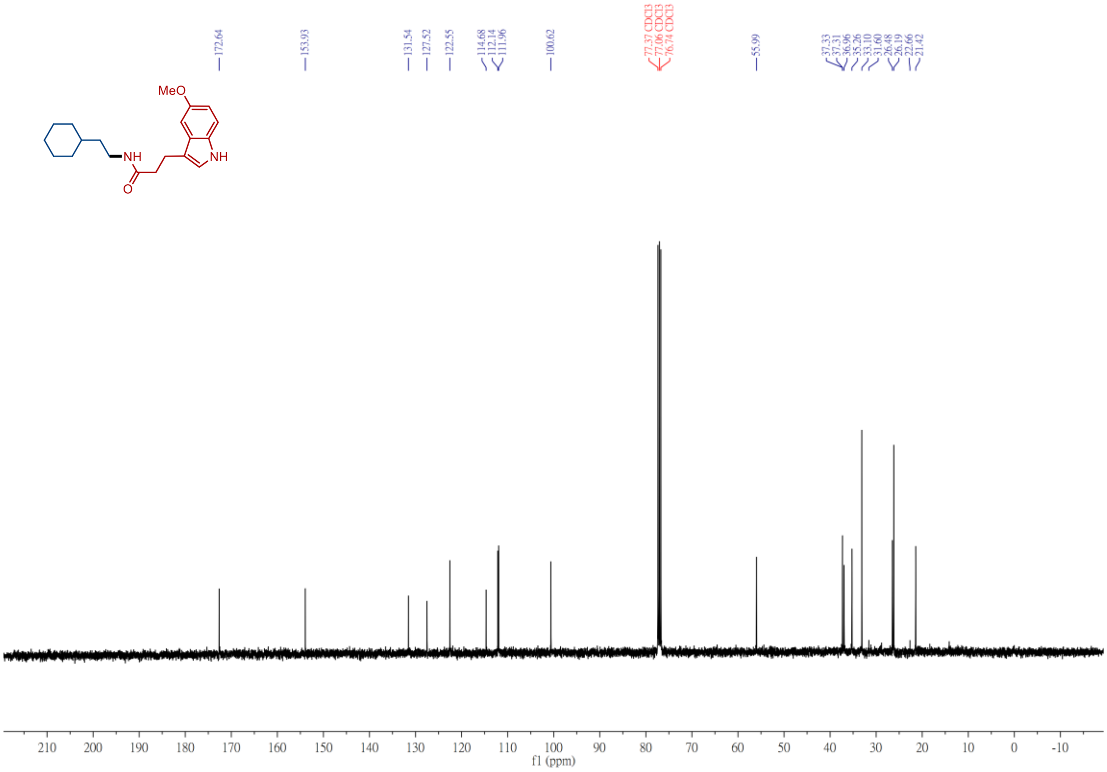

^{1}H and ^{13}C NMR spectrum of **17**

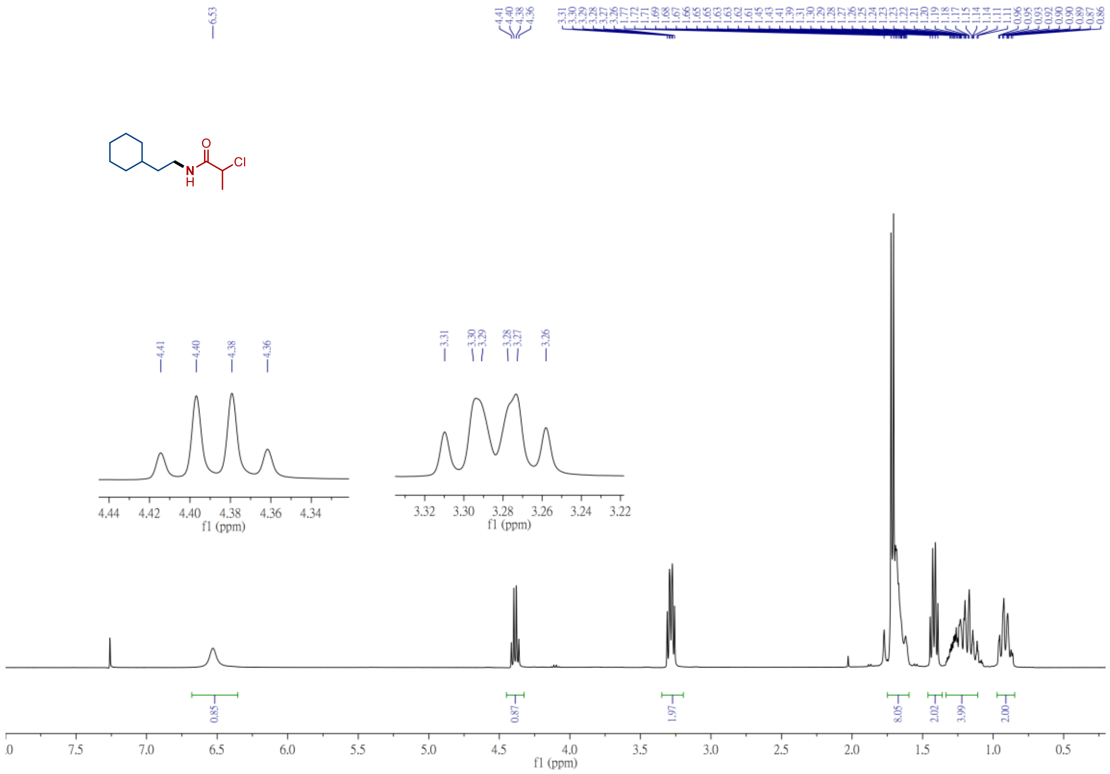

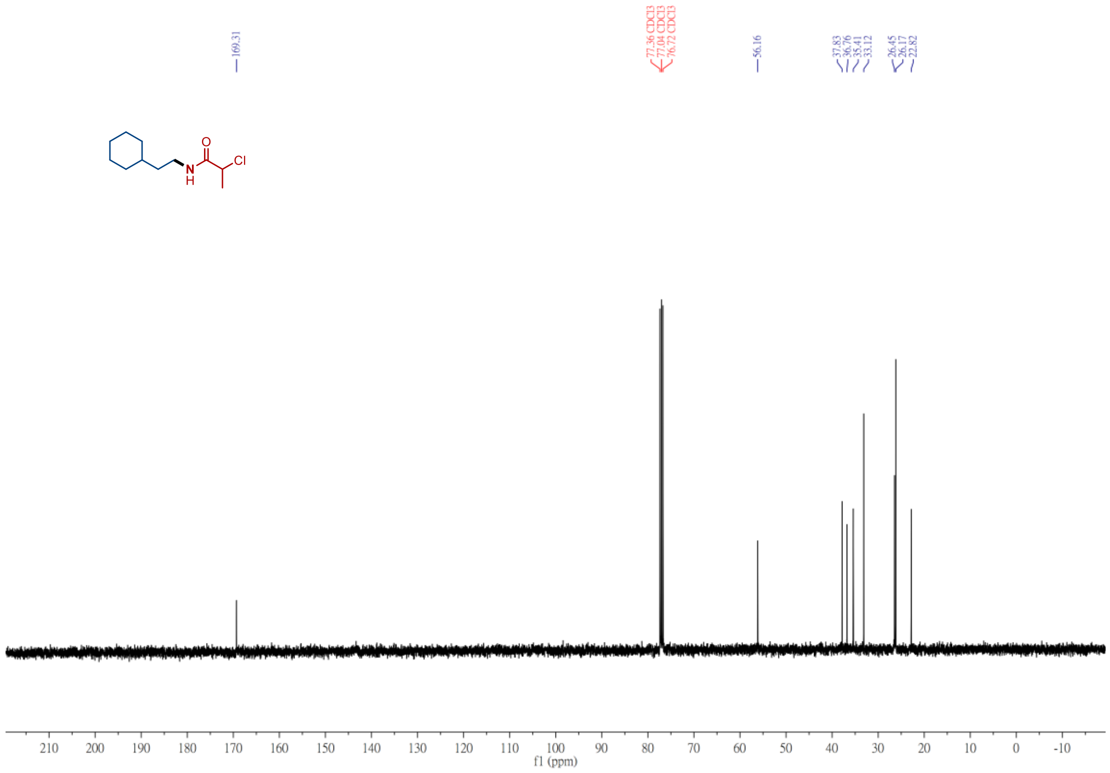

^{1}H and ^{13}C NMR spectrum of **18**

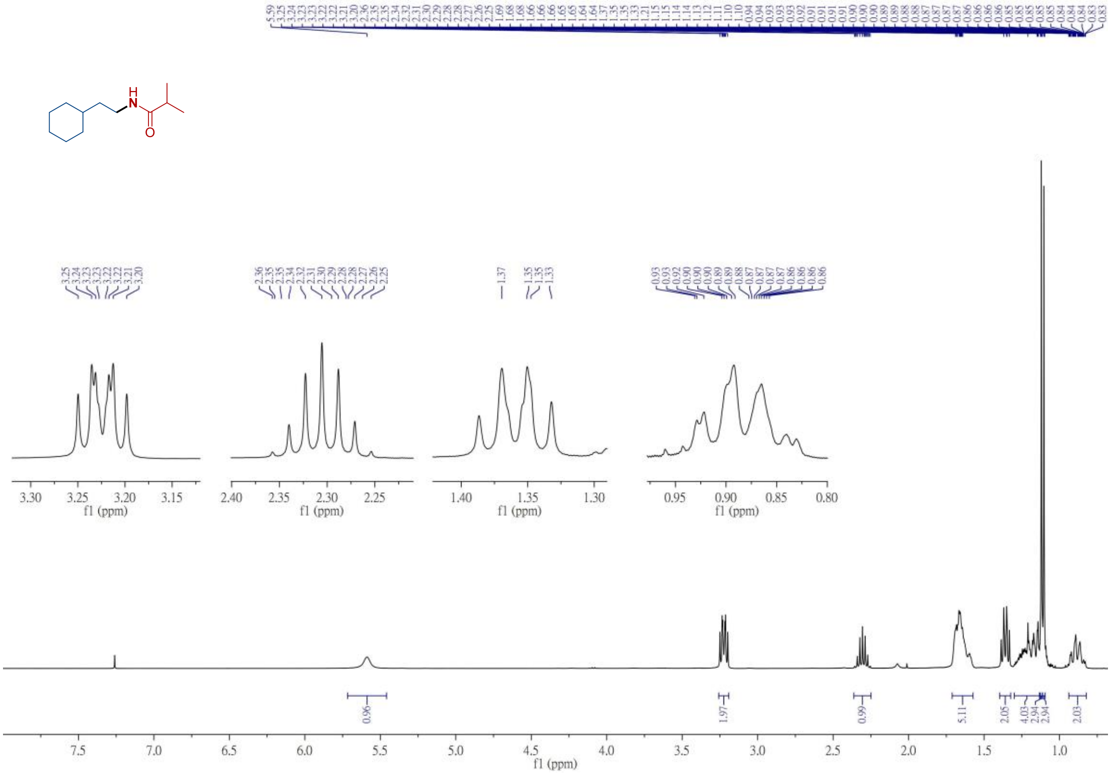

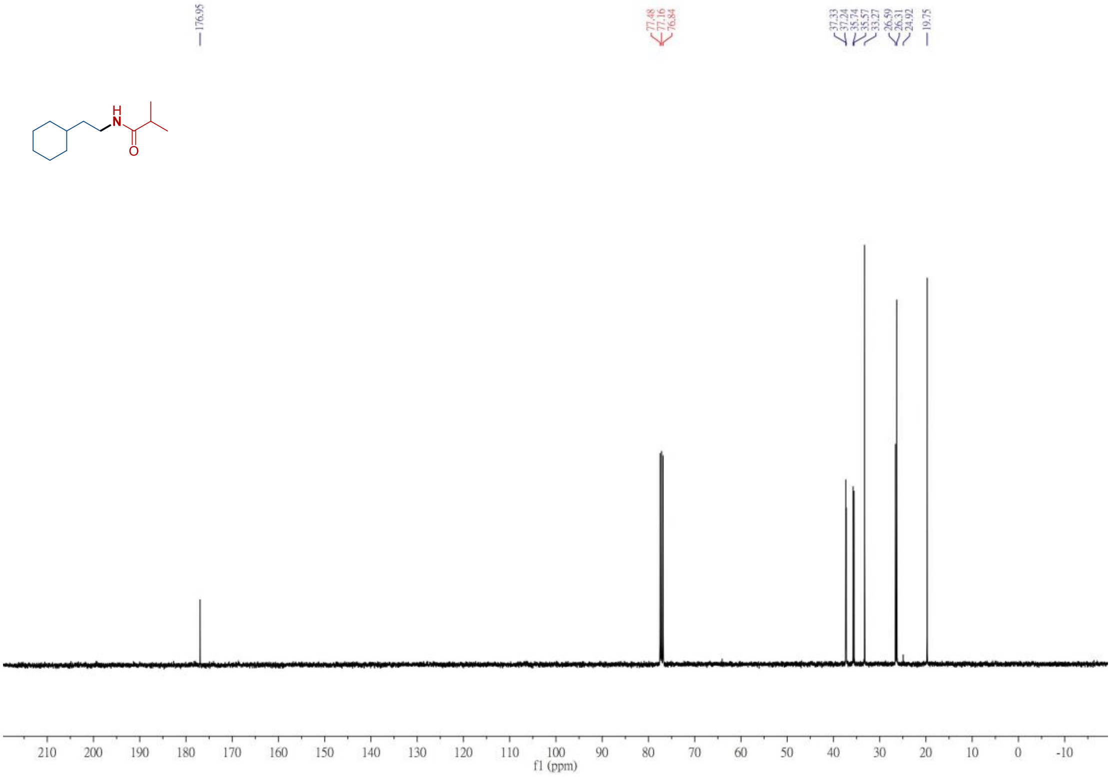

^1H and ^{13}C NMR spectrum of **19**

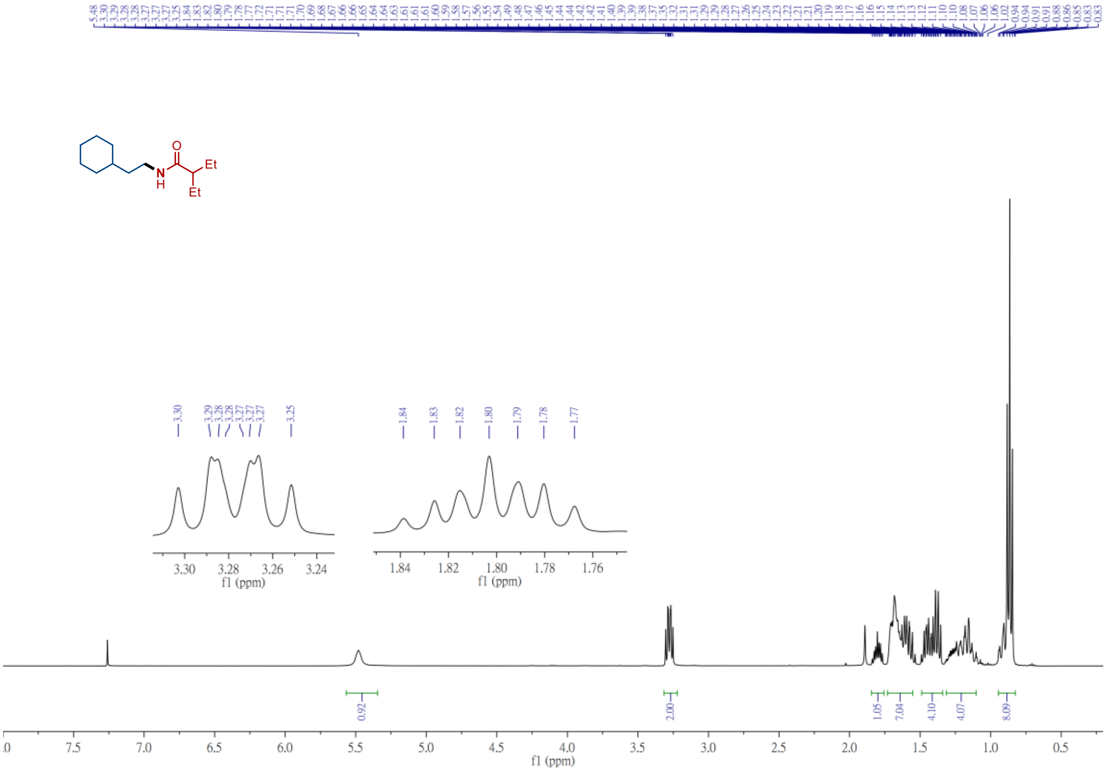

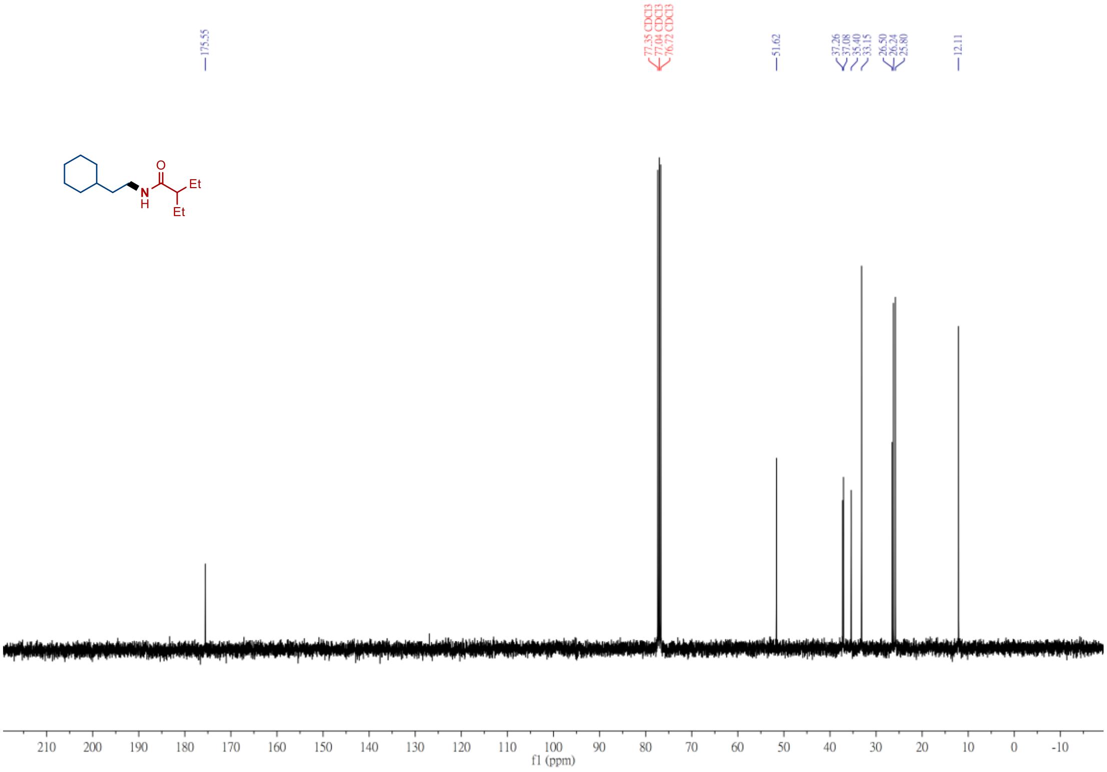

^{1}H and ^{13}C NMR spectrum of **20**

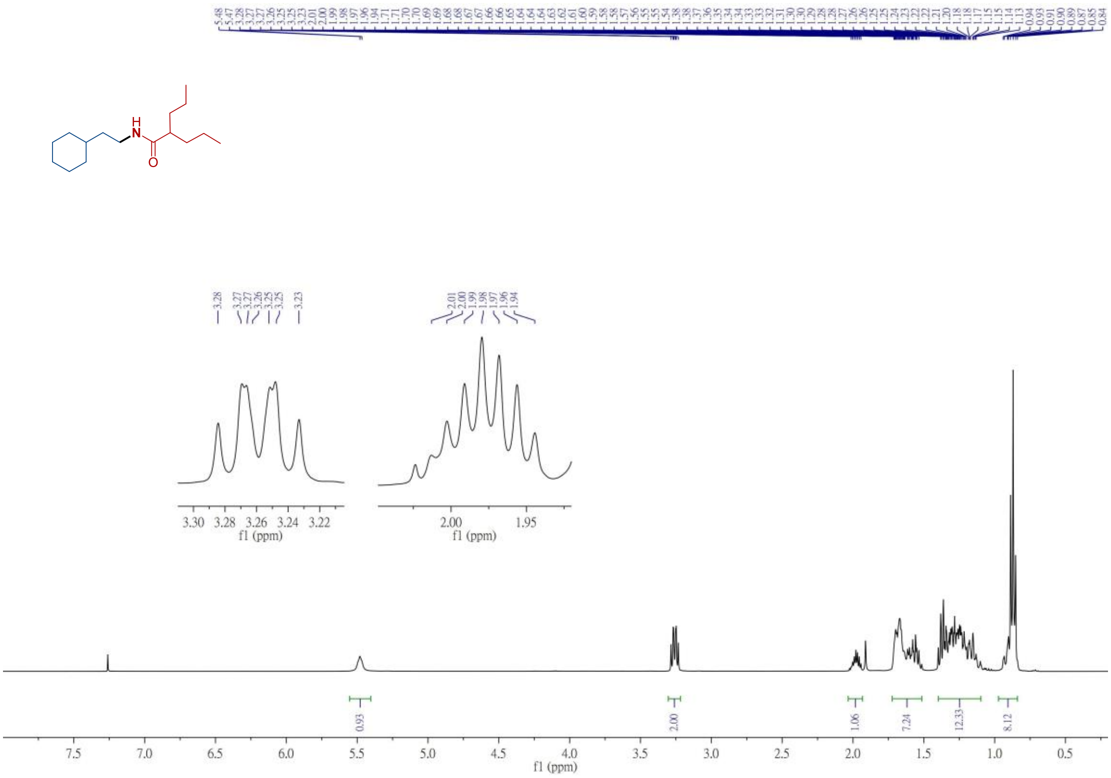

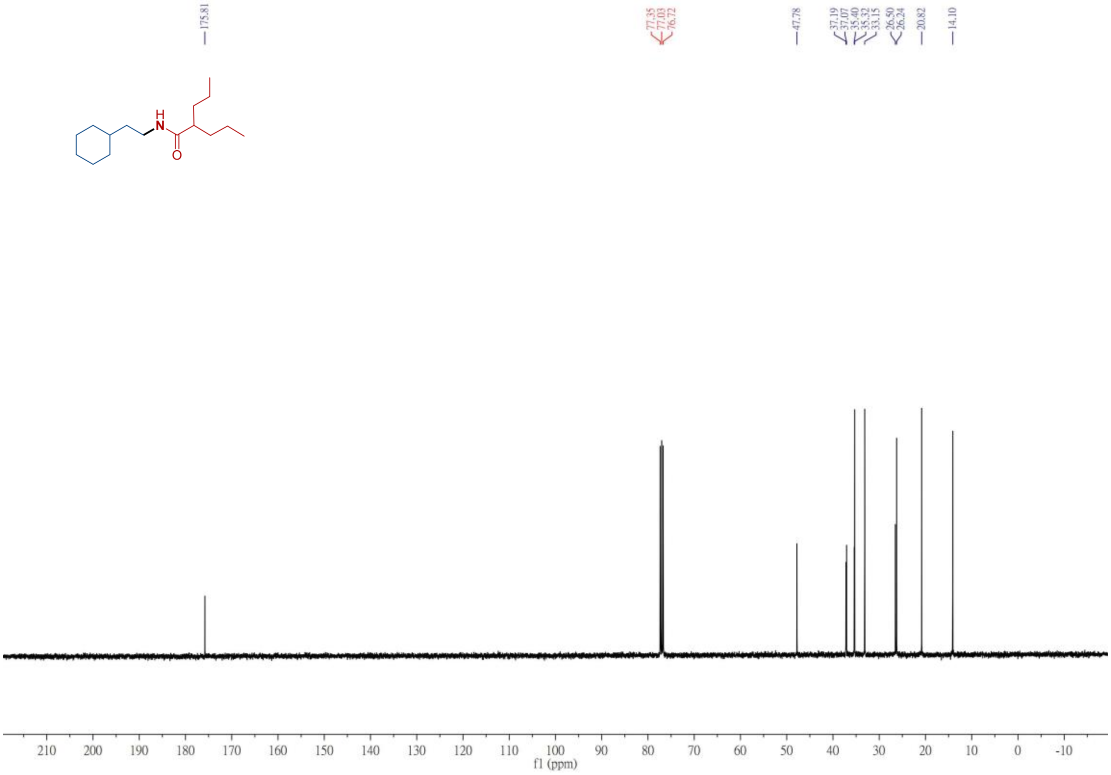

^{1}H and ^{13}C NMR spectrum of **21**

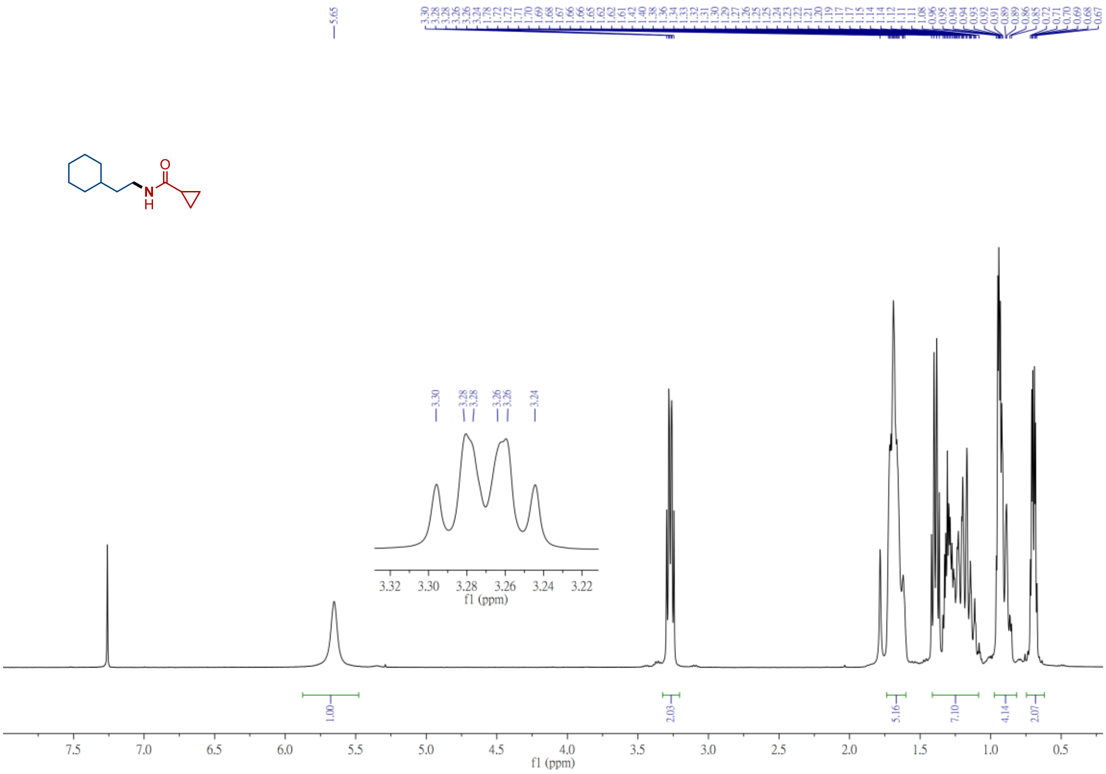

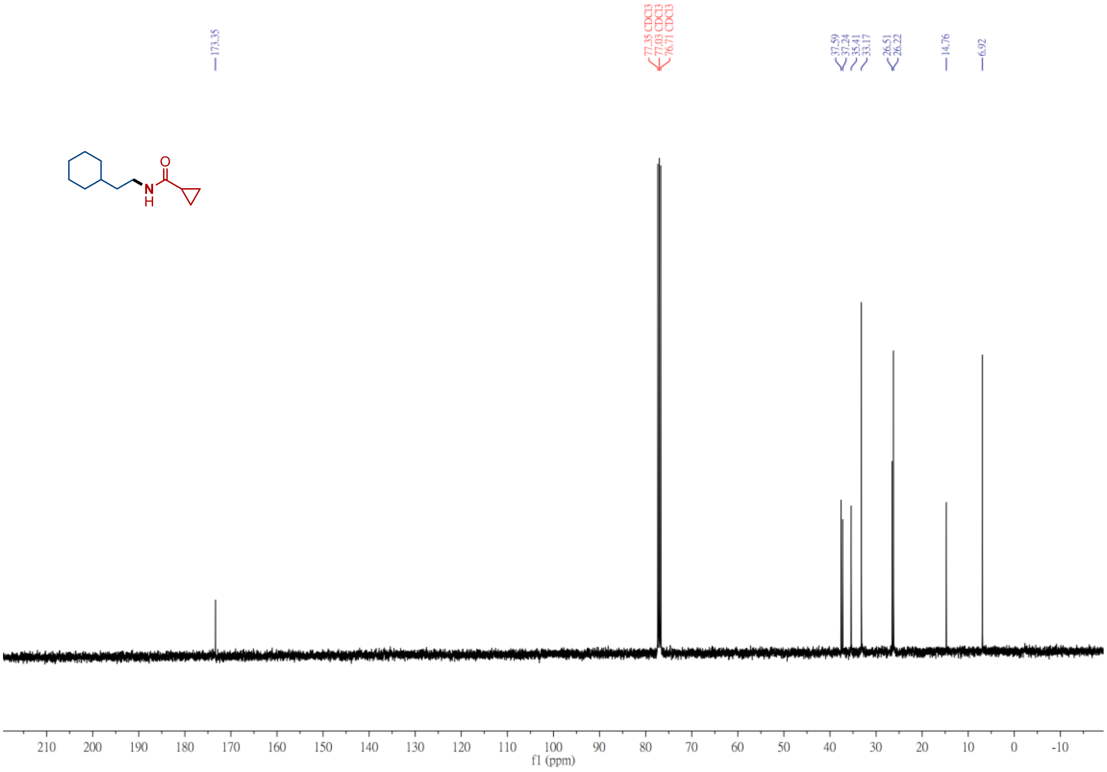

^{1}H and ^{13}C NMR spectrum of **3**

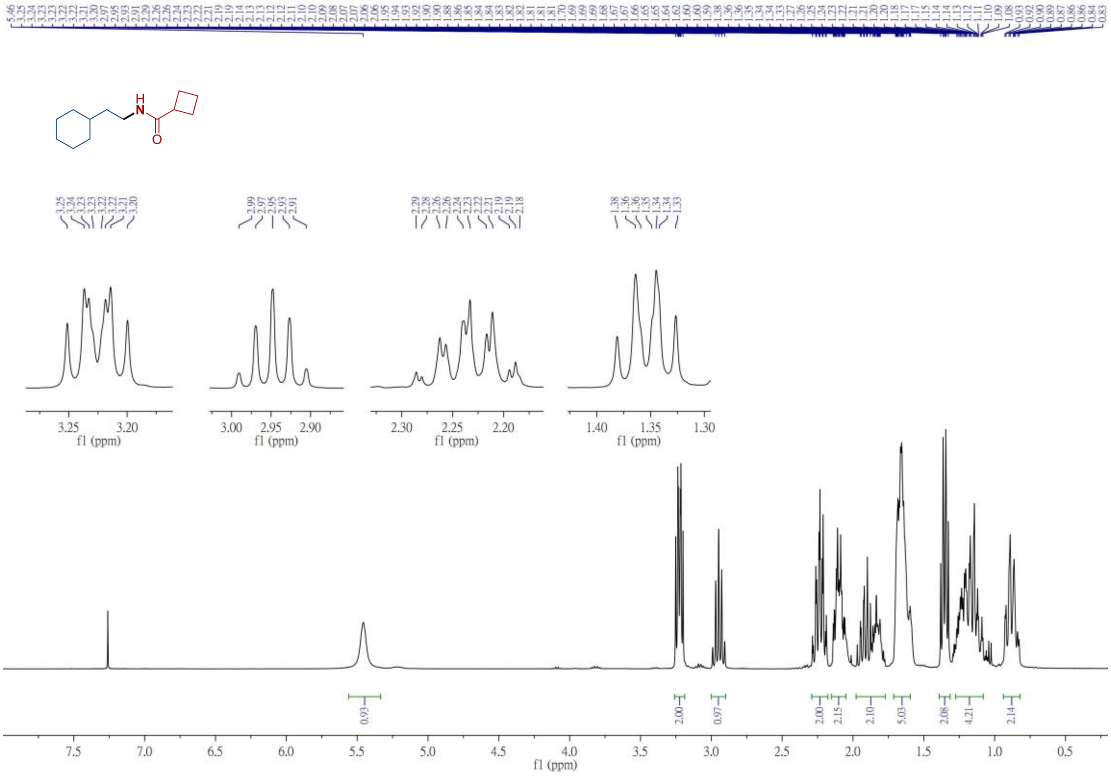

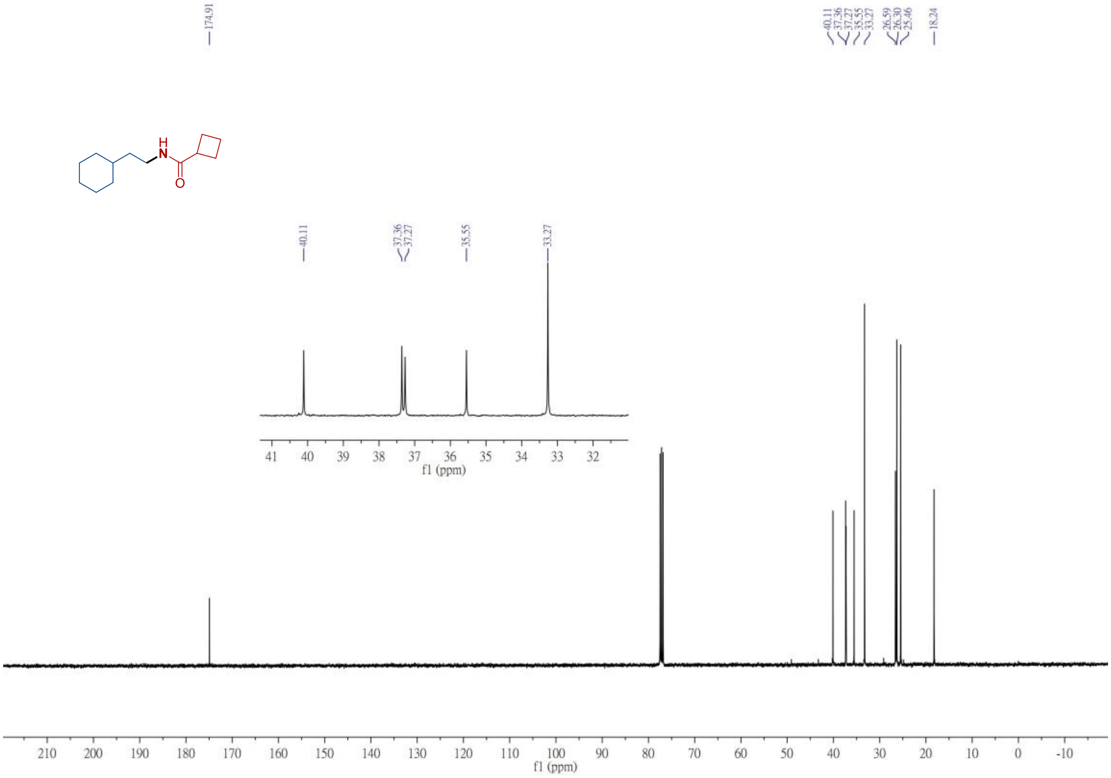

^{1}H and ^{13}C NMR spectrum of **22**

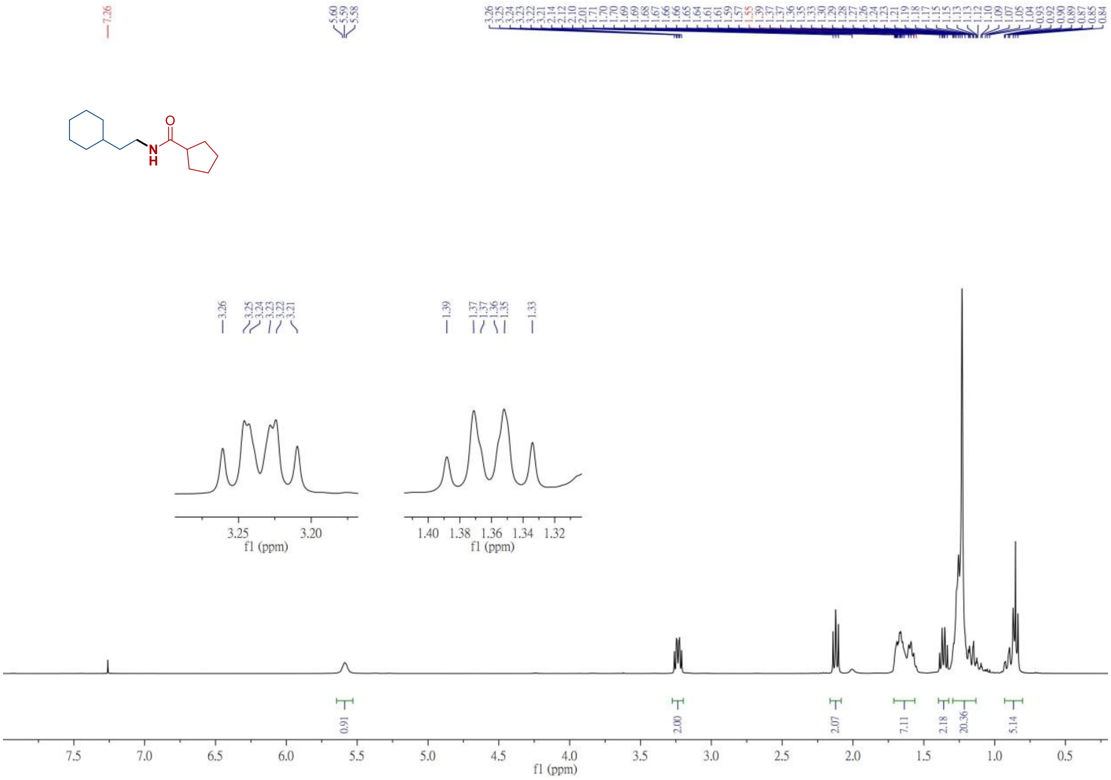

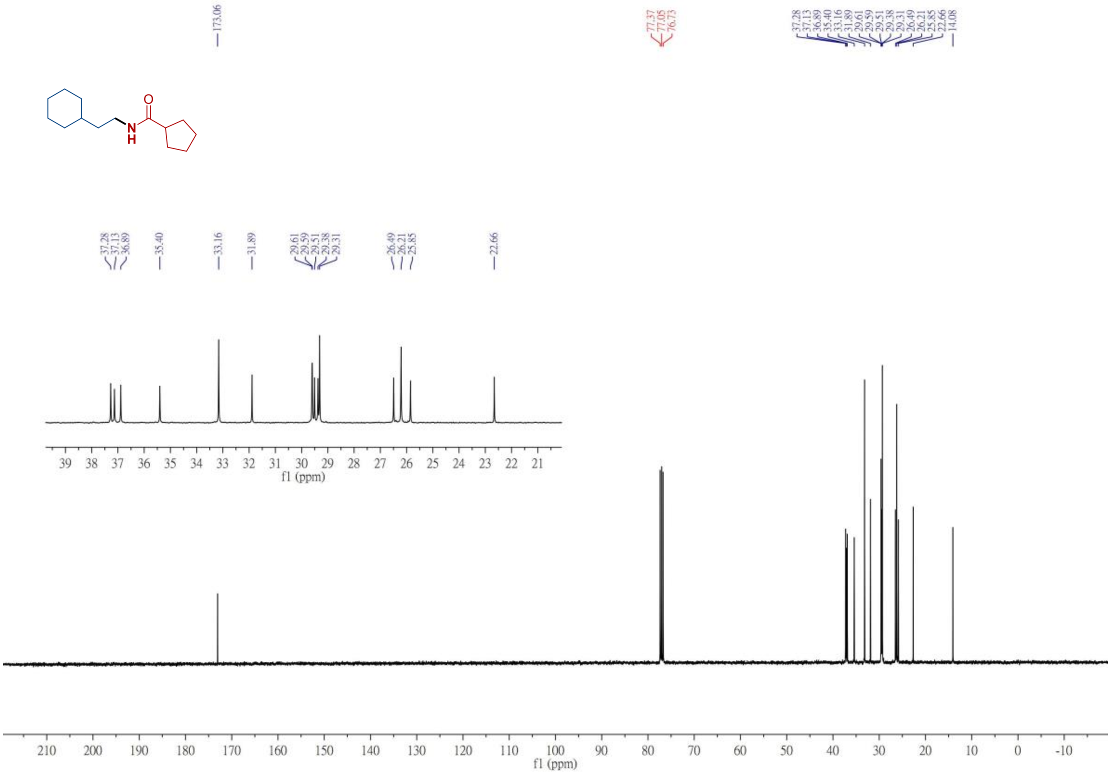

^{1}H and ^{13}C NMR spectrum of **23**

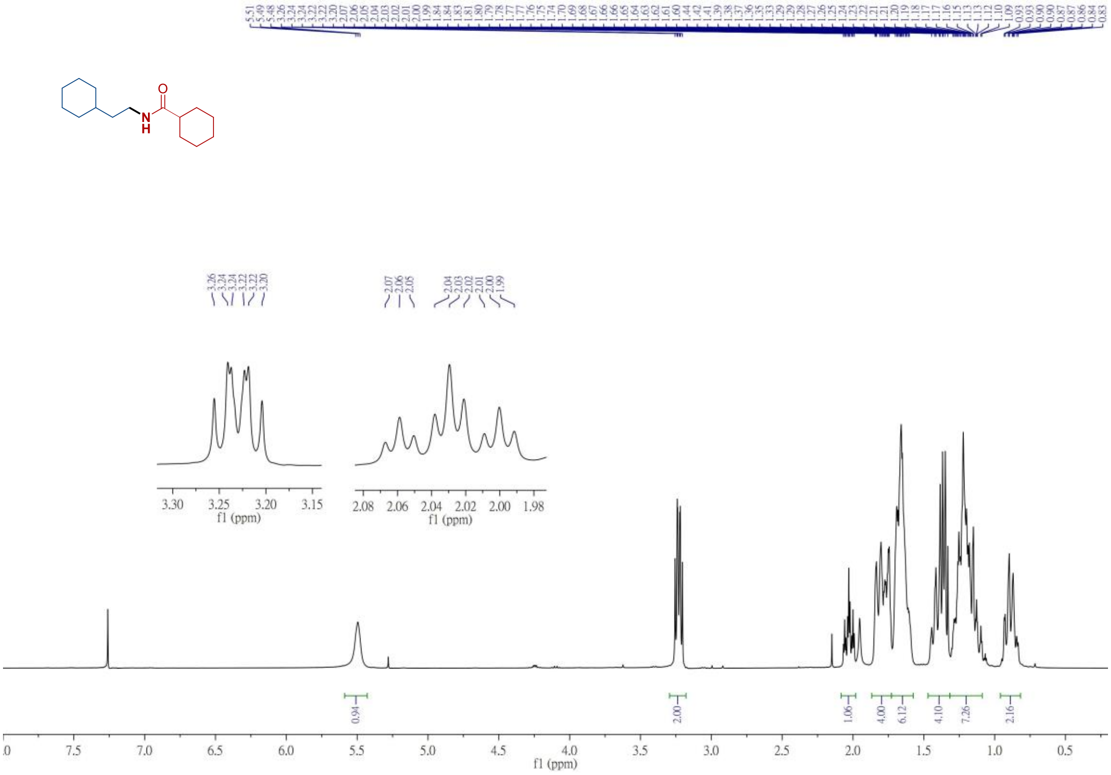

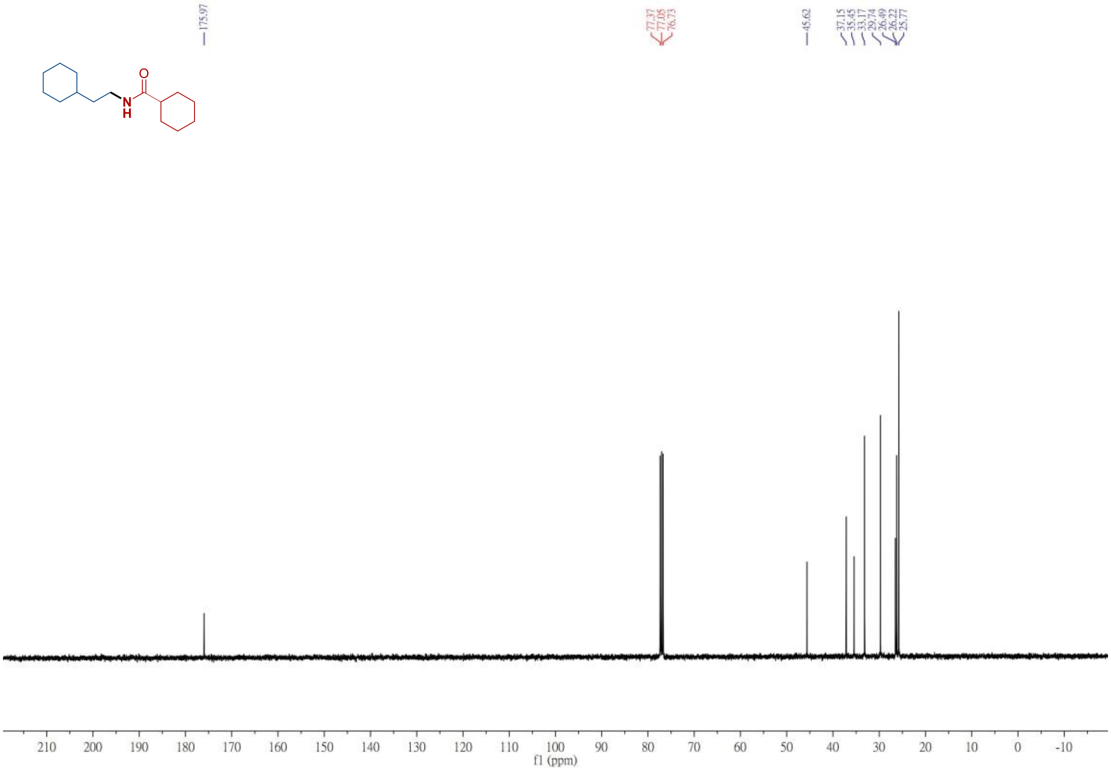

^{1}H and ^{13}C NMR spectrum of **24**

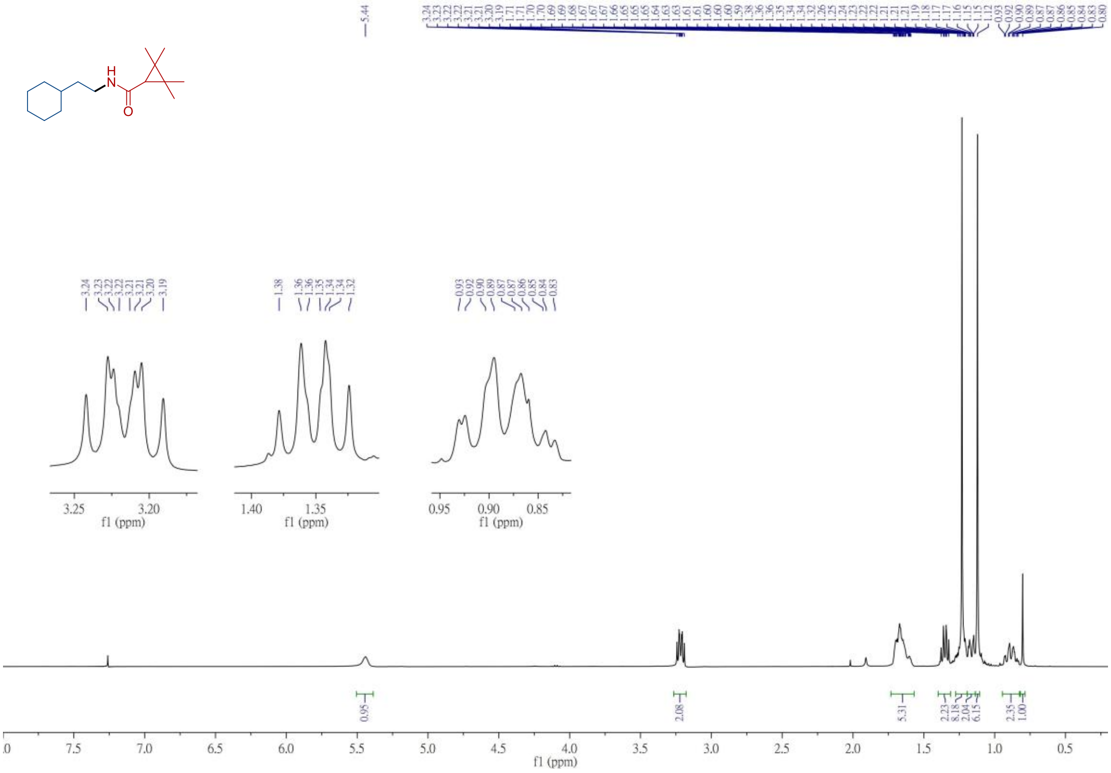

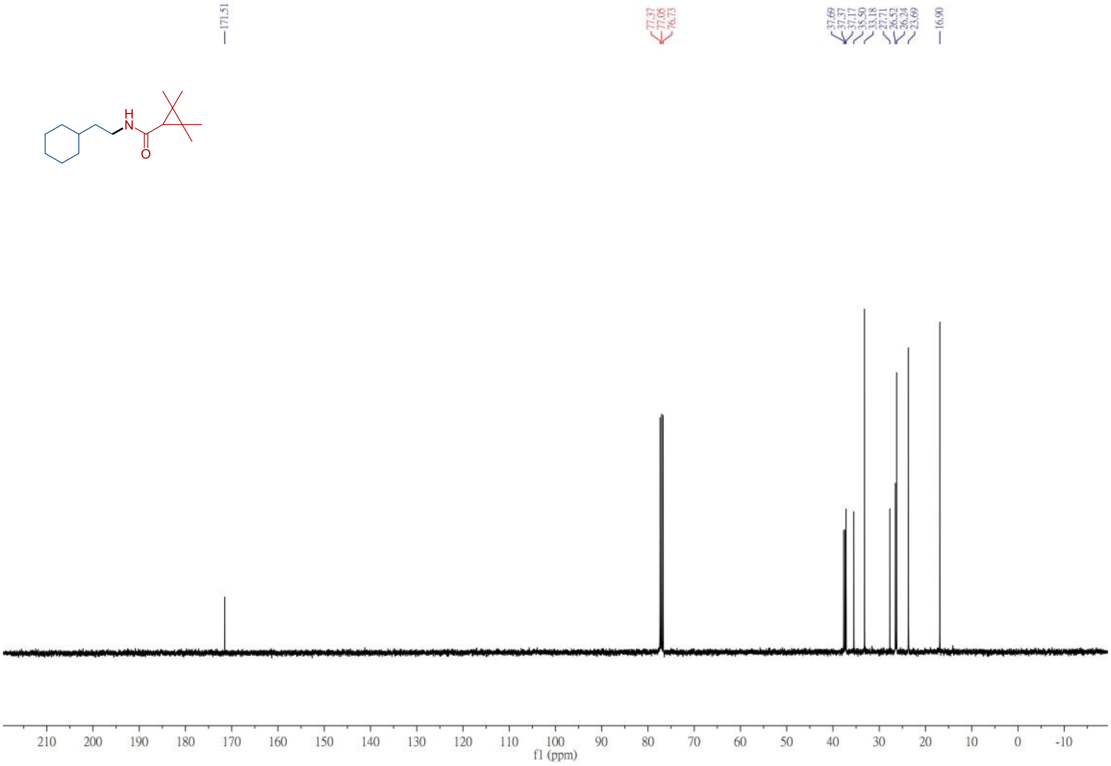

^{1}H and ^{13}C NMR spectrum of **25**

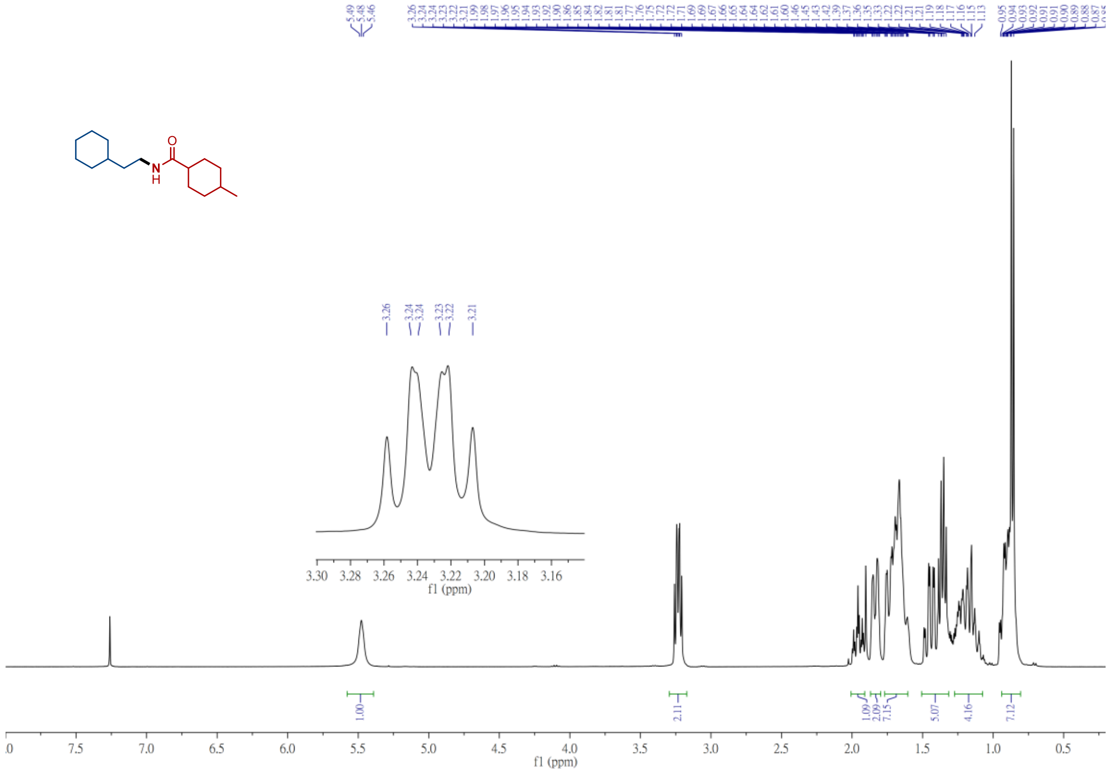
O
N
H
f1 (ppm)
1.00
2.11
1.09
2.09
7.15
5.07
4.16
7.12

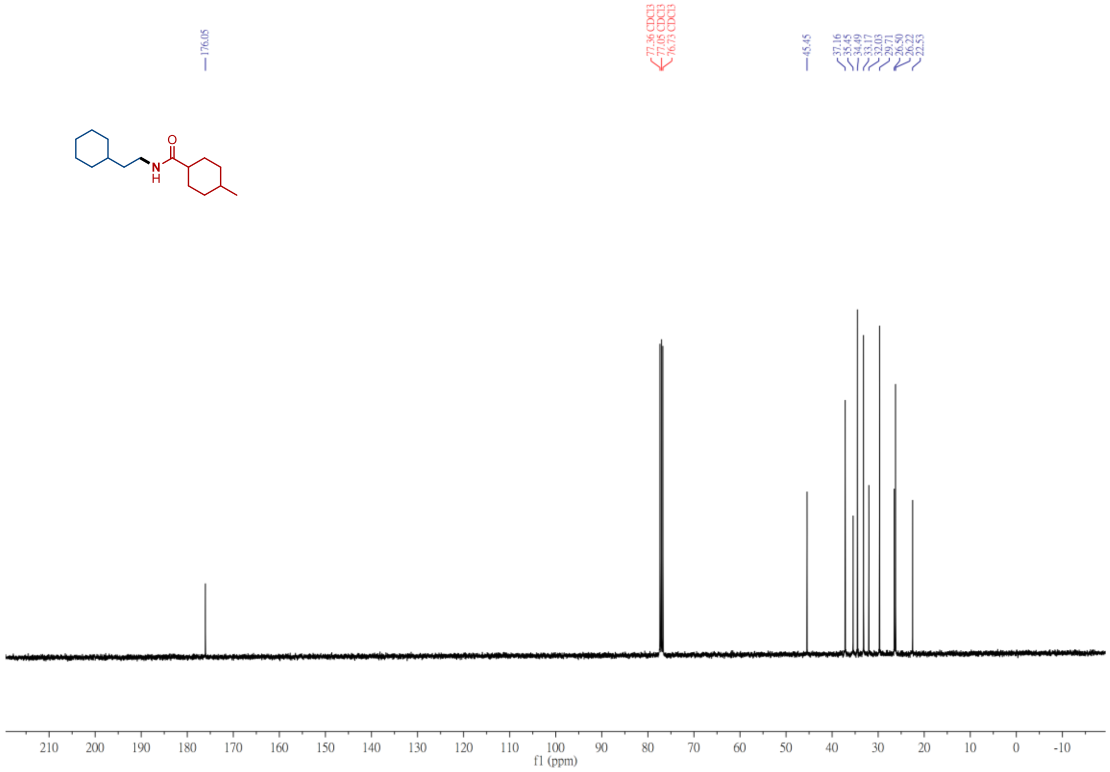
O
N
H
176.05
77.36 CDCl3
77.05 CDCl3
76.73 CDCl3
45.45
37.16
35.45
34.49
33.17
32.03
29.71
26.50
26.22
22.53
f1 (ppm)

^{1}H and ^{13}C NMR spectrum of **26**

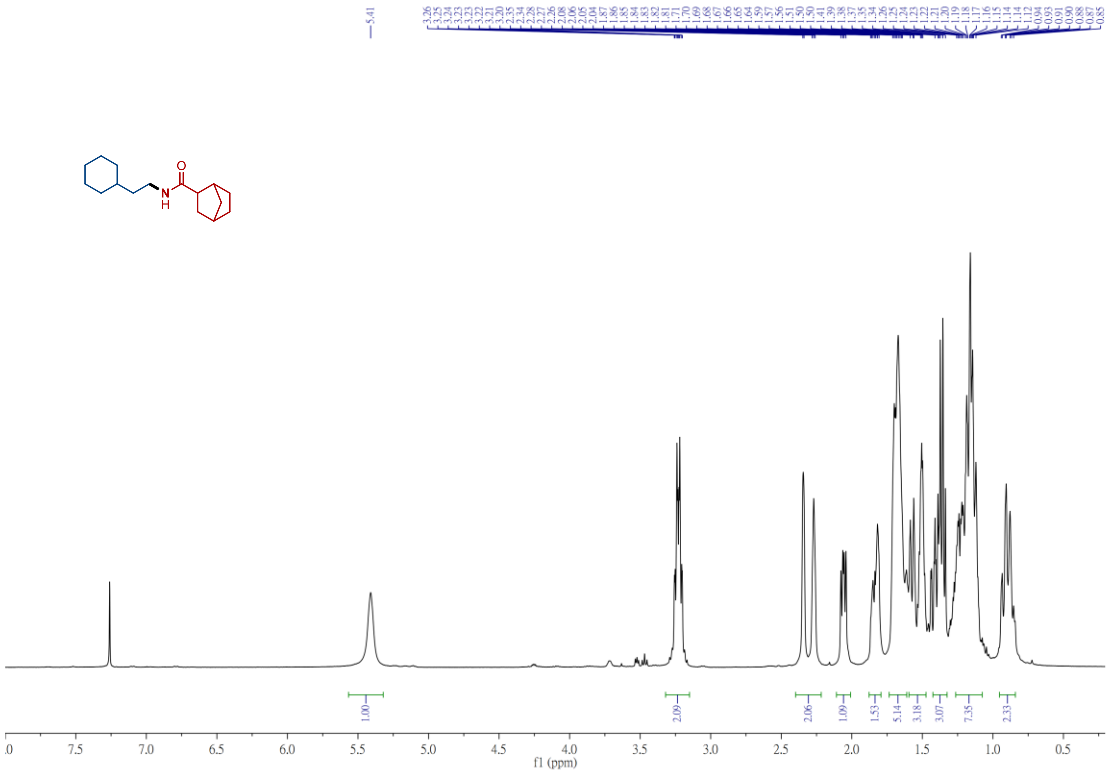

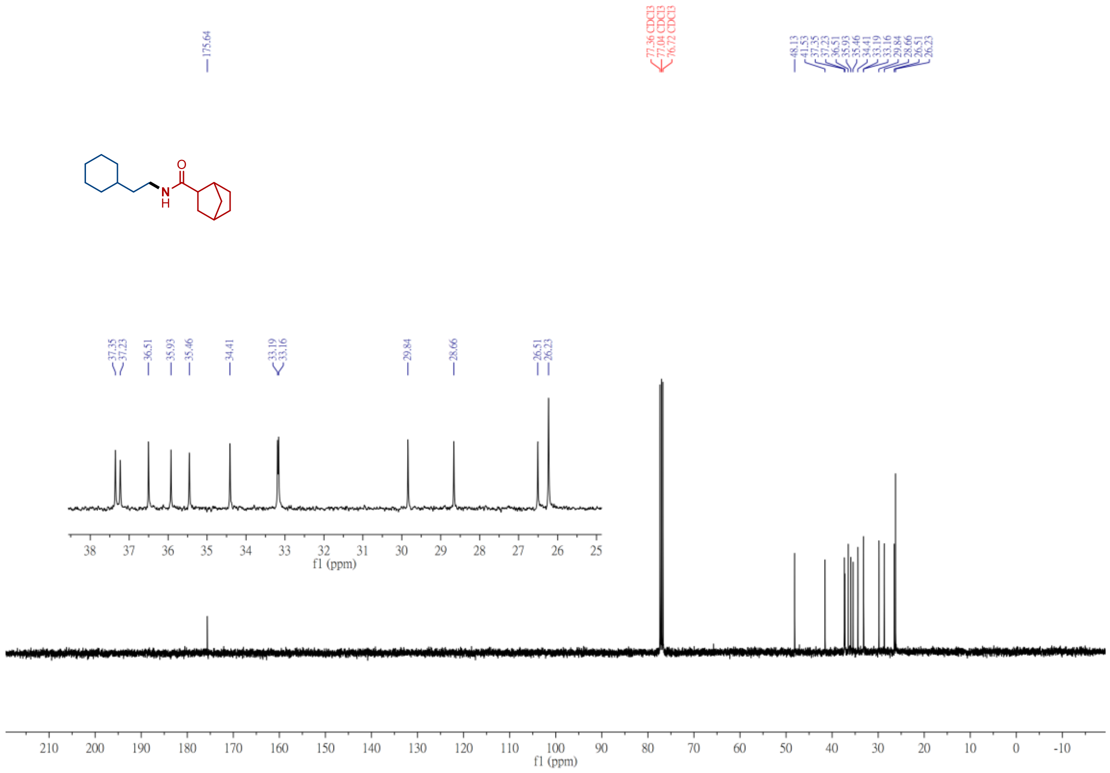

^{1}H and ^{13}C NMR spectrum of **27**

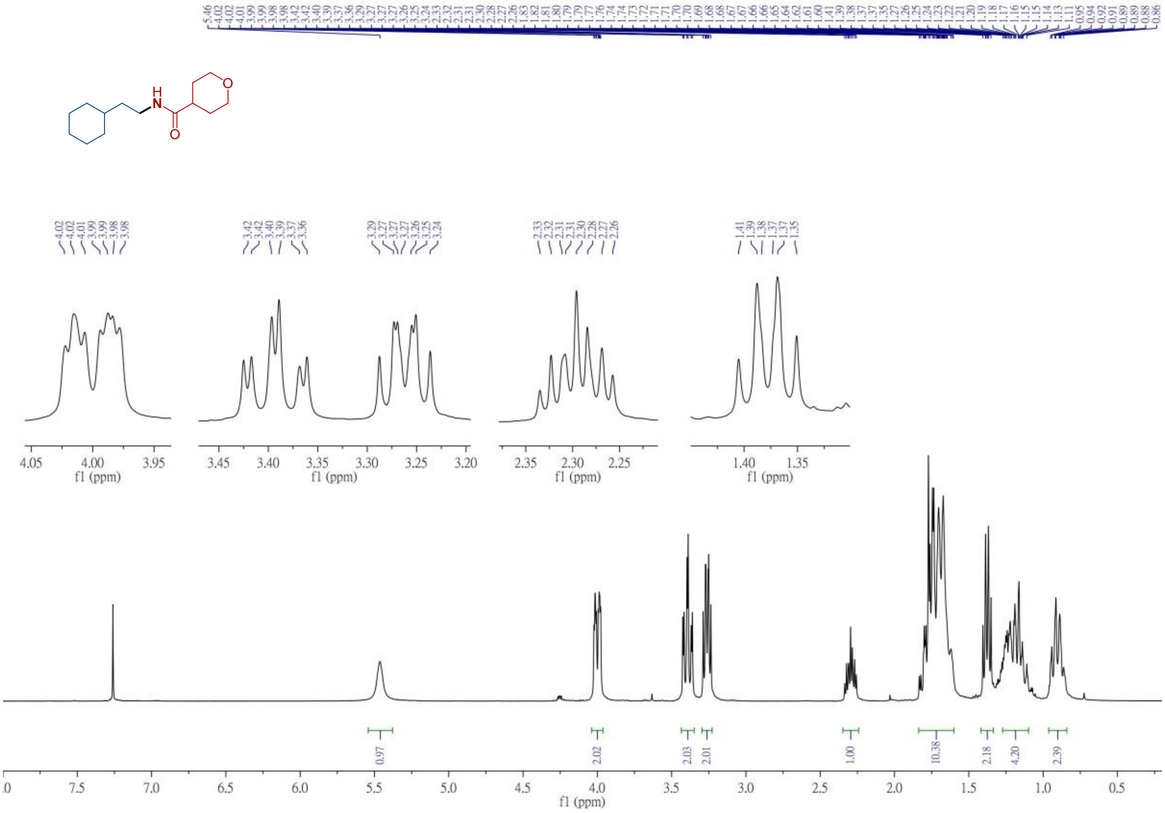

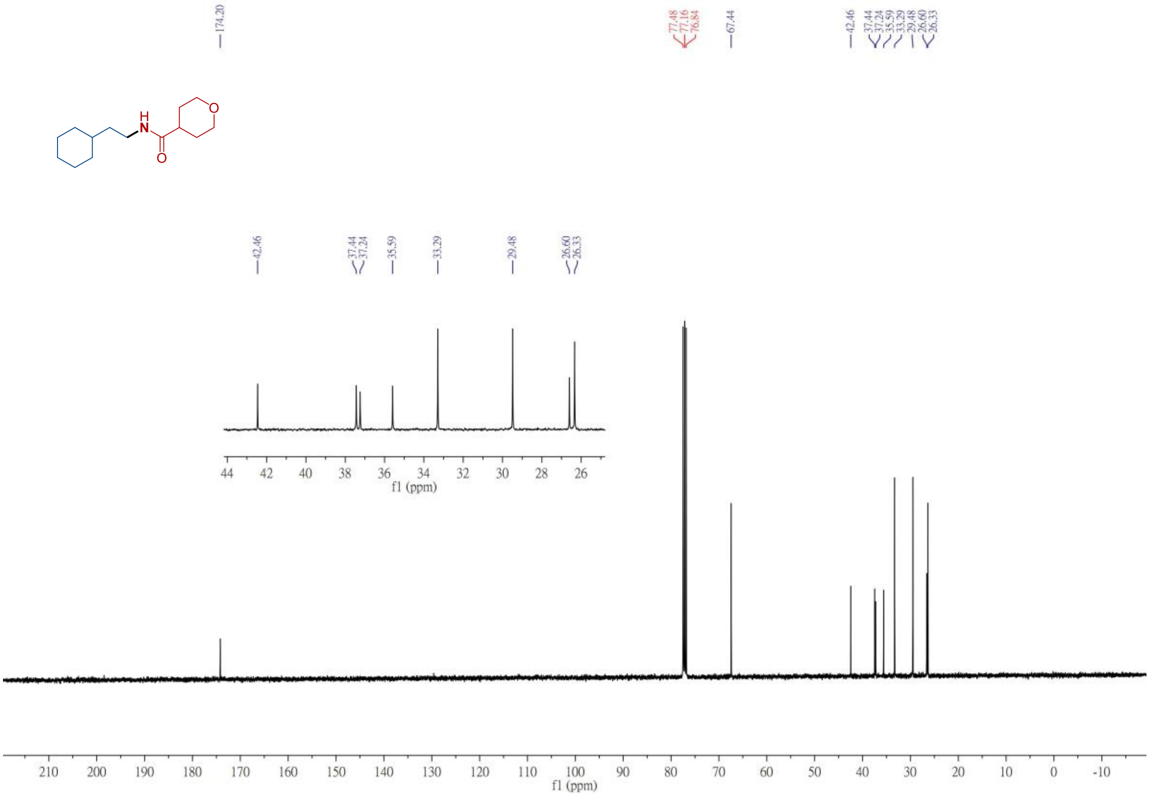

^{1}H and ^{13}C NMR spectrum of **28**

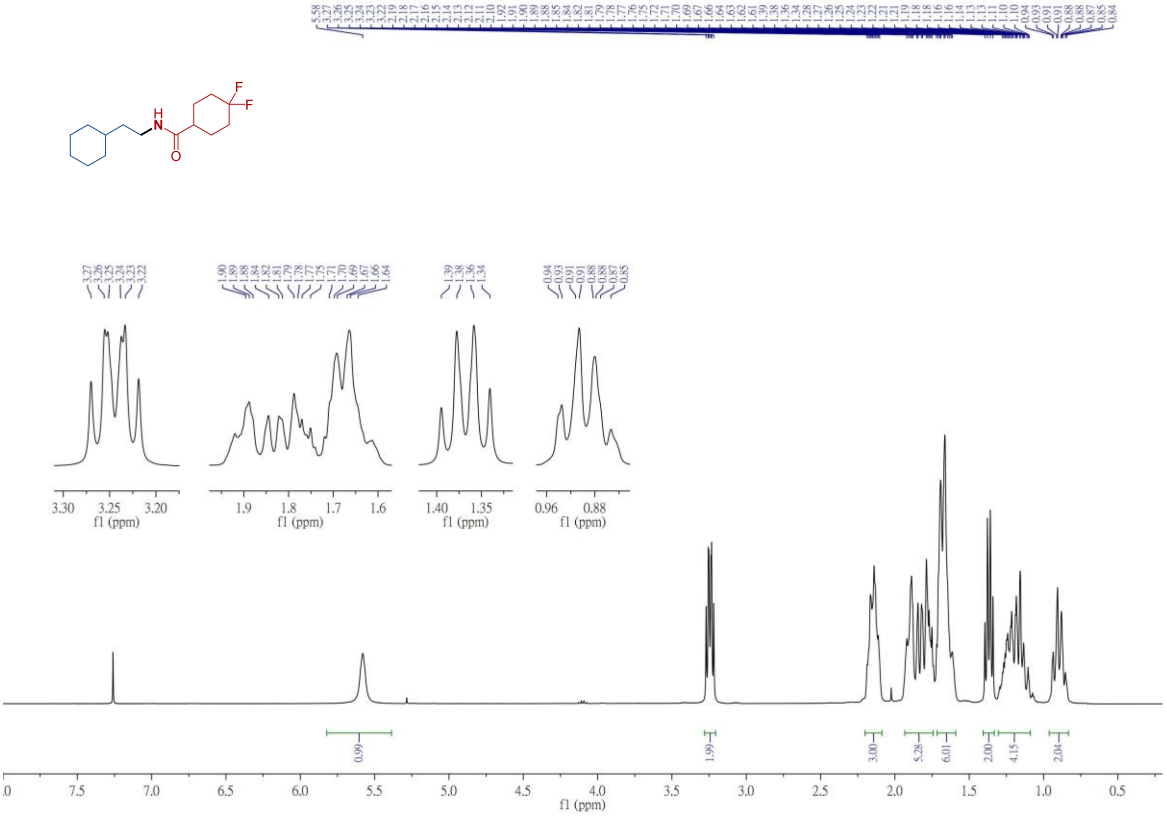

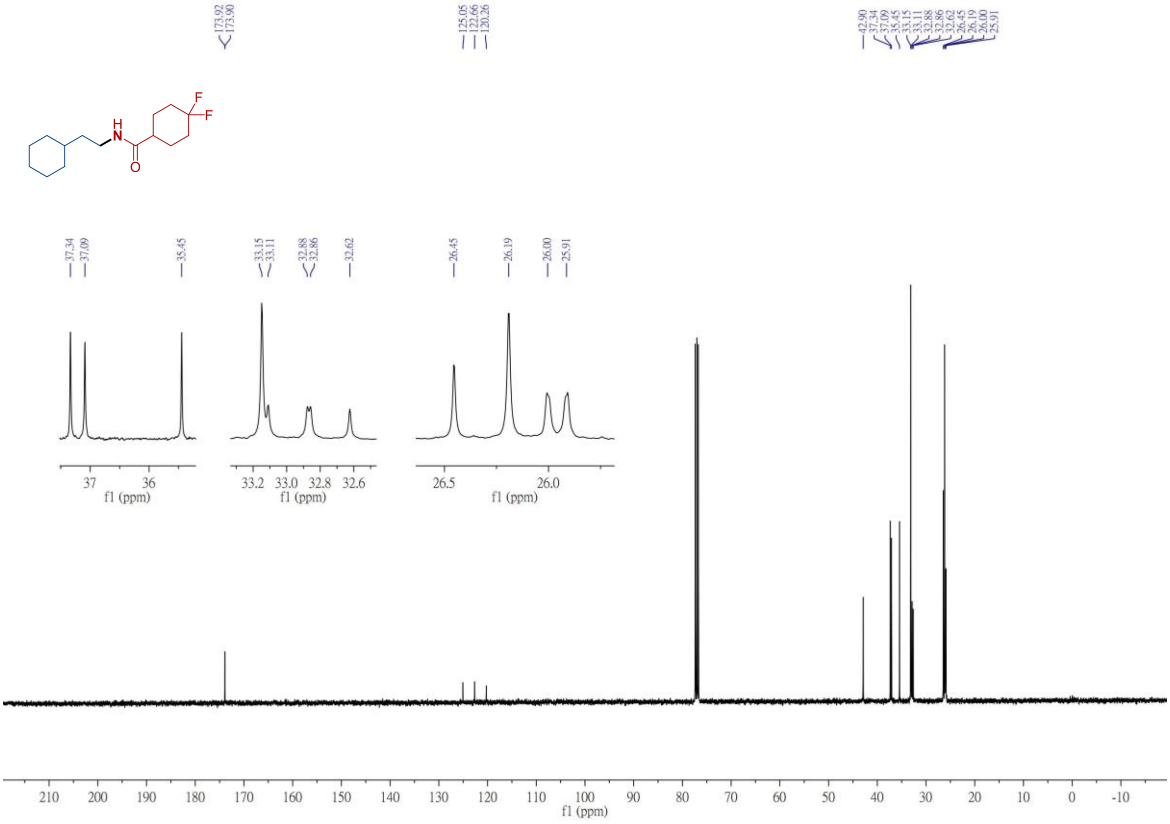

^{19}F NMR spectrum of **28**

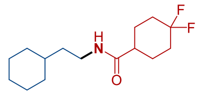

F
F
H
N
O

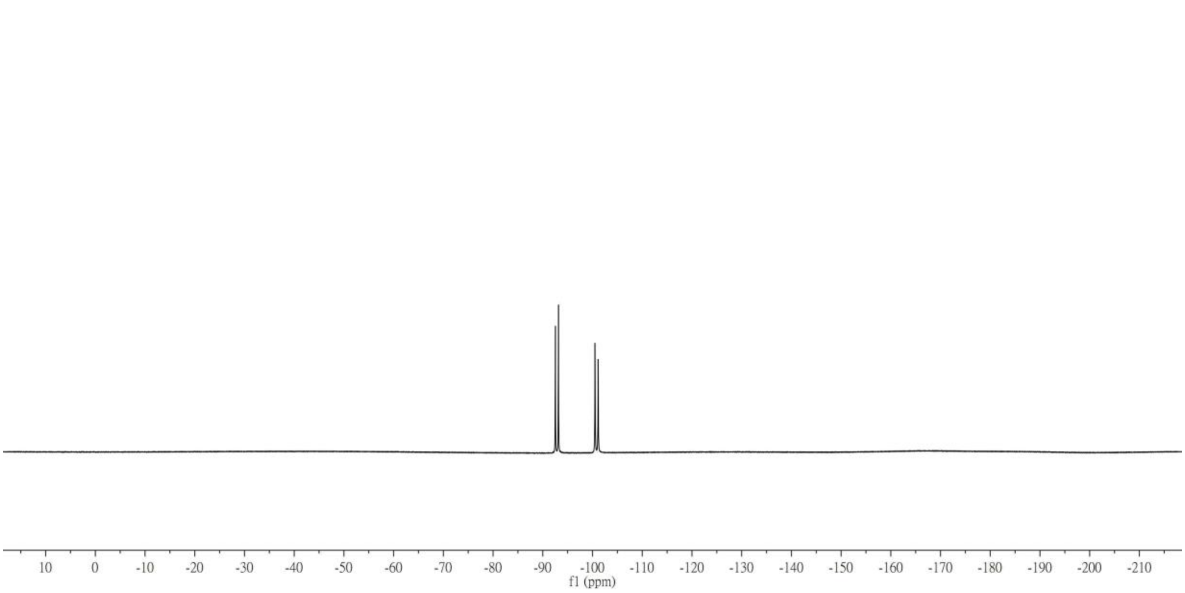

-92.52
-93.15
-100.51
-101.13
10
0
-10
-20
-30
-40
-50
-60
-70
-80
-90
-100
-110
-120
-130
-140
-150
-160
-170
-180
-190
-200
-210
f1 (ppm)

^{1}H and ^{13}C NMR spectrum of **29**

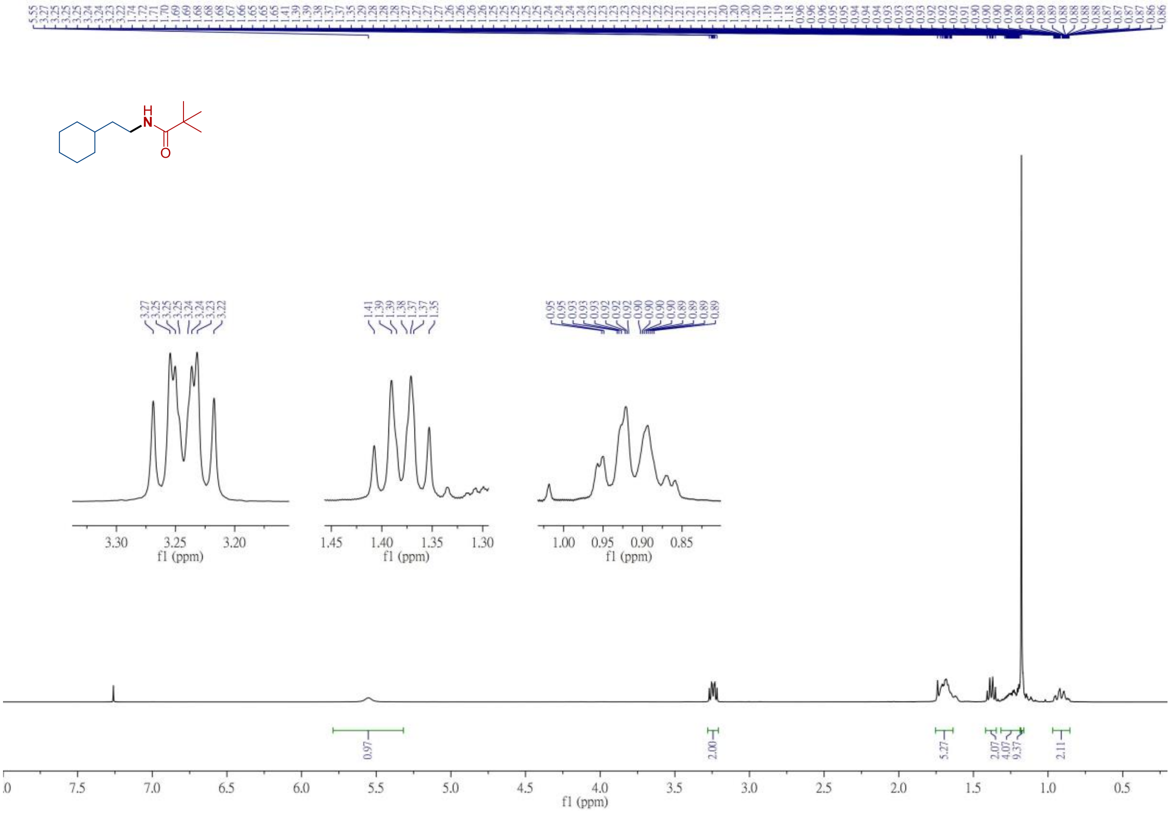

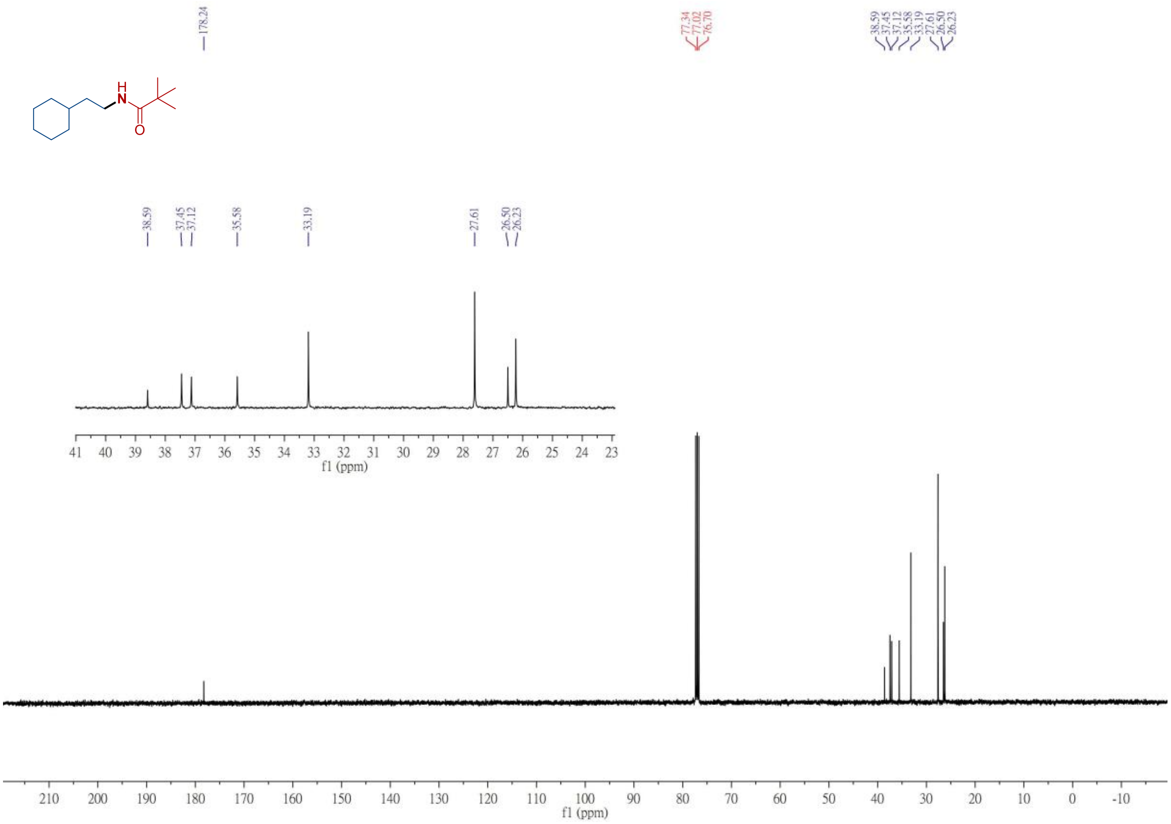

^{1}H and ^{13}C NMR spectrum of **30**

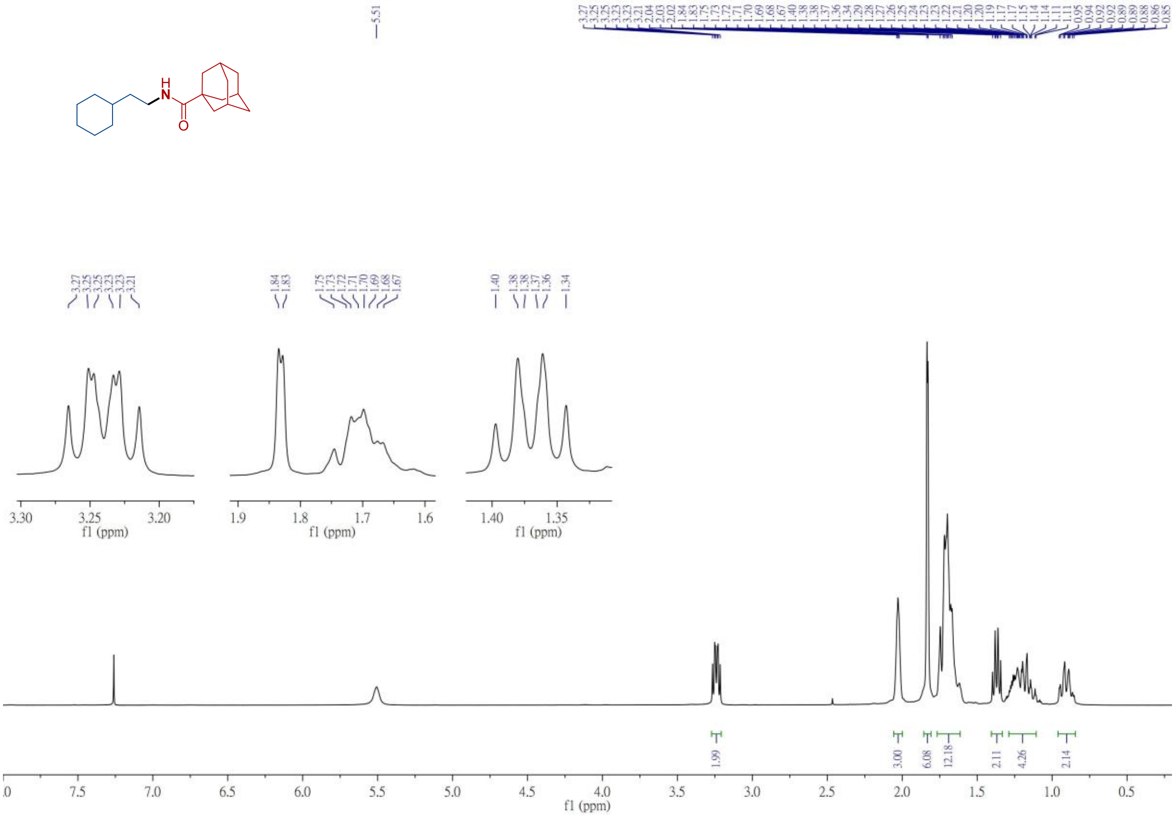

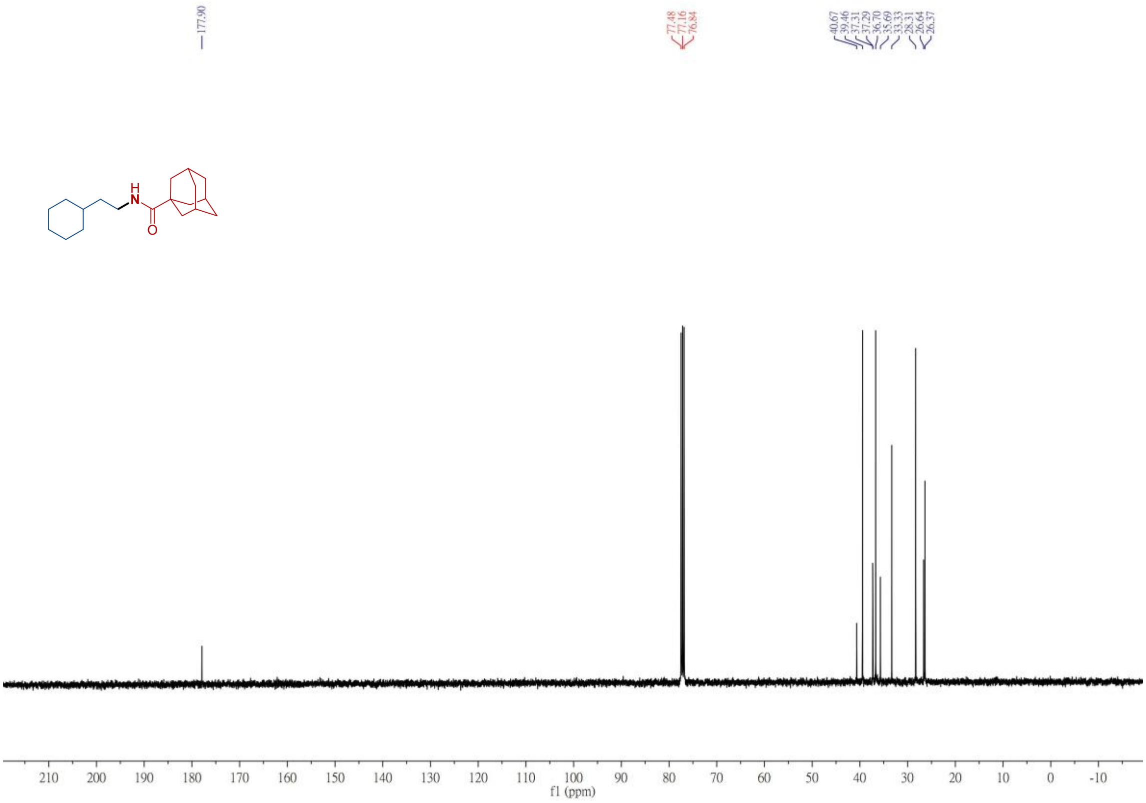

^{1}H and ^{13}C NMR spectrum of **31**

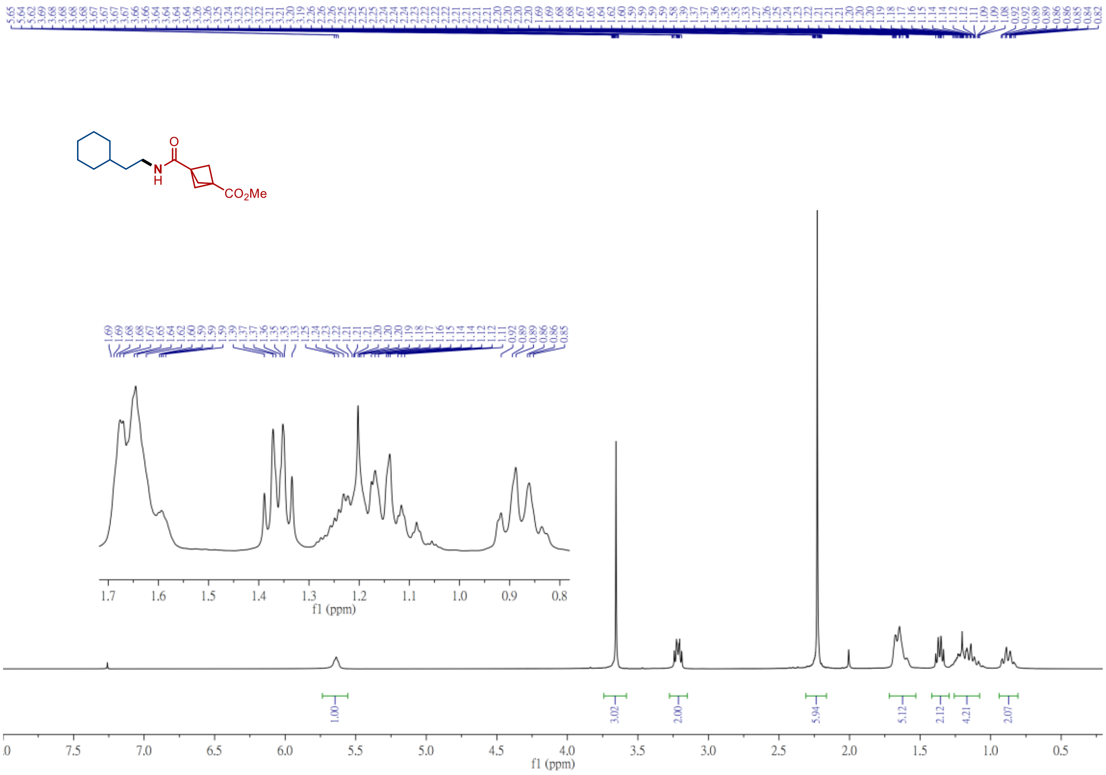

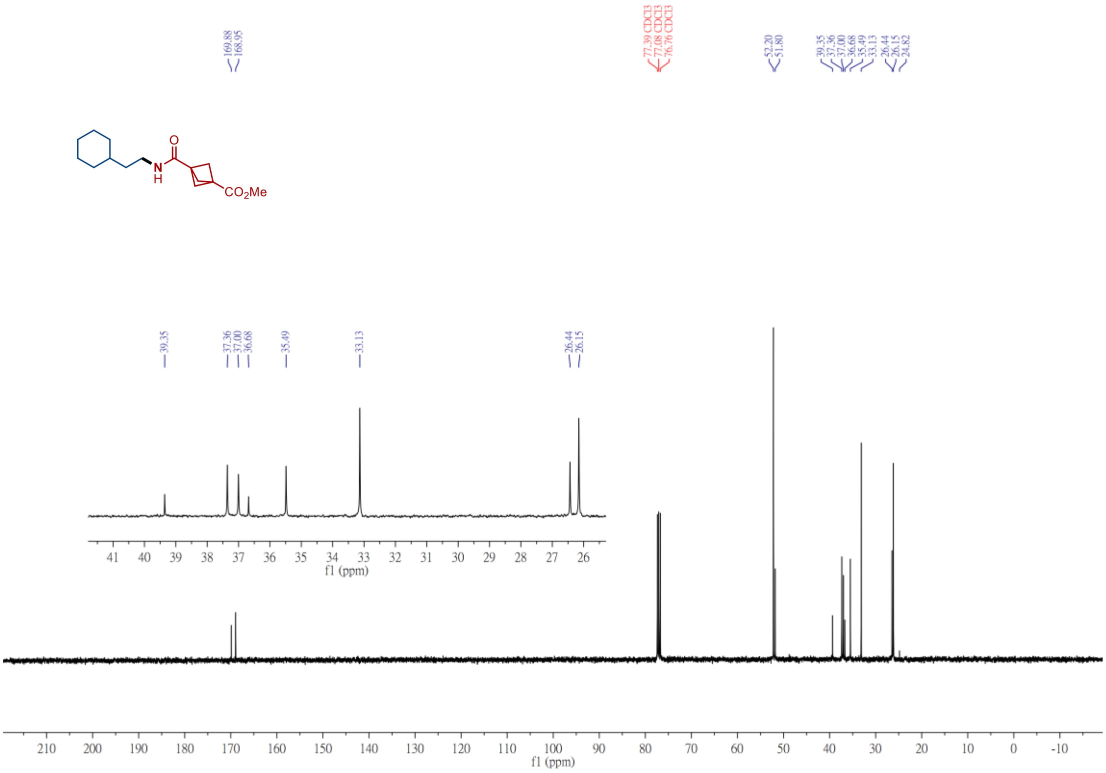

^{1}H and ^{13}C NMR spectrum of **32**

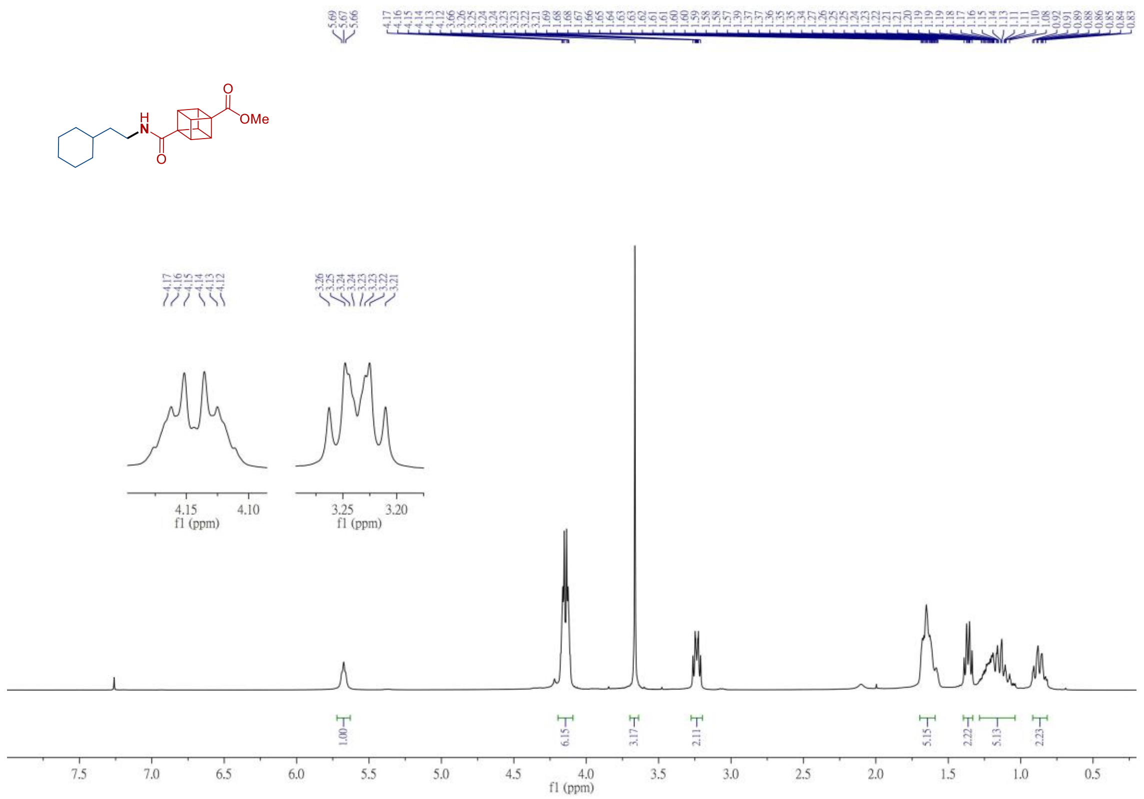

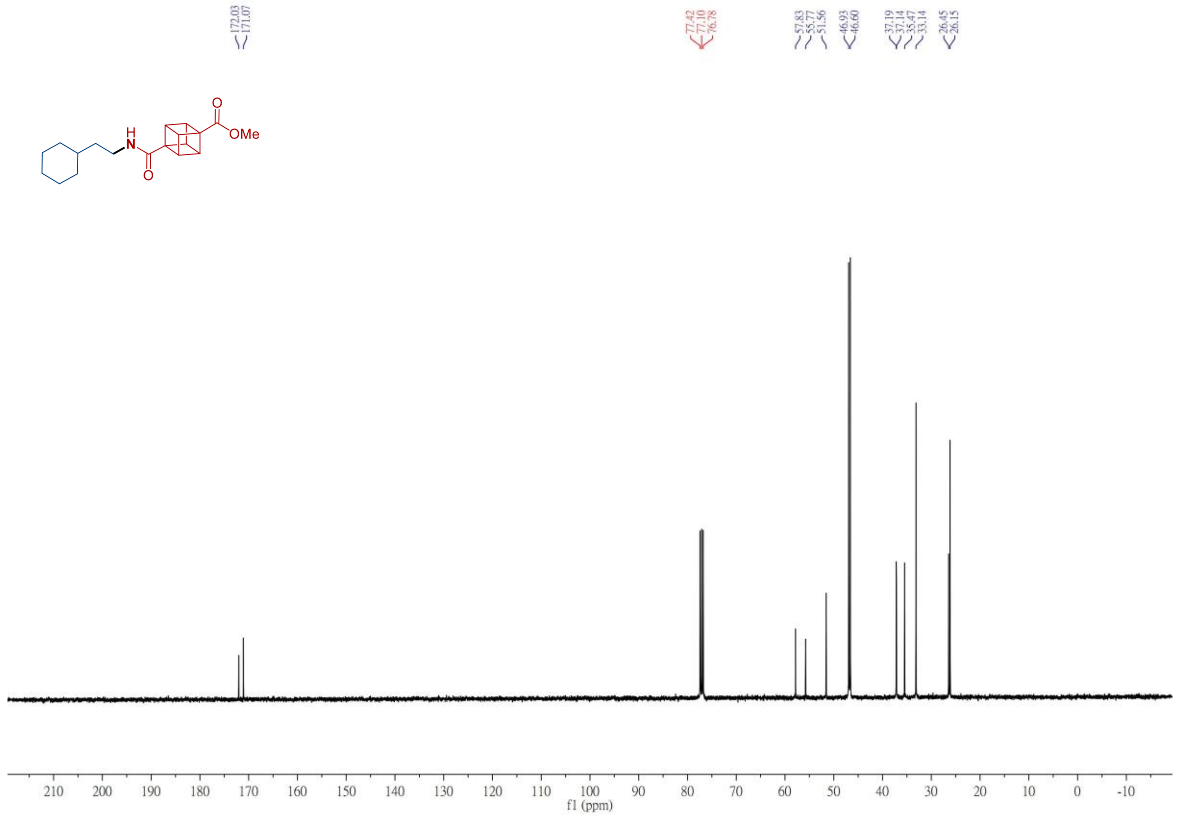

^{1}H and ^{13}C NMR spectrum of **33**

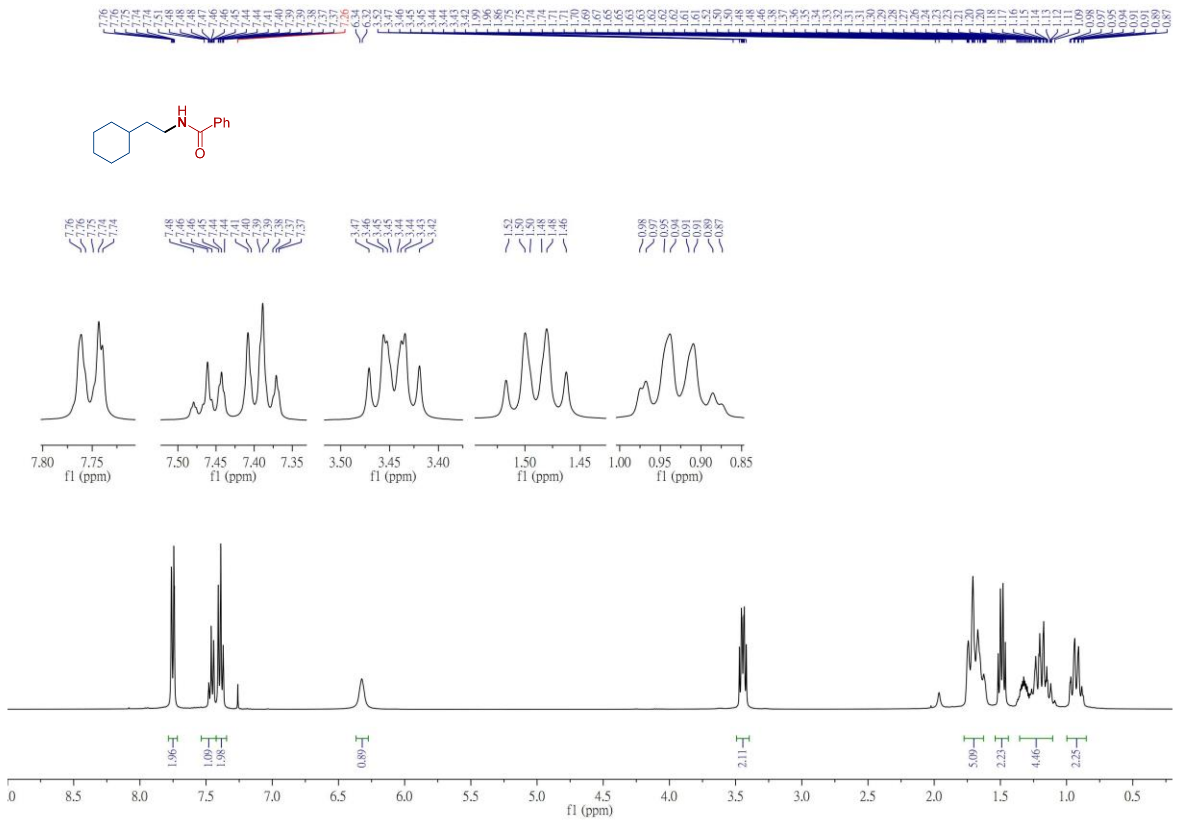

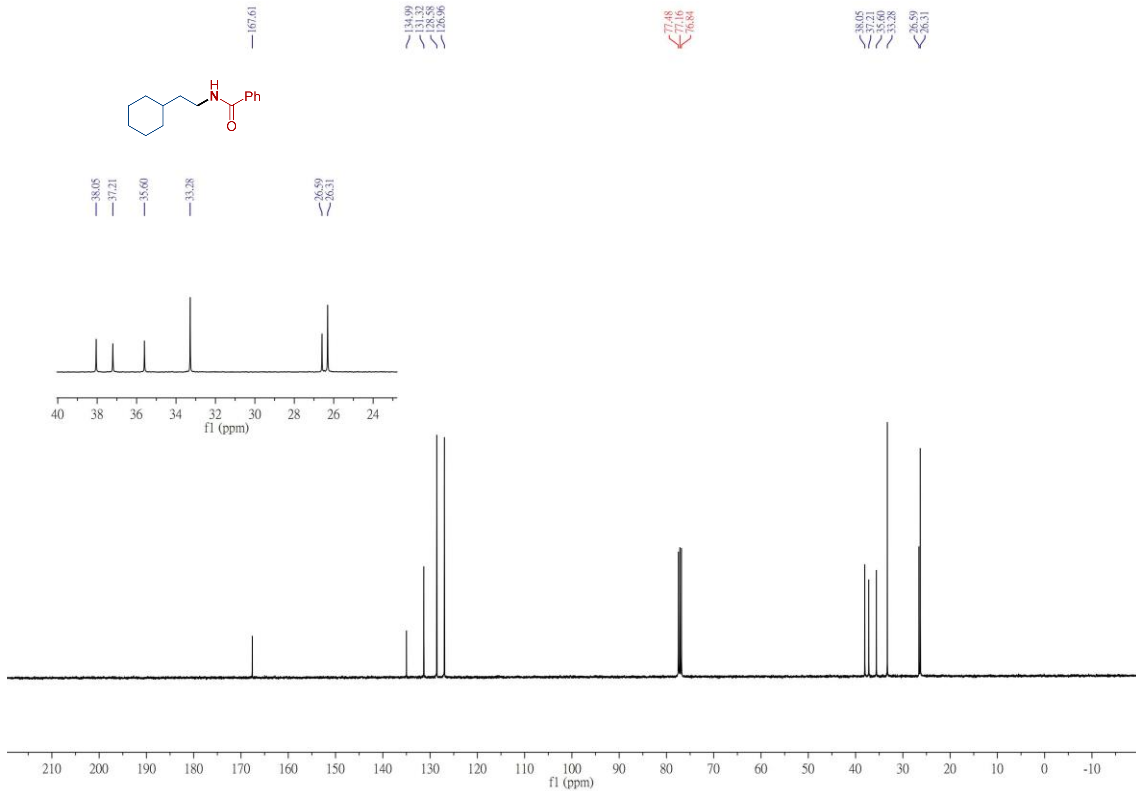

^{1}H and ^{13}C NMR spectrum of **34**

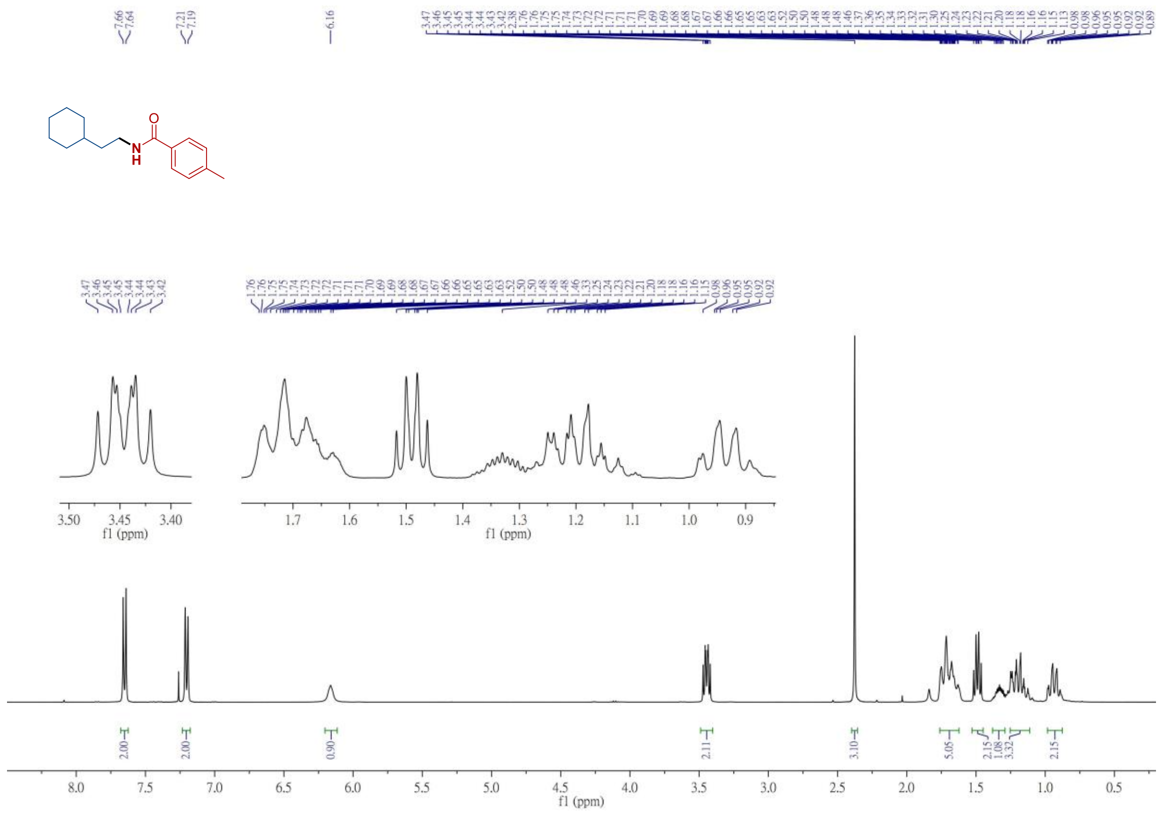

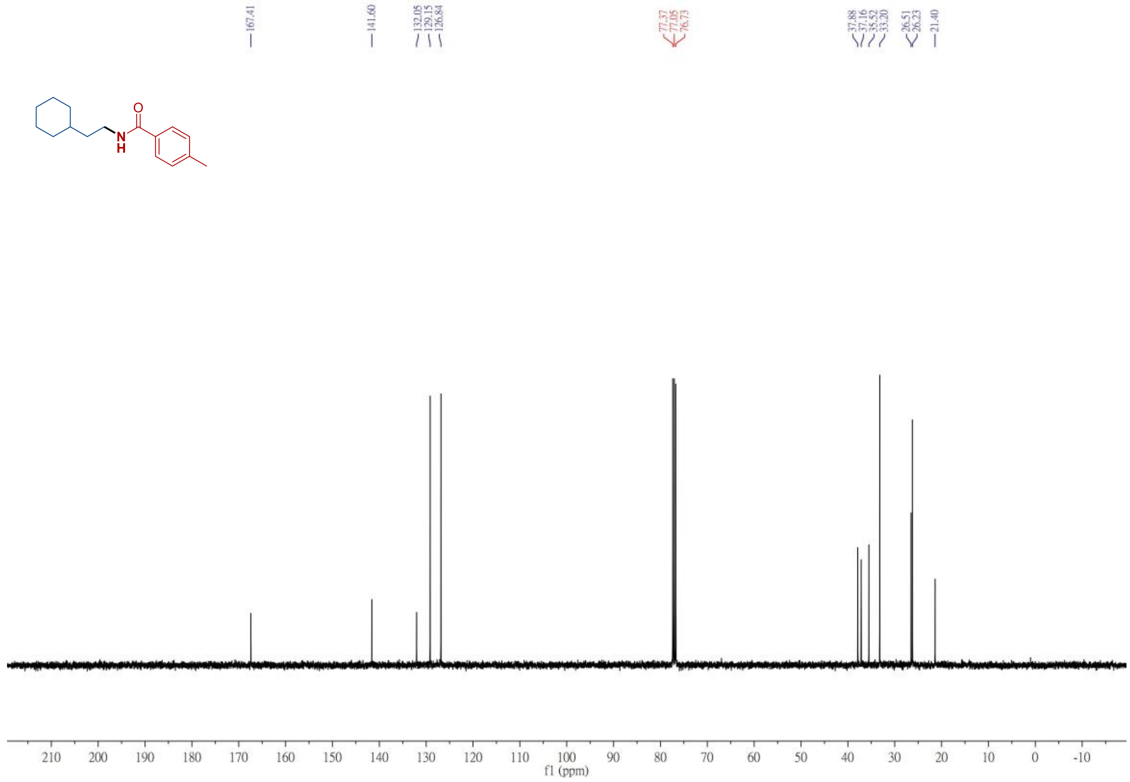

^{1}H and ^{13}C NMR spectrum of **35**

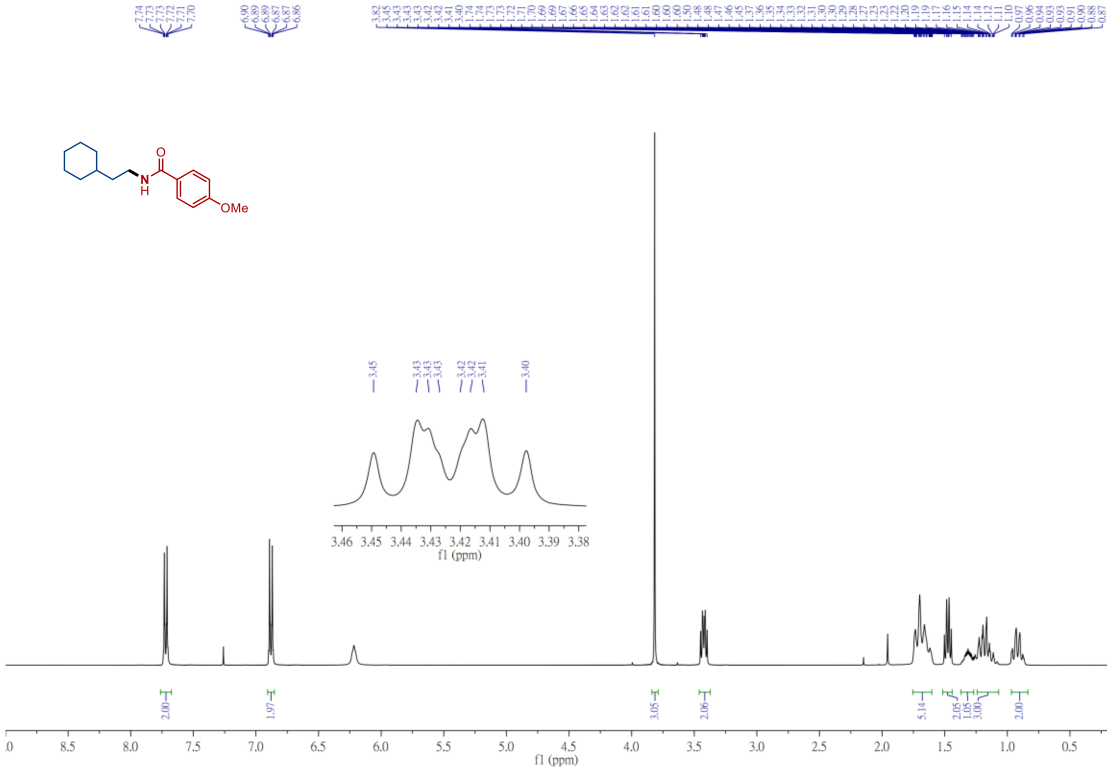

O
N
H
OMe
3.45
3.43
3.43
3.43
3.42
3.42
3.41
3.40
f1 (ppm)
2.00
1.97
3.05
2.06
5.14
2.05
1.05
3.00
2.00
f1 (ppm)

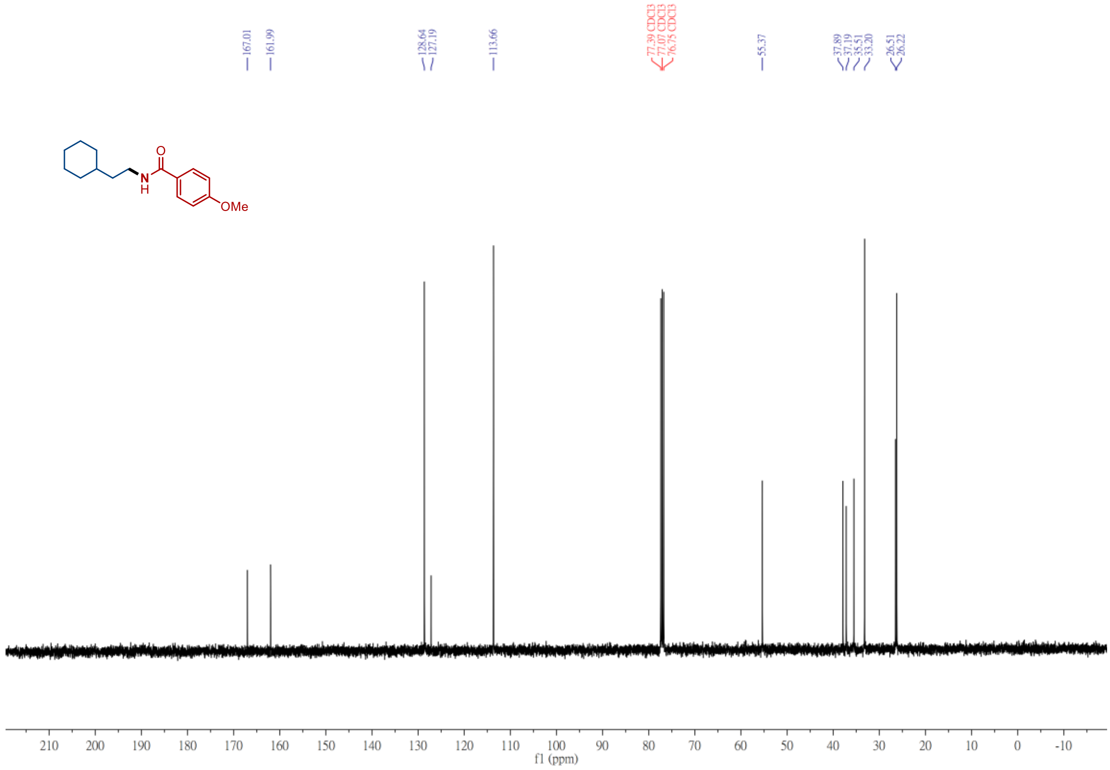

167.01
161.99
128.64
127.19
113.66
77.39 CDCl3
77.07 CDCl3
76.75 CDCl3
55.37
37.89
37.19
35.51
33.20
26.51
26.22
O
N
H
OMe
f1 (ppm)

^{1}H and ^{13}C NMR spectrum of **36**

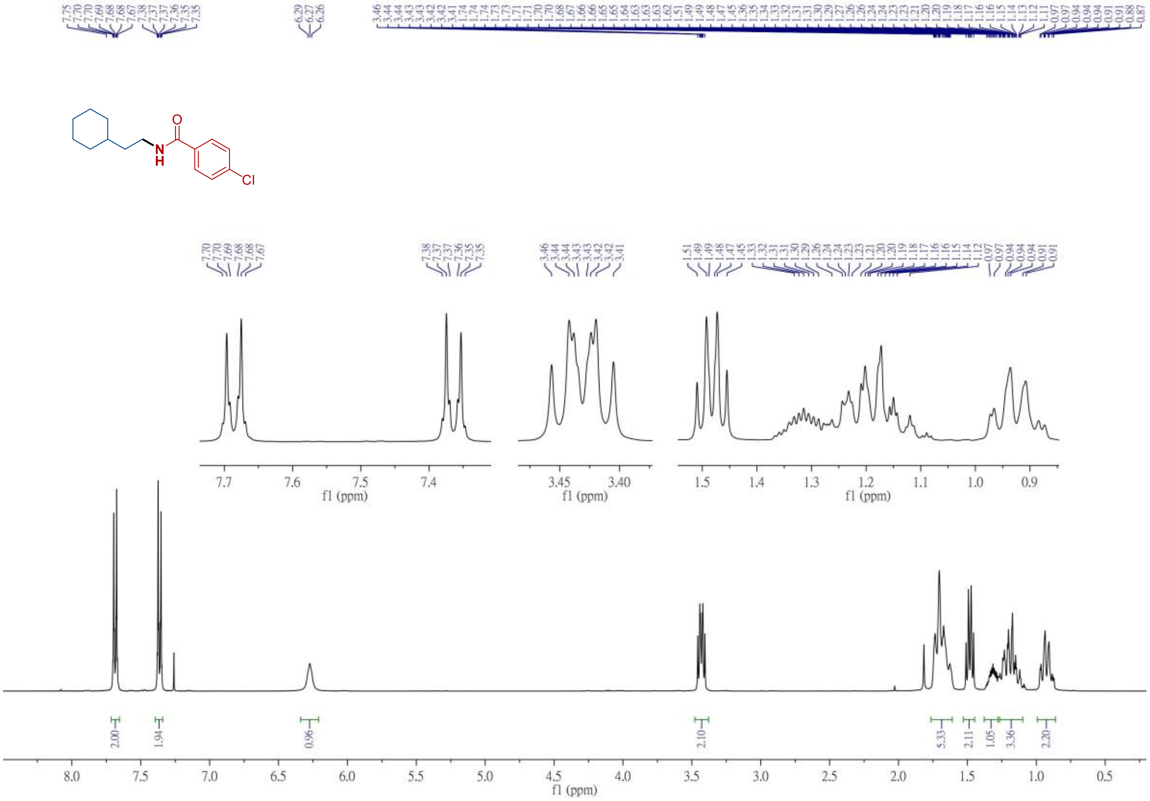

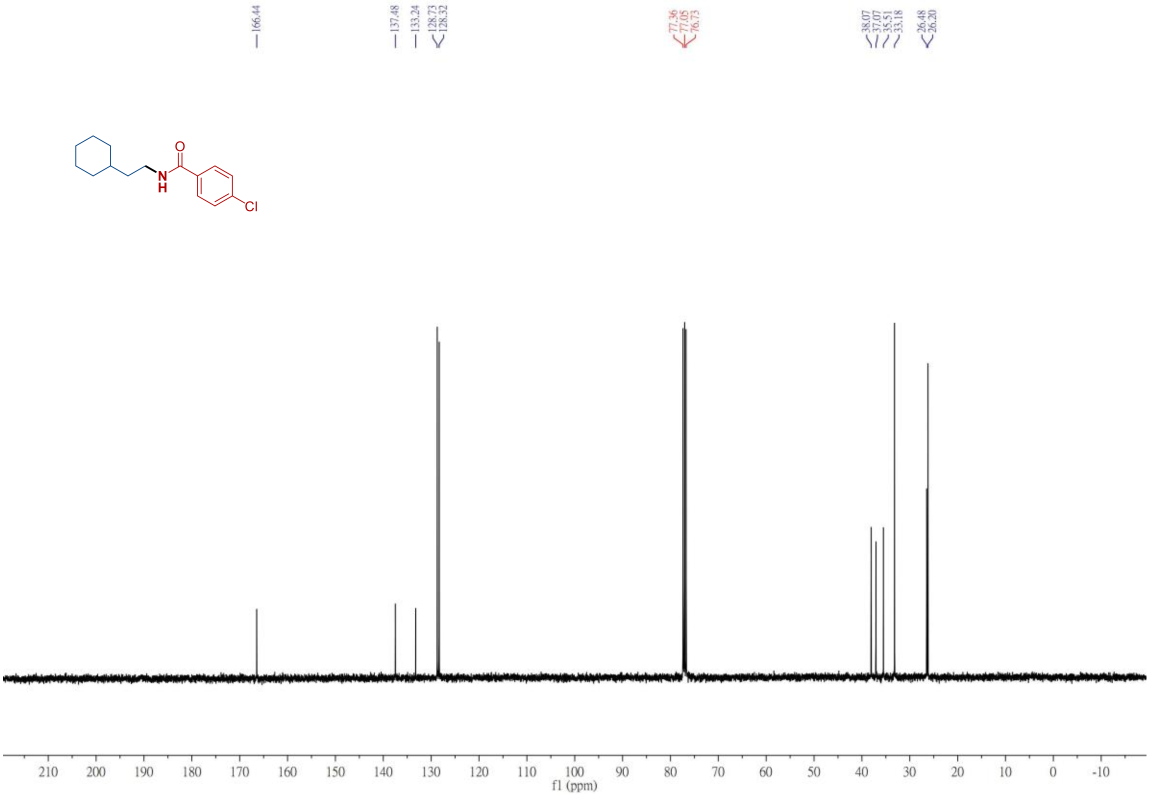

^1H and ^{13}C NMR spectrum of **37**

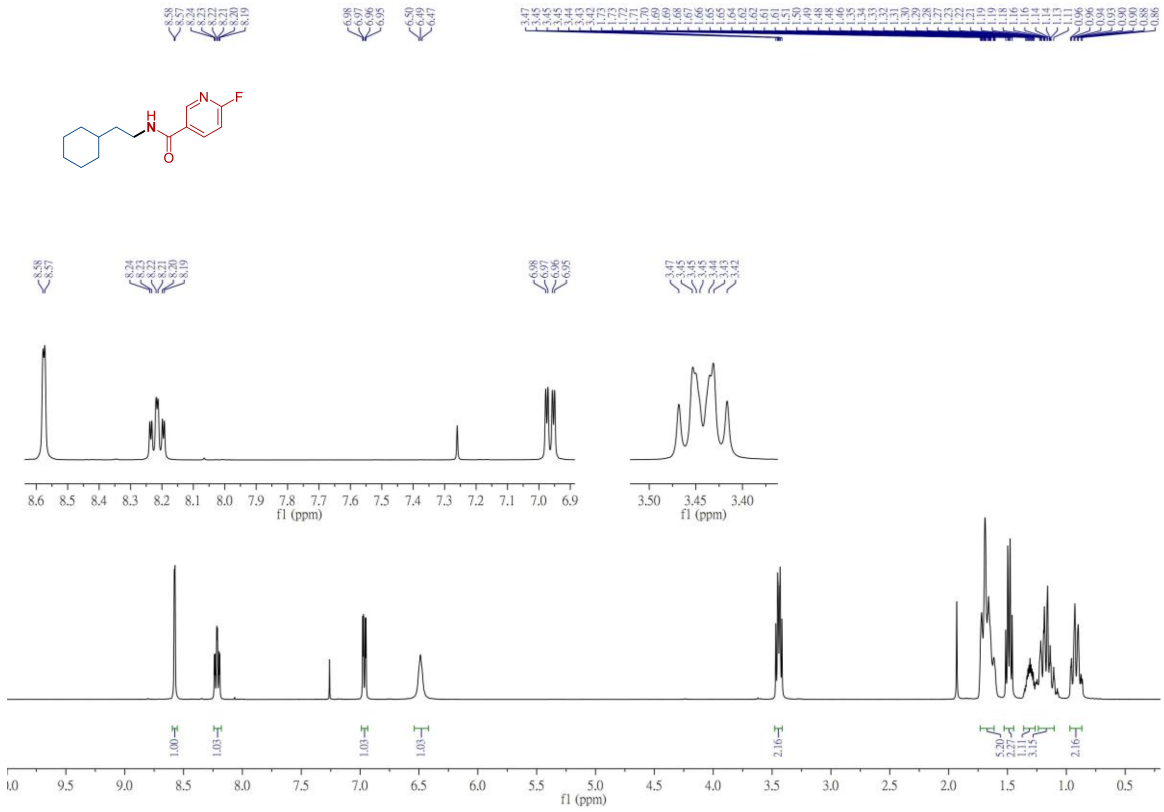

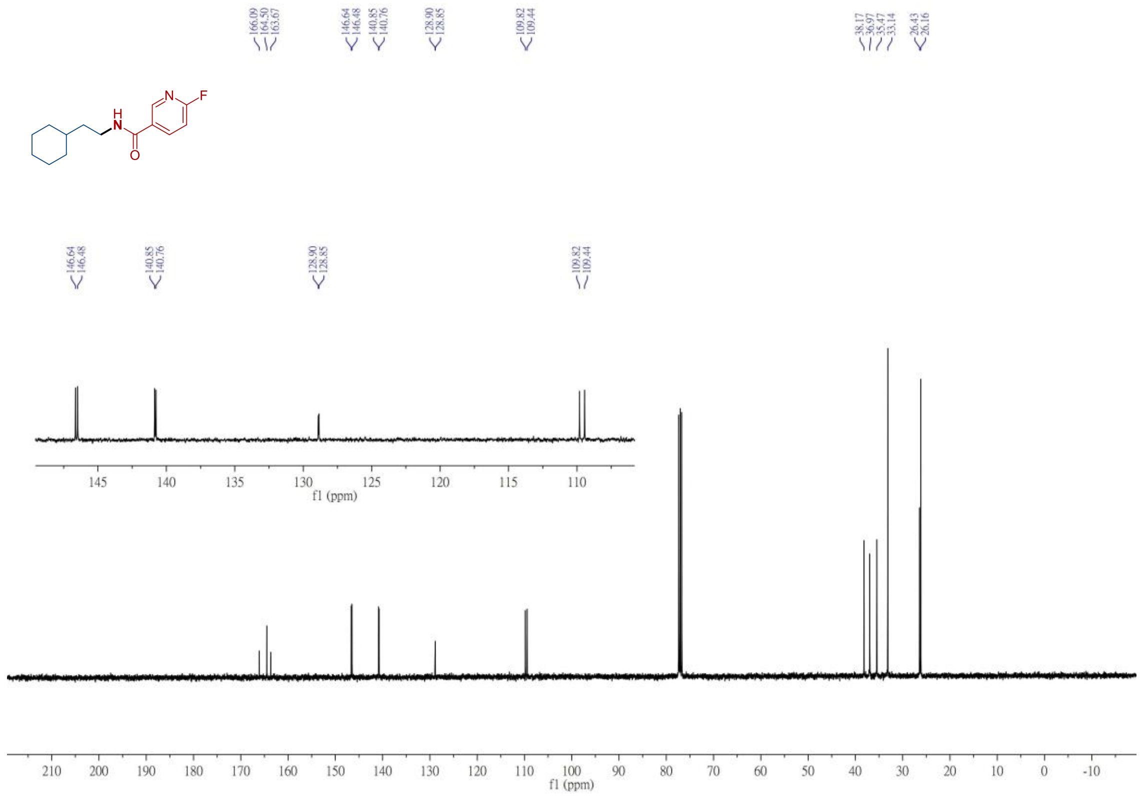

^{19}F NMR spectrum of **37**

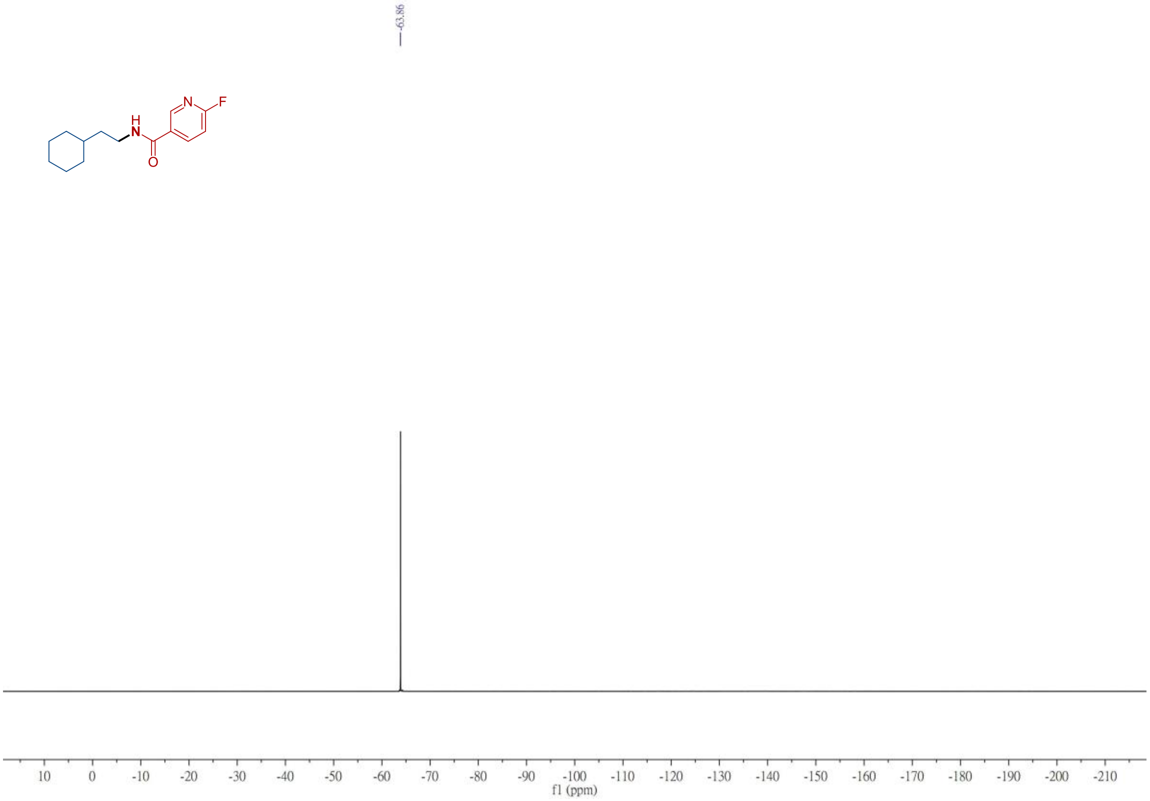

^{1}H and ^{13}C NMR spectrum of **38**

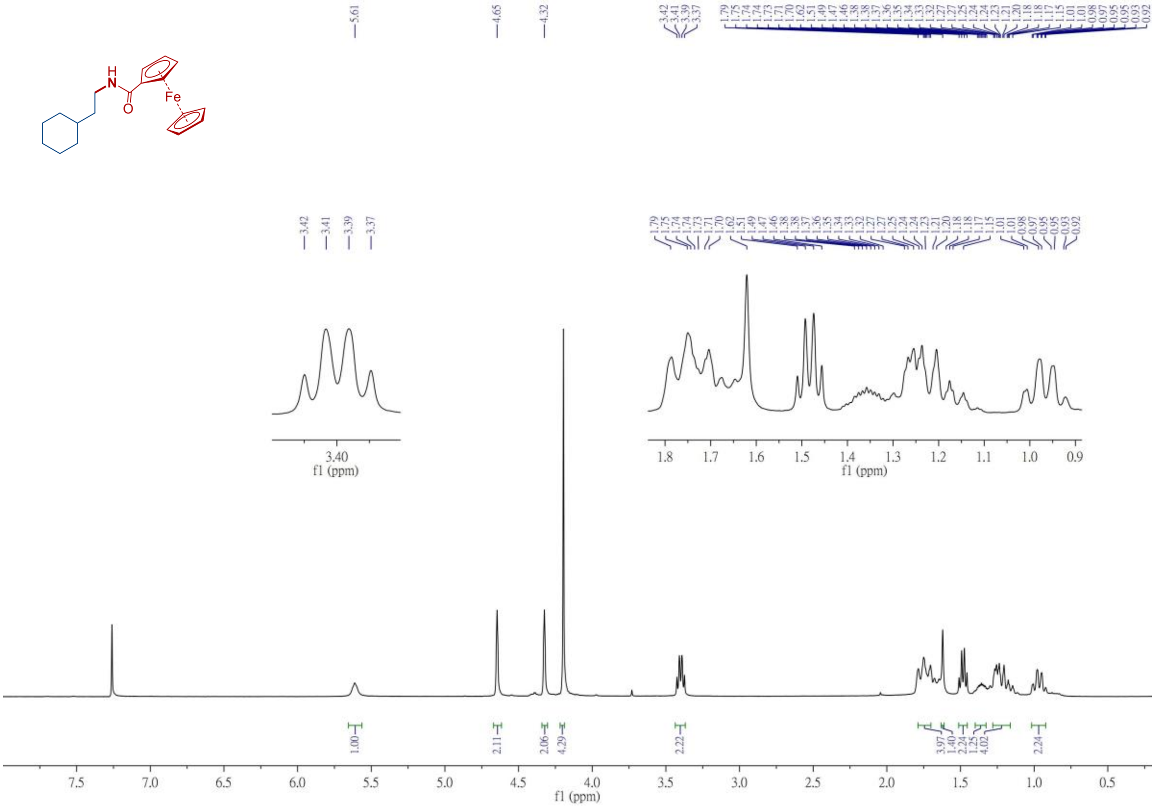

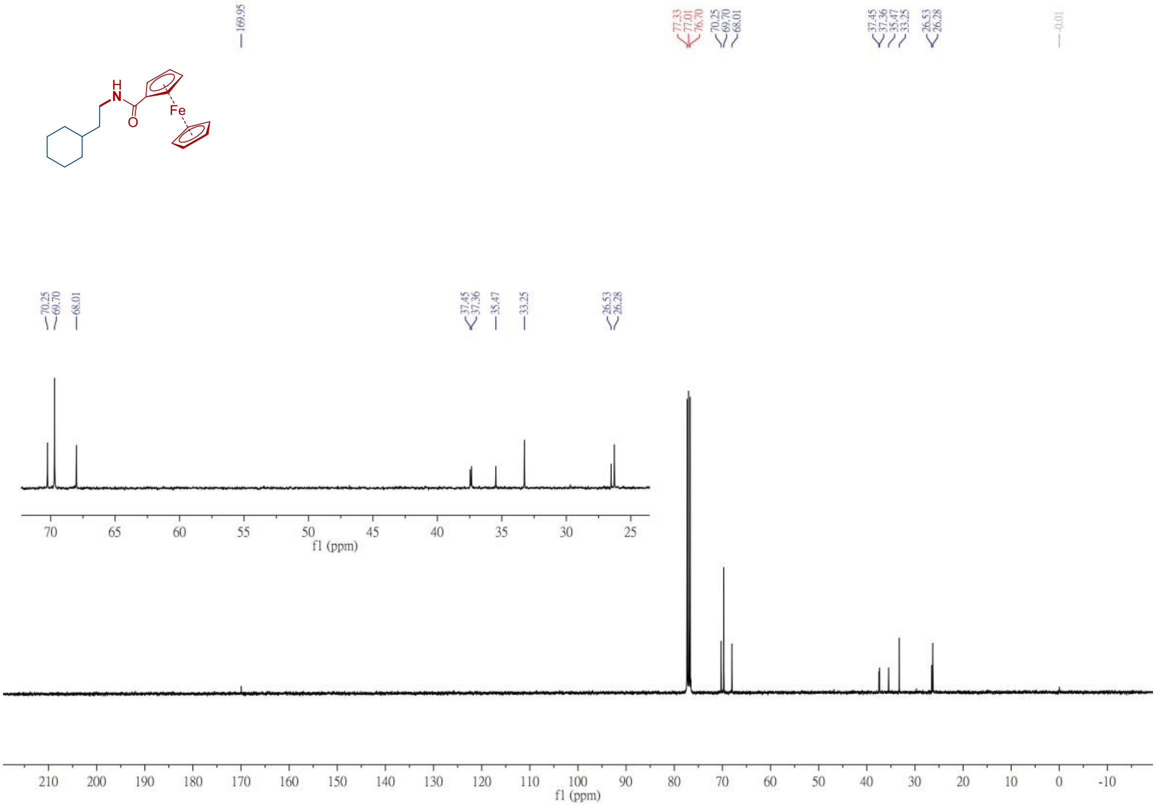

^{1}H and ^{13}C NMR spectrum of **39**

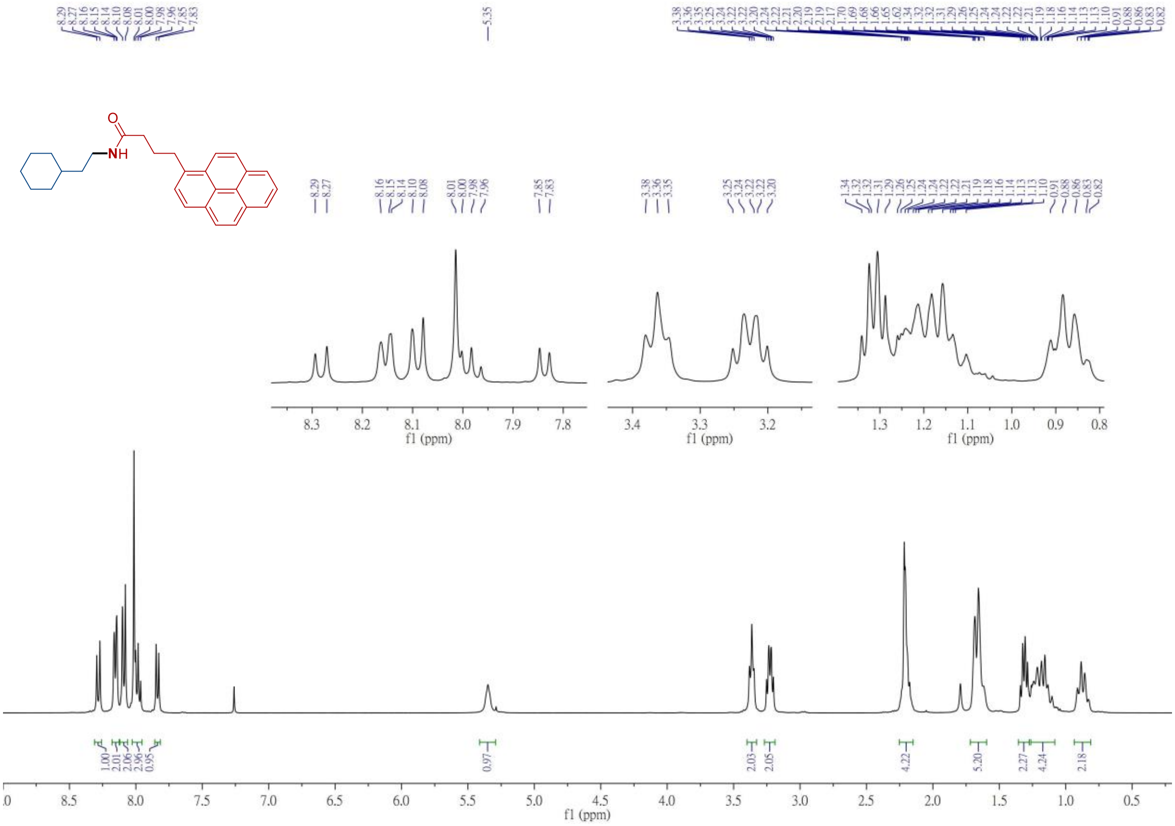

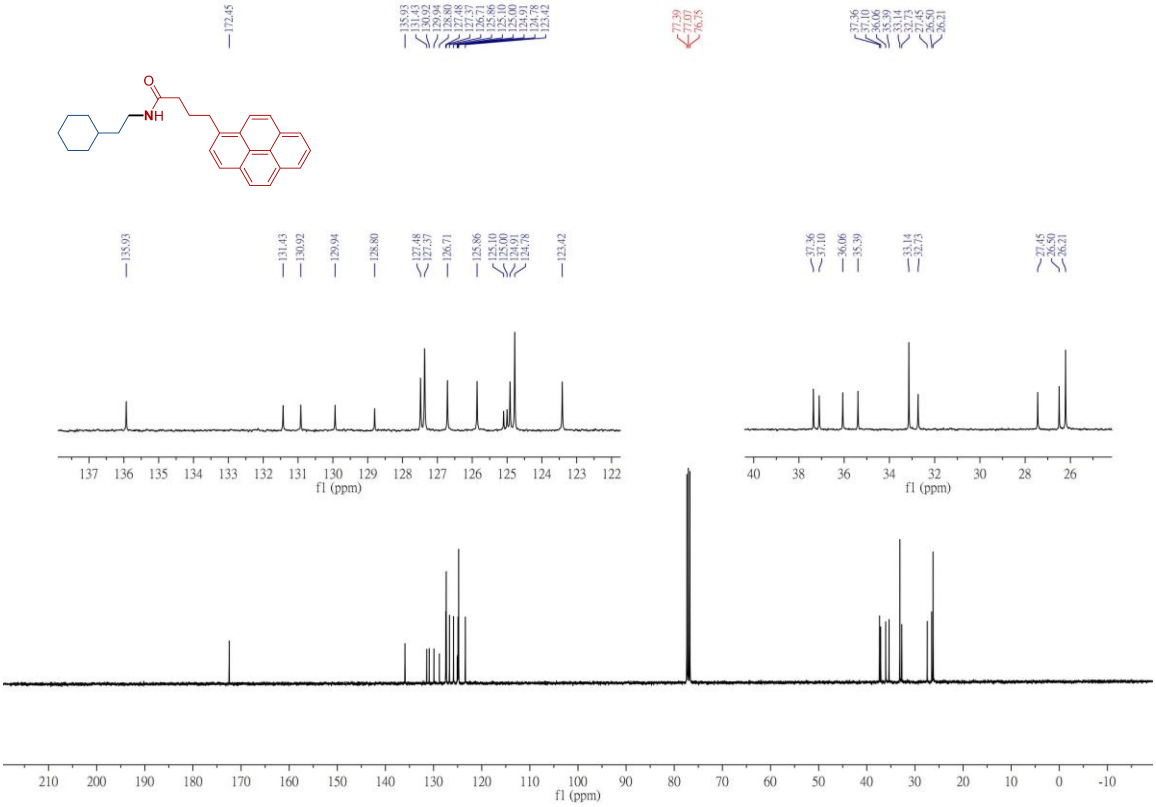

^{1}H and ^{13}C NMR spectrum of **40**

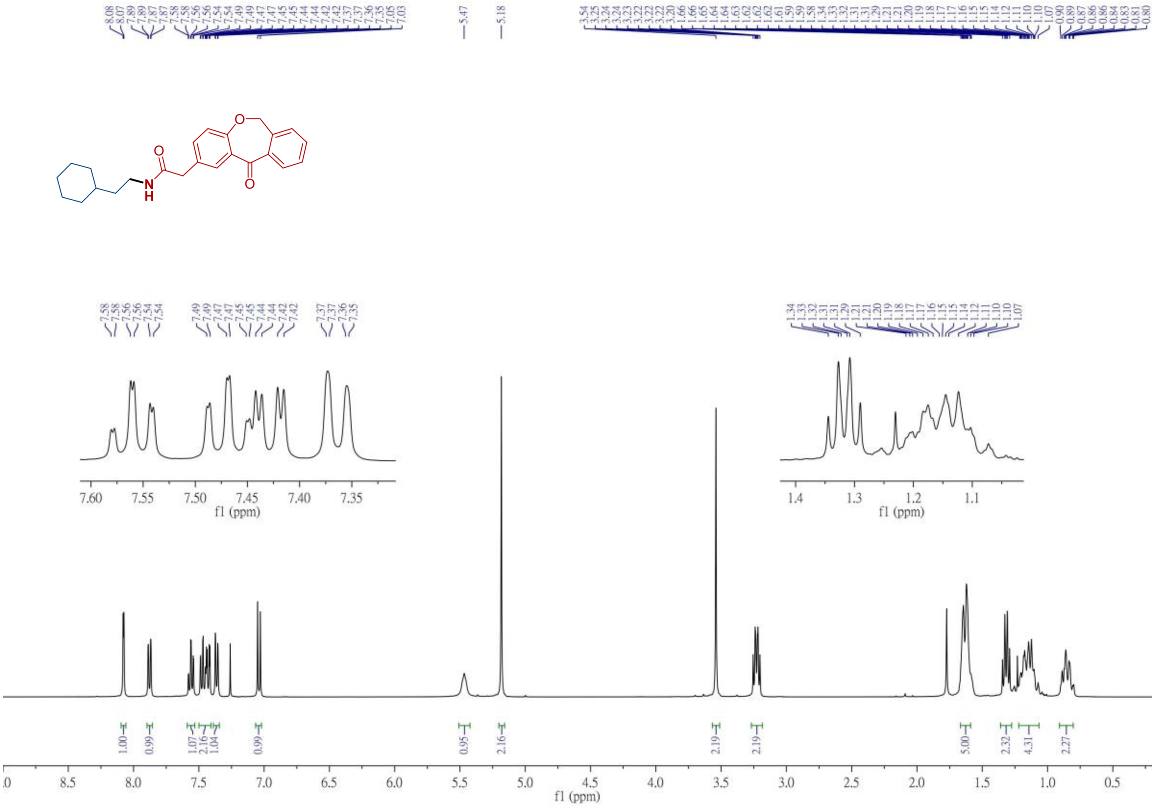

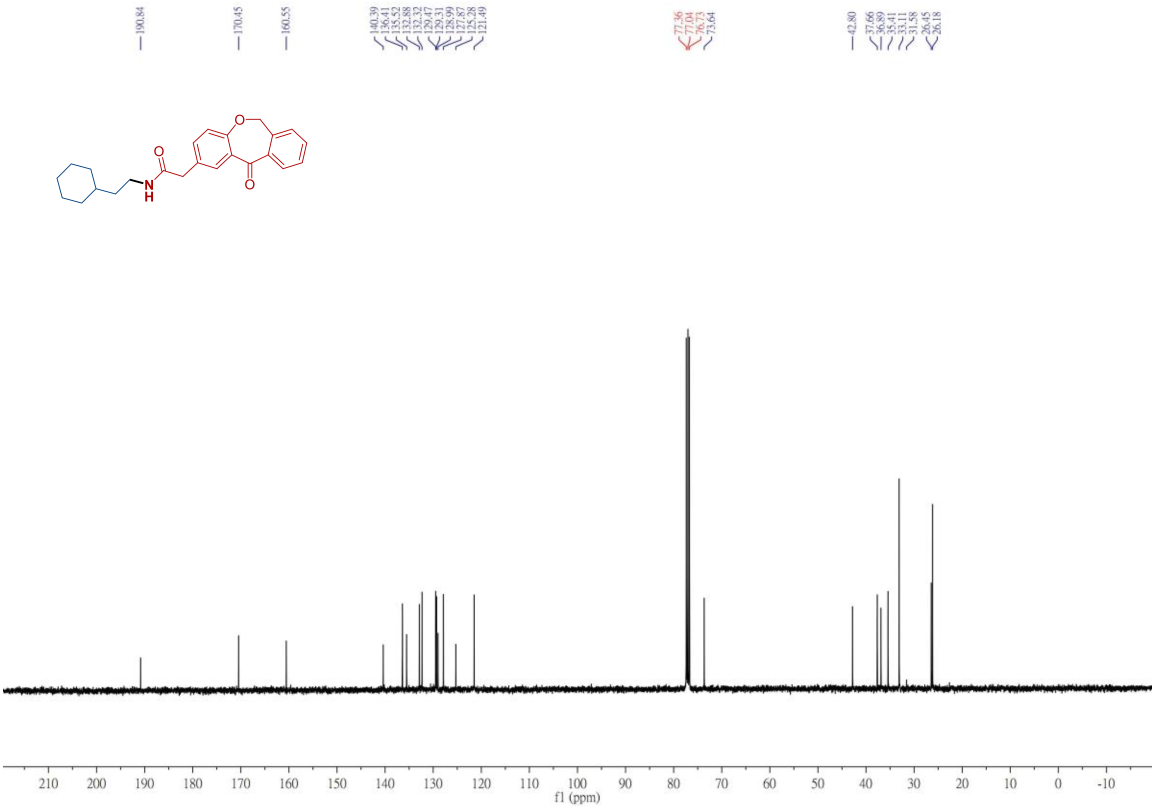

^{1}H and ^{13}C NMR spectrum of **41**

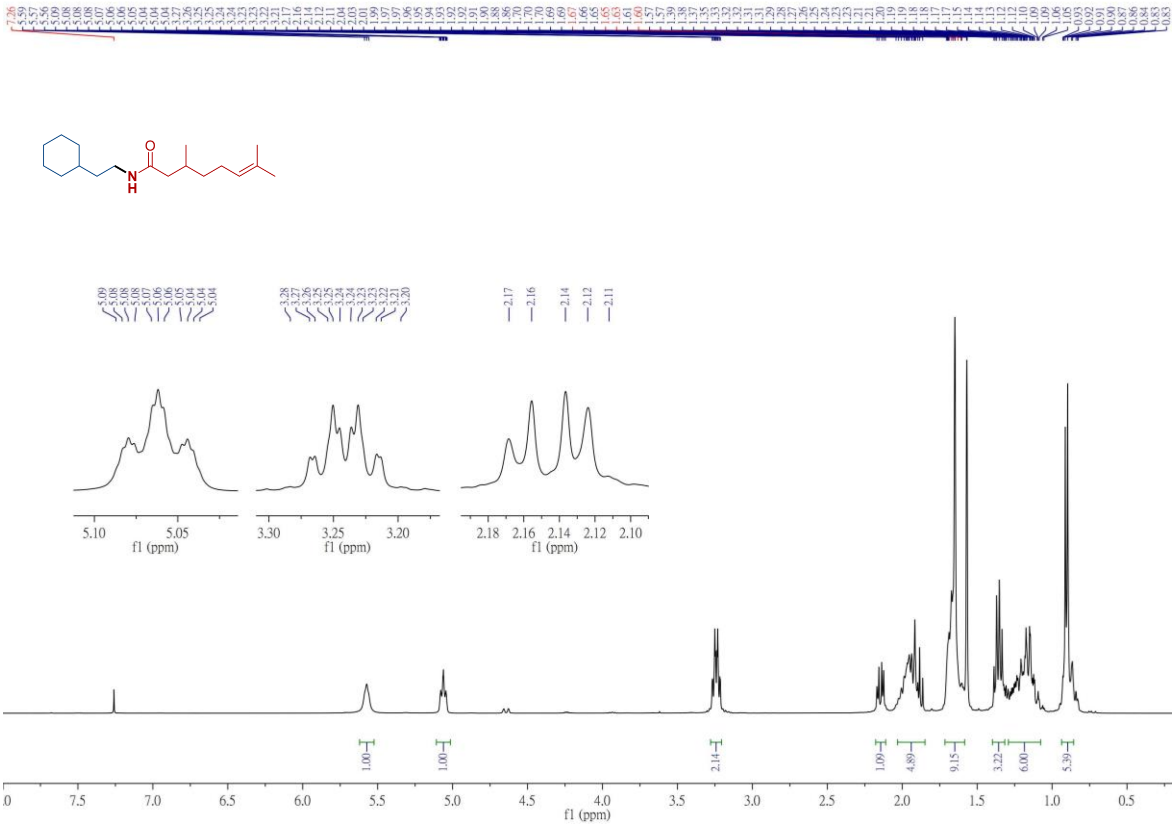
f1 (ppm)

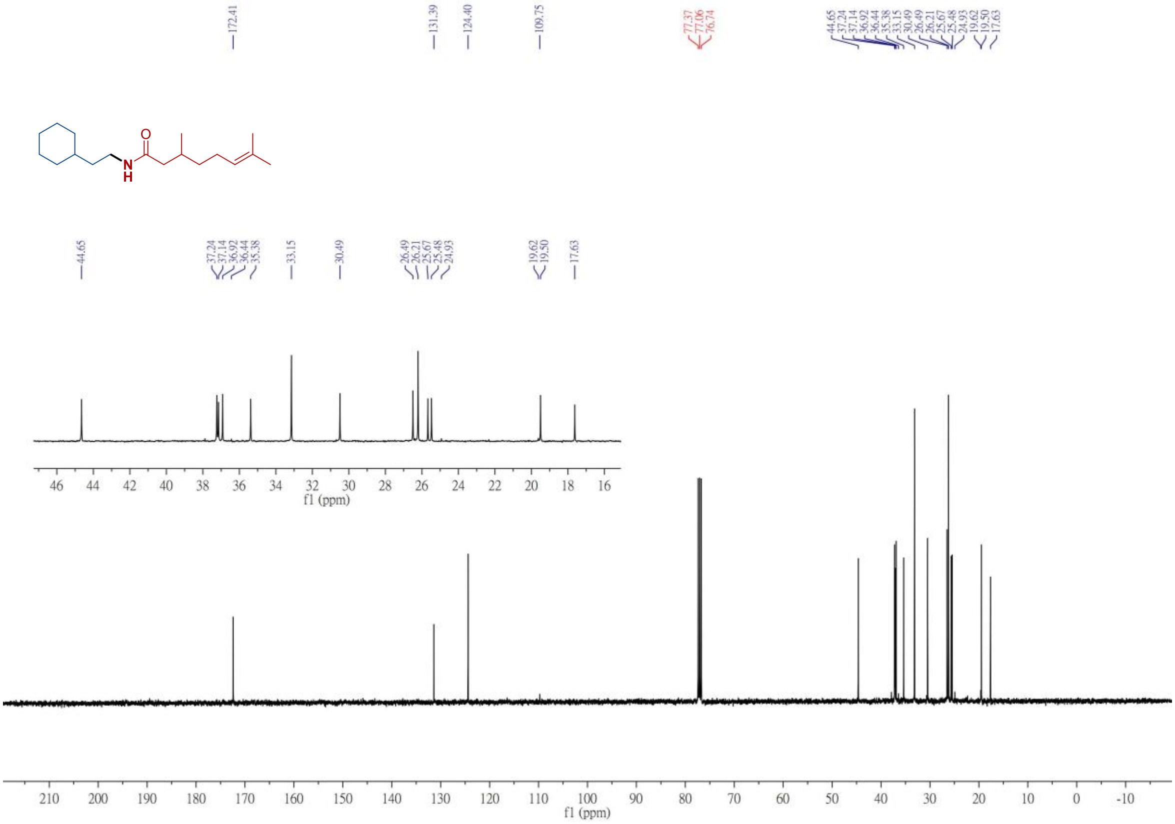
f1 (ppm)

^{1}H and ^{13}C NMR spectrum of **42**

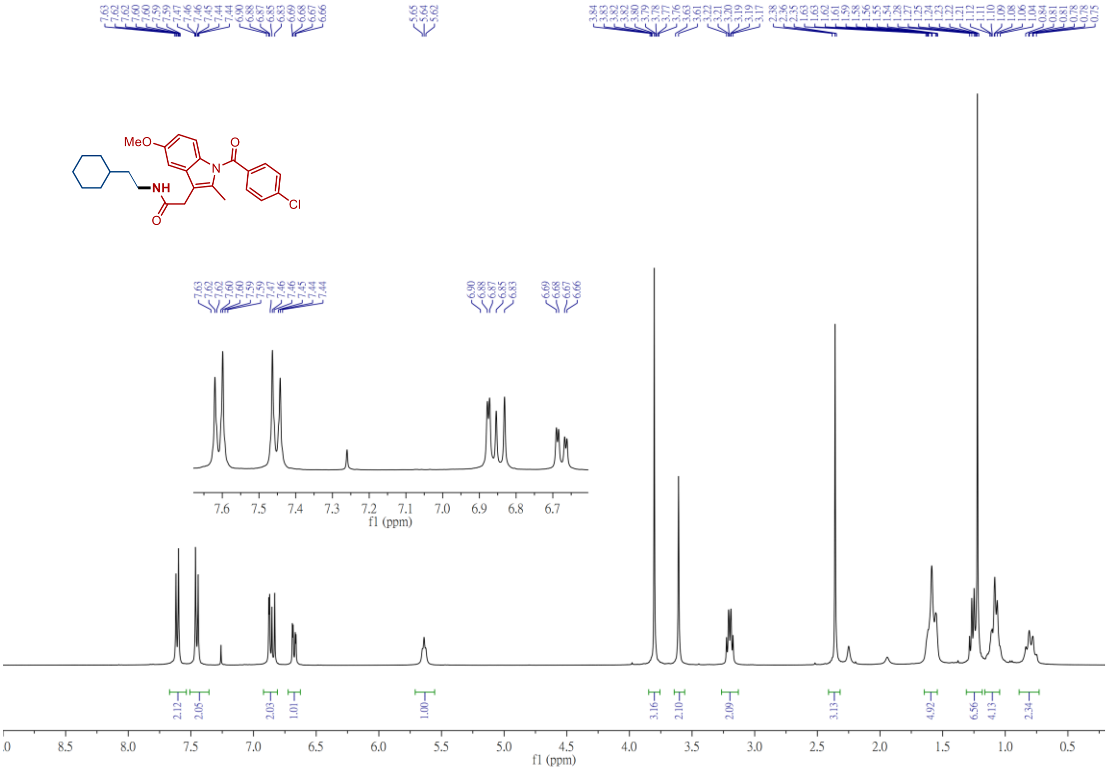

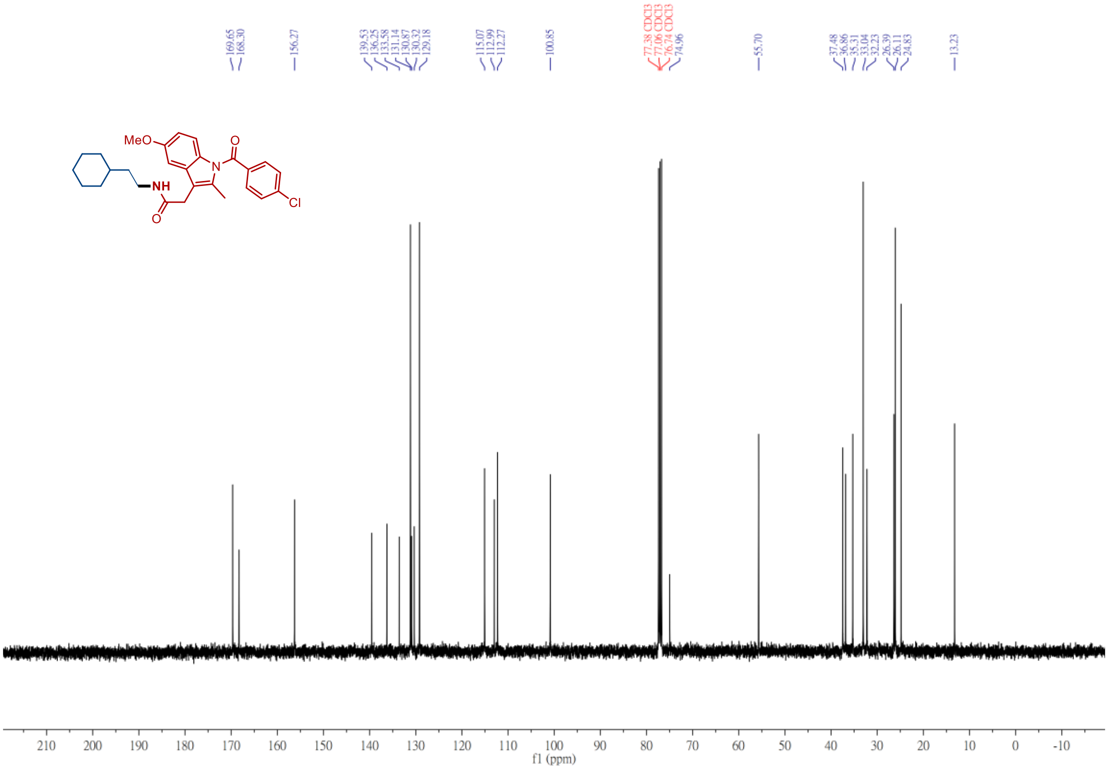

^{1}H and ^{13}C NMR spectrum of **43**

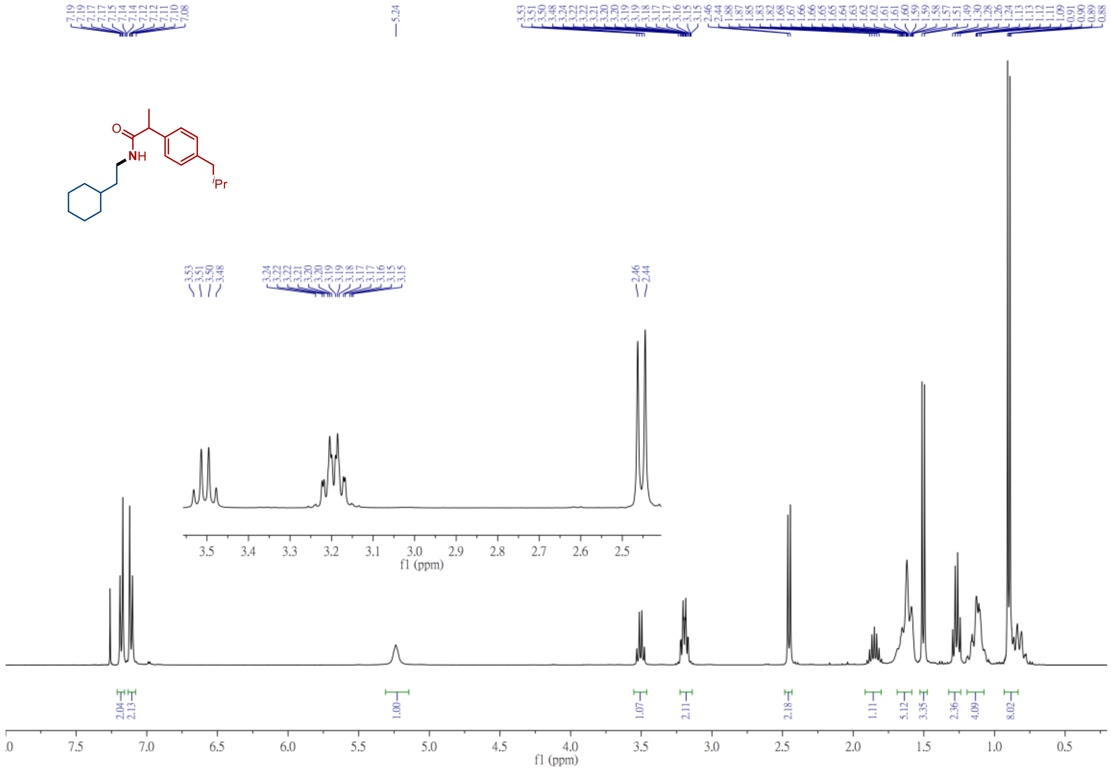

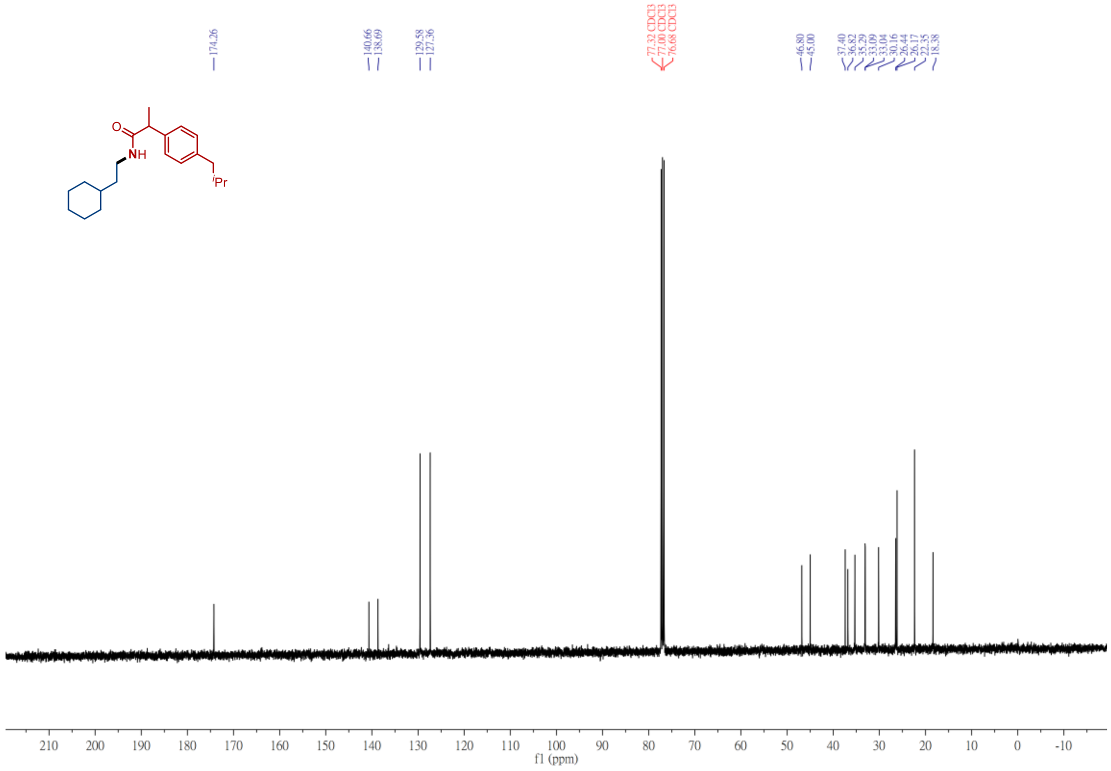

^{1}H and ^{13}C NMR spectrum of **44**

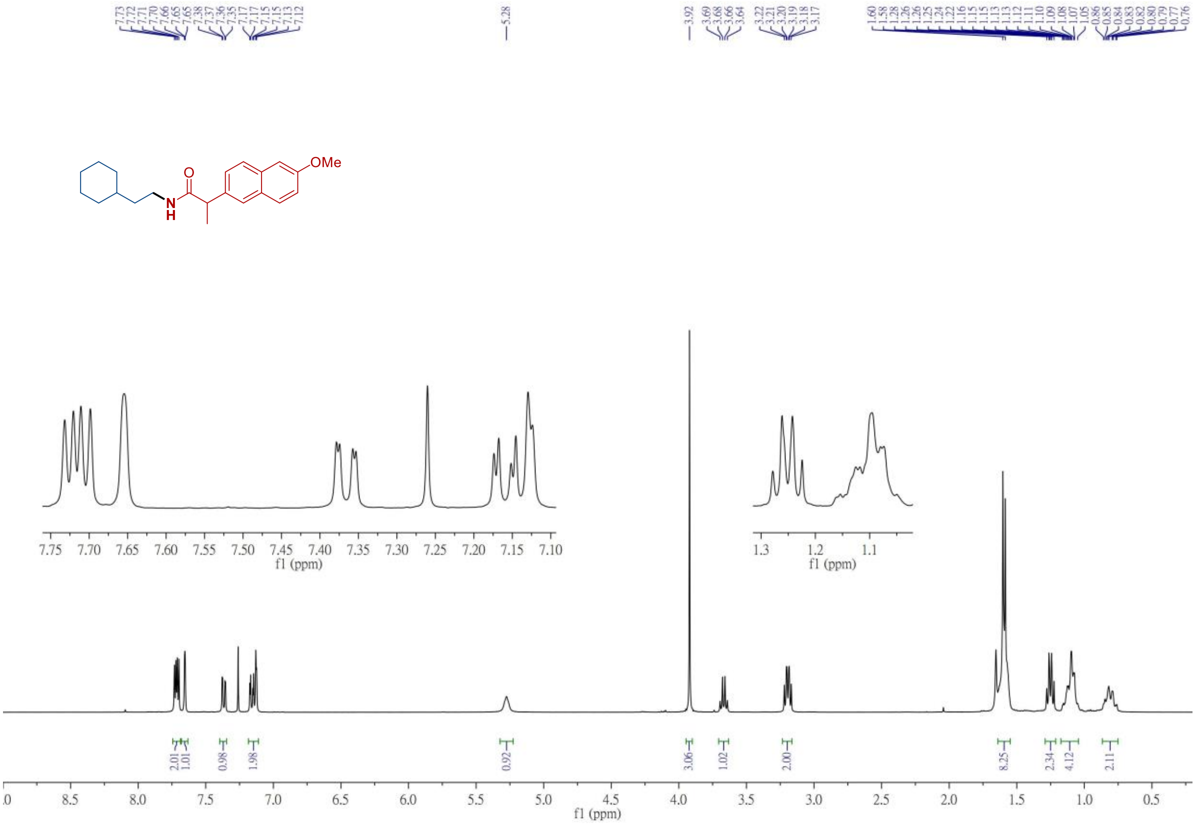

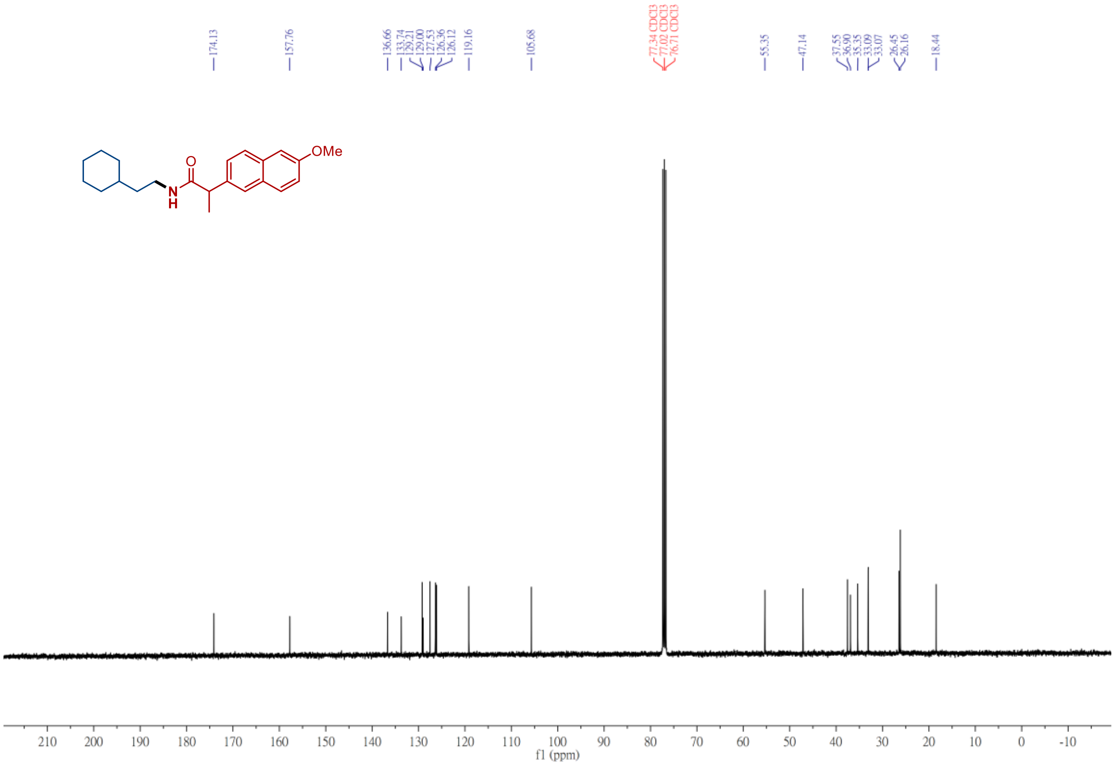

^{1}H and ^{13}C NMR spectrum of **45**

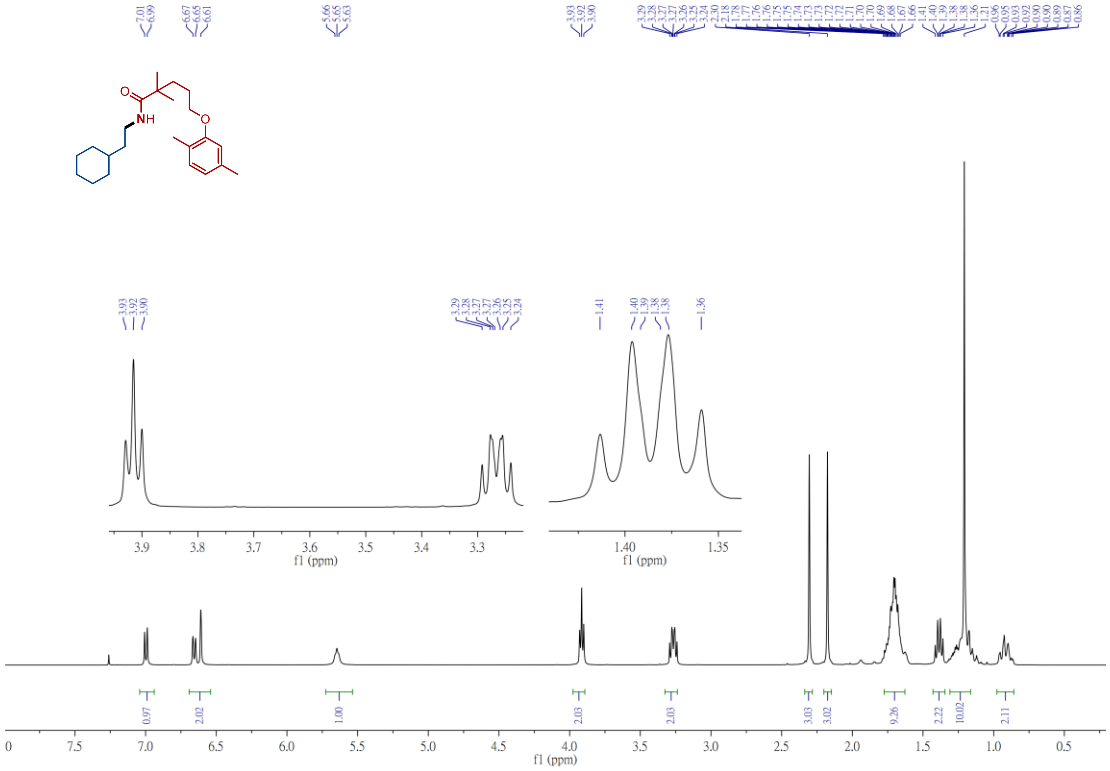

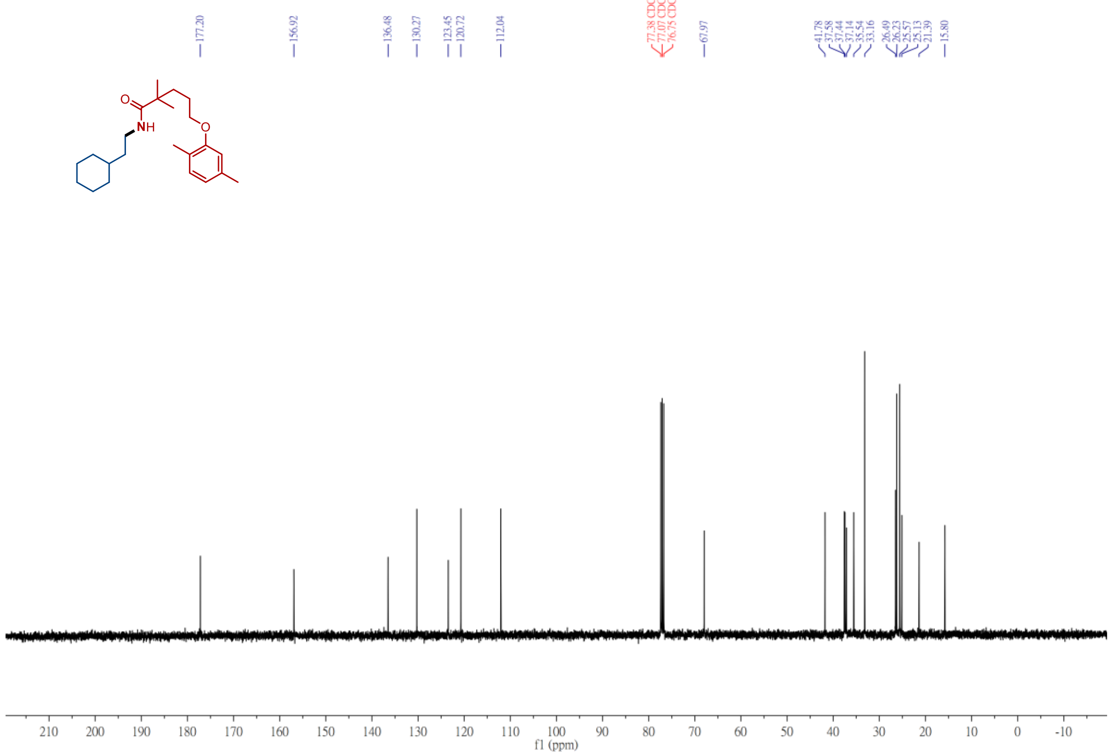

^{1}H and ^{13}C NMR spectrum of **46**

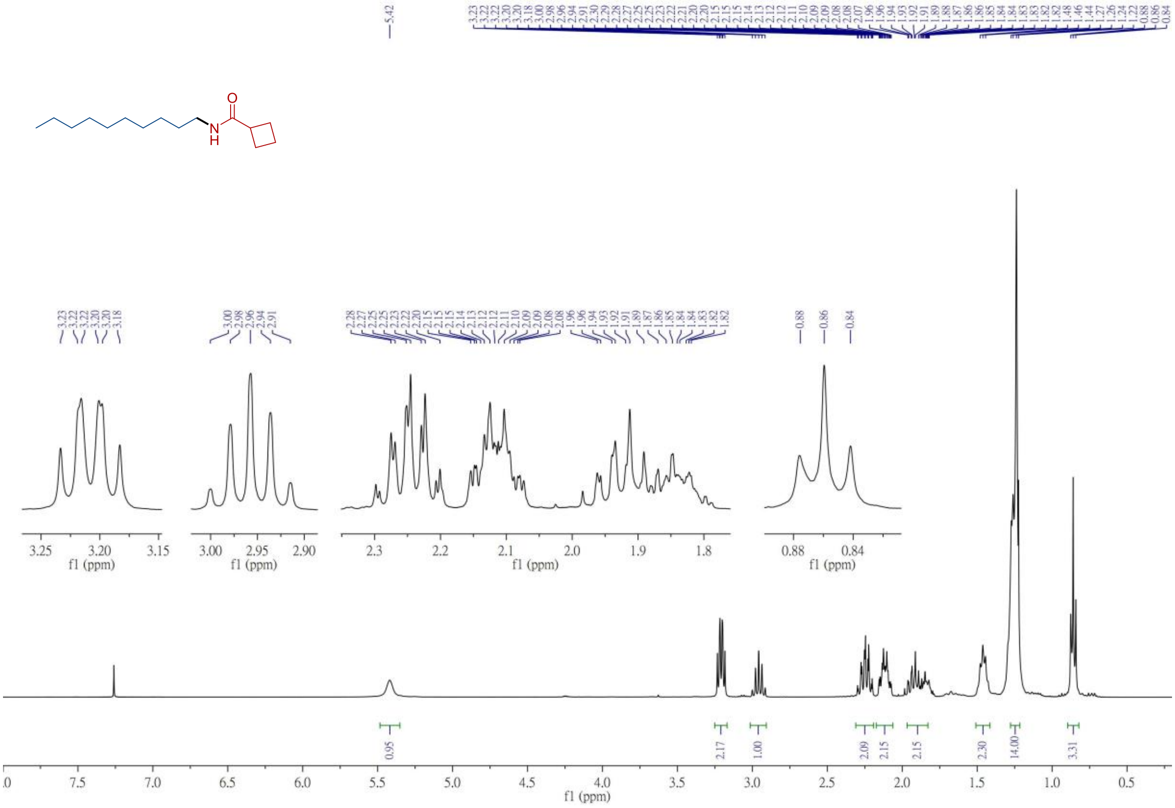

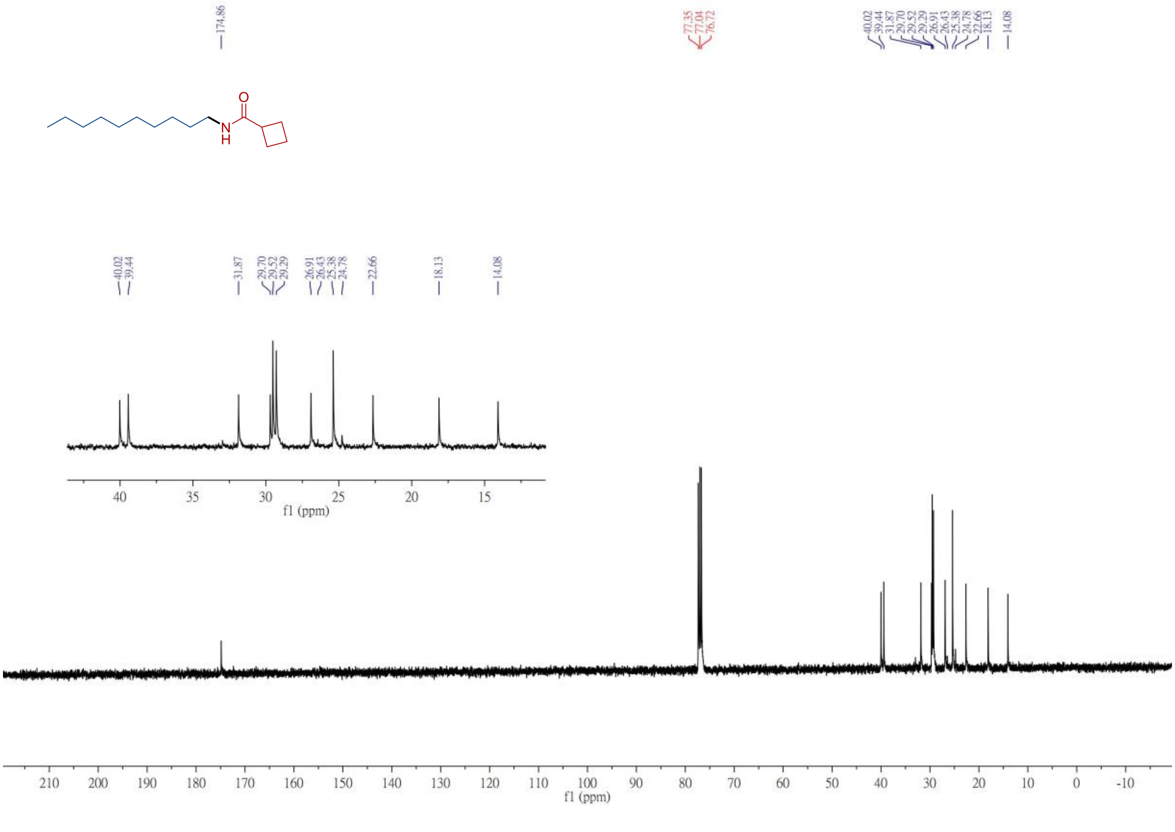

^{1}H and ^{13}C NMR spectrum of **47**

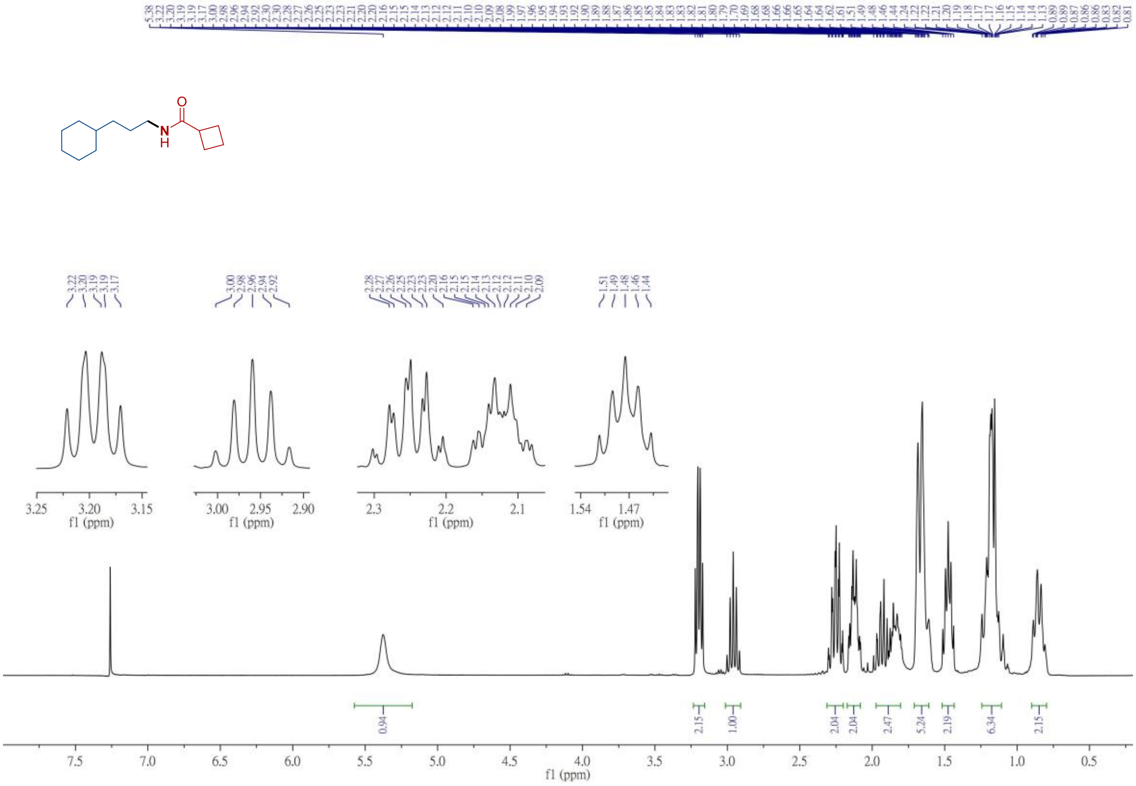

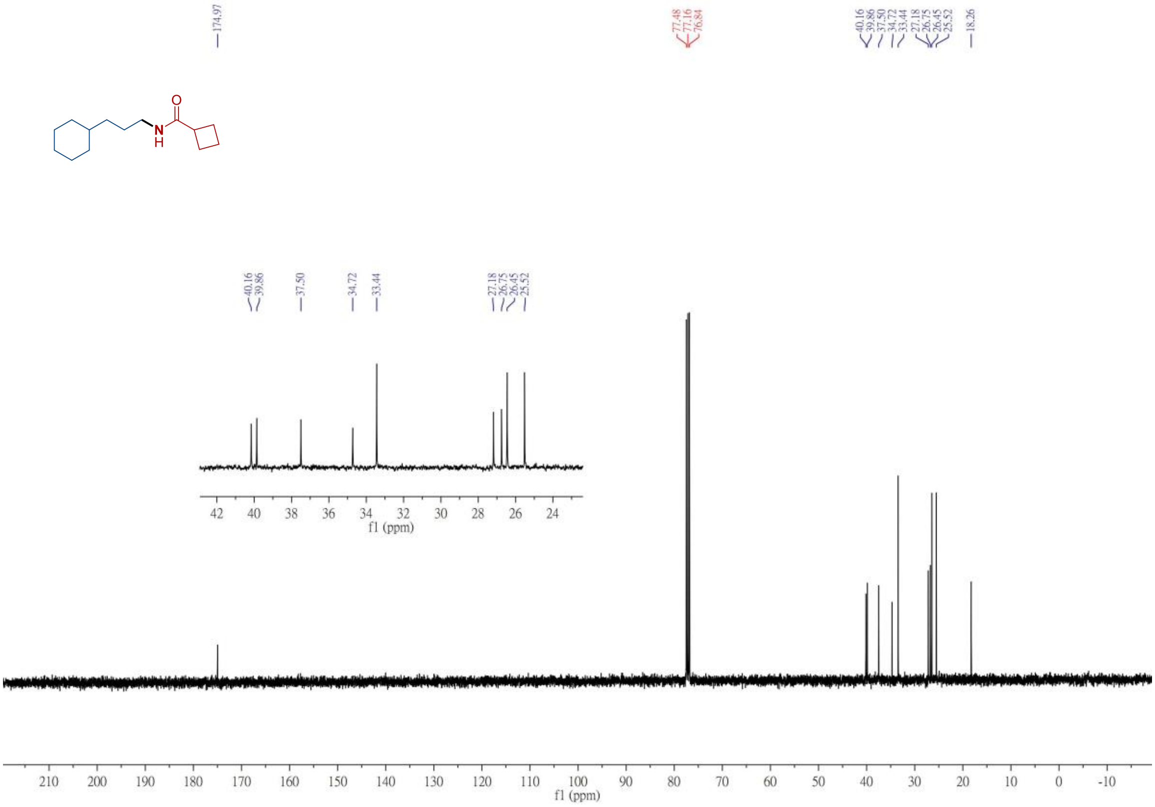

^{1}H and ^{13}C NMR spectrum of **48**

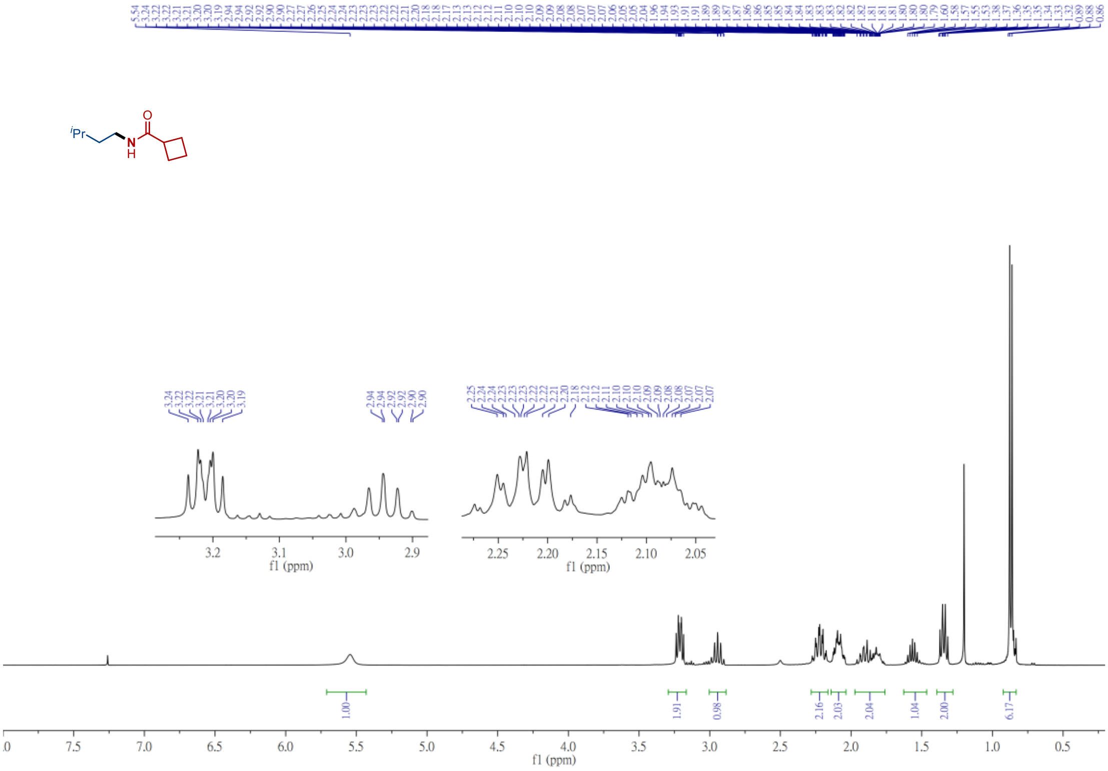

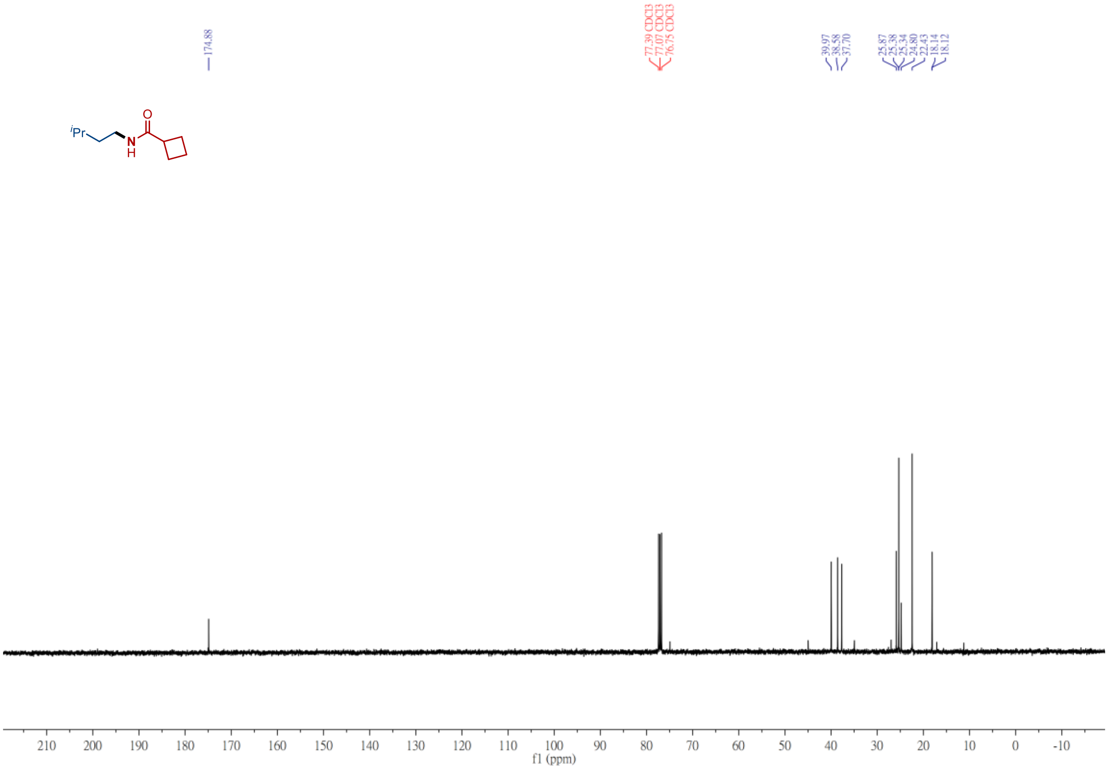

^{1}H and ^{13}C NMR spectrum of **49**

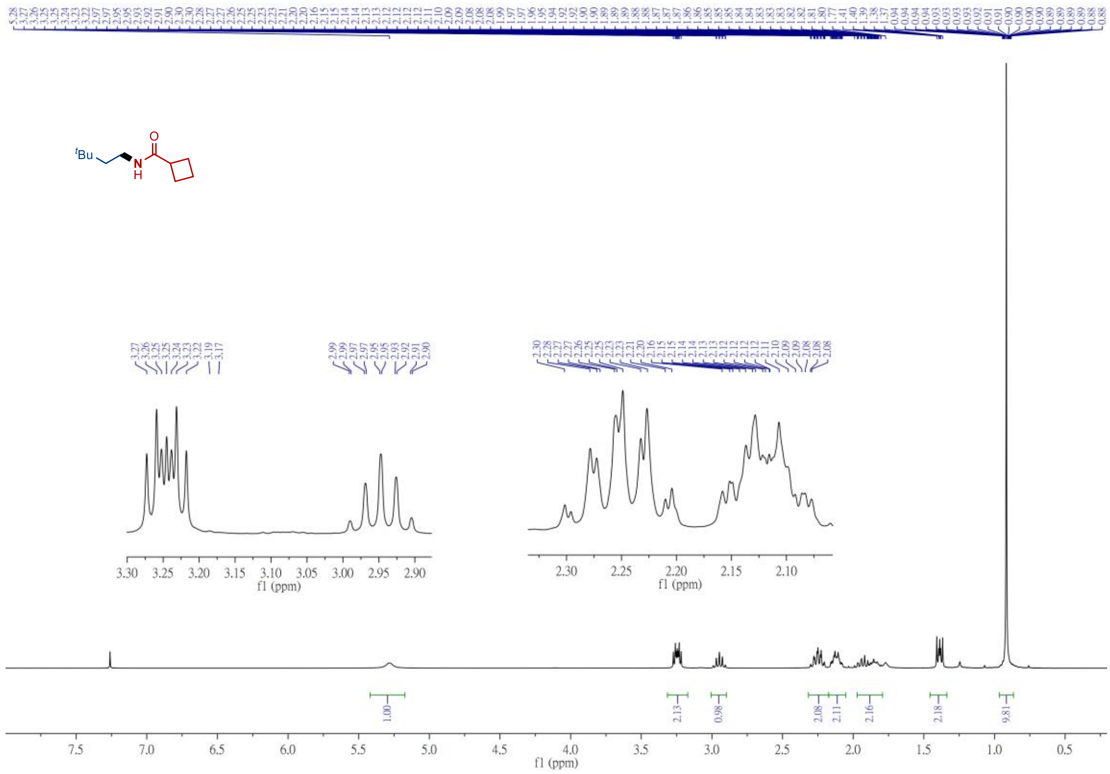

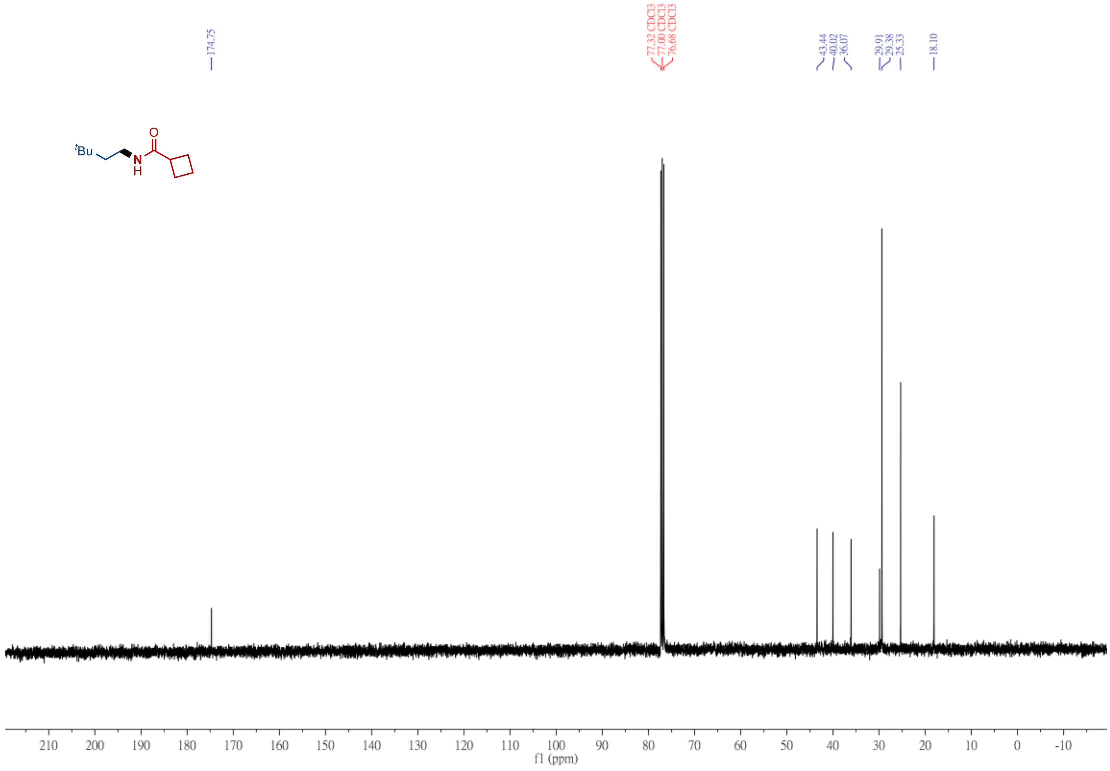

^{1}H and ^{13}C NMR spectrum of **50**

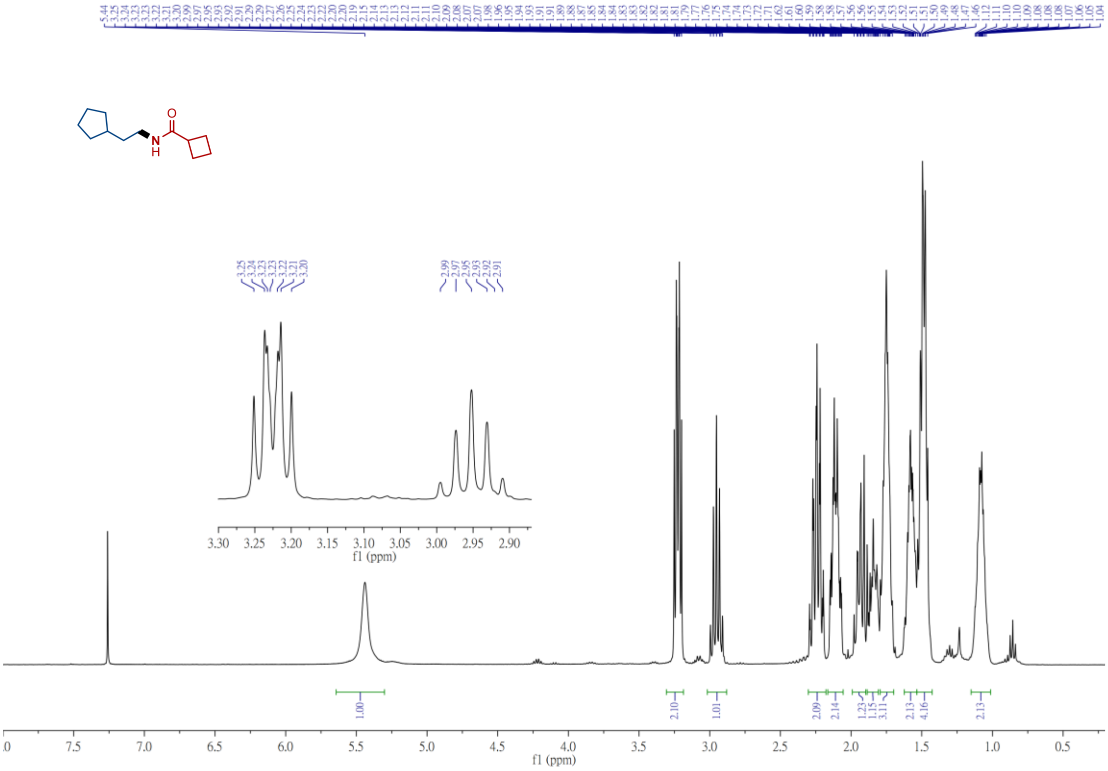

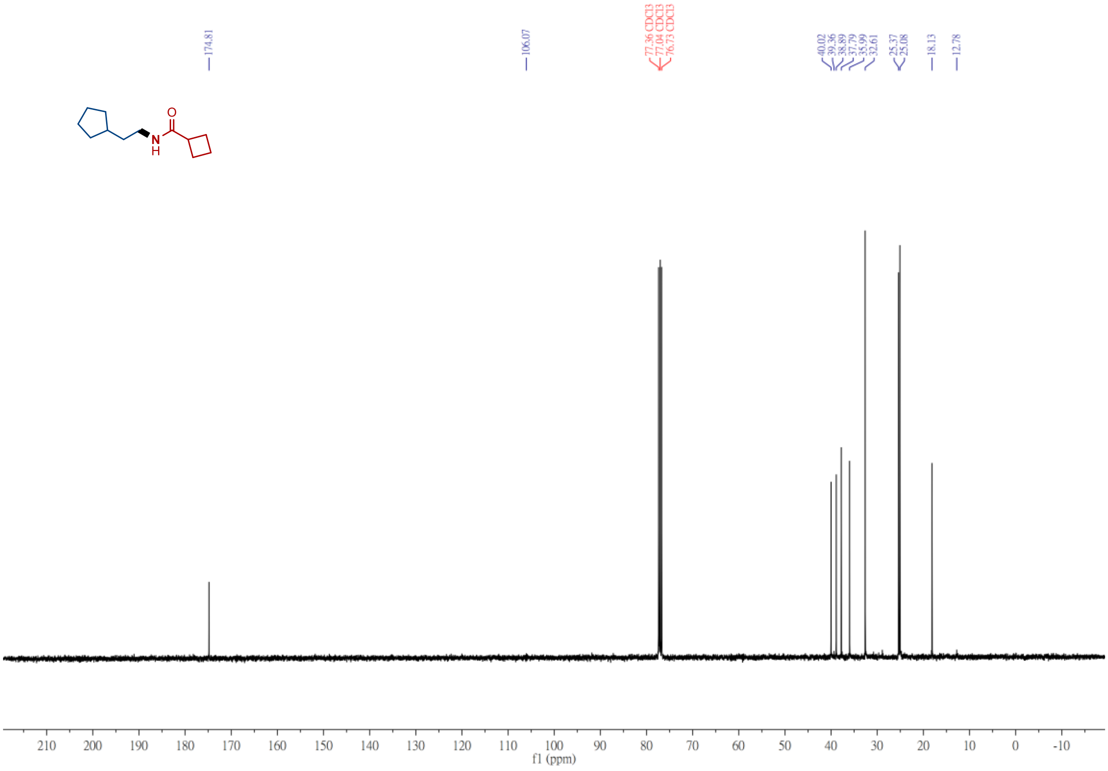

^{1}H and ^{13}C NMR spectrum of **51**

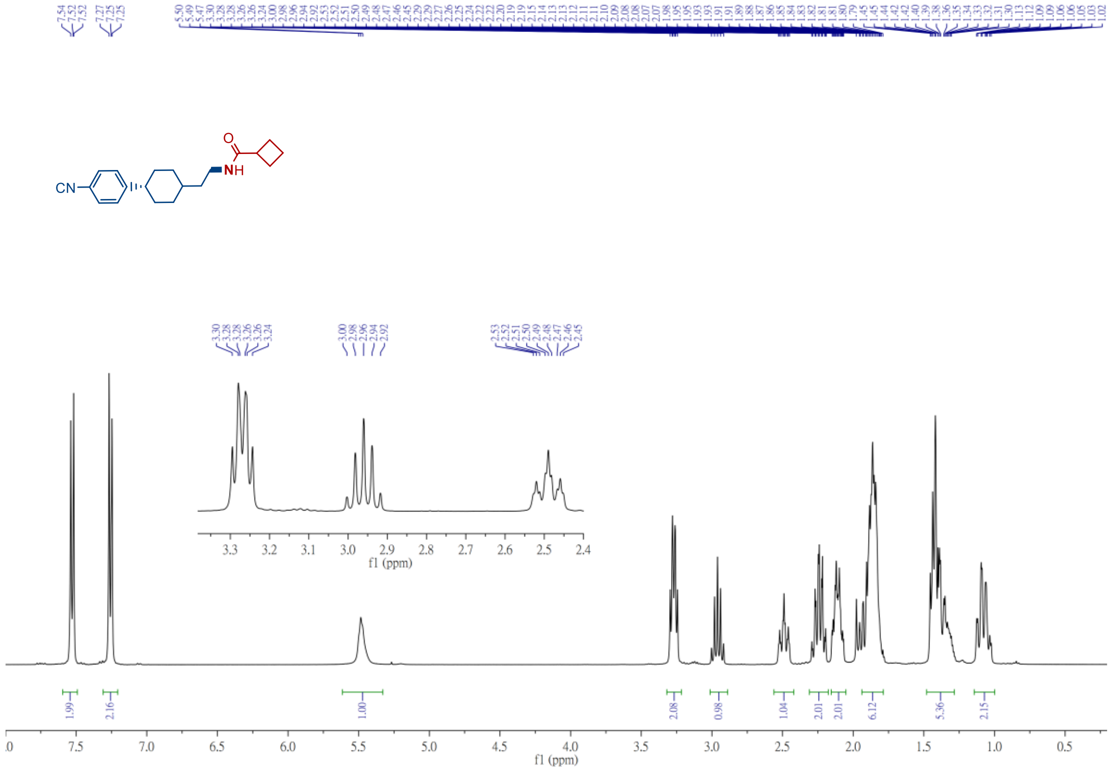

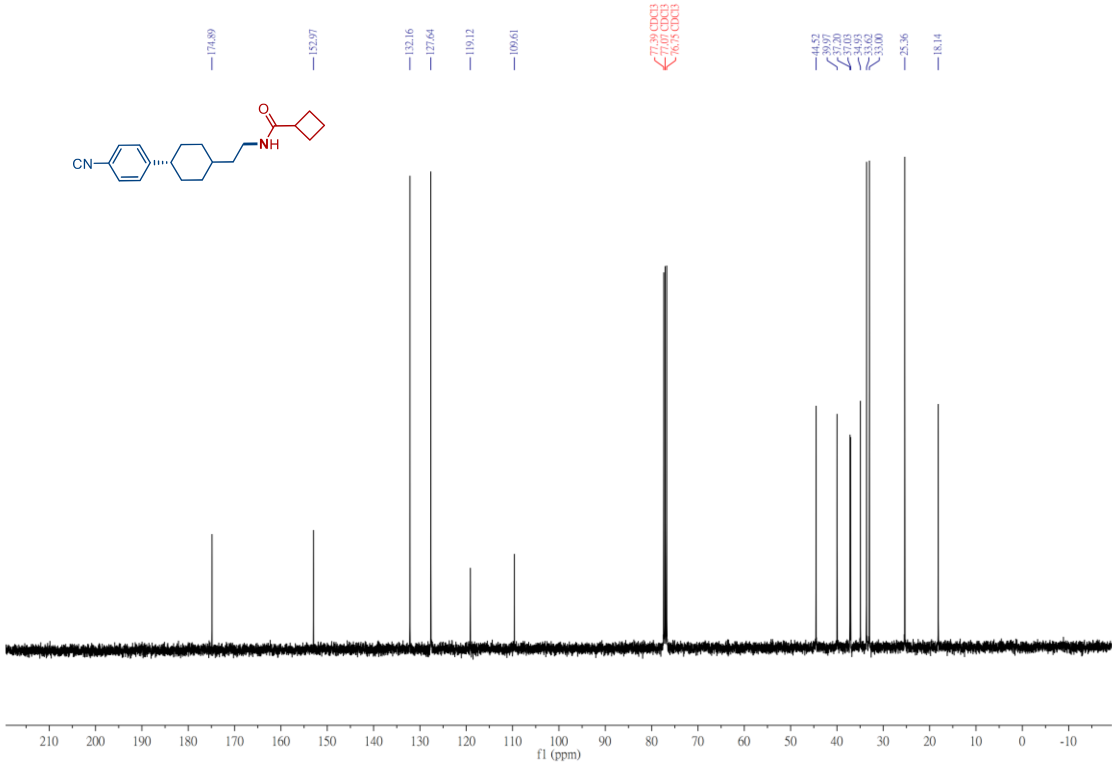

^{1}H and ^{13}C NMR spectrum of **52**

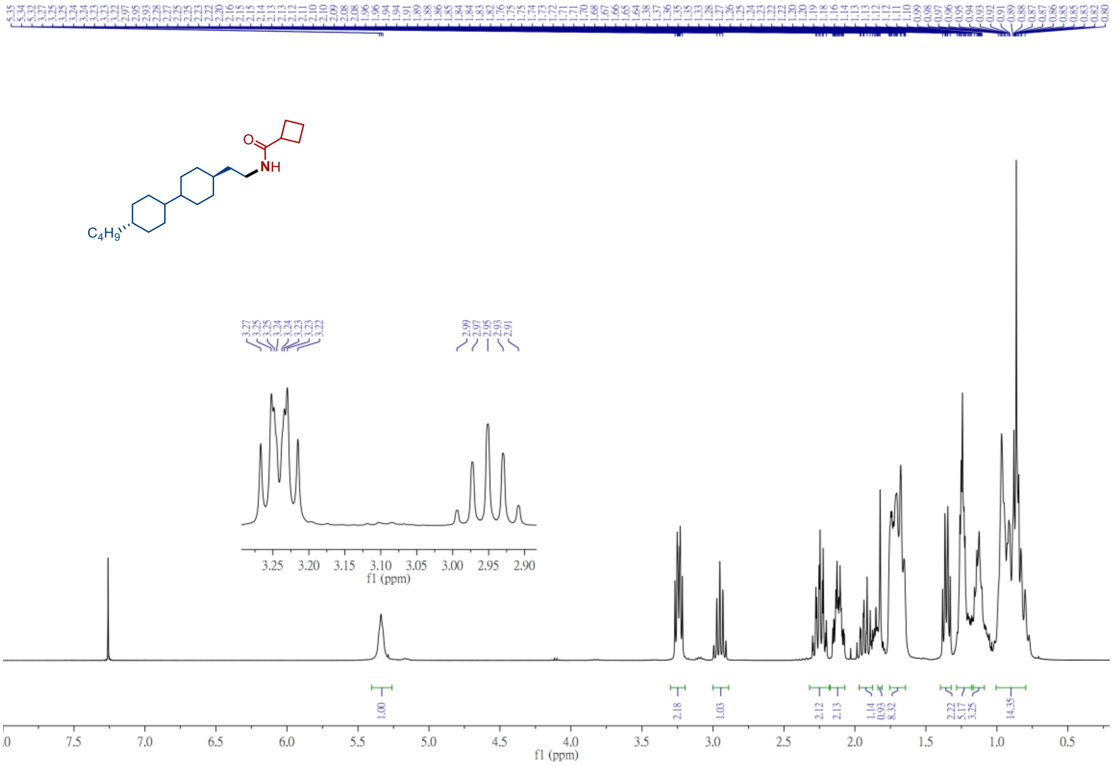

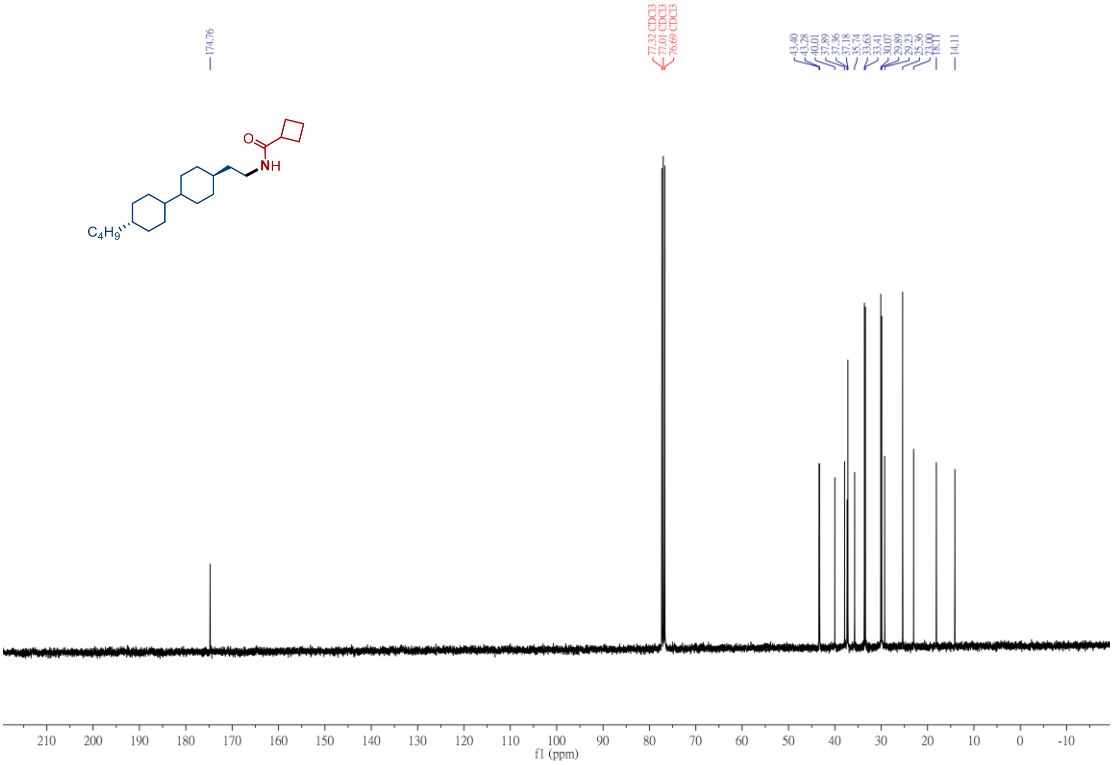

^{1}H and ^{13}C NMR spectrum of **53**

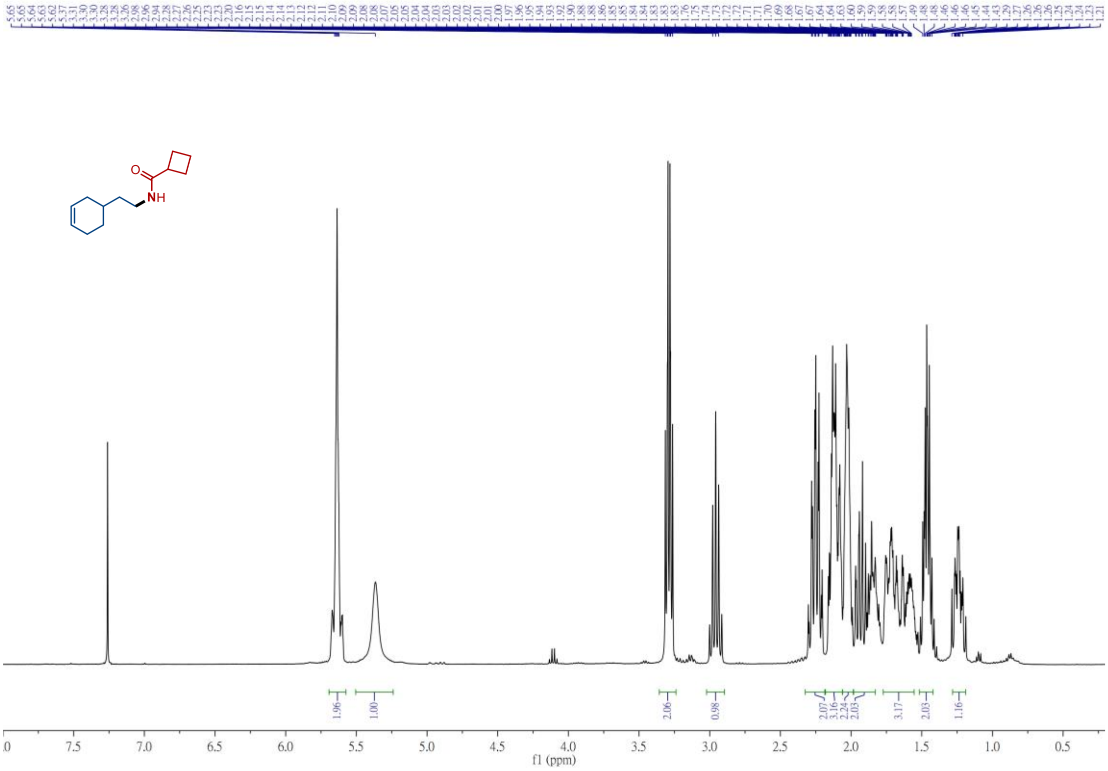

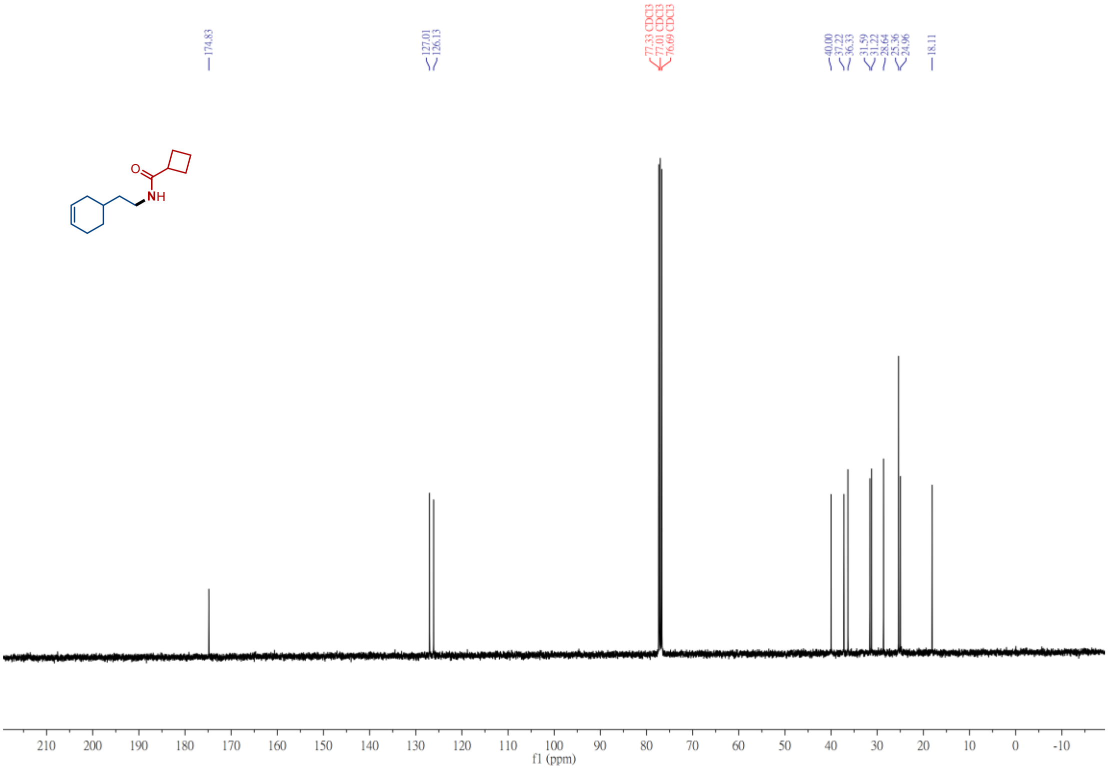

^{1}H spectrum of **54**

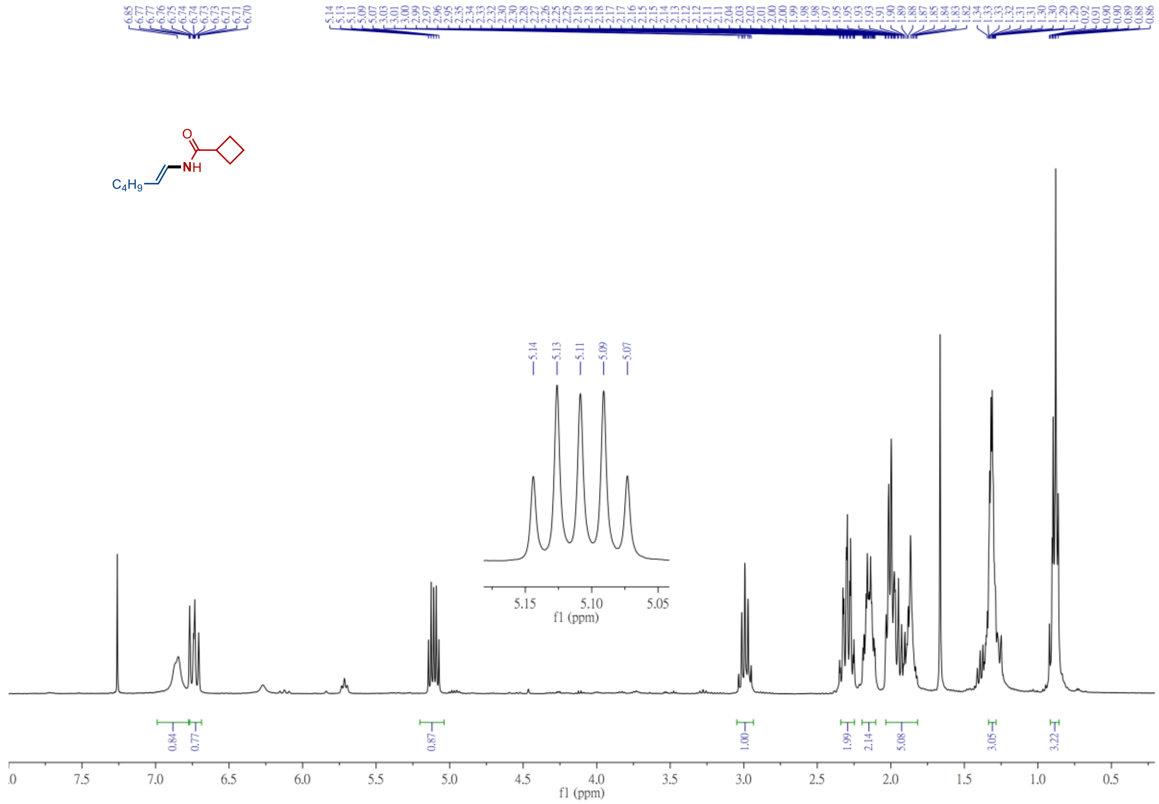

^{1}H and ^{13}C NMR spectrum of **55**

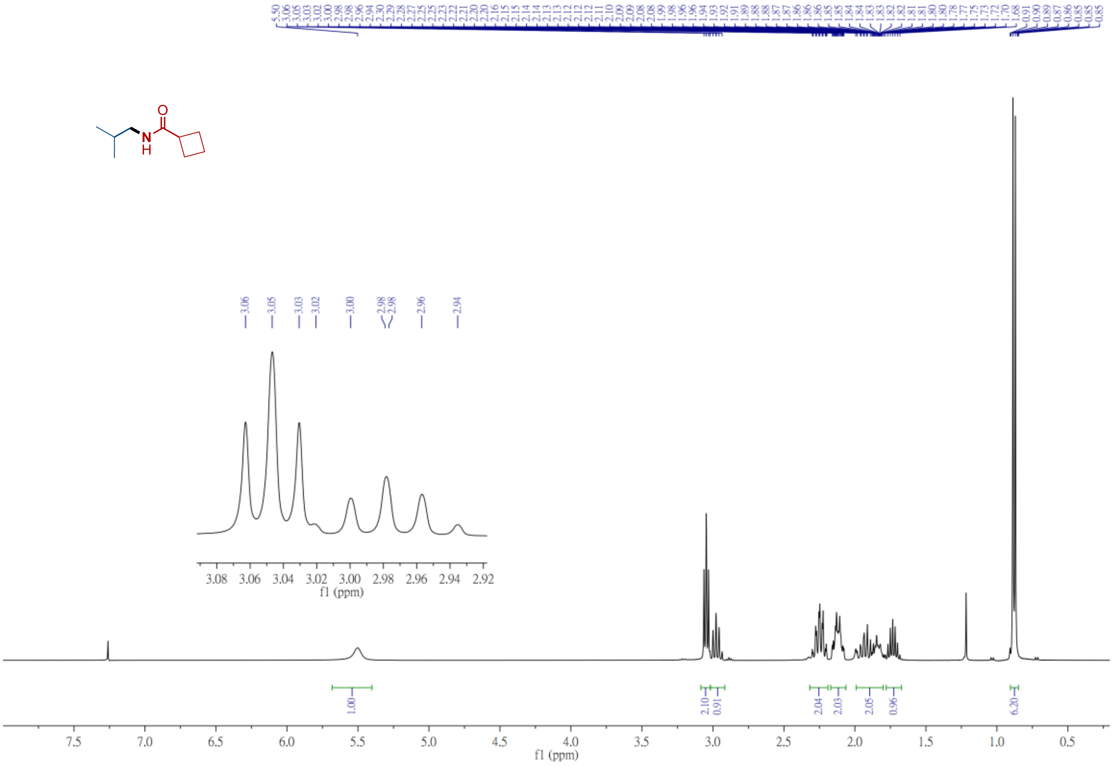

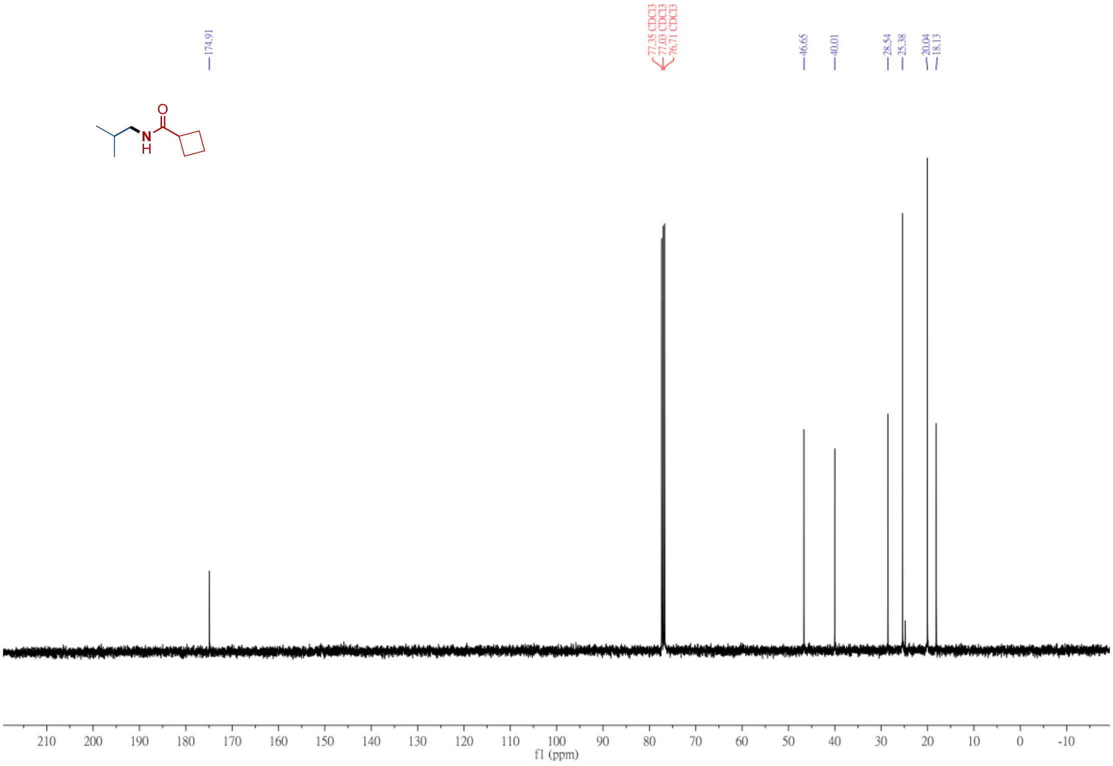

^{1}H and ^{13}C NMR spectrum of **56**

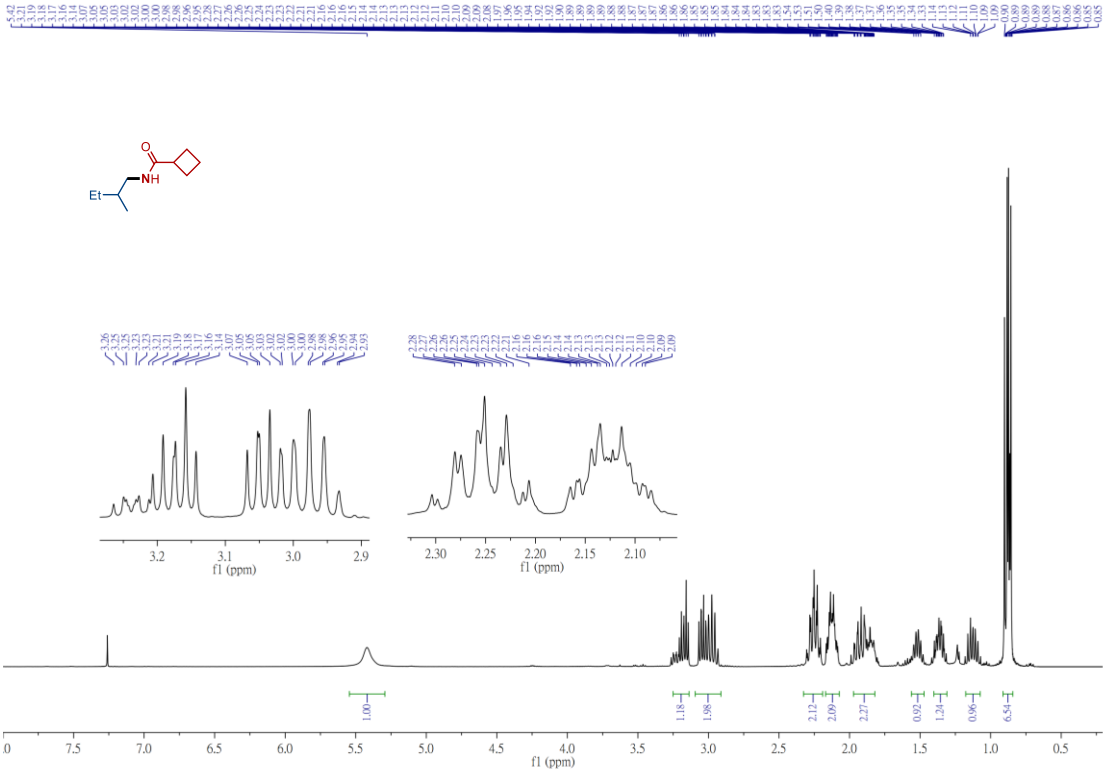

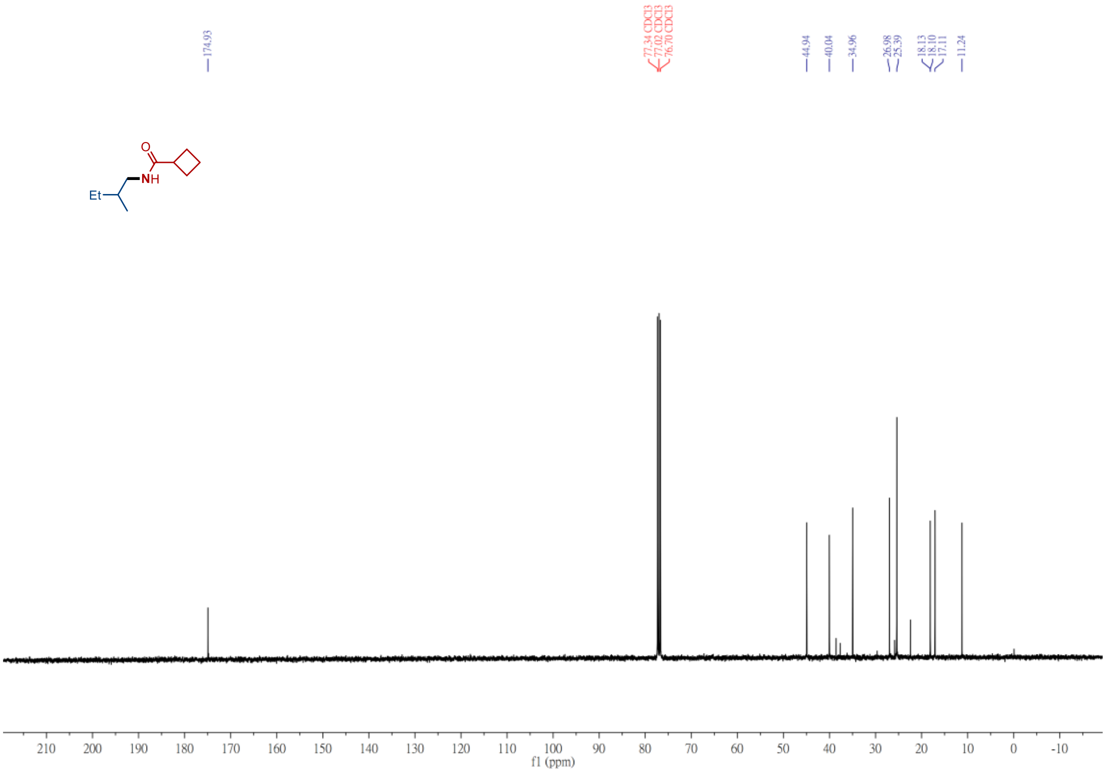

^{1}H and ^{13}C NMR spectrum of **57**

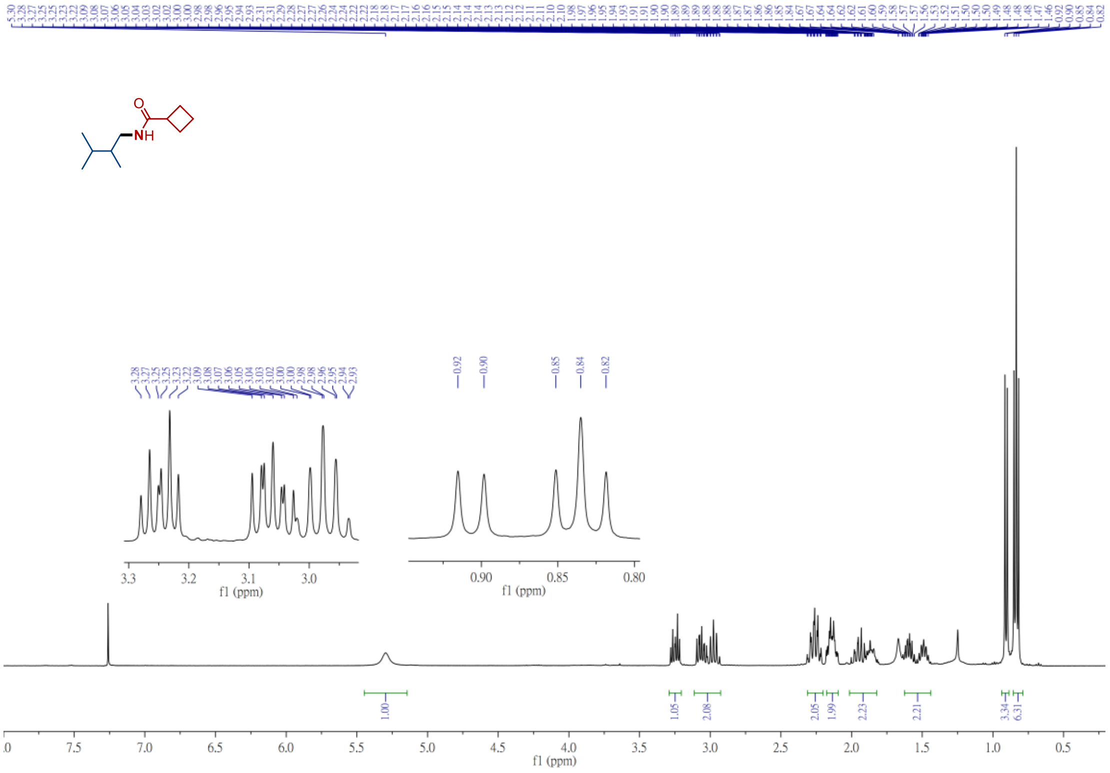

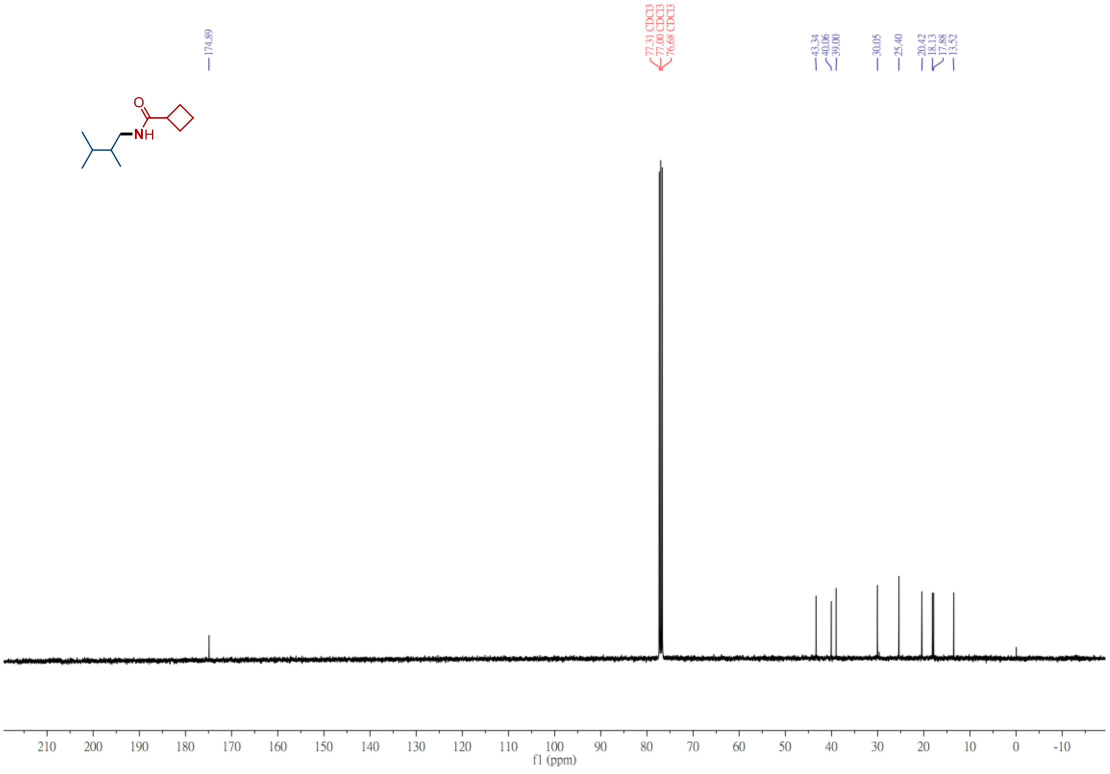

^{1}H and ^{13}C NMR spectrum of **58**

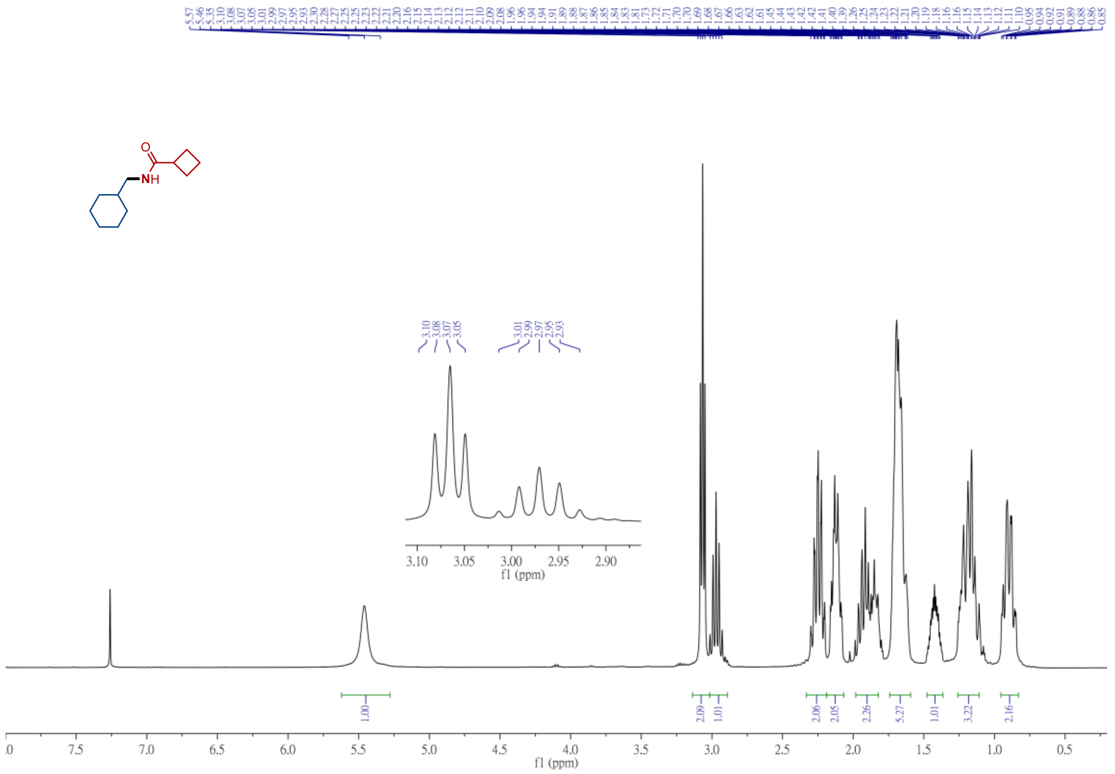

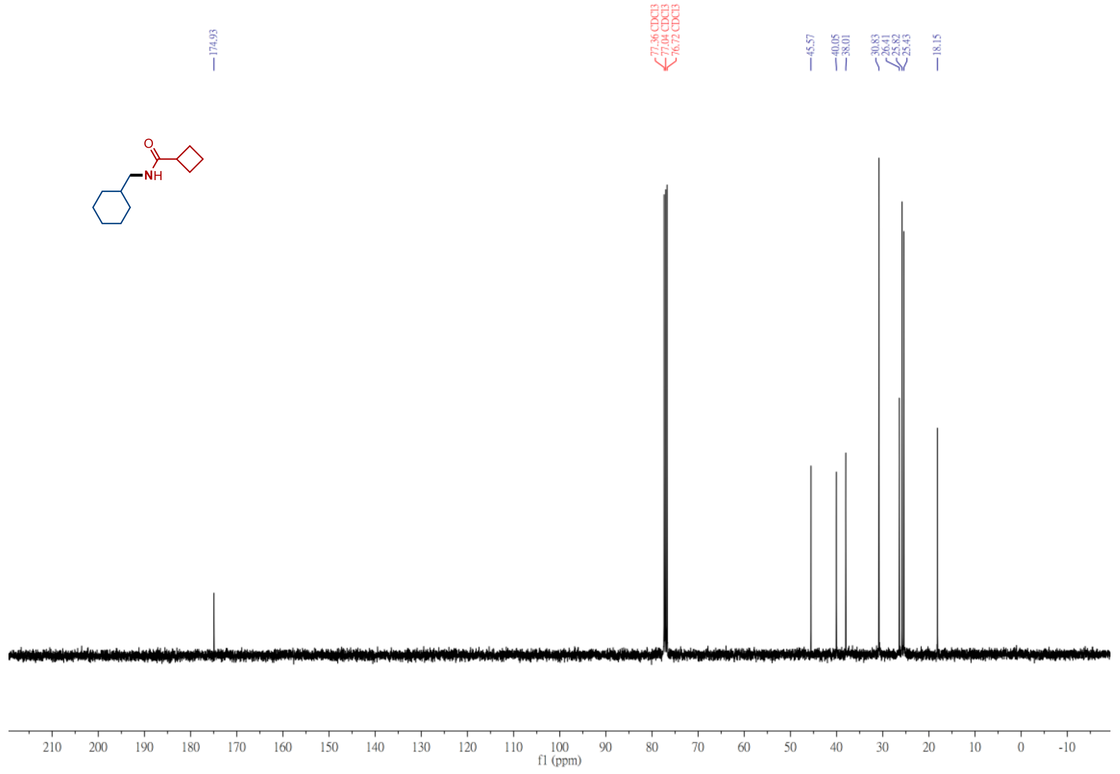

^{1}H and ^{13}C NMR spectrum of **59**

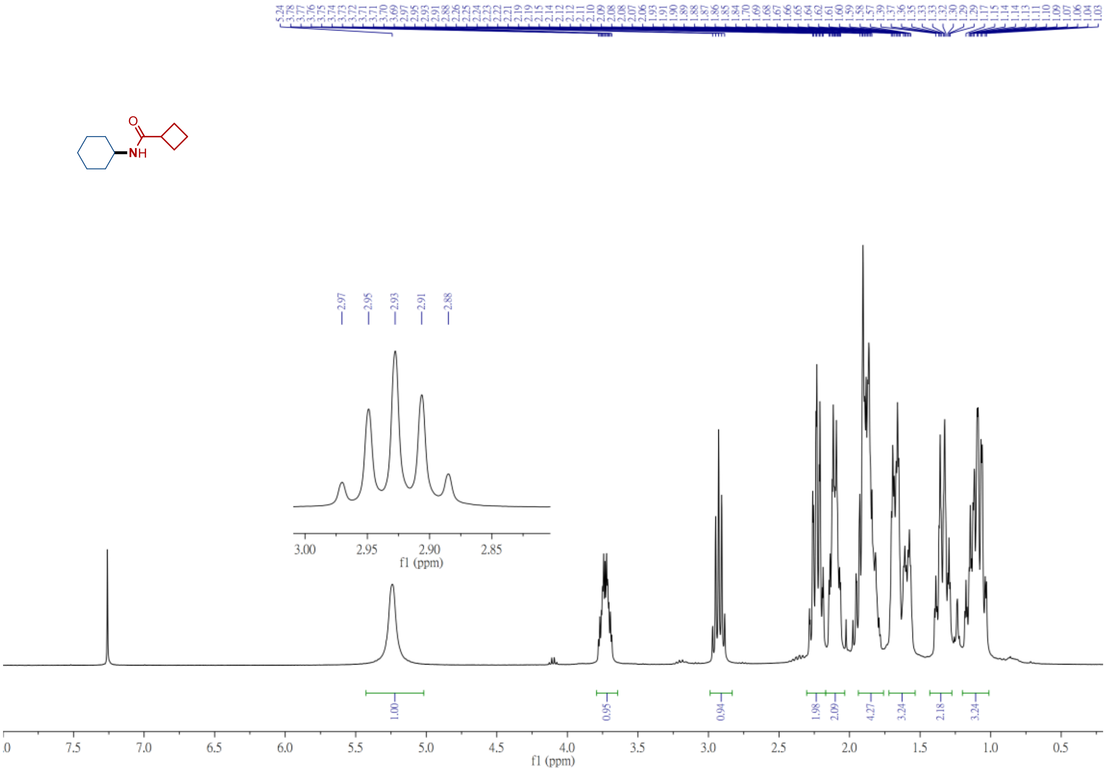

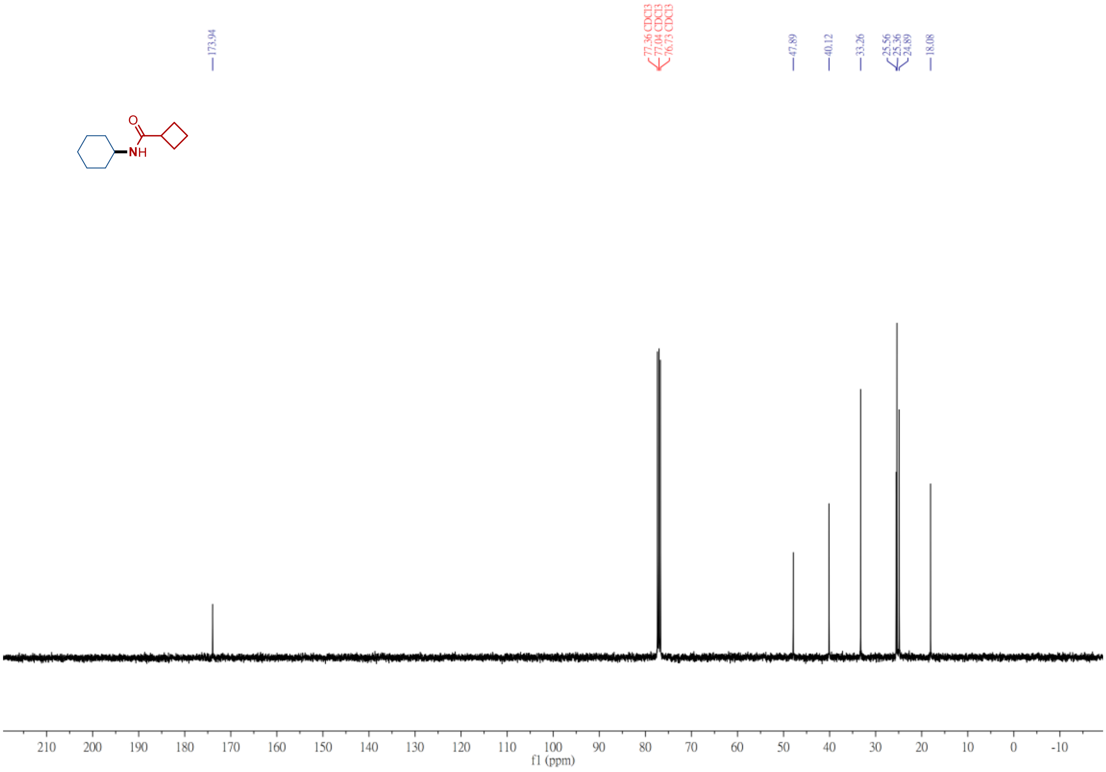

^{1}H and ^{13}C NMR spectrum of **60**

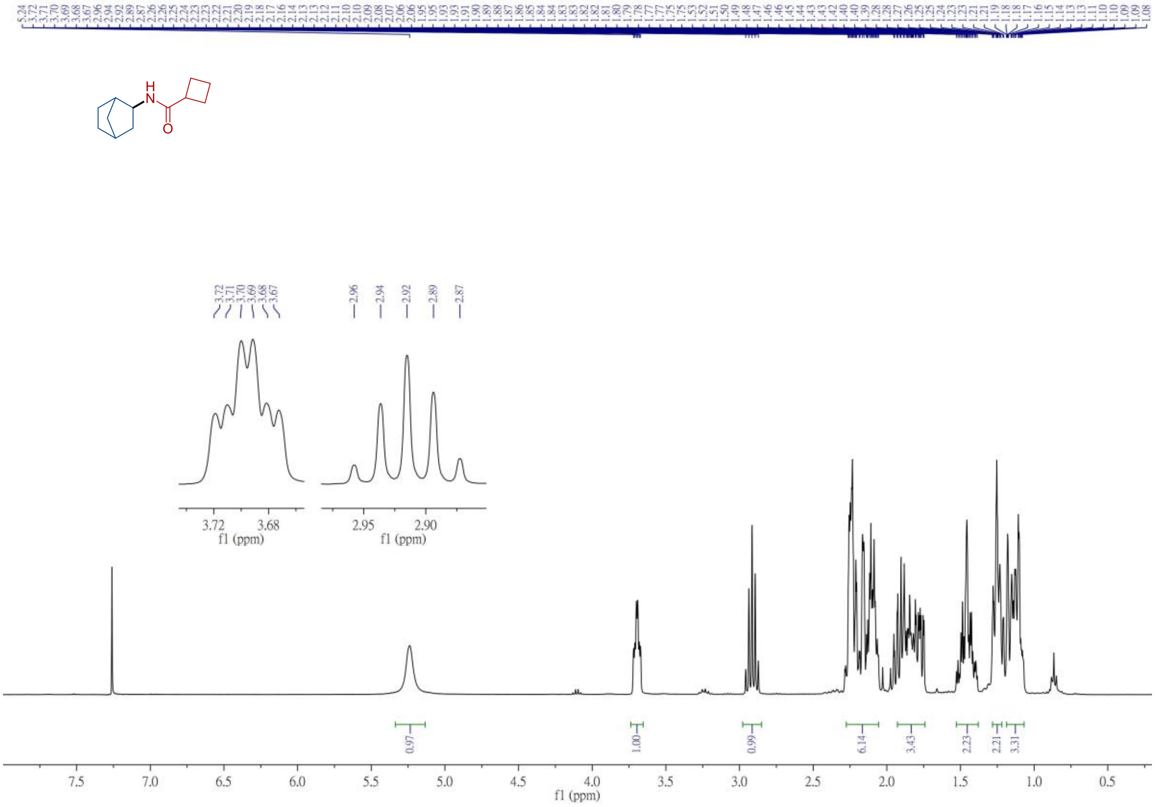

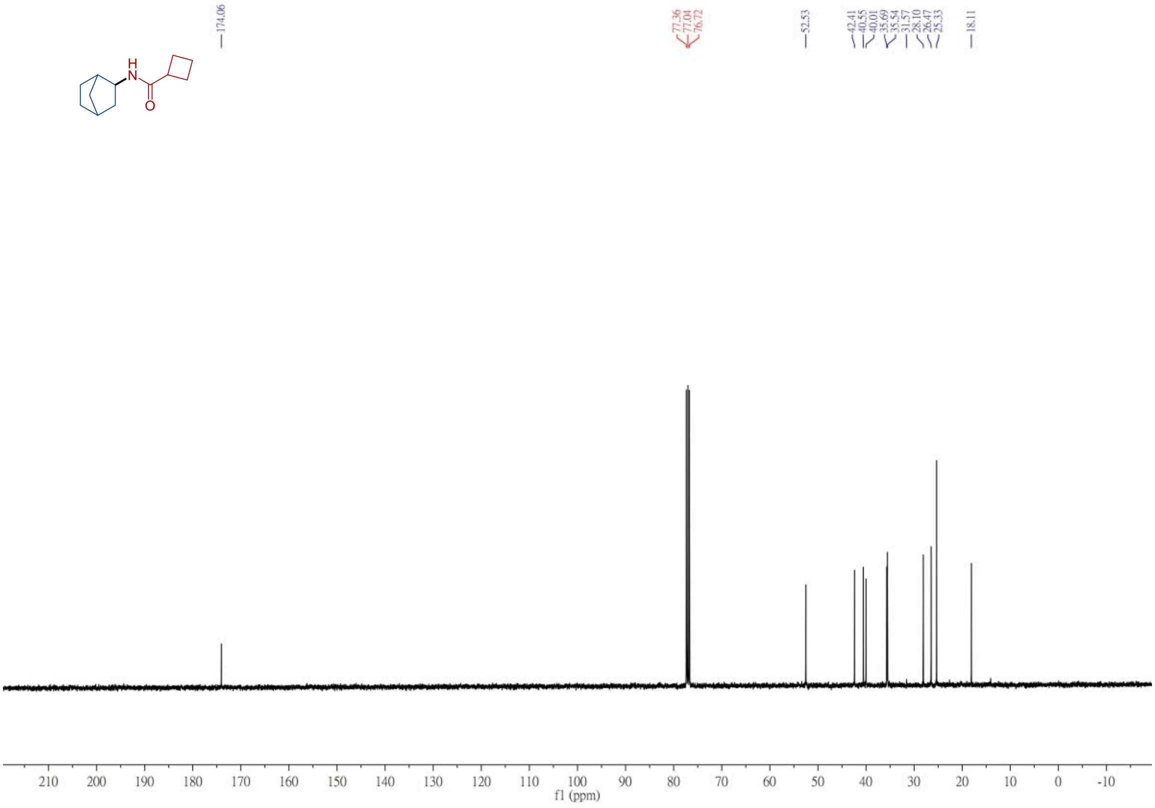

^{1}H and ^{13}C NMR spectrum of **61**

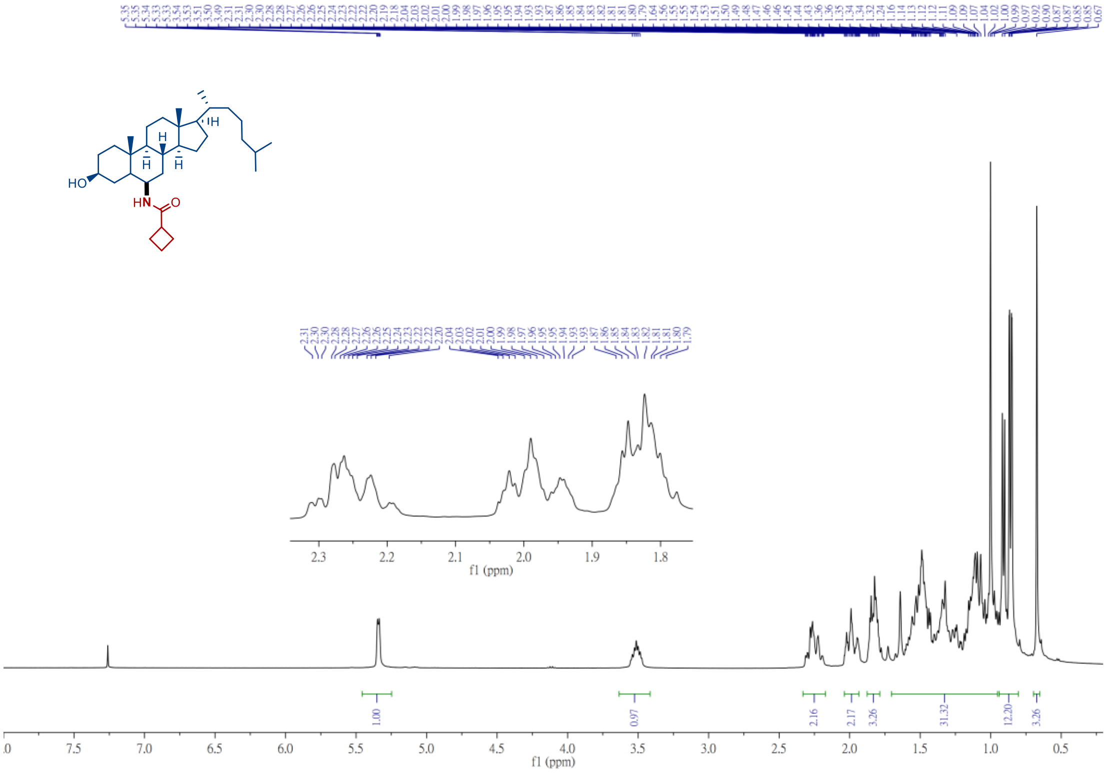

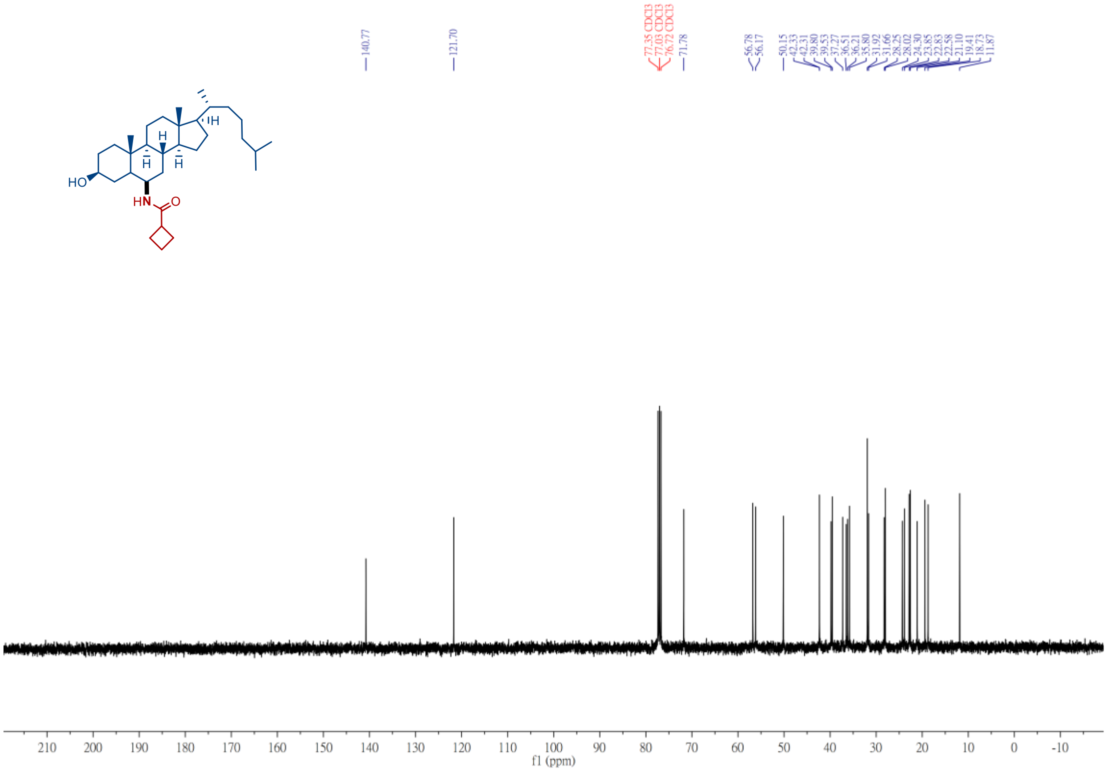

^{1}H and ^{13}C NMR spectrum of **63**

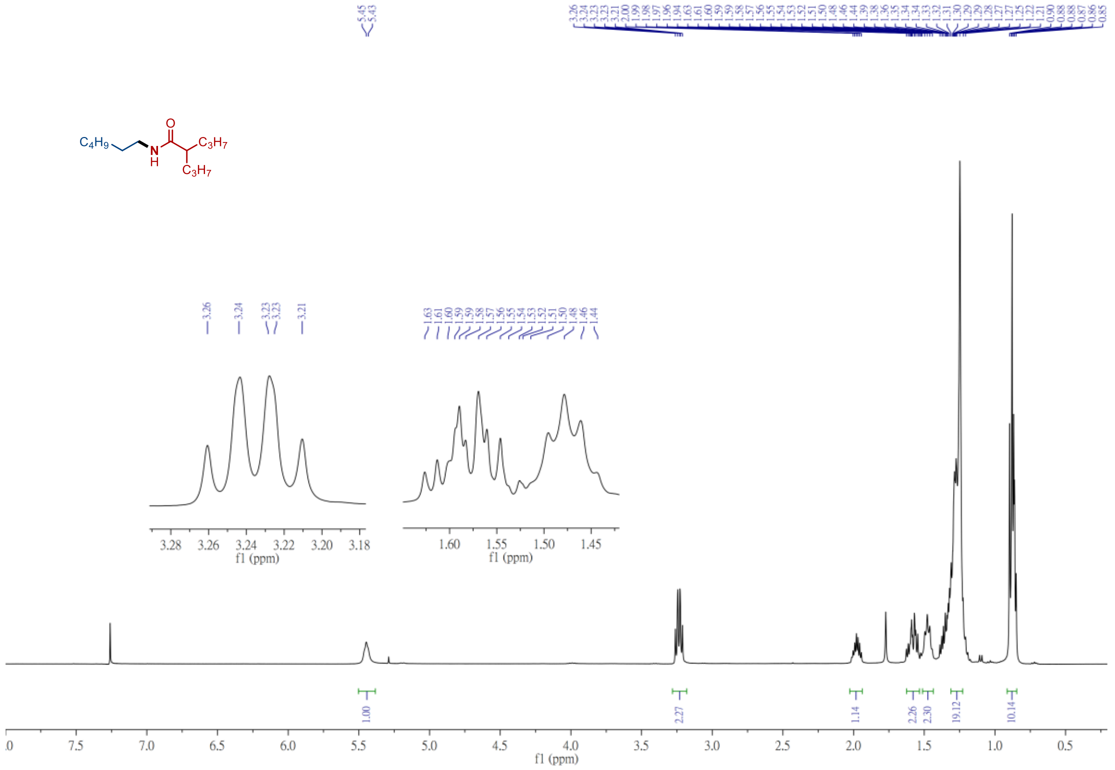

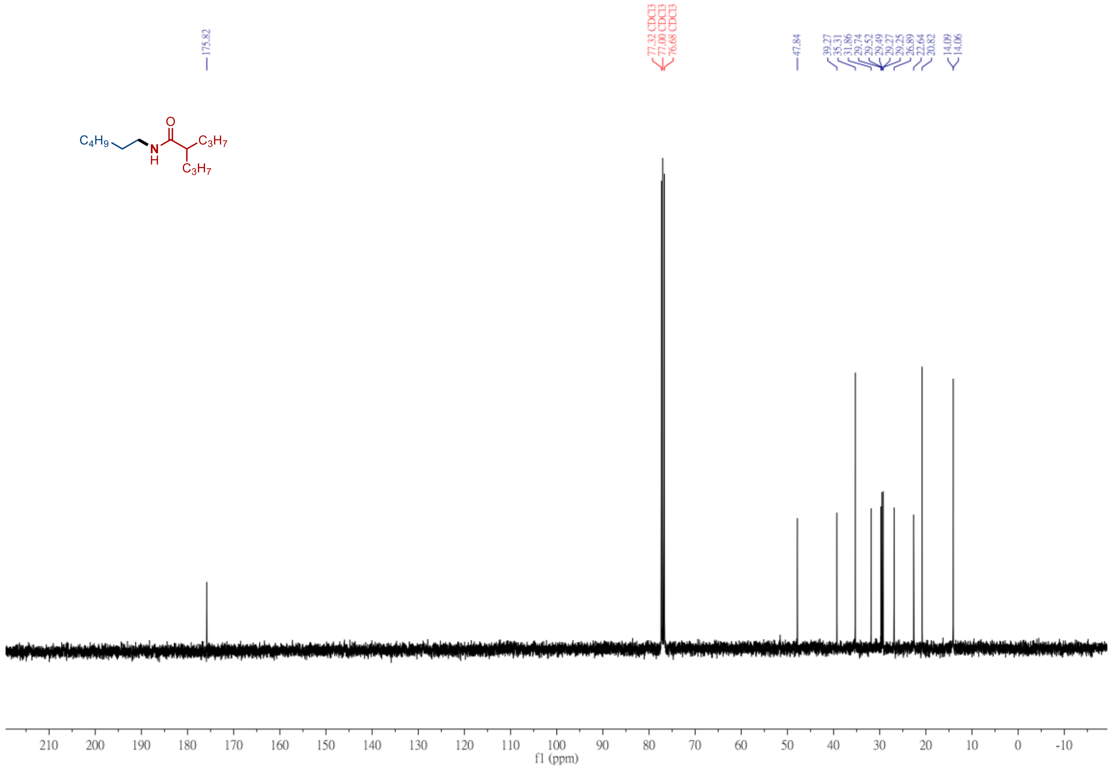

^{1}H and ^{13}C NMR spectrum of **64**

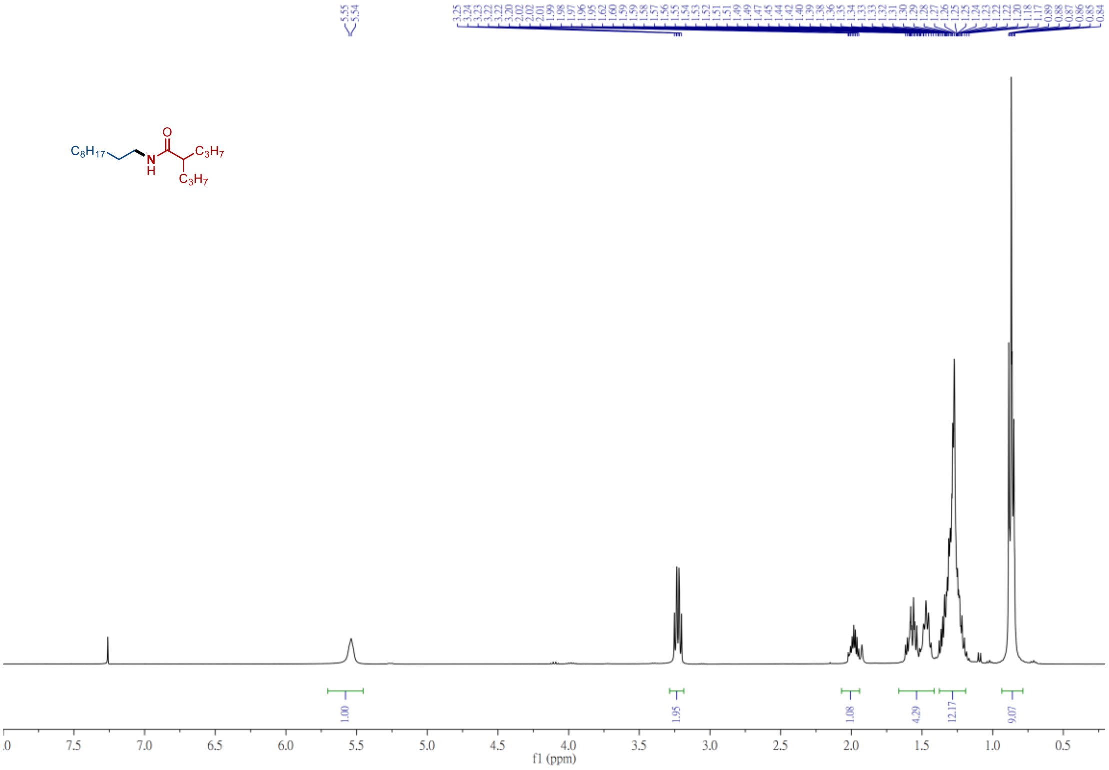

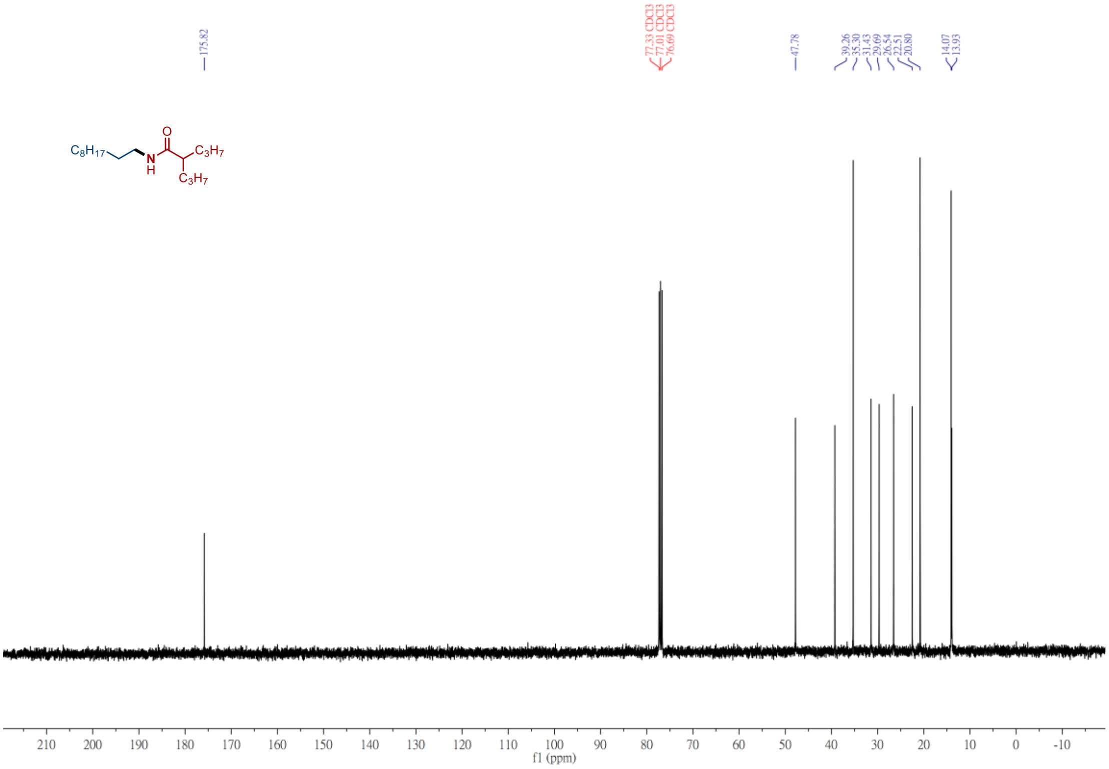

^1H and ^{13}C NMR spectrum of **65**

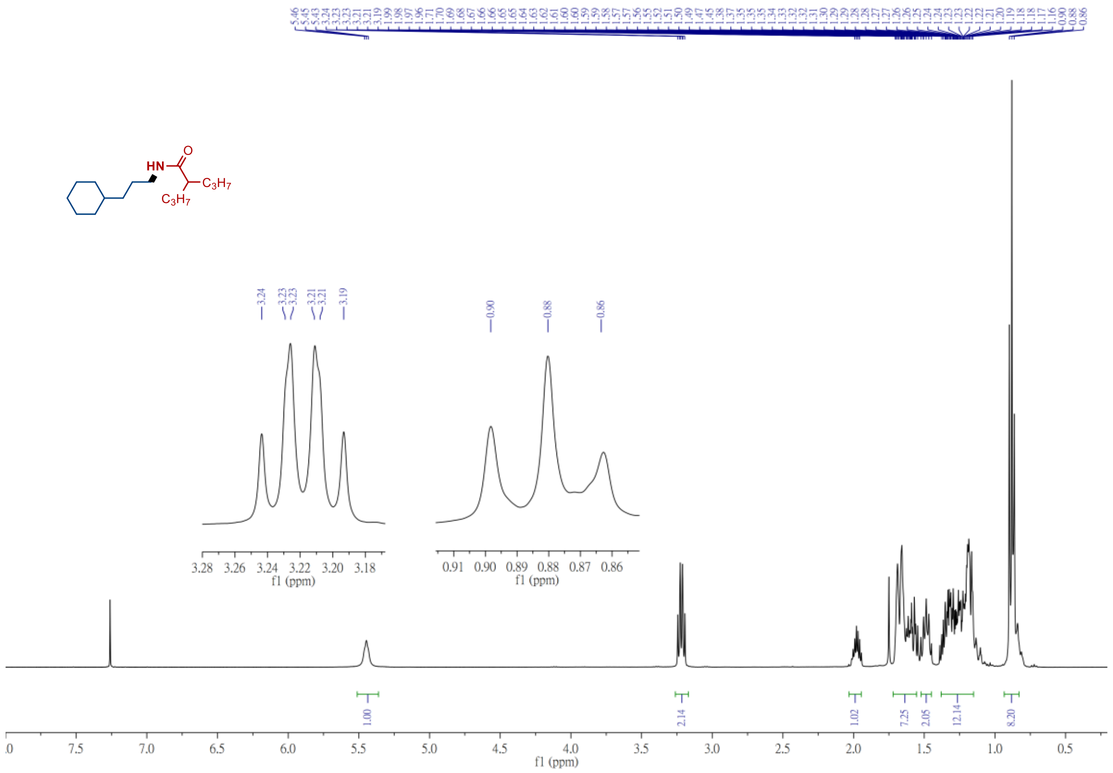

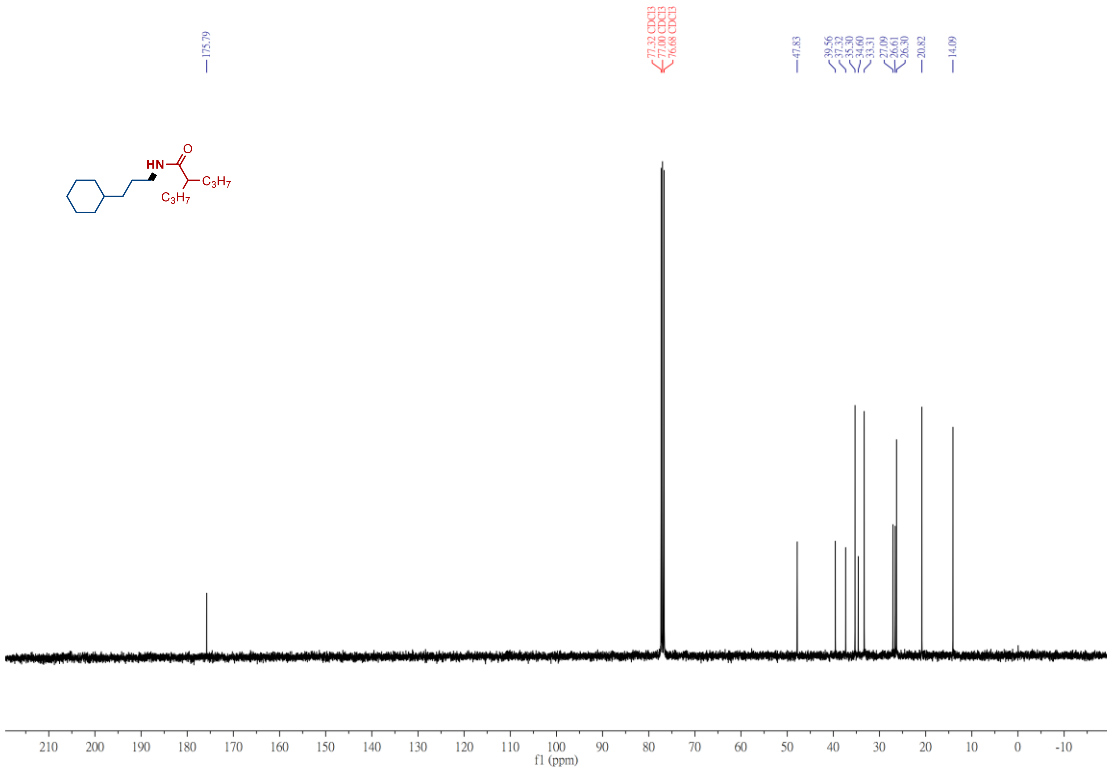

^{1}H and ^{13}C NMR spectrum of **66**

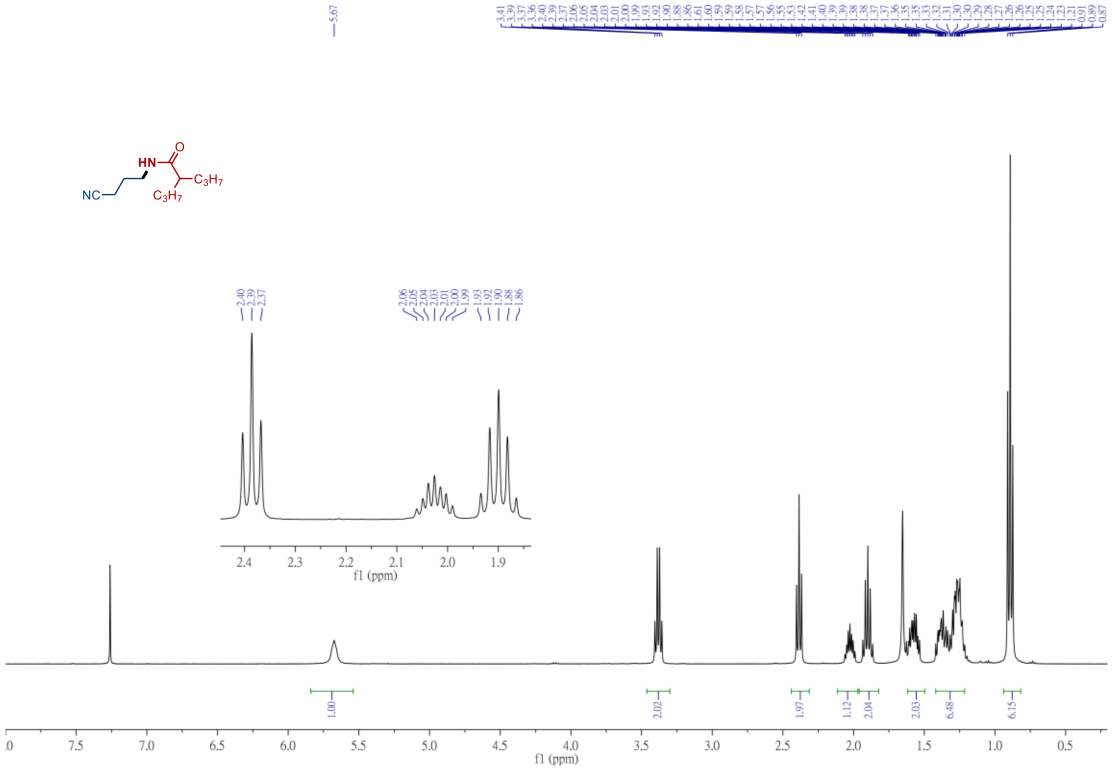

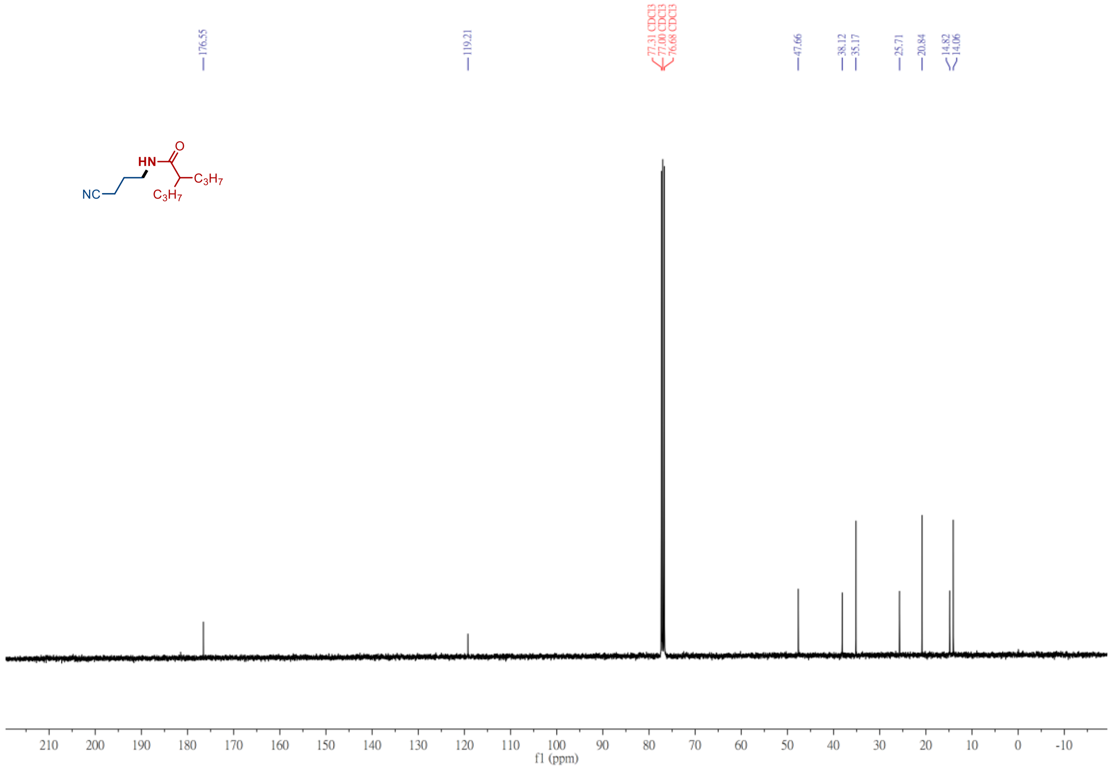

^{1}H and ^{13}C NMR spectrum of **67**

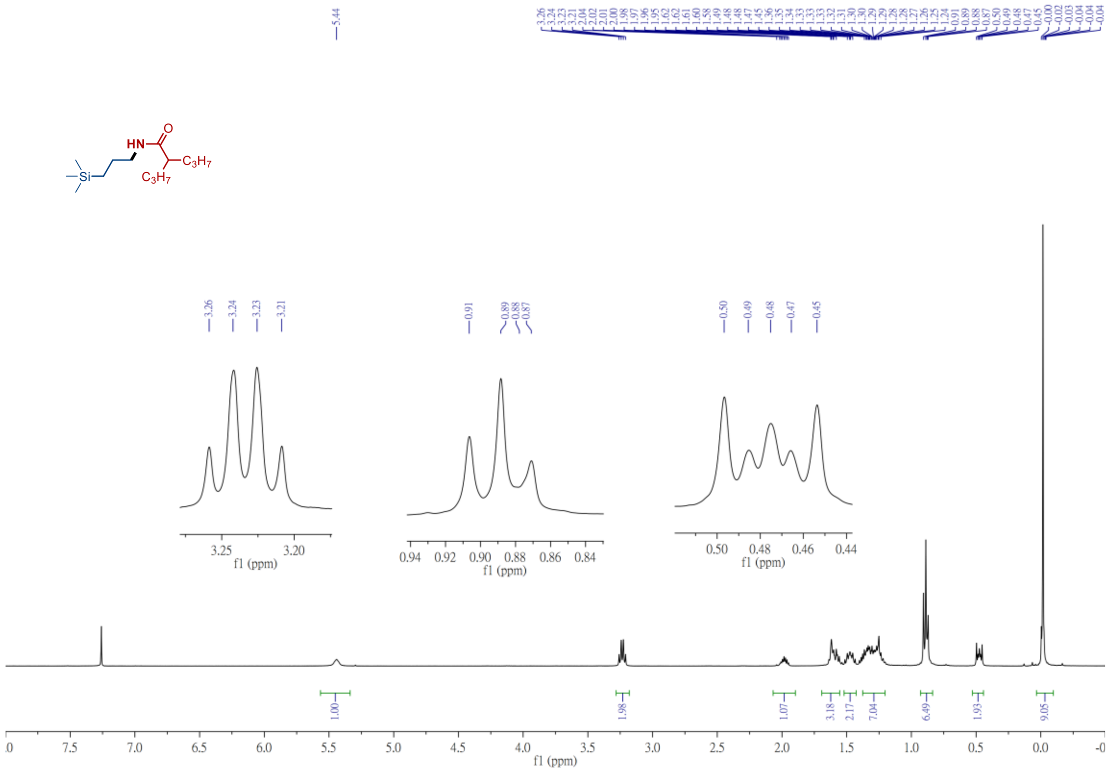

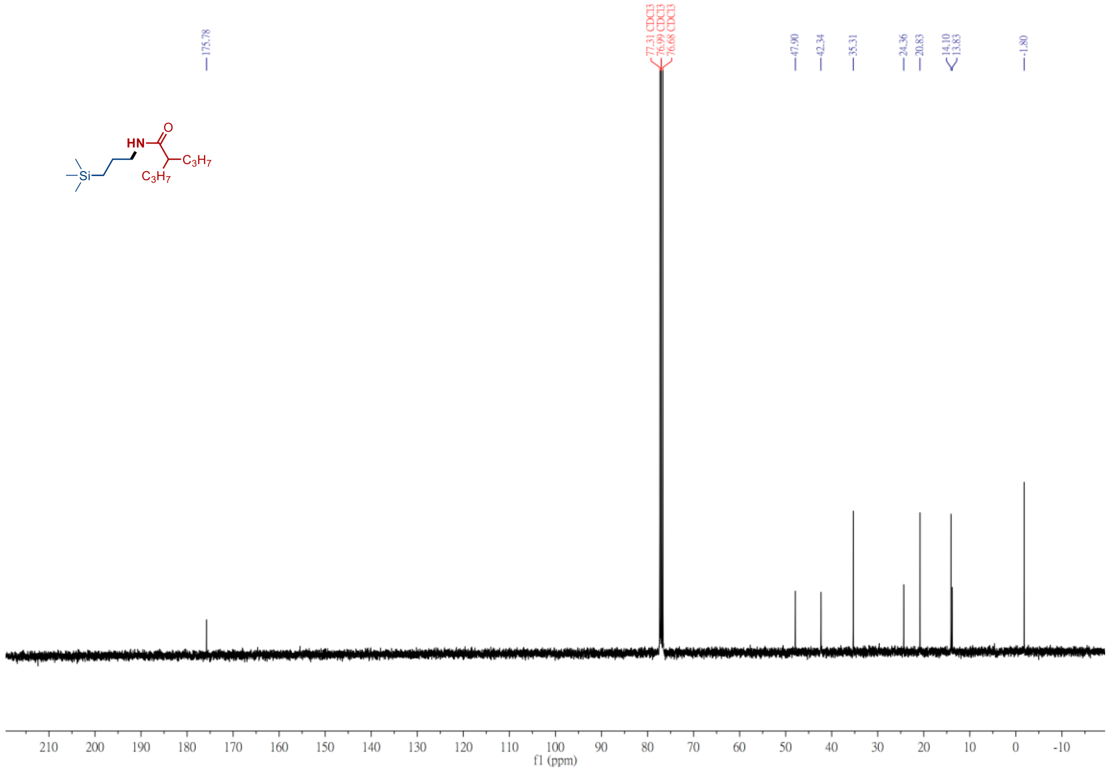

^{1}H and ^{13}C NMR spectrum of **68**

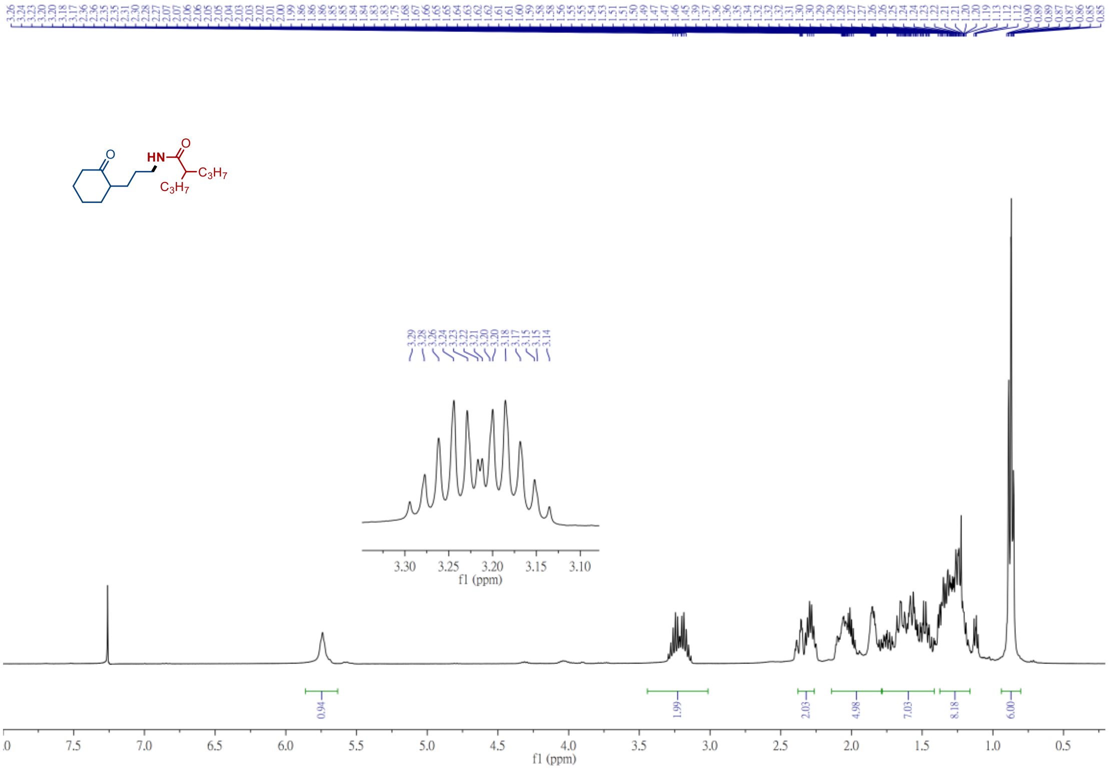

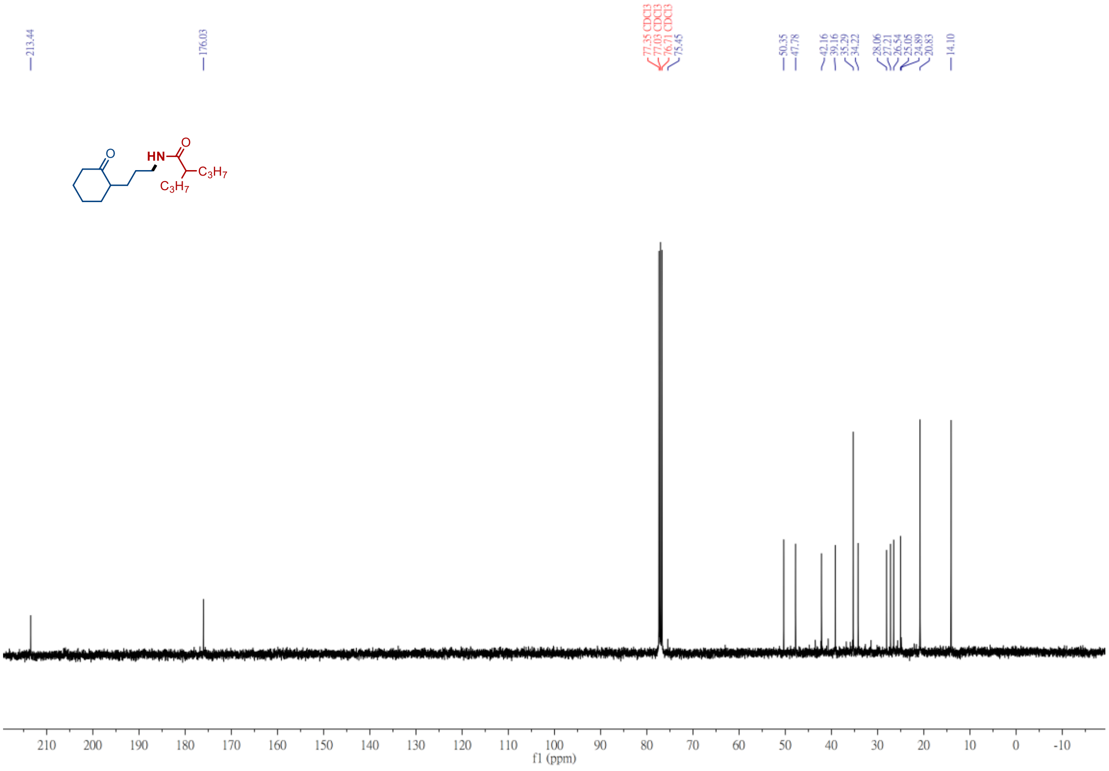

^{1}H and ^{13}C NMR spectrum of **69**

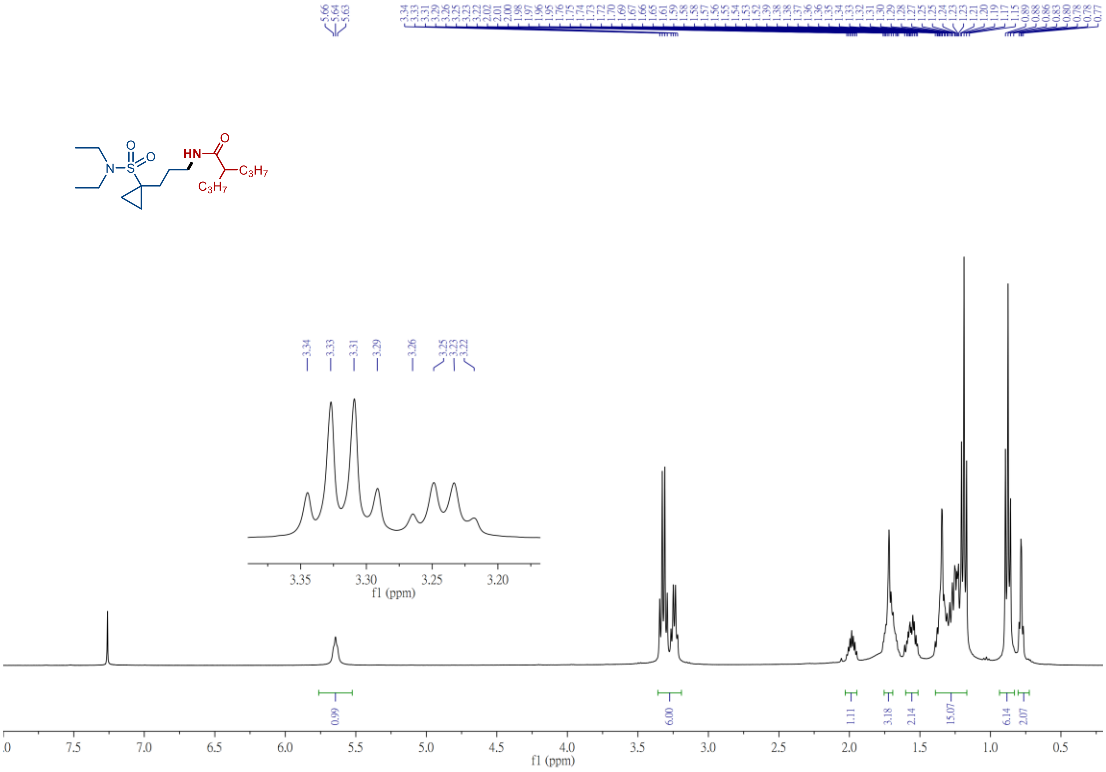

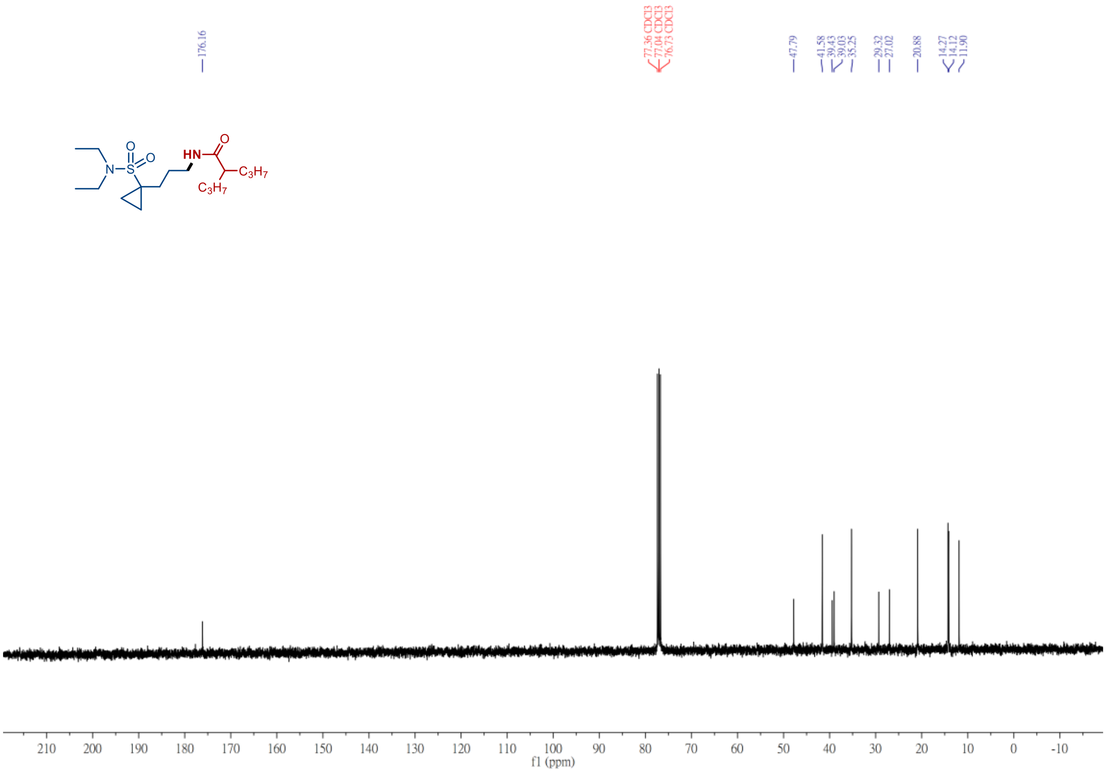

^{1}H and ^{13}C NMR spectrum of **70**

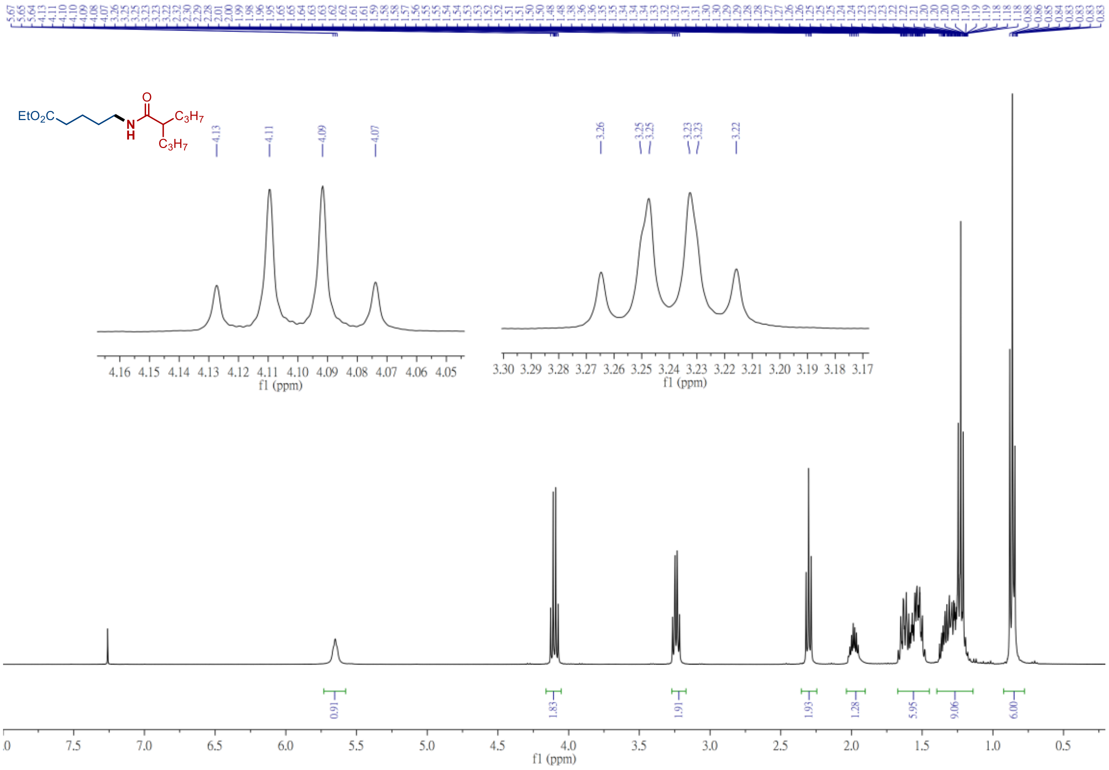

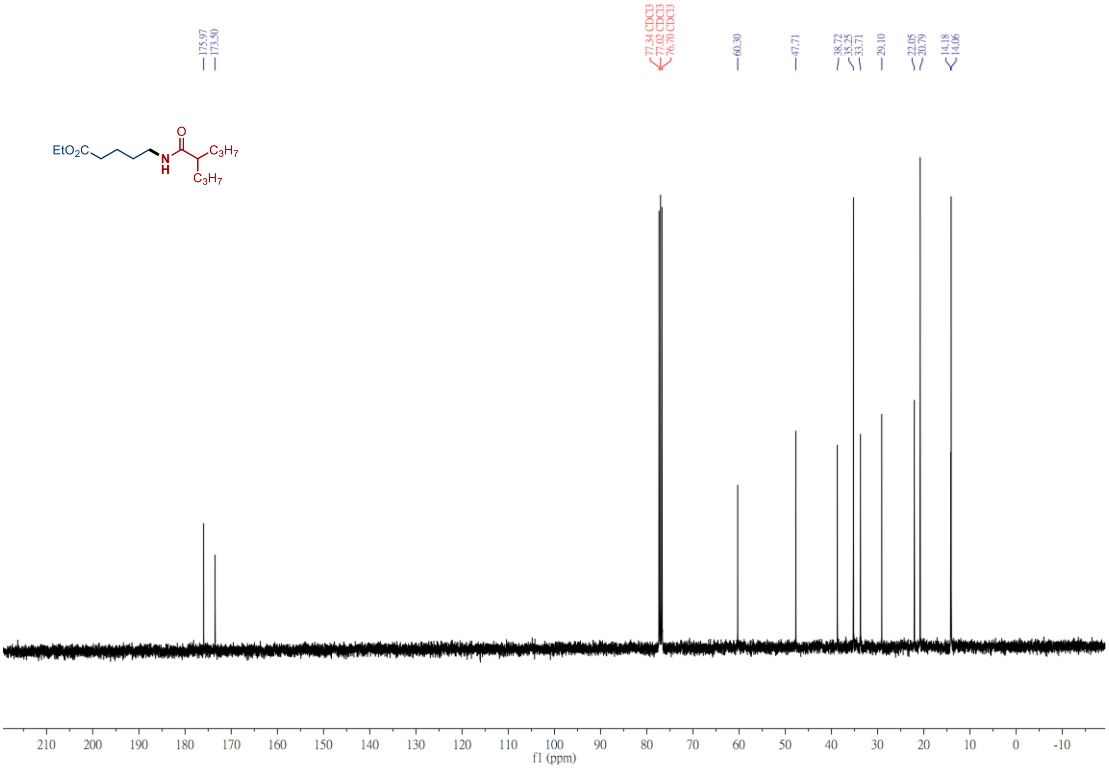

^{1}H and ^{13}C NMR spectrum of **71**

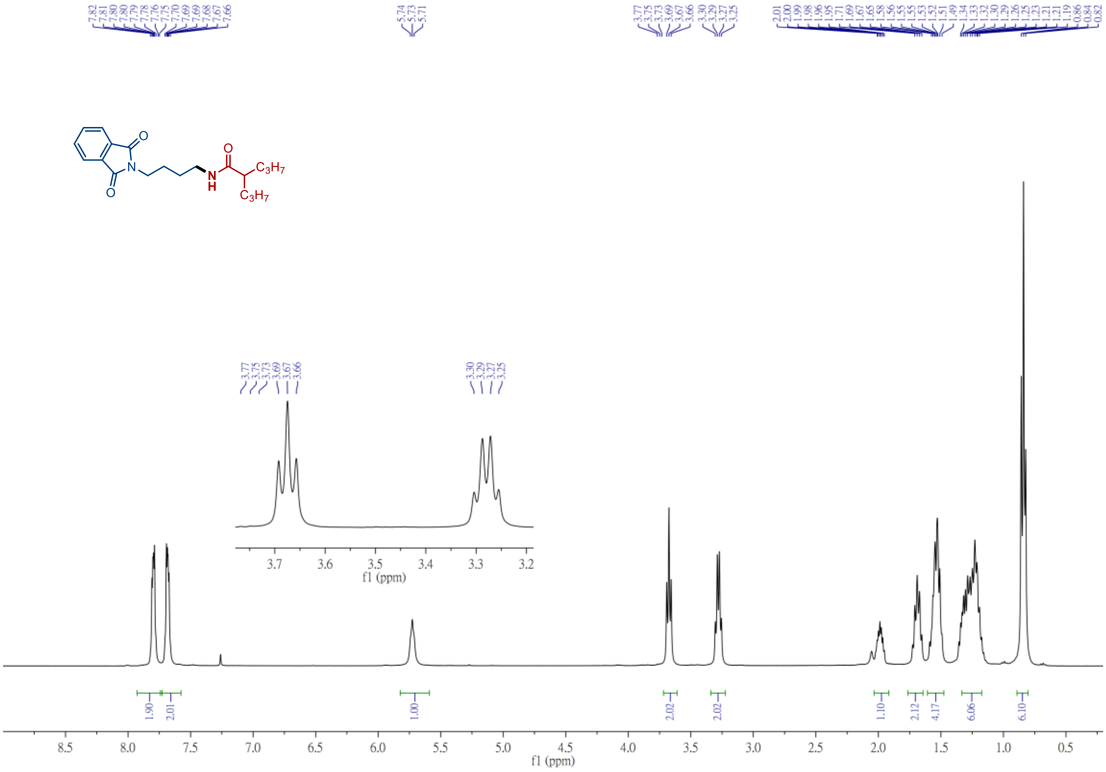

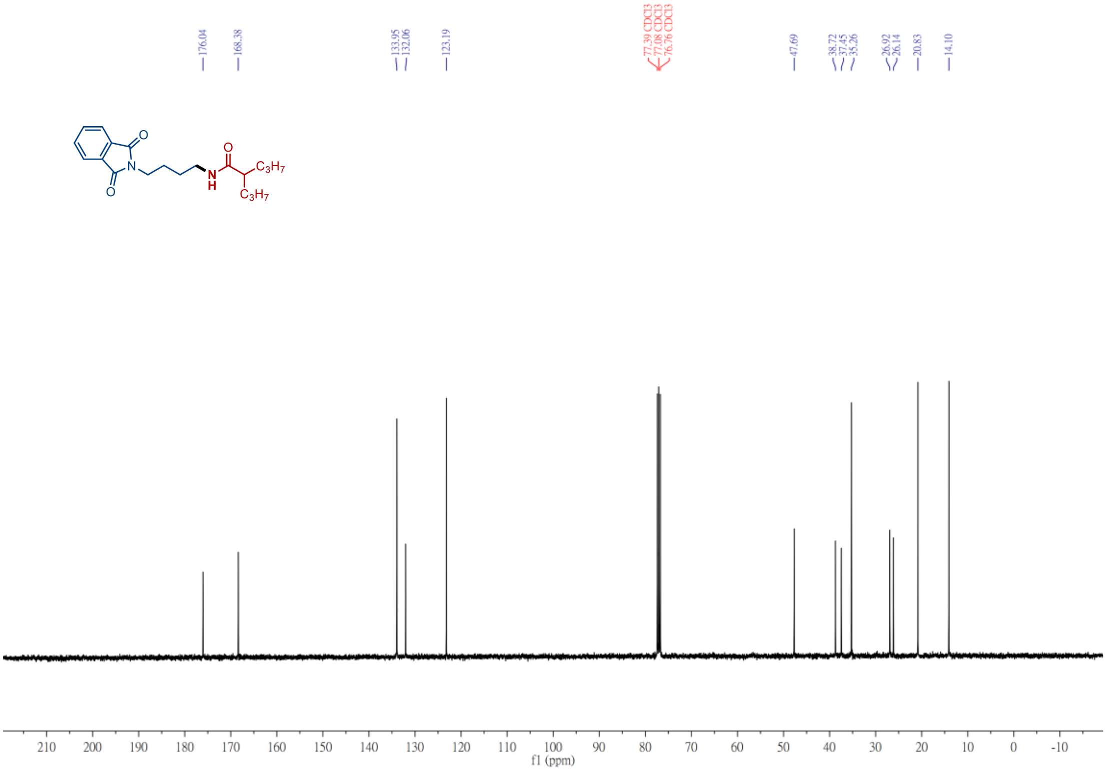

^{1}H and ^{13}C NMR spectrum of **72**

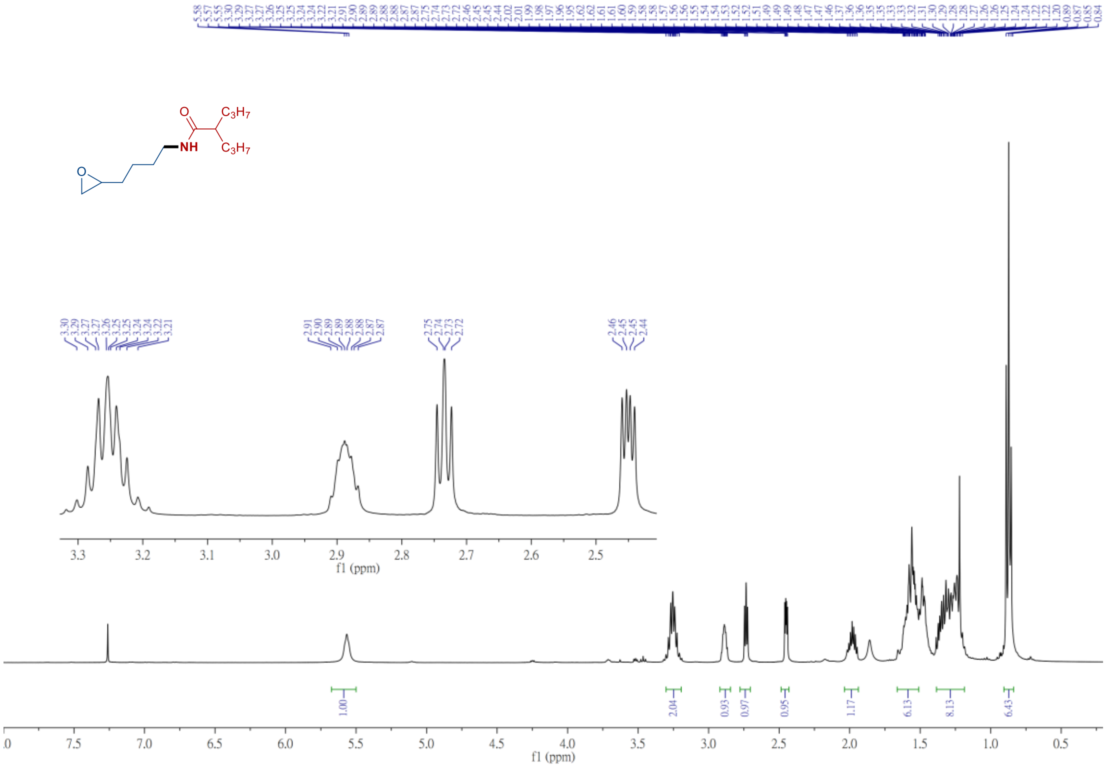

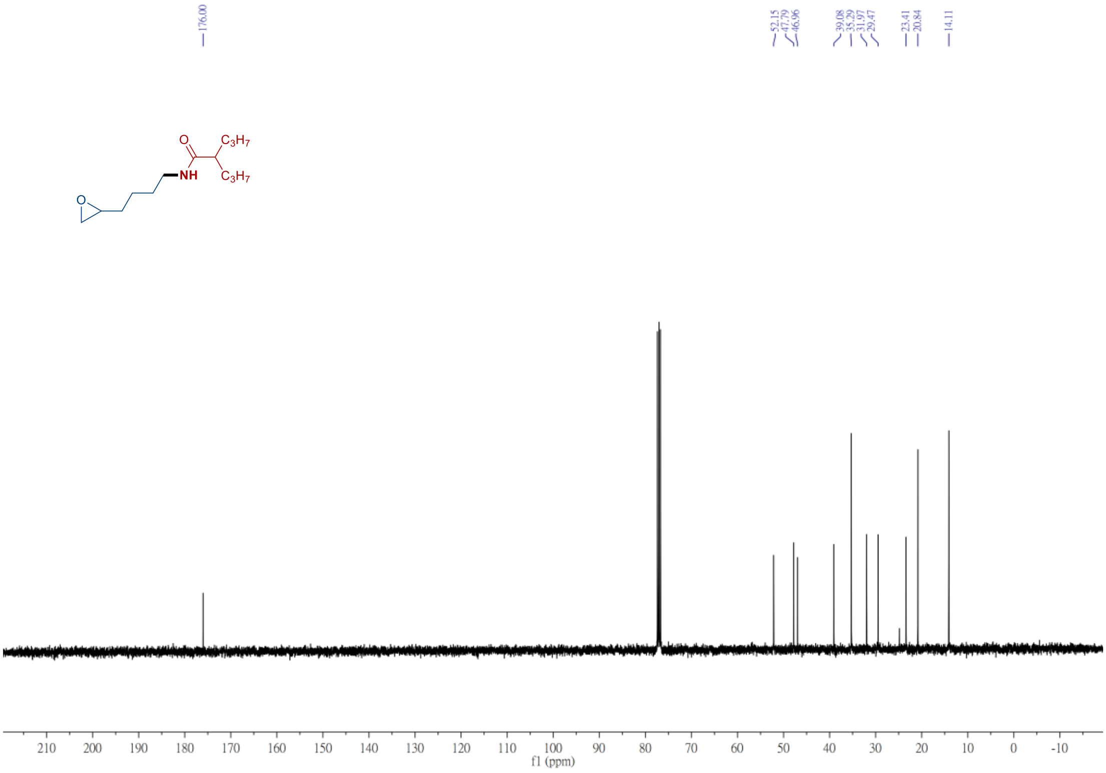

^{1}H and ^{13}C NMR spectrum of **73**

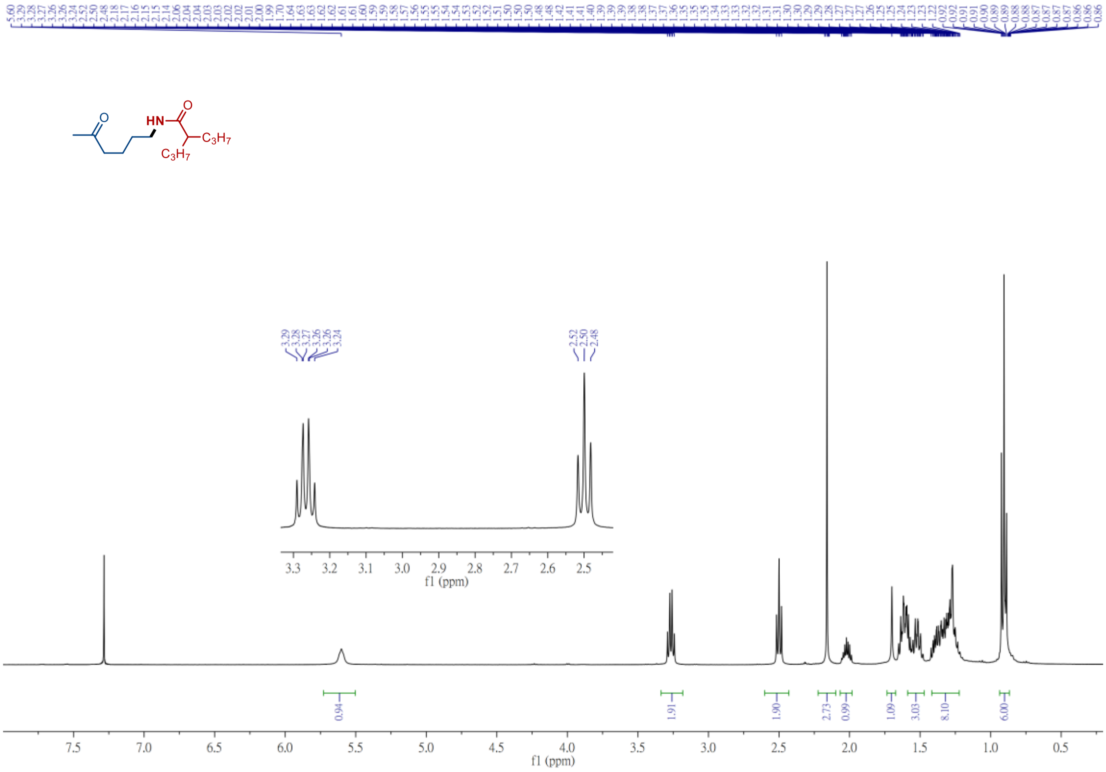

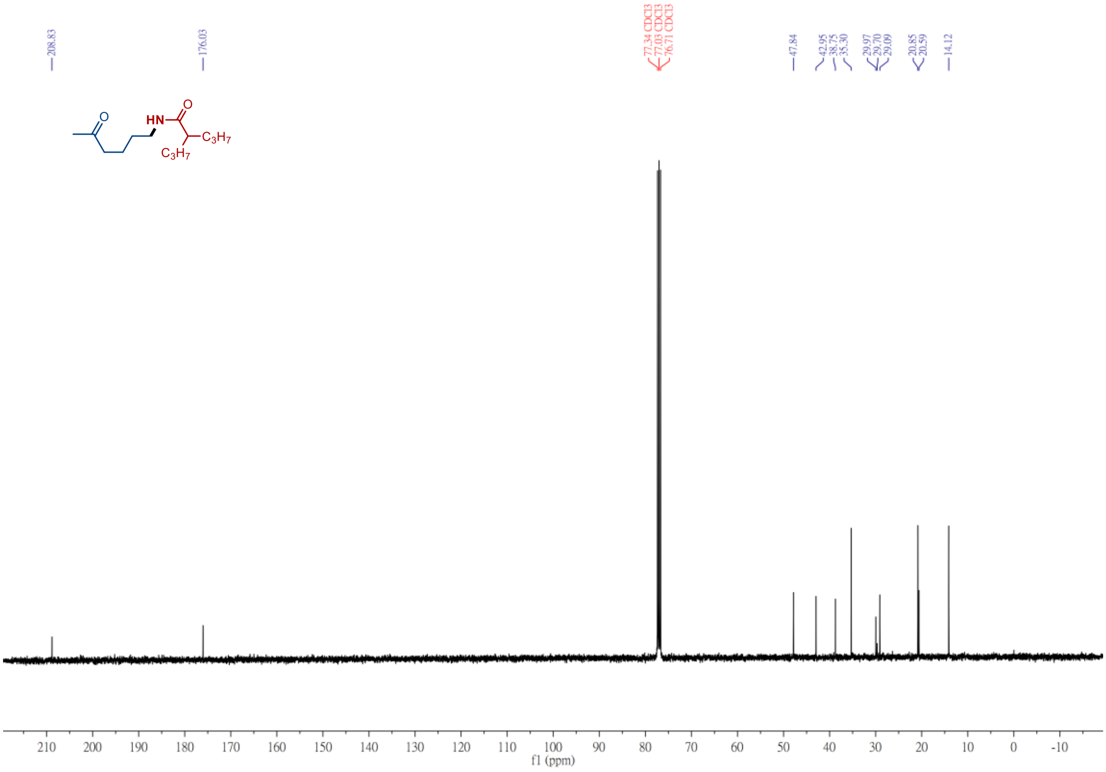

^{1}H and ^{13}C NMR spectrum of **74**

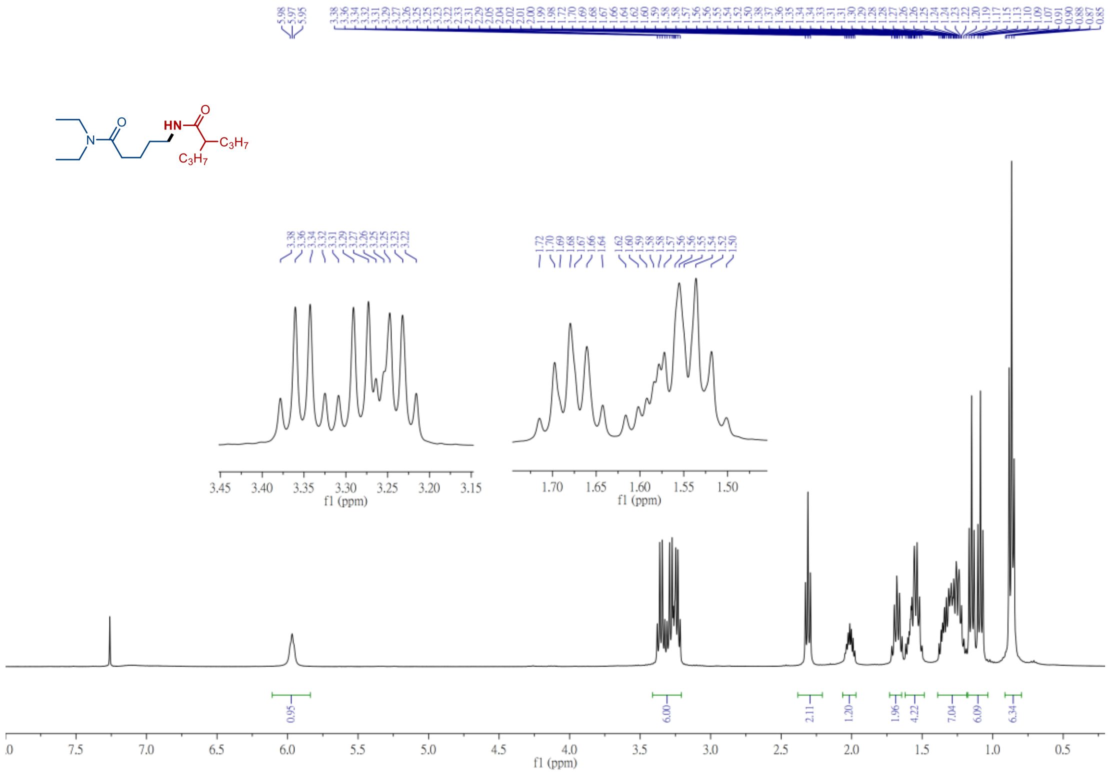

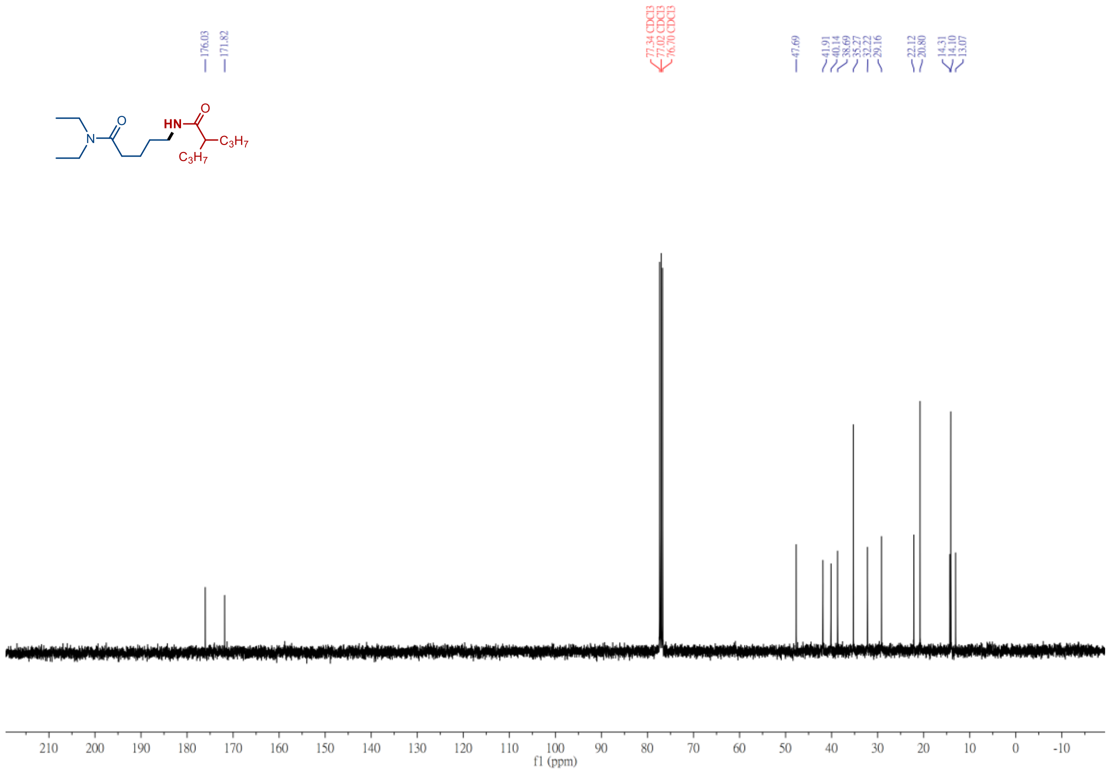

^{1}H and ^{13}C NMR spectrum of **75**

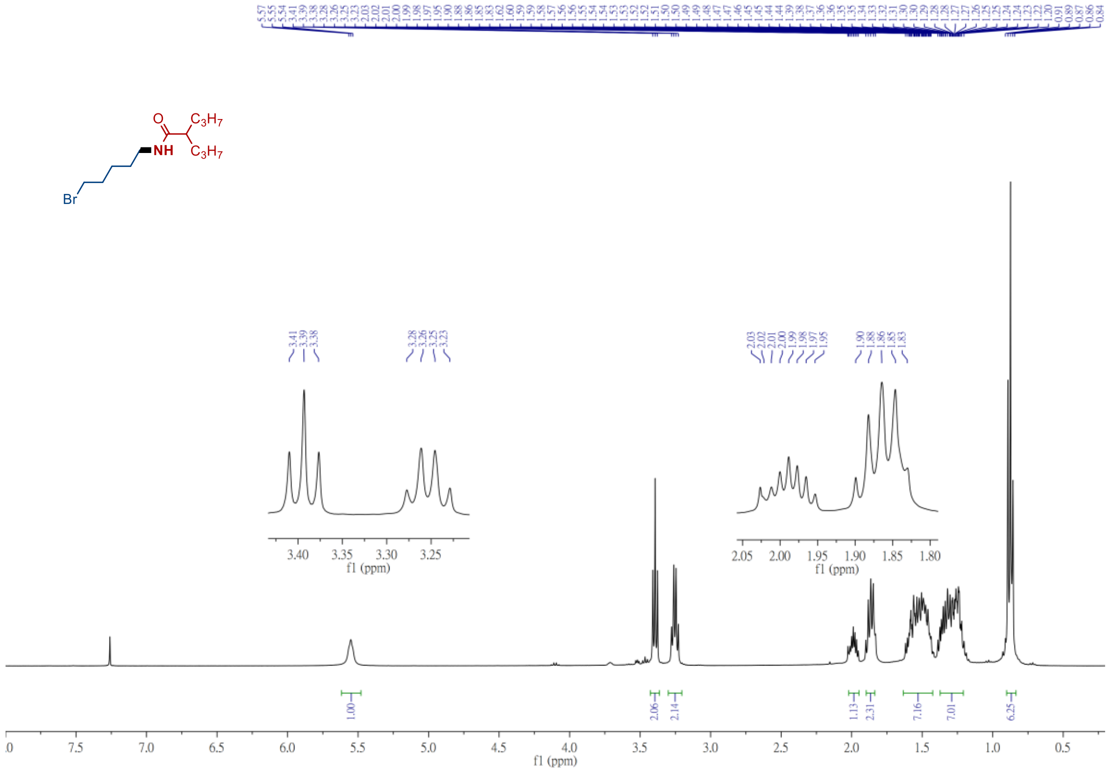

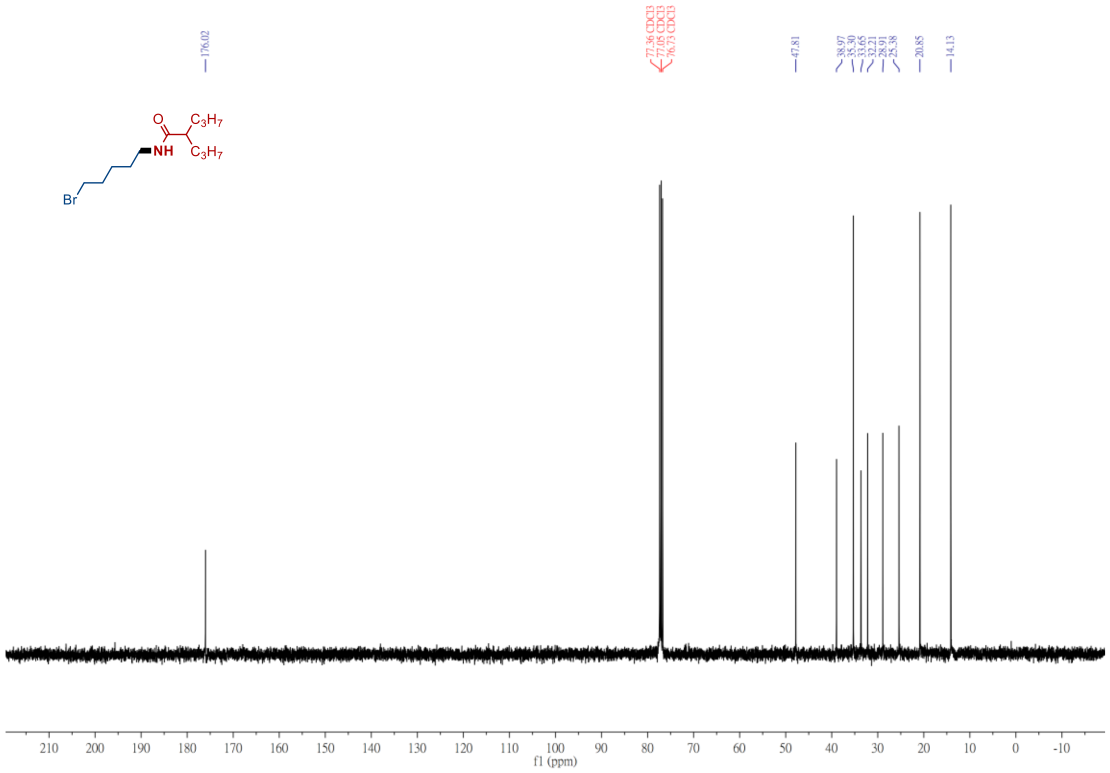

^{1}H and ^{13}C NMR spectrum of **76**

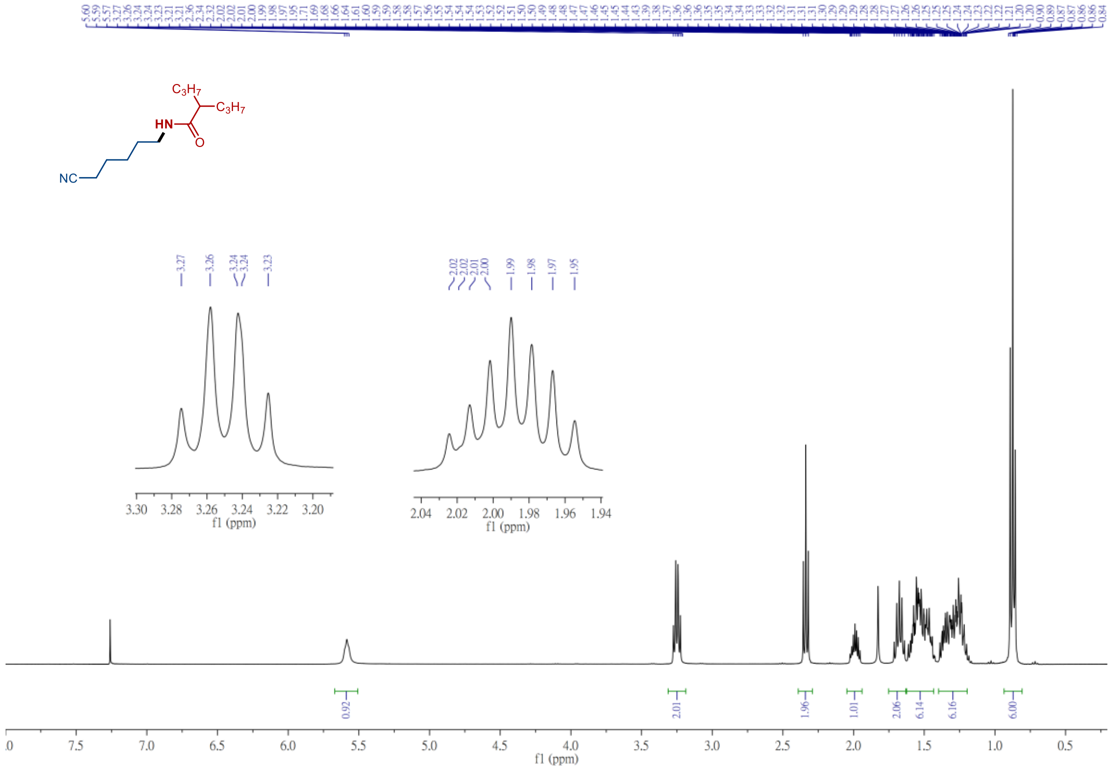

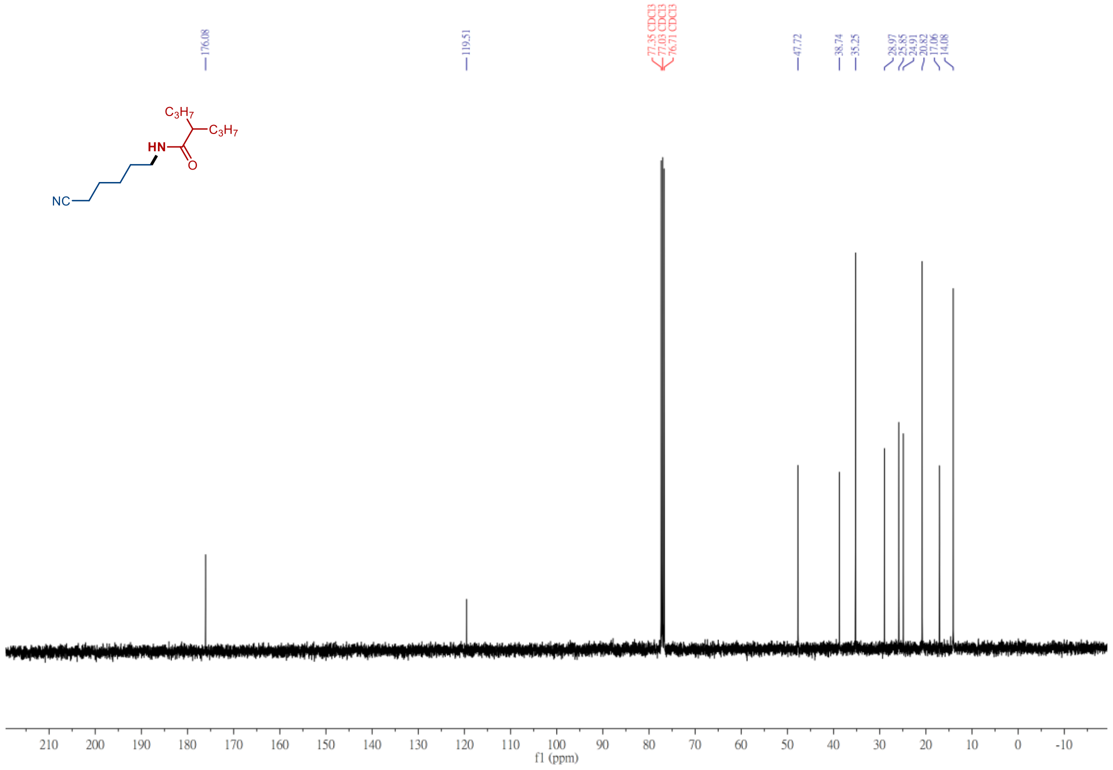

^{1}H and ^{13}C NMR spectrum of **77**

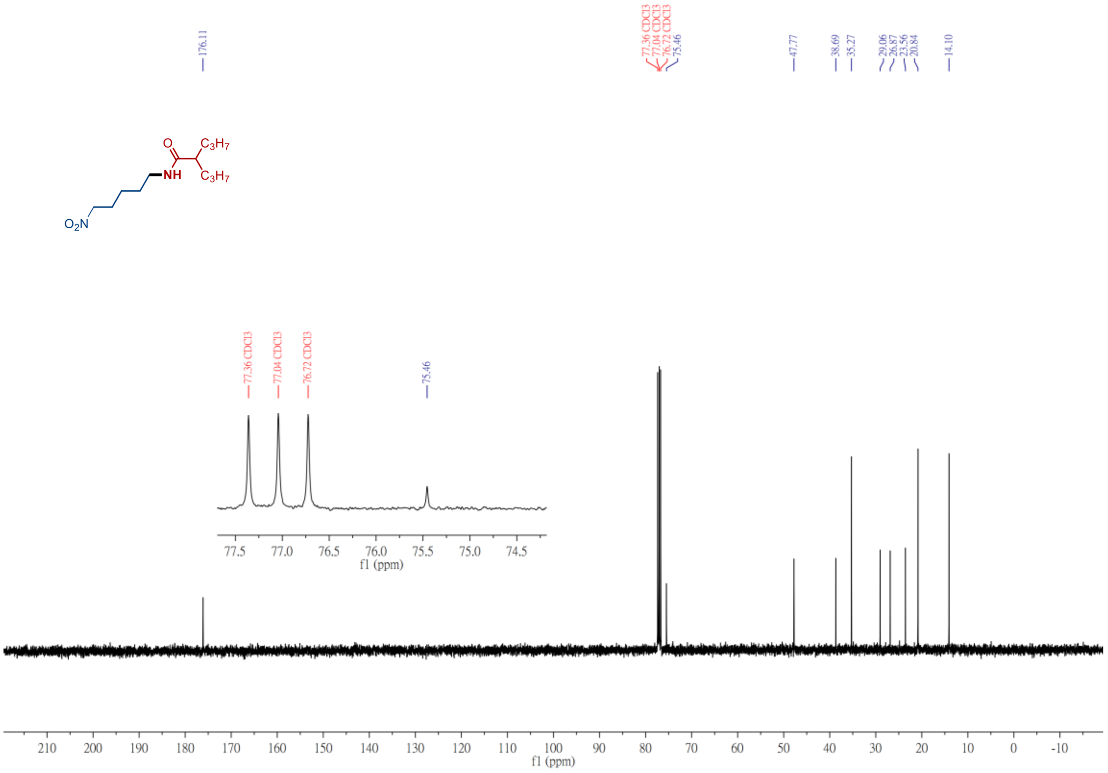

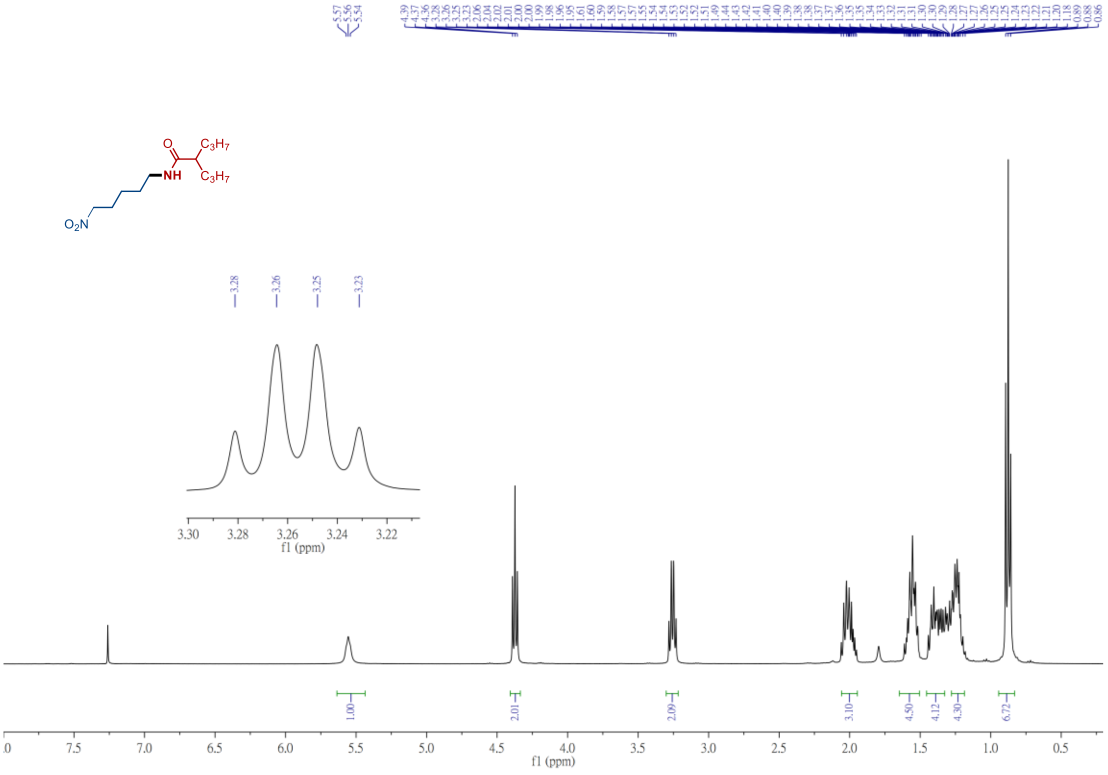

^{1}H and ^{13}C NMR spectrum of **78**

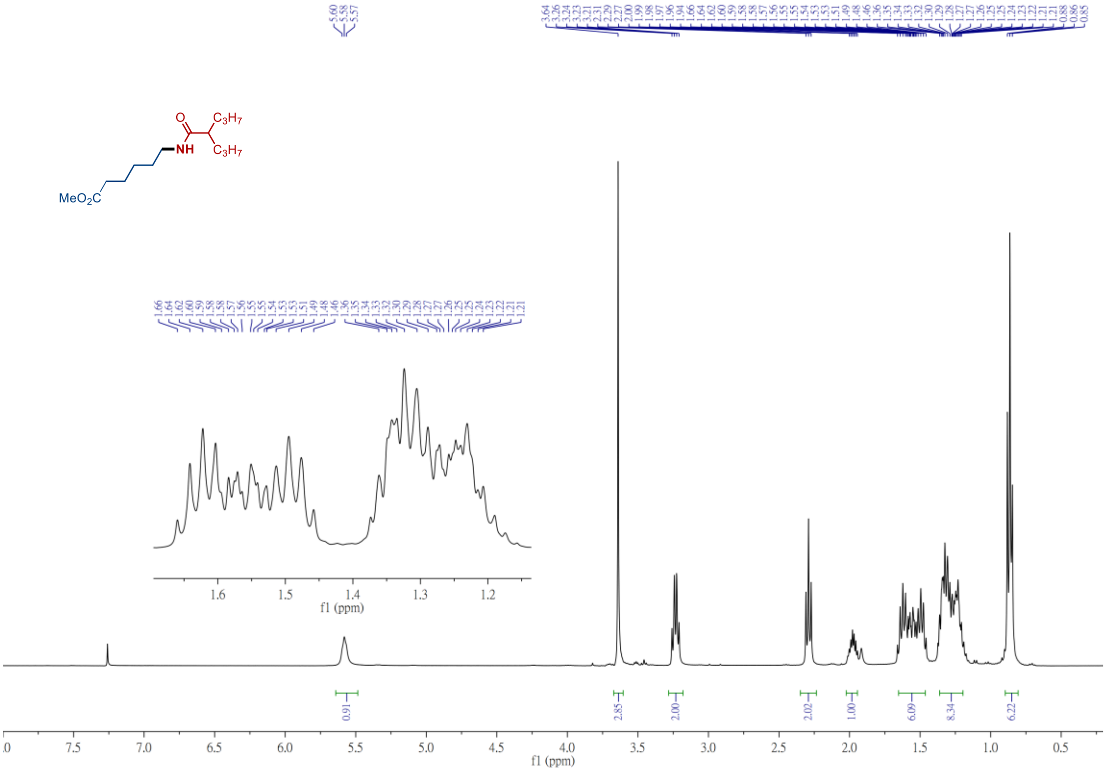

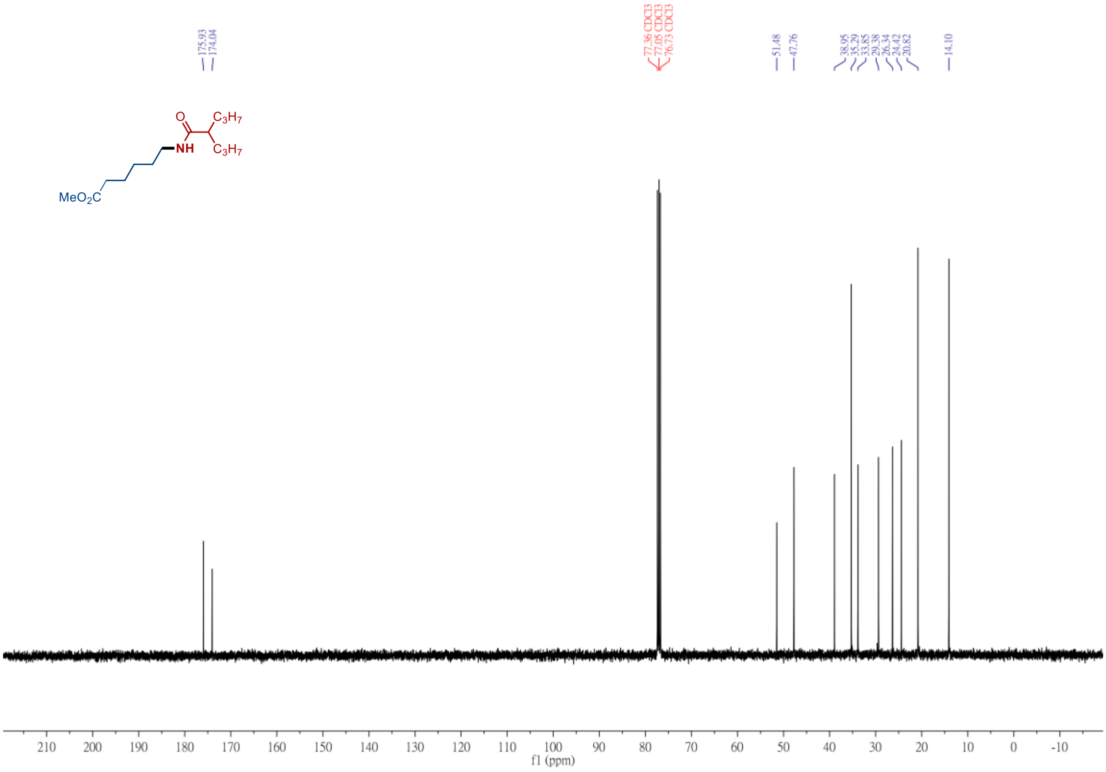

^{1}H and ^{13}C NMR spectrum of **79**

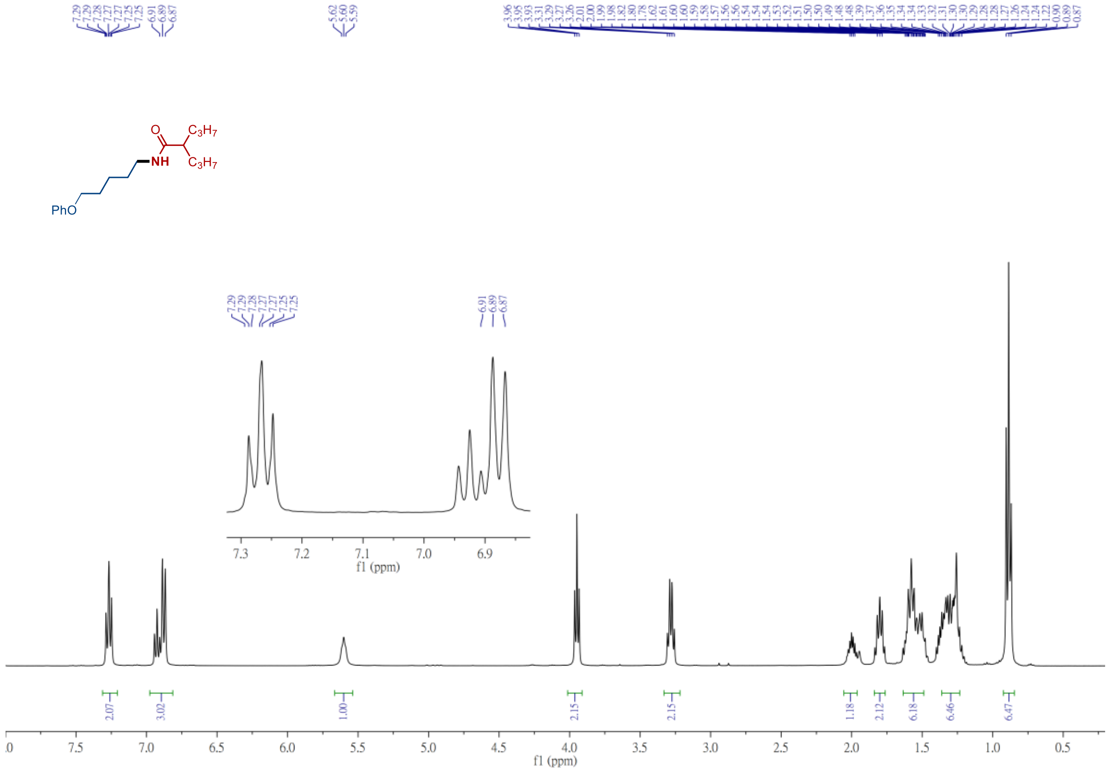

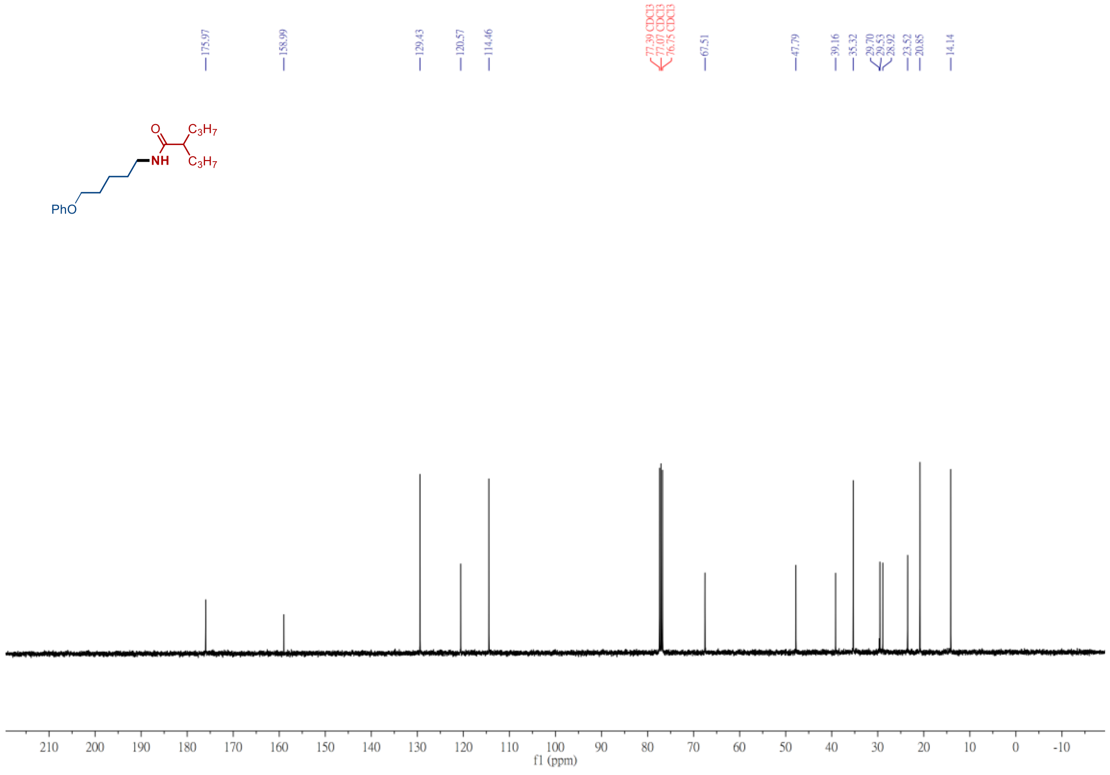

^{1}H and ^{13}C NMR spectrum of **80**

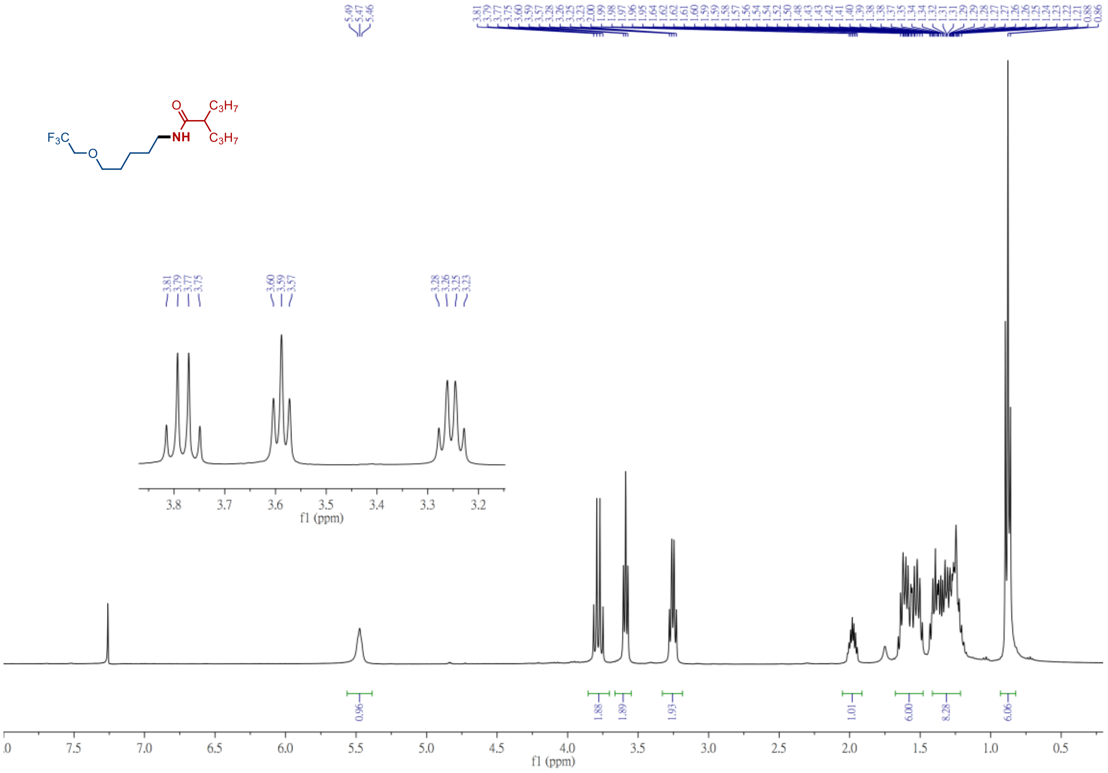

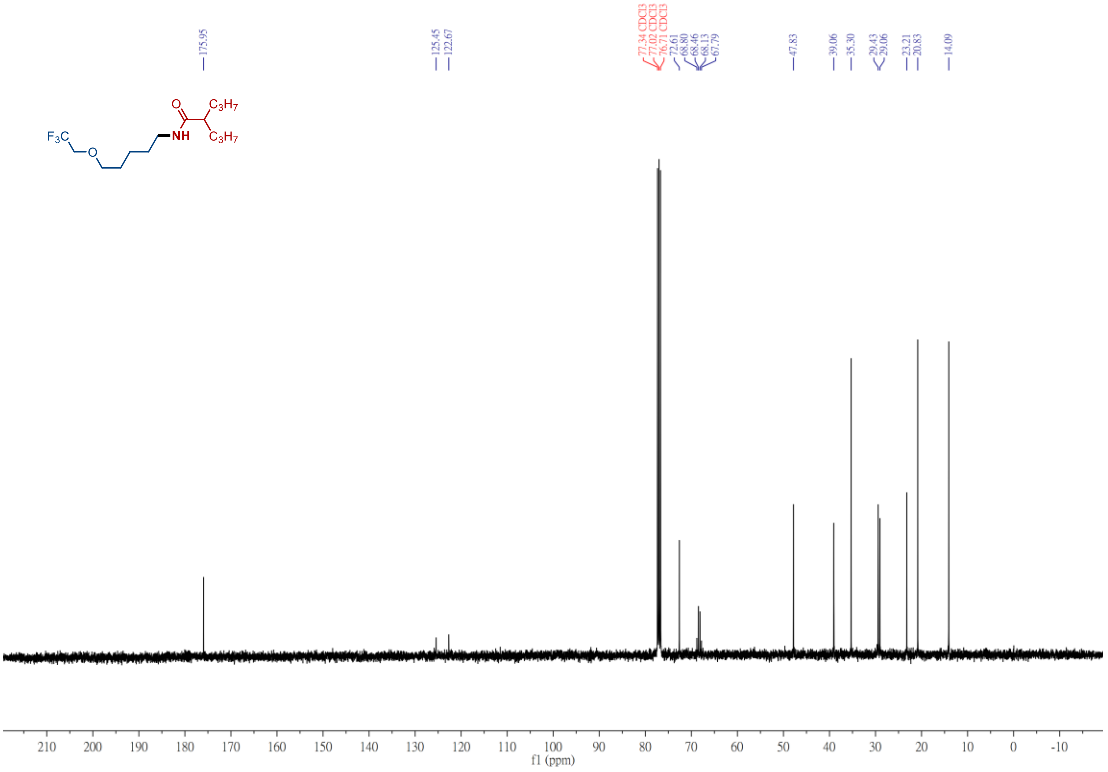

^{19}F NMR spectrum of **80**

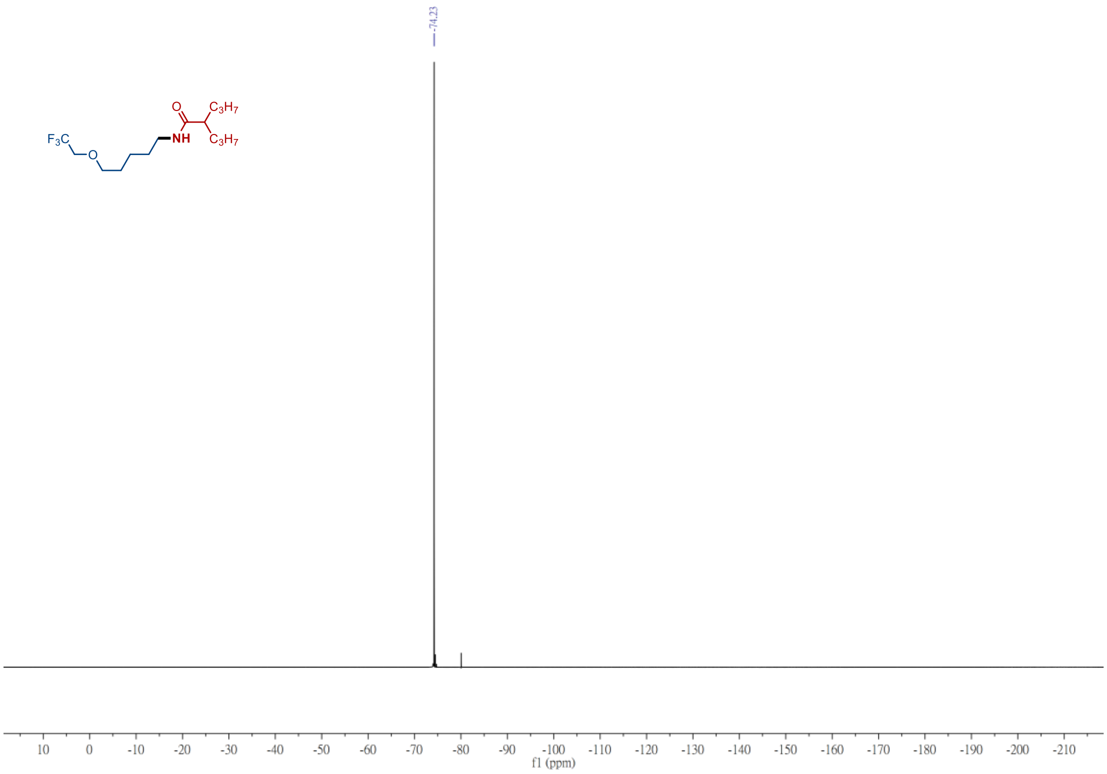

^{1}H and ^{13}C NMR spectrum of **81**

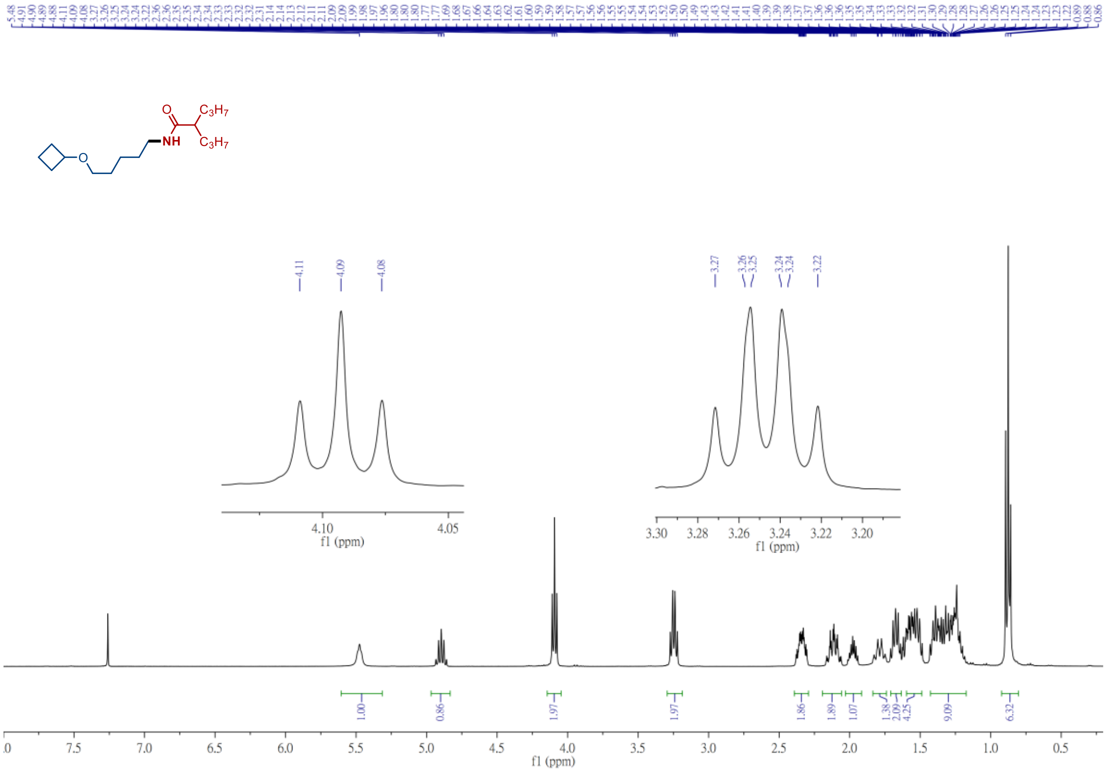

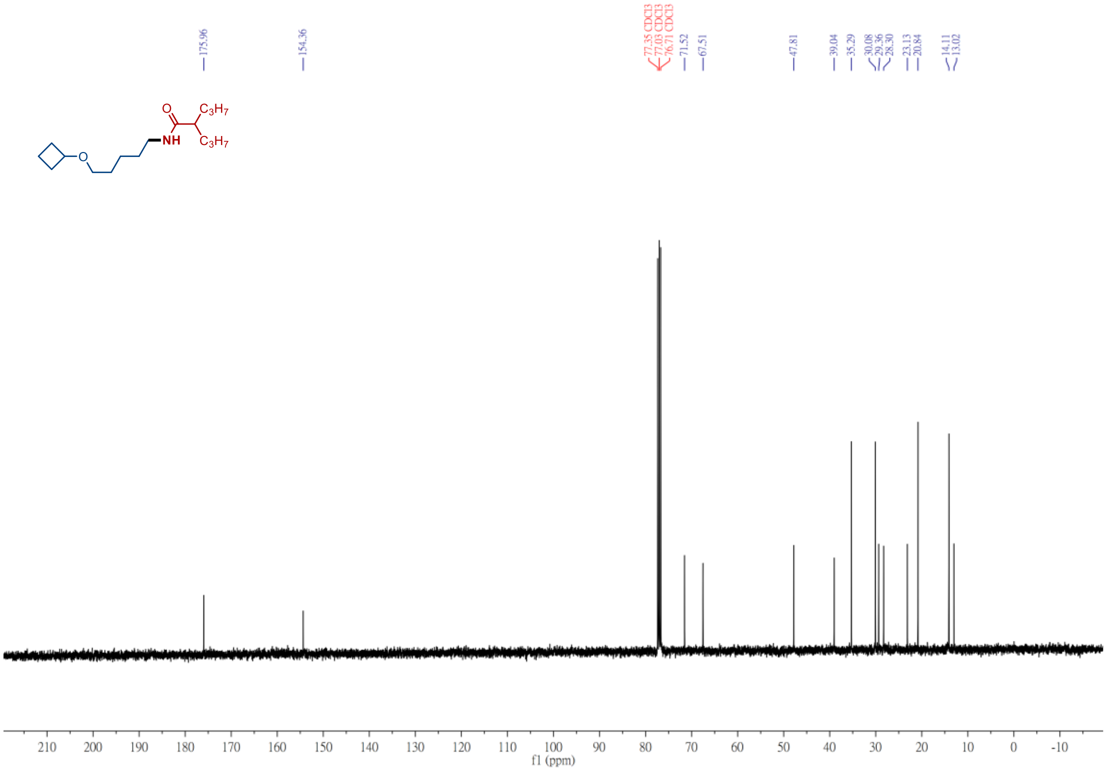

^{1}H and ^{13}C NMR spectrum of **82**

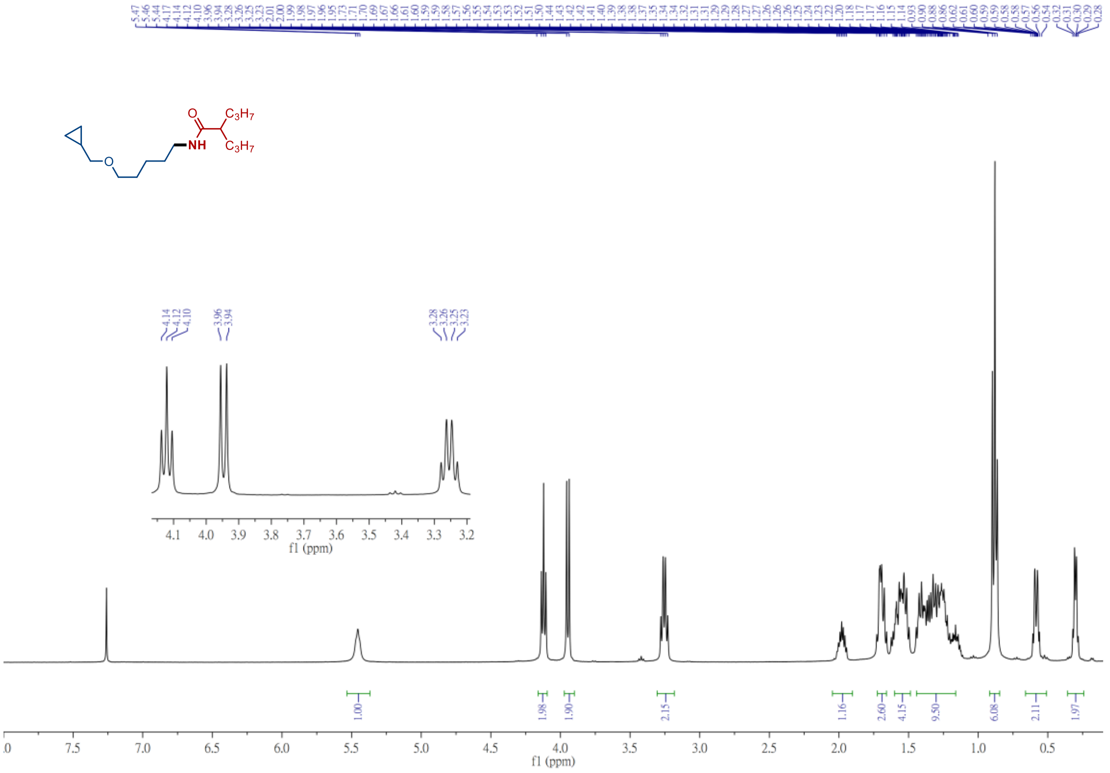

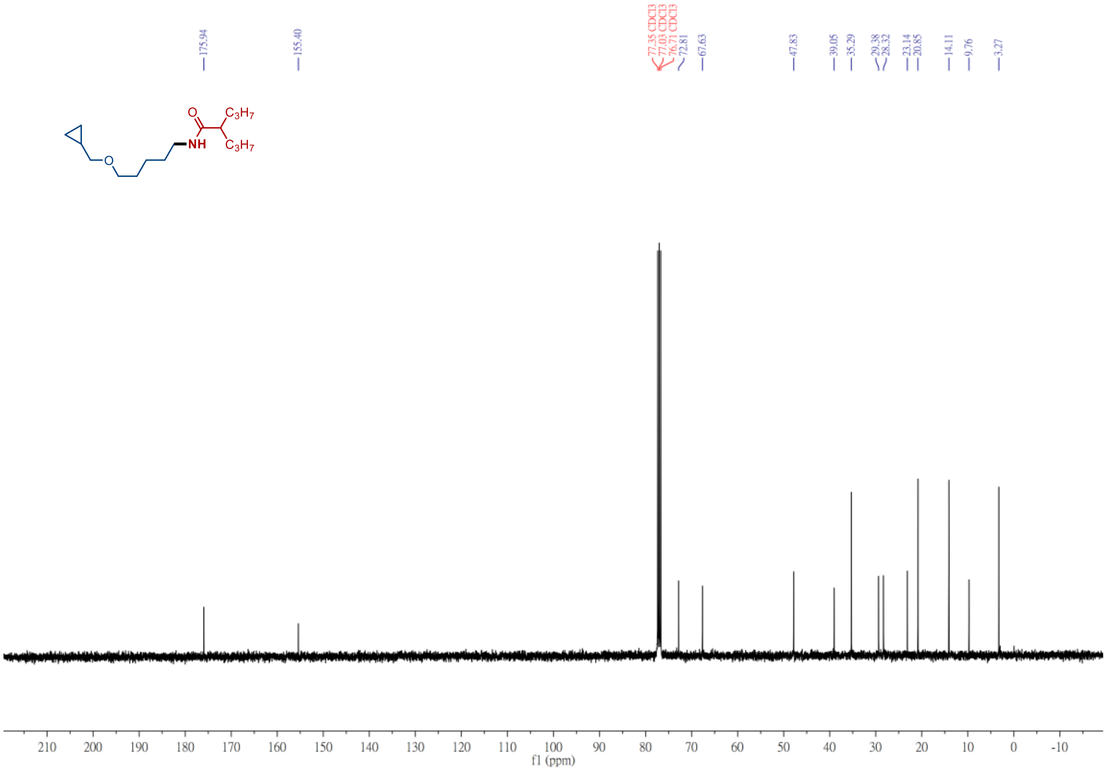

^{1}H and ^{13}C NMR spectrum of **83**

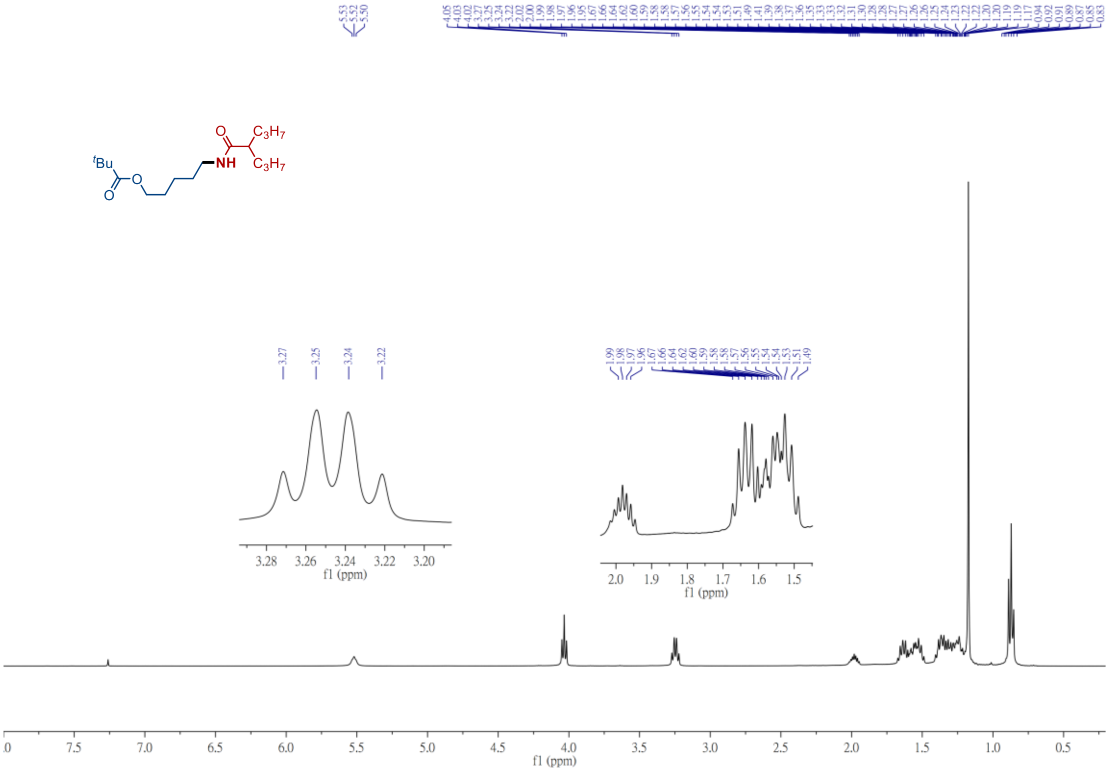

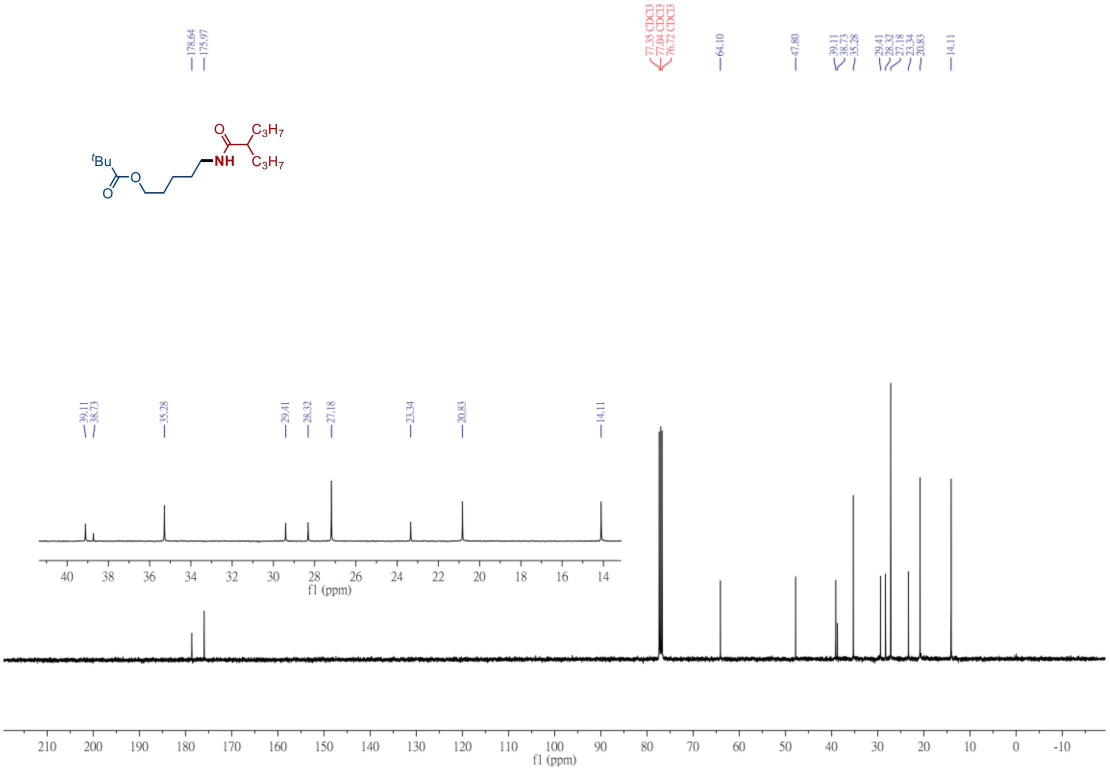

^{1}H and ^{13}C NMR spectrum of **84**

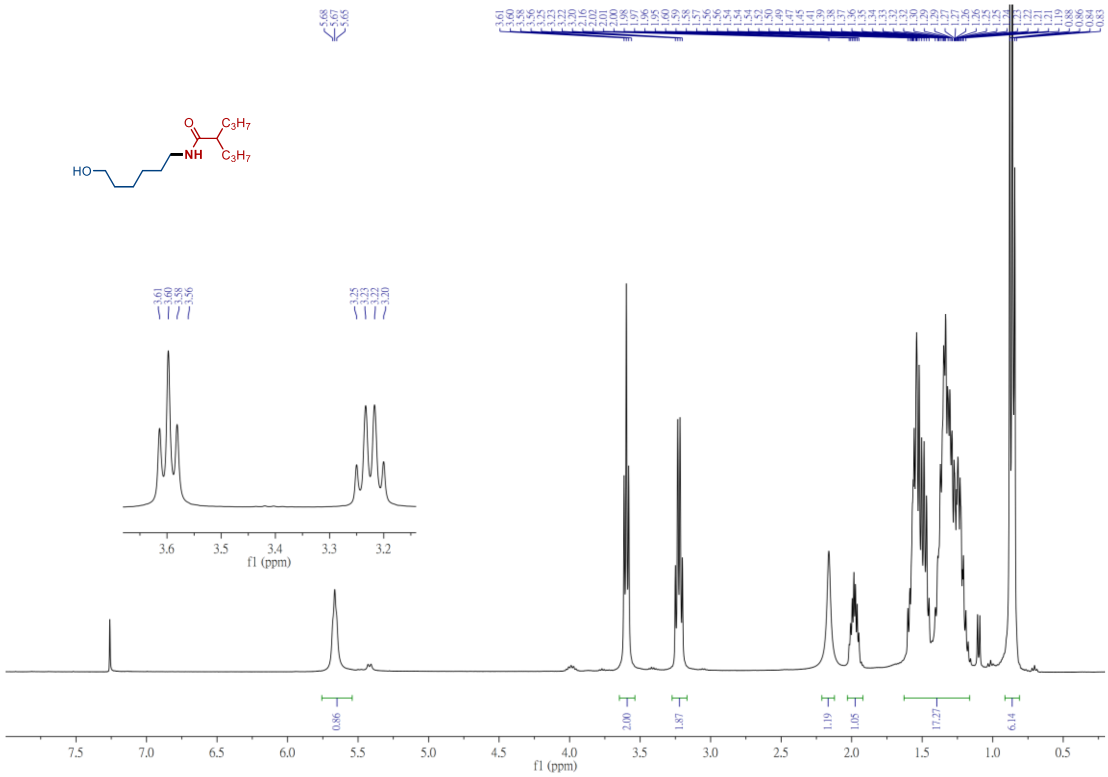

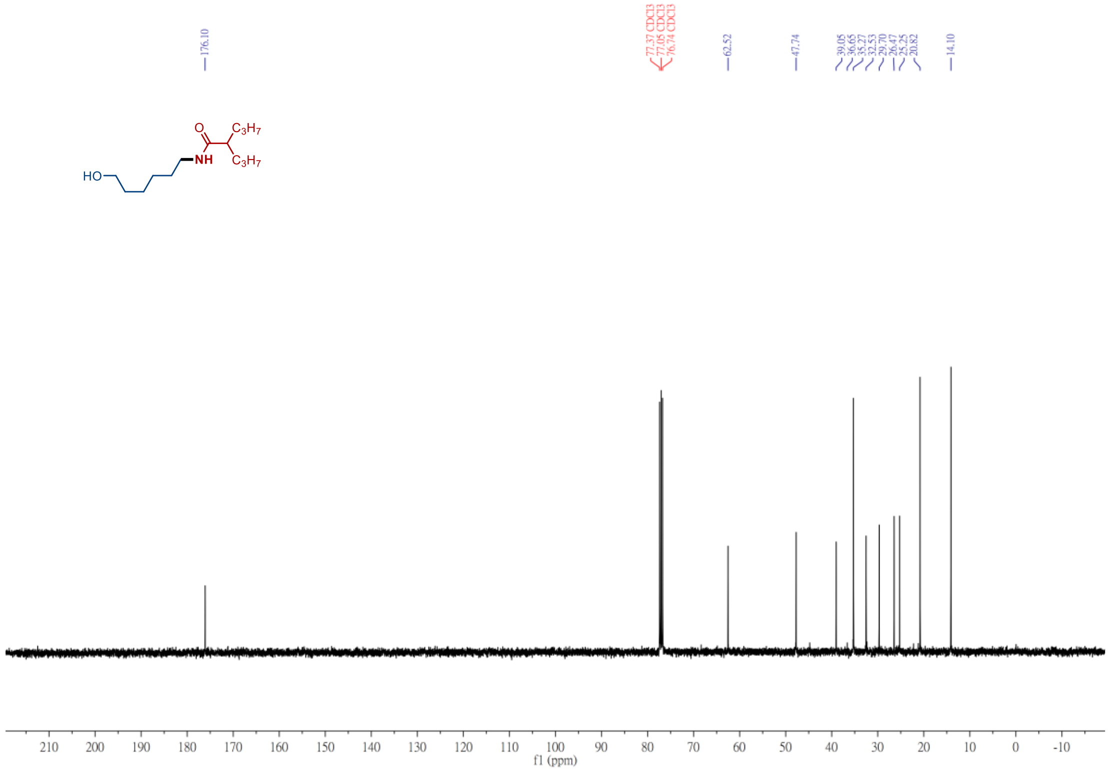

^{1}H and ^{13}C NMR spectrum of **85**

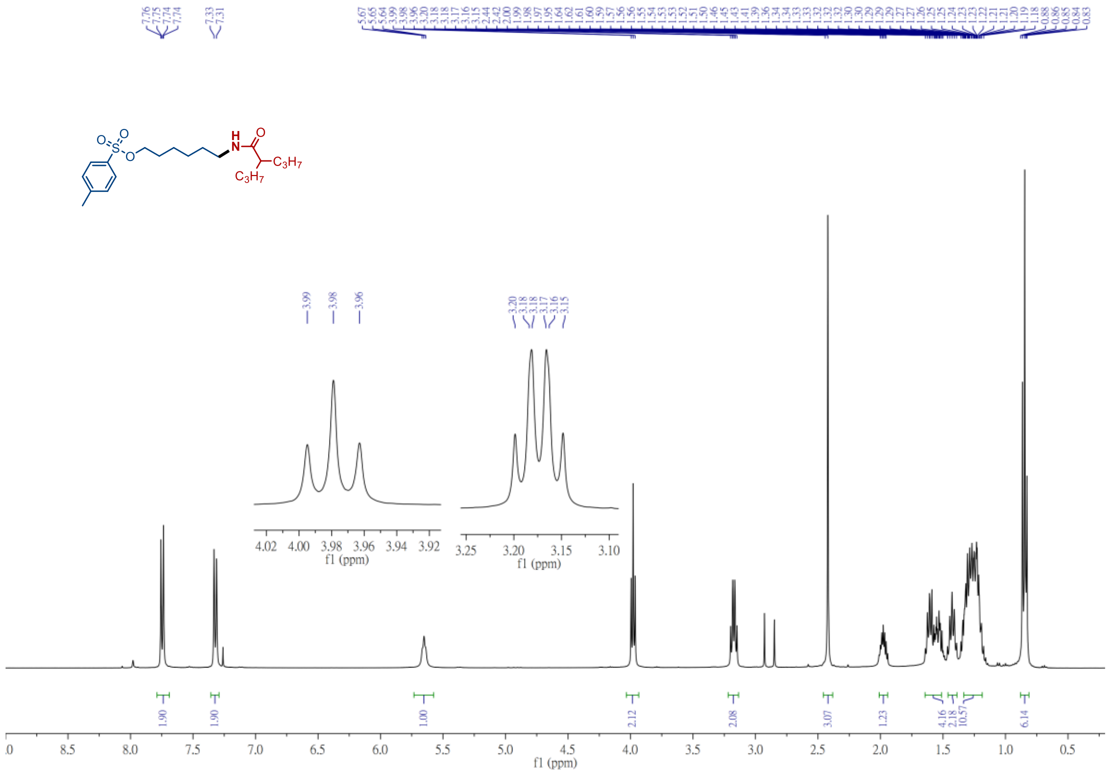

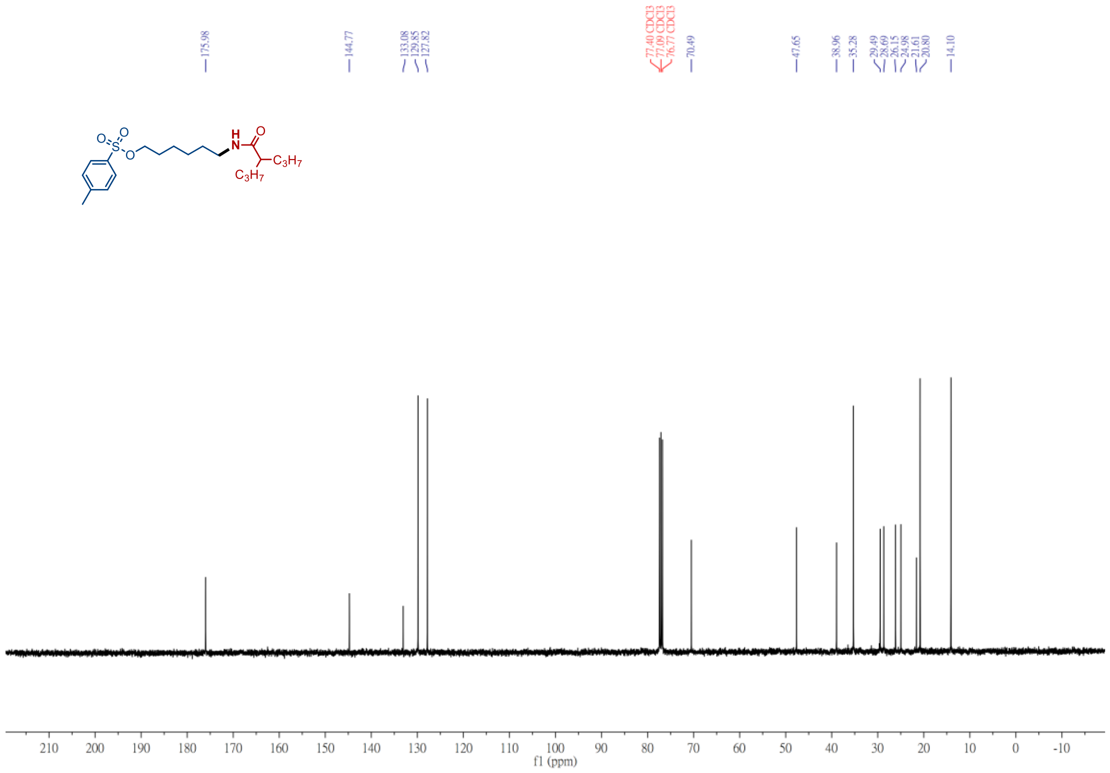

^{1}H and ^{13}C NMR spectrum of **86**

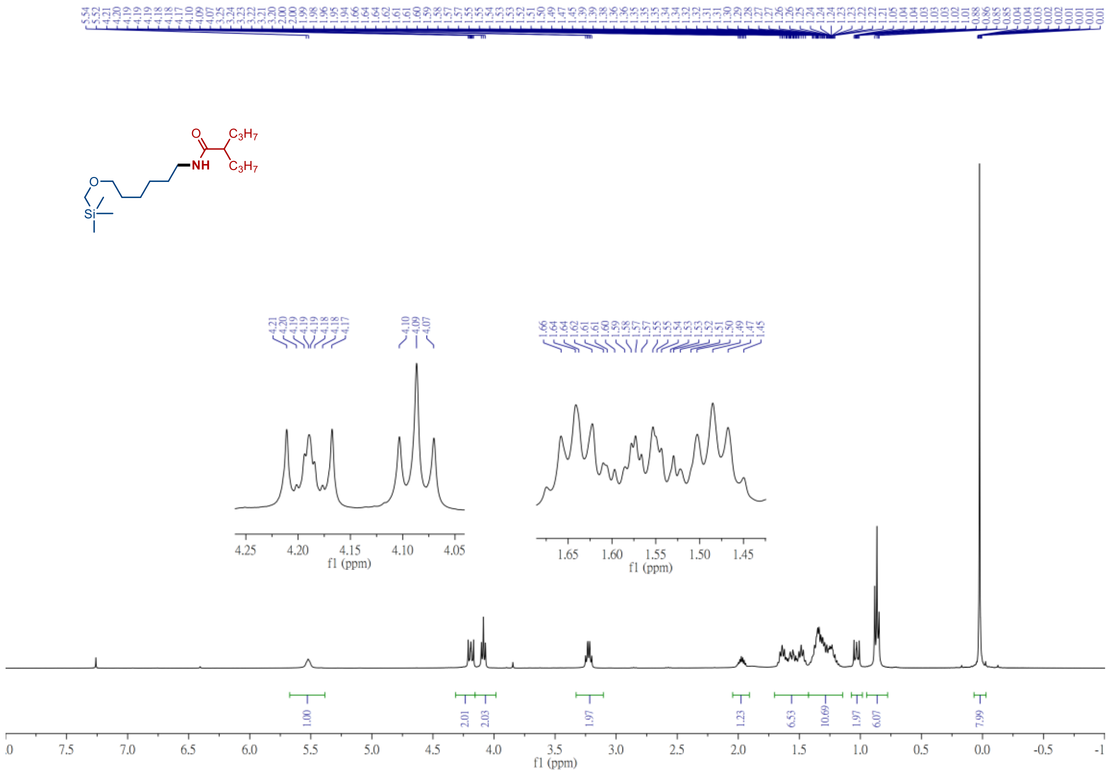

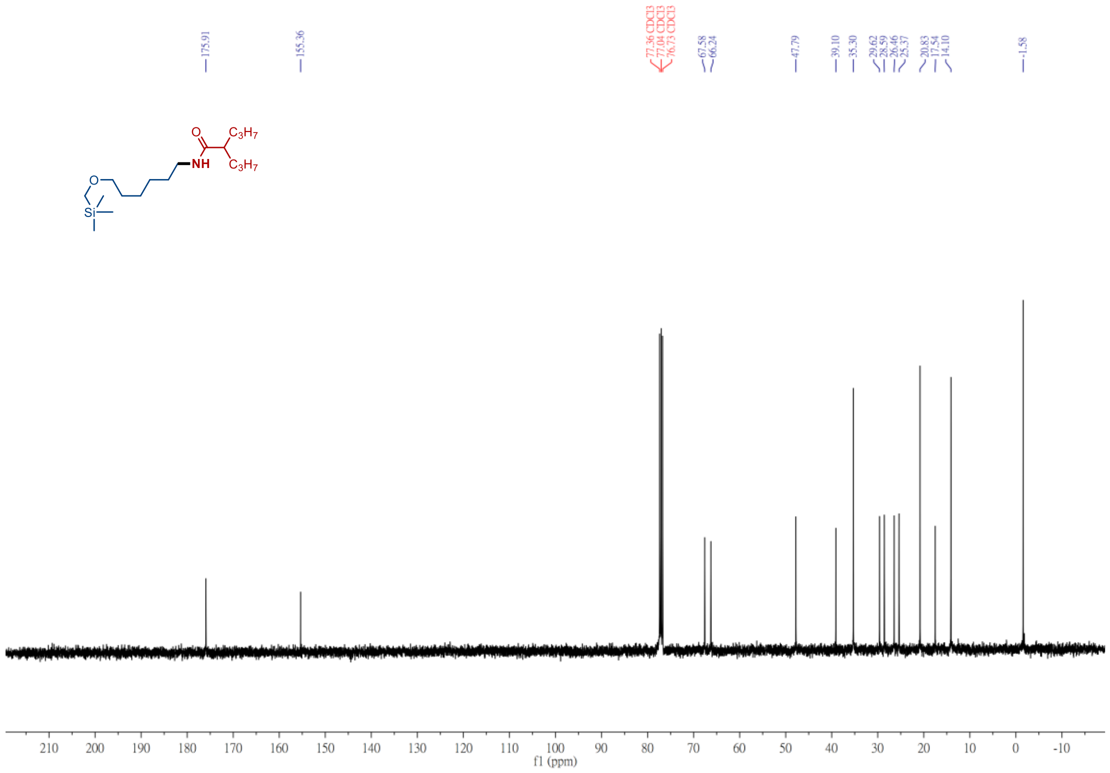

^{1}H and ^{13}C NMR spectrum of **87**

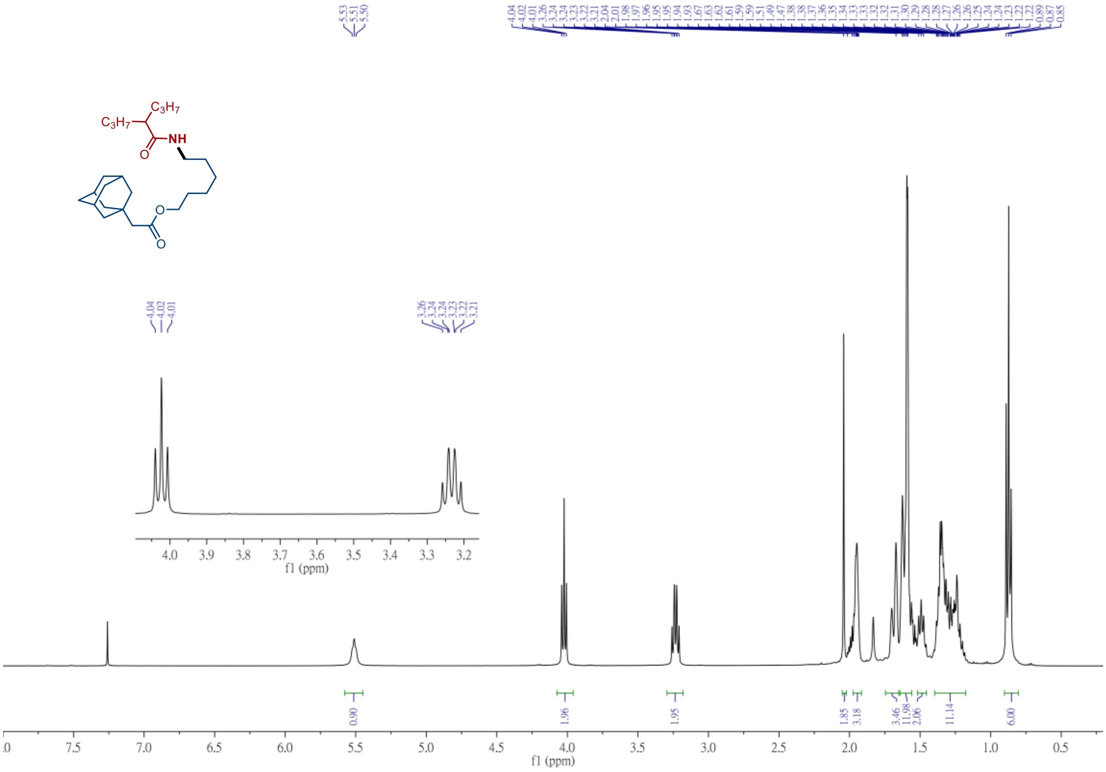

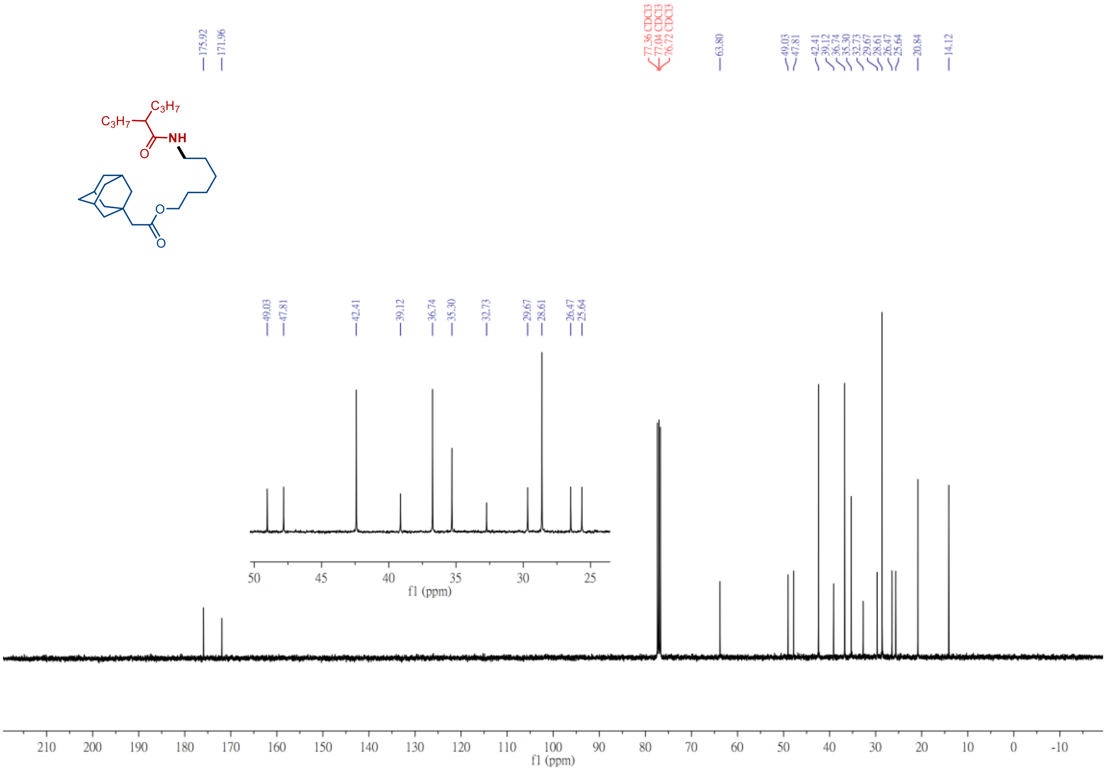

^{1}H and ^{13}C NMR spectrum of **88**

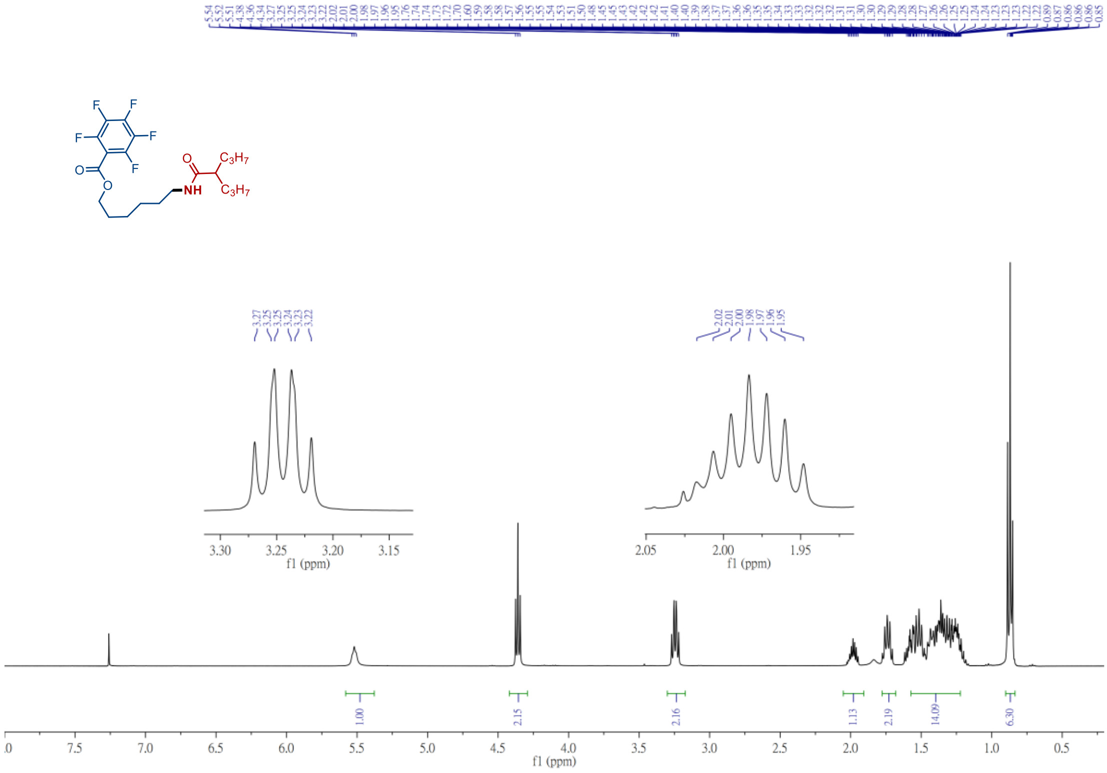

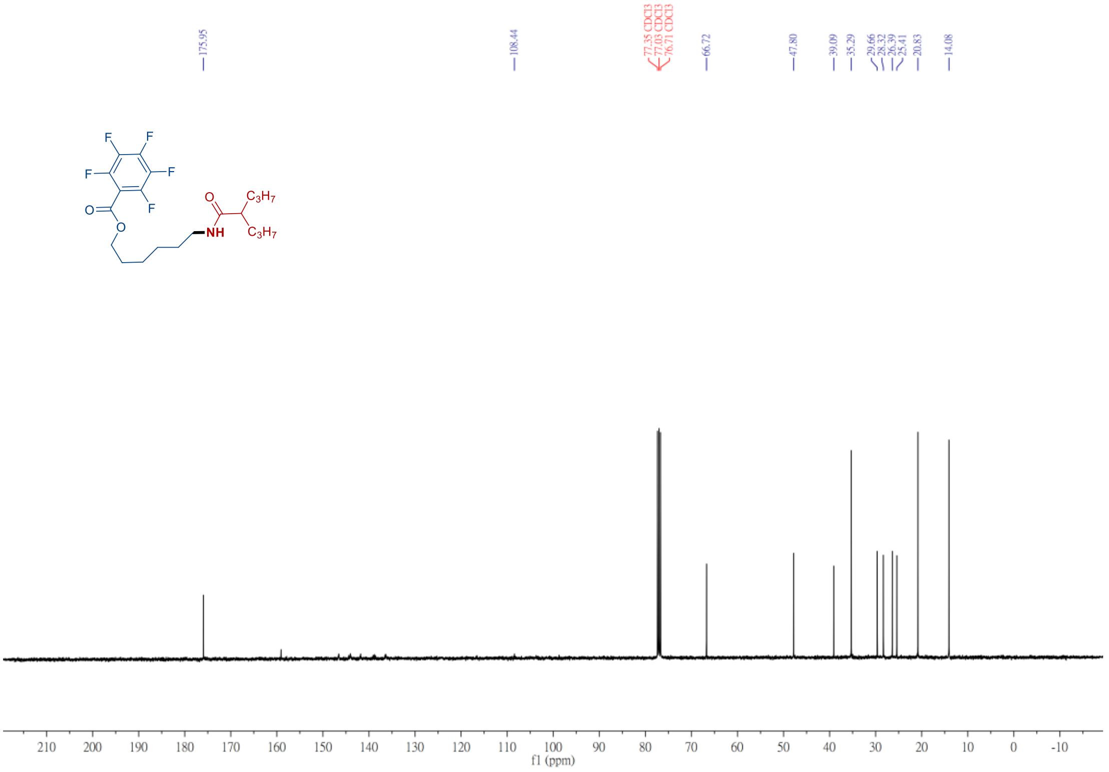

^{19}F NMR spectrum of **88**

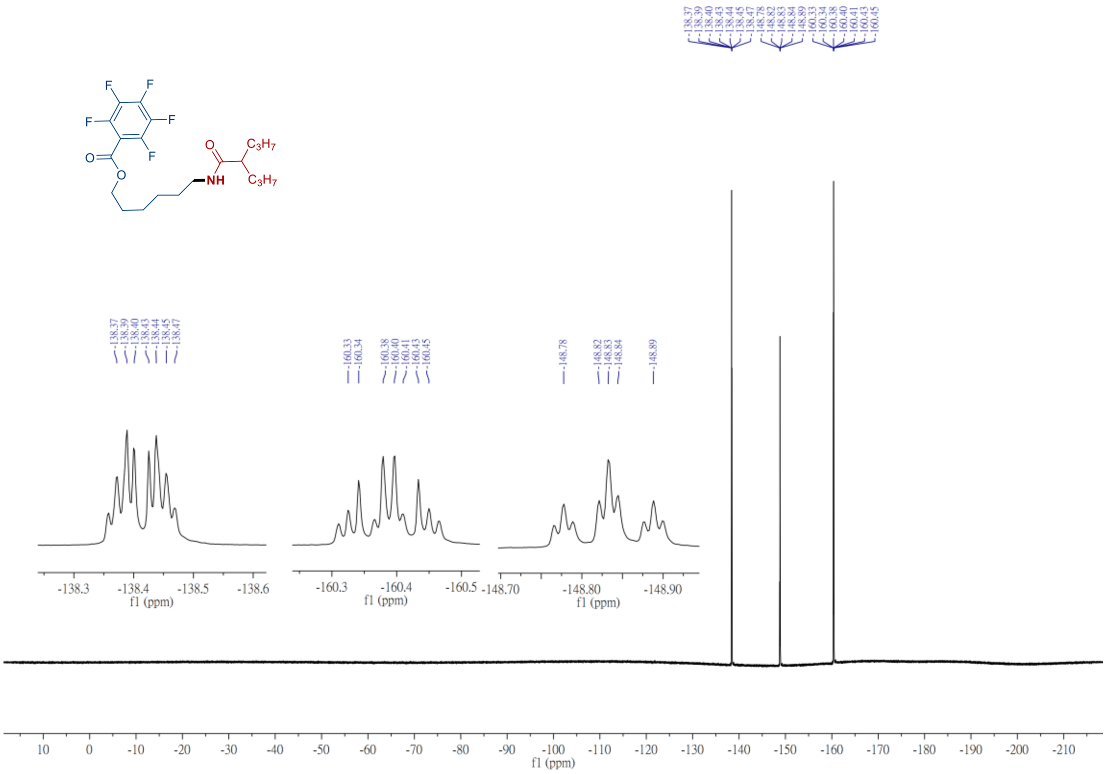

^{1}H and ^{13}C NMR spectrum of **89**

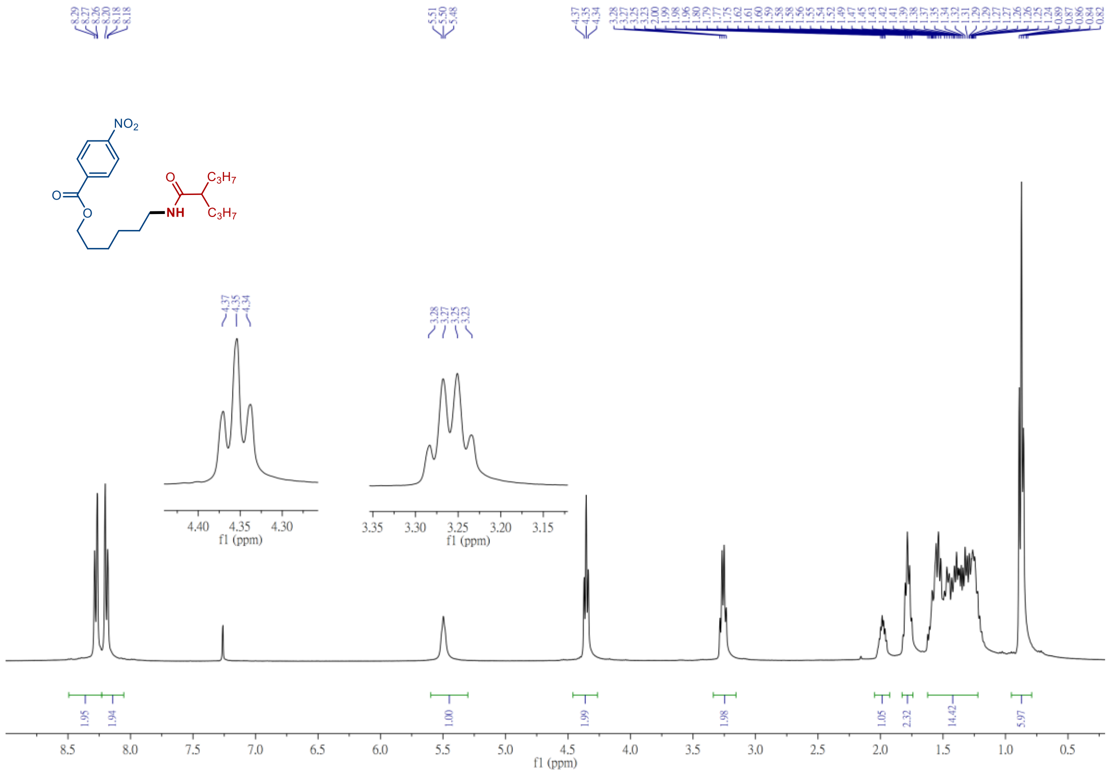

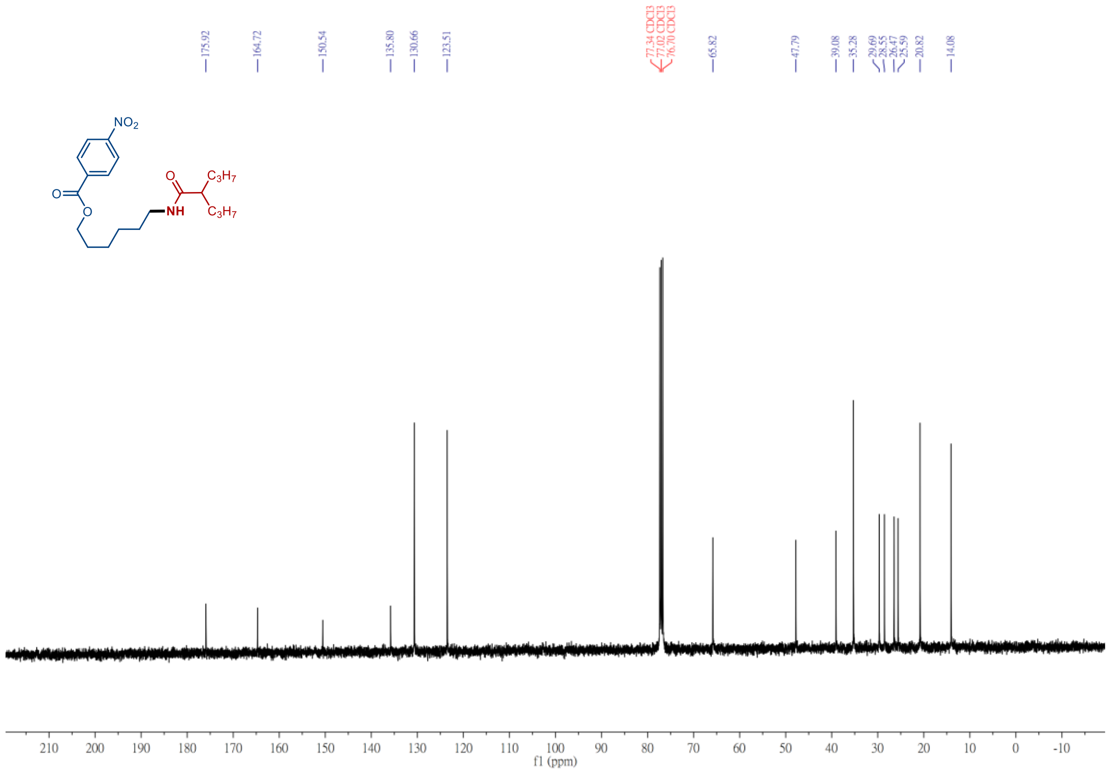

^{1}H and ^{13}C NMR spectrum of **90**

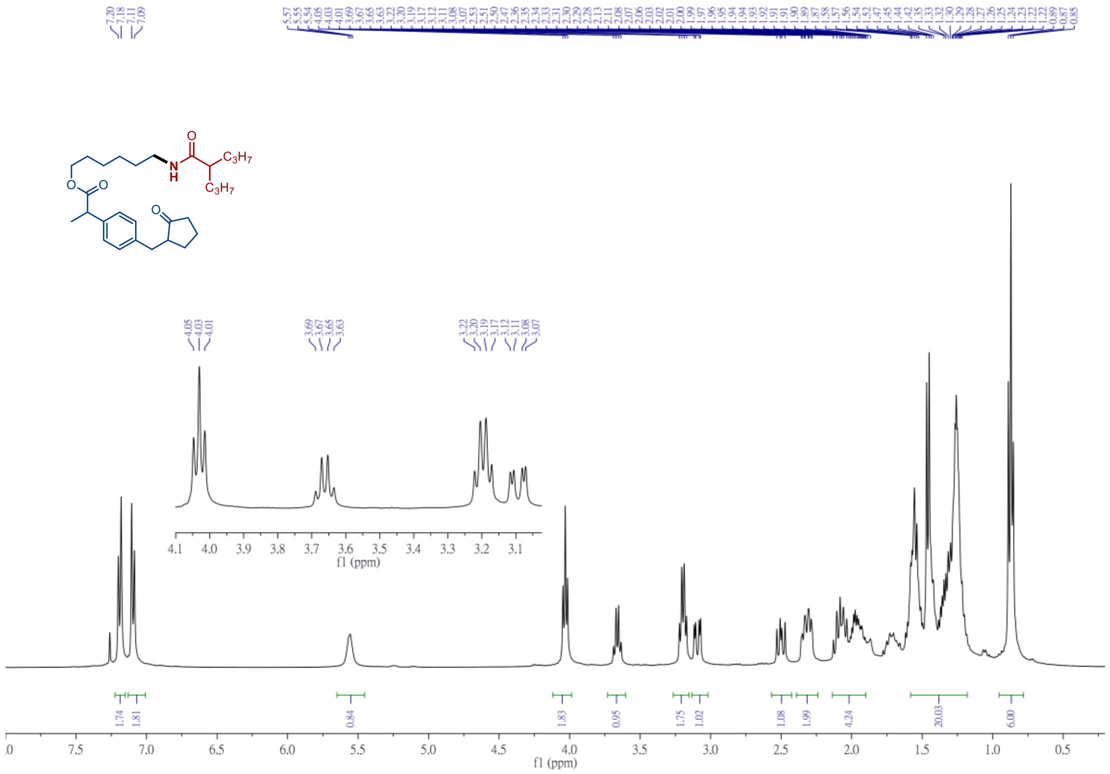

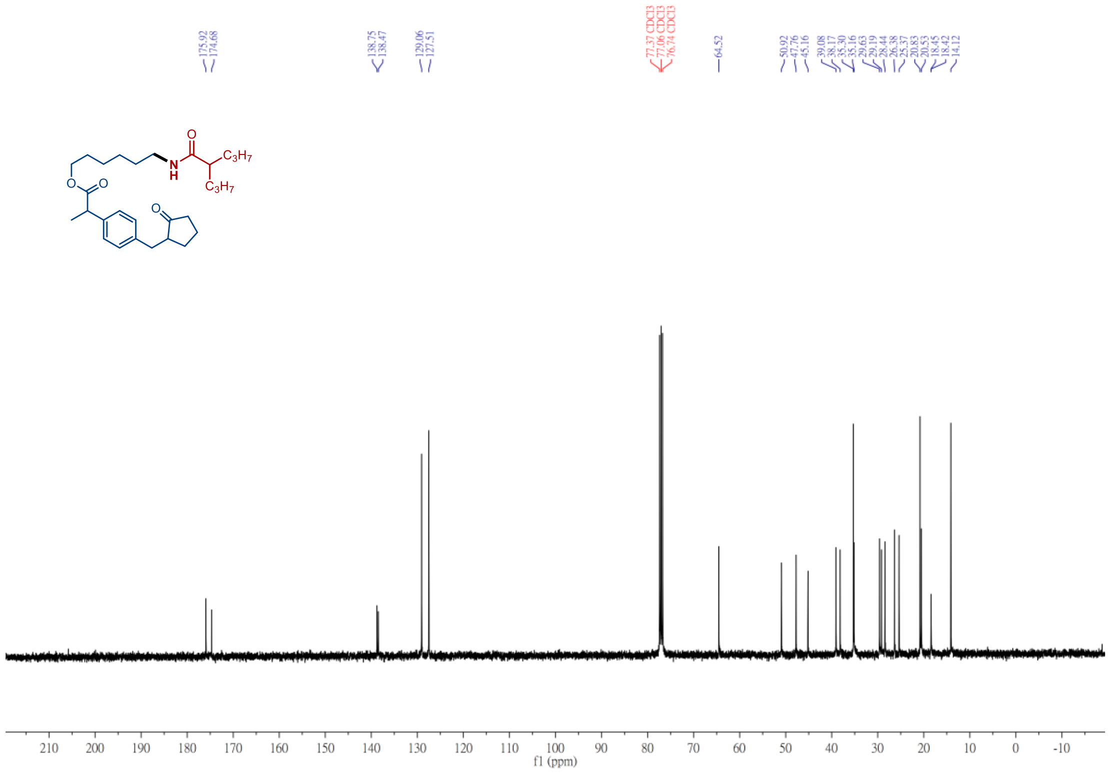

^{1}H and ^{13}C NMR spectrum of **91**

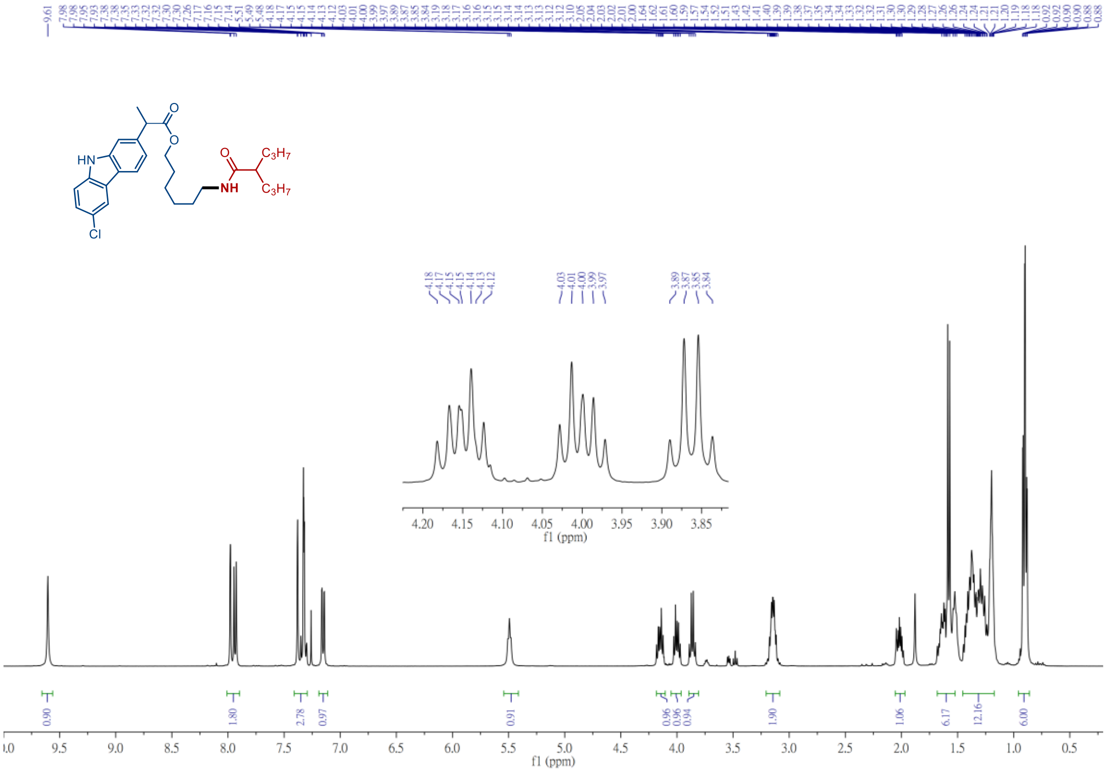

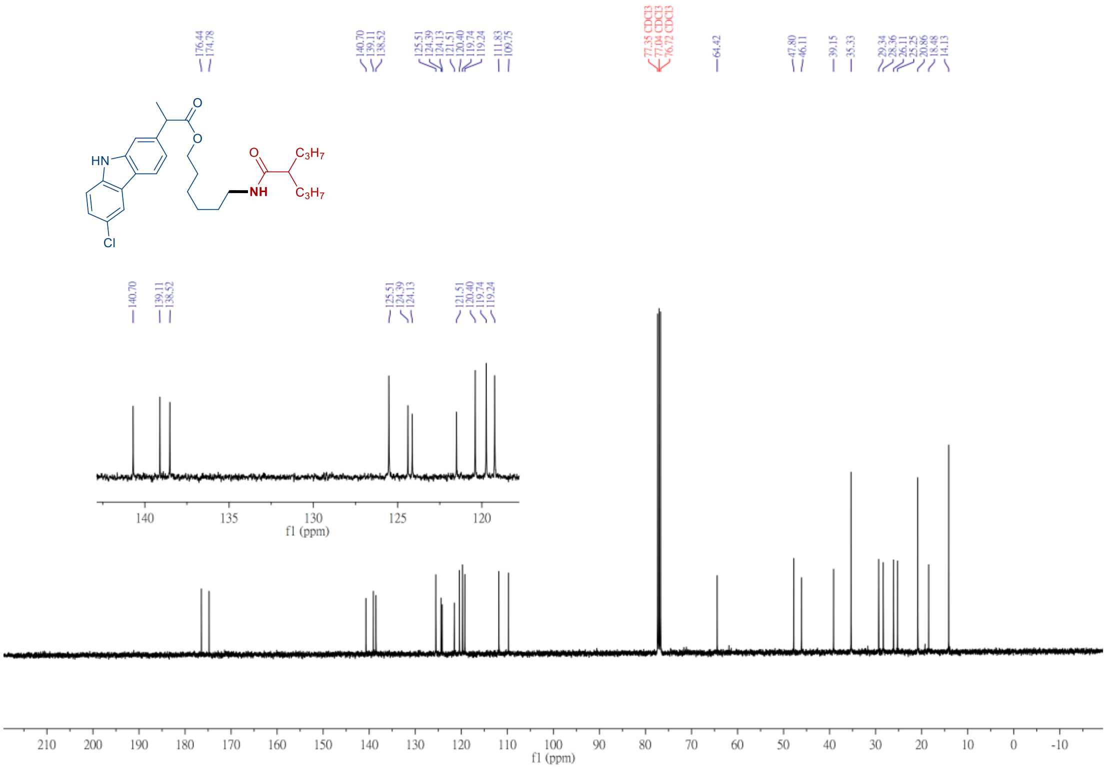

^{1}H and ^{13}C NMR spectrum of **92**

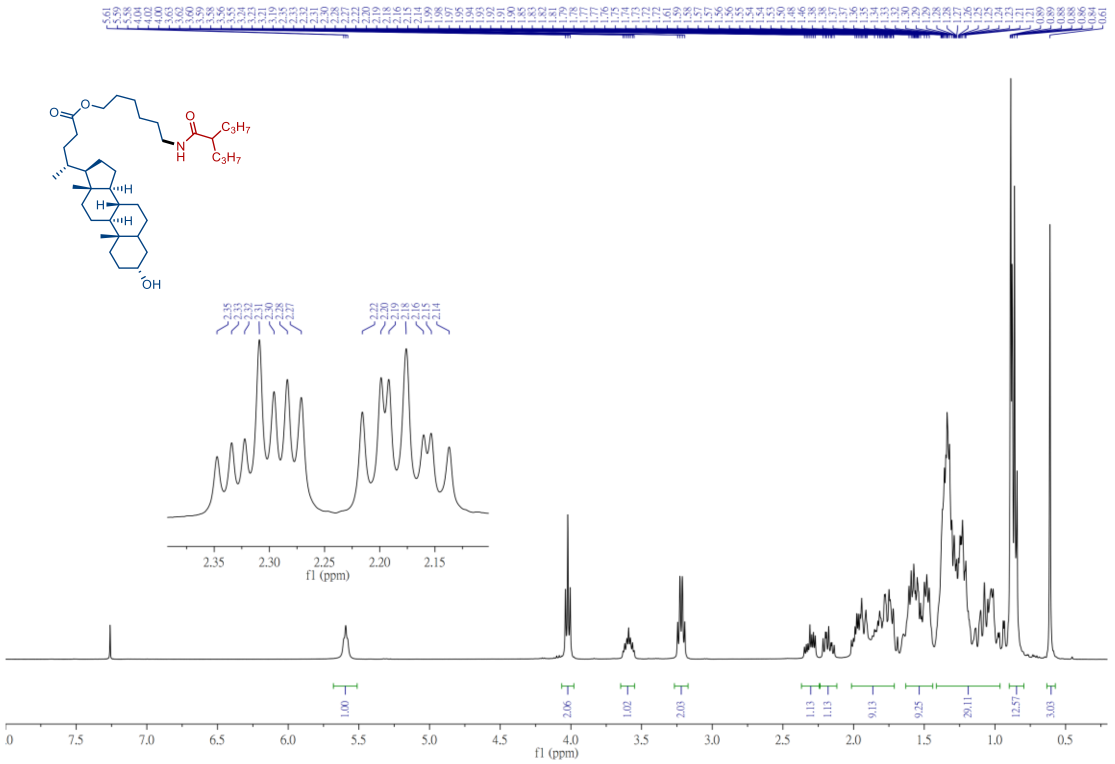

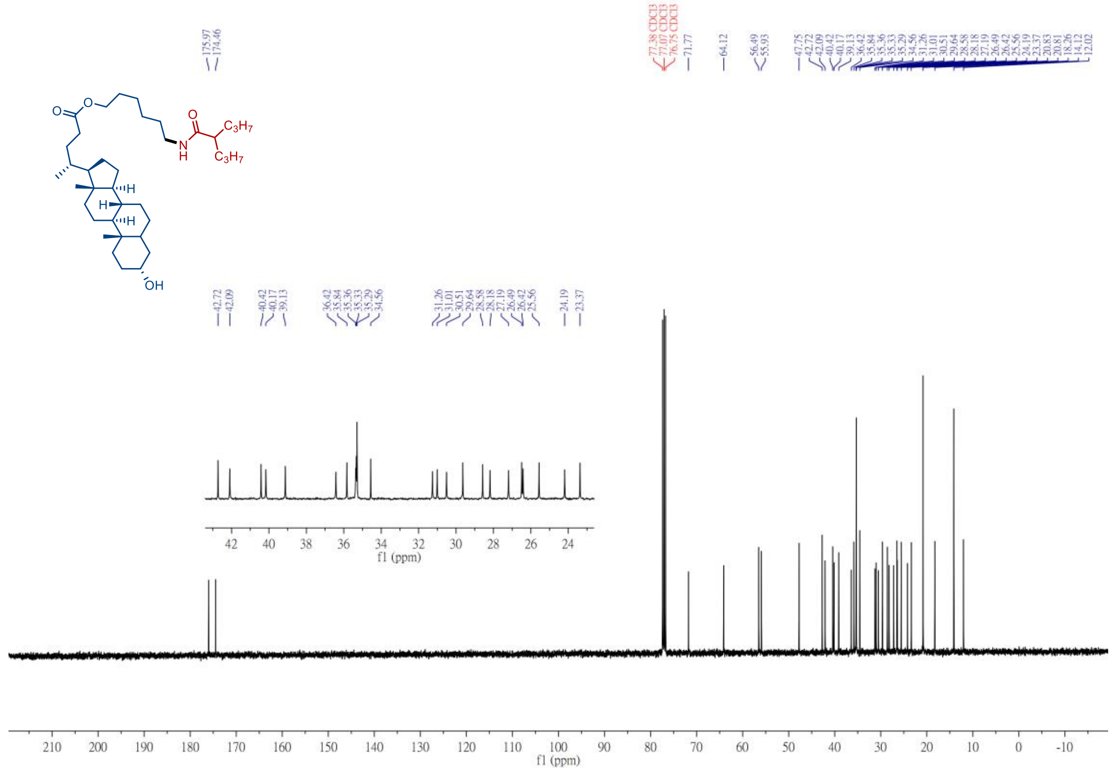

^{1}H and ^{13}C NMR spectrum of **93**

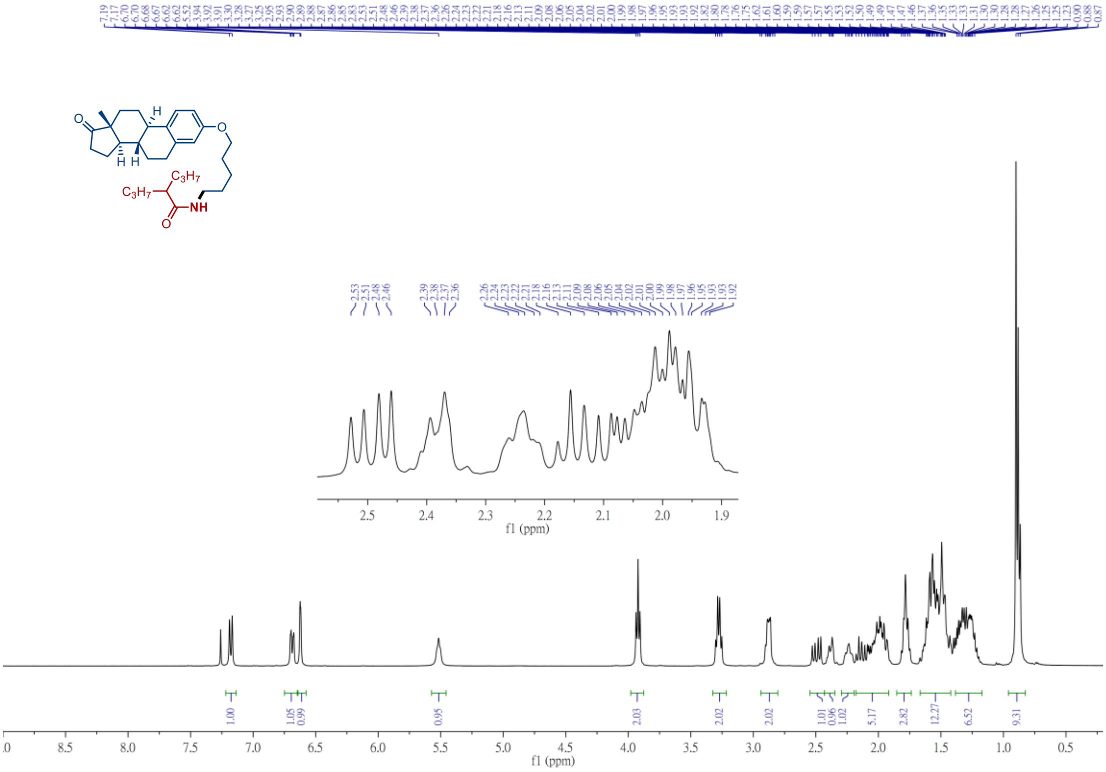

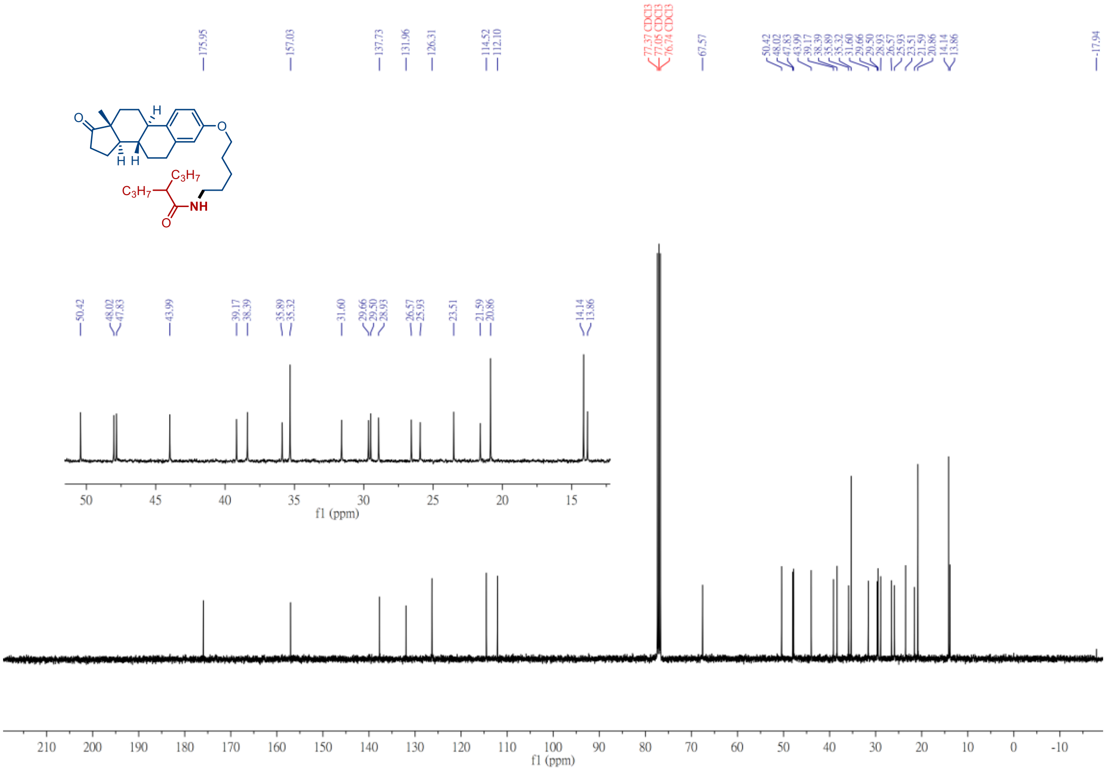

^{1}H and ^{13}C NMR spectrum of **134**

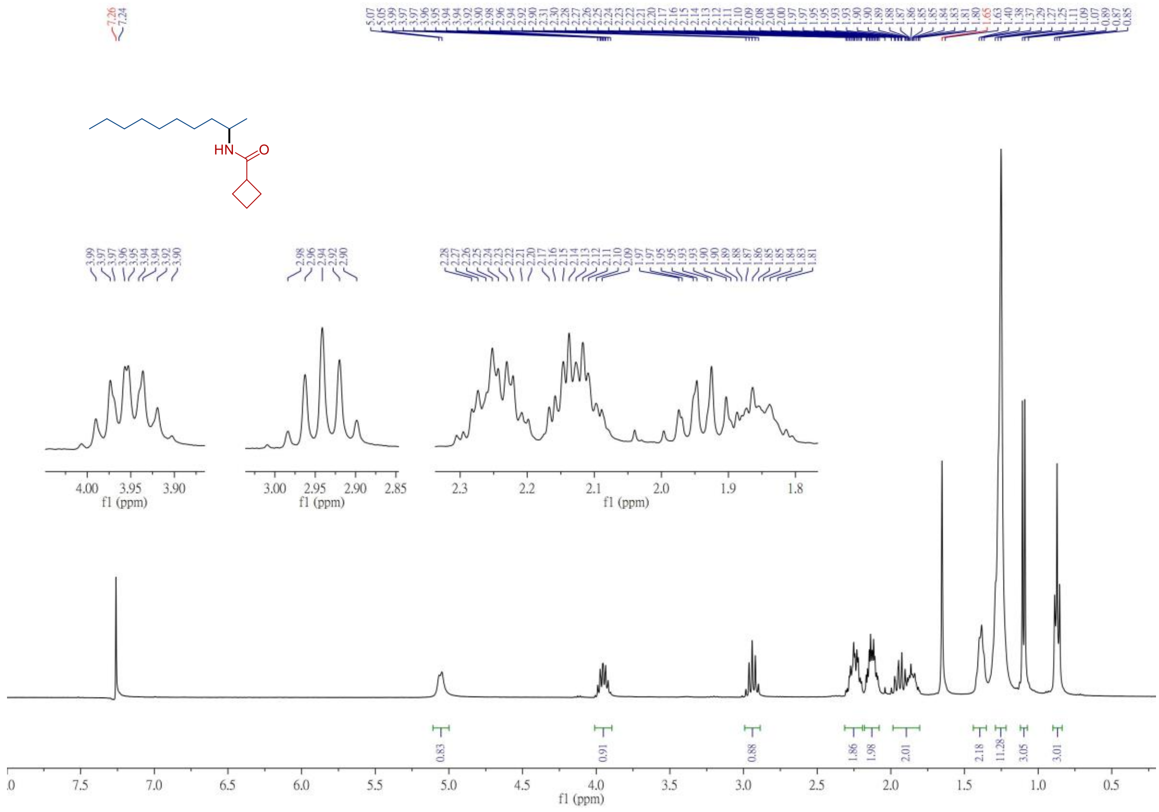

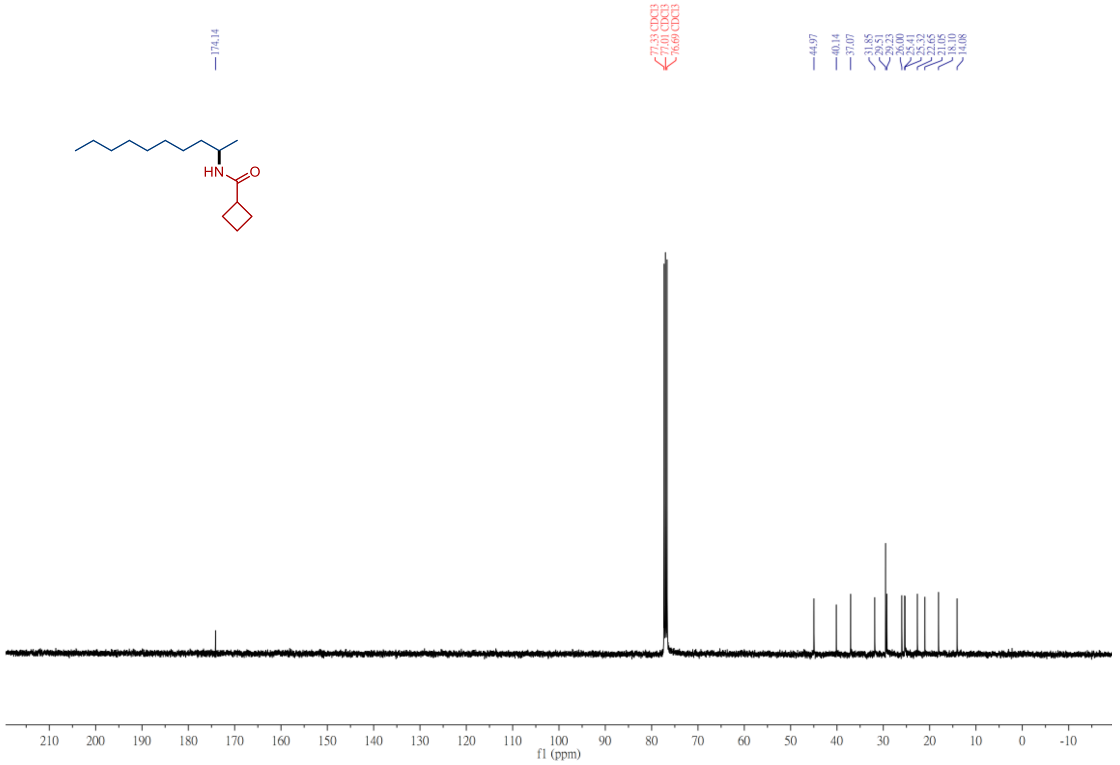

^{1}H and ^{13}C NMR spectrum of **S101**

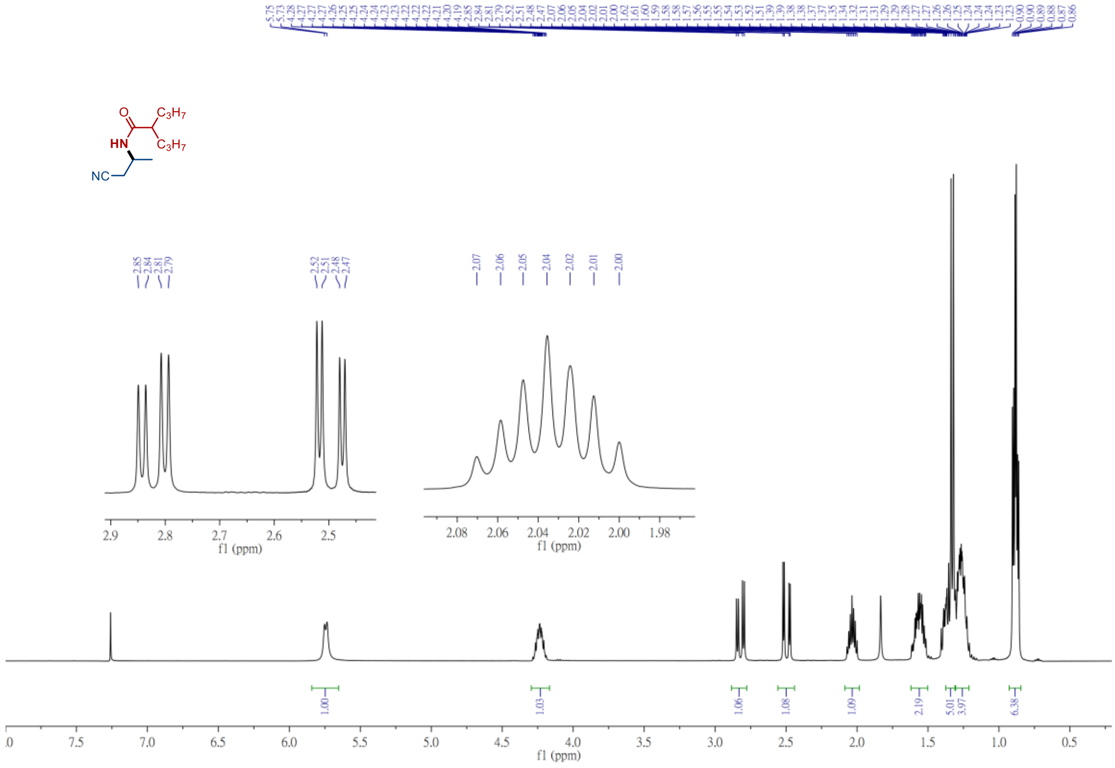

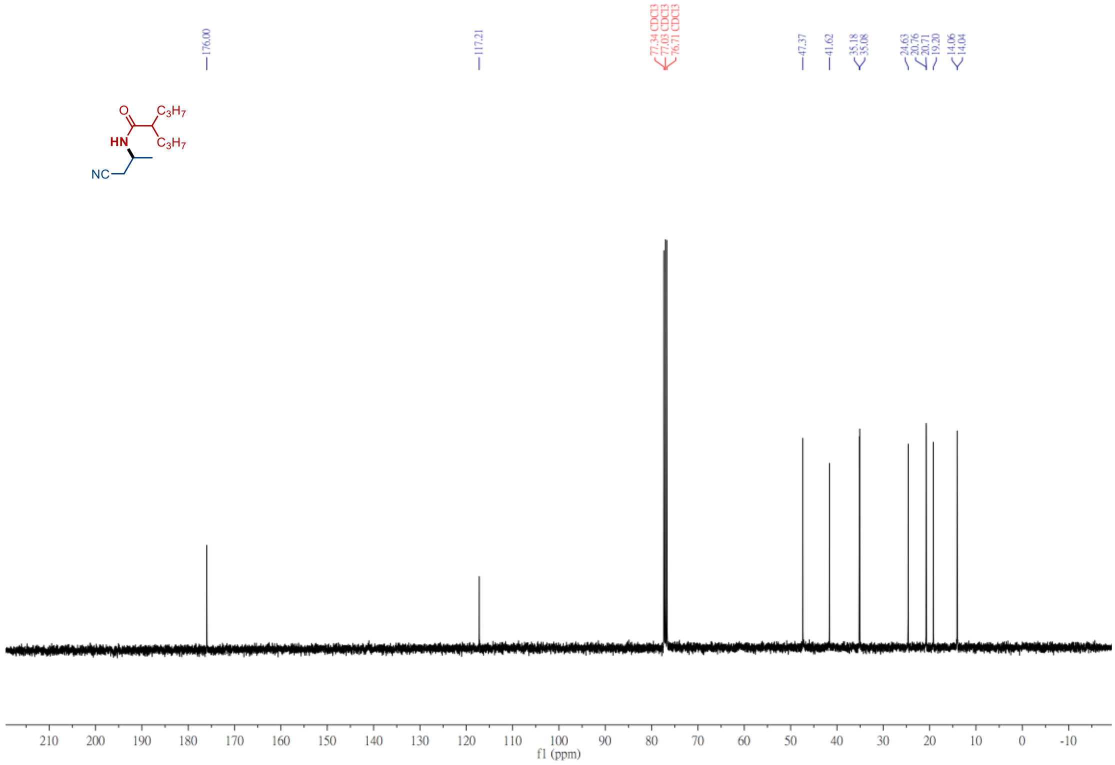

^{1}H and ^{13}C NMR spectrum of **S102**

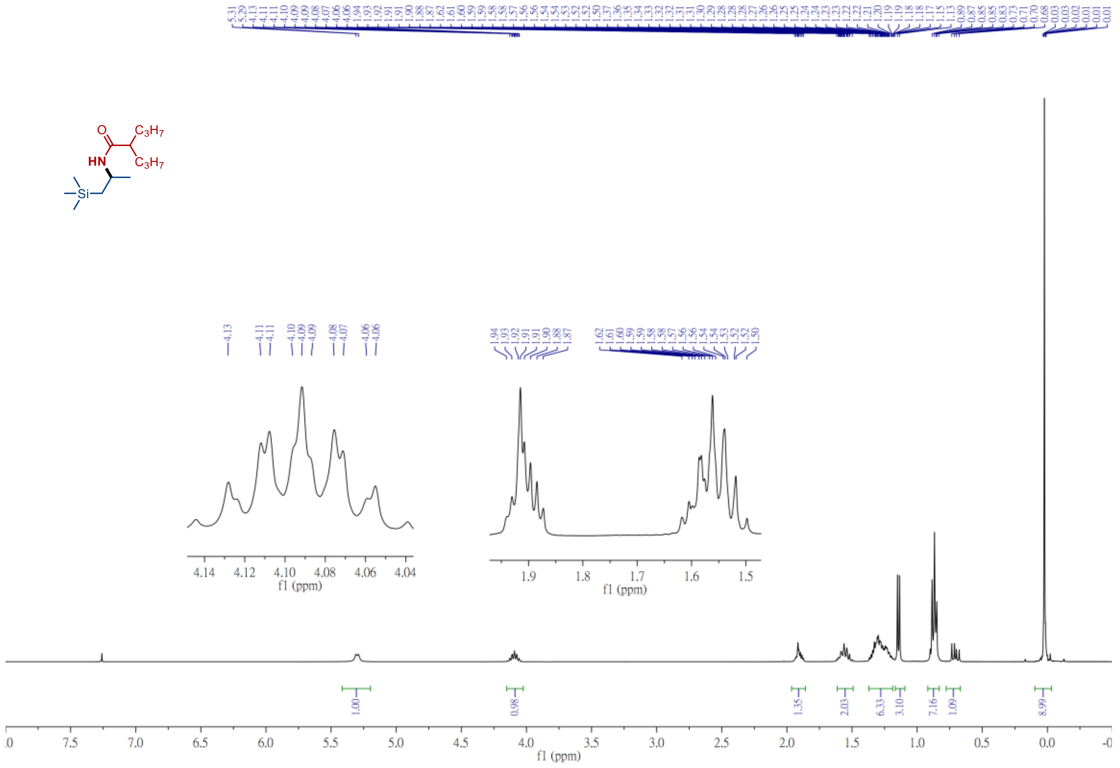

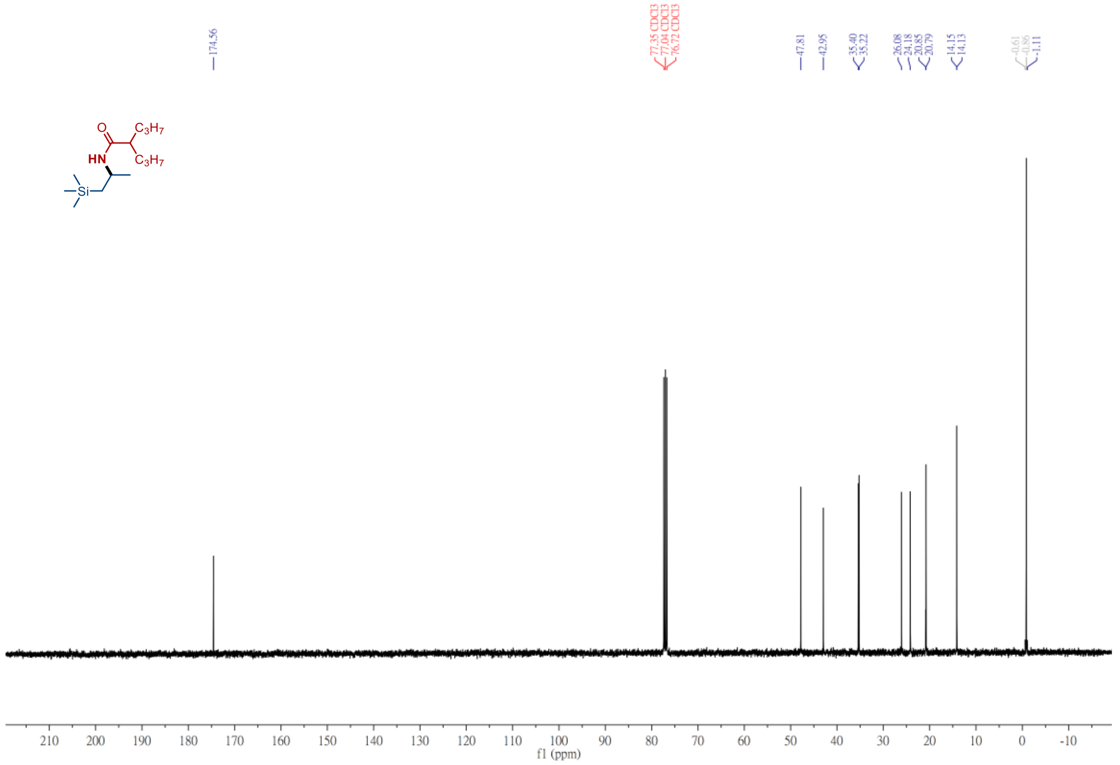

^{1}H and ^{13}C NMR spectrum of **S103**

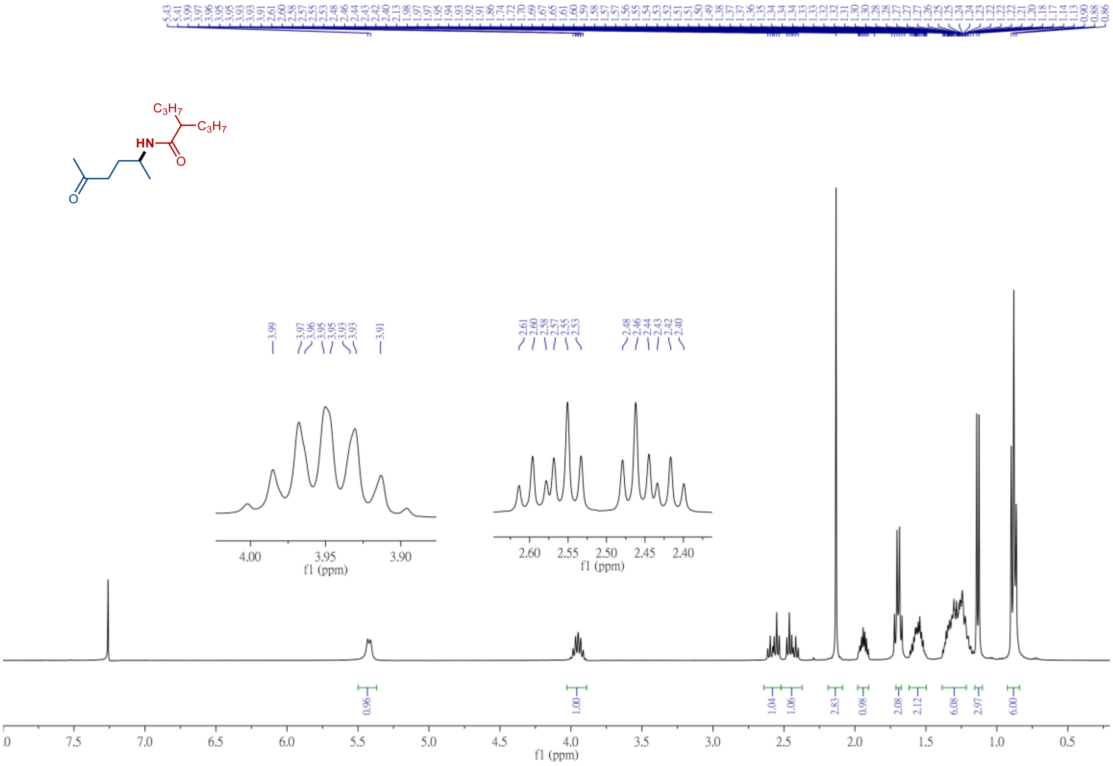

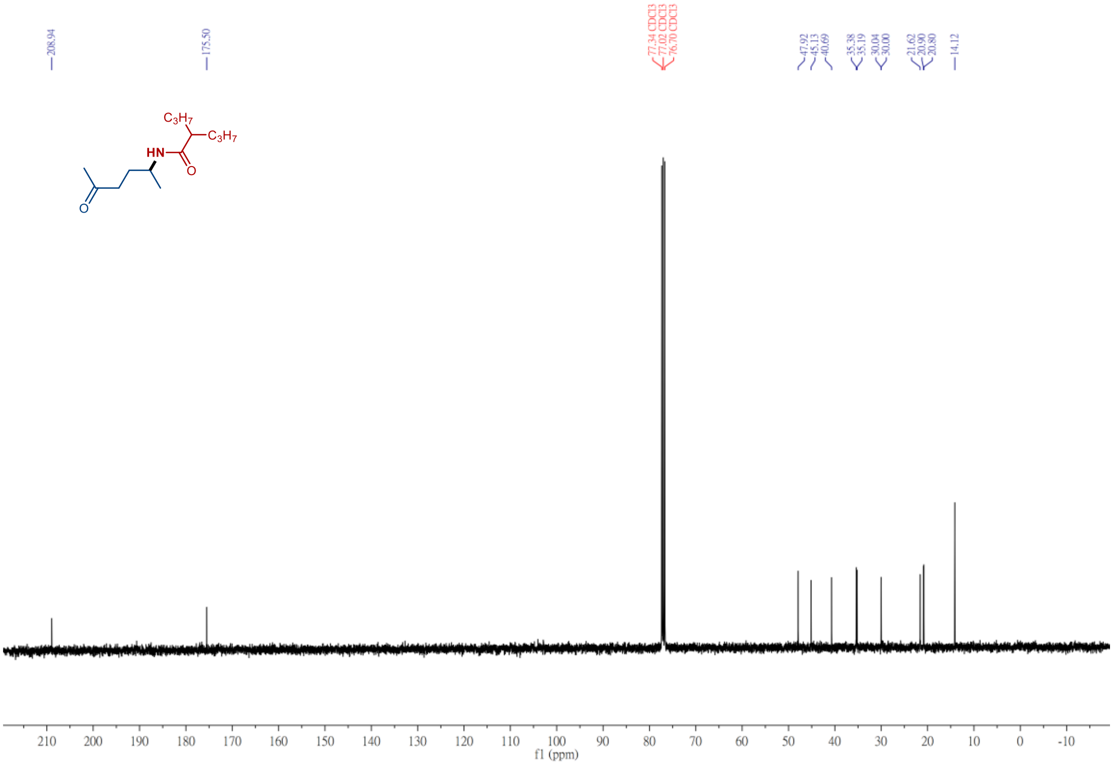

^{1}H and ^{13}C NMR spectrum of **S104**

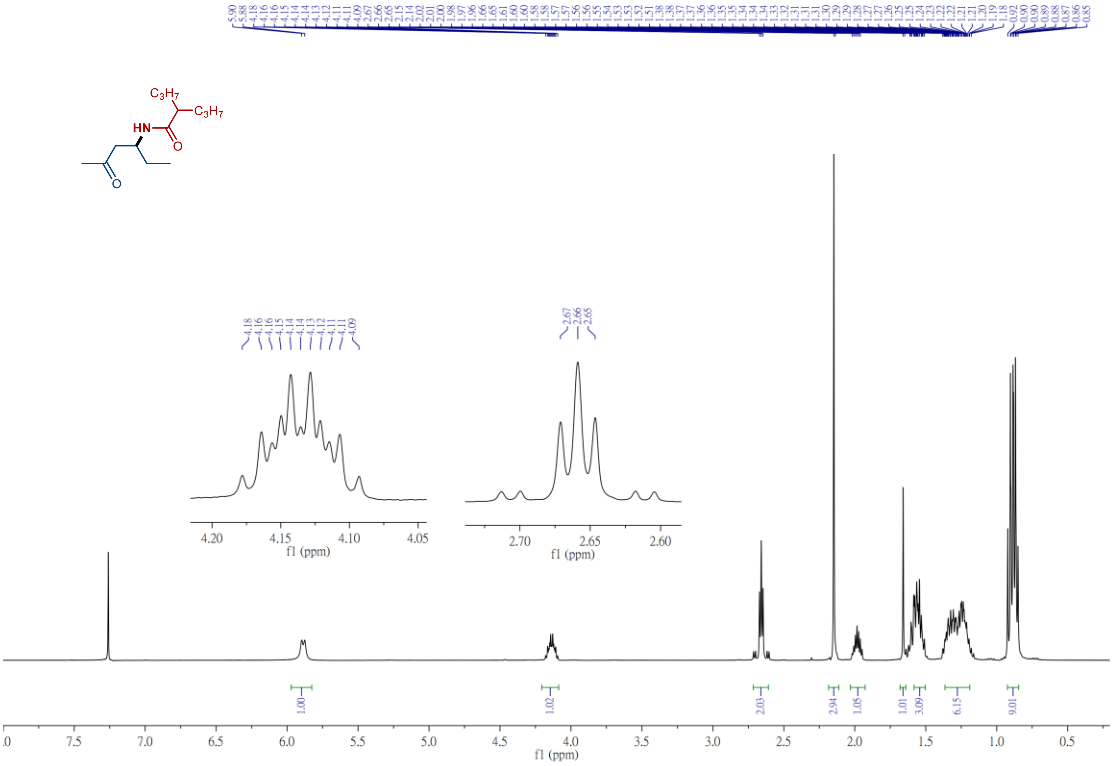

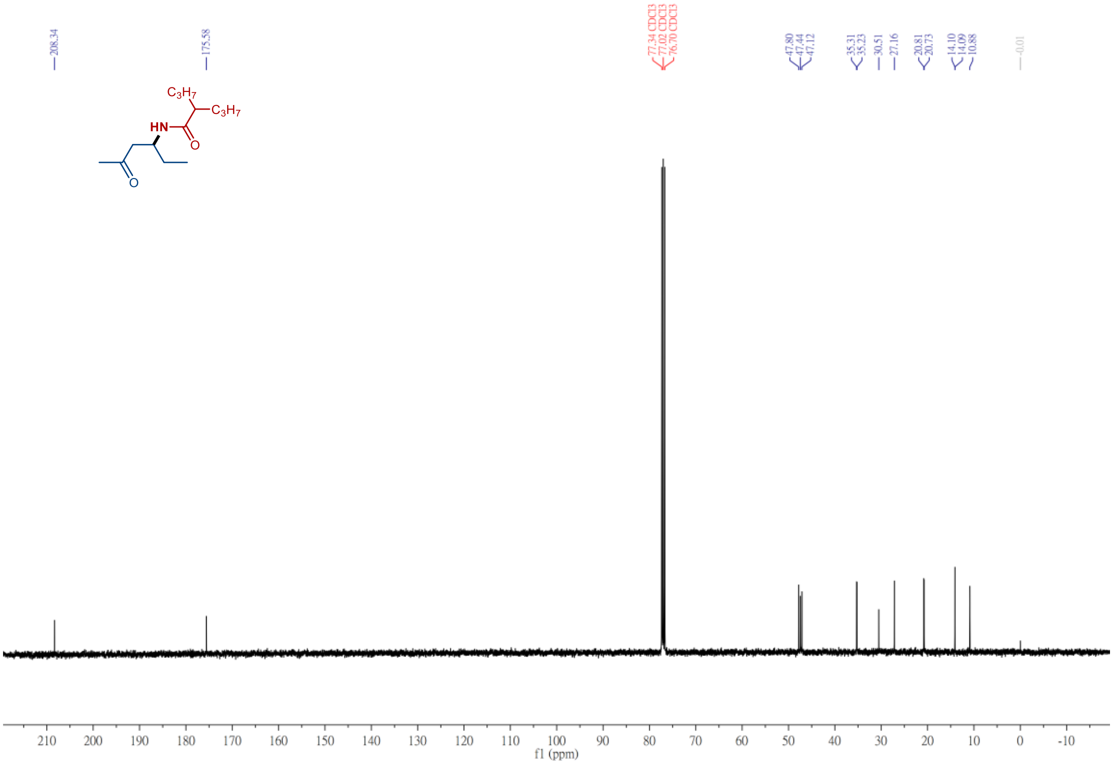

^{1}H and ^{13}C NMR spectrum of **S105**

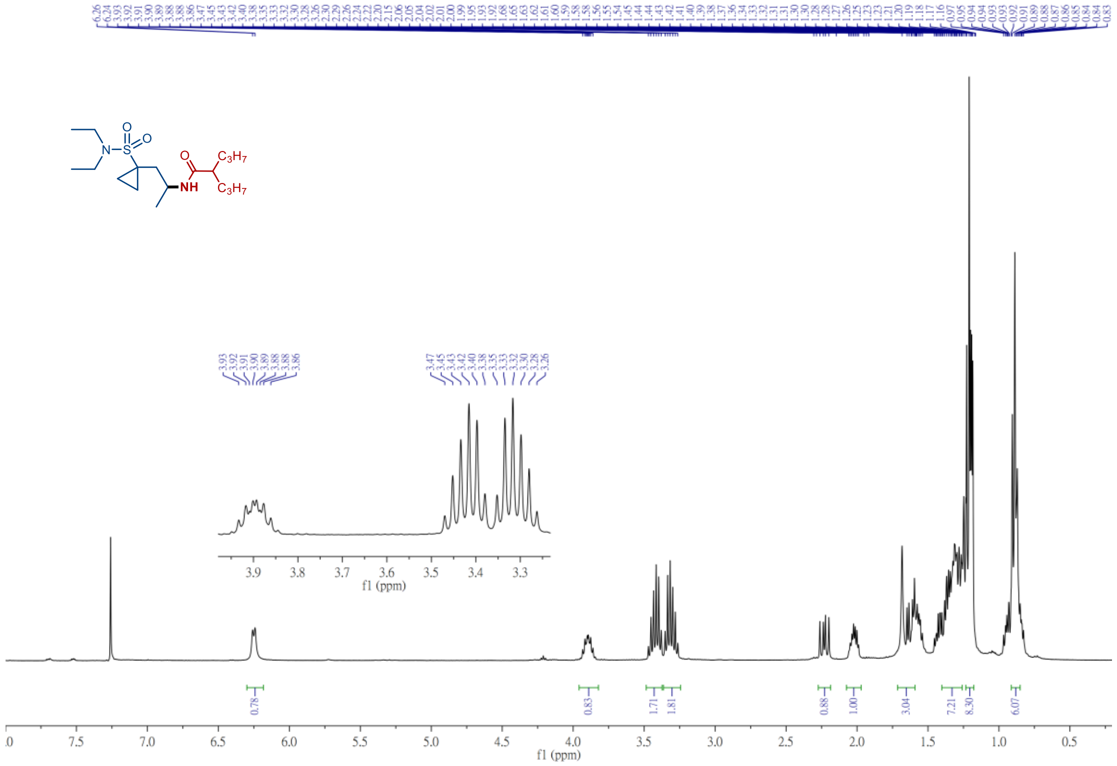

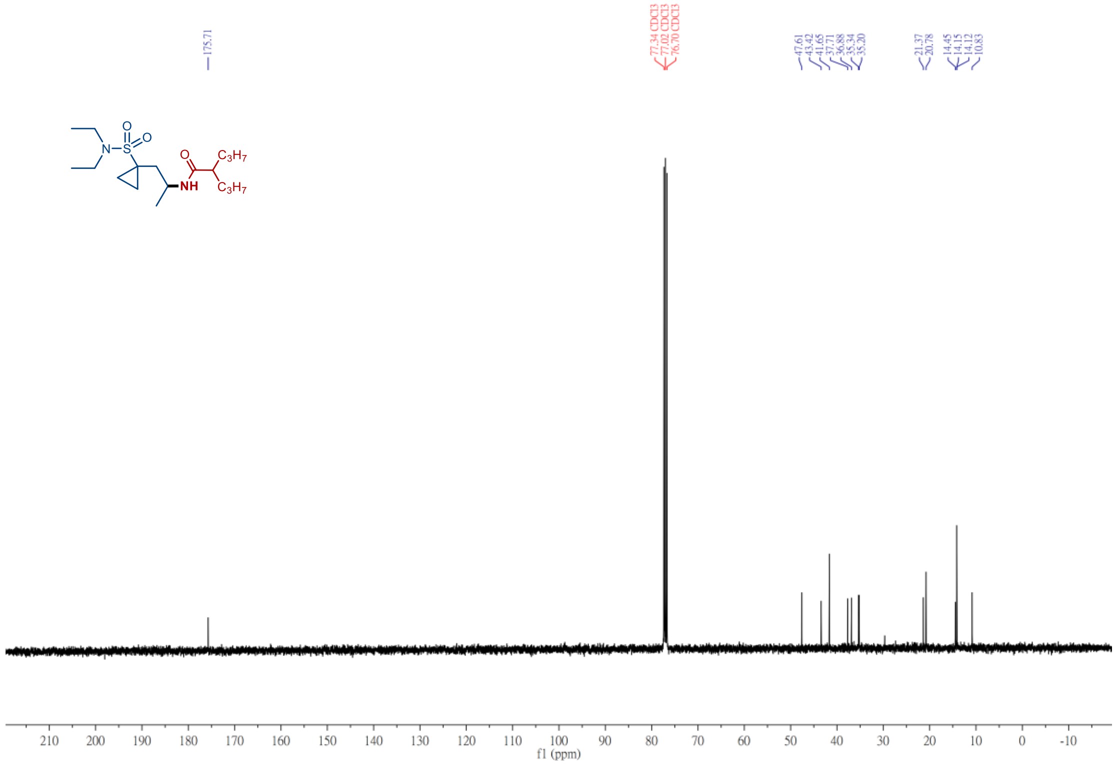

^{1}H and ^{13}C NMR spectrum of **S106**

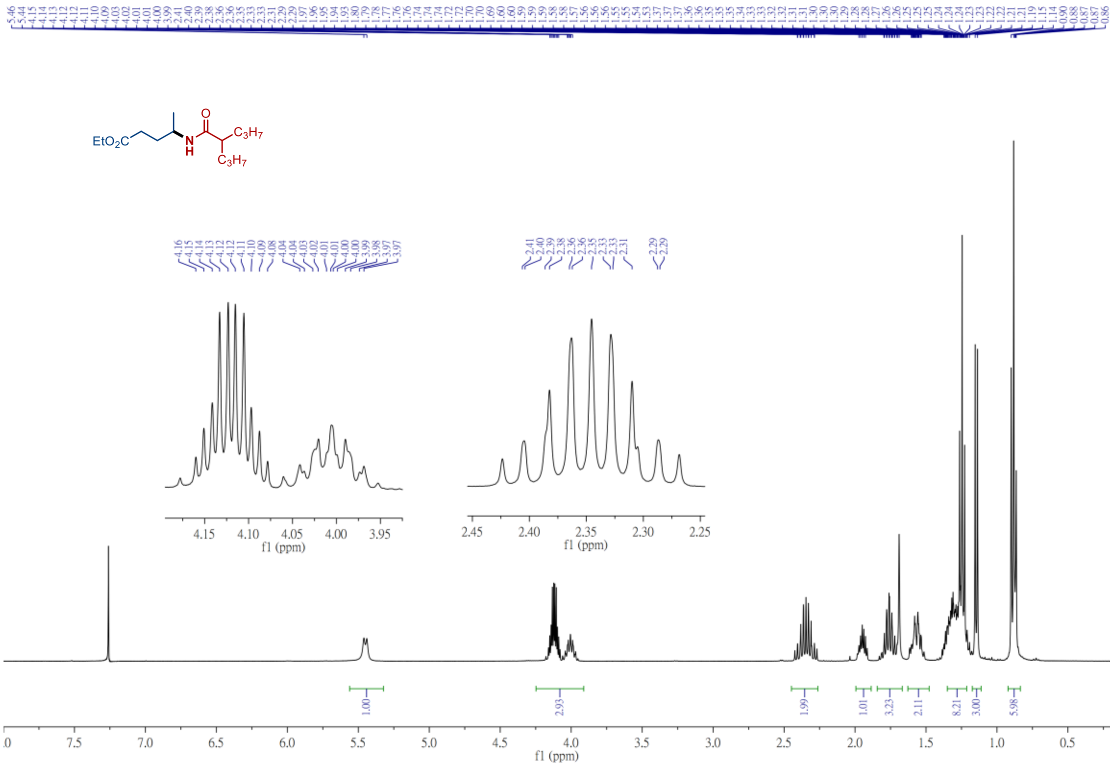

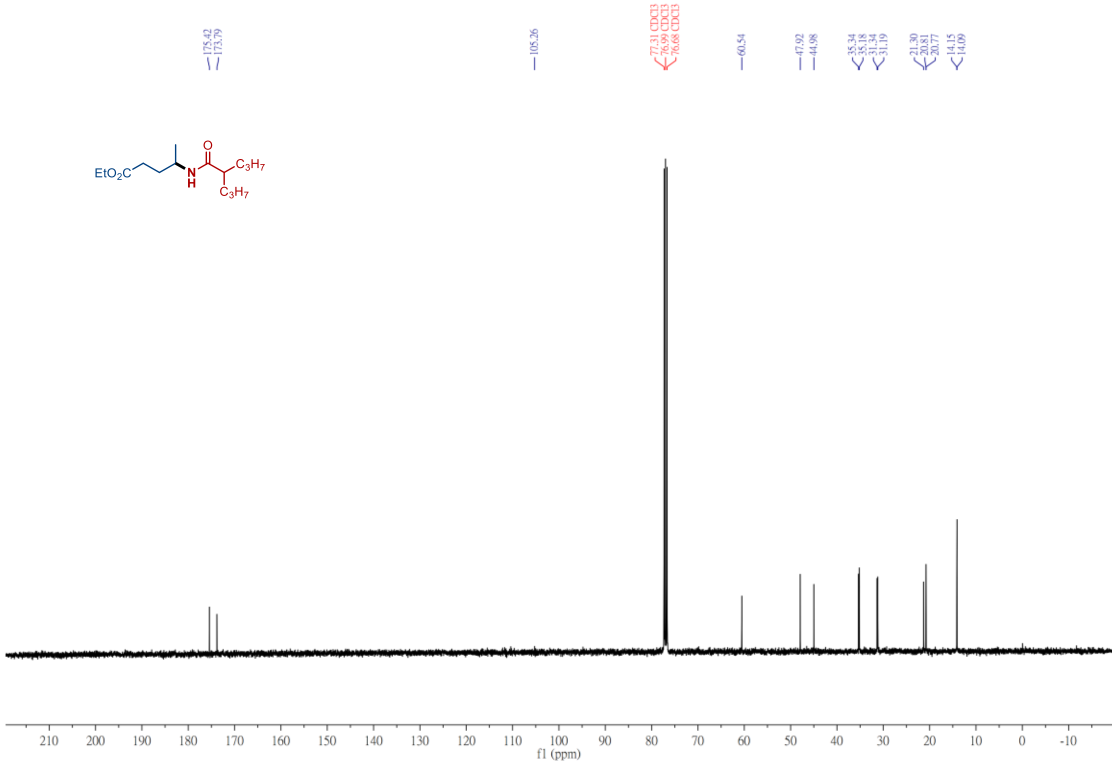

^{1}H and ^{13}C NMR spectrum of **S111**

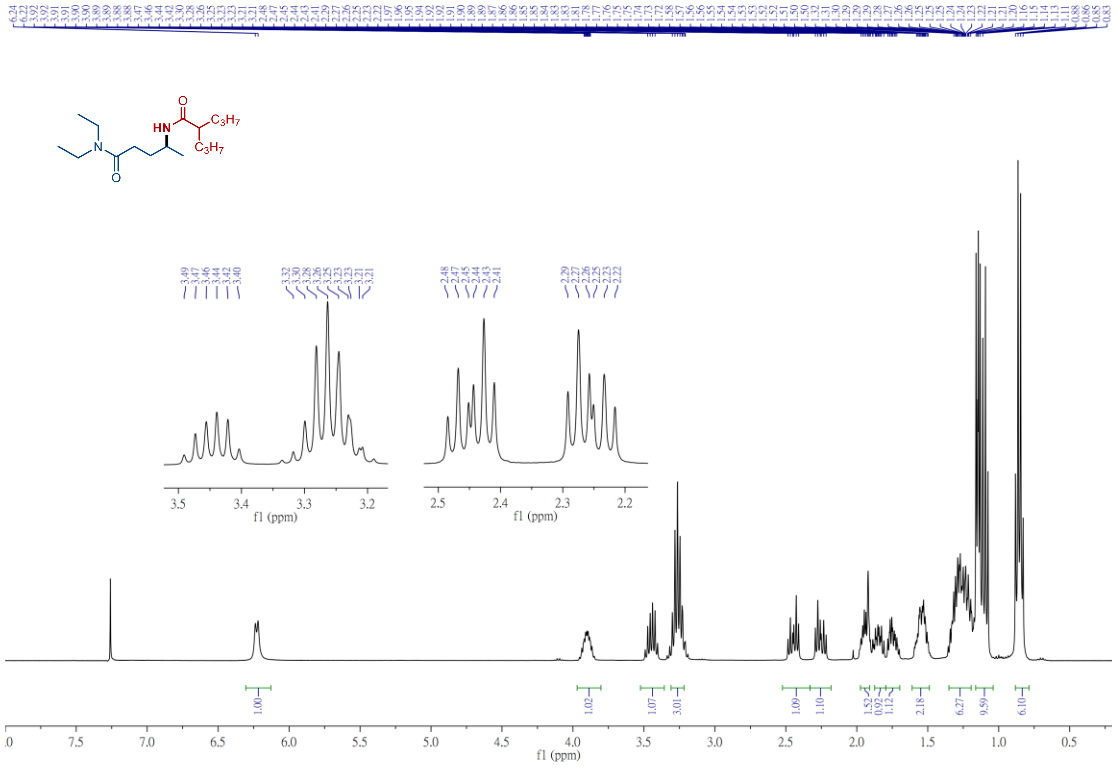

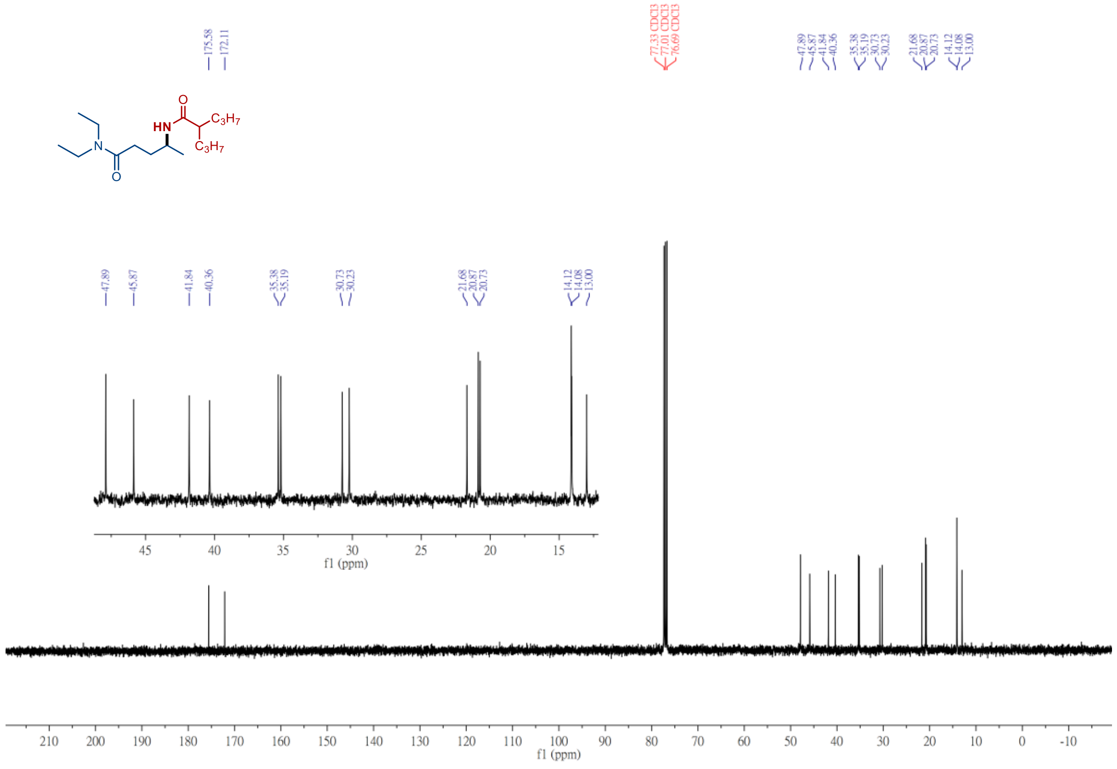

^{1}H and ^{13}C NMR spectrum of **S114**

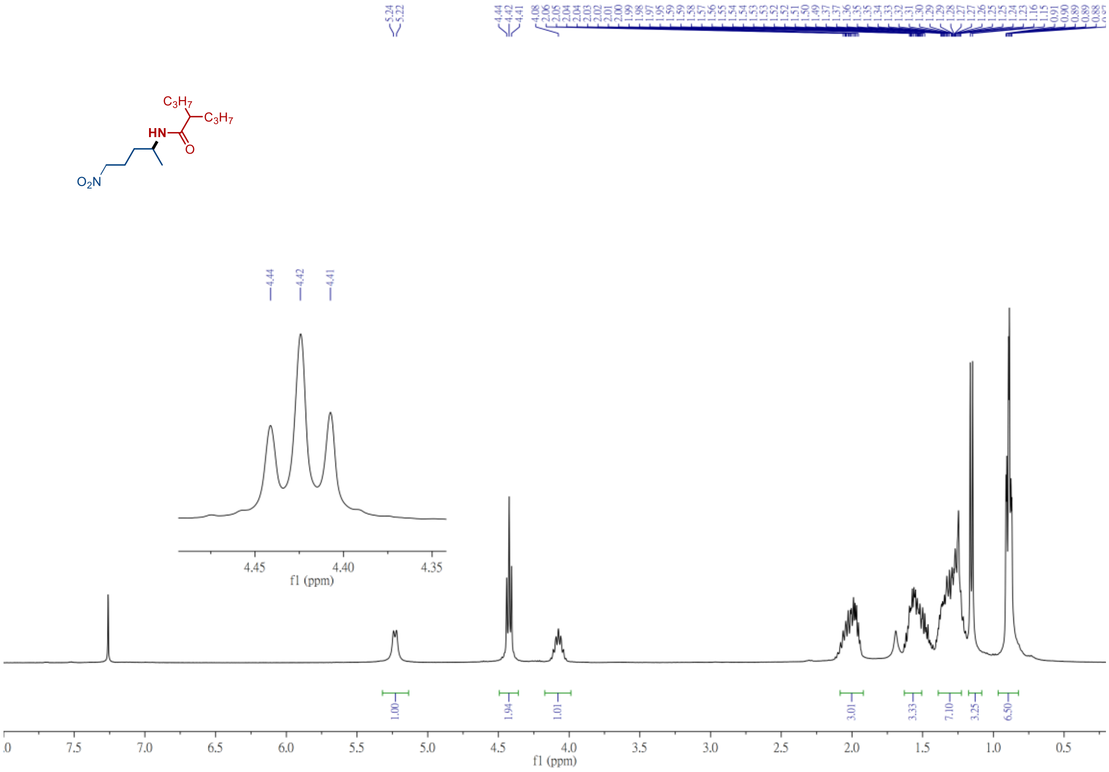

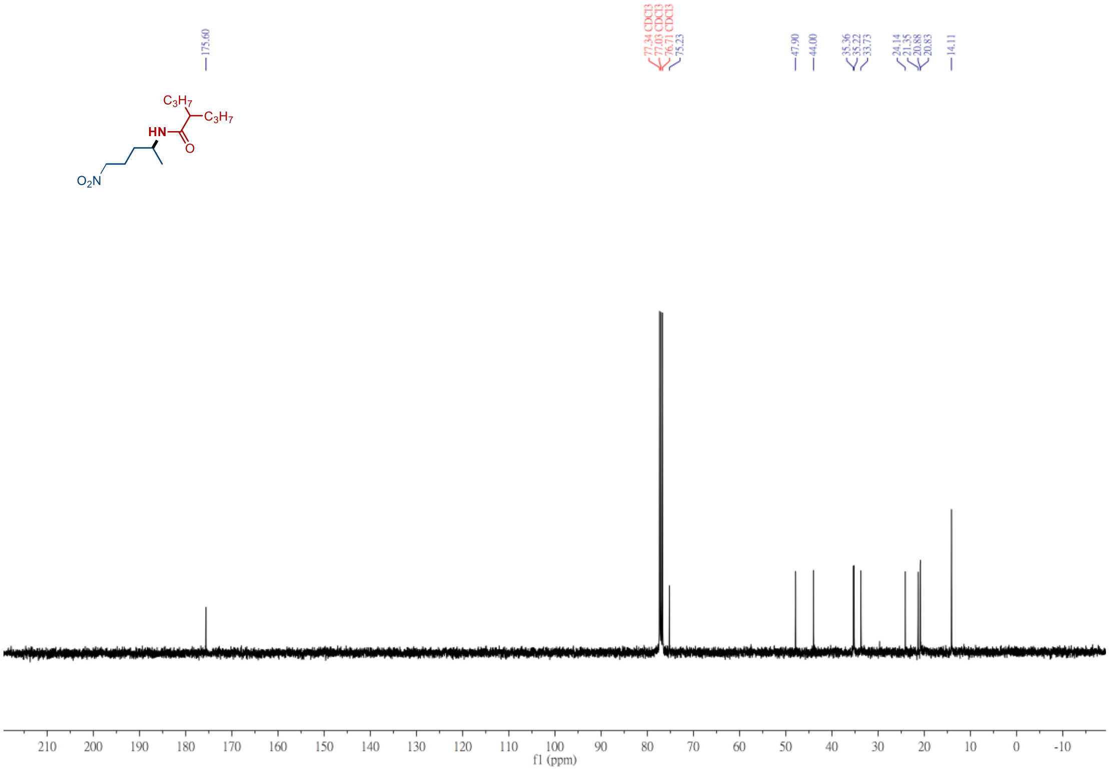

^{1}H and ^{13}C NMR spectrum of **S115**

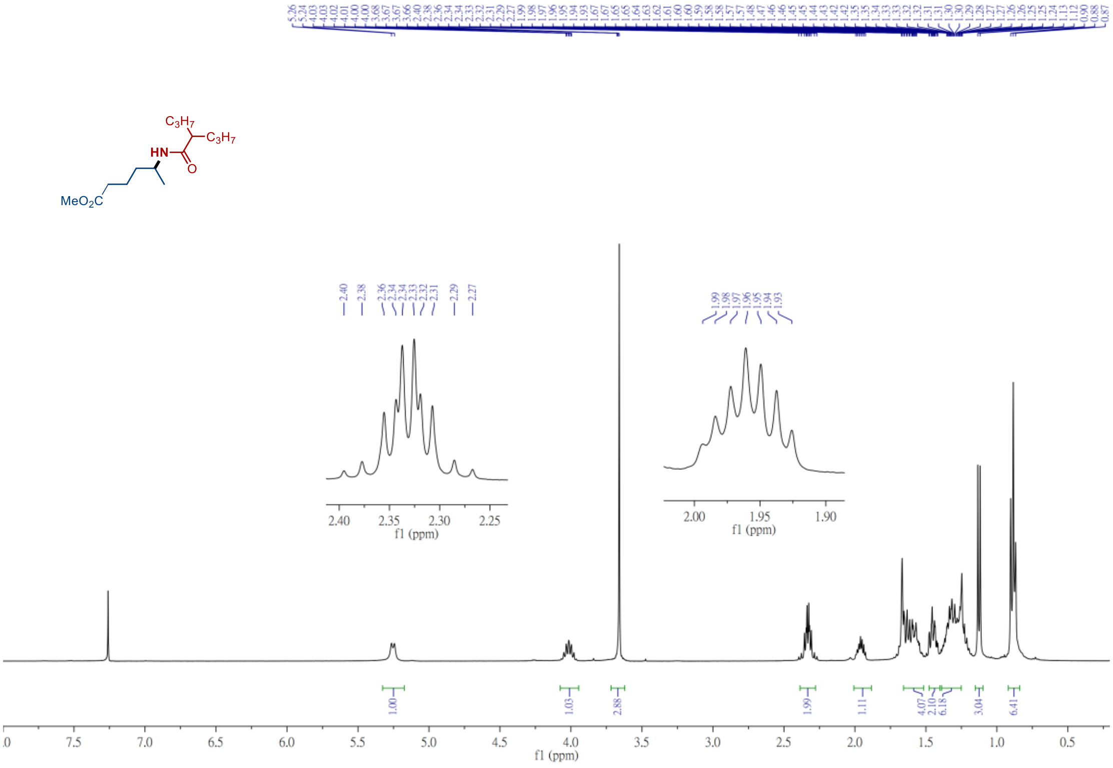

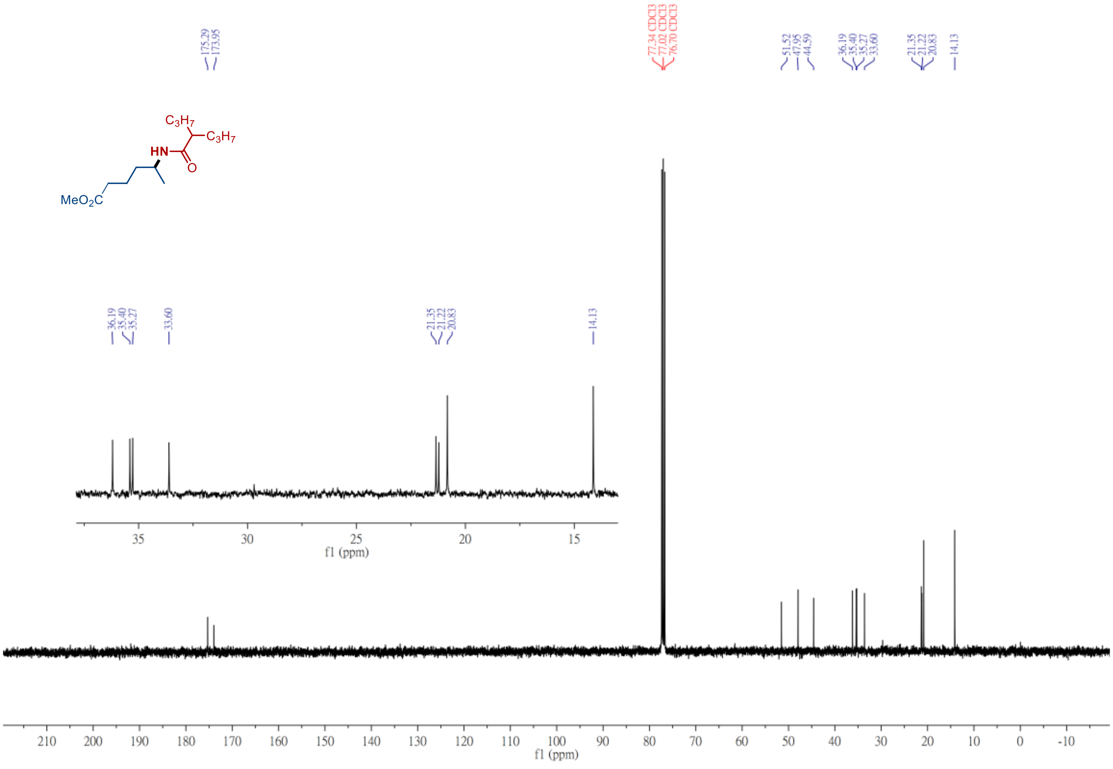

^{1}H and ^{13}C NMR spectrum of **S116**

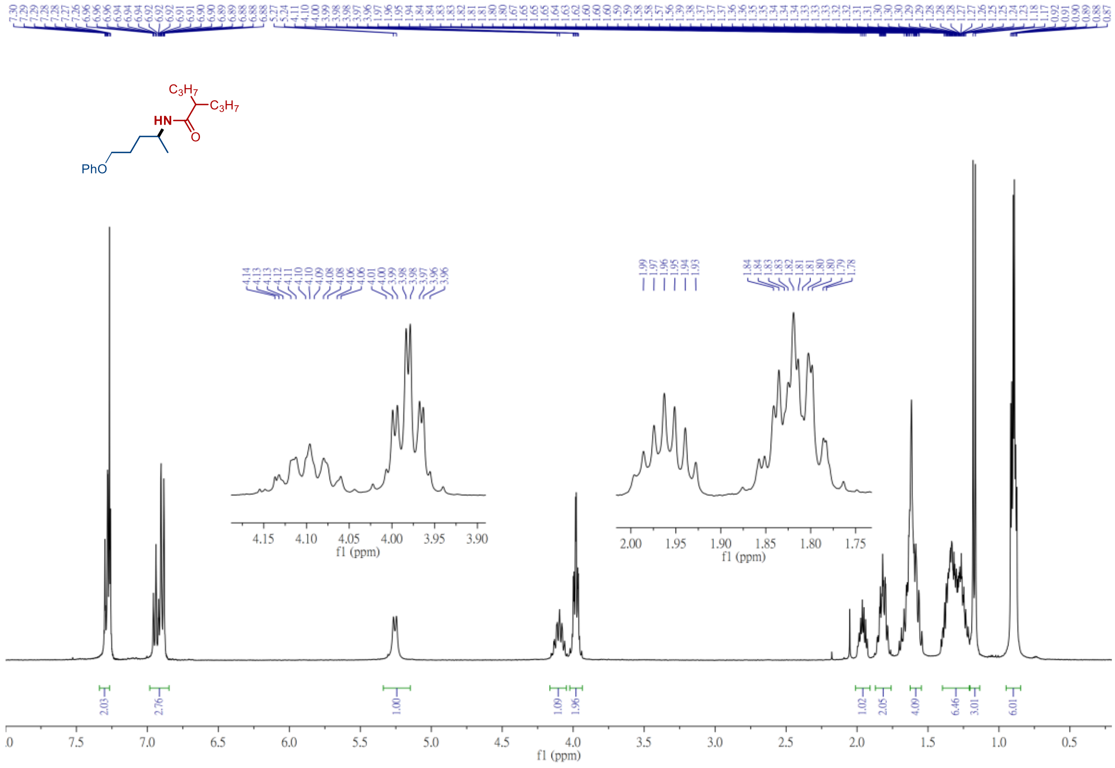

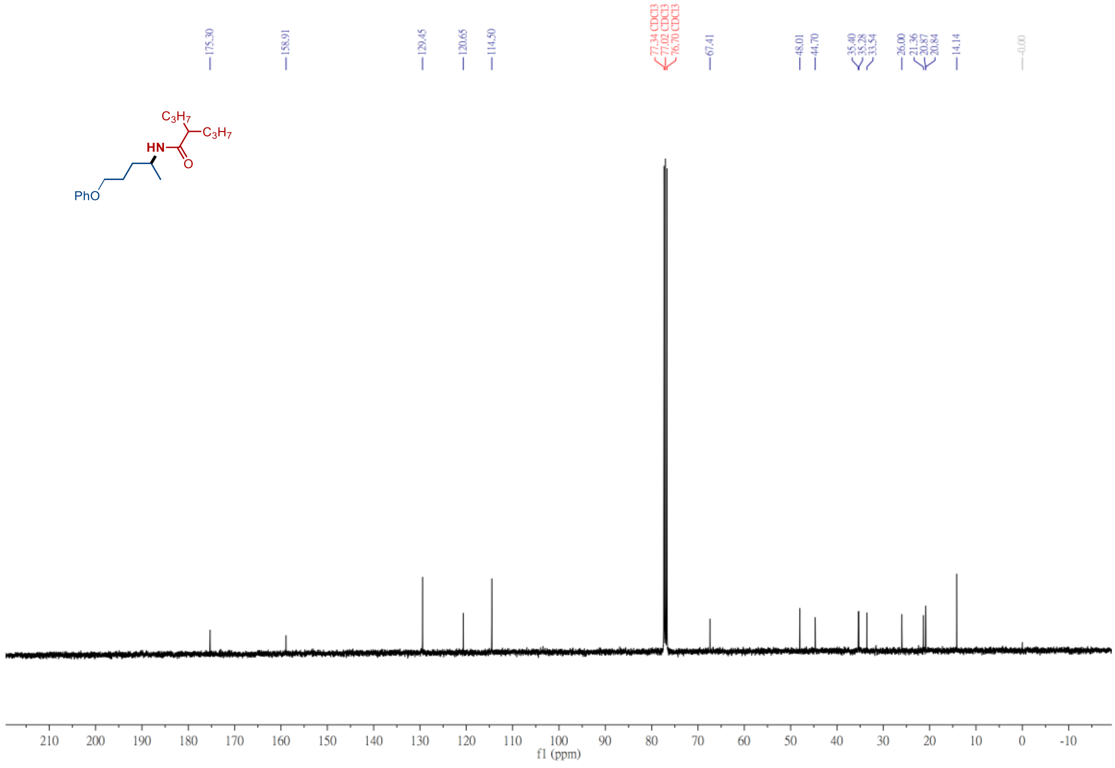

^{1}H and ^{13}C NMR spectrum of **S117**

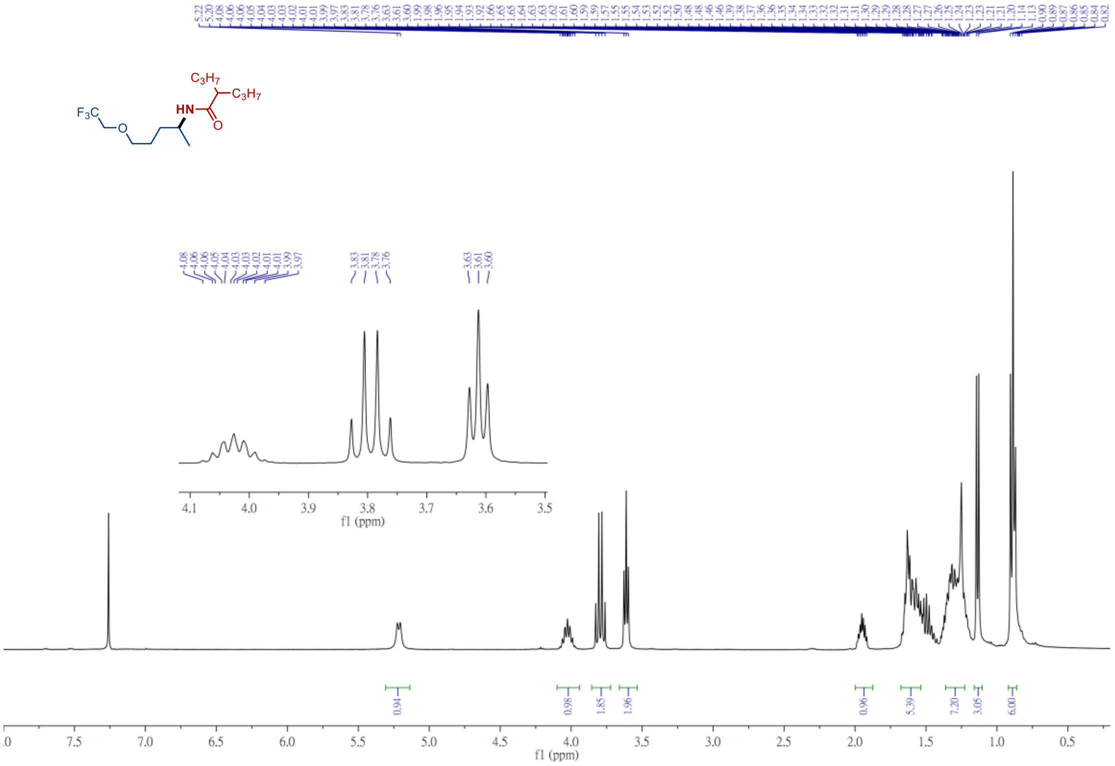

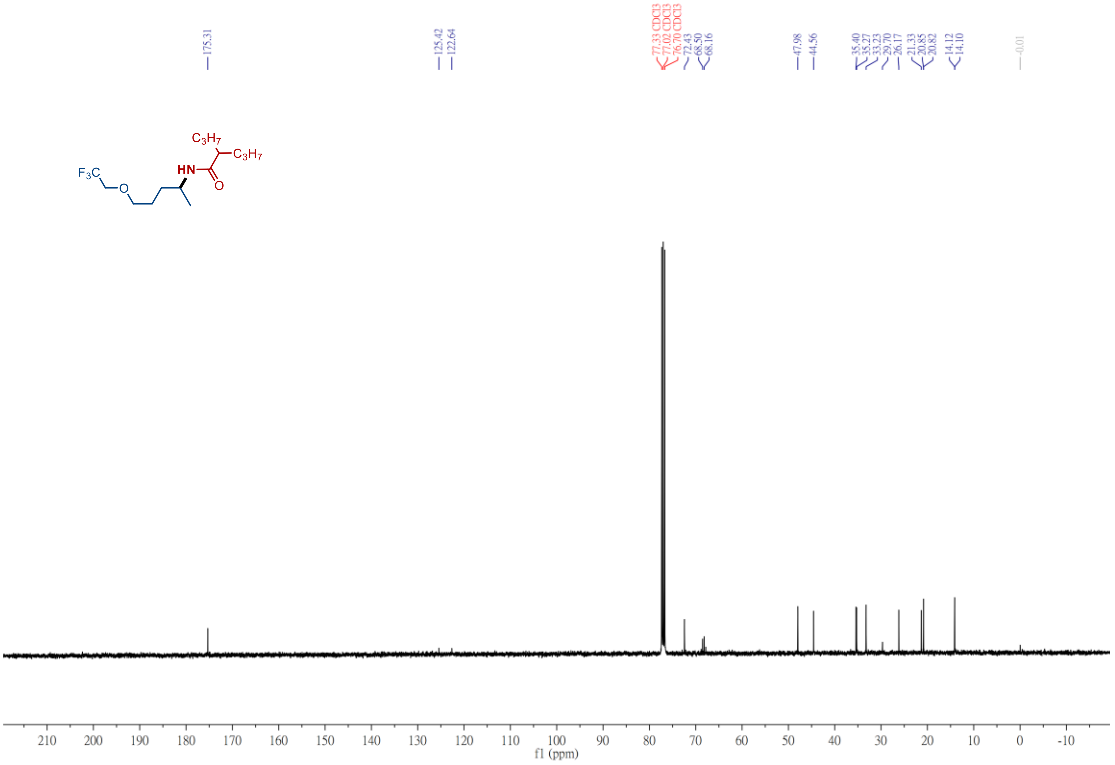

^{19}F NMR spectrum of **S117**

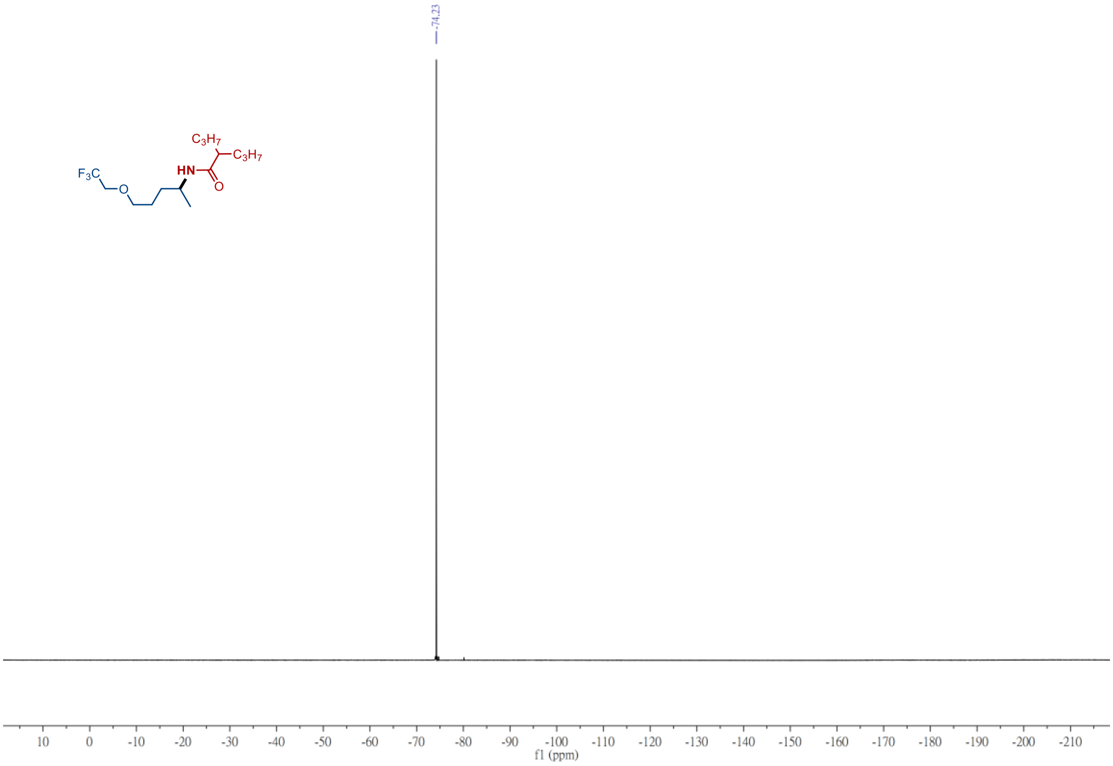

^{1}H and ^{13}C NMR spectrum of **S118**

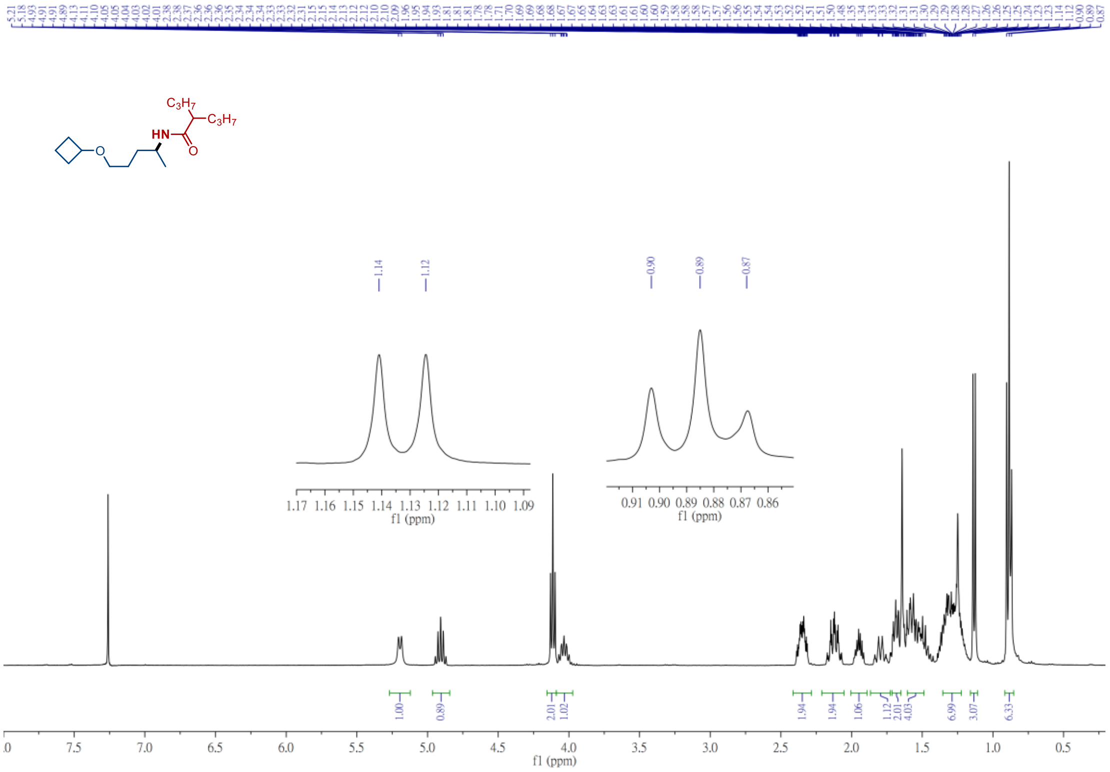

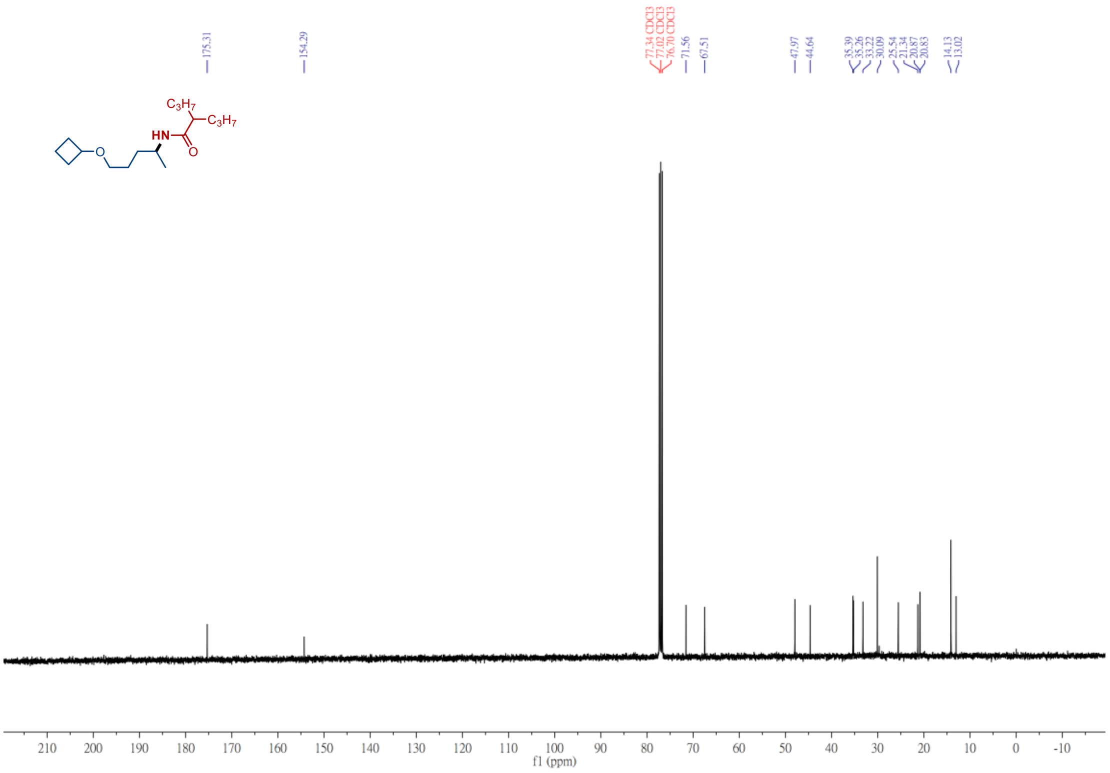

^{1}H and ^{13}C NMR spectrum of **S119**

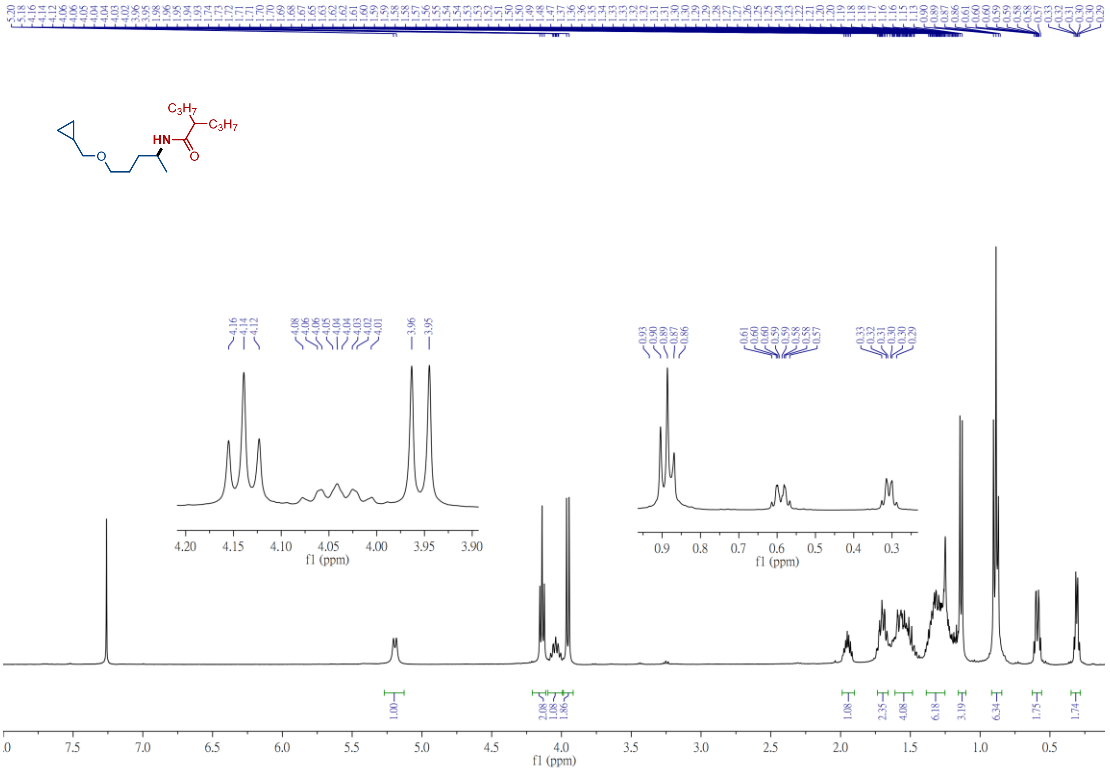

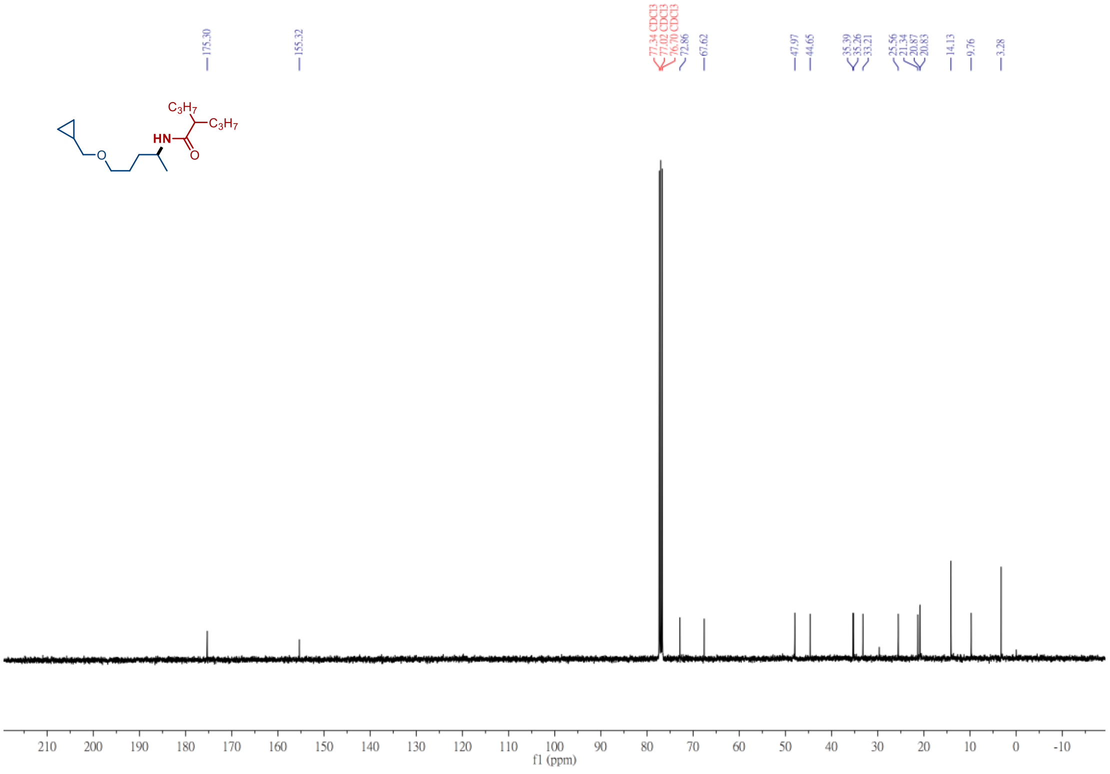

^{1}H and ^{13}C NMR spectrum of **S122**

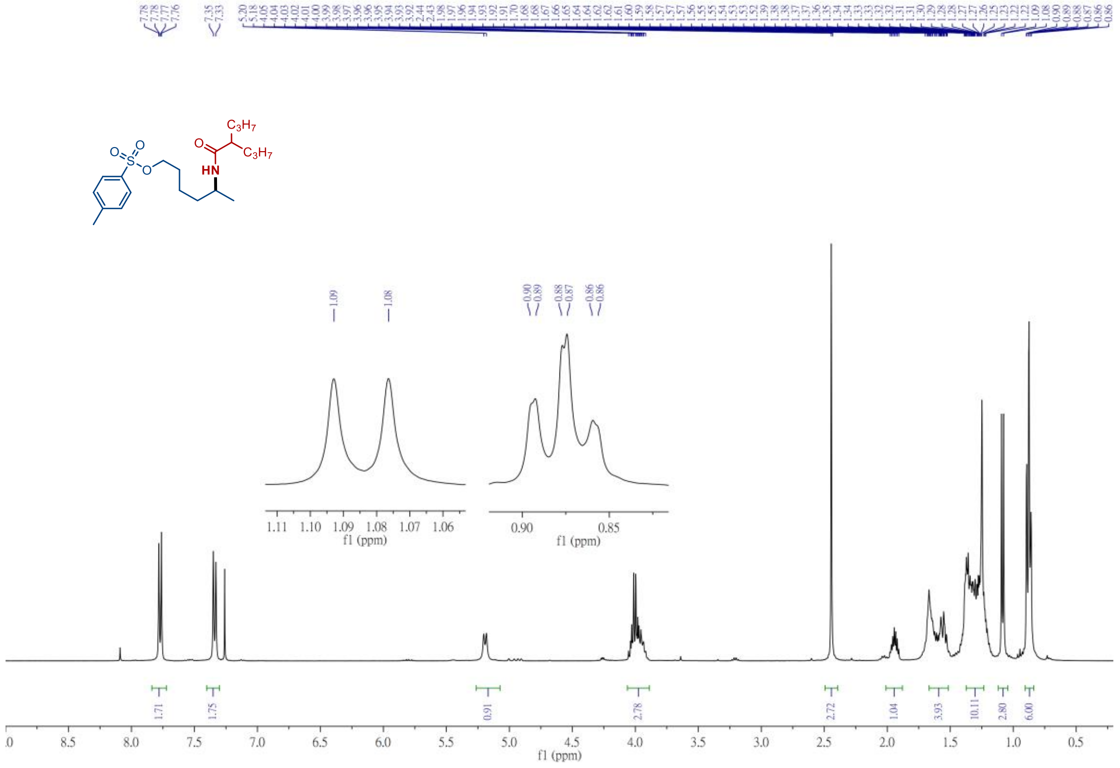

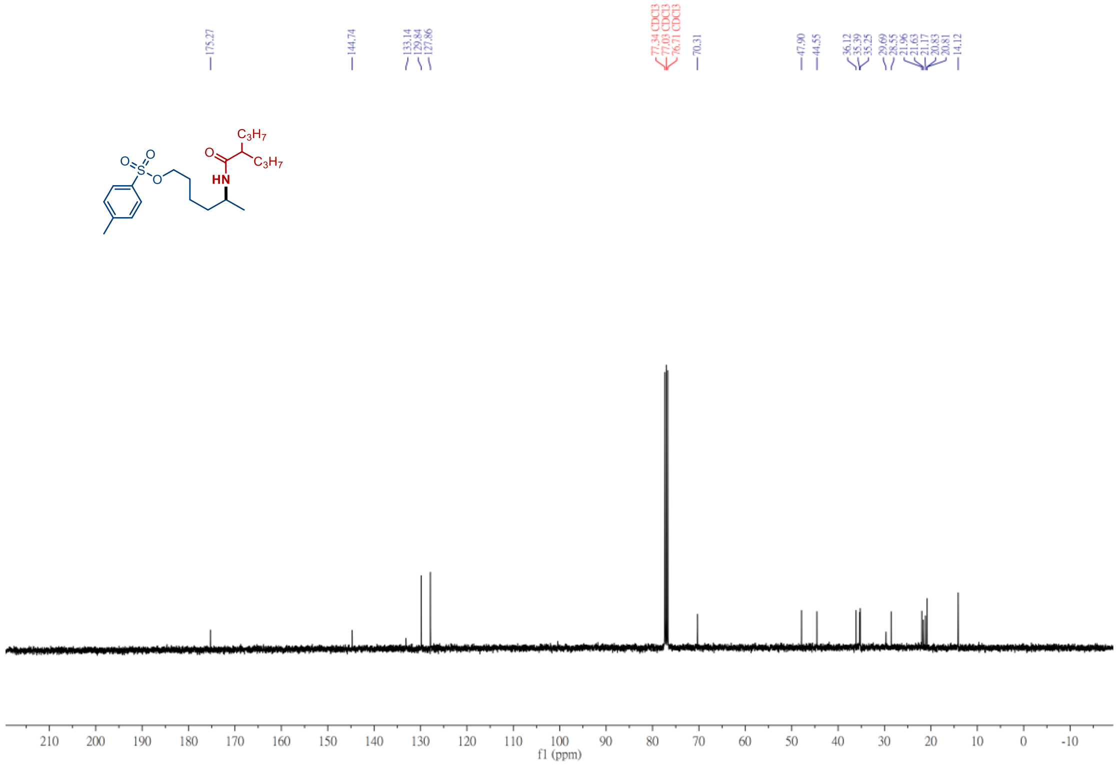

^{1}H and ^{13}C NMR spectrum of **S123**

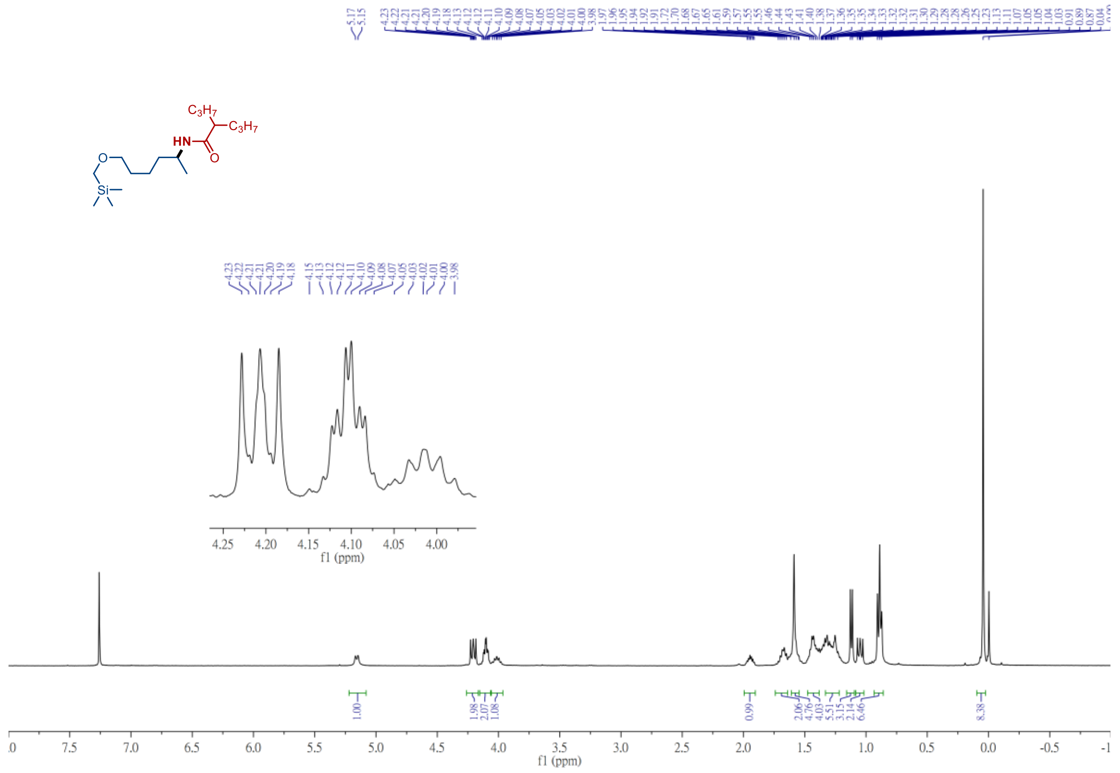

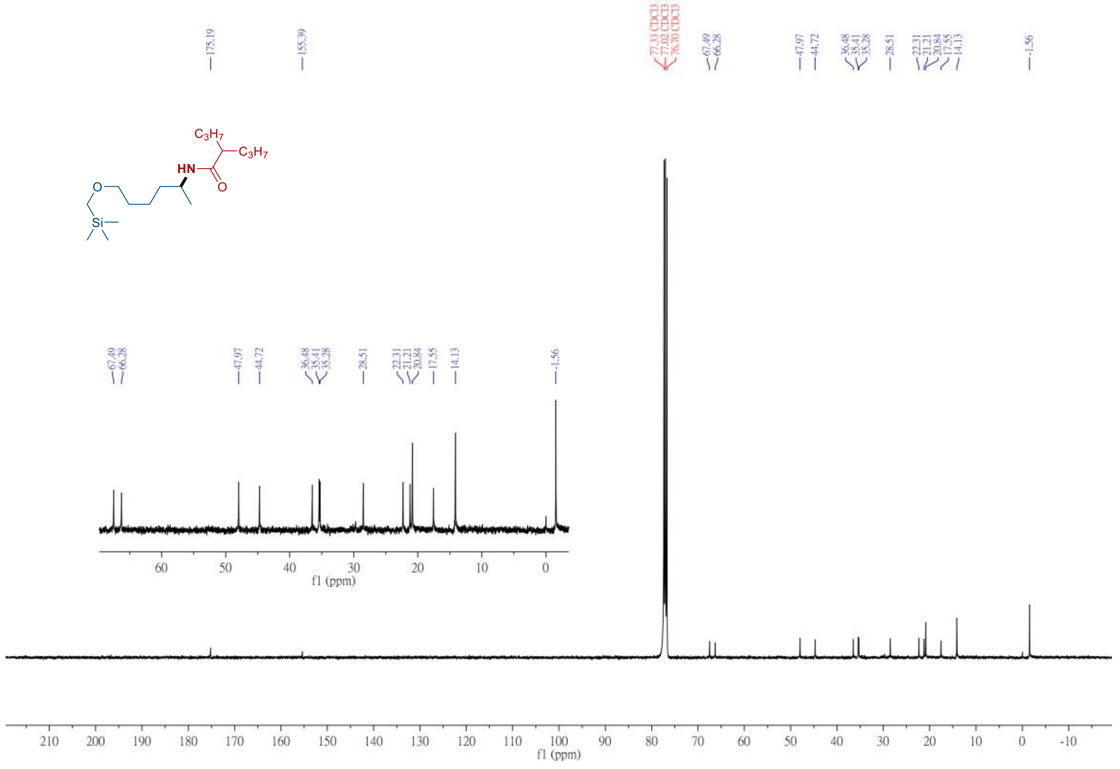

^{1}H and ^{13}C NMR spectrum of **S125**

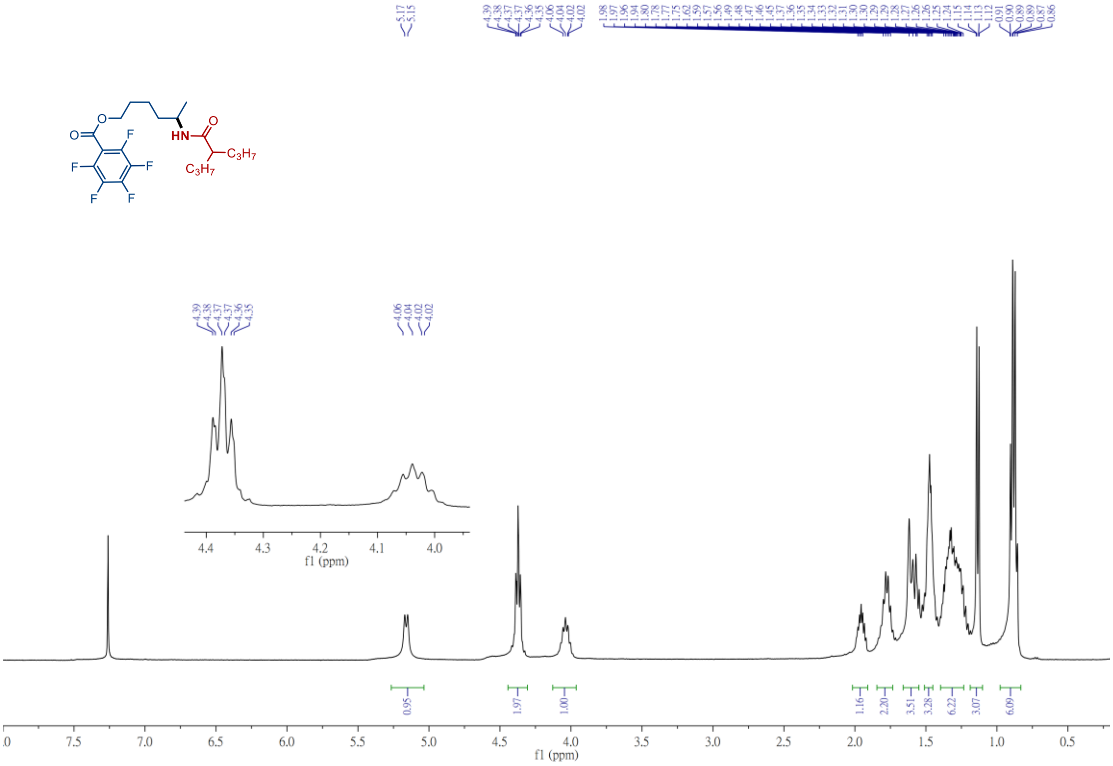

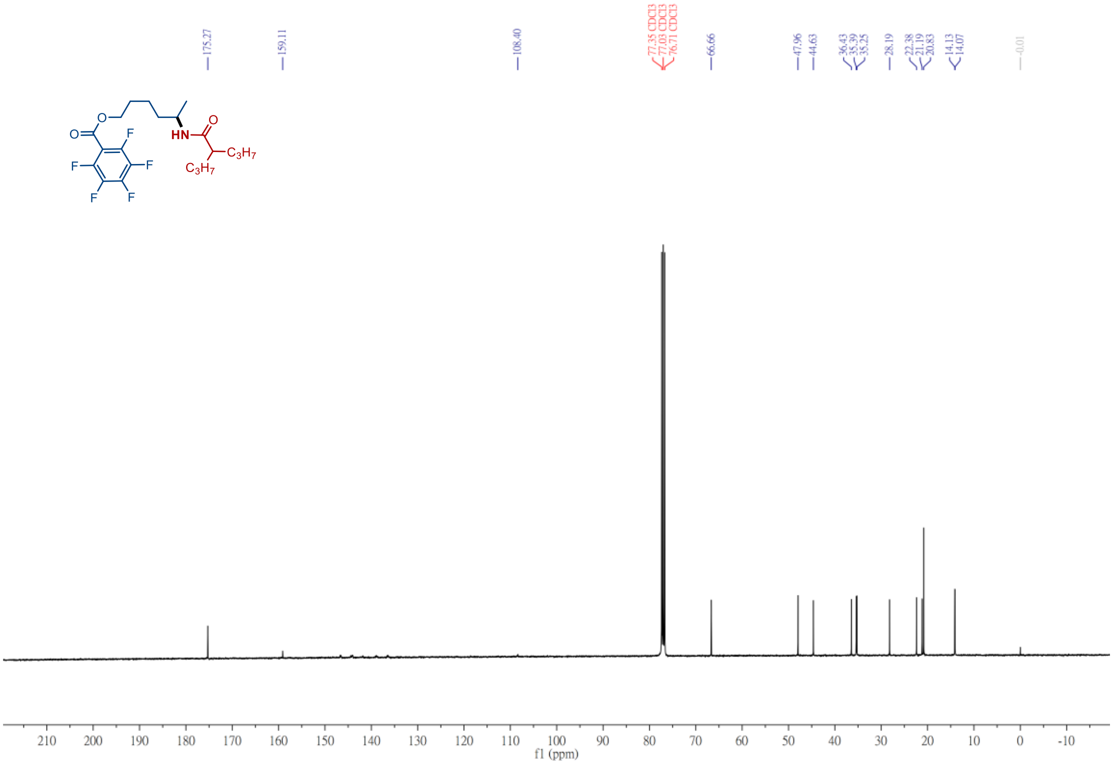

^{19}F NMR spectrum of **S125**

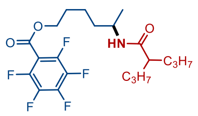

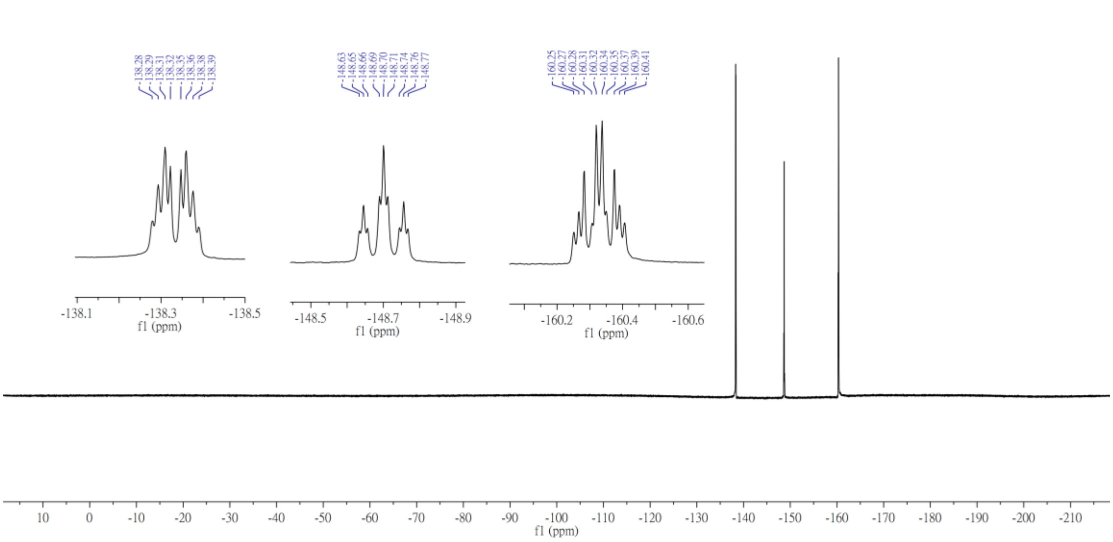

^{1}H and ^{13}C NMR spectrum of **S128**

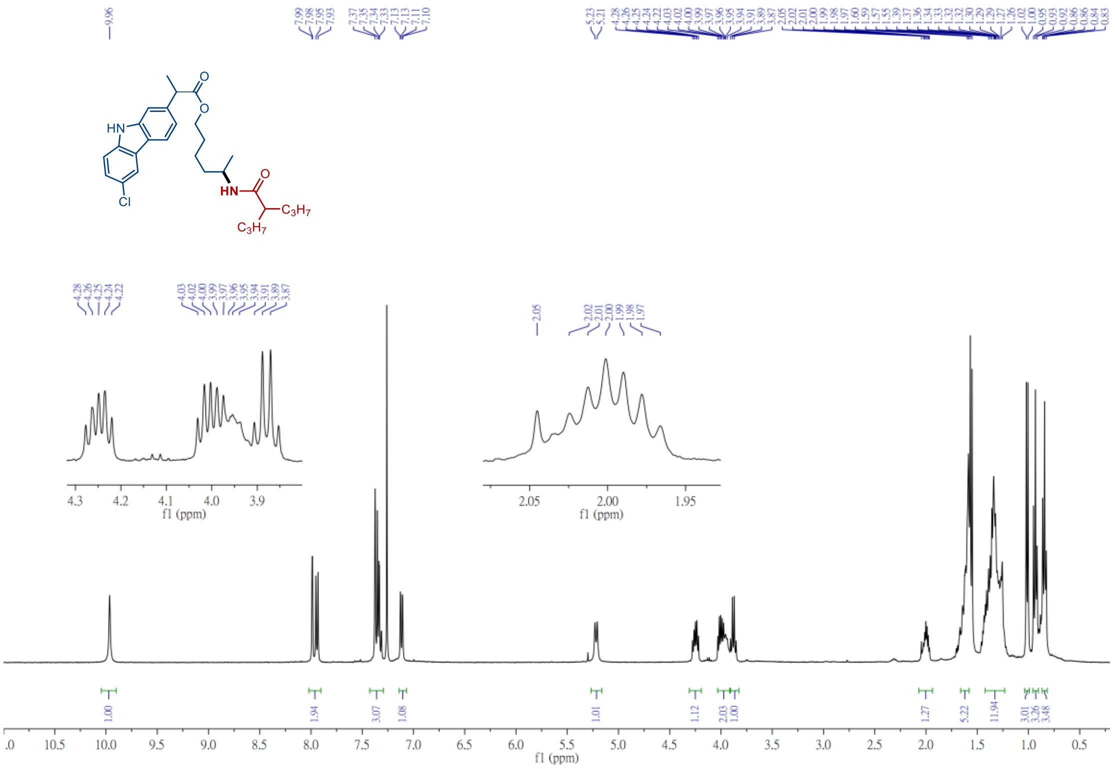

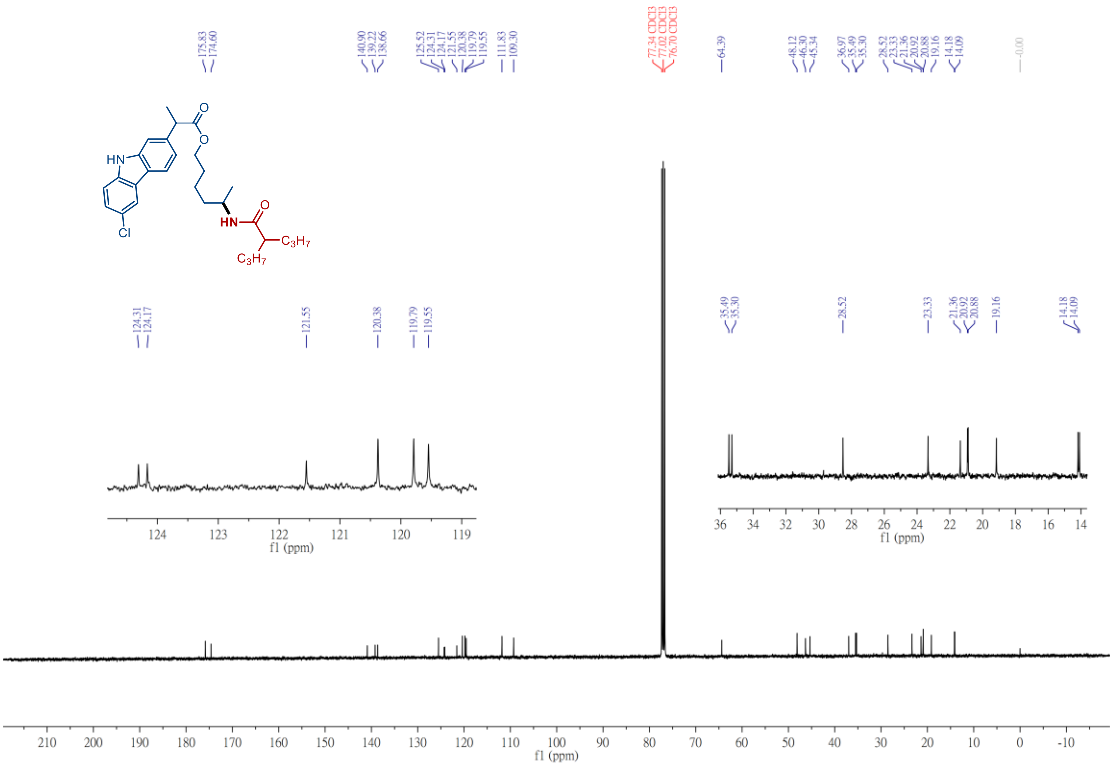

^{1}H and ^{13}C NMR spectrum of **95**

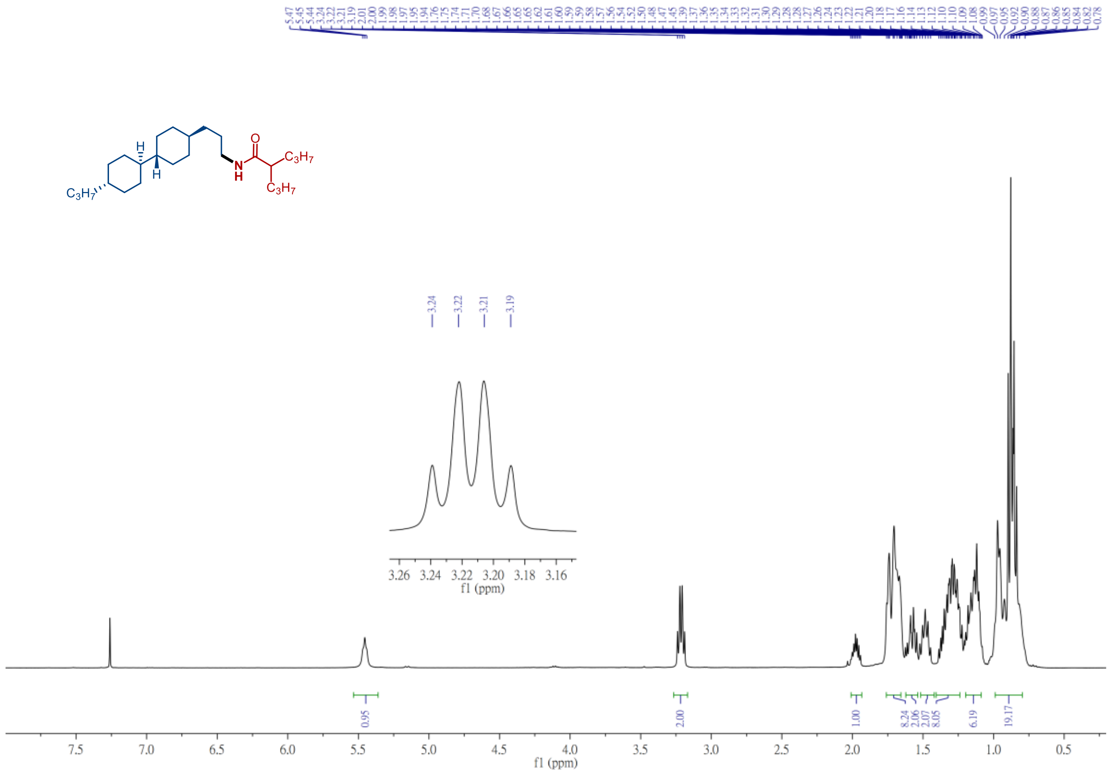

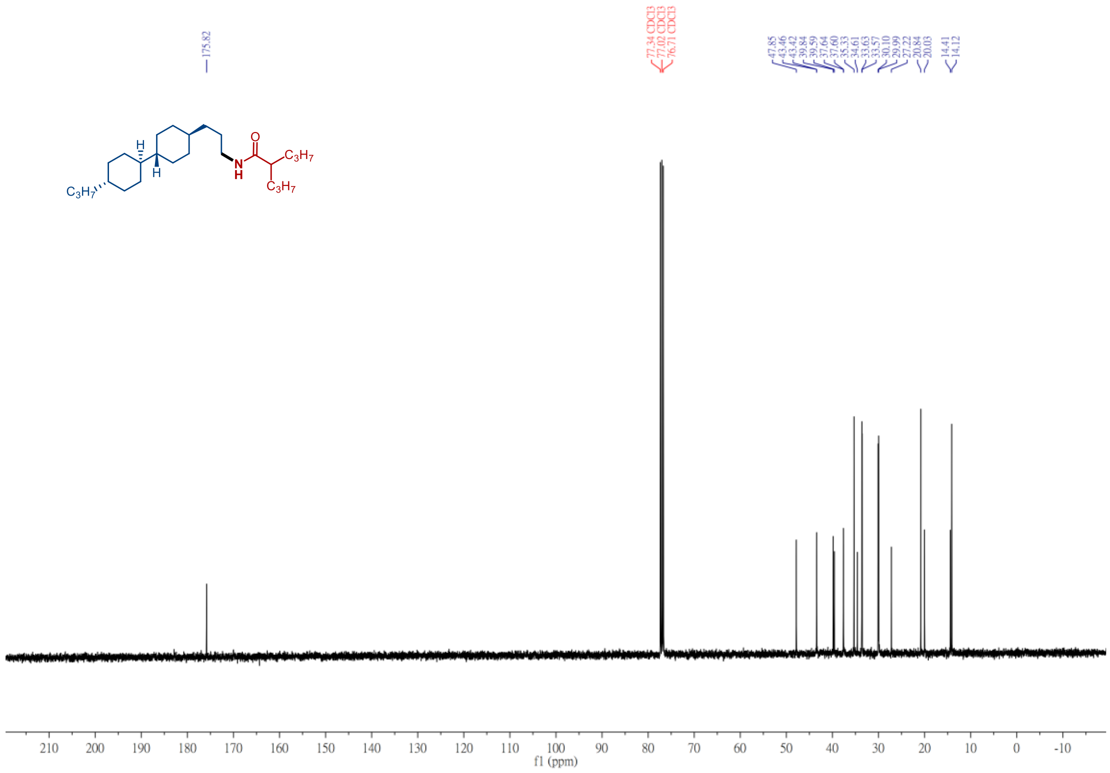

^{1}H and ^{13}C NMR spectrum of **97**

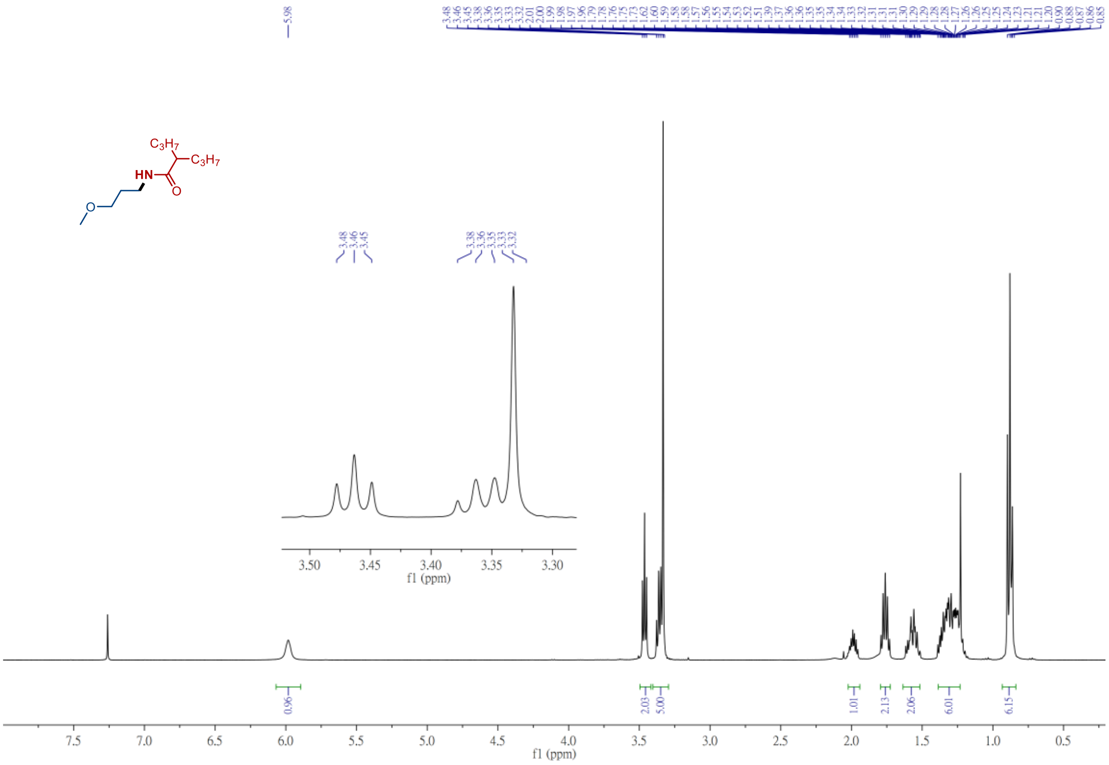

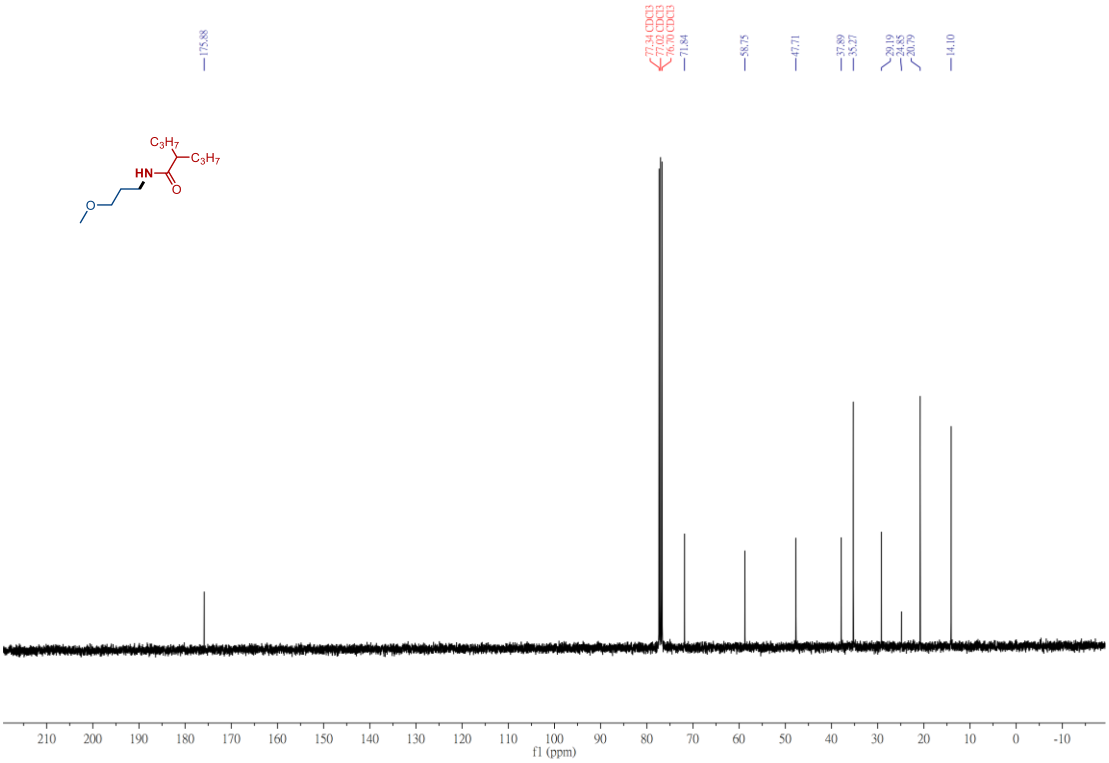

^{1}H spectrum of **98** with **99**

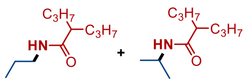

98 **99**

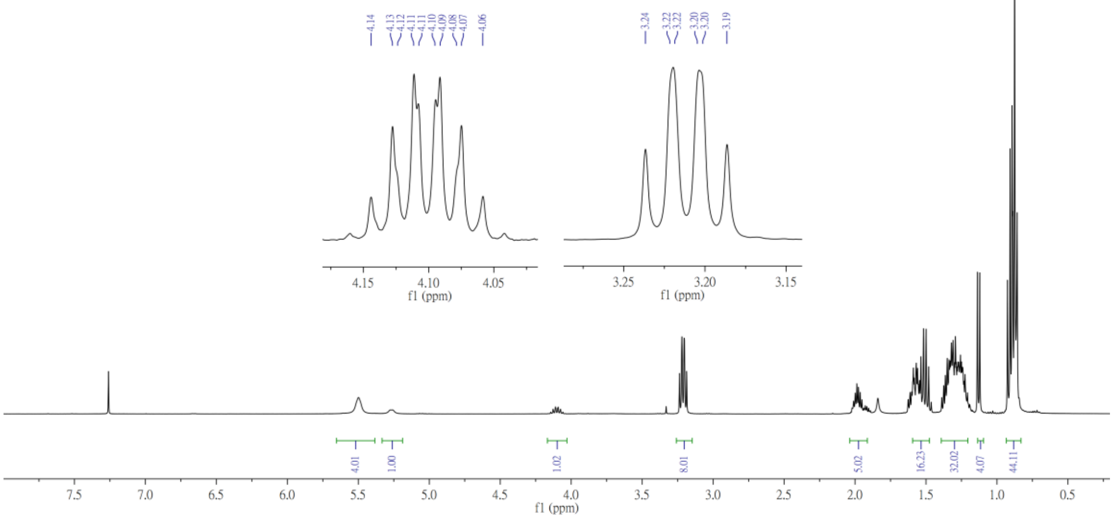

^{1}H and ^{13}C NMR spectrum of **100**

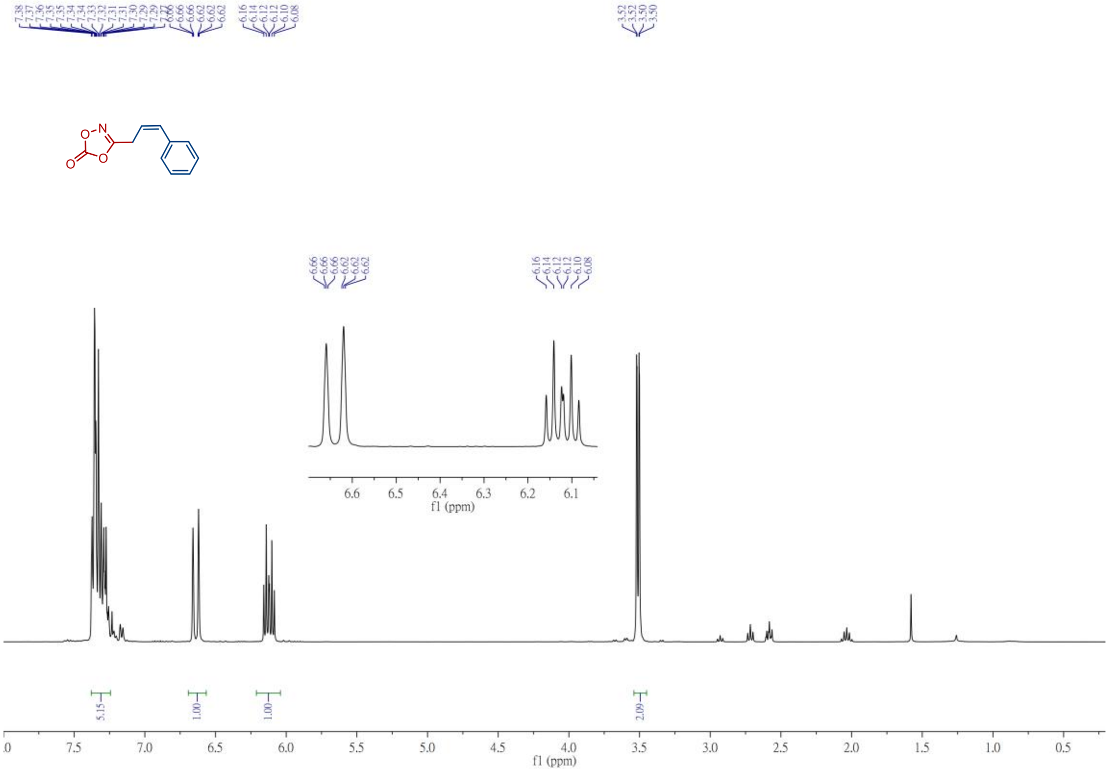

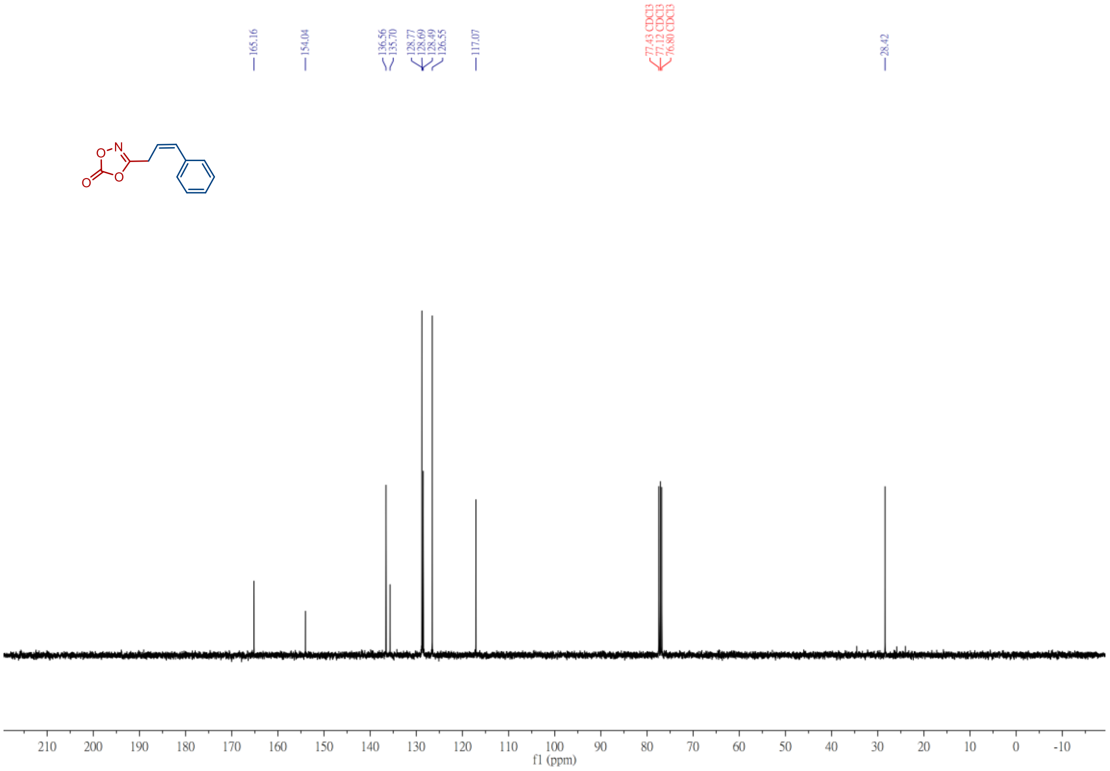

^{1}H and ^{13}C NMR spectrum of **101**

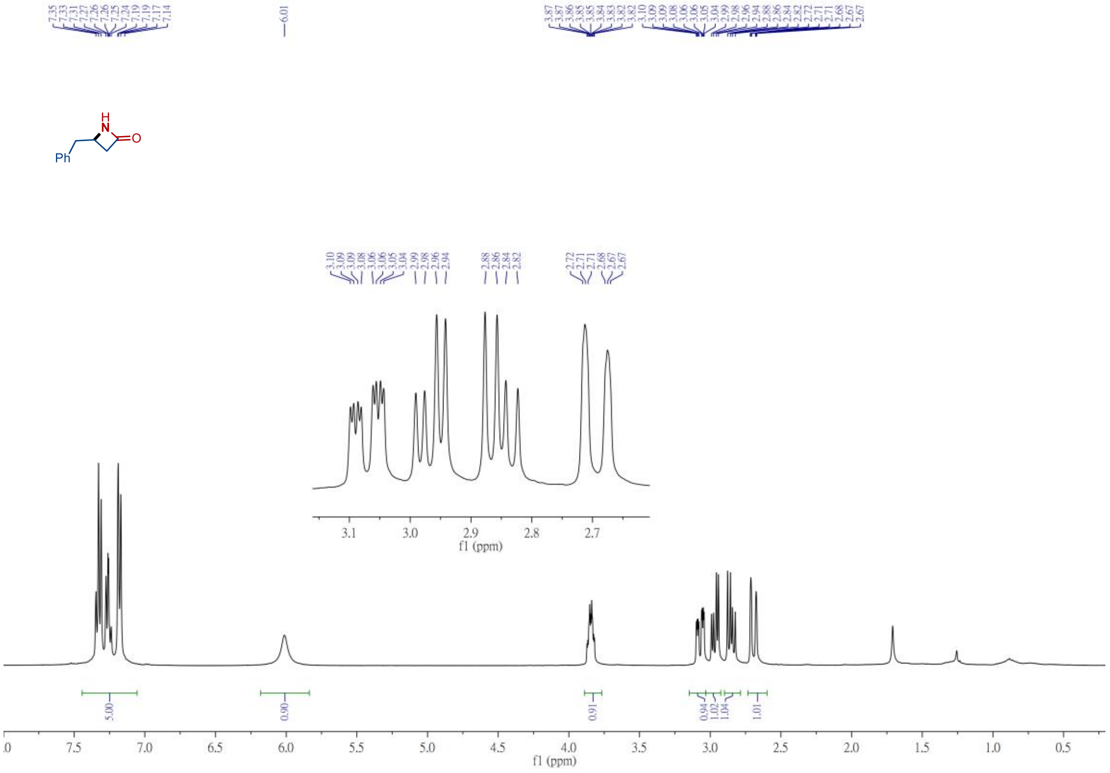

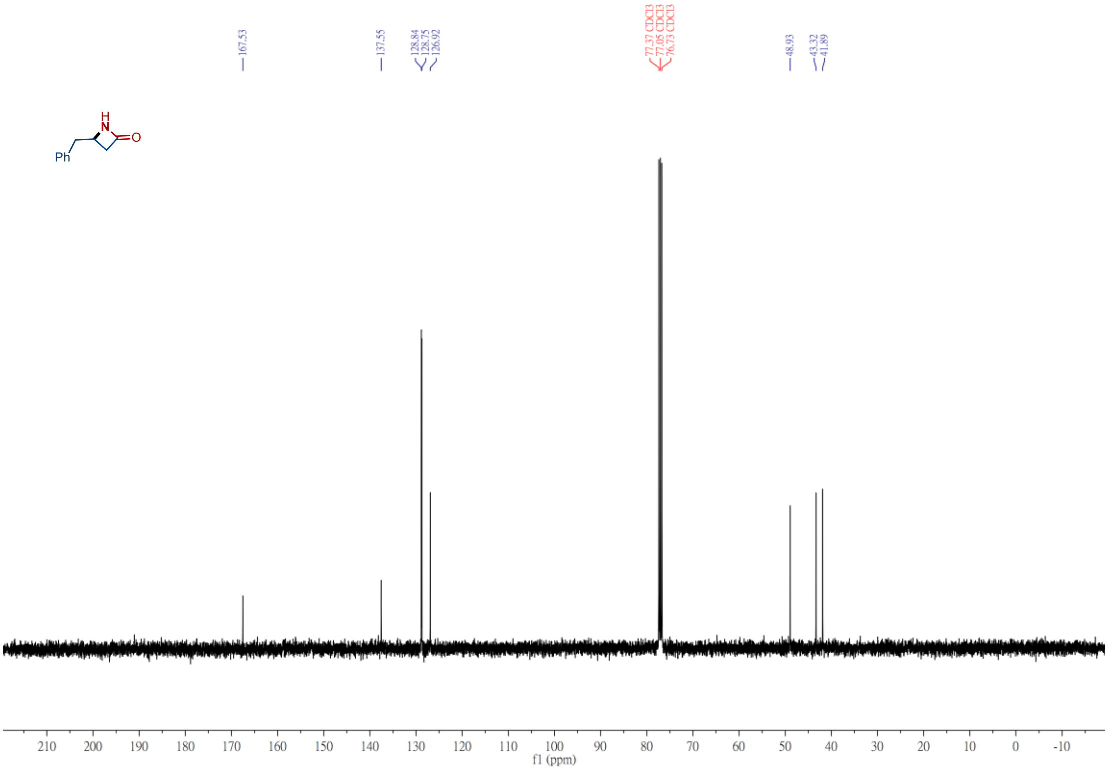

^{1}H spectrum of **122** with **123**

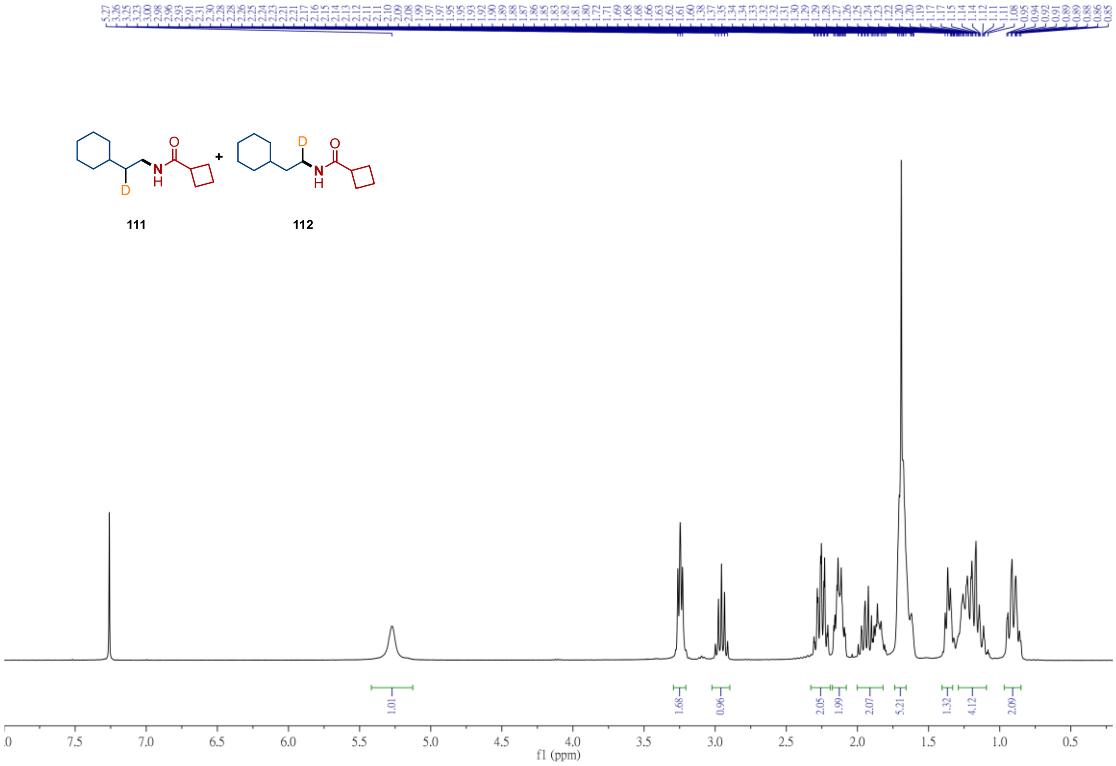

^1H and ^{13}C NMR spectrum of **S84**

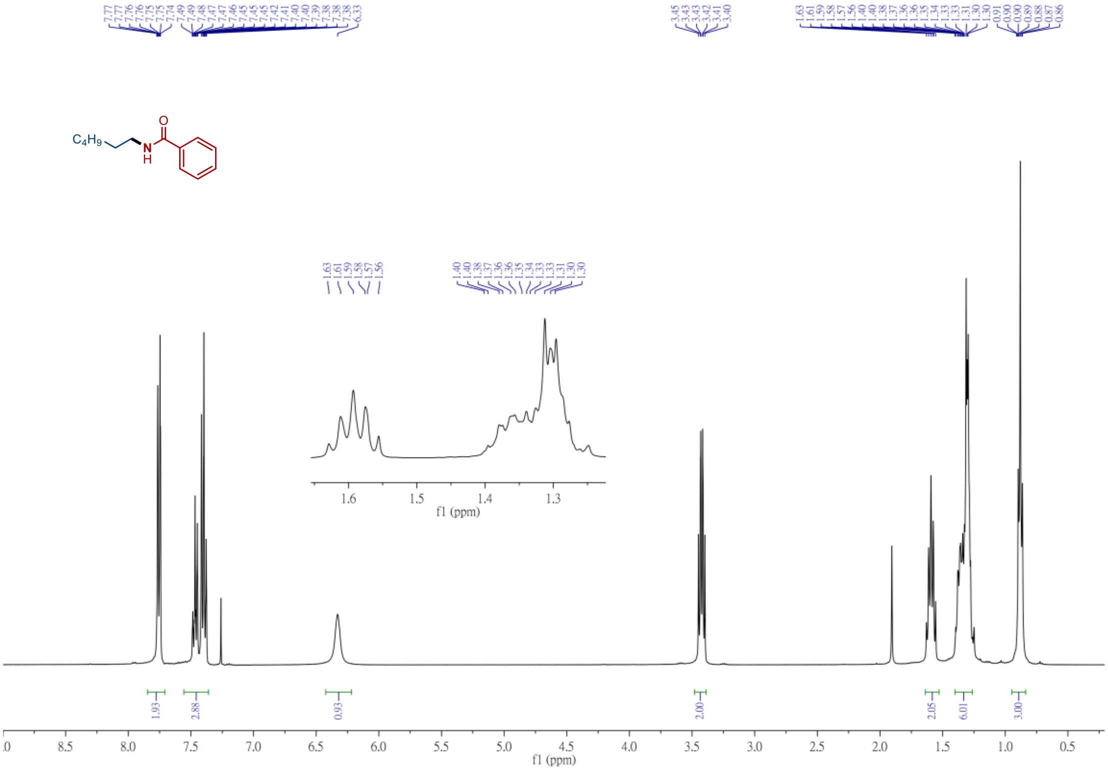

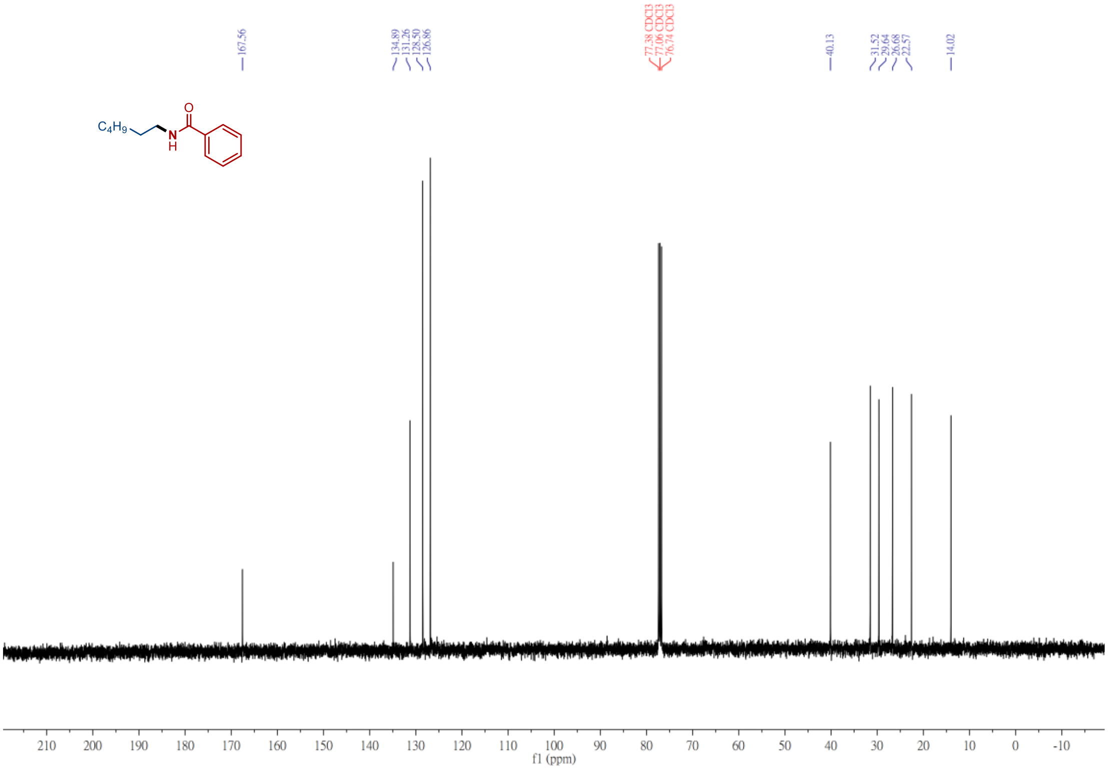

^{1}H and ^{13}C NMR spectrum of **S132**

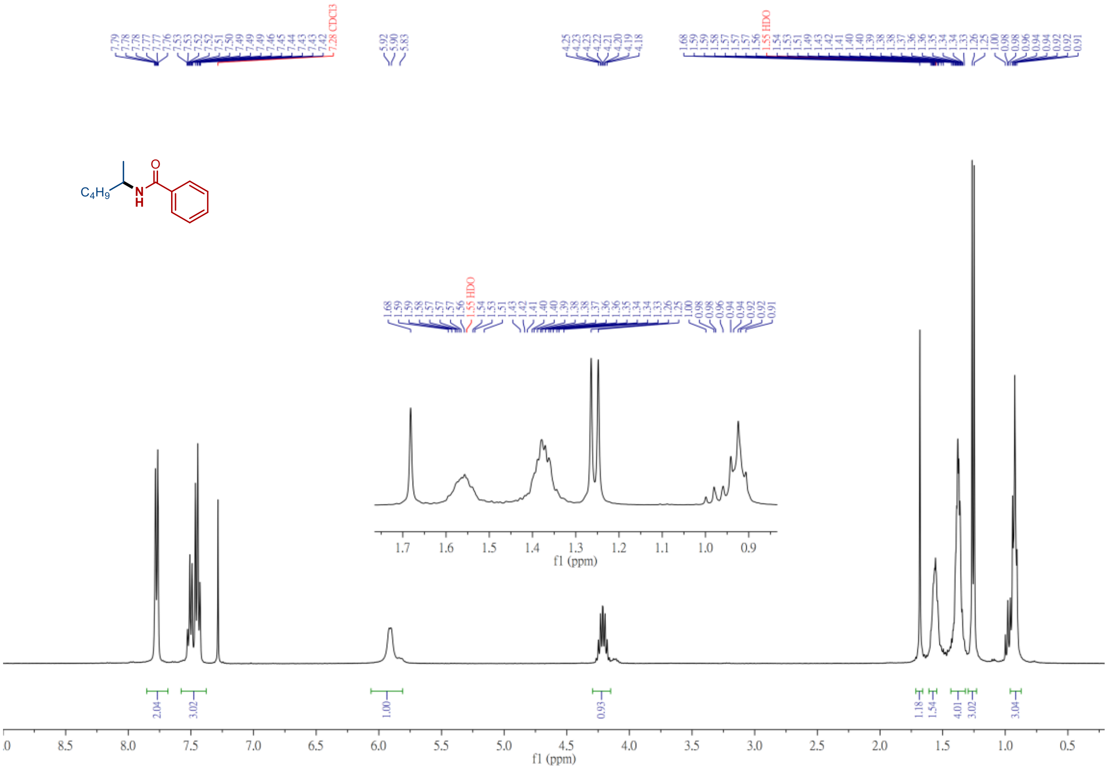

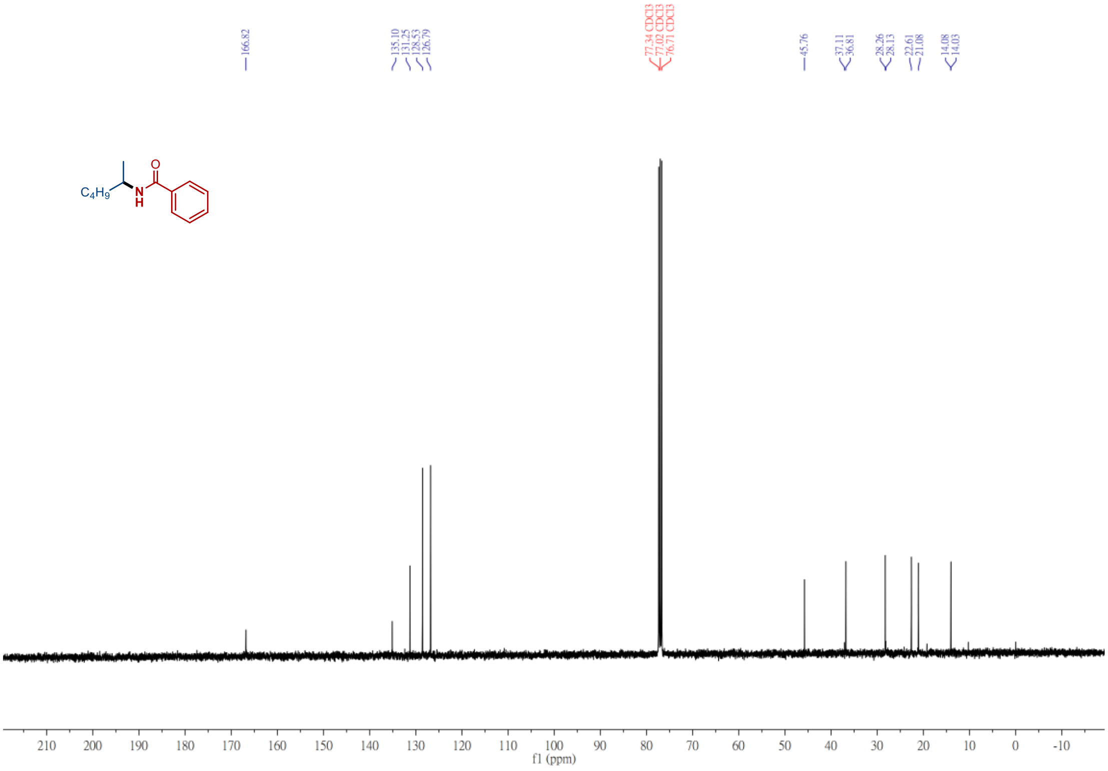

^{1}H spectrum of **S133** with **S134**

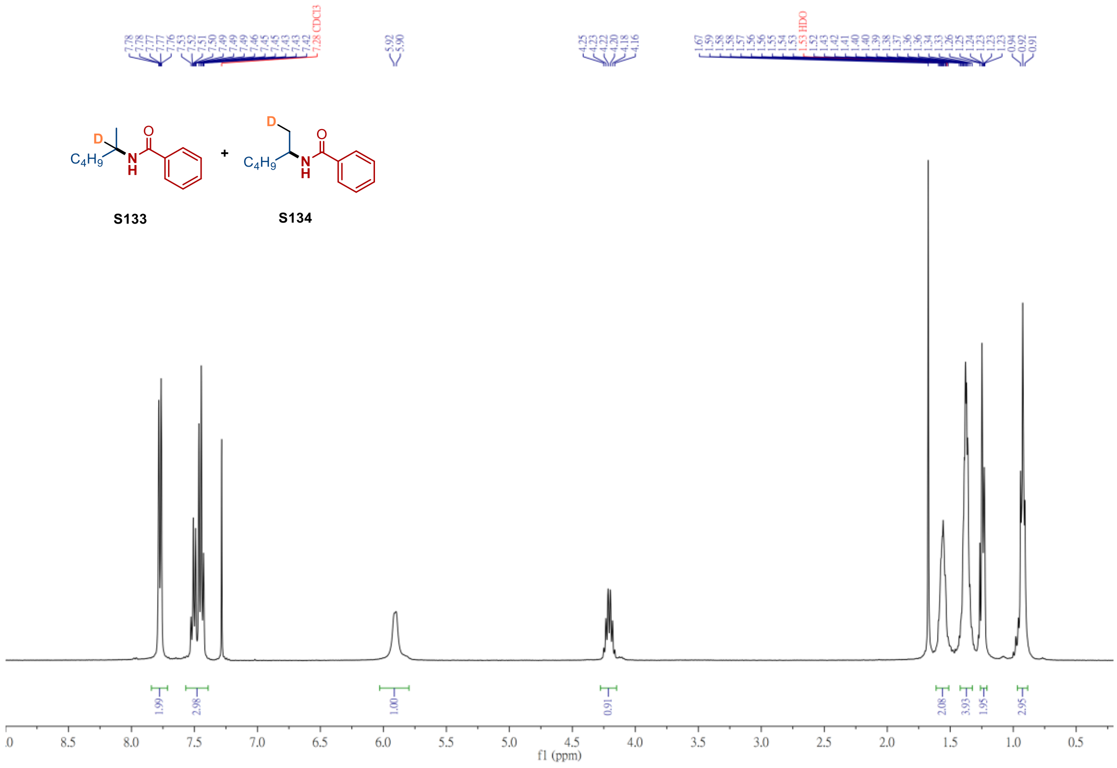

Supplement: Supplementary file 6 — Supplementary Data 3 [file 42004_2022_791_MOESM6_ESM.pdf]
